# Evolutionary Origin of the Scombridae (Tunas and Mackerels): Members of a Paleogene Adaptive Radiation with 14 Other Pelagic Fish Families

**DOI:** 10.1371/journal.pone.0073535

**Published:** 2013-09-04

**Authors:** Masaki Miya, Matt Friedman, Takashi P. Satoh, Hirohiko Takeshima, Tetsuya Sado, Wataru Iwasaki, Yusuke Yamanoue, Masanori Nakatani, Kohji Mabuchi, Jun G. Inoue, Jan Yde Poulsen, Tsukasa Fukunaga, Yukuto Sato, Mutsumi Nishida

**Affiliations:** 1 Natural History Museum and Institute, Chiba, Chiba, Japan; 2 Department of Earth Sciences, University of Oxford, Oxford, United Kingdom; 3 National Museum of Nature and Science, Tsukuba-shi, Ibaraki, Japan; 4 Atmosphere and Ocean Research Institute, The University of Tokyo, Kashiwa-shi, Chiba, Japan; 5 Natural History Collections, Bergen Museum, University of Bergen, Bergen, Norway; 6 Tohoku Medical Megabank Organization, Tohoku University, Miyagi, Japan; Fordham University, United States of America

## Abstract

Uncertainties surrounding the evolutionary origin of the epipelagic fish family Scombridae (tunas and mackerels) are symptomatic of the difficulties in resolving suprafamilial relationships within Percomorpha, a hyperdiverse teleost radiation that contains approximately 17,000 species placed in 13 ill-defined orders and 269 families. Here we find that scombrids share a common ancestry with 14 families based on (*i*) bioinformatic analyses using partial mitochondrial and nuclear gene sequences from all percomorphs deposited in GenBank (10,733 sequences) and (*ii*) subsequent mitogenomic analysis based on 57 species from those targeted 15 families and 67 outgroup taxa. Morphological heterogeneity among these 15 families is so extraordinary that they have been placed in six different perciform suborders. However, members of the 15 families are either coastal or oceanic pelagic in their ecology with diverse modes of life, suggesting that they represent a previously undetected adaptive radiation in the pelagic realm. Time-calibrated phylogenies imply that scombrids originated from a deep-ocean ancestor and began to radiate after the end-Cretaceous when large predatory epipelagic fishes were selective victims of the Cretaceous-Paleogene mass extinction. We name this clade of open-ocean fishes containing Scombridae “Pelagia” in reference to the common habitat preference that links the 15 families.

## Introduction

The upper 200 meters in coastal and oceanic waters constitute the epipelagic or euphotic zone [1]. Comprising approximately 70% of the earth’s surface, it represents one of the largest habitats [2]. The epipelagic zone is characterized by high solar insolation, variable primary production that can be very high in regions of upwelling or convergence of major currents, large volume, and a lack of physical barriers [1]. As a consequence of such a vast expanse and a patchy distribution of food in both time and space, fishes inhabiting the epipelagic realm are able to efficiently move long distances for foraging, bear streamlined bodies with forked or lunate tails, and often have special respiration and digestive systems and a high percentage of red muscle and lipids [1]. Epipelagic fishes are also commonly countershaded and silvery, frequently forming schools for foraging and for avoidance of predation [3].

Such a variety of ecological, morphological, and physiological adaptations are represented to varying degrees in approximately 850 epipelagic species of modern teleost fishes distributed across 41 families (calculated from FishBase ver. 02/2007 [4]), including herrings, sardines, anchovies, flyingfishes, halfbeaks, needlefishes, sauries, opahs, oarfishes, louvars, bluefish, Australian salmons, jacks, dolphinfishes, remoras, barracudas, cutlassfishes, mackerels, tunas, billfishes, butterfishes, and pomfrets. The most extreme examples of such adaptations, however, are found among the open water, migratory tunas, which are members of the family Scombridae (order Perciformes: suborder Scombroidei). They have the highest digestive and metabolic rates and the most profound specializations for sustained levels of rapid locomotion of any fishes [5]. Apart from these biological superlatives, scombrids are of considerable economic importance (e.g., they include the bluefin tuna, which is literally worth more than its weight in gold, and mackerels, which are heavily fished on a global scale) [6].

The evolutionary origin of the scombrids has remained unclear despite repeated attempts to resolve intra- and interrelationships of the family based on both morphological and molecular data [7]–[11]. This is largely because the taxonomic limits of the suborder Scombroidei (currently comprising six families including Scombridae [12]) have remained ambiguous since Regan [13] proposed the first modern definition of the group in the early 1900s. For example, cladistic studies of anatomical features have added Sphyraenidae (barracudas) to Scombroidei [8], while removing Luvaridae (louvar) from the group [14]. Early molecular phylogenetic studies [9], [10] questioned the affinity of scombrids with billfishes, which have been shown by more recent analyses to be close relatives of flatfishes, jacks and remoras [15], [16]. With clear evidence that the classical Scombroidei represents a polyphyletic group, there is no clear framework to guide taxon selection for analyses seeking to discover the immediate relatives of scombrids.

Continued molecular investigation has consistently revealed that scombrids form a strongly supported monophyletic group together with the perciform families Pomatomidae (bluefish) [10], [17], Bramidae (pomfrets) [17], [18], Arripidae (Australian salmons) [19], Chiasmodontidae (swallowers) [18], [20]–[23], Icosteidae (ragfish) [22], [23], Scombrolabracidae (longfin escolar) [10], Centrolophidae (medusafishes) [17]–[21], Nomeidae (driftfishes) [18], [19], [23], and Stromateidae (butterfishes) [17], [18], [20], [21]. Surprisingly, all of these families have been placed outside Scombroidei in morphological classifications, and no uniquely shared anatomical features that might unite them as a clade has been reported or recognized. However, Yagishita *et al.* [19] noticed that these non-scombroid fishes share with scombrids a pelagic ecology often associated with long-distance migrations, and suggested that future addition of other pelagic percomorph species of unknown phylogenetic affinity would further expand the limits of this distinct, previously unrecognized radiation.

**Figure 1:**
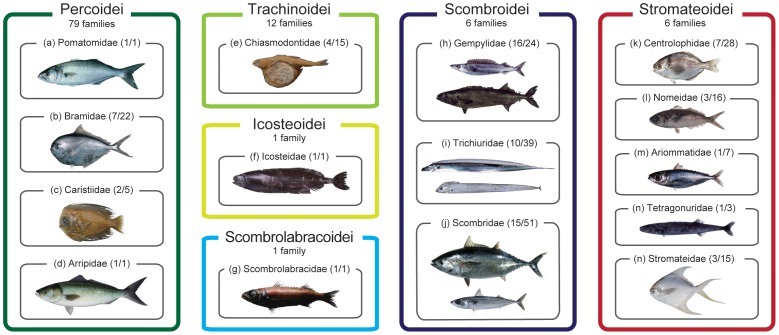
The 15 perciform families comprising the novel clade found in this study. Numerals in parentheses beside family names denote numbers of genera and species recognized in a current classification [12]. The 15 families are distributed across 6 suborders in the order Perciformes, which includes 20 suborders, 160 families, 1,539 genera, and 10,033 species [12]. (*a*) Pomatomidae (*Pomatomus saltarix*; ©State of Queensland, Department of Agriculture, Fisheries and Forestry, Australia); (*b*) Bramidae (*Brama japonica*); (c) Caristiidae (*Platyberyx macropus*); (*d*) Arripidae (*Arripis trutta*; photo courtesy of Pat Tully, © NSW Trade & Investment Primary Industries, New Zealand); (*e*) Chiasmodontidae (*Chiasmodon niger*); (*f*) Icosteidae (*Icosteus aenigmaticus*); (*g*) Scombrolabracidae **(** *Scombrolabrax heterolepis*; ©Marine Fisheries Research and Development Center, Fisheries Research Agency, Japan); (*h*) Gempylidae (*Promethichthys prometheus* and *Ruvettus pretiosus*); (*i*) Trichiuridae (*Trachurus japonicus* and *Evoxymetopon taeniatus*); (*j*) Scombridae (*Thunnus orientalis* and *Scomber australasicus*); (*k*) Centrolophoridae (*Psenopsis anomala*); (*l*) Nomeidae (*Cubiceps squamicepsi*); (*m*) Ariommatidae (*Ariomma indica*); (*n*) Tetragonuridae (*Tetraganurus cuvieri*); and (*o*) Stromateidae (*Pampus argenteus*). Photos (*b*, *f*, *h–j*, *m–o*) and (*k*, *l*) courtesy of Hiroshi Senou and Hisayuki Suzuki, respectively (©Kanagawa Prefectural Museum of Natural History).

To address the evolutionary origin of scombrids, we assembled partial nucleotide sequences of the mitochondrial (mt) and nuclear (nc) genes from all percomorphs deposited in GenBank (10,733 sequences from 5,368 species across all 13 suborders, 215 families, and 1,558 genera) and subjected them to phylogenetic analysis. The resulting phylogenies suggest that the 15 perciform families across 6 suborders (Fig. 1) constitute the least inclusive monophyletic group that contains all core members of the classical scombroid families (Gempylidae, Trichiuridae, Scombridae). Subsequent mitogenomic analyses with extensive taxon sampling from those 15 families (56 species) and outgroup taxa (124 species in total) strongly support a single origin of the 15 families. Considering the diverse modes of life of the 15 families, these results strongly suggest that there has been an undetected evolutionary radiation in the pelagic realm. This clade appears to represent a rarely documented example of adaptive radiation in a vast and apparently homogenous environment, which is in striking contrast to classic instances of adaptive radiations in small, isolated areas such as lakes or oceanic islands with diverse habitats [24].

## Results

### Bioinformatic Analysis

**Figure 2:**
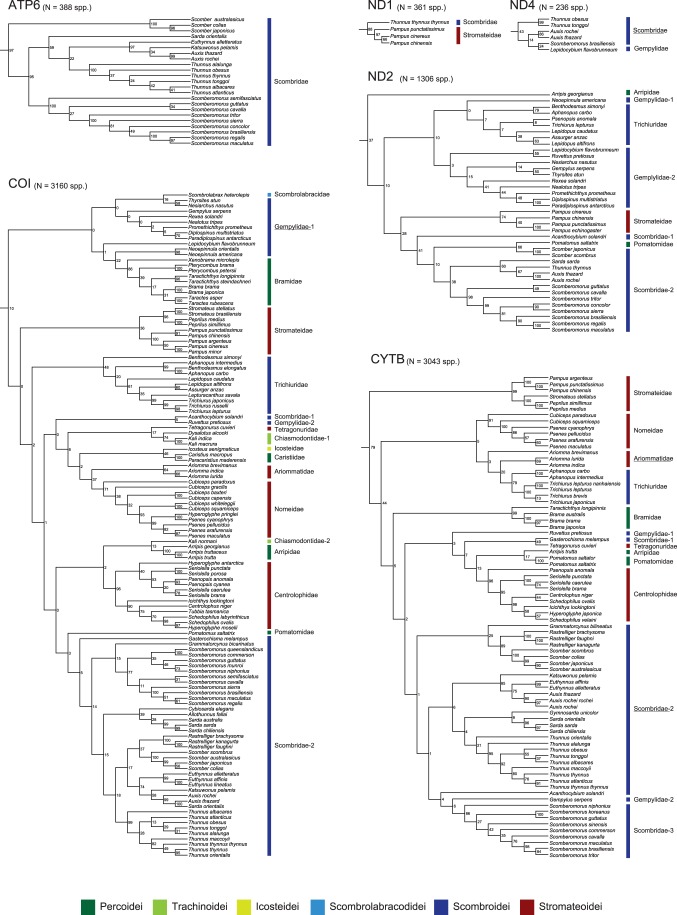
The best-scoring maximum likelihood trees based on 6 partial mitochondrial genes downloaded from GenBank. Only a portion of Pelagia shown. Numerals beside internal branches indicate bootstrap proportions based on 1000 replicates. Subordinal groupings of the 15 families are colored following Fig. 1. All tree files are available in Text S2.

**Figure 3:**
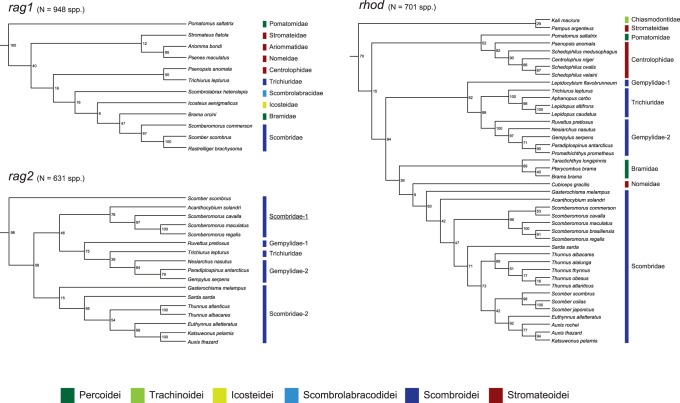
The best-scoring maximum likelihood trees based on 2 partial nuclear genes downloaded from GenBank. Only a portion of Pelagia shown. Numerals beside internal branches indicate bootstrap proportions based on 1000 replicates. Subordinal groupings of the 15 families are colored following Fig. 1. All tree files are available in Text S2.

A total of 10,731 sequences downloaded from GenBank were sorted into 6 mitochondrial (mt) and 3 nuclear (nc) genes (only protein-coding genes with >100 species) and those sequences together comprise 5,367 species (35% of total percomorph species diversity) distributed across 215 families (87%) and 1,558 genera (64%). Partitioned maximum likelihood (ML) analyses of the individual genes found that 12 perciform (non-scombroid) families are members of the least inclusive monophyletic group that contains all core members of the classical scombroid families (Gempylidae, Trichiuridae, Scombridae) and none of these 15 families are found outside this clade (Table 1; Figs. 2, 3). Total number of families covered and bootstrap percentages (BPs) supporting this clade vary greatly, ranging from one (mt *ATP6*) to 15 (mt *COI*) and from 10% (mt *COI*) to 100% (nc *rag1*), respectively (Table 1; Figs. 2, 3).

**Table 1:** Summary of species diversity and taxonomic coverage of the 15 families in the bioinformatic and mitogenomic analyses in this study.

Suborder^a^	Family^a^		Genus^b^	Species^b^	Mitochondrial genes^c^						Nuclear genes^c^			Mito-genomes
					*ATP6*	*COI*	*Cytb*	*ND1*	*ND2*	*ND4*	*rag1*	*rag2*	*rhod*	
Percoidei	Pomatomidae	1		1	–	1	2	–	1	–	1	–	1	1
	Bramidae		7	22	–	9	4	–	–	–	1	–	3	4
	Caristiidae		2	5	–	2	–	–	–	–	–	–	–	1
	Arripidae		1	4	–	3	1	–	1	–	–	–	–	1
Trachinoidei	Chiasmodontidae		4	15	–	***4***	–	–	–	–	–	–	1	3
Icosteoidei	Icosteidae		1	1	–	1	–	–	–	–	1	–	–	1
Scombrolabracoidei	Scombrolabracidae		1	1	–	1	–	–	–	–	1	–	–	1
Scombroidei	Gempylidae		16	24	–	***12***	***2***	–	***11***	***1***	–	***4***	***6***	***9***
	Trichiuridae		10	39	–	11	6	–	7	–	1	1	4	6
	Scombridae		15	51	23	***42***	***38***	1	***15***	***5***	3	***12***	20	18
Stromateoidei	Centrolophidae		7	28	–	13	9	–	–	–	1	–	5	3
	Nomeidae		3	16	–	11	6	–	–	–	1	–	1	2
	Ariommatidae		1	7	–	3	***3***	–	–	–	1	–	–	2
	Tetragonuridae		1	3	–	1	1	–	–	–	–	–	–	2
	Stromateidae		3	15	–	9	6	–	4	–	1	–	1	3
Total			73	232	23	123	78	4	39	6	12	17	42	57
Total number of families covered					1	15	11	2	6	2	10	3	9	15
Bootstrap probability for Pelagia (%)					97	10	79	88	43	43	100	98	70	100
Total number of percomorph species covered					388	3,160	3,042	360	1,306	234	1,073	639	753	121
Sequence length (bp) in the aligned data					666	1,536	1,119	972	1,044	1,380	1,515	1,185	1,035	13,506
Missing data (%)					2.8	52.0	17.2	7.9	4.0	43.0	12.4	29.0	31.5	0.1

a Classification and ^b^number of genera and species follow “*Fishes of the World*” [12]; ^c^ Italicized bold face fonts are those families reproduced as non-monophyletic groups.

Of those 12 non-scombroid families, a total of nine families have formed monophyletic groups with the scombrids in previous molecular phylogenetic studies [10], [18]–[23], but the placements of the remaining three (Caristiidae, Ariommatidae, Tetragonuridae) represent new discoveries (Table 1; Fig. 2). It should be noted that the two scombroid families (Gempylidae, Scombridae) were not reproduced as monophyletic across several genes when more than two or three species were analysed (Table 1; Figs. 2, 3). It appears that the lack of sufficient taxon sampling across genes and weak phylogenetic signal in some of the genes (particularly mt *COI* gene with only 10% BP; Table 1; Fig. 2) prevent us from drawing explicit conclusions about single-origin hypotheses for the 15 families generally and these two scombroid families specifically.

### Mitogenomic Analysis

Based on the results from the above bioinformatic analysis, we generated new whole mitogenome sequences for 37 species for the 15 targeted families and 17 species from the outgroup (total 54 species). The genome content of the 54 species includes 2 rRNA, 22 tRNA, and 13 protein-coding genes, plus the putative control region, as found in other vertebrates. Gene arrangements are identical to the typical mitochondrial gene order of other vertebrates.

**Figure 4:**
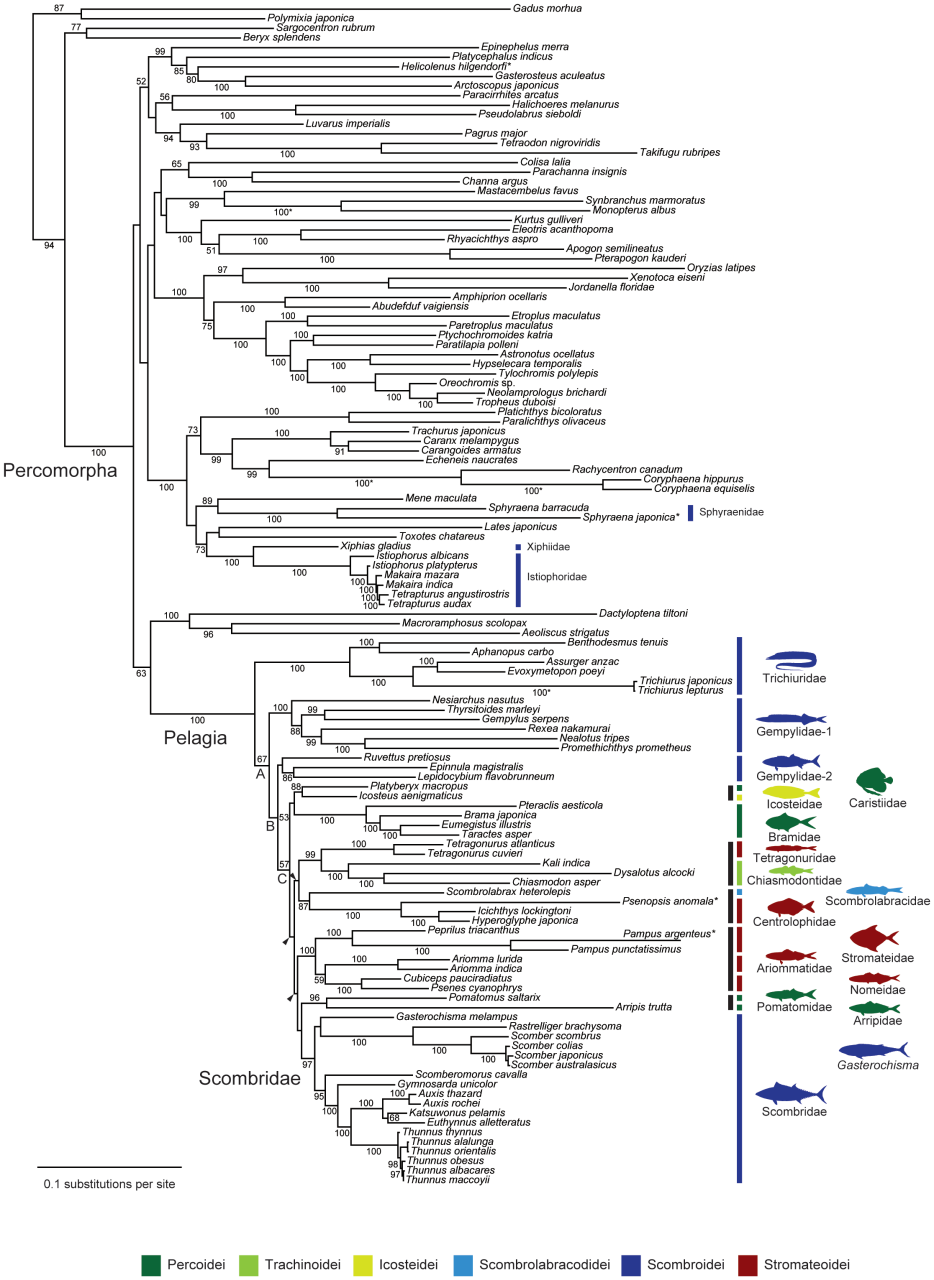
The best-scoring maximum-likelihood (ML) tree of the 124 species based on unambiguously aligned whole mitogenome sequences (12_n_3_r_RT_n_ dataset; 13,506 positions; ln *L = *–365160.5637). Numerals beside internal branches indicate bootstrap proportions (BPs) of ≥50% based on 1000 replicates. Arrowheads indicate those internal branches that are collapsed when a strict consensus tree is constructed from the best-scoring ML trees derived from 2 different data sets that include 3rd codon positions (12_n_3_r_RT_n_ and 123_n_RT_n_ datasets). Terminal and internal branches with asterisks indicate those shortened to a half of the original ones for a practical purpose. Subordinal grouping of the 15 families are colored following Fig. 1 and black bars denote 5 trans-familial clades supported by BPs of ≥87%. A tree file is available in Text S2.

**Figure 5:**
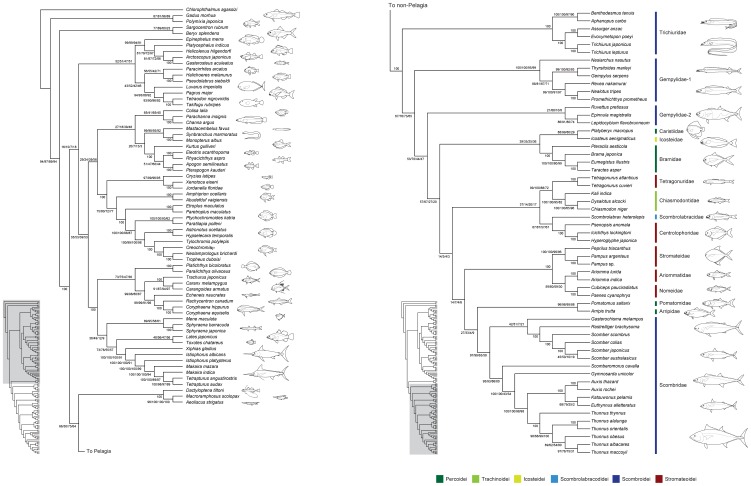
The best-scoring maximum likelihood trees based on whole mitogenome sequences from the 12_n_3_r_RT_n_ data set with bootstrap proportions (BPs) from the 4 data sets. BPs are based on 1000 replicates and those of ≥50% only indicated with the following sequences (12_n_3_r_RT_n_,123_n_RT_n_, 12_n_RT_n_, 123_a_RT_n_).

RAxML analysis of the 12_n_3_r_RT_n_ data set results in the best-scoring ML tree with a likelihood score of –365160.5637 (Fig. 4). Monophyly of the least inclusive monophyletic group that contains all core members of the classical scombroid families (hereafter called “Pelagia”; for etymology and definition, see below) is strongly supported with bootstrap percentages (BPs) of 100% across the 4 data sets (Fig. 5). As in previous molecular studies [15], [16], the 2 billfish families (Xiphiidae and Istiophoridae) and barracudas (Sphyraenidae) fall outside Pelagia, exhibiting more recent common ancestry with flatfishes, carangoids, and several “generalized” perciforms such as *Lates* and *Toxotes* (Figs. 4, 5). Monophyly of the textbook “Scombroidei” [12] (Xiphiidae, Istiophoridae, Sphyraenidae, Gempylidae, Trichiuridae, Scombridae) is confidently rejected by the approximately unbiased (AU) test across the four data sets (*P*<0.000).

Of the 15 pelagic families analyzed, 10 families include multiple species and are recovered as monophyletic with the exception of the Gempylidae, which is consistently recovered as paraphyletic grade consisting of two subclades (Gempylidae–1 and 2 in Figs. 4, 5). Monophyly of each of the remaining nine families is strongly supported (BPs = 97–100%) in the two nucleotide data sets that include 3rd codon positions (12_n_3_r_RT_n_ and 123_n_RT_n_ data sets), although exclusion of the entire 3rd codon positions or conversion of the 12 protein-coding genes into amino acid sequences occasionally lower the statistical support for these families (e.g., BP = 58% for the Scombridae in the 123_a_RT_n_ data set; Fig. 5). We interpret this as indicating lack of phylogenetic signal in the latter two data sets as observed in many groups of organisms [25], including fishes [26]. Accordingly the following description of the results and discussions on the interrelationships of Pelagia are based on the former two nucleotide data sets (12_n_3_r_RT_n_ and 123_n_RT_n_) only.

Within Pelagia, Trichiuridae is the earliest diverging lineage and is followed by Gempylidae–1 (Figs. 4, 5). Although monophyly of the three deepest, most inclusive clades (A–C in Fig. 4) are consistently recovered in the two data sets, bootstrap proportions for these groups are not high, ranging from 53% (Clade B in the 12_n_3_r_RT_n_ data set) to 76% (Clade A in the 123_n_RT_n_ data set). Clade C contains the remaining 13 families. Inter-familial relationships within Clade C that receive high statistical support include sister-group relationships between Icosteidae and Caristiidae (BPs = 88, 98%), Tetragonuridae and Chiasmodontidae (99, 100%), Scombrolabracidae and Centrolophidae (87, 87%), Stromateidae and Ariommatidae plus Nomeidae (100, 100%), and Pomatomidae and Arripidae (96, 98%) (Fig. 5). Interrelationships among these clades, however, are quite ambiguous and even the two similar data sets recover quite different relationships as indicated by the three arrowheads in Fig. 4. Although two suborders (Scombroidei and Stromateoidei) and one family (Gempylidae) are not recovered as monophyletic, AU tests are unable to reject monophyly of these groups (*P* = 0.109–0.349) with the exception of that of Stromateoidei in the 12_n_3_r_RT_n_ dataset (*P* = 0.005; Table 2).

**Table 2:** Statistical comparisons between the non-constrained and constrained ML tree using AU test for the three nucleotide data sets.

Family/Suborder	Dataset	Best LogLik	Constrained LogLik	Difference	AU p-value
Gempylidae	12_n_3_r_RT_n_	−365160.5637	−365176.6314	−16.0677	0.215
	123_n_RT_n_	−584499.1272	−584522.9874	−23.8603	0.349
	12_n_RT_n_	−217998.3486	−218026.8734	−28.5248	0.113
Scombroidei	12_n_3_r_RT_n_	−365160.5637	−365182.7665	−22.2028	0.333
	123_n_RT_n_	−584499.1272	−584537.3541	−38.2269	0.346
	12_n_RT_n_	−217998.3486	−218031.5529	−33.2043	0.113
Stromateoidei	12_n_3_r_RT_n_	−365160.5637	−365228.4912	−67.9275	0.005
	123_n_RT_n_	−584499.1272	−584522.9884	−23.8612	0.233
	12_n_RT_n_	−217998.3486	−218026.8736	−28.525	0.109

The Scombridae is recovered as monophyletic with strong statistical support (BPs = 97, 99%) and occupies an apical position, and is placed as sister to a clade comprising two families (Pomatomidae and Arripidae) (Fig. 4). This sister-group relationship, however, receives weak statistical support (BP<50%) in the two data sets where it is resolved, and is not found in analyses of the remaining two data sets.

### Divergence Time Estimation and Character Mapping

**Figure 6:**
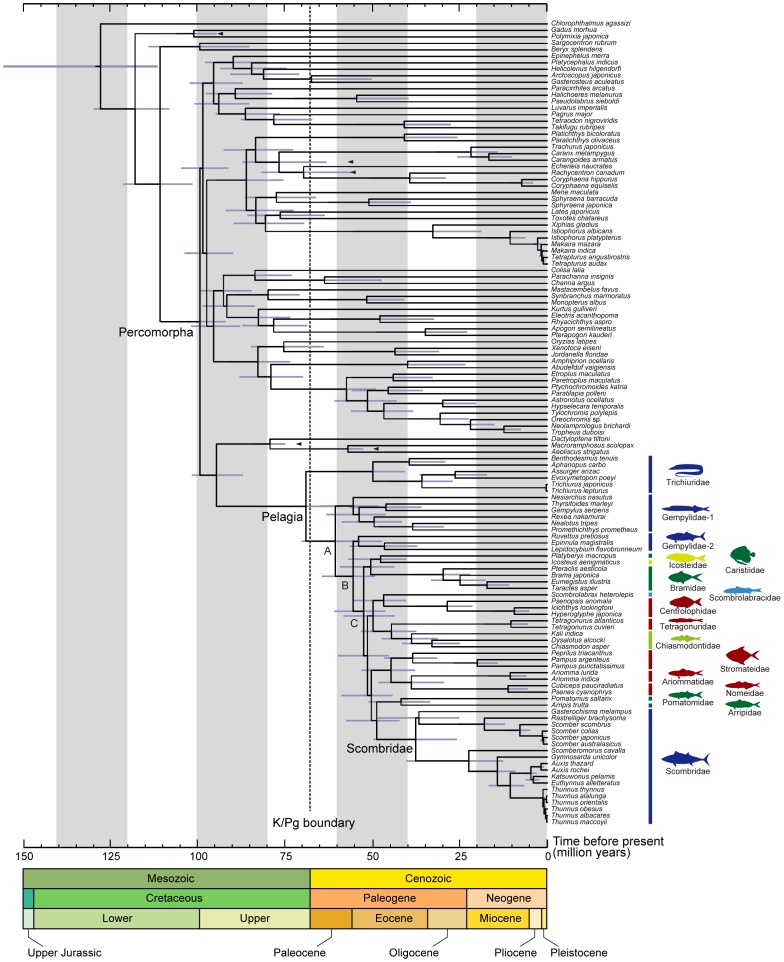
Timetree of Pelagia and outgroups derived from the Bayesian relaxed-molecular clock method using MCMCTREE implemented in PAML v. 4.5 [58]. Five nodes were used for time constraints based on fossil record (arrowheads; for details, see Table 2). Horizontal bars indicate 95% credible intervals of the divergence time estimates.

MCMCTREE analyses of divergence times with our five well-supported fossil calibrations indicate that Pelagia began to diversify around the latest Cretaceous (69.1 Ma) with a 95% credible interval of 59.4–80.1 Ma (Fig. 6). The basal divergences that led to the origin of modern scombrids were estimated to occur in a relatively small time window during the early Paleogene (48.9–60.7 Ma), with crown scombrids beginning to diversify during the middle Paleogene around 37.7 Ma with a 95% credible interval of 25.9–49.9 Ma (Fig. 6). With the addition of three further constraints based on concordance between the sequence of Gondwanan breakup and patterns of cichlid divergences, MCMCTREE analyses consistently yield older estimates (absolute differences in posterior means: 22.5 million years ±15.9 SD for all nodes and 15.9 million years ±10.9 SD for those nodes within Pelagia). In both estimates, onset of the diversification of crown scombrids is estimated to postdate the Late Cretaceous (37.7 and 60.2 Ma in posterior means, respectively; Table S1).

The timescales described above were intentionally estimated without any fossil calibrations within Pelagia. This strategy reflects a desire to produce divergence-time estimates within Pelagia that are as independent as possible from the fossil record of the clade, and which can be compared to a purely paleontological timescale for the evolution of the group.

**Figure 7:**
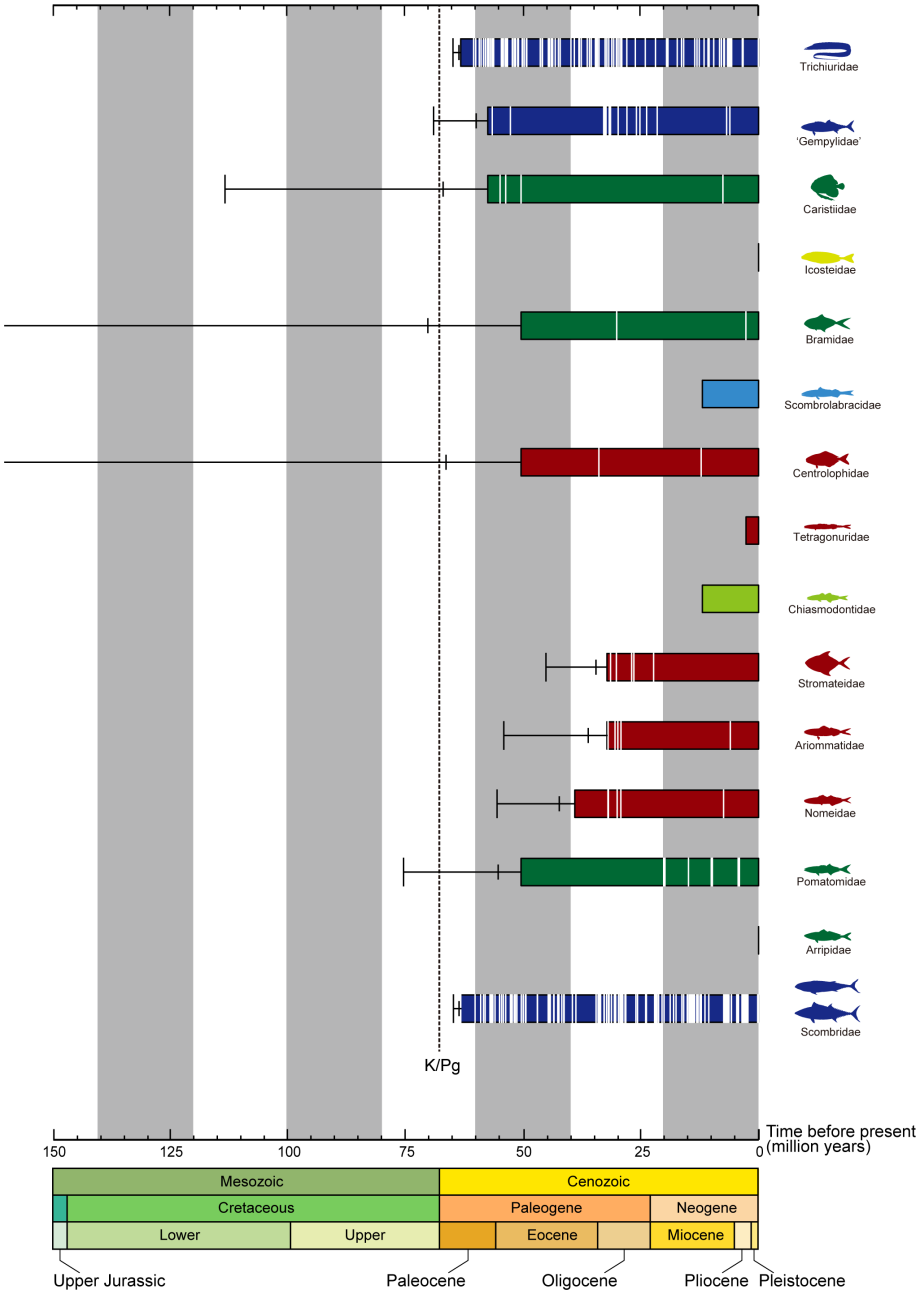
Summary of the fossil record of Pelagia, indicating rapid appearance of constituent lineages in the Paleogene. Solid bars represent known stratigraphic range, with white lines showing approximate distribution of horizons yielding body-fossil remains of groups (horizons within individual stratigraphic intervals are randomly distributed for clarity). One-tailed 95% (wide bracket) and 50% (narrow bracket) confidence intervals on the first appearance of each group assuming uniform preservation potential are indicated. Color-coding of classical taxonomic groups to which families have been assigned follows that used in Fig. 1. For fossil horizons yielding the 15 families of Pelagia, see Table S4.

Our paleontological timescale for Pelagia rests on a compendium of occurrences of members of the clade based on body-fossil remains (Fig. 7; see below in Materials and Methods). No formal cladistic analysis of fossil Pelagia has been published, so our data are coarsely sorted into divisions corresponding to the total groups for each of the 15 families that we place within this radiation. Icosteidae and Arripidae have no fossil record, while Scombrolabracidae, Tetragonuridae, and Chiasmodontidae are represented only by a single fossil horizon each (Text S1). The poor fossil records of these particular families is unsurprising, as they are characterized by low species richness, inhabit deep water environments with poor fossil records, or a combination of both.

**Figure 8:**
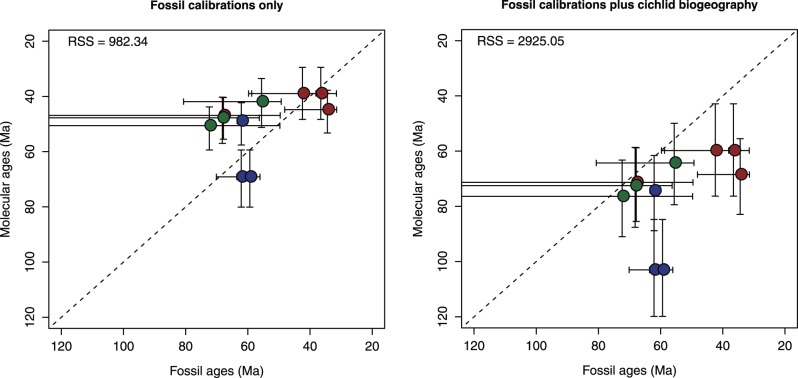
Comparison of paleontological timescales for the evolution of Pelagia with molecular clock estimates using five well-constrained fossil calibrations representing taxa outside of Pelagia (left) and with those same fossil calibrations plus additional calibrations derived from a vicariance hypothesis of cichlid biogeography (right). Points are specified by the paleontological maximum-likelihood estimate for the origin of the total-group families for which this value can be calculated and the mean molecular age estimate for that same lineage. Error bars represent 95% confidence intervals. Color-coding of classical taxonomic groups to which families have been assigned follows that used in Fig. 1. RSS = residual sum of squares measuring deviation from a hypothesized 1∶1 relationship. For paleontological timescale for the 15 families of Pelagia, see Table S5.

Although the 10 remaining families of Pelagia have much denser paleontological records, the oldest fossil occurrences of these groups necessarily postdate their true times of evolutionary origin. We therefore calculated confidence intervals and unbiased maximum-likelihood point estimates for the evolutionary appearance of these groups using simple quantitative biostratigraphical models that assume uniform preservation potential over time [27], [28] (see below in Materials and Methods). We compared our point estimates [27] for the time of origin for each of these families based on fossil evidence alone with our two molecular time estimates for the corresponding groups made without reference to the fossil record of Pelagia (i.e., neither these point estimates nor any other information about the fossil record of Pelagia was used in calibrating our molecular phylogenies). We quantified disagreement between the two timescales for the evolution of Pelagia using the residual sum of squares from 1∶1 a relationship. Molecular ages generated using only fossil-based calibrations show a much closer correspondence to paleontological estimates for the time of origin of groups within Pelagia than do molecular age estimates that use putative vicariant events in cichlid evolutionary history in addition to fossil specimens as calibration points (residual sum of squares: 982.34 vs 2925.05; Fig. 8). In the following, we use the molecular divergence time estimates using fossil calibrations only, noting that these age estimates are also more concordant with recent molecular studies of actinopterygian divergence times based on nuclear genes [16], [17].

**Figure 9:**
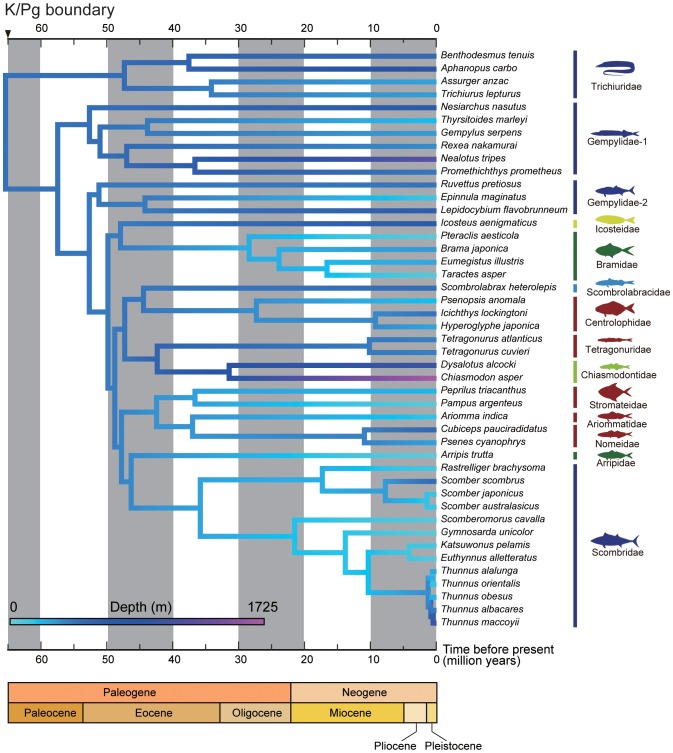
Mean depth ecology of Pelagia reconstructed on the timetree (Fig. 6). Depths are the averages of minimum and maximum depths reported in FishBase. Depth ecologies for internal nodes are estimated using maximum likelihood. The last common ancestor of Pelagia is inferred to have been mesopelagic. The invasion of the epipelagic realm by scombrids and other groups of Pelagia occurred in the early Paleogene.

Mapping of depth preferences on to our time-calibrated hypothesis of interrelationships of Pelagia allows us to infer shifts in depth ecology during the evolutionary history of the group (Fig. 9). Two striking features emerge regardless of the molecular timetree used. First, the last common ancestor of Pelagia is reconstructed as being mesopelagic, with an inferred mean depth range of approximately 470 m for both sets of molecular divergence estimates. Second, we infer that Scombridae has arisen from a deeper-living ancestor, and the shift to shallower depths (<300 m) in this and some other groups of Pelagia (e.g., stromateids, ariommatids, arripids, and bramids) took place in the Paleogene. This result is clearly not conclusive due to weak support for the deepest divergences within Pelagia combined with the considerable uncertainty that typically surrounds ancestral state estimates. However, we highlight this hypothesis here in the hope that future analyses will test it with more comprehensive datasets.

## Discussion

The evolutionary origin of the family Scombridae has proven enigmatic because of the unstable limits of the suborder Scombroidei within the order Perciformes [8], [14], [29], with the consequence that no clear framework existed to guide taxon selection to discover immediate relatives of scombrids. Our bioinformatic and mitogenomic analyses, however, successfully circumscribe a novel clade comprising 15 families including Scombridae (Fig. 1) with strong statistical support across four different data sets (BPs = 100%; Fig. 5). Monophyly of these 15 families would not be predicted from the current morphological classifications [12], where they are placed in six different perciform suborders for which no common morphological specializations have been recognized with the exception of three core members of the classical Scombroidei (Gempylidae, Trichiuridae, Scombridae) and two putative scombroid relatives (Pomatomidae, Scombrolabracidae) [7], [8]. Of the remaining 10 non-scombroid families, previous molecular studies [18]–[23] have shown that 3–7 of these taxa (Bramidae [18]; Arripidae [19]; Chiasmodontidae [18], [20]–[22]; Icosteidae [22], [23]; Centrolophidae [18]–[21]; Nomeidae [18], [19], [23]; Stromateidae [18], [20]–[22]), in various combinations, form strongly supported monophyletic groups with or without scombrids (BPs = 91–100% except for 55% in [21]). The present analysis adds three new families (Caristiidae, Ariommatidae, Tetragonuridae) as members of this novel clade (Table 3), providing the first comprehensive picture of this previously unrecognized radiation that includes scombrids.

**Table 3:** Summary of the previous molecular studies for taxon sampling and statistical support for Pelagia.

Suborder	Family	Chen*et al*. [20]	Orrell*et al*. [10]	Smith & Craig [22]	Dettai &Lecointre [21]	Li *et al*. [18]	Yagishita*et al*. [19]	Wainwright*et al*. [23]	Total
Percoidei	Pomatomidae	–	•	–	–	–	–	–	1
	Bramidae	–	–	–	–	•	–	–	1
	Caristiidae	–	–	–	–	–	–	–	–
	Arripidae	–	–	–	–	–	•	–	1
Trachinoidei	Chiasmodontidae	•	–	•	•	•	–	–	4
Icosteoidei	Icosteidae	–	–	•	–	–	–	•	2
Scombrolabracoidei	Scombrolabracidae	–	•	–	–	–	–	–	1
Scombroidei	Gempylidae	–	•	–	–	–	–	•	2
	Trichiuridae	–	•	–	–	•	–	•	3
	Scombridae	•	•	–	•	•	•	•	6
Stromateoidei	Centrolophidae	•	–	–	•	•	•	–	4
	Nomeidae	–	–	–	–	•	•	•	3
	Ariommatidae	–	–	–	–	–	–	–	–
	Tetragonuridae	–	–	–	–	–	–	–	–
	Stromateidae	•	–	•	•	•	–	–	4
Number of families sampled		4	5	3	4	7	4	5	12
Statistical support (BP/JP%)^a^		97	95	95	55	91	100	100	
Number of orders sampled		3	3	3	3	4	3	3	

Dots and dashes represent sampled and unsampled taxa in these studies, respectively.

a BP = bootstrap proportion; JK = jackknife proportion (only for Smith & Craig [22]).

Our taxon sampling, however, is incomplete with respect to perciform groups and there might still be unrecognized members of Pelagia among these unsampled families. The monotypic stromateoid family Amarsipidae represents the most likely candidate member of this clade, considering all the other five stromateoid families are confidently placed within Pelagia (Figs. 4, 5). However, this rare, monotypic family exhibits a unique combination of meristic and morphological characters among stromateoids [30], and lacks a pharyngeal sac, a unique morphological specialization observed throughout the remaining five stromateoid families [31]. The remaining 15 perciform families not included in our bioinformatic analysis (Table 4) are either small benthic fishes (mean maximum length = 2.5–16.2 cm) or medium to large reef-associated fishes (52.9–205.0 cm), implying distant common ancestry with Pelagia (see below).

**Table 4:** A list of 16 unsampled perciform families in the bioinformatic analyses and associated biological information.

Family	Genus	Species	Max length (cm)	Ecology
Perciliidae	1	2	9.3±0.4	freshwater
Symphysanodontidae	1	5	14.0±3.3	marine
Polyprionidae	2	9	205.0±37.0	marine
Centrogeniidae	1	1	25.0	marine
Notograptidae	1	3	10.3±0.4	marine
Dinopercidae	2	2	52.9±31.3	marine
Scytalinidae	1	1	15.0	marine
Pseudaphritidae	1	1	36.0	catadromous
Cheimarrhichthyidae	1	1	15.0	marine
Trichonotidae	1	6	16.2±3.3	marine
Creediidae	7	16	5.2±1.8	marine
Leptoscopidae	2	4	13.3±3.2	marine
Draconettidae	2	7	9.2±2.6	marine
Xenisthmidae	5	19	2.5±0.7	marine
Ptereleotridae	6	31	8.8±5.5	marine
Amarsipidae	1	1	12.2	marine

Biological data were taken from FishBase [4].

Previous analyses have hinted at the existence of a pelagic radiation of percomorphs. Yagishita *et al.* [19] found a strongly supported group comprising some members of Pelagia (Arripidae, Centrolophidae, Nomeidae, Scombridae), and noticed that the 4 families, together with other members of the novel clade (Chiasmodontidae [18], [20], [21] and Bramidae [18]), share a pelagic lifestyle associated with long-distance migrations [19]. Yagishita *et al*. [19] suggested that future addition of pelagic species of uncertain placement within Percomorpha would further expand the limits of this distinct, previously unrecognized pelagic clade. Our study confirms this prediction by adding 9 families that exclusively contain pelagic members to this novel clade. While this manuscript was under review, two papers on the euteleostean phylogenies based on extensive character sampling from 20 nuclear genes have been published [32], [33] and their resultant trees are congruent in terms of the limits of Pelagia with the exception of Arripidae (this group is unsampled in their studies [32], [33]). Intrarelationships of Pelagia, however, exhibit partial incongruence (e.g., non-monophyletic scombrids in [32], [33]), which clearly require further investigation.

Considering that current morphological classifications have placed representatives of these 15 families into six perciform suborders [12], the recognition of anatomical synapomorphies for Pelagia might prove difficult. Although members of this group are clearly united by a pelagic ecology, long-distance migrations are characteristic of only few of the 15 families. Most members of Pelagia are relatively large (≥30 cm) predatory marine fishes consuming various food items from mainly jellyfish and hydroids in five stromateoid families to bony fish, squid, and crustaceans in the remaining 10 families (Table 5). Exceptions to the general pattern of large body size is found in mesopelagic families occurring principally below 200 m, such as species of Chiasmodontidae (mean maximum length = 16.2 cm ±6.6 SD) and Caristiidae (20.5 cm ±10.2 SD). These taxa are often collectively called “micronekton” together with more abundant and diverse dragonfishes (Stomiiformes) and lanternfishes (Myctophidae) [34], and their smaller sizes might reflect ecological constraints in deep midwater environments where food availability is more limited than in the euphotic zone [34].

**Table 5:** Summary of taxonomic and biological information of Pelagia.

Suborder	Family	Genus	Species	Max length (cm)	Ecological division	Activity level	Main food
		73	232				
Percoidei	Pomatomidae	1	1	130	marine	normal	bony fish, squids
	Bramidae	7	22	51.6±22.2	marine	N/A	bony fish, crustaceans
	Caristiidae	2	5	20.5±10.2	marine	N/A	bony fish, crustaceans
	Arripidae	1	4	77.8±24.9	marine	active	bony fish, shrimp
Trachinoidei	Chiasmodontidae	4	15	16.2±6.6	marine	N/A	bony fish
Icosteoidei	Icosteidae	1	1	213	marine	N/A	bony fish, squids
Scombrolabracoidei	Scombrolabracidae	1	1	30	marine	N/A	bony fish, crustaceans, squids
Scombroidei	Gempylidae	16	24	83.0±74.2	marine	normal	bony fish, crustaceans, squids
	Trichiuridae	10	39	98.6±62.5	marine	normal	bony fish, crustaceans, squids
	Scombridae	15	51	126 6±82.5	marine	very active	bony fish, squids
Stromateoidei	Centrolophidae	7	28	63.4±35.5	marine	normal	jellyfish, hydroids
	Nomeidae	3	16	48.1±38.8	marine	N/A	salps
	Ariommatidae	1	7	34.8±20.6	marine	active	small benthic invertebrates
	Tetragonuridae	1	3	60.0±14.1	marine	N/A	jellyfish, hydroids
	Stromateidae	3	15	33.4±13.8	marine	normal	jellyfish, hydroids, invertebrates

Biological data were taken from FishBase [4].

In contrast to the obvious single origin of Pelagia, familial interrelationships within Pelagia are not robustly supported. Weak support for internal nodes is commonly reported in phylogenetic analyses of groups thought to have undergone adaptive radiations, such as those of cichlids in Lake Tanganyika [35], and is frequently attributed to the temporal bursts of species diversification associated with ecological divergence [36]. Evidence for both of these important attributes of adaptive radiation is apparent in the diversification of Pelagia.

The reconstructed timetree (Fig. 6) shows that internal branches connecting those poorly supported nodes are narrow, and are concentrated in a short interval during the early Paleogene between 48.9 and 51.5 Ma (representing only 2.6 Ma). Remarkable examples of adaptive morphological and ecological traits in members of Clade C (with the exception of those of scombrids) include: the greatly enlarged dorsal and anal fins of mesopelagic manefishes (Caristiidae; Fig. 1*c*) and fanfishes (Bramidae), which have been interpreted as an antipredation device [37]; the highly distensible stomachs of swallowers (Chiasmodontidae; Fig. 1*e*), which allow members of the group to consume prey considerably longer than their own bodies [38]; the unusual ecology of driftfishes (Nomeidae; Fig. 1*l*), which hover around and under floating logs, jellyfishes, and seaweed as juveniles, including particularly notable symbioses between juvenile *Nomeus gronouvii* and a pelagic cnidarian (Portuguese Man-of-War) [39]; and the preferred habitat of juvenile squaretails (Tetragonuridae; Fig. 1*n*), which live inside pelagic tunicates (salps and pyrosomes) and use their unusual dentition to graze on their hosts [40]. Thus it is evident that taxonomic radiation within Pelagia is accompanied by profound and rapid ecological divergence in the pelagic realm. The diversification of Pelagia is unusual in occurring in a vast and apparently homogenous environment, which stands in contrast to the small, isolated areas such as lakes or oceanic islands with heterogeneous habitats that represent the setting for most classic examples of adaptive radiation [24].

The Cretaceous-Paleogene extinction appears to have selected against large-bodied predatory fishes, which were represented in Late Cretaceous shallow marine environments by stem teleost clades (pachycormiforms, pachyrhizodontids, ichthyodectiforms) and a diversity of aulopiforms [41]–[43]. The apparent radiation of epipelagic clades of predatory acanthomorphs in the early Paleogene, including scombrids, has been interpreted by paleontologists as the evolutionary re-filling of the ecological roles vacated by these extinction victims [44]. Our new framework for understanding scombrid evolution supports an early Paleogene radiation of the group (but see [17] for older [mid-Cretaceous origin of crown Scombridae] and [45] younger [late Paleogene] crown ages based on different molecular data and calibration sets), but also suggests that the clade arose from a deep-water ancestor; this inference is particularly interesting given that both deep-water teleosts and elasmobranchs seem to have been little affected by the Cretaceous-Paleogene extinction [41], [46]. With epipelagic predators devastated by the end-Cretaceous event, Pelagia was free to radiate in the epipelagic realm during the early Paleogene, diversifying into 232 modern species distributed across 73 genera and 15 disparate families, including 51 species of Scombridae.

## Conclusions

Bioinformatic and mitogenomic analyses, which draw on an extensive taxon sample (5368 and 124 species, respectively), clearly demonstrate a single origin of Pelagia (Figs. 4, 5) despite the extraordinary morphological heterogeneity of the 15 families included within this radiation (Fig. 1). Monophyly of Pelagia is robustly supported (100% BPs in mitogenomic analysis across the four data sets), as is the monophyly of most polyspecific families within this radiation (Trichiuridae, a subset of Gempylidae, Bramidae, Tetragonuridae, Chiasmodontidae, Centrolophidae, Stromateidae, Nomeidae, Scombridae; all with BP≥95%). However, considerable uncertainty exists with respect to the relationships among these families with the exception of three well-supported suprafamilial clades (Tetragonuridae + Chiasmodontidae; Arripidae + Pomatomidae; Stromateidae + Ariommatidae, Nomeidae). Interpreted in the light of our molecular clock and paleontological investigations of Pelagia, we regard the ambiguity surrounding these early phylogenetic splits as a consequence of the rapid divergence in Late Cretaceous-early Paleogene interval (Fig. 6). These short internal nodes, combined with the divergent ecological and morphological traits shown by modern representatives (Figs. 1, 4, 6), suggest that Pelagia represents an adaptive radiation in the pelagic realm. The concentration of divergences among disparate lineages within Pelagia during the early Paleogene is consistent with an “ecological release” model, whereby the group radiated into emptied ecological roles following selective extinction of epipelagic predatory teleosts at the end of the Cretaceous.

### Phylogenetic Definition of Pelagia

We follow the PhyloCode Article 9 general requirements for establishment of clade name (http://www.ohio.edu/phylocode/art9.html).

Pelagia, M. Miya & M. Friedman, new clade name.

Definition (node-based): The least inclusive clade containing *Trichiurus lepturus* Linnaeus, 1758, *Gempylus serpens* Cuvier, 1829, *Ruvettus pretiosus* Cocco, 1833, *Platyberyx opalescens* Zugmayer, 1911, *Icosteus aenigmaticus* Lockington, 1880, *Taractes asper* Lower 1843, *Scombrolabrax heterolepis* Roule, 1921, *Icichthys lockingtoni* Jordan & Gilbert, 1880, *Tetragonurus cuvieri* Risso, 1810, *Chiasmodon niger* Johnson, 1864, *Pampus argenteus* (Euphrasen, 1788), *Ariomma indica* (Day, 1871), *Psenes cyanophrys* Valenciennes, 1833, *Pomatomus saltatrix* (Linnaeus, 1766), *Arripis trutta* (Forster, 1801), and *Scomber scombrus* Linnaeus, 1758.

Etymology: Greek. From the plural of the neutral adjective *pelágion* (πελάγιον), meaning “of the sea”.

Reference phylogeny (Fig. 4): Includes the species in the definition as well as all species in Pomatomidae, Bramidae, Caristiidae, Arripidae, Chiasmodontidae, Icosteidae, Scombrolabracidae, Gempylidae, Trichiuridae, Scombridae, Centrolophidae, Nomeidae, Ariommatidae, Tetragonuridae, Stromateidae, (and possibly Amarsipidae, unsampled in the present study).

## Materials and Methods

### Bioinformatic Analysis

To find possible close relatives of scombrids, we downloaded partial nucleotide sequences of mitochondrial (mt) and nuclear (nc) genes (≥300 bp) in a flat file format from all species of Percomorpha deposited in GenBank. Sequences were sorted into individual genes and only the longest sequences from identical species were retained using a Perl script (GenBankStrip.pl) written by O.R.P. Bininda-Emonds. For sufficient taxonomic coverage, those protein-coding genes with ≥100 species (six mt and three nc genes) were used for subsequent phylogenetic analysis with *Polymixia japonica* as an outgroup for rooting (total 10,731 ingroup sequences; for a list of species and accession numbers, see Table S2). Although numerous percomorph species have been deposited for the two mt ribosomal genes in the database (1,412 species for the 12S and 3,263 species for the 16S), they were not used in the analysis because of difficulties in unambiguous multiple alignment across all the taxa involved. All 9 data sets are deposited in DataDryad and available from http://datadryad.org/resource/doi:10.5061/dryad.5ns57.

Those nine protein-coding gene sequences were individually aligned using MAFFT v. 6 [47] and unambiguously aligned sequences were partitioned into codon positions. The resulting nine data sets were individually subjected to partitioned maximum likelihood (ML) analysis using RAxML v. 7.2.8 [48]. A general time reversible model with sites following a discrete gamma distribution (GTR + gamma [49]; the model recommended by the author of the program) was used and a rapid bootstrap (BS) analysis [50] was conducted with 1,000 replicates (–f a option).

### Mitogenomic Analysis

The above bioinformatic analyses identified that 12 non-scombroid families formed the least inclusive monophyletic group that contains all core members of the 3 classical scombroid families (Gempylidae, Trichiuridae, Scombridae; total 15 families) and constituent species of those 15 families were all pelagic. Based on these observations, we sampled 56 species from those 15 families (ingroup) and 68 outgroup species including representative pelagic percomorph taxa (total 124 species; for a list of species and accession numbers, see Table S3). We generated new whole mitogenome sequences for 37 ingroup and 18 outgroup species using a combination of long and short polymerase chain reactions (PCRs) and direct cycle sequencing techniques [51].

Whole mitogenome sequences from the 124 species were concatenated with the pre-aligned sequences used in Azuma *et al.* [52] in a FASTA format and they were together subjected to multiple alignment using MAFFT. We excluded the ND6 gene from the mt 13 protein-coding genes because of its heterogeneous base composition and poor phylogenetic performance [53].

Unambiguously aligned sequences were used to construct four data sets that treated 12 protein-coding genes differently. The first data set excluded quickly saturated transitional changes in the third codon positions by converting purine (A/G) and pyrimidine (C/T) nucleotides to A and C, respectively (12_n_3_r_RT_n_ where “R” and “T” denote ribosomal and transfer RNA genes, respectively, and the subscripts “n” and “r” denote nucleotides and a modified RY-coding, respectively, following Saitoh *et al*. [26]). The second data set (123_n_RT_n_) included all codon positions, while the third one (12_n_RT_n_) excluded third codon positions entirely. The fourth data set converted protein-coding genes into amino acids (designated as 123_a_RT_n_). All 4 data sets are deposited in DataDryad and available from http://datadryad.org/resource/doi:10.5061/dryad.5ns57.

These 4 data sets were divided into 3–5 partitions depending on the data set (three partitions in the 123_a_RT_n_ data set, four partitions in the 12_n_RT_n_ data set, and five partitions in the 12_n_3_r_RT_n_ and 123_n_RT_n_ data sets) and subjected to partitioned ML analysis using RAxML [48]. A general time reversible model with sites following a discrete gamma distribution (GTR+gamma) was used and a rapid bootstrap (BS) analysis was conducted with 1000 replications (–f a option). For amino acid sequences, the MTREV model [54] with sites following a discrete gamma distribution (*Γ*) was used.

Probabilities of alternative hypotheses were calculated using the likelihood-based approximately unbiased (AU) test [55], an alternative to the more commonly used SH test [56], as implemented in CONSEL v. 0.1 k [57]. We manually created constrained tree topologies with reference to alternative hypotheses and then performed RAxML analysis with each constraint. We conducted fast bootstrapping with 100 replicates as described above, and the resulting best-scoring ML tree was considered as the constrained ML tree. The constrained and unconstrained ML trees (best-scoring ML tree without constraint) were used to compute the per-site log likelihood scores for each tree using the –f g option in RAxML and the output was subjected to CONSEL [57] analysis to calculate statistical significance of the differences in likelihood scores.

### Divergence Time Estimation

A relaxed molecular-clock method implemented in an MCMCTREE program in PAML v. 4.5 [58] was used for dating analysis with the best-scoring ML tree from the 12_n_3_r_RT_n_ data set as the best estimate of the phylogeny following the argument by Saitoh *et al*. [26]. The ML estimates of branch lengths were obtained with the 12_n_RT_n_ data set under the GTR+gamma substitution model to avoid overestimation with quickly saturated third codon positions [59]. The independent-rates (IR) model was used, where the rates follow a log-normal distribution (i.e., the logarithm of the rate is normally distributed). MCMC approximation with a burn-in period of 10,000 cycles was obtained, with samples taken every 50 cycles in order to create a total of 10,000 samples. To diagnose possible failure of the Markov chains to converge to their stationary distribution, two replicate MCMC runs were performed with two different random seeds for each analysis. MCMC samples from the two runs were combined after checking the distributions of parameter values using Tracer 1.5 (available from http://tree.bio.ed.ac.uk/software/tracer/). The number of samples (20,000) was large enough to reach effective sample sizes (ESSs >200) for all parameters estimated in this study.

Five fossil-based time constraints outside the clade of 15 families were used following Near *et al.* [16] with slight updates (see Table 6). As these fossil-based time constraints were well documented and their phylogenetic placement could be justified, the minimum- and maximum-bound densities were assumed using the two shape parameters (*p* and *c*) of the truncated Cauchy distribution [58]. A separate dating analysis was performed with three additional biogeographic constraints based on concordance between the sequence of Gondwanan breakup and patterns of cichlid divergences [52]. Although scombroids and their close relatives are well represented in the fossil record, we did not apply any calibrations within our ingroup so that our results would represent a truly independent check on paleontological timescales for the radiation of this clade.

**Table 6:** Calibration points for the divergence time estimation using MCMCTREE.

MRCA of *Gadus morhua* and *Polymixia japonica*
*Homonotichthys dorsalis*. Lower Chalk of Sussex and Kent, UK [69].
*Source*.– Near *et al*. [16].
*Character*-*based justification*.– United with *Polymixia* on the basis of: four full-sized branchiostegals; anterior branchiostegals reduced and forming support for chin barbell [69], [70].
*Stratigraphy*.– Middle-upper Cenomanian (zone of *Holoaster subglobosus* [69], [71]).
*Absolute age estimate*.– 93.9 Ma.
*Calibration*.– a location parameter (*p*) = 0.1 and scale parameter (*c*) = 0.00180 to set 93.9 Ma as the 2.5% soft minimum and 125.0 Ma as the 97.5% soft maximum bounds. The upper bound is based on the Cenomanian aged stem-lineage acanthomorph ctenothrissiform taxa *Aulolepis*, *Ctenothrissa* and *Heterothrissa* [69], [72].
**MRCA of Carangidae and Echeneoidea**
*Archaeus oblongus*. Danatinsk Suite, Uylya-Kushlyuk locality, Turkmenistan [73].
*Source*.– Near *et al*. [16].
*Character*-*based justification*.– United with Carangidae on the basis of: broad gap between second and third anal-fin spines [74].
*Character*-*based justification*.– United with *Polymixia* on the basis of: four full-sized branchiostegals; anterior branchiostegals reduced and forming support for chin barbell [69], [70].
*Stratigraphy*.– uppermost Thanetian-lowermost Ypresian [75].
*Absolute age estimate*.–56.0 Ma [76].
*Calibration*.–a location parameter (*p*) = 0.1 and scale parameter (*c*) = 0.04175 to set 56.0 Ma as the 2.5% soft minimum and 93.9 Ma as the 97.5% soft maximum bounds. The upper bound is based on the calculation of FA_95_ following Marshall [77].
**MRCA of *Echeneis naucrates* and [Rachycentridae + Coryphaenidae]**
*Opisthomyzon glaronensis* and unnamed echeneid cf. *Echeneis*. Engi Slates, Matt, Glarus province, Switzerland (†*Opisthomyzon*; previously misreported as *Uropteryx* [78]); ‘fish shales’, Frauenweiler clay pit, Germany (cf. *Echeneis* [79]).
*Source*.–Near *et al*. [16].
*Cladistic*-*based justification*.–United with Echeneidae on the basis of: no supraneurals; multiple anal-fin pterygiophores insert anterior to first haemal spine; spinous dorsal fin modified as adhesion disc [80], [81].
*Stratigraphy*.–Engi slates: Rupelian, but younger than ca. 31.7 Ma (radiometric dates for underlying Taveyannaz Formation; K/Ar: 31.7±1.6 and 32.4±1.6 Ma; ^40^Ar/^39^Ar: 31.96±0.9 Ma [82], [83]. Frauenweiler: Rupelian (30.1 Ma [84]).
*Absolute age estimate*.–30.1 Ma [84].
*Calibration*.–a location parameter (*p*) = 0.1 and scale parameter (*c*) = 0.05332 to set 30.1 Ma as the 2.5% soft minimum and 56.1 Ma as the 97.5% soft maximum bounds. The upper bound is based on the calculation of FA_95_ following Marshall [77].
**MRCA of *Dactylopterus tiltoni* and Syngnathiformes**
*Gasterorhamphosus zuppichinii*. ‘Calcari di Melissano,’ Porto Selvaggio, Lecce province, Italy [85]. Source.– Near et al. [16].
*Character*-*based justification*.–United with Syngnathiformes by: anal-fin spine absent; enlarged dorsal-fin spine with serrated posterior margin; elongated tubular snout; pleural ribs absent; cleithrum bears enlarged posterodorsal process; rod-like anteroventral process of coracoid; pectoral rays simple [86], [87].
*Stratigraphy*.–Uppermost Campanian-lowermost Maastrichtian [88].
*Absolute age estimate*.–72.1 Ma [76].
*Calibration*.–a location parameter (*p*) = 0.1 and scale parameter (*c*) = 0.01595 to set 72.1 Ma as the 2.5% soft minimum and 93.9 Ma as the 97.5% soft maximum bounds. The upper bound is based on the calculation of FA_95_ following Marshall [77].
**MRCA of *Aeoliscus strigatus* and *Macroramphosus scolopax***
*Paramphisile weileri* and *Paraeoliscus robinetae*. Pesciara beds of ‘Calcari nummulitici’, Bolca, Italy [89].
*Source*.–Near *et al*. [16].
*Character*-*based justification*.–United with Centriscidae by: caudal fin directed posteroventrally (*Paraeoliscus*); dorsal spine jointed distally [87].
Stratigraphy.–upper Ypresian [NP14] [90].
*Absolute age estimate*.–50.0 Ma [90].
*Calibration*.–a location parameter (*p*) = 0.1 and scale parameter (*c*) = 0.02602 to set 50.0 Ma as the 2.5% soft minimum and 72.1 Ma as the 97.5% soft maximum bounds. The upper bound is based on the calculation of FA_95_ following Marshall [77].

### Database of Fossil Occurrences and Estimated Confidence Intervals

We assembled a database of fossil representatives of the 15 families of Pelagia through a literature review combined with the Paleobiology Database (PBDB; www.paleodb.org). Specifically, we attempted to estimate the approximate number of horizons yielding body-fossil remains of these pelagian families in order to establish probabilistic confidence limits for first appearances that can be compared to molecular-clock divergence time estimates within Pelagia. In addition to the age of localities and the genera of Pelagia that they are reported to yield, we have also recorded whether the material found at these sites is articulated or consists of fragmentary remains.

Unfortunately, no family assigned to Pelagia has been the focus of a published cladistic analysis that includes both fossil and living taxa. Hypothesized placements of fossil members of Pelagia exist, but either take the form of hand-drawn trees, verbal arguments, or cladograms in unpublished theses [60]. In addition, the identification of some fossils, especially those represented by highly fragmentary remains (e.g., teeth), is often based on overall similarity rather than specific apomorphies. A revision of the rich fossil record of Pelagia is well beyond the scope of this analysis. Rather, it is our aim to determine the degree to which the fossil record of the group, as it is currently understood, agrees with the independent evolutionary timescale we have estimated using molecular datasets combined with fossil calibrations not belonging to Pelagia.

For many examples, we are uncertain whether relevant fossils belong to crown families or lie on the familial stem, outside the crown. In light of the limited information concerning the precise relationships of fossil taxa to modern representatives of Pelagia, we argue that it is most appropriate to organize extinct taxa relative to the total-group families to which they most likely belong. Therefore the paleontological estimates we estimate here pertain to total-group–rather than crown-group–ages. Following Monsch [60], we consider the placement of some Paleogene genera (*Tamesichthys*, *Eocoelopoma*, *Scombramphodon*, *Spyraenodus*, *Wetherellus*, *Woodwardella*) traditionally associated with Scombridae as incertae sedis with respect to core members of a classical Scombroidei. Our molecular result resolves Gempylidae as a grade, but most fossils identified as gempylids are not sufficiently well described for us to determine to which of the two lineages they should be assigned. We therefore place all gempylid fossils in a single category, and acknowledge that our fossil age estimate represents that for a potentially non-monophyletic group. Finally, we have not made a count of fossils assigned to the extinct Euzaphlegidae, an ill-defined and likely para- or polyphyletic assemblage of taxa that share many features with members of the classical Scombroidei [61], [62]. Euzaphlegids are relatively common in Paleogene fish assemblages, particularly those thought to be deposited in more open marine settings. A better understanding the relationships of Pelagia might help to better interpret the likely affinities of individual taxa assigned to this problematic extinct group.

We provide an account of the first appearances of each of the 15 families of Pelagia in Text S1. Where first appearances are based on highly fragmentary remains (e.g., isolated teeth), we have also provided first appearances based on articulated or associated body fossils that might be considered more reliable markers. First appearances in the fossil record will always underestimate true times of origin, so comparisons between first appearances as gauged from a literal reading of paleontological data with molecular clock estimates for the same divergence events are of questionable value. Therefore, we have estimated confidence limits on the time of origin for each of the families of Pelagia for which this is possible. We have calculated two-tailed 95% confidence intervals for the first appearance of these groups using the expressions provided by Strauss and Sadler [27] and Marshall [28]:
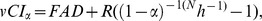
where *CI_α_* equals the age of the confidence limit for the origin of the group, *FAD* is the age of the oldest fossil assigned to the group, *R* is the stratigraphic range of the group, *α* is the level of confidence, and *N_h_* is the number of horizons yielding remains of the group. Two-tailed 95% confidence intervals for the origin of the group correspond to *CI_0.975_* (upper [oldest] CI) and *CI_0.05_* (lower [youngest] CI). We have used the unbiased maximum-likelihood point estimator for the time of first origin based on fossils in our comparisons with molecular timescales for evolution within Pelagia. This is given by Strauss & Sadler [27] as:



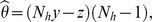
where 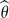 is the unbiased maximum-likelihood point estimate for the time of evolutionary origin, *N_h_* is as in the previous expression, *y* is the age of the oldest observed occurrence, and *z* is the age of the youngest observed occurrence.

The approach outlined above assumes uniform preservation potential over the history of a group, an assumption that is very unlikely to be met [63], [64]. Preservation potential can vary as a function of many factors, the most obvious being changes in the taxonomic richness or numerical abundance of a clade over its evolutionary history, and variation in the nature of the stratigraphic record that might favor preservation in some intervals more so than others. In order to test how sensitive our estimated paleontological timescales are to variation in preservation potential over time, we have also calculated *CI_0.5_* for families where this is possible under the assumption that preservation potential for each was an order of magnitude higher during its observed fossil range than in earlier parts of its evolutionary history (this is indicated as *CI_0.5, 10%_*). These and other *CI* are given in the spreadsheets associated with this supplement (Tables S4, S5).

### Character Mapping

Depth ranges of ingroup species were accessed from FishBase using the package *rfishbase* [65] for R [66]. Mean depths were calculated for those species where minimum and maximum depths were given. We then mapped depth ecology onto our time-scaled phylogenies, visualizing patterns of evolution in depth preferences using the *phytools* package [67]. This method computes maximum-likelihood estimates of ancestral states by sequentially re-rooting the tree at each internal node and calculating the contrast state at the root using Felsenstein’s [68] contrast algorithm. States along each branch are then interpolated using equation 2 of Felsenstein [68].

## Supporting Information

Figure S1 **Node numbers for reference to the estimated node ages and 95% credible intervals shown in Table S2.**
(EPS)

Table S1 **Summary of node ages in MCMCTREE analysis with two different node constraints (fossil constraints only vs. fossil constraints plus cichlid biogeography).** For node number, see Fig. S1.
(DOCX)

Table S2 **List of species used in the bioinformatic analysis.**
(DOCX)

Table S3 **List of species used in the mitogenomic analysis.**
(DOCX)

Table S4 **Fossil horizons yielding the 15 families of Pelagia.**
(DOCX)

Table S5 **Paleontological timescale for the 15 families of Pelagia.**
(DOCX)

Text S1 **First appearances of the 15 families of Pelagia as body fossils.**
(DOCX)

Text S2 **Tree topologies in a newick format shown in** **Figures 2** **–** **4** **.**
(DOCX)
