# Supplementary material for: Evolutionary Origin of the Scombridae (Tunas and Mackerels): Members of a Paleogene Adaptive Radiation with 14 Other Pelagic Fish Families
Source: PLoS One. 2013 Sep 4;8(9):e73535. doi: 10.1371/journal.pone.0073535 (PMC3762723; doi:10.1371/journal.pone.0073535)
Supplement: Table S1 — Summary of node ages in MCMCTREE analysis with two different node constraints (fossil constraints only vs. fossil constraints plus cichlid biogeography). For node number, see Fig. S1. (DOCX) [file pone.0073535.s002.docx]

**Table S1** Summary of node ages in MCMCTREE analysis. For node number, see Fig. S1

.

| Node | Mean | Upper | Lower | Remarks |  | Node | Mean | Upper | Lower | Remarks |
| --- | --- | --- | --- | --- | --- | --- | --- | --- | --- | --- |
| 125 | 128.0 | 111.7 | 155.8 |  |  | 125 | 166.1 | 149.9 | 186.9 |  |
| 126 | 118.1 | 108.2 | 129.9 |  |  | 126 | 163.2 | 147.7 | 182.2 |  |
| 127 | 101.3 | 94.7 | 106.3 |  |  | 127 | 120.2 | 103.9 | 126.6 |  |
| 128 | 110.9 | 101.6 | 121.5 |  |  | 128 | 157.6 | 142.6 | 176.0 |  |
| 129 | 99.5 | 92.2 | 105.8 | Percomorpha |  | 129 | 145.9 | 132.3 | 161.2 | Percomorpha |
| 130 | 98.7 | 91.3 | 105.0 |  |  | 130 | 144.7 | 131.3 | 159.7 |  |
| 131 | 97.6 | 90.0 | 103.9 |  |  | 131 | 143.3 | 130.2 | 158.1 |  |
| 132 | 95.6 | 87.9 | 102.1 |  |  | 132 | 140.8 | 128.2 | 155.4 |  |
| 133 | 92.8 | 84.7 | 99.7 |  |  | 133 | 136.2 | 122.5 | 150.9 |  |
| 134 | 91.8 | 83.7 | 98.8 |  |  | 134 | 134.8 | 121.2 | 149.5 |  |
| 135 | 80.0 | 70.9 | 89.0 |  |  | 135 | 116.5 | 102.1 | 133.1 |  |
| 136 | 51.8 | 41.0 | 63.1 |  |  | 136 | 75.3 | 59.3 | 91.9 |  |
| 137 | 82.8 | 73.6 | 91.4 |  |  | 137 | 120.3 | 106.4 | 136.1 |  |
| 138 | 78.4 | 68.7 | 87.3 |  |  | 138 | 113.5 | 98.2 | 128.8 |  |
| 139 | 47.9 | 32.4 | 62.4 |  |  | 139 | 71.0 | 47.8 | 91.5 |  |
| 140 | 35.0 | 22.9 | 49.1 |  |  | 140 | 52.1 | 32.8 | 75.2 |  |
| 141 | 83.8 | 73.2 | 93.1 |  |  | 141 | 122.6 | 106.6 | 140.3 |  |
| 142 | 63.8 | 47.4 | 80.5 |  |  | 142 | 92.1 | 67.9 | 117.6 |  |
| 143 | 82.9 | 73.6 | 91.5 |  |  | 143 | 130.0 | 120.1 | 141.8 |  |
| 144 | 79.2 | 69.9 | 88.2 |  |  | 144 | 127.1 | 118.0 | 138.1 |  |
| 145 | 57.5 | 49.0 | 68.2 |  |  | 145 | 116.1 | 110.4 | 122.5 |  |
| 146 | 51.5 | 43.1 | 61.0 |  |  | 146 | 111.8 | 107.2 | 116.3 |  |
| 147 | 46.8 | 38.4 | 56.2 |  |  | 147 | 104.0 | 99.6 | 110.1 |  |
| 148 | 29.9 | 20.4 | 40.3 |  |  | 148 | 66.0 | 42.2 | 84.6 |  |
| 149 | 30.7 | 22.6 | 39.8 |  |  | 149 | 57.6 | 37.2 | 80.1 |  |
| 150 | 22.0 | 15.2 | 30.3 |  |  | 150 | 39.4 | 24.6 | 59.6 |  |
| 151 | 12.4 | 7.6 | 18.9 |  |  | 151 | 20.4 | 11.1 | 33.9 |  |
| 152 | 45.6 | 35.7 | 56.0 |  |  | 152 | 99.9 | 78.2 | 110.6 |  |
| 153 | 44.1 | 32.8 | 56.0 |  |  | 153 | 90.0 | 85.0 | 95.0 |  |
| 154 | 40.0 | 23.4 | 57.6 |  |  | 154 | 64.9 | 35.8 | 92.5 |  |
| 155 | 75.5 | 64.0 | 84.8 |  |  | 155 | 116.7 | 103.5 | 130.6 |  |
| 156 | 43.6 | 31.0 | 56.3 |  |  | 156 | 68.1 | 47.0 | 87.6 |  |
| 157 | 86.2 | 75.5 | 94.8 |  |  | 157 | 111.9 | 84.3 | 130.5 |  |
| 158 | 83.6 | 72.7 | 92.7 |  |  | 158 | 106.6 | 80.0 | 125.3 |  |
| 159 | 40.9 | 25.7 | 56.4 |  |  | 159 | 56.3 | 34.2 | 79.9 |  |
| 160 | 76.8 | 63.2 | 87.3 |  |  | 160 | 78.4 | 65.3 | 93.4 |  |
| 161 | 21.9 | 14.3 | 31.8 |  |  | 161 | 30.9 | 19.7 | 45.4 |  |
| 162 | 16.7 | 10.1 | 25.8 |  |  | 162 | 23.8 | 13.8 | 37.2 |  |
| 163 | 69.8 | 55.1 | 82.0 |  |  | 163 | 56.4 | 53.0 | 60.4 |  |
| 164 | 39.4 | 29.1 | 51.0 |  |  | 164 | 34.2 | 27.4 | 41.0 |  |
| 165 | 7.3 | 4.1 | 11.7 |  |  | 165 | 8.7 | 5.0 | 13.5 |  |
| 166 | 83.5 | 72.6 | 92.2 |  |  | 166 | 108.4 | 80.3 | 126.4 |  |
| 167 | 80.8 | 69.6 | 89.8 |  |  | 167 | 104.5 | 75.7 | 122.6 |  |
| 168 | 32.8 | 18.9 | 55.6 |  |  | 168 | 52.8 | 28.5 | 84.2 |  |
| 169 | 10.7 | 6.3 | 17.3 |  |  | 169 | 16.4 | 9.1 | 27.8 |  |
| 170 | 2.8 | 1.7 | 4.1 |  |  | 170 | 4.0 | 2.4 | 6.1 |  |
| 171 | 1.8 | 1.1 | 2.7 |  |  | 171 | 2.6 | 1.5 | 4.0 |  |
| 172 | 1.5 | 0.9 | 2.3 |  |  | 172 | 2.2 | 1.2 | 3.5 |  |
| 173 | 1.2 | 0.6 | 1.9 |  |  | 173 | 1.7 | 0.8 | 2.8 |  |
| 174 | 76.5 | 63.7 | 85.9 |  |  | 174 | 98.3 | 69.5 | 117.6 |  |
| 175 | 77.6 | 66.2 | 86.7 |  |  | 175 | 100.9 | 72.9 | 119.2 |  |
| 176 | 51.1 | 39.0 | 62.8 |  |  | 176 | 67.2 | 44.9 | 86.6 |  |
| 177 | 95.6 | 87.2 | 102.4 |  |  | 177 | 139.2 | 124.1 | 154.8 |  |
| 178 | 90.0 | 80.0 | 98.1 |  |  | 178 | 129.6 | 114.7 | 146.8 |  |
| 179 | 84.7 | 74.6 | 93.7 |  |  | 179 | 121.2 | 106.5 | 138.8 |  |
| 180 | 81.3 | 71.1 | 90.8 |  |  | 180 | 116.0 | 100.5 | 134.0 |  |
| 181 | 67.6 | 50.2 | 81.6 |  |  | 181 | 93.8 | 68.9 | 117.8 |  |
| 182 | 94.1 | 85.4 | 101.1 |  |  | 182 | 136.9 | 121.9 | 152.5 |  |
| 183 | 86.5 | 76.6 | 95.0 |  |  | 183 | 125.0 | 109.4 | 141.8 |  |
| 184 | 78.3 | 67.3 | 87.8 |  |  | 184 | 113.2 | 94.3 | 130.5 |  |
| 185 | 41.0 | 27.6 | 54.8 |  |  | 185 | 61.2 | 39.1 | 82.6 |  |
| 186 | 89.4 | 78.8 | 97.7 |  |  | 186 | 129.5 | 112.7 | 146.6 |  |
| 187 | 54.6 | 39.7 | 72.4 |  |  | 187 | 79.0 | 57.6 | 105.6 |  |
| 188 | 94.8 | 87.1 | 101.8 |  |  | 188 | 138.1 | 120.5 | 154.6 |  |
| 189 | 79.4 | 75.0 | 84.3 |  |  | 189 | 85.1 | 74.1 | 94.1 |  |
| 190 | 57.1 | 52.7 | 68.2 |  |  | 190 | 67.8 | 57.9 | 72.8 |  |
| 191 | 69.1 | 59.4 | 80.1 | Pelagia |  | 191 | 103.0 | 84.7 | 119.9 | Pelagia |
| 192 | 50.0 | 40.8 | 59.3 |  |  | 192 | 75.1 | 59.2 | 91.2 |  |
| 193 | 35.9 | 27.1 | 45.6 |  |  | 193 | 54.2 | 39.1 | 71.6 |  |
| 194 | 26.4 | 17.2 | 36.6 |  |  | 194 | 40.0 | 24.5 | 57.6 |  |
| 195 | 0.3 | 0.0 | 0.8 |  |  | 195 | 0.5 | 0.0 | 1.2 |  |
| 196 | 39.5 | 29.2 | 49.7 |  |  | 196 | 59.7 | 41.8 | 77.0 |  |
| 197 | 60.7 | 53.4 | 70.4 | Clade A |  | 197 | 90.4 | 74.5 | 107.9 | Clade A |
| 198 | 55.6 | 49.3 | 64.6 | Clade B |  | 198 | 83.1 | 69.1 | 98.2 | Clade B |
| 199 | 52.6 | 46.4 | 61.2 | Clade C |  | 199 | 79.1 | 66.3 | 93.2 | Clade C |
| 200 | 50.6 | 43.8 | 59.4 |  |  | 200 | 76.4 | 63.2 | 91.0 |  |
| 201 | 29.8 | 22.0 | 38.5 |  |  | 201 | 44.8 | 32.2 | 58.2 |  |
| 202 | 25.0 | 17.7 | 33.3 |  |  | 202 | 37.6 | 25.8 | 50.7 |  |
| 203 | 17.2 | 10.9 | 25.3 |  |  | 203 | 25.8 | 15.4 | 38.7 |  |
| 204 | 47.8 | 40.3 | 57.0 |  |  | 204 | 72.5 | 58.6 | 87.6 |  |
| 205 | 51.5 | 45.4 | 60.0 |  |  | 205 | 77.8 | 65.1 | 92.0 |  |
| 206 | 49.9 | 43.7 | 58.4 |  |  | 206 | 75.6 | 63.0 | 89.8 |  |
| 207 | 44.7 | 37.5 | 53.4 |  |  | 207 | 68.1 | 54.8 | 83.0 |  |
| 208 | 10.4 | 5.5 | 17.9 |  |  | 208 | 15.8 | 7.9 | 28.1 |  |
| 209 | 38.9 | 31.4 | 47.5 |  |  | 209 | 59.2 | 45.5 | 75.0 |  |
| 210 | 33.1 | 25.0 | 41.6 |  |  | 210 | 50.5 | 36.7 | 66.9 |  |
| 211 | 46.9 | 40.4 | 55.5 |  |  | 211 | 71.3 | 58.8 | 85.4 |  |
| 212 | 28.7 | 21.4 | 37.5 |  |  | 212 | 43.5 | 31.2 | 56.9 |  |
| 213 | 9.4 | 5.2 | 15.4 |  |  | 213 | 14.1 | 7.6 | 23.6 |  |
| 214 | 50.4 | 44.3 | 59.0 |  |  | 214 | 76.4 | 63.8 | 90.6 |  |
| 215 | 44.8 | 37.8 | 53.3 |  |  | 215 | 68.5 | 55.5 | 82.9 |  |
| 216 | 38.6 | 31.5 | 46.7 |  |  | 216 | 59.2 | 46.1 | 74.1 |  |
| 217 | 20.0 | 14.1 | 26.9 |  |  | 217 | 30.3 | 20.2 | 42.4 |  |
| 218 | 39.0 | 29.5 | 48.3 |  |  | 218 | 59.8 | 42.9 | 76.3 |  |
| 219 | 10.6 | 6.0 | 17.1 |  |  | 219 | 15.9 | 8.5 | 26.4 |  |
| 220 | 11.2 | 5.6 | 20.4 |  |  | 220 | 16.9 | 8.1 | 31.8 |  |
| 221 | 48.9 | 42.3 | 57.6 |  |  | 221 | 74.3 | 61.6 | 88.8 |  |
| 222 | 37.7 | 25.9 | 49.9 | Scombridae |  | 222 | 60.2 | 39.1 | 79.3 | Scombridae |
| 223 | 36.8 | 25.2 | 48.9 |  |  | 223 | 58.8 | 37.9 | 77.9 |  |
| 224 | 18.0 | 12.1 | 25.6 |  |  | 224 | 28.1 | 18.4 | 41.0 |  |
| 225 | 7.8 | 4.8 | 11.9 |  |  | 225 | 12.1 | 7.2 | 18.9 |  |
| 226 | 1.4 | 0.7 | 2.4 |  |  | 226 | 2.1 | 1.0 | 3.7 |  |
| 227 | 1.1 | 0.5 | 2.1 |  |  | 227 | 1.7 | 0.7 | 3.2 |  |
| 228 | 22.5 | 12.6 | 40.4 |  |  | 228 | 36.8 | 18.1 | 69.6 |  |
| 229 | 14.3 | 9.1 | 22.8 |  |  | 229 | 20.8 | 12.8 | 33.3 |  |
| 230 | 10.6 | 6.6 | 16.8 |  |  | 230 | 15.6 | 9.4 | 25.4 |  |
| 231 | 4.7 | 3.0 | 7.0 |  |  | 231 | 6.9 | 4.3 | 10.6 |  |
| 232 | 1.8 | 0.9 | 3.1 |  |  | 232 | 2.7 | 1.3 | 4.7 |  |
| 233 | 4.0 | 2.3 | 6.3 |  |  | 233 | 5.9 | 3.3 | 9.4 |  |
| 234 | 1.2 | 0.6 | 2.1 |  |  | 234 | 1.8 | 0.9 | 3.2 |  |
| 235 | 0.9 | 0.5 | 1.6 |  |  | 235 | 1.4 | 0.6 | 2.4 |  |
| 236 | 0.5 | 0.1 | 1.0 |  |  | 236 | 0.8 | 0.2 | 1.6 |  |
| 237 | 0.1 | 0.0 | 0.4 |  |  | 237 | 0.2 | 0.0 | 0.6 |  |
| 238 | 0.3 | 0.0 | 0.6 |  |  | 238 | 0.3 | 0.0 | 0.9 |  |
| 239 | 41.9 | 33.5 | 51.2 |  |  | 239 | 64.3 | 49.9 | 79.4 |  |
| 240 | 54.1 | 47.4 | 63.2 |  |  | 240 | 80.5 | 66.4 | 95.7 |  |
| 241 | 46.6 | 37.2 | 56.7 |  |  | 241 | 70.2 | 54.7 | 86.3 |  |
| 242 | 55.6 | 48.2 | 65.0 |  |  | 242 | 82.4 | 67.1 | 99.5 |  |
| 243 | 53.9 | 46.3 | 63.3 |  |  | 243 | 79.8 | 64.8 | 96.3 |  |
| 244 | 46.3 | 36.0 | 56.8 |  |  | 244 | 68.9 | 51.5 | 86.3 |  |
| 245 | 49.6 | 41.6 | 58.9 |  |  | 245 | 73.5 | 58.5 | 89.7 |  |
| 246 | 38.6 | 29.7 | 48.2 |  |  | 246 | 57.3 | 41.6 | 74.5 |  |
| 247 | 99.5 | 85.3 | 114.2 |  |  | 247 | 144.0 | 117.3 | 166.4 |  |
