# Supplementary material for: Evolutionary Origin of the Scombridae (Tunas and Mackerels): Members of a Paleogene Adaptive Radiation with 14 Other Pelagic Fish Families
Source: PLoS One. 2013 Sep 4;8(9):e73535. doi: 10.1371/journal.pone.0073535 (PMC3762723; doi:10.1371/journal.pone.0073535)
Supplement: Table S2 — List of species used in the bioinformatic analysis. (DOCX) [file pone.0073535.s003.docx]

**Table S2** List of the species used in the bioinformatic analysis

| Order | Suborder | Family | Species | Accession Number | Gene |
| --- | --- | --- | --- | --- | --- |
| Total = 10 | Total = 33 | Total = 215 | Total = 5,367 | Total = 10,731 |  |
| Mugiliformes |  | Mugilidae | *Agonostomus monticola* | FN545594 | *CO1* |
| Mugiliformes |  | Mugilidae | *Aldrichetta forsteri* | EF609279 | *CO1* |
| Mugiliformes |  | Mugilidae | *Chelon labrosus* | EU392233 | *CO1* |
| Mugiliformes |  | Mugilidae | *Chelon labrosus* | EF427544 | *CYB* |
| Mugiliformes |  | Mugilidae | *Chelon labrosus* | DQ197837 | *rhod* |
| Mugiliformes |  | Mugilidae | *Crenimugil crenilabis* | FN600160 | *CO1* |
| Mugiliformes |  | Mugilidae | *Liza affinis* | EU083808 | *CYB* |
| Mugiliformes |  | Mugilidae | *Liza aurata* | EU392234 | *CO1* |
| Mugiliformes |  | Mugilidae | *Liza aurata* | EU224057 | *CYB* |
| Mugiliformes |  | Mugilidae | *Liza aurata* | EF439127 | *rhod* |
| Mugiliformes |  | Mugilidae | *Liza carinata* | FN600159 | *CO1* |
| Mugiliformes |  | Mugilidae | *Liza haematocheila* | EU392236 | *CO1* |
| Mugiliformes |  | Mugilidae | *Liza macrolepis* | EF609544 | *CO1* |
| Mugiliformes |  | Mugilidae | *Liza macrolepis* | EU078397 | *CYB* |
| Mugiliformes |  | Mugilidae | *Liza melinoptera* | HQ654719 | *CO1* |
| Mugiliformes |  | Mugilidae | *Liza parsia* | FJ384677 | *CO1* |
| Mugiliformes |  | Mugilidae | *Liza ramado* | EU392240 | *CO1* |
| Mugiliformes |  | Mugilidae | *Liza ramado* | EU224058 | *CYB* |
| Mugiliformes |  | Mugilidae | *Liza ramado* | EU224157 | *rhod* |
| Mugiliformes |  | Mugilidae | *Liza saliens* | EU392241 | *CO1* |
| Mugiliformes |  | Mugilidae | *Liza saliens* | EU715506 | *CYB* |
| Mugiliformes |  | Mugilidae | *Liza subviridis* | EU392242 | *CO1* |
| Mugiliformes |  | Mugilidae | *Liza tade* | EU014264 | *CO1* |
| Mugiliformes |  | Mugilidae | *Mugil cephalus* | HM007011 | *ATP6* |
| Mugiliformes |  | Mugilidae | *Mugil cephalus* | HQ149715 | *CO1* |
| Mugiliformes |  | Mugilidae | *Mugil cephalus* | EU083858 | *CYB* |
| Mugiliformes |  | Mugilidae | *Mugil cephalus* | EF095639 | *rag1* |
| Mugiliformes |  | Mugilidae | *Mugil cephalus* | EF095609 | *rhod* |
| Mugiliformes |  | Mugilidae | *Mugil curema* | GU225398 | *CO1* |
| Mugiliformes |  | Mugilidae | *Mugil curema* | DQ225775 | *CYB* |
| Mugiliformes |  | Mugilidae | *Mugil curema* | AY308783 | *rag1* |
| Mugiliformes |  | Mugilidae | *Mugil hospes* | EF426357 | *CYB* |
| Mugiliformes |  | Mugilidae | *Mugil incilis* | EF426382 | *CYB* |
| Mugiliformes |  | Mugilidae | *Mugil liza* | EU715462 | *CO1* |
| Mugiliformes |  | Mugilidae | *Mugil liza* | FJ228699 | *CYB* |
| Mugiliformes |  | Mugilidae | *Mugil platanus* | EU074483 | *CO1* |
| Mugiliformes |  | Mugilidae | *Mugil platanus* | EU715484 | *CYB* |
| Mugiliformes |  | Mugilidae | *Mugil trichodon* | FJ918950 | *CO1* |
| Mugiliformes |  | Mugilidae | *Mugil trichodon* | FJ896466 | *rag1* |
| Mugiliformes |  | Mugilidae | *Valamugil buchanani* | HQ149965 | *CO1* |
| Mugiliformes |  | Mugilidae | *Valamugil cunnesius* | EU595343 | *CO1* |
| Mugiliformes |  | Mugilidae | *Valamugil engeli* | EF607615 | *CO1* |
| Mugiliformes |  | Mugilidae | *Valamugil seheli* | EF609494 | *CO1* |
| Atheriniformes |  | Atherinopsidae | *Atherinella crystallina* | EF602045 | *ND2* |
| Atheriniformes |  | Atherinopsidae | *Atherinella milleri* | EF602046 | *ND2* |
| Atheriniformes |  | Atherinopsidae | *Atherinella panamensis* | AY655525 | *CO1* |
| Atheriniformes |  | Atherinopsidae | *Atherinella schultzi* | EF602044 | *ND2* |
| Atheriniformes |  | Atherinopsidae | *Atherinops affinis* | FJ264404 | *CYB* |
| Atheriniformes |  | Atherinopsidae | *Atherinosoma elongata* | GU932759 | *CYB* |
| Atheriniformes |  | Atherinopsidae | *Atherinosoma microstoma* | AY655523 | *CO1* |
| Atheriniformes |  | Atherinopsidae | *Atherinosoma microstoma* | GU932760 | *CYB* |
| Atheriniformes |  | Atherinopsidae | *Basilichthys microlepidotus* | FJ380116 | *CO1* |
| Atheriniformes |  | Atherinopsidae | *Basilichthys semotilus* | FJ380108 | *CO1* |
| Atheriniformes |  | Atherinopsidae | *Chirostoma arge* | EF602098 | *ND2* |
| Atheriniformes |  | Atherinopsidae | *Chirostoma attenuatum* | EF602083 | *ND2* |
| Atheriniformes |  | Atherinopsidae | *Chirostoma chapalae* | EF602076 | *ND2* |
| Atheriniformes |  | Atherinopsidae | *Chirostoma consocium* | EF602079 | *ND2* |
| Atheriniformes |  | Atherinopsidae | *Chirostoma contrerasi* | EF602099 | *ND2* |
| Atheriniformes |  | Atherinopsidae | *Chirostoma grandocule* | EF602062 | *ND2* |
| Atheriniformes |  | Atherinopsidae | *Chirostoma humboldtianum* | EF602074 | *ND2* |
| Atheriniformes |  | Atherinopsidae | *Chirostoma jordani* | EU751737 | *CO1* |
| Atheriniformes |  | Atherinopsidae | *Chirostoma jordani* | EF602090 | *ND2* |
| Atheriniformes |  | Atherinopsidae | *Chirostoma labarcae* | EU751746 | *CO1* |
| Atheriniformes |  | Atherinopsidae | *Chirostoma labarcae* | EF602084 | *ND2* |
| Atheriniformes |  | Atherinopsidae | *Chirostoma lucius* | EF602059 | *ND2* |
| Atheriniformes |  | Atherinopsidae | *Chirostoma patzcuaro* | EF602064 | *ND2* |
| Atheriniformes |  | Atherinopsidae | *Chirostoma promelas* | EF602060 | *ND2* |
| Atheriniformes |  | Atherinopsidae | *Chirostoma riojai* | EU751750 | *CO1* |
| Atheriniformes |  | Atherinopsidae | *Chirostoma riojai* | EF602096 | *ND2* |
| Atheriniformes |  | Atherinopsidae | *Chirostoma sphyraena* | EF602066 | *ND2* |
| Atheriniformes |  | Atherinopsidae | *Labidesthes sicculus* | EU524691 | *CO1* |
| Atheriniformes |  | Atherinopsidae | *Labidesthes sicculus* | HQ691297 | *CYB* |
| Atheriniformes |  | Atherinopsidae | *Labidesthes sicculus* | EF602056 | *ND2* |
| Atheriniformes |  | Atherinopsidae | *Leuresthes tenuis* | GU440373 | *CO1* |
| Atheriniformes |  | Atherinopsidae | *Membras martinica* | HQ691296 | *CYB* |
| Atheriniformes |  | Atherinopsidae | *Membras martinica* | EF602047 | *ND2* |
| Atheriniformes |  | Atherinopsidae | *Menidia audens* | HQ691304 | *CYB* |
| Atheriniformes |  | Atherinopsidae | *Menidia beryllina* | JN027207 | *CO1* |
| Atheriniformes |  | Atherinopsidae | *Menidia beryllina* | HQ691329 | *CYB* |
| Atheriniformes |  | Atherinopsidae | *Menidia beryllina* | EF602048 | *ND2* |
| Atheriniformes |  | Atherinopsidae | *Menidia colei* | EF602052 | *ND2* |
| Atheriniformes |  | Atherinopsidae | *Menidia conchorum* | EF602068 | *ND2* |
| Atheriniformes |  | Atherinopsidae | *Menidia extensa* | EF602055 | *ND2* |
| Atheriniformes |  | Atherinopsidae | *Menidia menidia* | AY290819 | *CO1* |
| Atheriniformes |  | Atherinopsidae | *Menidia menidia* | HQ691298 | *CYB* |
| Atheriniformes |  | Atherinopsidae | *Menidia menidia* | EF602050 | *ND2* |
| Atheriniformes |  | Atherinopsidae | *Menidia menidia* | AY430225 | *rag1* |
| Atheriniformes |  | Atherinopsidae | *Menidia menidia* | EU637977 | *rhod* |
| Atheriniformes |  | Atherinopsidae | *Menidia peninsulae* | HQ691299 | *CYB* |
| Atheriniformes |  | Atherinopsidae | *Menidia peninsulae* | EF602054 | *ND2* |
| Atheriniformes |  | Atherinopsidae | *Odontesthes argentinensis* | EU074512 | *CO1* |
| Atheriniformes |  | Atherinopsidae | *Odontesthes argentinensis* | GQ352664 | *CYB* |
| Atheriniformes |  | Atherinopsidae | *Odontesthes bonariensis* | FJ810255 | *CO1* |
| Atheriniformes |  | Atherinopsidae | *Odontesthes bonariensis* | GQ352667 | *CYB* |
| Atheriniformes |  | Atherinopsidae | *Odontesthes hatcheri* | GQ352683 | *CO1* |
| Atheriniformes |  | Atherinopsidae | *Odontesthes hatcheri* | GQ352668 | *CYB* |
| Atheriniformes |  | Atherinopsidae | *Odontesthes incisa* | FJ810254 | *CO1* |
| Atheriniformes |  | Atherinopsidae | *Odontesthes platensis* | EU074513 | *CO1* |
| Atheriniformes |  | Atherinopsidae | *Odontesthes regia* | FJ810257 | *CO1* |
| Atheriniformes |  | Atherinopsidae | *Odontesthes smitti* | GQ352677 | *CO1* |
| Atheriniformes |  | Atherinopsidae | *Poblana alchichica* | EU751902 | *CO1* |
| Atheriniformes |  | Atherinopsidae | *Poblana alchichica* | EF602110 | *ND2* |
| Atheriniformes |  | Atherinopsidae | *Poblana ferdebueni* | EF602100 | *ND2* |
| Atheriniformes |  | Atherinopsidae | *Poblana letholepis* | EU751904 | *CO1* |
| Atheriniformes |  | Atherinopsidae | *Poblana letholepis* | EF602105 | *ND2* |
| Atheriniformes |  | Atherinopsidae | *Poblana squamata* | EU751906 | *CO1* |
| Atheriniformes |  | Atherinopsidae | *Poblana squamata* | EF602116 | *ND2* |
| Atheriniformes |  | Notocheiridae | *Iso natalensis* | HQ945909 | *CO1* |
| Atheriniformes |  | Notocheiridae | *Iso natalensis* | HM855162 | *rhod* |
| Atheriniformes |  | Melanotaeniidae | *Bedotia geayi* | AY290799 | *CO1* |
| Atheriniformes |  | Melanotaeniidae | *Bedotia geayi* | EF095640 | *rag1* |
| Atheriniformes |  | Melanotaeniidae | *Bedotia geayi* | AY141267 | *rhod* |
| Atheriniformes |  | Melanotaeniidae | *Bedotia longianalis* | AY290802 | *CO1* |
| Atheriniformes |  | Melanotaeniidae | *Bedotia madagascariensis* | AY290794 | *CO1* |
| Atheriniformes |  | Melanotaeniidae | *Bedotia marojejy* | AY290792 | *CO1* |
| Atheriniformes |  | Melanotaeniidae | *Bedotia masoala* | AY290797 | *CO1* |
| Atheriniformes |  | Melanotaeniidae | *Chilatherina bleheri* | AY655519 | *CO1* |
| Atheriniformes |  | Melanotaeniidae | *Glossolepis incisus* | AY290814 | *CO1* |
| Atheriniformes |  | Melanotaeniidae | *Iriatherina werneri* | AY290813 | *CO1* |
| Atheriniformes |  | Melanotaeniidae | *Marosatherina ladigesi* | AY290808 | *CO1* |
| Atheriniformes |  | Melanotaeniidae | *Melanotaenia duboulayi* | HM007006 | *ATP6* |
| Atheriniformes |  | Melanotaeniidae | *Melanotaenia duboulayi* | HM006965 | *CO1* |
| Atheriniformes |  | Melanotaeniidae | *Melanotaenia duboulayi* | HM007047 | *CYB* |
| Atheriniformes |  | Melanotaeniidae | *Melanotaenia fluviatilis* | HM007007 | *ATP6* |
| Atheriniformes |  | Melanotaeniidae | *Melanotaenia fluviatilis* | HM006966 | *CO1* |
| Atheriniformes |  | Melanotaeniidae | *Melanotaenia fluviatilis* | HM007048 | *CYB* |
| Atheriniformes |  | Melanotaeniidae | *Melanotaenia praecox* | AY290815 | *CO1* |
| Atheriniformes |  | Melanotaeniidae | *Melanotaenia splendida* | HM007008 | *ATP6* |
| Atheriniformes |  | Melanotaeniidae | *Melanotaenia splendida* | HM006967 | *CO1* |
| Atheriniformes |  | Melanotaeniidae | *Melanotaenia splendida* | HM007049 | *CYB* |
| Atheriniformes |  | Melanotaeniidae | *Melanotaenia splendida splendida* | AY008738 | *ATP6* |
| Atheriniformes |  | Melanotaeniidae | *Melanotaenia splendida splendida* | GU288551 | *CYB* |
| Atheriniformes |  | Melanotaeniidae | *Melanotaenia splendida splendida* | GU288533 | *rag1* |
| Atheriniformes |  | Melanotaeniidae | *Pseudomugil gertrudae* | AY305900 | *ATP6* |
| Atheriniformes |  | Melanotaeniidae | *Pseudomugil gertrudae* | AY290817 | *CO1* |
| Atheriniformes |  | Melanotaeniidae | *Pseudomugil mellis* | AY334550 | *ATP6* |
| Atheriniformes |  | Melanotaeniidae | *Pseudomugil mellis* | HM006981 | *CO1* |
| Atheriniformes |  | Melanotaeniidae | *Pseudomugil mellis* | HM007063 | *CYB* |
| Atheriniformes |  | Melanotaeniidae | *Pseudomugil signifer* | AF272102 | *ATP6* |
| Atheriniformes |  | Melanotaeniidae | *Pseudomugil signifer* | AY655521 | *CO1* |
| Atheriniformes |  | Melanotaeniidae | *Pseudomugil signifer* | HM007064 | *CYB* |
| Atheriniformes |  | Melanotaeniidae | *Pseudomugil tenellus* | AY305897 | *ATP6* |
| Atheriniformes |  | Melanotaeniidae | *Rhadinocentrus ornatus* | HM007026 | *ATP6* |
| Atheriniformes |  | Melanotaeniidae | *Rhadinocentrus ornatus* | AY655520 | *CO1* |
| Atheriniformes |  | Melanotaeniidae | *Rhadinocentrus ornatus* | HM007069 | *CYB* |
| Atheriniformes |  | Melanotaeniidae | *Rheocles alaotrensis* | AY290804 | *CO1* |
| Atheriniformes |  | Melanotaeniidae | *Rheocles derhami* | AY290805 | *CO1* |
| Atheriniformes |  | Melanotaeniidae | *Rheocles lateralis* | AY290806 | *CO1* |
| Atheriniformes |  | Melanotaeniidae | *Rheocles vatosoa* | AY290807 | *CO1* |
| Atheriniformes |  | Melanotaeniidae | *Rheocles wrightae* | AY290803 | *CO1* |
| Atheriniformes |  | Phallostethidae | *Neostethus bicornis* | AY655527 | *CO1* |
| Atheriniformes |  | Atherinidae | *Alepidomus evermanni* | FN545672 | *CO1* |
| Atheriniformes |  | Atherinidae | *Atherina boyeri* | GQ352688 | *CO1* |
| Atheriniformes |  | Atherinidae | *Atherina boyeri* | EU036422 | *CYB* |
| Atheriniformes |  | Atherinidae | *Atherina boyeri* | EF439339 | *rhod* |
| Atheriniformes |  | Atherinidae | *Atherina breviceps* | GU804983 | *CO1* |
| Atheriniformes |  | Atherinidae | *Atherina breviceps* | HM855160 | *rhod* |
| Atheriniformes |  | Atherinidae | *Atherina hepsetus* | GQ352684 | *CO1* |
| Atheriniformes |  | Atherinidae | *Atherina hepsetus* | GQ352669 | *CYB* |
| Atheriniformes |  | Atherinidae | *Atherina hepsetus* | HM855173 | *rhod* |
| Atheriniformes |  | Atherinidae | *Atherina presbyter* | EF439189 | *CYB* |
| Atheriniformes |  | Atherinidae | *Atherina presbyter* | EF439256 | *rhod* |
| Atheriniformes |  | Atherinidae | *Atherinason hepsetoides* | GU932758 | *CYB* |
| Atheriniformes |  | Atherinidae | *Atherinomorus endrachtensis* | HQ654666 | *CO1* |
| Atheriniformes |  | Atherinidae | *Atherinomorus endrachtensis* | AB295519 | *CYB* |
| Atheriniformes |  | Atherinidae | *Atherinomorus lacunosus* | AY655522 | *CO1* |
| Atheriniformes |  | Atherinidae | *Atherinomorus lacunosus* | GU932753 | *CYB* |
| Atheriniformes |  | Atherinidae | *Atherinomorus ogilbyi* | AF031130 | *CYB* |
| Atheriniformes |  | Atherinidae | *Atherinomorus stipes* | FJ237592 | *CO1* |
| Atheriniformes |  | Atherinidae | *Atherinomorus stipes* | GU932754 | *CYB* |
| Atheriniformes |  | Atherinidae | *Atherinomorus vaigiensis* | GU932755 | *CYB* |
| Atheriniformes |  | Atherinidae | *Atherinops affinis* | AY290818 | *CO1* |
| Atheriniformes |  | Atherinidae | *Atherion elymus* | AY655528 | *CO1* |
| Atheriniformes |  | Atherinidae | *Craterocephalus amniculus* | JF292542 | *ATP6* |
| Atheriniformes |  | Atherinidae | *Craterocephalus amniculus* | GU932898 | *CYB* |
| Atheriniformes |  | Atherinidae | *Craterocephalus capreoli* | GU932792 | *CYB* |
| Atheriniformes |  | Atherinidae | *Craterocephalus centralis* | JF292561 | *ATP6* |
| Atheriniformes |  | Atherinidae | *Craterocephalus centralis* | GU932887 | *CYB* |
| Atheriniformes |  | Atherinidae | *Craterocephalus cuneiceps* | JF292572 | *ATP6* |
| Atheriniformes |  | Atherinidae | *Craterocephalus cuneiceps* | GU932771 | *CYB* |
| Atheriniformes |  | Atherinidae | *Craterocephalus dalhousiensis* | GU932880 | *CYB* |
| Atheriniformes |  | Atherinidae | *Craterocephalus eyresii* | JF292537 | *CYB* |
| Atheriniformes |  | Atherinidae | *Craterocephalus fluviatilis* | JF292550 | *ATP6* |
| Atheriniformes |  | Atherinidae | *Craterocephalus fluviatilis* | JF292523 | *CYB* |
| Atheriniformes |  | Atherinidae | *Craterocephalus gloveri* | GU932883 | *CYB* |
| Atheriniformes |  | Atherinidae | *Craterocephalus helenae* | GU932774 | *CYB* |
| Atheriniformes |  | Atherinidae | *Craterocephalus honoriae* | AY655524 | *CO1* |
| Atheriniformes |  | Atherinidae | *Craterocephalus honoriae* | GU932765 | *CYB* |
| Atheriniformes |  | Atherinidae | *Craterocephalus lentigenosus* | GU932803 | *CYB* |
| Atheriniformes |  | Atherinidae | *Craterocephalus marianae* | GU932776 | *CYB* |
| Atheriniformes |  | Atherinidae | *Craterocephalus marjoriae* | JF292573 | *ATP6* |
| Atheriniformes |  | Atherinidae | *Craterocephalus marjoriae* | HM006953 | *CO1* |
| Atheriniformes |  | Atherinidae | *Craterocephalus marjoriae* | GU932780 | *CYB* |
| Atheriniformes |  | Atherinidae | *Craterocephalus mugiloides* | GU932767 | *CYB* |
| Atheriniformes |  | Atherinidae | *Craterocephalus munroi* | JF292574 | *ATP6* |
| Atheriniformes |  | Atherinidae | *Craterocephalus munroi* | GU932778 | *CYB* |
| Atheriniformes |  | Atherinidae | *Craterocephalus nouhuysi* | GU932801 | *CYB* |
| Atheriniformes |  | Atherinidae | *Craterocephalus pauciradiatus* | GU932794 | *CYB* |
| Atheriniformes |  | Atherinidae | *Craterocephalus randi* | AF271895 | *ATP6* |
| Atheriniformes |  | Atherinidae | *Craterocephalus randi* | GU932798 | *CYB* |
| Atheriniformes |  | Atherinidae | *Craterocephalus stercusmuscarum* | HM006996 | *ATP6* |
| Atheriniformes |  | Atherinidae | *Craterocephalus stercusmuscarum* | HM006954 | *CO1* |
| Atheriniformes |  | Atherinidae | *Craterocephalus stercusmuscarum* | HM007036 | *CYB* |
| Atheriniformes |  | Atherinidae | *Craterocephalus stercusmuscarum fulvus* | JF292575 | *ATP6* |
| Atheriniformes |  | Atherinidae | *Craterocephalus stercusmuscarum fulvus* | GU932851 | *CYB* |
| Atheriniformes |  | Atherinidae | *Craterocephalus stramineus* | HM006997 | *ATP6* |
| Atheriniformes |  | Atherinidae | *Craterocephalus stramineus* | HM006955 | *CO1* |
| Atheriniformes |  | Atherinidae | *Craterocephalus stramineus* | GU932787 | *CYB* |
| Atheriniformes |  | Atherinidae | *Hypoatherina harringtonensis* | GU932756 | *CYB* |
| Atheriniformes |  | Atherinidae | *Hypoatherina temminckii* | GU932757 | *CYB* |
| Atheriniformes |  | Atherinidae | *Hypoatherina valenciennei* | JF952761 | *CO1* |
| Atheriniformes |  | Atherinidae | *Kestratherina brevirostris* | GU932761 | *CYB* |
| Atheriniformes |  | Atherinidae | *Kestratherina esox* | GU932762 | *CYB* |
| Atheriniformes |  | Atherinidae | *Leptatherina presbyteroides* | GU932763 | *CYB* |
| Atheriniformes |  | Atherinidae | *Leptatherina wallacei* | GU932764 | *CYB* |
| Atheriniformes |  | Atherinidae | *Teramulus waterloti* | AY290811 | *CO1* |
| Beloniformes |  | Adrianichthyidae | *Oryzias carnaticus* | AF257253 | *CYB* |
| Beloniformes |  | Adrianichthyidae | *Oryzias celebensis* | AF257254 | *CYB* |
| Beloniformes |  | Adrianichthyidae | *Oryzias curvinotus* | AB084754 | *CYB* |
| Beloniformes |  | Adrianichthyidae | *Oryzias dancena* | GU731586 | *CYB* |
| Beloniformes |  | Adrianichthyidae | *Oryzias javanicus* | AF257258 | *CYB* |
| Beloniformes |  | Adrianichthyidae | *Oryzias latipes* | AB480878 | *CYB* |
| Beloniformes |  | Adrianichthyidae | *Oryzias latipes* | EF095641 | *rag1* |
| Beloniformes |  | Adrianichthyidae | *Oryzias luzonensis* | AB084755 | *CYB* |
| Beloniformes |  | Adrianichthyidae | *Oryzias marmoratus* | AF257261 | *CYB* |
| Beloniformes |  | Adrianichthyidae | *Oryzias matanensis* | AF243916 | *CYB* |
| Beloniformes |  | Adrianichthyidae | *Oryzias mekongensis* | AB084756 | *CYB* |
| Beloniformes |  | Adrianichthyidae | *Oryzias minutillus* | AF257263 | *CYB* |
| Beloniformes |  | Adrianichthyidae | *Oryzias nigrimas* | AF257264 | *CYB* |
| Beloniformes |  | Adrianichthyidae | *Oryzias sinensis* | HQ536426 | *CO1* |
| Beloniformes |  | Adrianichthyidae | *Oryzias sinensis* | GU731595 | *CYB* |
| Beloniformes |  | Adrianichthyidae | *Xenopoecilus oophorus* | AF257265 | *CYB* |
| Beloniformes |  | Adrianichthyidae | *Xenopoecilus sarasinorum* | AF257266 | *CYB* |
| Beloniformes |  | Exocoetidae | *Cheilopogon abei* | HQ325605 | *CYB* |
| Beloniformes |  | Exocoetidae | *Cheilopogon abei* | HQ325672 | *rag2* |
| Beloniformes |  | Exocoetidae | *Cheilopogon agoo agoo* | AB569473 | *CYB* |
| Beloniformes |  | Exocoetidae | *Cheilopogon antoncichi* | AB569471 | *CYB* |
| Beloniformes |  | Exocoetidae | *Cheilopogon atrisignis* | HQ325606 | *CYB* |
| Beloniformes |  | Exocoetidae | *Cheilopogon atrisignis* | HQ325674 | *rag2* |
| Beloniformes |  | Exocoetidae | *Cheilopogon cyanopterus* | HQ678690 | *CYB* |
| Beloniformes |  | Exocoetidae | *Cheilopogon cyanopterus* | HQ325675 | *rag2* |
| Beloniformes |  | Exocoetidae | *Cheilopogon doederleinii* | AB569475 | *CYB* |
| Beloniformes |  | Exocoetidae | *Cheilopogon dorsomacula* | HQ325610 | *CYB* |
| Beloniformes |  | Exocoetidae | *Cheilopogon dorsomacula* | AY693569 | *rag2* |
| Beloniformes |  | Exocoetidae | *Cheilopogon exsiliens* | HQ325612 | *CYB* |
| Beloniformes |  | Exocoetidae | *Cheilopogon exsiliens* | HQ325679 | *rag2* |
| Beloniformes |  | Exocoetidae | *Cheilopogon furcatus* | HQ325615 | *CYB* |
| Beloniformes |  | Exocoetidae | *Cheilopogon furcatus* | HQ325682 | *rag2* |
| Beloniformes |  | Exocoetidae | *Cheilopogon heterurus* | EU637950 | *rhod* |
| Beloniformes |  | Exocoetidae | *Cheilopogon melanurus* | HQ325617 | *CYB* |
| Beloniformes |  | Exocoetidae | *Cheilopogon melanurus* | AY693528 | *rag2* |
| Beloniformes |  | Exocoetidae | *Cheilopogon pinnatibarbatus* | JF952700 | *CO1* |
| Beloniformes |  | Exocoetidae | *Cheilopogon pinnatibarbatus californicus* | HQ325618 | *CYB* |
| Beloniformes |  | Exocoetidae | *Cheilopogon pinnatibarbatus californicus* | HQ325685 | *rag2* |
| Beloniformes |  | Exocoetidae | *Cheilopogon spilonotopterus* | HQ678692 | *CYB* |
| Beloniformes |  | Exocoetidae | *Cheilopogon spilonotopterus* | AY693574 | *rag2* |
| Beloniformes |  | Exocoetidae | *Cheilopogon unicolor* | HQ678693 | *CYB* |
| Beloniformes |  | Exocoetidae | *Cheilopogon xenopterus* | HQ325622 | *CYB* |
| Beloniformes |  | Exocoetidae | *Cheilopogon xenopterus* | HQ325689 | *rag2* |
| Beloniformes |  | Exocoetidae | *Cypselurus angusticeps* | HQ325624 | *CYB* |
| Beloniformes |  | Exocoetidae | *Cypselurus angusticeps* | HQ325691 | *rag2* |
| Beloniformes |  | Exocoetidae | *Cypselurus callopterus* | HQ325626 | *CYB* |
| Beloniformes |  | Exocoetidae | *Cypselurus callopterus* | HQ325692 | *rag2* |
| Beloniformes |  | Exocoetidae | *Cypselurus hexazona* | HQ325627 | *CYB* |
| Beloniformes |  | Exocoetidae | *Cypselurus hexazona* | HQ325694 | *rag2* |
| Beloniformes |  | Exocoetidae | *Cypselurus pinnatibarbatus japonicus* | AB569470 | *CYB* |
| Beloniformes |  | Exocoetidae | *Cypselurus poecilopterus* | HQ678691 | *CYB* |
| Beloniformes |  | Exocoetidae | *Euleptorhamphus viridis* | AY693573 | *rag2* |
| Beloniformes |  | Exocoetidae | *Exocoetus monocirrhus* | HQ945899 | *CO1* |
| Beloniformes |  | Exocoetidae | *Exocoetus monocirrhus* | HQ325629 | *CYB* |
| Beloniformes |  | Exocoetidae | *Exocoetus monocirrhus* | AY693567 | *rag2* |
| Beloniformes |  | Exocoetidae | *Exocoetus obtusirostris* | HQ325630 | *CYB* |
| Beloniformes |  | Exocoetidae | *Exocoetus obtusirostris* | HQ325697 | *rag2* |
| Beloniformes |  | Exocoetidae | *Exocoetus peruvianus* | HQ325633 | *CYB* |
| Beloniformes |  | Exocoetidae | *Exocoetus peruvianus* | HQ325700 | *rag2* |
| Beloniformes |  | Exocoetidae | *Exocoetus volitans* | HQ325636 | *CYB* |
| Beloniformes |  | Exocoetidae | *Exocoetus volitans* | HQ325703 | *rag2* |
| Beloniformes |  | Exocoetidae | *Fodiator acutus* | HQ010079 | *CO1* |
| Beloniformes |  | Exocoetidae | *Fodiator acutus* | AY693500 | *CYB* |
| Beloniformes |  | Exocoetidae | *Fodiator acutus* | AY693570 | *rag2* |
| Beloniformes |  | Exocoetidae | *Fodiator rostratus* | HQ325638 | *CYB* |
| Beloniformes |  | Exocoetidae | *Fodiator rostratus* | HQ325706 | *rag2* |
| Beloniformes |  | Exocoetidae | *Hirundichthys affinis* | HQ325642 | *CYB* |
| Beloniformes |  | Exocoetidae | *Hirundichthys affinis* | HQ325708 | *rag2* |
| Beloniformes |  | Exocoetidae | *Hirundichthys albimaculatus* | HQ325643 | *CYB* |
| Beloniformes |  | Exocoetidae | *Hirundichthys albimaculatus* | HQ325710 | *rag2* |
| Beloniformes |  | Exocoetidae | *Hirundichthys marginatus* | HQ325645 | *CYB* |
| Beloniformes |  | Exocoetidae | *Hirundichthys marginatus* | AY693562 | *rag2* |
| Beloniformes |  | Exocoetidae | *Hirundichthys oxycephalus* | JF952756 | *CO1* |
| Beloniformes |  | Exocoetidae | *Hirundichthys rondeletii* | EF607404 | *CO1* |
| Beloniformes |  | Exocoetidae | *Hirundichthys speculiger* | HQ325653 | *CYB* |
| Beloniformes |  | Exocoetidae | *Hirundichthys speculiger* | HQ325719 | *rag2* |
| Beloniformes |  | Exocoetidae | *Hirundichthys volador* | HQ325646 | *CYB* |
| Beloniformes |  | Exocoetidae | *Hirundichthys volador* | HQ325714 | *rag2* |
| Beloniformes |  | Exocoetidae | *Parexocoetus brachypterus* | HQ325656 | *CYB* |
| Beloniformes |  | Exocoetidae | *Parexocoetus brachypterus* | HQ325723 | *rag2* |
| Beloniformes |  | Exocoetidae | *Parexocoetus hillianus* | HQ325659 | *CYB* |
| Beloniformes |  | Exocoetidae | *Parexocoetus hillianus* | HQ325725 | *rag2* |
| Beloniformes |  | Exocoetidae | *Parexocoetus mento* | HQ945954 | *CO1* |
| Beloniformes |  | Exocoetidae | *Parexocoetus mento* | HQ325660 | *CYB* |
| Beloniformes |  | Exocoetidae | *Parexocoetus mento* | HQ325728 | *rag2* |
| Beloniformes |  | Exocoetidae | *Prognichthys gibbifrons* | HQ325662 | *CYB* |
| Beloniformes |  | Exocoetidae | *Prognichthys gibbifrons* | HQ325729 | *rag2* |
| Beloniformes |  | Exocoetidae | *Prognichthys glaphyrae* | HQ325664 | *CYB* |
| Beloniformes |  | Exocoetidae | *Prognichthys glaphyrae* | HQ325730 | *rag2* |
| Beloniformes |  | Exocoetidae | *Prognichthys occidentalis* | HQ325666 | *CYB* |
| Beloniformes |  | Exocoetidae | *Prognichthys occidentalis* | HQ325732 | *rag2* |
| Beloniformes |  | Exocoetidae | *Prognichthys sealei* | HQ325668 | *CYB* |
| Beloniformes |  | Exocoetidae | *Prognichthys sealei* | HQ325734 | *rag2* |
| Beloniformes |  | Exocoetidae | *Prognichthys tringa* | HQ325670 | *CYB* |
| Beloniformes |  | Exocoetidae | *Prognichthys tringa* | AY693565 | *rag2* |
| Beloniformes |  | Hemiramphidae | *Arrhamphus sclerolepis* | AY693511 | *CYB* |
| Beloniformes |  | Hemiramphidae | *Chriodorus atherinoides* | GU225191 | *CO1* |
| Beloniformes |  | Hemiramphidae | *Chriodorus atherinoides* | AF243924 | *CYB* |
| Beloniformes |  | Hemiramphidae | *Euleptorhamphus viridis* | AY693501 | *CYB* |
| Beloniformes |  | Hemiramphidae | *Hemiramphus archipelagicus* | HQ149857 | *CO1* |
| Beloniformes |  | Hemiramphidae | *Hemiramphus balao* | AF243873 | *CYB* |
| Beloniformes |  | Hemiramphidae | *Hemiramphus balao* | AY693530 | *rag2* |
| Beloniformes |  | Hemiramphidae | *Hemiramphus brasiliensis* | AF243864 | *CYB* |
| Beloniformes |  | Hemiramphidae | *Hemiramphus brasiliensis* | AY693523 | *rag2* |
| Beloniformes |  | Hemiramphidae | *Hemiramphus far* | EU148546 | *CO1* |
| Beloniformes |  | Hemiramphidae | *Hemiramphus far* | AY693517 | *CYB* |
| Beloniformes |  | Hemiramphidae | *Hemiramphus far* | AY693583 | *rag2* |
| Beloniformes |  | Hemiramphidae | *Hemirhamphodon pogonognathus* | AF243930 | *CYB* |
| Beloniformes |  | Hemiramphidae | *Hemirhamphodon pogonognathus* | AY693559 | *rag2* |
| Beloniformes |  | Hemiramphidae | *Hyporhamphus affinis* | EF609376 | *CO1* |
| Beloniformes |  | Hemiramphidae | *Hyporhamphus dussumieri* | EF607400 | *CO1* |
| Beloniformes |  | Hemiramphidae | *Hyporhamphus limbatus* | FJ237993 | *CO1* |
| Beloniformes |  | Hemiramphidae | *Hyporhamphus quoyi* | FJ237994 | *CO1* |
| Beloniformes |  | Hemiramphidae | *Hyporhamphus quoyi* | AF243920 | *CYB* |
| Beloniformes |  | Hemiramphidae | *Hyporhamphus quoyi* | AY693551 | *rag2* |
| Beloniformes |  | Hemiramphidae | *Hyporhamphus sajori* | JF952763 | *CO1* |
| Beloniformes |  | Hemiramphidae | *Hyporhamphus sajori* | AY693508 | *CYB* |
| Beloniformes |  | Hemiramphidae | *Hyporhamphus sajori* | AY693579 | *rag2* |
| Beloniformes |  | Hemiramphidae | *Hyporhamphus unifasciatus* | AF231638 | *ATP6* |
| Beloniformes |  | Hemiramphidae | *Hyporhamphus unifasciatus* | GU225337 | *CO1* |
| Beloniformes |  | Hemiramphidae | *Hyporhamphus unifasciatus* | AF231665 | *CYB* |
| Beloniformes |  | Hemiramphidae | *Hyporhamphus xanthopterus* | EU148544 | *CO1* |
| Beloniformes |  | Hemiramphidae | *Nomorhamphus ravnaki* | AF243925 | *CYB* |
| Beloniformes |  | Hemiramphidae | *Nomorhamphus ravnaki* | AY693555 | *rag2* |
| Beloniformes |  | Hemiramphidae | *Nomorhamphus weberi* | AF243928 | *CYB* |
| Beloniformes |  | Hemiramphidae | *Nomorhamphus weberi* | AY693557 | *rag2* |
| Beloniformes |  | Hemiramphidae | *Oxyporhamphus micropterus* | AY693489 | *CYB* |
| Beloniformes |  | Hemiramphidae | *Oxyporhamphus micropterus* | AY693560 | *rag2* |
| Beloniformes |  | Hemiramphidae | *Oxyporhamphus micropterus similis* | HQ325654 | *CYB* |
| Beloniformes |  | Hemiramphidae | *Oxyporhamphus micropterus similis* | HQ325721 | *rag2* |
| Beloniformes |  | Hemiramphidae | *Zenarchopterus buffonis* | AY693554 | *rag2* |
| Beloniformes |  | Belonidae | *Ablennes hians* | AF231583 | *ATP6* |
| Beloniformes |  | Belonidae | *Ablennes hians* | JF952659 | *CO1* |
| Beloniformes |  | Belonidae | *Ablennes hians* | AY693520 | *rag2* |
| Beloniformes |  | Belonidae | *Belone belone* | AF231584 | *ATP6* |
| Beloniformes |  | Belonidae | *Belone belone* | EU492078 | *CYB* |
| Beloniformes |  | Belonidae | *Belone belone* | AY693547 | *rag2* |
| Beloniformes |  | Belonidae | *Belone svetovidovi* | AF243880 | *CYB* |
| Beloniformes |  | Belonidae | *Belone svetovidovi* | AY693531 | *rag2* |
| Beloniformes |  | Belonidae | *Belonion apodion* | AF185092 | *CYB* |
| Beloniformes |  | Belonidae | *Belonion apodion* | AF306488 | *rag2* |
| Beloniformes |  | Belonidae | *Belonion dibranchodon* | AF185091 | *CYB* |
| Beloniformes |  | Belonidae | *Belonion dibranchodon* | AF306469 | *rag2* |
| Beloniformes |  | Belonidae | *Petalichthys capensis* | AY693507 | *CYB* |
| Beloniformes |  | Belonidae | *Petalichthys capensis* | AY693577 | *rag2* |
| Beloniformes |  | Belonidae | *Platybelone argala* | AF243874 | *CYB* |
| Beloniformes |  | Belonidae | *Platybelone argala* | AF306465 | *rag2* |
| Beloniformes |  | Belonidae | *Potamorrhaphis eigenmanni* | AF185079 | *CYB* |
| Beloniformes |  | Belonidae | *Potamorrhaphis eigenmanni* | AF306470 | *rag2* |
| Beloniformes |  | Belonidae | *Potamorrhaphis guianensis* | AF185073 | *CYB* |
| Beloniformes |  | Belonidae | *Potamorrhaphis guianensis* | AF306466 | *rag2* |
| Beloniformes |  | Belonidae | *Potamorrhaphis petersi* | AF185090 | *CYB* |
| Beloniformes |  | Belonidae | *Potamorrhaphis petersi* | AF306474 | *rag2* |
| Beloniformes |  | Belonidae | *Pseudotylosurus angusticeps* | AF186104 | *CYB* |
| Beloniformes |  | Belonidae | *Pseudotylosurus angusticeps* | AF306486 | *rag2* |
| Beloniformes |  | Belonidae | *Pseudotylosurus microps* | AF186102 | *CYB* |
| Beloniformes |  | Belonidae | *Pseudotylosurus microps* | AY693584 | *rag2* |
| Beloniformes |  | Belonidae | *Strongylura anastomella* | AY693515 | *CYB* |
| Beloniformes |  | Belonidae | *Strongylura exilis* | AF231586 | *ATP6* |
| Beloniformes |  | Belonidae | *Strongylura exilis* | AF231641 | *CYB* |
| Beloniformes |  | Belonidae | *Strongylura exilis* | AF306483 | *rag2* |
| Beloniformes |  | Belonidae | *Strongylura fluviatilis* | AF231590 | *ATP6* |
| Beloniformes |  | Belonidae | *Strongylura fluviatilis* | AF231643 | *CYB* |
| Beloniformes |  | Belonidae | *Strongylura fluviatilis* | AF306478 | *rag2* |
| Beloniformes |  | Belonidae | *Strongylura hubbsi* | AF231592 | *ATP6* |
| Beloniformes |  | Belonidae | *Strongylura hubbsi* | AF231644 | *CYB* |
| Beloniformes |  | Belonidae | *Strongylura hubbsi* | AF306480 | *rag2* |
| Beloniformes |  | Belonidae | *Strongylura incisa* | AF231593 | *ATP6* |
| Beloniformes |  | Belonidae | *Strongylura incisa* | AF231646 | *CYB* |
| Beloniformes |  | Belonidae | *Strongylura incisa* | AY693533 | *rag2* |
| Beloniformes |  | Belonidae | *Strongylura krefftii* | AF243900 | *CYB* |
| Beloniformes |  | Belonidae | *Strongylura krefftii* | AY693540 | *rag2* |
| Beloniformes |  | Belonidae | *Strongylura leiura* | FJ237566 | *CO1* |
| Beloniformes |  | Belonidae | *Strongylura leiura* | AF243901 | *CYB* |
| Beloniformes |  | Belonidae | *Strongylura leiura* | AY693542 | *rag2* |
| Beloniformes |  | Belonidae | *Strongylura marina* | AF231597 | *ATP6* |
| Beloniformes |  | Belonidae | *Strongylura marina* | AF231647 | *CYB* |
| Beloniformes |  | Belonidae | *Strongylura marina* | AF306462 | *rag2* |
| Beloniformes |  | Belonidae | *Strongylura notata* | AF231605 | *ATP6* |
| Beloniformes |  | Belonidae | *Strongylura notata* | GU225487 | *CO1* |
| Beloniformes |  | Belonidae | *Strongylura notata* | AF231650 | *CYB* |
| Beloniformes |  | Belonidae | *Strongylura notata* | AF306489 | *rag2* |
| Beloniformes |  | Belonidae | *Strongylura notata forsythia* | AF243856 | *CYB* |
| Beloniformes |  | Belonidae | *Strongylura notata forsythia* | AY693519 | *rag2* |
| Beloniformes |  | Belonidae | *Strongylura notata notata* | AF243857 | *CYB* |
| Beloniformes |  | Belonidae | *Strongylura scapularis* | AF231607 | *ATP6* |
| Beloniformes |  | Belonidae | *Strongylura scapularis* | AF231652 | *CYB* |
| Beloniformes |  | Belonidae | *Strongylura scapularis* | AF306487 | *rag2* |
| Beloniformes |  | Belonidae | *Strongylura senegalensis* | AF231608 | *ATP6* |
| Beloniformes |  | Belonidae | *Strongylura senegalensis* | AF231653 | *CYB* |
| Beloniformes |  | Belonidae | *Strongylura senegalensis* | AF306484 | *rag2* |
| Beloniformes |  | Belonidae | *Strongylura strongylura* | EU014257 | *CO1* |
| Beloniformes |  | Belonidae | *Strongylura strongylura* | AF243887 | *CYB* |
| Beloniformes |  | Belonidae | *Strongylura strongylura* | AY693535 | *rag2* |
| Beloniformes |  | Belonidae | *Strongylura timucu* | AF231611 | *ATP6* |
| Beloniformes |  | Belonidae | *Strongylura timucu* | GU225492 | *CO1* |
| Beloniformes |  | Belonidae | *Strongylura timucu* | AF231654 | *CYB* |
| Beloniformes |  | Belonidae | *Strongylura timucu* | AF306461 | *rag2* |
| Beloniformes |  | Belonidae | *Tylosurus acus acus* | AF231620 | *ATP6* |
| Beloniformes |  | Belonidae | *Tylosurus acus acus* | EF392635 | *CYB* |
| Beloniformes |  | Belonidae | *Tylosurus acus acus* | AY693521 | *rag2* |
| Beloniformes |  | Belonidae | *Tylosurus acus imperialis* | AF231622 | *ATP6* |
| Beloniformes |  | Belonidae | *Tylosurus acus imperialis* | AF231657 | *CYB* |
| Beloniformes |  | Belonidae | *Tylosurus acus melanotus* | AF231623 | *ATP6* |
| Beloniformes |  | Belonidae | *Tylosurus acus melanotus* | AF231659 | *CYB* |
| Beloniformes |  | Belonidae | *Tylosurus acus melanotus* | AY693522 | *rag2* |
| Beloniformes |  | Belonidae | *Tylosurus acus rafale* | AF231629 | *ATP6* |
| Beloniformes |  | Belonidae | *Tylosurus acus rafale* | AF231661 | *CYB* |
| Beloniformes |  | Belonidae | *Tylosurus crocodilus* | AF231632 | *ATP6* |
| Beloniformes |  | Belonidae | *Tylosurus crocodilus* | GU805099 | *CO1* |
| Beloniformes |  | Belonidae | *Tylosurus crocodilus* | AF231662 | *CYB* |
| Beloniformes |  | Belonidae | *Tylosurus crocodilus* | AY693536 | *rag2* |
| Beloniformes |  | Belonidae | *Tylosurus crocodilus crocodilus* | AF231634 | *ATP6* |
| Beloniformes |  | Belonidae | *Tylosurus crocodilus crocodilus* | HQ149963 | *CO1* |
| Beloniformes |  | Belonidae | *Tylosurus crocodilus crocodilus* | AF231664 | *CYB* |
| Beloniformes |  | Belonidae | *Tylosurus gavialoides* | AF243904 | *CYB* |
| Beloniformes |  | Belonidae | *Tylosurus gavialoides* | AY693544 | *rag2* |
| Beloniformes |  | Belonidae | *Tylosurus pacificus* | AF231628 | *ATP6* |
| Beloniformes |  | Belonidae | *Tylosurus pacificus* | AF231660 | *CYB* |
| Beloniformes |  | Belonidae | *Tylosurus punctulatus* | AF243905 | *CYB* |
| Beloniformes |  | Belonidae | *Tylosurus punctulatus* | AY693545 | *rag2* |
| Beloniformes |  | Belonidae | *Xenentodon cancila* | AF243891 | *CYB* |
| Beloniformes |  | Belonidae | *Xenentodon cancila* | AF306473 | *rag2* |
| Beloniformes |  | Scomberesocidae | *Cololabis adocetus* | AY693512 | *CYB* |
| Beloniformes |  | Scomberesocidae | *Cololabis adocetus* | AY693580 | *rag2* |
| Beloniformes |  | Scomberesocidae | *Cololabis saira* | EU595078 | *CO1* |
| Beloniformes |  | Scomberesocidae | *Cololabis saira* | AY754822 | *CYB* |
| Beloniformes |  | Scomberesocidae | *Cololabis saira* | AY693550 | *rag2* |
| Beloniformes |  | Scomberesocidae | *Scomberesox saurus* | AB355965 | *CYB* |
| Beloniformes |  | Scomberesocidae | *Scomberesox saurus* | AY308771 | *rag1* |
| Beloniformes |  | Scomberesocidae | *Scomberesox saurus* | AF306481 | *rag2* |
| Cyprinodontiformes |  | Aplocheilidae | *Aplocheilus panchax* | EF455702 | *ND2* |
| Cyprinodontiformes |  | Nothobranchiidae | *Aphyosemion ahli* | EF417002 | *CO1* |
| Cyprinodontiformes |  | Nothobranchiidae | *Aphyosemion ahli* | EU272798 | *CYB* |
| Cyprinodontiformes |  | Nothobranchiidae | *Aphyosemion australe* | EF417017 | *CO1* |
| Cyprinodontiformes |  | Nothobranchiidae | *Aphyosemion australe* | EU272816 | *CYB* |
| Cyprinodontiformes |  | Nothobranchiidae | *Aphyosemion calliurum* | EF417030 | *CO1* |
| Cyprinodontiformes |  | Nothobranchiidae | *Aphyosemion calliurum* | EU272797 | *CYB* |
| Cyprinodontiformes |  | Nothobranchiidae | *Aphyosemion cameronense* | AY748279 | *CYB* |
| Cyprinodontiformes |  | Nothobranchiidae | *Aphyosemion celiae* | EF417034 | *CO1* |
| Cyprinodontiformes |  | Nothobranchiidae | *Aphyosemion celiae* | EU885234 | *CYB* |
| Cyprinodontiformes |  | Nothobranchiidae | *Aphyosemion celiae winifredae* | EU885233 | *CYB* |
| Cyprinodontiformes |  | Nothobranchiidae | *Aphyosemion edeanum* | EF417038 | *CO1* |
| Cyprinodontiformes |  | Nothobranchiidae | *Aphyosemion edeanum* | EU272812 | *CYB* |
| Cyprinodontiformes |  | Nothobranchiidae | *Aphyosemion elberti* | AF092355 | *ND2* |
| Cyprinodontiformes |  | Nothobranchiidae | *Aphyosemion etsamense* | AY748294 | *CYB* |
| Cyprinodontiformes |  | Nothobranchiidae | *Aphyosemion festivum* | EF417040 | *CO1* |
| Cyprinodontiformes |  | Nothobranchiidae | *Aphyosemion franzwerneri* | EF417044 | *CO1* |
| Cyprinodontiformes |  | Nothobranchiidae | *Aphyosemion franzwerneri* | EU885237 | *CYB* |
| Cyprinodontiformes |  | Nothobranchiidae | *Aphyosemion heinemanni* | EF417045 | *CO1* |
| Cyprinodontiformes |  | Nothobranchiidae | *Aphyosemion heinemanni* | EU885236 | *CYB* |
| Cyprinodontiformes |  | Nothobranchiidae | *Aphyosemion herzogi* | EU885235 | *CYB* |
| Cyprinodontiformes |  | Nothobranchiidae | *Aphyosemion lividum* | EU282845 | *CO1* |
| Cyprinodontiformes |  | Nothobranchiidae | *Aphyosemion lugens* | EU885232 | *CYB* |
| Cyprinodontiformes |  | Nothobranchiidae | *Aphyosemion mimbon* | AY748288 | *CYB* |
| Cyprinodontiformes |  | Nothobranchiidae | *Aphyosemion pascheni* | EF417046 | *CO1* |
| Cyprinodontiformes |  | Nothobranchiidae | *Archiaphyosemion guineense* | FJ872058 | *ND2* |
| Cyprinodontiformes |  | Nothobranchiidae | *Chromaphyosemion alpha* | DQ267387 | *CO1* |
| Cyprinodontiformes |  | Nothobranchiidae | *Chromaphyosemion alpha* | EU056925 | *CYB* |
| Cyprinodontiformes |  | Nothobranchiidae | *Chromaphyosemion bitaeniatum* | DQ267390 | *CO1* |
| Cyprinodontiformes |  | Nothobranchiidae | *Chromaphyosemion bitaeniatum* | DQ522281 | *CYB* |
| Cyprinodontiformes |  | Nothobranchiidae | *Chromaphyosemion bivittatum* | AF002576 | *CO1* |
| Cyprinodontiformes |  | Nothobranchiidae | *Chromaphyosemion bivittatum* | DQ522269 | *CYB* |
| Cyprinodontiformes |  | Nothobranchiidae | *Chromaphyosemion ecucuense* | EU249507 | *CYB* |
| Cyprinodontiformes |  | Nothobranchiidae | *Chromaphyosemion erythron* | EU249500 | *CYB* |
| Cyprinodontiformes |  | Nothobranchiidae | *Chromaphyosemion kouamense* | EF063366 | *CO1* |
| Cyprinodontiformes |  | Nothobranchiidae | *Chromaphyosemion kouamense* | EU249497 | *CYB* |
| Cyprinodontiformes |  | Nothobranchiidae | *Chromaphyosemion koungueense* | EU056927 | *CYB* |
| Cyprinodontiformes |  | Nothobranchiidae | *Chromaphyosemion loennbergii* | DQ267372 | *CO1* |
| Cyprinodontiformes |  | Nothobranchiidae | *Chromaphyosemion loennbergii* | DQ342225 | *CYB* |
| Cyprinodontiformes |  | Nothobranchiidae | *Chromaphyosemion lugens* | DQ267356 | *CO1* |
| Cyprinodontiformes |  | Nothobranchiidae | *Chromaphyosemion lugens* | EU056930 | *CYB* |
| Cyprinodontiformes |  | Nothobranchiidae | *Chromaphyosemion malumbresi* | EF185308 | *CO1* |
| Cyprinodontiformes |  | Nothobranchiidae | *Chromaphyosemion malumbresi* | EU249510 | *CYB* |
| Cyprinodontiformes |  | Nothobranchiidae | *Chromaphyosemion melanogaster* | DQ267299 | *CO1* |
| Cyprinodontiformes |  | Nothobranchiidae | *Chromaphyosemion melanogaster* | DQ342224 | *CYB* |
| Cyprinodontiformes |  | Nothobranchiidae | *Chromaphyosemion melinoeides* | EU056933 | *CYB* |
| Cyprinodontiformes |  | Nothobranchiidae | *Chromaphyosemion omega* | EU056934 | *CYB* |
| Cyprinodontiformes |  | Nothobranchiidae | *Chromaphyosemion poliaki* | DQ267406 | *CO1* |
| Cyprinodontiformes |  | Nothobranchiidae | *Chromaphyosemion poliaki* | EU056935 | *CYB* |
| Cyprinodontiformes |  | Nothobranchiidae | *Chromaphyosemion punctulatum* | DQ267346 | *CO1* |
| Cyprinodontiformes |  | Nothobranchiidae | *Chromaphyosemion punctulatum* | EU056937 | *CYB* |
| Cyprinodontiformes |  | Nothobranchiidae | *Chromaphyosemion riggenbachi* | DQ267411 | *CO1* |
| Cyprinodontiformes |  | Nothobranchiidae | *Chromaphyosemion riggenbachi* | DQ342205 | *CYB* |
| Cyprinodontiformes |  | Nothobranchiidae | *Chromaphyosemion splendopleure* | DQ267268 | *CO1* |
| Cyprinodontiformes |  | Nothobranchiidae | *Chromaphyosemion splendopleure* | EU056941 | *CYB* |
| Cyprinodontiformes |  | Nothobranchiidae | *Chromaphyosemion volcanum* | DQ267286 | *CO1* |
| Cyprinodontiformes |  | Nothobranchiidae | *Diapteron georgiae* | DQ981775 | *CYB* |
| Cyprinodontiformes |  | Nothobranchiidae | *Epiplatys sexfasciatus* | DQ981783 | *CYB* |
| Cyprinodontiformes |  | Nothobranchiidae | *Epiplatys sexfasciatus* | FJ872061 | *ND2* |
| Cyprinodontiformes |  | Nothobranchiidae | *Epiplatys singa* | AF092358 | *ND2* |
| Cyprinodontiformes |  | Nothobranchiidae | *Episemion callipteron* | DQ981772 | *CYB* |
| Cyprinodontiformes |  | Nothobranchiidae | *Episemion krystallinoron* | DQ981771 | *CYB* |
| Cyprinodontiformes |  | Nothobranchiidae | *Fundulopanchax gardneri* | AF092356 | *ND2* |
| Cyprinodontiformes |  | Nothobranchiidae | *Fundulopanchax sjostedti* | DQ981782 | *CYB* |
| Cyprinodontiformes |  | Nothobranchiidae | *Kathetys bamilekorum* | DQ981778 | *CYB* |
| Cyprinodontiformes |  | Nothobranchiidae | *Kathetys elberti* | DQ981776 | *CYB* |
| Cyprinodontiformes |  | Nothobranchiidae | *Kathetys exiguum* | DQ981777 | *CYB* |
| Cyprinodontiformes |  | Nothobranchiidae | *Nimbapanchax jeanpoli* | FJ872057 | *ND2* |
| Cyprinodontiformes |  | Nothobranchiidae | *Nimbapanchax leucopterygius* | FJ872053 | *ND2* |
| Cyprinodontiformes |  | Nothobranchiidae | *Nimbapanchax melanopterygius* | FJ872052 | *ND2* |
| Cyprinodontiformes |  | Nothobranchiidae | *Nimbapanchax viridis* | FJ872055 | *ND2* |
| Cyprinodontiformes |  | Nothobranchiidae | *Nothobranchius cardinalis* | EF464711 | *CO1* |
| Cyprinodontiformes |  | Nothobranchiidae | *Nothobranchius eggersi* | EF464686 | *CO1* |
| Cyprinodontiformes |  | Nothobranchiidae | *Nothobranchius eggersi* | GU138045 | *ND2* |
| Cyprinodontiformes |  | Nothobranchiidae | *Nothobranchius elongatus* | EU182591 | *ND2* |
| Cyprinodontiformes |  | Nothobranchiidae | *Nothobranchius foerschi* | EF464687 | *CO1* |
| Cyprinodontiformes |  | Nothobranchiidae | *Nothobranchius furzeri* | EF464691 | *CO1* |
| Cyprinodontiformes |  | Nothobranchiidae | *Nothobranchius furzeri* | AF092357 | *ND2* |
| Cyprinodontiformes |  | Nothobranchiidae | *Nothobranchius guentheri* | EF464692 | *CO1* |
| Cyprinodontiformes |  | Nothobranchiidae | *Nothobranchius hengstleri* | EF464709 | *CO1* |
| Cyprinodontiformes |  | Nothobranchiidae | *Nothobranchius hengstleri* | EU401646 | *ND2* |
| Cyprinodontiformes |  | Nothobranchiidae | *Nothobranchius jubbi* | EU401628 | *ND2* |
| Cyprinodontiformes |  | Nothobranchiidae | *Nothobranchius kafuensis* | GU138059 | *ND2* |
| Cyprinodontiformes |  | Nothobranchiidae | *Nothobranchius kilomberoensis* | EF464693 | *CO1* |
| Cyprinodontiformes |  | Nothobranchiidae | *Nothobranchius kirki* | JF444830 | *CO1* |
| Cyprinodontiformes |  | Nothobranchiidae | *Nothobranchius krammeri* | EF464707 | *CO1* |
| Cyprinodontiformes |  | Nothobranchiidae | *Nothobranchius krysanovi* | GU138058 | *ND2* |
| Cyprinodontiformes |  | Nothobranchiidae | *Nothobranchius kuhntae* | EF464695 | *CO1* |
| Cyprinodontiformes |  | Nothobranchiidae | *Nothobranchius lucius* | EU401631 | *ND2* |
| Cyprinodontiformes |  | Nothobranchiidae | *Nothobranchius makondorum* | EU401633 | *ND2* |
| Cyprinodontiformes |  | Nothobranchiidae | *Nothobranchius melanospilus* | EF464712 | *CO1* |
| Cyprinodontiformes |  | Nothobranchiidae | *Nothobranchius melanospilus* | EU401642 | *ND2* |
| Cyprinodontiformes |  | Nothobranchiidae | *Nothobranchius ocellatus* | EU401643 | *ND2* |
| Cyprinodontiformes |  | Nothobranchiidae | *Nothobranchius orthonotus* | EF464696 | *CO1* |
| Cyprinodontiformes |  | Nothobranchiidae | *Nothobranchius orthonotus* | EU401644 | *ND2* |
| Cyprinodontiformes |  | Nothobranchiidae | *Nothobranchius pienaari* | GU138054 | *ND2* |
| Cyprinodontiformes |  | Nothobranchiidae | *Nothobranchius polli* | GU138051 | *ND2* |
| Cyprinodontiformes |  | Nothobranchiidae | *Nothobranchius rachovii* | EF464703 | *CO1* |
| Cyprinodontiformes |  | Nothobranchiidae | *Nothobranchius rachovii* | GU138049 | *ND2* |
| Cyprinodontiformes |  | Nothobranchiidae | *Nothobranchius rubroreticulatus* | GU138047 | *ND2* |
| Cyprinodontiformes |  | Nothobranchiidae | *Pronothobranchius kiyawensis* | EF464705 | *CO1* |
| Cyprinodontiformes |  | Nothobranchiidae | *Scriptaphyosemion geryi* | AF002574 | *CO1* |
| Cyprinodontiformes |  | Nothobranchiidae | *Scriptaphyosemion geryi* | AF092359 | *ND2* |
| Cyprinodontiformes |  | Nothobranchiidae | *Scriptaphyosemion guignardi* | EF455703 | *ND2* |
| Cyprinodontiformes |  | Rivulidae | *Aphyolebias peruensis* | AF002638 | *CO1* |
| Cyprinodontiformes |  | Rivulidae | *Aphyolebias peruensis* | AF092407 | *ND2* |
| Cyprinodontiformes |  | Rivulidae | *Austrofundulus guajira* | AY850649 | *ND2* |
| Cyprinodontiformes |  | Rivulidae | *Austrofundulus leohoignei* | AY850656 | *ND2* |
| Cyprinodontiformes |  | Rivulidae | *Austrofundulus leoni* | AY850651 | *ND2* |
| Cyprinodontiformes |  | Rivulidae | *Austrofundulus limnaeus* | AF002589 | *CO1* |
| Cyprinodontiformes |  | Rivulidae | *Austrofundulus limnaeus* | AY850645 | *ND2* |
| Cyprinodontiformes |  | Rivulidae | *Austrofundulus rupununi* | AY850657 | *ND2* |
| Cyprinodontiformes |  | Rivulidae | *Austrofundulus transilis* | AY850662 | *ND2* |
| Cyprinodontiformes |  | Rivulidae | *Austrolebias charrua* | AY724390 | *CYB* |
| Cyprinodontiformes |  | Rivulidae | *Austrolebias nigripinnis* | AF245013 | *CYB* |
| Cyprinodontiformes |  | Rivulidae | *Austrolebias nigrofasciatus* | AY724407 | *CYB* |
| Cyprinodontiformes |  | Rivulidae | *Austrolebias reicherti* | AY724392 | *CYB* |
| Cyprinodontiformes |  | Rivulidae | *Austrolebias wolterstorffi* | AF245014 | *CYB* |
| Cyprinodontiformes |  | Rivulidae | *Campellolebias dorsimaculatus* | AF092362 | *ND2* |
| Cyprinodontiformes |  | Rivulidae | *Cynolebias adloffi* | AY724372 | *CYB* |
| Cyprinodontiformes |  | Rivulidae | *Cynolebias affinis* | AF002579 | *CO1* |
| Cyprinodontiformes |  | Rivulidae | *Cynolebias affinis* | AF245464 | *CYB* |
| Cyprinodontiformes |  | Rivulidae | *Cynolebias alexandri* | AF245011 | *CYB* |
| Cyprinodontiformes |  | Rivulidae | *Cynolebias alexandri* | AF092369 | *ND2* |
| Cyprinodontiformes |  | Rivulidae | *Cynolebias antenori* | AF002580 | *CO1* |
| Cyprinodontiformes |  | Rivulidae | *Cynolebias bellottii* | AF245007 | *CYB* |
| Cyprinodontiformes |  | Rivulidae | *Cynolebias bellottii* | AF092370 | *ND2* |
| Cyprinodontiformes |  | Rivulidae | *Cynolebias bokermanni* | AF092367 | *ND2* |
| Cyprinodontiformes |  | Rivulidae | *Cynolebias cheradophilus* | AF245467 | *CYB* |
| Cyprinodontiformes |  | Rivulidae | *Cynolebias cinereus* | AF245005 | *CYB* |
| Cyprinodontiformes |  | Rivulidae | *Cynolebias costai* | AF002578 | *CO1* |
| Cyprinodontiformes |  | Rivulidae | *Cynolebias cyaneus* | AF245461 | *CYB* |
| Cyprinodontiformes |  | Rivulidae | *Cynolebias gymnoventris* | AF245463 | *CYB* |
| Cyprinodontiformes |  | Rivulidae | *Cynolebias magnificus* | AF092368 | *ND2* |
| Cyprinodontiformes |  | Rivulidae | *Cynolebias melanoorus* | AY724373 | *CYB* |
| Cyprinodontiformes |  | Rivulidae | *Cynolebias myersi* | AF092366 | *ND2* |
| Cyprinodontiformes |  | Rivulidae | *Cynolebias nioni* | AF245457 | *CYB* |
| Cyprinodontiformes |  | Rivulidae | *Cynolebias vazferreirai* | AF245015 | *CYB* |
| Cyprinodontiformes |  | Rivulidae | *Cynolebias viarius* | AY724386 | *CYB* |
| Cyprinodontiformes |  | Rivulidae | *Cynopoecilus melanotaenia* | AF245465 | *CYB* |
| Cyprinodontiformes |  | Rivulidae | *Cynopoecilus melanotaenia* | AF092363 | *ND2* |
| Cyprinodontiformes |  | Rivulidae | *Gnatholebias hoignei* | EF455704 | *ND2* |
| Cyprinodontiformes |  | Rivulidae | *Gnatholebias zonatus* | AF092419 | *ND2* |
| Cyprinodontiformes |  | Rivulidae | *Gnatholepis anjerensis* | AF537730 | *ND2* |
| Cyprinodontiformes |  | Rivulidae | *Gnatholepis cauerensis* | AF391508 | *ND2* |
| Cyprinodontiformes |  | Rivulidae | *Gnatholepis davaoensis* | AY295339 | *ND2* |
| Cyprinodontiformes |  | Rivulidae | *Gnatholepis knighti* | AF504307 | *ND2* |
| Cyprinodontiformes |  | Rivulidae | *Gnatholepis scapulostigma* | AF391520 | *ND2* |
| Cyprinodontiformes |  | Rivulidae | *Gnatholepis thompsoni* | AF391487 | *ND2* |
| Cyprinodontiformes |  | Rivulidae | *Kryptolebias braziliensis* | AY946276 | *ND2* |
| Cyprinodontiformes |  | Rivulidae | *Kryptolebias marmoratus* | GQ389361 | *ATP6* |
| Cyprinodontiformes |  | Rivulidae | *Kryptolebias marmoratus* | FN544255 | *CO1* |
| Cyprinodontiformes |  | Rivulidae | *Kryptolebias marmoratus* | AY946275 | *ND2* |
| Cyprinodontiformes |  | Rivulidae | *Kryptolebias ocellatus* | GQ389385 | *ATP6* |
| Cyprinodontiformes |  | Rivulidae | *Kryptolebias sepia* | AY946272 | *ND2* |
| Cyprinodontiformes |  | Rivulidae | *Leptolebias aureoguttatus* | AF002581 | *CO1* |
| Cyprinodontiformes |  | Rivulidae | *Leptolebias citrinipinnis* | AF002582 | *CO1* |
| Cyprinodontiformes |  | Rivulidae | *Leptolebias minimus* | AF002583 | *CO1* |
| Cyprinodontiformes |  | Rivulidae | *Llanolebias stellifer* | AF092420 | *ND2* |
| Cyprinodontiformes |  | Rivulidae | *Maratecoara formosa* | AF092411 | *ND2* |
| Cyprinodontiformes |  | Rivulidae | *Maratecoara lacortei* | AF002585 | *CO1* |
| Cyprinodontiformes |  | Rivulidae | *Maratecoara lacortei* | EF585266 | *ND2* |
| Cyprinodontiformes |  | Rivulidae | *Micromoema xiphophorus* | AF092418 | *ND2* |
| Cyprinodontiformes |  | Rivulidae | *Moema piriana* | AF002639 | *CO1* |
| Cyprinodontiformes |  | Rivulidae | *Moema staecki* | AF002640 | *CO1* |
| Cyprinodontiformes |  | Rivulidae | *Moema staecki* | AF092406 | *ND2* |
| Cyprinodontiformes |  | Rivulidae | *Nematolebias whitei* | AF002577 | *CO1* |
| Cyprinodontiformes |  | Rivulidae | *Nematolebias whitei* | AF092403 | *ND2* |
| Cyprinodontiformes |  | Rivulidae | *Neofundulus paraguayensis* | AF002643 | *CO1* |
| Cyprinodontiformes |  | Rivulidae | *Papiliolebias bitteri* | AF002588 | *CO1* |
| Cyprinodontiformes |  | Rivulidae | *Papiliolebias bitteri* | AF092408 | *ND2* |
| Cyprinodontiformes |  | Rivulidae | *Pituna poranga* | AF002586 | *CO1* |
| Cyprinodontiformes |  | Rivulidae | *Pituna poranga* | AF092412 | *ND2* |
| Cyprinodontiformes |  | Rivulidae | *Plesiolebias aruana* | AF002587 | *CO1* |
| Cyprinodontiformes |  | Rivulidae | *Plesiolebias glaucopterus* | AF245468 | *CYB* |
| Cyprinodontiformes |  | Rivulidae | *Pterolebias longipinnis* | AF002595 | *CO1* |
| Cyprinodontiformes |  | Rivulidae | *Pterolebias longipinnis* | AF245462 | *CYB* |
| Cyprinodontiformes |  | Rivulidae | *Pterolebias longipinnis* | AF092415 | *ND2* |
| Cyprinodontiformes |  | Rivulidae | *Pterolebias phasianus* | AF002596 | *CO1* |
| Cyprinodontiformes |  | Rivulidae | *Pterolebias phasianus* | AF092414 | *ND2* |
| Cyprinodontiformes |  | Rivulidae | *Rachovia brevis* | AY850640 | *ND2* |
| Cyprinodontiformes |  | Rivulidae | *Rachovia hummelincki* | AY850642 | *ND2* |
| Cyprinodontiformes |  | Rivulidae | *Rachovia maculipinnis* | AF002590 | *CO1* |
| Cyprinodontiformes |  | Rivulidae | *Rachovia maculipinnis* | AF092417 | *ND2* |
| Cyprinodontiformes |  | Rivulidae | *Rachovia pyropunctata* | AY850641 | *ND2* |
| Cyprinodontiformes |  | Rivulidae | *Renova oscari* | AF002594 | *CO1* |
| Cyprinodontiformes |  | Rivulidae | *Renova oscari* | AF092413 | *ND2* |
| Cyprinodontiformes |  | Rivulidae | *Rivulus agilae* | AF002603 | *CO1* |
| Cyprinodontiformes |  | Rivulidae | *Rivulus agilae* | AF092377 | *ND2* |
| Cyprinodontiformes |  | Rivulidae | *Rivulus amphoreus* | AF002618 | *CO1* |
| Cyprinodontiformes |  | Rivulidae | *Rivulus apiamici* | AF002635 | *CO1* |
| Cyprinodontiformes |  | Rivulidae | *Rivulus birkhani* | AF002625 | *CO1* |
| Cyprinodontiformes |  | Rivulidae | *Rivulus breviceps* | AF092376 | *ND2* |
| Cyprinodontiformes |  | Rivulidae | *Rivulus brunneus* | AF092383 | *ND2* |
| Cyprinodontiformes |  | Rivulidae | *Rivulus caudomarginatus* | AF092361 | *ND2* |
| Cyprinodontiformes |  | Rivulidae | *Rivulus chucunaque* | AF002624 | *CO1* |
| Cyprinodontiformes |  | Rivulidae | *Rivulus compressus* | AY578714 | *ND2* |
| Cyprinodontiformes |  | Rivulidae | *Rivulus cryptocalus* | AF092394 | *ND2* |
| Cyprinodontiformes |  | Rivulidae | *Rivulus cylindraceus* | FN544247 | *CO1* |
| Cyprinodontiformes |  | Rivulidae | *Rivulus cylindraceus* | AF092371 | *ND2* |
| Cyprinodontiformes |  | Rivulidae | *Rivulus deltaphilus* | AF002616 | *CO1* |
| Cyprinodontiformes |  | Rivulidae | *Rivulus deltaphilus* | AF092395 | *ND2* |
| Cyprinodontiformes |  | Rivulidae | *Rivulus duckensis* | AY578711 | *ND2* |
| Cyprinodontiformes |  | Rivulidae | *Rivulus frenatus* | AF002606 | *CO1* |
| Cyprinodontiformes |  | Rivulidae | *Rivulus frenatus* | AF092378 | *ND2* |
| Cyprinodontiformes |  | Rivulidae | *Rivulus frommi* | AF092384 | *ND2* |
| Cyprinodontiformes |  | Rivulidae | *Rivulus fuscolineatus* | AF002631 | *CO1* |
| Cyprinodontiformes |  | Rivulidae | *Rivulus geayi* | AF002604 | *CO1* |
| Cyprinodontiformes |  | Rivulidae | *Rivulus geayi* | AY578712 | *ND2* |
| Cyprinodontiformes |  | Rivulidae | *Rivulus gransabanae* | AF092375 | *ND2* |
| Cyprinodontiformes |  | Rivulidae | *Rivulus haraldsiolii* | AF092379 | *ND2* |
| Cyprinodontiformes |  | Rivulidae | *Rivulus hartii* | AY619630 | *CYB* |
| Cyprinodontiformes |  | Rivulidae | *Rivulus hartii* | AF092393 | *ND2* |
| Cyprinodontiformes |  | Rivulidae | *Rivulus hildebrandi* | AF002621 | *CO1* |
| Cyprinodontiformes |  | Rivulidae | *Rivulus immaculatus* | AF002620 | *CO1* |
| Cyprinodontiformes |  | Rivulidae | *Rivulus insulaepinorum* | FN544252 | *CO1* |
| Cyprinodontiformes |  | Rivulidae | *Rivulus iridescens* | AF092391 | *ND2* |
| Cyprinodontiformes |  | Rivulidae | *Rivulus isthmensis* | AF002630 | *CO1* |
| Cyprinodontiformes |  | Rivulidae | *Rivulus janeiroensis* | AF002632 | *CO1* |
| Cyprinodontiformes |  | Rivulidae | *Rivulus jucundus* | AF002612 | *CO1* |
| Cyprinodontiformes |  | Rivulidae | *Rivulus jucundus* | AF092392 | *ND2* |
| Cyprinodontiformes |  | Rivulidae | *Rivulus luelingi* | AF002633 | *CO1* |
| Cyprinodontiformes |  | Rivulidae | *Rivulus luelingi* | AF092381 | *ND2* |
| Cyprinodontiformes |  | Rivulidae | *Rivulus lyricauda* | AF002610 | *CO1* |
| Cyprinodontiformes |  | Rivulidae | *Rivulus lyricauda* | AY578717 | *ND2* |
| Cyprinodontiformes |  | Rivulidae | *Rivulus magdalenae* | AF002623 | *CO1* |
| Cyprinodontiformes |  | Rivulidae | *Rivulus magdalenae* | AF092382 | *ND2* |
| Cyprinodontiformes |  | Rivulidae | *Rivulus obscurus* | AY578715 | *ND2* |
| Cyprinodontiformes |  | Rivulidae | *Rivulus occellatus* | AF002599 | *CO1* |
| Cyprinodontiformes |  | Rivulidae | *Rivulus ophiomimus* | AF002613 | *CO1* |
| Cyprinodontiformes |  | Rivulidae | *Rivulus ophiomimus* | AF092399 | *ND2* |
| Cyprinodontiformes |  | Rivulidae | *Rivulus pictus* | AF092388 | *ND2* |
| Cyprinodontiformes |  | Rivulidae | *Rivulus punctatus* | AF002636 | *CO1* |
| Cyprinodontiformes |  | Rivulidae | *Rivulus punctatus* | AF092389 | *ND2* |
| Cyprinodontiformes |  | Rivulidae | *Rivulus rectocaudatus* | AF002611 | *CO1* |
| Cyprinodontiformes |  | Rivulidae | *Rivulus rectocaudatus* | AY578716 | *ND2* |
| Cyprinodontiformes |  | Rivulidae | *Rivulus roloffi* | AF002602 | *CO1* |
| Cyprinodontiformes |  | Rivulidae | *Rivulus roloffi* | AF092372 | *ND2* |
| Cyprinodontiformes |  | Rivulidae | *Rivulus rubrolineatus* | AF002614 | *CO1* |
| Cyprinodontiformes |  | Rivulidae | *Rivulus santensis* | AF002634 | *CO1* |
| Cyprinodontiformes |  | Rivulidae | *Rivulus santensis* | AF092380 | *ND2* |
| Cyprinodontiformes |  | Rivulidae | *Rivulus stagnatus* | AF002615 | *CO1* |
| Cyprinodontiformes |  | Rivulidae | *Rivulus stagnatus* | AF092398 | *ND2* |
| Cyprinodontiformes |  | Rivulidae | *Rivulus strigatus* | AF002605 | *CO1* |
| Cyprinodontiformes |  | Rivulidae | *Rivulus tecminae* | AF092374 | *ND2* |
| Cyprinodontiformes |  | Rivulidae | *Rivulus tenuis* | EU751964 | *CO1* |
| Cyprinodontiformes |  | Rivulidae | *Rivulus tenuis* | AF092386 | *ND2* |
| Cyprinodontiformes |  | Rivulidae | *Rivulus uroflammeus* | AF002622 | *CO1* |
| Cyprinodontiformes |  | Rivulidae | *Rivulus urophthalmus* | AY946273 | *ND2* |
| Cyprinodontiformes |  | Rivulidae | *Rivulus violaceus* | AF002637 | *CO1* |
| Cyprinodontiformes |  | Rivulidae | *Rivulus violaceus* | AF092387 | *ND2* |
| Cyprinodontiformes |  | Rivulidae | *Rivulus waimacui* | AF092397 | *ND2* |
| Cyprinodontiformes |  | Rivulidae | *Rivulus weberi* | AF002629 | *CO1* |
| Cyprinodontiformes |  | Rivulidae | *Rivulus weberi* | AF092385 | *ND2* |
| Cyprinodontiformes |  | Rivulidae | *Rivulus xiphidius* | AF002607 | *CO1* |
| Cyprinodontiformes |  | Rivulidae | *Simpsonichthys flagellatus* | HQ833481 | *CO1* |
| Cyprinodontiformes |  | Rivulidae | *Simpsonichthys flavicaudatus* | HQ833492 | *CO1* |
| Cyprinodontiformes |  | Rivulidae | *Simpsonichthys guanambi* | HQ833486 | *CO1* |
| Cyprinodontiformes |  | Rivulidae | *Simpsonichthys janaubensis* | HQ833489 | *CO1* |
| Cyprinodontiformes |  | Rivulidae | *Simpsonichthys mediopapillatus* | HQ833478 | *CO1* |
| Cyprinodontiformes |  | Rivulidae | *Terranatos dolichopterus* | AF002593 | *CO1* |
| Cyprinodontiformes |  | Rivulidae | *Terranatos dolichopterus* | AF092421 | *ND2* |
| Cyprinodontiformes |  | Rivulidae | *Trigonectes aplocheiloides* | AF092400 | *ND2* |
| Cyprinodontiformes |  | Rivulidae | *Trigonectes balzanii* | AF092401 | *ND2* |
| Cyprinodontiformes |  | Rivulidae | *Trigonectes rubromarginatus* | AF002642 | *CO1* |
| Cyprinodontiformes |  | Rivulidae | *Trigonectes rubromarginatus* | AF092402 | *ND2* |
| Cyprinodontiformes |  | Profundulidae | *Profundulus candalarius* | HQ691243 | *CO1* |
| Cyprinodontiformes |  | Profundulidae | *Profundulus candalarius* | HQ691237 | *CYB* |
| Cyprinodontiformes |  | Profundulidae | *Profundulus guatemalensis* | JN028283 | *CO1* |
| Cyprinodontiformes |  | Profundulidae | *Profundulus guatemalensis* | AY155568 | *CYB* |
| Cyprinodontiformes |  | Profundulidae | *Profundulus guatemalensis* | GQ119857 | *rag1* |
| Cyprinodontiformes |  | Profundulidae | *Profundulus labialis* | AY356596 | *CO1* |
| Cyprinodontiformes |  | Profundulidae | *Profundulus labialis* | AY155567 | *CYB* |
| Cyprinodontiformes |  | Profundulidae | *Profundulus punctatus* | HQ691246 | *CO1* |
| Cyprinodontiformes |  | Profundulidae | *Profundulus punctatus* | AY155566 | *CYB* |
| Cyprinodontiformes |  | Goodeidae | *Allodontichthys hubbsi* | AY356553 | *CO1* |
| Cyprinodontiformes |  | Goodeidae | *Allodontichthys hubbsi* | AF510836 | *CYB* |
| Cyprinodontiformes |  | Goodeidae | *Allodontichthys polylepis* | AY356555 | *CO1* |
| Cyprinodontiformes |  | Goodeidae | *Allodontichthys polylepis* | AF510839 | *CYB* |
| Cyprinodontiformes |  | Goodeidae | *Allodontichthys tamazulae* | AY356556 | *CO1* |
| Cyprinodontiformes |  | Goodeidae | *Allodontichthys tamazulae* | AF510838 | *CYB* |
| Cyprinodontiformes |  | Goodeidae | *Allodontichthys zonistius* | AY356558 | *CO1* |
| Cyprinodontiformes |  | Goodeidae | *Allodontichthys zonistius* | AF510840 | *CYB* |
| Cyprinodontiformes |  | Goodeidae | *Alloophorus robustus* | AY356561 | *CO1* |
| Cyprinodontiformes |  | Goodeidae | *Alloophorus robustus* | AF510810 | *CYB* |
| Cyprinodontiformes |  | Goodeidae | *Allotoca catarinae* | AY356562 | *CO1* |
| Cyprinodontiformes |  | Goodeidae | *Allotoca catarinae* | AF510795 | *CYB* |
| Cyprinodontiformes |  | Goodeidae | *Allotoca diazi* | AY356554 | *CO1* |
| Cyprinodontiformes |  | Goodeidae | *Allotoca diazi* | AF510790 | *CYB* |
| Cyprinodontiformes |  | Goodeidae | *Allotoca dugesii* | AY356557 | *CO1* |
| Cyprinodontiformes |  | Goodeidae | *Allotoca dugesii* | AF510801 | *CYB* |
| Cyprinodontiformes |  | Goodeidae | *Allotoca goslinei* | AY356559 | *CO1* |
| Cyprinodontiformes |  | Goodeidae | *Allotoca goslinei* | AF510800 | *CYB* |
| Cyprinodontiformes |  | Goodeidae | *Allotoca maculata* | AY356560 | *CO1* |
| Cyprinodontiformes |  | Goodeidae | *Allotoca maculata* | AF510797 | *CYB* |
| Cyprinodontiformes |  | Goodeidae | *Allotoca meeki* | AF510791 | *CYB* |
| Cyprinodontiformes |  | Goodeidae | *Allotoca regalis* | AY356563 | *CO1* |
| Cyprinodontiformes |  | Goodeidae | *Allotoca regalis* | AF510799 | *CYB* |
| Cyprinodontiformes |  | Goodeidae | *Allotoca zacapuensis* | AF510789 | *CYB* |
| Cyprinodontiformes |  | Goodeidae | *Alphestes afer* | AY313996 | *CYB* |
| Cyprinodontiformes |  | Goodeidae | *Alphestes immaculatus* | AY314002 | *CYB* |
| Cyprinodontiformes |  | Goodeidae | *Alphestes multiguttatus* | AY313995 | *CYB* |
| Cyprinodontiformes |  | Goodeidae | *Ameca splendens* | AY356564 | *CO1* |
| Cyprinodontiformes |  | Goodeidae | *Ameca splendens* | AF510818 | *CYB* |
| Cyprinodontiformes |  | Goodeidae | *Ataeniobius toweri* | AY356566 | *CO1* |
| Cyprinodontiformes |  | Goodeidae | *Ataeniobius toweri* | AF510779 | *CYB* |
| Cyprinodontiformes |  | Goodeidae | *Chapalichthys encaustus* | AY356570 | *CO1* |
| Cyprinodontiformes |  | Goodeidae | *Chapalichthys encaustus* | AF510816 | *CYB* |
| Cyprinodontiformes |  | Goodeidae | *Chapalichthys pardalis* | AY356567 | *CO1* |
| Cyprinodontiformes |  | Goodeidae | *Characodon audax* | AY356568 | *CO1* |
| Cyprinodontiformes |  | Goodeidae | *Characodon audax* | AF510822 | *CYB* |
| Cyprinodontiformes |  | Goodeidae | *Characodon lateralis* | AY356569 | *CO1* |
| Cyprinodontiformes |  | Goodeidae | *Characodon lateralis* | AF510821 | *CYB* |
| Cyprinodontiformes |  | Goodeidae | *Crenichthys baileyi* | AY356571 | *CO1* |
| Cyprinodontiformes |  | Goodeidae | *Crenichthys baileyi* | AF510819 | *CYB* |
| Cyprinodontiformes |  | Goodeidae | *Crenichthys baileyi* | FJ185089 | *rag1* |
| Cyprinodontiformes |  | Goodeidae | *Empetrichthys latos* | AY356573 | *CO1* |
| Cyprinodontiformes |  | Goodeidae | *Girardinichthys multiradiatus* | AY356576 | *CO1* |
| Cyprinodontiformes |  | Goodeidae | *Girardinichthys multiradiatus* | AF510785 | *CYB* |
| Cyprinodontiformes |  | Goodeidae | *Girardinichthys viviparus* | AY356575 | *CO1* |
| Cyprinodontiformes |  | Goodeidae | *Girardinichthys viviparus* | AF510788 | *CYB* |
| Cyprinodontiformes |  | Goodeidae | *Goodea atripinnis* | AY356577 | *CO1* |
| Cyprinodontiformes |  | Goodeidae | *Goodea atripinnis* | AF510773 | *CYB* |
| Cyprinodontiformes |  | Goodeidae | *Goodea gracilis* | AF510769 | *CYB* |
| Cyprinodontiformes |  | Goodeidae | *Hubbsina turneri* | AY356578 | *CO1* |
| Cyprinodontiformes |  | Goodeidae | *Hubbsina turneri* | AF510841 | *CYB* |
| Cyprinodontiformes |  | Goodeidae | *Ilyodon amecae* | AF510826 | *CYB* |
| Cyprinodontiformes |  | Goodeidae | *Ilyodon furcidens* | AY356579 | *CO1* |
| Cyprinodontiformes |  | Goodeidae | *Ilyodon furcidens* | AF510831 | *CYB* |
| Cyprinodontiformes |  | Goodeidae | *Ilyodon whitei* | AY356580 | *CO1* |
| Cyprinodontiformes |  | Goodeidae | *Ilyodon whitei* | AF510833 | *CYB* |
| Cyprinodontiformes |  | Goodeidae | *Ilyodon xantusi* | AF510829 | *CYB* |
| Cyprinodontiformes |  | Goodeidae | *Neotoca bilineata* | AF510749 | *CYB* |
| Cyprinodontiformes |  | Goodeidae | *Skiffia bilineatus* | AY356583 | *CO1* |
| Cyprinodontiformes |  | Goodeidae | *Skiffia francesae* | AY356582 | *CO1* |
| Cyprinodontiformes |  | Goodeidae | *Skiffia francesae* | AF510845 | *CYB* |
| Cyprinodontiformes |  | Goodeidae | *Skiffia lermae* | AY356584 | *CO1* |
| Cyprinodontiformes |  | Goodeidae | *Skiffia lermae* | AF510781 | *CYB* |
| Cyprinodontiformes |  | Goodeidae | *Skiffia multipunctata* | AY356585 | *CO1* |
| Cyprinodontiformes |  | Goodeidae | *Skiffia multipunctata* | AF510842 | *CYB* |
| Cyprinodontiformes |  | Goodeidae | *Xenoophorus captiva* | AY356586 | *CO1* |
| Cyprinodontiformes |  | Goodeidae | *Xenoophorus captiva* | AF510758 | *CYB* |
| Cyprinodontiformes |  | Goodeidae | *Xenotaenia resolanae* | AY356590 | *CO1* |
| Cyprinodontiformes |  | Goodeidae | *Xenotaenia resolanae* | AF510825 | *CYB* |
| Cyprinodontiformes |  | Goodeidae | *Xenotoca eiseni* | AY356587 | *CO1* |
| Cyprinodontiformes |  | Goodeidae | *Xenotoca eiseni* | AF510765 | *CYB* |
| Cyprinodontiformes |  | Goodeidae | *Xenotoca melanosoma* | AY356588 | *CO1* |
| Cyprinodontiformes |  | Goodeidae | *Xenotoca melanosoma* | AF510761 | *CYB* |
| Cyprinodontiformes |  | Goodeidae | *Xenotoca variatus* | AY356589 | *CO1* |
| Cyprinodontiformes |  | Goodeidae | *Xenotoca variatus* | AF510805 | *CYB* |
| Cyprinodontiformes |  | Goodeidae | *Zoogoneticus quitzeoensis* | AY356592 | *CO1* |
| Cyprinodontiformes |  | Goodeidae | *Zoogoneticus quitzeoensis* | AF510753 | *CYB* |
| Cyprinodontiformes |  | Goodeidae | *Zoogoneticus tequila* | AY356591 | *CO1* |
| Cyprinodontiformes |  | Goodeidae | *Zoogoneticus tequila* | AF510757 | *CYB* |
| Cyprinodontiformes |  | Fundulidae | *Adinia xenica* | JN024714 | *CO1* |
| Cyprinodontiformes |  | Fundulidae | *Adinia xenica* | GQ119680 | *CYB* |
| Cyprinodontiformes |  | Fundulidae | *Adinia xenica* | GQ119858 | *rag1* |
| Cyprinodontiformes |  | Fundulidae | *Fundulus bermudae* | GQ119682 | *CYB* |
| Cyprinodontiformes |  | Fundulidae | *Fundulus bermudae* | GQ119859 | *rag1* |
| Cyprinodontiformes |  | Fundulidae | *Fundulus blairae* | JN026620 | *CO1* |
| Cyprinodontiformes |  | Fundulidae | *Fundulus blairae* | GQ119685 | *CYB* |
| Cyprinodontiformes |  | Fundulidae | *Fundulus blairae* | GQ119860 | *rag1* |
| Cyprinodontiformes |  | Fundulidae | *Fundulus catenatus* | JN026622 | *CO1* |
| Cyprinodontiformes |  | Fundulidae | *Fundulus catenatus* | GQ119689 | *CYB* |
| Cyprinodontiformes |  | Fundulidae | *Fundulus catenatus* | GQ119865 | *rag1* |
| Cyprinodontiformes |  | Fundulidae | *Fundulus chrysotus* | JN026633 | *CO1* |
| Cyprinodontiformes |  | Fundulidae | *Fundulus chrysotus* | GQ119696 | *CYB* |
| Cyprinodontiformes |  | Fundulidae | *Fundulus chrysotus* | GQ119869 | *rag1* |
| Cyprinodontiformes |  | Fundulidae | *Fundulus cingulatus* | GQ119699 | *CYB* |
| Cyprinodontiformes |  | Fundulidae | *Fundulus confluentus* | GQ119700 | *CYB* |
| Cyprinodontiformes |  | Fundulidae | *Fundulus confluentus* | GQ119873 | *rag1* |
| Cyprinodontiformes |  | Fundulidae | *Fundulus diaphanus* | EU524626 | *CO1* |
| Cyprinodontiformes |  | Fundulidae | *Fundulus diaphanus* | GQ119704 | *CYB* |
| Cyprinodontiformes |  | Fundulidae | *Fundulus diaphanus* | GQ119877 | *rag1* |
| Cyprinodontiformes |  | Fundulidae | *Fundulus dispar* | JN026644 | *CO1* |
| Cyprinodontiformes |  | Fundulidae | *Fundulus dispar* | GQ119708 | *CYB* |
| Cyprinodontiformes |  | Fundulidae | *Fundulus dispar* | GQ119880 | *rag1* |
| Cyprinodontiformes |  | Fundulidae | *Fundulus escambiae* | GQ119709 | *CYB* |
| Cyprinodontiformes |  | Fundulidae | *Fundulus escambiae* | GQ119882 | *rag1* |
| Cyprinodontiformes |  | Fundulidae | *Fundulus euryzonus* | JN026647 | *CO1* |
| Cyprinodontiformes |  | Fundulidae | *Fundulus euryzonus* | GQ119712 | *CYB* |
| Cyprinodontiformes |  | Fundulidae | *Fundulus grandis* | FN545585 | *CO1* |
| Cyprinodontiformes |  | Fundulidae | *Fundulus grandis* | GQ119716 | *CYB* |
| Cyprinodontiformes |  | Fundulidae | *Fundulus grandis* | GQ119884 | *rag1* |
| Cyprinodontiformes |  | Fundulidae | *Fundulus heteroclitus* | EU524627 | *CO1* |
| Cyprinodontiformes |  | Fundulidae | *Fundulus heteroclitus* | GQ119718 | *CYB* |
| Cyprinodontiformes |  | Fundulidae | *Fundulus heteroclitus* | GQ119890 | *rag1* |
| Cyprinodontiformes |  | Fundulidae | *Fundulus jenkinsi* | GQ119721 | *CYB* |
| Cyprinodontiformes |  | Fundulidae | *Fundulus julisia* | GQ119722 | *CYB* |
| Cyprinodontiformes |  | Fundulidae | *Fundulus julisia* | GQ119891 | *rag1* |
| Cyprinodontiformes |  | Fundulidae | *Fundulus kansae* | JN026650 | *CO1* |
| Cyprinodontiformes |  | Fundulidae | *Fundulus kansae* | GQ119725 | *CYB* |
| Cyprinodontiformes |  | Fundulidae | *Fundulus kansae* | GQ119893 | *rag1* |
| Cyprinodontiformes |  | Fundulidae | *Fundulus lima* | GQ119731 | *CYB* |
| Cyprinodontiformes |  | Fundulidae | *Fundulus lima* | GQ119899 | *rag1* |
| Cyprinodontiformes |  | Fundulidae | *Fundulus lineolatus* | JN026657 | *CO1* |
| Cyprinodontiformes |  | Fundulidae | *Fundulus lineolatus* | GQ119727 | *CYB* |
| Cyprinodontiformes |  | Fundulidae | *Fundulus lineolatus* | GQ119895 | *rag1* |
| Cyprinodontiformes |  | Fundulidae | *Fundulus luciae* | GQ119732 | *CYB* |
| Cyprinodontiformes |  | Fundulidae | *Fundulus luciae* | GQ119900 | *rag1* |
| Cyprinodontiformes |  | Fundulidae | *Fundulus majalis* | GQ119733 | *CYB* |
| Cyprinodontiformes |  | Fundulidae | *Fundulus majalis* | GQ119903 | *rag1* |
| Cyprinodontiformes |  | Fundulidae | *Fundulus notatus* | JN026663 | *CO1* |
| Cyprinodontiformes |  | Fundulidae | *Fundulus notatus* | GQ119740 | *CYB* |
| Cyprinodontiformes |  | Fundulidae | *Fundulus notatus* | GQ119906 | *rag1* |
| Cyprinodontiformes |  | Fundulidae | *Fundulus nottii* | GQ119737 | *CYB* |
| Cyprinodontiformes |  | Fundulidae | *Fundulus nottii* | GQ119904 | *rag1* |
| Cyprinodontiformes |  | Fundulidae | *Fundulus olivaceus* | JN026666 | *CO1* |
| Cyprinodontiformes |  | Fundulidae | *Fundulus olivaceus* | GQ119745 | *CYB* |
| Cyprinodontiformes |  | Fundulidae | *Fundulus olivaceus* | GQ119913 | *rag1* |
| Cyprinodontiformes |  | Fundulidae | *Fundulus parvipinnis* | GU440324 | *CO1* |
| Cyprinodontiformes |  | Fundulidae | *Fundulus parvipinnis* | GQ119746 | *CYB* |
| Cyprinodontiformes |  | Fundulidae | *Fundulus parvipinnis* | GQ119914 | *rag1* |
| Cyprinodontiformes |  | Fundulidae | *Fundulus pulvereus* | GQ119749 | *CYB* |
| Cyprinodontiformes |  | Fundulidae | *Fundulus pulvereus* | GQ119917 | *rag1* |
| Cyprinodontiformes |  | Fundulidae | *Fundulus rathbuni* | GQ119750 | *CYB* |
| Cyprinodontiformes |  | Fundulidae | *Fundulus rathbuni* | GQ119918 | *rag1* |
| Cyprinodontiformes |  | Fundulidae | *Fundulus relictus* | GQ119752 | *CYB* |
| Cyprinodontiformes |  | Fundulidae | *Fundulus rubrifrons* | GQ119755 | *CYB* |
| Cyprinodontiformes |  | Fundulidae | *Fundulus rubrifrons* | GQ119920 | *rag1* |
| Cyprinodontiformes |  | Fundulidae | *Fundulus sciadicus* | JN026676 | *CO1* |
| Cyprinodontiformes |  | Fundulidae | *Fundulus sciadicus* | GQ119756 | *CYB* |
| Cyprinodontiformes |  | Fundulidae | *Fundulus sciadicus* | GQ119922 | *rag1* |
| Cyprinodontiformes |  | Fundulidae | *Fundulus seminolis* | GQ119762 | *CYB* |
| Cyprinodontiformes |  | Fundulidae | *Fundulus seminolis* | GQ119927 | *rag1* |
| Cyprinodontiformes |  | Fundulidae | *Fundulus similis* | JN026681 | *CO1* |
| Cyprinodontiformes |  | Fundulidae | *Fundulus similis* | GQ119759 | *CYB* |
| Cyprinodontiformes |  | Fundulidae | *Fundulus similis* | GQ119925 | *rag1* |
| Cyprinodontiformes |  | Fundulidae | *Fundulus stellifer* | JN026686 | *CO1* |
| Cyprinodontiformes |  | Fundulidae | *Fundulus stellifer* | GQ119763 | *CYB* |
| Cyprinodontiformes |  | Fundulidae | *Fundulus stellifer* | GQ119929 | *rag1* |
| Cyprinodontiformes |  | Fundulidae | *Fundulus waccamensis* | JN026688 | *CO1* |
| Cyprinodontiformes |  | Fundulidae | *Fundulus zebrinus* | JN026692 | *CO1* |
| Cyprinodontiformes |  | Fundulidae | *Fundulus zebrinus* | GQ119766 | *CYB* |
| Cyprinodontiformes |  | Fundulidae | *Fundulus zebrinus* | GQ119930 | *rag1* |
| Cyprinodontiformes |  | Fundulidae | *Leptolucania ommata* | JN027058 | *CO1* |
| Cyprinodontiformes |  | Fundulidae | *Lucania goodei* | GQ119768 | *CYB* |
| Cyprinodontiformes |  | Fundulidae | *Lucania goodei* | GQ119933 | *rag1* |
| Cyprinodontiformes |  | Fundulidae | *Lucania parva* | GQ119769 | *CYB* |
| Cyprinodontiformes |  | Fundulidae | *Lucania parva* | GQ119934 | *rag1* |
| Cyprinodontiformes |  | Valenciidae | *Valencia hispanica* | AF449339 | *ND1* |
| Cyprinodontiformes |  | Valenciidae | *Valencia hispanica* | AF449339 | *ND2* |
| Cyprinodontiformes |  | Valenciidae | *Valencia letourneuxi* | AF449336 | *ND1* |
| Cyprinodontiformes |  | Valenciidae | *Valencia letourneuxi* | AF449336 | *ND2* |
| Cyprinodontiformes |  | Cyprinodontidae | *Aphanius anatoliae* | AY356565 | *CO1* |
| Cyprinodontiformes |  | Cyprinodontidae | *Aphanius anatoliae anatoliae* | AF451631 | *ND1* |
| Cyprinodontiformes |  | Cyprinodontidae | *Aphanius anatoliae anatoliae* | AF451631 | *ND2* |
| Cyprinodontiformes |  | Cyprinodontidae | *Aphanius anatoliae splendens* | AF451623 | *ND1* |
| Cyprinodontiformes |  | Cyprinodontidae | *Aphanius anatoliae splendens* | AF451623 | *ND2* |
| Cyprinodontiformes |  | Cyprinodontidae | *Aphanius anatoliae sureyanus* | AF449288 | *ND1* |
| Cyprinodontiformes |  | Cyprinodontidae | *Aphanius anatoliae sureyanus* | AF451624 | *ND2* |
| Cyprinodontiformes |  | Cyprinodontidae | *Aphanius anatoliae transgrediens* | AF451627 | *ND1* |
| Cyprinodontiformes |  | Cyprinodontidae | *Aphanius anatoliae transgrediens* | AF451627 | *ND2* |
| Cyprinodontiformes |  | Cyprinodontidae | *Aphanius apodus* | AF449323 | *ND1* |
| Cyprinodontiformes |  | Cyprinodontidae | *Aphanius apodus* | AF449323 | *ND2* |
| Cyprinodontiformes |  | Cyprinodontidae | *Aphanius asquamatus* | AF449306 | *ND1* |
| Cyprinodontiformes |  | Cyprinodontidae | *Aphanius asquamatus* | AF449306 | *ND2* |
| Cyprinodontiformes |  | Cyprinodontidae | *Aphanius danfordii* | AF449299 | *ND1* |
| Cyprinodontiformes |  | Cyprinodontidae | *Aphanius danfordii* | AF451655 | *ND2* |
| Cyprinodontiformes |  | Cyprinodontidae | *Aphanius dispar dispar* | AF449332 | *ND1* |
| Cyprinodontiformes |  | Cyprinodontidae | *Aphanius dispar dispar* | AF449332 | *ND2* |
| Cyprinodontiformes |  | Cyprinodontidae | *Aphanius dispar richardsoni* | AF449329 | *ND1* |
| Cyprinodontiformes |  | Cyprinodontidae | *Aphanius dispar richardsoni* | AF449329 | *ND2* |
| Cyprinodontiformes |  | Cyprinodontidae | *Aphanius fasciatus* | AF299273 | *CYB* |
| Cyprinodontiformes |  | Cyprinodontidae | *Aphanius fasciatus* | AF449313 | *ND1* |
| Cyprinodontiformes |  | Cyprinodontidae | *Aphanius fasciatus* | AF449313 | *ND2* |
| Cyprinodontiformes |  | Cyprinodontidae | *Aphanius ginaonis* | AF449335 | *ND1* |
| Cyprinodontiformes |  | Cyprinodontidae | *Aphanius ginaonis* | AF449335 | *ND2* |
| Cyprinodontiformes |  | Cyprinodontidae | *Aphanius iberus* | AF299281 | *CYB* |
| Cyprinodontiformes |  | Cyprinodontidae | *Aphanius iberus* | AF449316 | *ND1* |
| Cyprinodontiformes |  | Cyprinodontidae | *Aphanius iberus* | AF449316 | *ND2* |
| Cyprinodontiformes |  | Cyprinodontidae | *Aphanius isfahani* | AY593488 | *ND1* |
| Cyprinodontiformes |  | Cyprinodontidae | *Aphanius isfahani* | AY593488 | *ND2* |
| Cyprinodontiformes |  | Cyprinodontidae | *Aphanius mento* | AF449327 | *ND1* |
| Cyprinodontiformes |  | Cyprinodontidae | *Aphanius mento* | AF449327 | *ND2* |
| Cyprinodontiformes |  | Cyprinodontidae | *Aphanius persicus* | AY593484 | *ND1* |
| Cyprinodontiformes |  | Cyprinodontidae | *Aphanius persicus* | AY593484 | *ND2* |
| Cyprinodontiformes |  | Cyprinodontidae | *Aphanius saourensis* | DQ367527 | *CYB* |
| Cyprinodontiformes |  | Cyprinodontidae | *Aphanius sirhani* | AF449328 | *ND1* |
| Cyprinodontiformes |  | Cyprinodontidae | *Aphanius sirhani* | AF449328 | *ND2* |
| Cyprinodontiformes |  | Cyprinodontidae | *Aphanius sophiae* | AY593481 | *ND1* |
| Cyprinodontiformes |  | Cyprinodontidae | *Aphanius sophiae* | AY593481 | *ND2* |
| Cyprinodontiformes |  | Cyprinodontidae | *Aphanius villwocki* | AF451650 | *ND1* |
| Cyprinodontiformes |  | Cyprinodontidae | *Aphanius villwocki* | AF451650 | *ND2* |
| Cyprinodontiformes |  | Cyprinodontidae | *Aphanius vladykovi* | DQ367526 | *CYB* |
| Cyprinodontiformes |  | Cyprinodontidae | *Aphanius vladykovi* | AY593487 | *ND1* |
| Cyprinodontiformes |  | Cyprinodontidae | *Aphanius vladykovi* | AY593487 | *ND2* |
| Cyprinodontiformes |  | Cyprinodontidae | *Cualac tessellatus* | AY902051 | *CYB* |
| Cyprinodontiformes |  | Cyprinodontidae | *Cualac tessellatus* | AY902109 | *ND2* |
| Cyprinodontiformes |  | Cyprinodontidae | *Cubanichthys cubensis* | FN544256 | *CO1* |
| Cyprinodontiformes |  | Cyprinodontidae | *Cubanichthys pengelleyi* | AY356593 | *CO1* |
| Cyprinodontiformes |  | Cyprinodontidae | *Cyprinodon albivelis* | AY902106 | *CYB* |
| Cyprinodontiformes |  | Cyprinodontidae | *Cyprinodon albivelis* | AY902164 | *ND2* |
| Cyprinodontiformes |  | Cyprinodontidae | *Cyprinodon alvarezi* | AY902081 | *CYB* |
| Cyprinodontiformes |  | Cyprinodontidae | *Cyprinodon alvarezi* | AY902139 | *ND2* |
| Cyprinodontiformes |  | Cyprinodontidae | *Cyprinodon artifrons* | EU751778 | *CO1* |
| Cyprinodontiformes |  | Cyprinodontidae | *Cyprinodon artifrons* | AY902054 | *CYB* |
| Cyprinodontiformes |  | Cyprinodontidae | *Cyprinodon artifrons* | AY902111 | *ND2* |
| Cyprinodontiformes |  | Cyprinodontidae | *Cyprinodon atrorus* | AY902099 | *CYB* |
| Cyprinodontiformes |  | Cyprinodontidae | *Cyprinodon atrorus* | AY902157 | *ND2* |
| Cyprinodontiformes |  | Cyprinodontidae | *Cyprinodon bifasciatus* | AY902097 | *CYB* |
| Cyprinodontiformes |  | Cyprinodontidae | *Cyprinodon bifasciatus* | AY902156 | *ND2* |
| Cyprinodontiformes |  | Cyprinodontidae | *Cyprinodon bondi* | AY902066 | *CYB* |
| Cyprinodontiformes |  | Cyprinodontidae | *Cyprinodon bondi* | DQ218098 | *ND2* |
| Cyprinodontiformes |  | Cyprinodontidae | *Cyprinodon bovinus* | AY902074 | *CYB* |
| Cyprinodontiformes |  | Cyprinodontidae | *Cyprinodon bovinus* | AY902133 | *ND2* |
| Cyprinodontiformes |  | Cyprinodontidae | *Cyprinodon dearborni* | AY902064 | *CYB* |
| Cyprinodontiformes |  | Cyprinodontidae | *Cyprinodon dearborni* | AY902122 | *ND2* |
| Cyprinodontiformes |  | Cyprinodontidae | *Cyprinodon diabolis* | AF028309 | *ND2* |
| Cyprinodontiformes |  | Cyprinodontidae | *Cyprinodon elegans* | AY902076 | *CYB* |
| Cyprinodontiformes |  | Cyprinodontidae | *Cyprinodon elegans* | AY902134 | *ND2* |
| Cyprinodontiformes |  | Cyprinodontidae | *Cyprinodon eximius* | AY902085 | *CYB* |
| Cyprinodontiformes |  | Cyprinodontidae | *Cyprinodon eximius* | AY902144 | *ND2* |
| Cyprinodontiformes |  | Cyprinodontidae | *Cyprinodon fontinalis* | AY902102 | *CYB* |
| Cyprinodontiformes |  | Cyprinodontidae | *Cyprinodon fontinalis* | AF028310 | *ND2* |
| Cyprinodontiformes |  | Cyprinodontidae | *Cyprinodon higuey* | DQ218122 | *ND2* |
| Cyprinodontiformes |  | Cyprinodontidae | *Cyprinodon macrolepis* | AY902082 | *CYB* |
| Cyprinodontiformes |  | Cyprinodontidae | *Cyprinodon macrolepis* | AY902140 | *ND2* |
| Cyprinodontiformes |  | Cyprinodontidae | *Cyprinodon macularius* | AY902103 | *CYB* |
| Cyprinodontiformes |  | Cyprinodontidae | *Cyprinodon macularius* | AY902161 | *ND2* |
| Cyprinodontiformes |  | Cyprinodontidae | *Cyprinodon macularius eremus* | AF198984 | *ND2* |
| Cyprinodontiformes |  | Cyprinodontidae | *Cyprinodon macularius macularius* | AF198971 | *ND2* |
| Cyprinodontiformes |  | Cyprinodontidae | *Cyprinodon meeki* | AY902094 | *CYB* |
| Cyprinodontiformes |  | Cyprinodontidae | *Cyprinodon meeki* | AY902152 | *ND2* |
| Cyprinodontiformes |  | Cyprinodontidae | *Cyprinodon nazas* | AY902093 | *CYB* |
| Cyprinodontiformes |  | Cyprinodontidae | *Cyprinodon nazas* | AY902148 | *ND2* |
| Cyprinodontiformes |  | Cyprinodontidae | *Cyprinodon nevadensis* | AY902100 | *CYB* |
| Cyprinodontiformes |  | Cyprinodontidae | *Cyprinodon nevadensis* | AF028302 | *ND2* |
| Cyprinodontiformes |  | Cyprinodontidae | *Cyprinodon nevadensis nevadensis* | AF028301 | *ND2* |
| Cyprinodontiformes |  | Cyprinodontidae | *Cyprinodon nichollsi* | DQ218114 | *ND2* |
| Cyprinodontiformes |  | Cyprinodontidae | *Cyprinodon pachycephalus* | AY902088 | *CYB* |
| Cyprinodontiformes |  | Cyprinodontidae | *Cyprinodon pachycephalus* | AY902146 | *ND2* |
| Cyprinodontiformes |  | Cyprinodontidae | *Cyprinodon pecosensis* | AY902072 | *CYB* |
| Cyprinodontiformes |  | Cyprinodontidae | *Cyprinodon pecosensis* | AY902130 | *ND2* |
| Cyprinodontiformes |  | Cyprinodontidae | *Cyprinodon pisteri* | AY902104 | *CYB* |
| Cyprinodontiformes |  | Cyprinodontidae | *Cyprinodon pisteri* | AY902163 | *ND2* |
| Cyprinodontiformes |  | Cyprinodontidae | *Cyprinodon radiosus* | AY902107 | *CYB* |
| Cyprinodontiformes |  | Cyprinodontidae | *Cyprinodon radiosus* | AF028311 | *ND2* |
| Cyprinodontiformes |  | Cyprinodontidae | *Cyprinodon rubrofluviatilis* | AY902068 | *CYB* |
| Cyprinodontiformes |  | Cyprinodontidae | *Cyprinodon rubrofluviatilis* | AY902127 | *ND2* |
| Cyprinodontiformes |  | Cyprinodontidae | *Cyprinodon salinus* | AF028300 | *ND2* |
| Cyprinodontiformes |  | Cyprinodontidae | *Cyprinodon tularosa* | AY902065 | *CYB* |
| Cyprinodontiformes |  | Cyprinodontidae | *Cyprinodon tularosa* | AY902123 | *ND2* |
| Cyprinodontiformes |  | Cyprinodontidae | *Cyprinodon variegatus* | JN025275 | *CO1* |
| Cyprinodontiformes |  | Cyprinodontidae | *Cyprinodon variegatus* | AY902060 | *CYB* |
| Cyprinodontiformes |  | Cyprinodontidae | *Cyprinodon variegatus* | AF449344 | *ND1* |
| Cyprinodontiformes |  | Cyprinodontidae | *Cyprinodon variegatus* | AY902116 | *ND2* |
| Cyprinodontiformes |  | Cyprinodontidae | *Cyprinodon variegatus riverendi* | DQ218124 | *ND2* |
| Cyprinodontiformes |  | Cyprinodontidae | *Cyprinodon veronicae* | AY902079 | *CYB* |
| Cyprinodontiformes |  | Cyprinodontidae | *Cyprinodon veronicae* | AY902137 | *ND2* |
| Cyprinodontiformes |  | Cyprinodontidae | *Floridichthys carpio* | JN026618 | *CO1* |
| Cyprinodontiformes |  | Cyprinodontidae | *Floridichthys carpio* | AF449345 | *ND1* |
| Cyprinodontiformes |  | Cyprinodontidae | *Floridichthys carpio* | AF449345 | *ND2* |
| Cyprinodontiformes |  | Cyprinodontidae | *Floridichthys polyommus* | GU225275 | *CO1* |
| Cyprinodontiformes |  | Cyprinodontidae | *Garmanella pulchra* | EU751820 | *CO1* |
| Cyprinodontiformes |  | Cyprinodontidae | *Jordanella floridae* | JN026923 | *CO1* |
| Cyprinodontiformes |  | Cyprinodontidae | *Jordanella floridae* | AY902050 | *CYB* |
| Cyprinodontiformes |  | Cyprinodontidae | *Jordanella floridae* | AF449343 | *ND1* |
| Cyprinodontiformes |  | Cyprinodontidae | *Jordanella floridae* | AY902108 | *ND2* |
| Cyprinodontiformes |  | Cyprinodontidae | *Megupsilon aporus* | AY902052 | *CYB* |
| Cyprinodontiformes |  | Cyprinodontidae | *Megupsilon aporus* | AY902110 | *ND2* |
| Cyprinodontiformes |  | Cyprinodontidae | *Orestias agassizii* | AF449346 | *ND1* |
| Cyprinodontiformes |  | Cyprinodontidae | *Orestias agassizii* | AF449346 | *ND2* |
| Cyprinodontiformes |  | Cyprinodontidae | *Orestias gilsoni* | AF449347 | *ND1* |
| Cyprinodontiformes |  | Cyprinodontidae | *Orestias gilsoni* | AF449347 | *ND2* |
| Cyprinodontiformes |  | Cyprinodontidae | *Orestias imarpe* | AF449348 | *ND1* |
| Cyprinodontiformes |  | Cyprinodontidae | *Orestias imarpe* | AF449348 | *ND2* |
| Cyprinodontiformes |  | Cyprinodontidae | *Orestias silustani* | AY155565 | *CYB* |
| Cyprinodontiformes |  | Anablepidae | *Anableps anableps* | EF017508 | *CYB* |
| Cyprinodontiformes |  | Anablepidae | *Anableps anableps* | EF017558 | *ND1* |
| Cyprinodontiformes |  | Anablepidae | *Anableps anableps* | EF017558 | *ND2* |
| Cyprinodontiformes |  | Anablepidae | *Anableps anableps* | EU637935 | *rhod* |
| Cyprinodontiformes |  | Anablepidae | *Jenynsia lineata* | EF017509 | *CYB* |
| Cyprinodontiformes |  | Anablepidae | *Jenynsia lineata* | EF017559 | *ND1* |
| Cyprinodontiformes |  | Anablepidae | *Jenynsia lineata* | EF017559 | *ND2* |
| Cyprinodontiformes |  | Anablepidae | *Oxyzygonectes dovii* | AY356581 | *CO1* |
| Cyprinodontiformes |  | Anablepidae | *Oxyzygonectes dovii* | EF017510 | *CYB* |
| Cyprinodontiformes |  | Anablepidae | *Oxyzygonectes dovii* | EF017560 | *ND1* |
| Cyprinodontiformes |  | Anablepidae | *Oxyzygonectes dovii* | EF017560 | *ND2* |
| Cyprinodontiformes |  | Poeciliidae | *Alfaro cultratus* | FJ178772 | *CYB* |
| Cyprinodontiformes |  | Poeciliidae | *Alfaro cultratus* | EF017580 | *ND1* |
| Cyprinodontiformes |  | Poeciliidae | *Aplocheilichthys hutereaui* | AY356594 | *CO1* |
| Cyprinodontiformes |  | Poeciliidae | *Belone belone* | AY141268 | *rhod* |
| Cyprinodontiformes |  | Poeciliidae | *Belonesox belizanus* | EU751663 | *CO1* |
| Cyprinodontiformes |  | Poeciliidae | *Belonesox belizanus* | EF017519 | *CYB* |
| Cyprinodontiformes |  | Poeciliidae | *Belonesox belizanus* | HM443919 | *ND2* |
| Cyprinodontiformes |  | Poeciliidae | *Brachyrhaphis cascajalensis* | FJ178767 | *CYB* |
| Cyprinodontiformes |  | Poeciliidae | *Brachyrhaphis hartwegi* | FJ178769 | *CYB* |
| Cyprinodontiformes |  | Poeciliidae | *Brachyrhaphis hartwegi* | EF017571 | *ND1* |
| Cyprinodontiformes |  | Poeciliidae | *Brachyrhaphis hartwegi* | EF017571 | *ND2* |
| Cyprinodontiformes |  | Poeciliidae | *Brachyrhaphis hartwegi* | FJ185090 | *rag1* |
| Cyprinodontiformes |  | Poeciliidae | *Brachyrhaphis parismina* | FJ178768 | *CYB* |
| Cyprinodontiformes |  | Poeciliidae | *Brachyrhaphis rhabdophora* | EF017522 | *CYB* |
| Cyprinodontiformes |  | Poeciliidae | *Brachyrhaphis rhabdophora* | EF017572 | *ND1* |
| Cyprinodontiformes |  | Poeciliidae | *Brachyrhaphis rhabdophora* | EF017572 | *ND2* |
| Cyprinodontiformes |  | Poeciliidae | *Brachyrhaphis terrabensis* | EF017520 | *CYB* |
| Cyprinodontiformes |  | Poeciliidae | *Brachyrhaphis terrabensis* | EF017570 | *ND1* |
| Cyprinodontiformes |  | Poeciliidae | *Brachyrhaphis terrabensis* | EF017570 | *ND2* |
| Cyprinodontiformes |  | Poeciliidae | *Carlhubbsia kidderi* | FJ178778 | *CYB* |
| Cyprinodontiformes |  | Poeciliidae | *Carlhubbsia kidderi* | FJ185091 | *rag1* |
| Cyprinodontiformes |  | Poeciliidae | *Carlhubbsia stuarti* | EF017532 | *CYB* |
| Cyprinodontiformes |  | Poeciliidae | *Carlhubbsia stuarti* | EF017581 | *ND1* |
| Cyprinodontiformes |  | Poeciliidae | *Carlhubbsia stuarti* | EF017581 | *ND2* |
| Cyprinodontiformes |  | Poeciliidae | *Cnesterodon decemmaculatus* | EF017529 | *CYB* |
| Cyprinodontiformes |  | Poeciliidae | *Cnesterodon decemmaculatus* | EF017579 | *ND1* |
| Cyprinodontiformes |  | Poeciliidae | *Cnesterodon decemmaculatus* | EF017579 | *ND2* |
| Cyprinodontiformes |  | Poeciliidae | *Cnesterodon decemmaculatus* | GU179271 | *rhod* |
| Cyprinodontiformes |  | Poeciliidae | *Cnesterodon hypselurus* | GU179185 | *CYB* |
| Cyprinodontiformes |  | Poeciliidae | *Cnesterodon hypselurus* | GU179231 | *ND2* |
| Cyprinodontiformes |  | Poeciliidae | *Cnesterodon hypselurus* | GU179260 | *rag1* |
| Cyprinodontiformes |  | Poeciliidae | *Cnesterodon hypselurus* | GU179272 | *rhod* |
| Cyprinodontiformes |  | Poeciliidae | *Fluviphylax pygmaeus* | EF017511 | *CYB* |
| Cyprinodontiformes |  | Poeciliidae | *Fluviphylax pygmaeus* | EF017561 | *ND1* |
| Cyprinodontiformes |  | Poeciliidae | *Fluviphylax pygmaeus* | EF017561 | *ND2* |
| Cyprinodontiformes |  | Poeciliidae | *Gambusia affinis* | JN026703 | *CO1* |
| Cyprinodontiformes |  | Poeciliidae | *Gambusia affinis* | EF017514 | *CYB* |
| Cyprinodontiformes |  | Poeciliidae | *Gambusia affinis* | EF017564 | *ND1* |
| Cyprinodontiformes |  | Poeciliidae | *Gambusia affinis* | EF017564 | *ND2* |
| Cyprinodontiformes |  | Poeciliidae | *Gambusia atrora* | EF017515 | *CYB* |
| Cyprinodontiformes |  | Poeciliidae | *Gambusia atrora* | EF017565 | *ND1* |
| Cyprinodontiformes |  | Poeciliidae | *Gambusia atrora* | EF017565 | *ND2* |
| Cyprinodontiformes |  | Poeciliidae | *Gambusia eurystoma* | U18206 | *CYB* |
| Cyprinodontiformes |  | Poeciliidae | *Gambusia geiseri* | U18207 | *CYB* |
| Cyprinodontiformes |  | Poeciliidae | *Gambusia heterochir* | DQ075684 | *CYB* |
| Cyprinodontiformes |  | Poeciliidae | *Gambusia hispaniolae* | U18209 | *CYB* |
| Cyprinodontiformes |  | Poeciliidae | *Gambusia holbrooki* | HM006998 | *ATP6* |
| Cyprinodontiformes |  | Poeciliidae | *Gambusia holbrooki* | GU183103 | *CO1* |
| Cyprinodontiformes |  | Poeciliidae | *Gambusia holbrooki* | GU183104 | *CYB* |
| Cyprinodontiformes |  | Poeciliidae | *Gambusia holbrooki* | HM443936 | *ND2* |
| Cyprinodontiformes |  | Poeciliidae | *Gambusia hubbsi* | U18211 | *CYB* |
| Cyprinodontiformes |  | Poeciliidae | *Gambusia hubbsi* | EF534748 | *ND2* |
| Cyprinodontiformes |  | Poeciliidae | *Gambusia hurtadoi* | U18212 | *CYB* |
| Cyprinodontiformes |  | Poeciliidae | *Gambusia luma* | U18213 | *CYB* |
| Cyprinodontiformes |  | Poeciliidae | *Gambusia manni* | U18214 | *CYB* |
| Cyprinodontiformes |  | Poeciliidae | *Gambusia marshi* | U18215 | *CYB* |
| Cyprinodontiformes |  | Poeciliidae | *Gambusia nicaraguensis* | U18217 | *CYB* |
| Cyprinodontiformes |  | Poeciliidae | *Gambusia oligosticta* | U18218 | *CYB* |
| Cyprinodontiformes |  | Poeciliidae | *Gambusia panuco* | U18219 | *CYB* |
| Cyprinodontiformes |  | Poeciliidae | *Gambusia punctata* | FN545624 | *CO1* |
| Cyprinodontiformes |  | Poeciliidae | *Gambusia punctata* | U18220 | *CYB* |
| Cyprinodontiformes |  | Poeciliidae | *Gambusia puncticulata* | FN545646 | *CO1* |
| Cyprinodontiformes |  | Poeciliidae | *Gambusia rhizophorae* | FN545633 | *CO1* |
| Cyprinodontiformes |  | Poeciliidae | *Gambusia rhizophorae* | U18223 | *CYB* |
| Cyprinodontiformes |  | Poeciliidae | *Gambusia sexradiata* | EU751809 | *CO1* |
| Cyprinodontiformes |  | Poeciliidae | *Gambusia sexradiata* | U18224 | *CYB* |
| Cyprinodontiformes |  | Poeciliidae | *Gambusia vittata* | EF017518 | *CYB* |
| Cyprinodontiformes |  | Poeciliidae | *Gambusia vittata* | EF017568 | *ND1* |
| Cyprinodontiformes |  | Poeciliidae | *Gambusia vittata* | EF017568 | *ND2* |
| Cyprinodontiformes |  | Poeciliidae | *Gambusia wrayi* | EF017516 | *CYB* |
| Cyprinodontiformes |  | Poeciliidae | *Gambusia wrayi* | EF017566 | *ND1* |
| Cyprinodontiformes |  | Poeciliidae | *Gambusia wrayi* | EF017566 | *ND2* |
| Cyprinodontiformes |  | Poeciliidae | *Gambusia yucatana* | EU751813 | *CO1* |
| Cyprinodontiformes |  | Poeciliidae | *Girardinus creolus* | FN545612 | *CO1* |
| Cyprinodontiformes |  | Poeciliidae | *Girardinus creolus* | FJ178745 | *CYB* |
| Cyprinodontiformes |  | Poeciliidae | *Girardinus creolus* | EF017594 | *ND1* |
| Cyprinodontiformes |  | Poeciliidae | *Girardinus creolus* | EF017594 | *ND2* |
| Cyprinodontiformes |  | Poeciliidae | *Girardinus creolus* | FJ185096 | *rag1* |
| Cyprinodontiformes |  | Poeciliidae | *Girardinus denticulatus* | FN545606 | *CO1* |
| Cyprinodontiformes |  | Poeciliidae | *Girardinus denticulatus* | FJ178723 | *CYB* |
| Cyprinodontiformes |  | Poeciliidae | *Girardinus denticulatus* | FJ185102 | *rag1* |
| Cyprinodontiformes |  | Poeciliidae | *Girardinus falcatus* | FN545600 | *CO1* |
| Cyprinodontiformes |  | Poeciliidae | *Girardinus falcatus* | FJ178758 | *CYB* |
| Cyprinodontiformes |  | Poeciliidae | *Girardinus falcatus* | FJ185098 | *rag1* |
| Cyprinodontiformes |  | Poeciliidae | *Girardinus metallicus* | FN545598 | *CO1* |
| Cyprinodontiformes |  | Poeciliidae | *Girardinus metallicus* | FJ178654 | *CYB* |
| Cyprinodontiformes |  | Poeciliidae | *Girardinus metallicus* | EF017593 | *ND1* |
| Cyprinodontiformes |  | Poeciliidae | *Girardinus metallicus* | EF017593 | *ND2* |
| Cyprinodontiformes |  | Poeciliidae | *Girardinus metallicus* | FJ185103 | *rag1* |
| Cyprinodontiformes |  | Poeciliidae | *Girardinus microdactylus* | FN545615 | *CO1* |
| Cyprinodontiformes |  | Poeciliidae | *Girardinus microdactylus* | FJ178688 | *CYB* |
| Cyprinodontiformes |  | Poeciliidae | *Girardinus microdactylus* | FJ185097 | *rag1* |
| Cyprinodontiformes |  | Poeciliidae | *Girardinus ramsdeni* | FJ178733 | *CYB* |
| Cyprinodontiformes |  | Poeciliidae | *Girardinus rivasi* | FJ178697 | *CYB* |
| Cyprinodontiformes |  | Poeciliidae | *Girardinus rivasi* | FJ185099 | *rag1* |
| Cyprinodontiformes |  | Poeciliidae | *Girardinus uninotatus* | FN545605 | *CO1* |
| Cyprinodontiformes |  | Poeciliidae | *Girardinus uninotatus* | FJ178714 | *CYB* |
| Cyprinodontiformes |  | Poeciliidae | *Girardinus uninotatus* | FJ185093 | *rag1* |
| Cyprinodontiformes |  | Poeciliidae | *Heterandria bimaculata* | EU751836 | *CO1* |
| Cyprinodontiformes |  | Poeciliidae | *Heterandria bimaculata* | EF017523 | *CYB* |
| Cyprinodontiformes |  | Poeciliidae | *Heterandria bimaculata* | EF017573 | *ND1* |
| Cyprinodontiformes |  | Poeciliidae | *Heterandria bimaculata* | EF017573 | *ND2* |
| Cyprinodontiformes |  | Poeciliidae | *Heterandria formosa* | JN026765 | *CO1* |
| Cyprinodontiformes |  | Poeciliidae | *Heterandria formosa* | AF412125 | *CYB* |
| Cyprinodontiformes |  | Poeciliidae | *Heterandria formosa* | EF017575 | *ND1* |
| Cyprinodontiformes |  | Poeciliidae | *Heterandria formosa* | EF017575 | *ND2* |
| Cyprinodontiformes |  | Poeciliidae | *Heterandria jonesi* | EF017524 | *CYB* |
| Cyprinodontiformes |  | Poeciliidae | *Heterandria jonesi* | EF017574 | *ND1* |
| Cyprinodontiformes |  | Poeciliidae | *Heterandria jonesi* | EF017574 | *ND2* |
| Cyprinodontiformes |  | Poeciliidae | *Heterophallus milleri* | EF017517 | *CYB* |
| Cyprinodontiformes |  | Poeciliidae | *Heterophallus milleri* | EF017567 | *ND1* |
| Cyprinodontiformes |  | Poeciliidae | *Heterophallus milleri* | EF017567 | *ND2* |
| Cyprinodontiformes |  | Poeciliidae | *Heterophallus rachovii* | U18222 | *CYB* |
| Cyprinodontiformes |  | Poeciliidae | *Heterophallus rachovii* | HM443920 | *ND2* |
| Cyprinodontiformes |  | Poeciliidae | *Limia caymanensis* | AF353192 | *ND2* |
| Cyprinodontiformes |  | Poeciliidae | *Limia dominicensis* | EF017533 | *CYB* |
| Cyprinodontiformes |  | Poeciliidae | *Limia dominicensis* | EF017582 | *ND1* |
| Cyprinodontiformes |  | Poeciliidae | *Limia dominicensis* | EF017582 | *ND2* |
| Cyprinodontiformes |  | Poeciliidae | *Limia dominicensis* | GU179273 | *rhod* |
| Cyprinodontiformes |  | Poeciliidae | *Limia heterandria* | HQ857426 | *CYB* |
| Cyprinodontiformes |  | Poeciliidae | *Limia heterandria* | HQ857450 | *ND2* |
| Cyprinodontiformes |  | Poeciliidae | *Limia heterandria* | HQ857444 | *rag1* |
| Cyprinodontiformes |  | Poeciliidae | *Limia heterandria* | HQ857438 | *rhod* |
| Cyprinodontiformes |  | Poeciliidae | *Limia melanogaster* | EF017534 | *CYB* |
| Cyprinodontiformes |  | Poeciliidae | *Limia melanogaster* | EF017583 | *ND1* |
| Cyprinodontiformes |  | Poeciliidae | *Limia melanogaster* | AF353202 | *ND2* |
| Cyprinodontiformes |  | Poeciliidae | *Limia melanogaster* | GU179274 | *rhod* |
| Cyprinodontiformes |  | Poeciliidae | *Limia melanonotata* | AF353197 | *ND2* |
| Cyprinodontiformes |  | Poeciliidae | *Limia nigrofasciata* | AF031391 | *ND2* |
| Cyprinodontiformes |  | Poeciliidae | *Limia pauciradiata* | AF353196 | *ND2* |
| Cyprinodontiformes |  | Poeciliidae | *Limia perugiae* | AF031392 | *ND2* |
| Cyprinodontiformes |  | Poeciliidae | *Limia tridens* | EF017535 | *CYB* |
| Cyprinodontiformes |  | Poeciliidae | *Limia tridens* | EF017584 | *ND1* |
| Cyprinodontiformes |  | Poeciliidae | *Limia tridens* | EF017584 | *ND2* |
| Cyprinodontiformes |  | Poeciliidae | *Limia versicolor* | AF353193 | *ND2* |
| Cyprinodontiformes |  | Poeciliidae | *Limia vittata* | FN545658 | *CO1* |
| Cyprinodontiformes |  | Poeciliidae | *Limia vittata* | FJ178766 | *CYB* |
| Cyprinodontiformes |  | Poeciliidae | *Limia vittata* | AF353201 | *ND2* |
| Cyprinodontiformes |  | Poeciliidae | *Limia zonata* | AF353194 | *ND2* |
| Cyprinodontiformes |  | Poeciliidae | *Micropoecilia bifurca* | GU179186 | *CYB* |
| Cyprinodontiformes |  | Poeciliidae | *Micropoecilia bifurca* | GU179232 | *ND2* |
| Cyprinodontiformes |  | Poeciliidae | *Micropoecilia bifurca* | GU179261 | *rag1* |
| Cyprinodontiformes |  | Poeciliidae | *Micropoecilia bifurca* | GU179275 | *rhod* |
| Cyprinodontiformes |  | Poeciliidae | *Micropoecilia branneri* | GU179187 | *CYB* |
| Cyprinodontiformes |  | Poeciliidae | *Micropoecilia branneri* | GU179233 | *ND2* |
| Cyprinodontiformes |  | Poeciliidae | *Micropoecilia branneri* | GU179262 | *rag1* |
| Cyprinodontiformes |  | Poeciliidae | *Micropoecilia branneri* | GU179276 | *rhod* |
| Cyprinodontiformes |  | Poeciliidae | *Micropoecilia parae* | GU179189 | *CYB* |
| Cyprinodontiformes |  | Poeciliidae | *Micropoecilia parae* | GU179235 | *ND2* |
| Cyprinodontiformes |  | Poeciliidae | *Micropoecilia parae* | GU179264 | *rag1* |
| Cyprinodontiformes |  | Poeciliidae | *Micropoecilia parae* | GU179277 | *rhod* |
| Cyprinodontiformes |  | Poeciliidae | *Micropoecilia picta* | GU179190 | *CYB* |
| Cyprinodontiformes |  | Poeciliidae | *Micropoecilia picta* | EF017586 | *ND1* |
| Cyprinodontiformes |  | Poeciliidae | *Micropoecilia picta* | AF031395 | *ND2* |
| Cyprinodontiformes |  | Poeciliidae | *Micropoecilia picta* | GU179266 | *rag1* |
| Cyprinodontiformes |  | Poeciliidae | *Micropoecilia picta* | GU179280 | *rhod* |
| Cyprinodontiformes |  | Poeciliidae | *Neoheterandria elegans* | EF017528 | *CYB* |
| Cyprinodontiformes |  | Poeciliidae | *Neoheterandria tridentiger* | EF017526 | *CYB* |
| Cyprinodontiformes |  | Poeciliidae | *Neoheterandria tridentiger* | EF017576 | *ND1* |
| Cyprinodontiformes |  | Poeciliidae | *Neoheterandria tridentiger* | EF017576 | *ND2* |
| Cyprinodontiformes |  | Poeciliidae | *Pamphorichthys araguaiensis* | GU179195 | *CYB* |
| Cyprinodontiformes |  | Poeciliidae | *Pamphorichthys araguaiensis* | AF031398 | *ND2* |
| Cyprinodontiformes |  | Poeciliidae | *Pamphorichthys araguaiensis* | GU179269 | *rag1* |
| Cyprinodontiformes |  | Poeciliidae | *Pamphorichthys araguaiensis* | GU179284 | *rhod* |
| Cyprinodontiformes |  | Poeciliidae | *Pamphorichthys hasemani* | HQ857427 | *CYB* |
| Cyprinodontiformes |  | Poeciliidae | *Pamphorichthys hasemani* | HQ857451 | *ND2* |
| Cyprinodontiformes |  | Poeciliidae | *Pamphorichthys hasemani* | HQ857445 | *rag1* |
| Cyprinodontiformes |  | Poeciliidae | *Pamphorichthys hasemani* | HQ857439 | *rhod* |
| Cyprinodontiformes |  | Poeciliidae | *Pamphorichthys hollandi* | HM405175 | *CO1* |
| Cyprinodontiformes |  | Poeciliidae | *Pamphorichthys hollandi* | EF017538 | *CYB* |
| Cyprinodontiformes |  | Poeciliidae | *Pamphorichthys hollandi* | EF017587 | *ND1* |
| Cyprinodontiformes |  | Poeciliidae | *Pamphorichthys hollandi* | HQ857452 | *ND2* |
| Cyprinodontiformes |  | Poeciliidae | *Pamphorichthys hollandi* | HQ857446 | *rag1* |
| Cyprinodontiformes |  | Poeciliidae | *Pamphorichthys hollandi* | HQ857440 | *rhod* |
| Cyprinodontiformes |  | Poeciliidae | *Pamphorichthys minor* | GU179196 | *CYB* |
| Cyprinodontiformes |  | Poeciliidae | *Pamphorichthys minor* | AF031397 | *ND2* |
| Cyprinodontiformes |  | Poeciliidae | *Pamphorichthys minor* | GU179270 | *rag1* |
| Cyprinodontiformes |  | Poeciliidae | *Pamphorichthys minor* | GU179285 | *rhod* |
| Cyprinodontiformes |  | Poeciliidae | *Pamphorichthys scalpridens* | HQ857429 | *CYB* |
| Cyprinodontiformes |  | Poeciliidae | *Pamphorichthys scalpridens* | HQ857453 | *ND2* |
| Cyprinodontiformes |  | Poeciliidae | *Pamphorichthys scalpridens* | HQ857447 | *rag1* |
| Cyprinodontiformes |  | Poeciliidae | *Pamphorichthys scalpridens* | HQ857441 | *rhod* |
| Cyprinodontiformes |  | Poeciliidae | *Phallichthys amates* | EF017513 | *CYB* |
| Cyprinodontiformes |  | Poeciliidae | *Phallichthys amates* | EF017563 | *ND1* |
| Cyprinodontiformes |  | Poeciliidae | *Phallichthys amates* | EF017563 | *ND2* |
| Cyprinodontiformes |  | Poeciliidae | *Phallichthys pittieri* | FJ178770 | *CYB* |
| Cyprinodontiformes |  | Poeciliidae | *Phallichthys tico* | EF017512 | *CYB* |
| Cyprinodontiformes |  | Poeciliidae | *Phallichthys tico* | EF017562 | *ND1* |
| Cyprinodontiformes |  | Poeciliidae | *Phallichthys tico* | AF412168 | *ND2* |
| Cyprinodontiformes |  | Poeciliidae | *Phalloceros caudimaculatus* | EF017578 | *ND1* |
| Cyprinodontiformes |  | Poeciliidae | *Phalloceros caudimaculatus* | EF017578 | *ND2* |
| Cyprinodontiformes |  | Poeciliidae | *Phalloceros reisi* | HM065013 | *CO1* |
| Cyprinodontiformes |  | Poeciliidae | *Phalloceros uai* | HM404947 | *CO1* |
| Cyprinodontiformes |  | Poeciliidae | *Phalloptychus januarius* | EF017530 | *CYB* |
| Cyprinodontiformes |  | Poeciliidae | *Poecilia butleri* | AF412124 | *CYB* |
| Cyprinodontiformes |  | Poeciliidae | *Poecilia butleri* | AY743248 | *ND2* |
| Cyprinodontiformes |  | Poeciliidae | *Poecilia catemaconis* | AF080487 | *ND2* |
| Cyprinodontiformes |  | Poeciliidae | *Poecilia caucana* | EF017540 | *CYB* |
| Cyprinodontiformes |  | Poeciliidae | *Poecilia caucana* | EF017589 | *ND1* |
| Cyprinodontiformes |  | Poeciliidae | *Poecilia caucana* | EF017589 | *ND2* |
| Cyprinodontiformes |  | Poeciliidae | *Poecilia caucana* | GU179286 | *rhod* |
| Cyprinodontiformes |  | Poeciliidae | *Poecilia gillii* | FJ446282 | *CYB* |
| Cyprinodontiformes |  | Poeciliidae | *Poecilia gillii* | AF031388 | *ND2* |
| Cyprinodontiformes |  | Poeciliidae | *Poecilia latipinna* | JN028261 | *CO1* |
| Cyprinodontiformes |  | Poeciliidae | *Poecilia latipinna* | FJ446153 | *CYB* |
| Cyprinodontiformes |  | Poeciliidae | *Poecilia latipinna* | AF031389 | *ND2* |
| Cyprinodontiformes |  | Poeciliidae | *Poecilia latipunctata* | EF017539 | *CYB* |
| Cyprinodontiformes |  | Poeciliidae | *Poecilia latipunctata* | EF017588 | *ND1* |
| Cyprinodontiformes |  | Poeciliidae | *Poecilia latipunctata* | AF080489 | *ND2* |
| Cyprinodontiformes |  | Poeciliidae | *Poecilia latipunctata* | GU179287 | *rhod* |
| Cyprinodontiformes |  | Poeciliidae | *Poecilia mexicana* | EU751936 | *CO1* |
| Cyprinodontiformes |  | Poeciliidae | *Poecilia mexicana* | FJ178776 | *CYB* |
| Cyprinodontiformes |  | Poeciliidae | *Poecilia mexicana limantouri* | AF080488 | *ND2* |
| Cyprinodontiformes |  | Poeciliidae | *Poecilia orri* | AF031400 | *ND2* |
| Cyprinodontiformes |  | Poeciliidae | *Poecilia petenensis* | EU751941 | *CO1* |
| Cyprinodontiformes |  | Poeciliidae | *Poecilia petenensis* | AF031401 | *ND2* |
| Cyprinodontiformes |  | Poeciliidae | *Poecilia reticulata* | JN028265 | *CO1* |
| Cyprinodontiformes |  | Poeciliidae | *Poecilia reticulata* | EF017536 | *CYB* |
| Cyprinodontiformes |  | Poeciliidae | *Poecilia reticulata* | EF017585 | *ND1* |
| Cyprinodontiformes |  | Poeciliidae | *Poecilia reticulata* | EF017585 | *ND2* |
| Cyprinodontiformes |  | Poeciliidae | *Poecilia reticulata* | AY141269 | *rhod* |
| Cyprinodontiformes |  | Poeciliidae | *Poecilia sphenops* | JN028268 | *CO1* |
| Cyprinodontiformes |  | Poeciliidae | *Poecilia sphenops* | AF031390 | *ND2* |
| Cyprinodontiformes |  | Poeciliidae | *Poecilia sulphuraria* | AF080490 | *ND2* |
| Cyprinodontiformes |  | Poeciliidae | *Poecilia velifera* | AF031402 | *ND2* |
| Cyprinodontiformes |  | Poeciliidae | *Poecilia vivipara* | HQ857430 | *CYB* |
| Cyprinodontiformes |  | Poeciliidae | *Poecilia vivipara* | HQ857455 | *ND2* |
| Cyprinodontiformes |  | Poeciliidae | *Poecilia vivipara* | HQ857449 | *rag1* |
| Cyprinodontiformes |  | Poeciliidae | *Poecilia vivipara* | HQ857442 | *rhod* |
| Cyprinodontiformes |  | Poeciliidae | *Poecilia wingei* | GU179193 | *CYB* |
| Cyprinodontiformes |  | Poeciliidae | *Poecilia wingei* | GU179239 | *ND2* |
| Cyprinodontiformes |  | Poeciliidae | *Poecilia wingei* | GU179268 | *rag1* |
| Cyprinodontiformes |  | Poeciliidae | *Poecilia wingei* | GU179282 | *rhod* |
| Cyprinodontiformes |  | Poeciliidae | *Poeciliopsis baenschi* | AF412148 | *CYB* |
| Cyprinodontiformes |  | Poeciliidae | *Poeciliopsis baenschi* | AF412191 | *ND2* |
| Cyprinodontiformes |  | Poeciliidae | *Poeciliopsis catemaco* | EU751945 | *CO1* |
| Cyprinodontiformes |  | Poeciliidae | *Poeciliopsis catemaco* | AF412161 | *CYB* |
| Cyprinodontiformes |  | Poeciliidae | *Poeciliopsis catemaco* | AF412201 | *ND2* |
| Cyprinodontiformes |  | Poeciliidae | *Poeciliopsis elongata* | AF412129 | *CYB* |
| Cyprinodontiformes |  | Poeciliidae | *Poeciliopsis elongata* | AF412172 | *ND2* |
| Cyprinodontiformes |  | Poeciliidae | *Poeciliopsis fasciata* | AF412150 | *CYB* |
| Cyprinodontiformes |  | Poeciliidae | *Poeciliopsis fasciata* | EF017595 | *ND1* |
| Cyprinodontiformes |  | Poeciliidae | *Poeciliopsis fasciata* | AF412193 | *ND2* |
| Cyprinodontiformes |  | Poeciliidae | *Poeciliopsis gracilis* | JN028273 | *CO1* |
| Cyprinodontiformes |  | Poeciliidae | *Poeciliopsis gracilis* | AF412155 | *CYB* |
| Cyprinodontiformes |  | Poeciliidae | *Poeciliopsis gracilis* | AF412200 | *ND2* |
| Cyprinodontiformes |  | Poeciliidae | *Poeciliopsis hnilickai* | AF412156 | *CYB* |
| Cyprinodontiformes |  | Poeciliidae | *Poeciliopsis hnilickai* | EF017596 | *ND1* |
| Cyprinodontiformes |  | Poeciliidae | *Poeciliopsis hnilickai* | EF017596 | *ND2* |
| Cyprinodontiformes |  | Poeciliidae | *Poeciliopsis infans* | AF412135 | *CYB* |
| Cyprinodontiformes |  | Poeciliidae | *Poeciliopsis infans* | AF412179 | *ND2* |
| Cyprinodontiformes |  | Poeciliidae | *Poeciliopsis latidens* | AF412151 | *CYB* |
| Cyprinodontiformes |  | Poeciliidae | *Poeciliopsis latidens* | AF412194 | *ND2* |
| Cyprinodontiformes |  | Poeciliidae | *Poeciliopsis lucida* | AF412139 | *CYB* |
| Cyprinodontiformes |  | Poeciliidae | *Poeciliopsis lucida* | AF412184 | *ND2* |
| Cyprinodontiformes |  | Poeciliidae | *Poeciliopsis monacha* | AF047346 | *CYB* |
| Cyprinodontiformes |  | Poeciliidae | *Poeciliopsis monacha* | AF412173 | *ND2* |
| Cyprinodontiformes |  | Poeciliidae | *Poeciliopsis occidentalis* | AF412141 | *CYB* |
| Cyprinodontiformes |  | Poeciliidae | *Poeciliopsis occidentalis* | DQ138946 | *ND2* |
| Cyprinodontiformes |  | Poeciliidae | *Poeciliopsis occidentalis sonorensis* | DQ138944 | *CYB* |
| Cyprinodontiformes |  | Poeciliidae | *Poeciliopsis occidentalis sonorensis* | DQ138947 | *ND2* |
| Cyprinodontiformes |  | Poeciliidae | *Poeciliopsis paucimaculata* | AF412128 | *CYB* |
| Cyprinodontiformes |  | Poeciliidae | *Poeciliopsis paucimaculata* | AF412171 | *ND2* |
| Cyprinodontiformes |  | Poeciliidae | *Poeciliopsis pleurospilus* | EU751949 | *CO1* |
| Cyprinodontiformes |  | Poeciliidae | *Poeciliopsis presidionis* | AF412157 | *CYB* |
| Cyprinodontiformes |  | Poeciliidae | *Poeciliopsis presidionis* | AY743254 | *ND2* |
| Cyprinodontiformes |  | Poeciliidae | *Poeciliopsis prolifica* | AF412146 | *CYB* |
| Cyprinodontiformes |  | Poeciliidae | *Poeciliopsis prolifica* | AF412190 | *ND2* |
| Cyprinodontiformes |  | Poeciliidae | *Poeciliopsis retropinna* | AF412130 | *CYB* |
| Cyprinodontiformes |  | Poeciliidae | *Poeciliopsis scarlli* | AF412159 | *CYB* |
| Cyprinodontiformes |  | Poeciliidae | *Poeciliopsis scarlli* | AF412198 | *ND2* |
| Cyprinodontiformes |  | Poeciliidae | *Poeciliopsis turneri* | AF412158 | *CYB* |
| Cyprinodontiformes |  | Poeciliidae | *Poeciliopsis turneri* | AF412197 | *ND2* |
| Cyprinodontiformes |  | Poeciliidae | *Poeciliopsis turrubarensis* | AF412163 | *CYB* |
| Cyprinodontiformes |  | Poeciliidae | *Poeciliopsis turrubarensis* | AF412203 | *ND2* |
| Cyprinodontiformes |  | Poeciliidae | *Poeciliopsis viriosa* | AF412133 | *CYB* |
| Cyprinodontiformes |  | Poeciliidae | *Poeciliopsis viriosa* | AF412175 | *ND2* |
| Cyprinodontiformes |  | Poeciliidae | *Priapella chamulae* | JF892548 | *CYB* |
| Cyprinodontiformes |  | Poeciliidae | *Priapella compressa* | EF017554 | *CYB* |
| Cyprinodontiformes |  | Poeciliidae | *Priapella compressa* | EF017603 | *ND1* |
| Cyprinodontiformes |  | Poeciliidae | *Priapella compressa* | EF017603 | *ND2* |
| Cyprinodontiformes |  | Poeciliidae | *Priapella compressa* | DQ235860 | *rag1* |
| Cyprinodontiformes |  | Poeciliidae | *Priapella intermedia* | AY356595 | *CO1* |
| Cyprinodontiformes |  | Poeciliidae | *Priapella intermedia* | EF017553 | *CYB* |
| Cyprinodontiformes |  | Poeciliidae | *Priapella intermedia* | EF017602 | *ND1* |
| Cyprinodontiformes |  | Poeciliidae | *Priapella intermedia* | EF017602 | *ND2* |
| Cyprinodontiformes |  | Poeciliidae | *Priapella lacandonae* | JF892549 | *CYB* |
| Cyprinodontiformes |  | Poeciliidae | *Priapella olmecae* | EF017555 | *CYB* |
| Cyprinodontiformes |  | Poeciliidae | *Priapella olmecae* | EF017604 | *ND1* |
| Cyprinodontiformes |  | Poeciliidae | *Priapella olmecae* | EF017604 | *ND2* |
| Cyprinodontiformes |  | Poeciliidae | *Priapella olmecae* | DQ235861 | *rag1* |
| Cyprinodontiformes |  | Poeciliidae | *Priapichthys annectens* | FJ518864 | *CYB* |
| Cyprinodontiformes |  | Poeciliidae | *Priapichthys annectens* | EF017591 | *ND1* |
| Cyprinodontiformes |  | Poeciliidae | *Priapichthys annectens* | EF017591 | *ND2* |
| Cyprinodontiformes |  | Poeciliidae | *Pseudopoecilia festae* | EF017543 | *CYB* |
| Cyprinodontiformes |  | Poeciliidae | *Pseudopoecilia festae* | EF017592 | *ND1* |
| Cyprinodontiformes |  | Poeciliidae | *Pseudopoecilia festae* | GQ995777 | *ND2* |
| Cyprinodontiformes |  | Poeciliidae | *Quintana atrizona* | FN545619 | *CO1* |
| Cyprinodontiformes |  | Poeciliidae | *Quintana atrizona* | EF017556 | *CYB* |
| Cyprinodontiformes |  | Poeciliidae | *Quintana atrizona* | EF017605 | *ND1* |
| Cyprinodontiformes |  | Poeciliidae | *Quintana atrizona* | EF017605 | *ND2* |
| Cyprinodontiformes |  | Poeciliidae | *Quintana atrizona* | FJ185092 | *rag1* |
| Cyprinodontiformes |  | Poeciliidae | *Scolichthys greenwayi* | EF017541 | *CYB* |
| Cyprinodontiformes |  | Poeciliidae | *Scolichthys greenwayi* | EF017590 | *ND1* |
| Cyprinodontiformes |  | Poeciliidae | *Scolichthys greenwayi* | EF017590 | *ND2* |
| Cyprinodontiformes |  | Poeciliidae | *Tomeurus gracilis* | EF017607 | *ND1* |
| Cyprinodontiformes |  | Poeciliidae | *Xenodexia ctenolepis* | EF017557 | *CYB* |
| Cyprinodontiformes |  | Poeciliidae | *Xenodexia ctenolepis* | EF017606 | *ND1* |
| Cyprinodontiformes |  | Poeciliidae | *Xenodexia ctenolepis* | EF017606 | *ND2* |
| Cyprinodontiformes |  | Poeciliidae | *Xenophallus umbratilis* | FJ518836 | *CYB* |
| Cyprinodontiformes |  | Poeciliidae | *Xenophallus umbratilis* | EF017577 | *ND2* |
| Cyprinodontiformes |  | Poeciliidae | *Xiphophorus alvarezi* | EU752036 | *CO1* |
| Cyprinodontiformes |  | Poeciliidae | *Xiphophorus alvarezi* | DQ235873 | *rag1* |
| Cyprinodontiformes |  | Poeciliidae | *Xiphophorus andersi* | DQ235870 | *rag1* |
| Cyprinodontiformes |  | Poeciliidae | *Xiphophorus birchmanni* | DQ235878 | *rag1* |
| Cyprinodontiformes |  | Poeciliidae | *Xiphophorus clemenciae* | EF017552 | *CYB* |
| Cyprinodontiformes |  | Poeciliidae | *Xiphophorus clemenciae* | EF017601 | *ND1* |
| Cyprinodontiformes |  | Poeciliidae | *Xiphophorus clemenciae* | EF017601 | *ND2* |
| Cyprinodontiformes |  | Poeciliidae | *Xiphophorus clemenciae* | DQ235883 | *rag1* |
| Cyprinodontiformes |  | Poeciliidae | *Xiphophorus continens* | DQ235863 | *rag1* |
| Cyprinodontiformes |  | Poeciliidae | *Xiphophorus cortezi* | DQ235868 | *rag1* |
| Cyprinodontiformes |  | Poeciliidae | *Xiphophorus couchianus* | DQ235869 | *rag1* |
| Cyprinodontiformes |  | Poeciliidae | *Xiphophorus evelynae* | EF017550 | *CYB* |
| Cyprinodontiformes |  | Poeciliidae | *Xiphophorus evelynae* | EF017599 | *ND1* |
| Cyprinodontiformes |  | Poeciliidae | *Xiphophorus evelynae* | EF017599 | *ND2* |
| Cyprinodontiformes |  | Poeciliidae | *Xiphophorus evelynae* | DQ235882 | *rag1* |
| Cyprinodontiformes |  | Poeciliidae | *Xiphophorus gordoni* | DQ235866 | *rag1* |
| Cyprinodontiformes |  | Poeciliidae | *Xiphophorus hellerii* | EU752040 | *CO1* |
| Cyprinodontiformes |  | Poeciliidae | *Xiphophorus hellerii* | AY056056 | *CYB* |
| Cyprinodontiformes |  | Poeciliidae | *Xiphophorus hellerii* | EF017597 | *ND1* |
| Cyprinodontiformes |  | Poeciliidae | *Xiphophorus hellerii* | EF017597 | *ND2* |
| Cyprinodontiformes |  | Poeciliidae | *Xiphophorus hellerii* | DQ235872 | *rag1* |
| Cyprinodontiformes |  | Poeciliidae | *Xiphophorus hellerii* | GU454735 | *rhod* |
| Cyprinodontiformes |  | Poeciliidae | *Xiphophorus maculatus* | EU752046 | *CO1* |
| Cyprinodontiformes |  | Poeciliidae | *Xiphophorus maculatus* | EF017551 | *CYB* |
| Cyprinodontiformes |  | Poeciliidae | *Xiphophorus maculatus* | EF017600 | *ND1* |
| Cyprinodontiformes |  | Poeciliidae | *Xiphophorus maculatus* | EF017600 | *ND2* |
| Cyprinodontiformes |  | Poeciliidae | *Xiphophorus maculatus* | DQ235880 | *rag1* |
| Cyprinodontiformes |  | Poeciliidae | *Xiphophorus malinche* | DQ235867 | *rag1* |
| Cyprinodontiformes |  | Poeciliidae | *Xiphophorus mayae* | AF404296 | *CYB* |
| Cyprinodontiformes |  | Poeciliidae | *Xiphophorus meyeri* | DQ235875 | *rag1* |
| Cyprinodontiformes |  | Poeciliidae | *Xiphophorus milleri* | DQ235864 | *rag1* |
| Cyprinodontiformes |  | Poeciliidae | *Xiphophorus montezumae* | DQ235876 | *rag1* |
| Cyprinodontiformes |  | Poeciliidae | *Xiphophorus multilineatus* | DQ235874 | *rag1* |
| Cyprinodontiformes |  | Poeciliidae | *Xiphophorus nigrensis* | AF031386 | *ND2* |
| Cyprinodontiformes |  | Poeciliidae | *Xiphophorus nigrensis* | DQ235862 | *rag1* |
| Cyprinodontiformes |  | Poeciliidae | *Xiphophorus pygmaeus* | DQ235865 | *rag1* |
| Cyprinodontiformes |  | Poeciliidae | *Xiphophorus signum* | DQ235871 | *rag1* |
| Cyprinodontiformes |  | Poeciliidae | *Xiphophorus variatus* | DQ235881 | *rag1* |
| Cyprinodontiformes |  | Poeciliidae | *Xiphophorus xiphidium* | EF017549 | *CYB* |
| Cyprinodontiformes |  | Poeciliidae | *Xiphophorus xiphidium* | EF017598 | *ND1* |
| Cyprinodontiformes |  | Poeciliidae | *Xiphophorus xiphidium* | EF017598 | *ND2* |
| Cyprinodontiformes |  | Poeciliidae | *Xiphophorus xiphidium* | DQ235879 | *rag1* |
| Gasterosteiformes | Gasterosteoidei | Hypoptychidae | *Hypoptychus dybowskii* | AB445176 | *rag1* |
| Gasterosteiformes | Gasterosteoidei | Aulorhynchidae | *Aulichthys japonicus* | AB445177 | *rag1* |
| Gasterosteiformes | Gasterosteoidei | Aulorhynchidae | *Aulorhynchus flavidus* | AY283340 | *ATP6* |
| Gasterosteiformes | Gasterosteoidei | Aulorhynchidae | *Aulorhynchus flavidus* | FJ164340 | *CO1* |
| Gasterosteiformes | Gasterosteoidei | Aulorhynchidae | *Aulorhynchus flavidus* | AY283298 | *CYB* |
| Gasterosteiformes | Gasterosteoidei | Gasterosteidae | *Apeltes quadracus* | AY283342 | *ATP6* |
| Gasterosteiformes | Gasterosteoidei | Gasterosteidae | *Apeltes quadracus* | EU524443 | *CO1* |
| Gasterosteiformes | Gasterosteoidei | Gasterosteidae | *Apeltes quadracus* | AY283300 | *CYB* |
| Gasterosteiformes | Gasterosteoidei | Gasterosteidae | *Apeltes quadracus* | AB445179 | *rag1* |
| Gasterosteiformes | Gasterosteoidei | Gasterosteidae | *Culaea inconstans* | DQ359855 | *ATP6* |
| Gasterosteiformes | Gasterosteoidei | Gasterosteidae | *Culaea inconstans* | EU524538 | *CO1* |
| Gasterosteiformes | Gasterosteoidei | Gasterosteidae | *Culaea inconstans* | DQ359764 | *CYB* |
| Gasterosteiformes | Gasterosteoidei | Gasterosteidae | *Culaea inconstans* | AB445180 | *rag1* |
| Gasterosteiformes | Gasterosteoidei | Gasterosteidae | *Gasterosteus aculeatus* | AY787224 | *CYB* |
| Gasterosteiformes | Gasterosteoidei | Gasterosteidae | *Gasterosteus aculeatus* | EF033039 | *rag1* |
| Gasterosteiformes | Gasterosteoidei | Gasterosteidae | *Gasterosteus aculeatus* | EU637962 | *rhod* |
| Gasterosteiformes | Gasterosteoidei | Gasterosteidae | *Gasterosteus wheatlandi* | AY283343 | *ATP6* |
| Gasterosteiformes | Gasterosteoidei | Gasterosteidae | *Gasterosteus wheatlandi* | EU524641 | *CO1* |
| Gasterosteiformes | Gasterosteoidei | Gasterosteidae | *Gasterosteus wheatlandi* | AF356078 | *CYB* |
| Gasterosteiformes | Gasterosteoidei | Gasterosteidae | *Gasterosteus wheatlandi* | AB445181 | *rag1* |
| Gasterosteiformes | Gasterosteoidei | Gasterosteidae | *Pungitius kaibarae* | FJ197681 | *CO1* |
| Gasterosteiformes | Gasterosteoidei | Gasterosteidae | *Pungitius kaibarae* | AY787225 | *CYB* |
| Gasterosteiformes | Gasterosteoidei | Gasterosteidae | *Pungitius pungitius* | AY283348 | *ATP6* |
| Gasterosteiformes | Gasterosteoidei | Gasterosteidae | *Pungitius pungitius* | EU524320 | *CO1* |
| Gasterosteiformes | Gasterosteoidei | Gasterosteidae | *Pungitius pungitius* | AF356080 | *CYB* |
| Gasterosteiformes | Gasterosteoidei | Gasterosteidae | *Pungitius pungitius* | AB445183 | *rag1* |
| Gasterosteiformes | Gasterosteoidei | Gasterosteidae | *Pungitius pungitius pungitius* | AB094628 | *CYB* |
| Gasterosteiformes | Gasterosteoidei | Gasterosteidae | *Spinachia spinachia* | AY283341 | *ATP6* |
| Gasterosteiformes | Gasterosteoidei | Gasterosteidae | *Spinachia spinachia* | AY283299 | *CYB* |
| Gasterosteiformes | Gasterosteoidei | Gasterosteidae | *Spinachia spinachia* | AB445184 | *rag1* |
| Gasterosteiformes | Gasterosteoidei | Gasterosteidae | *Spinachia spinachia* | AY141281 | *rhod* |
| Gasterosteiformes | Gasterosteoidei | Indostomidae | *Indostomus paradoxus* | EU637967 | *rhod* |
| Gasterosteiformes | Syngnathoidei | Syngnathidae | *Bryx dunckeri* | GU224734 | *CO1* |
| Gasterosteiformes | Syngnathoidei | Syngnathidae | *Corythoichthys haematopterus* | AY787229 | *CYB* |
| Gasterosteiformes | Syngnathoidei | Syngnathidae | *Corythoichthys intestinalis* | GQ502114 | *CO1* |
| Gasterosteiformes | Syngnathoidei | Syngnathidae | *Corythoichthys intestinalis* | AF356052 | *CYB* |
| Gasterosteiformes | Syngnathoidei | Syngnathidae | *Cosmocampus elucens* | FJ583271 | *CO1* |
| Gasterosteiformes | Syngnathoidei | Syngnathidae | *Doryrhamphus dactyliophorus* | FJ583371 | *CO1* |
| Gasterosteiformes | Syngnathoidei | Syngnathidae | *Doryrhamphus dactyliophorus* | AY787231 | *CYB* |
| Gasterosteiformes | Syngnathoidei | Syngnathidae | *Doryrhamphus excisus* | DQ111300 | *ATP6* |
| Gasterosteiformes | Syngnathoidei | Syngnathidae | *Doryrhamphus excisus* | AF356075 | *CYB* |
| Gasterosteiformes | Syngnathoidei | Syngnathidae | *Doryrhamphus excisus* | FJ905778 | *rag1* |
| Gasterosteiformes | Syngnathoidei | Syngnathidae | *Doryrhamphus excisus* | FJ905800 | *rhod* |
| Gasterosteiformes | Syngnathoidei | Syngnathidae | *Doryrhamphus pessuliferus* | FJ583377 | *CO1* |
| Gasterosteiformes | Syngnathoidei | Syngnathidae | *Entelurus aequoreus* | EU148162 | *CO1* |
| Gasterosteiformes | Syngnathoidei | Syngnathidae | *Entelurus aequoreus* | AF356044 | *CYB* |
| Gasterosteiformes | Syngnathoidei | Syngnathidae | *Hippichthys cyanospilos* | GQ502116 | *CO1* |
| Gasterosteiformes | Syngnathoidei | Syngnathidae | *Hippichthys penicillus* | AF356053 | *CYB* |
| Gasterosteiformes | Syngnathoidei | Syngnathidae | *Hippichthys penicillus* | FJ905774 | *rag1* |
| Gasterosteiformes | Syngnathoidei | Syngnathidae | *Hippichthys penicillus* | FJ905795 | *rhod* |
| Gasterosteiformes | Syngnathoidei | Syngnathidae | *Hippocampus abdominalis* | GQ502118 | *CO1* |
| Gasterosteiformes | Syngnathoidei | Syngnathidae | *Hippocampus abdominalis* | AF356065 | *CYB* |
| Gasterosteiformes | Syngnathoidei | Syngnathidae | *Hippocampus algiricus* | GQ502119 | *CO1* |
| Gasterosteiformes | Syngnathoidei | Syngnathidae | *Hippocampus algiricus* | AF192643 | *CYB* |
| Gasterosteiformes | Syngnathoidei | Syngnathidae | *Hippocampus angustus* | GQ502122 | *CO1* |
| Gasterosteiformes | Syngnathoidei | Syngnathidae | *Hippocampus angustus* | DQ068467 | *CYB* |
| Gasterosteiformes | Syngnathoidei | Syngnathidae | *Hippocampus barbouri* | GQ502124 | *CO1* |
| Gasterosteiformes | Syngnathoidei | Syngnathidae | *Hippocampus barbouri* | AF192646 | *CYB* |
| Gasterosteiformes | Syngnathoidei | Syngnathidae | *Hippocampus bargibanti* | FJ905772 | *rag1* |
| Gasterosteiformes | Syngnathoidei | Syngnathidae | *Hippocampus bargibanti* | FJ905793 | *rhod* |
| Gasterosteiformes | Syngnathoidei | Syngnathidae | *Hippocampus biocellatus* | GQ502126 | *CO1* |
| Gasterosteiformes | Syngnathoidei | Syngnathidae | *Hippocampus breviceps* | AF192647 | *CYB* |
| Gasterosteiformes | Syngnathoidei | Syngnathidae | *Hippocampus breviceps* | FJ905771 | *rag1* |
| Gasterosteiformes | Syngnathoidei | Syngnathidae | *Hippocampus breviceps* | FJ905792 | *rhod* |
| Gasterosteiformes | Syngnathoidei | Syngnathidae | *Hippocampus camelopardalis* | GQ502127 | *CO1* |
| Gasterosteiformes | Syngnathoidei | Syngnathidae | *Hippocampus camelopardalis* | AF192649 | *CYB* |
| Gasterosteiformes | Syngnathoidei | Syngnathidae | *Hippocampus capensis* | GQ502129 | *CO1* |
| Gasterosteiformes | Syngnathoidei | Syngnathidae | *Hippocampus capensis* | AF192650 | *CYB* |
| Gasterosteiformes | Syngnathoidei | Syngnathidae | *Hippocampus comes* | GQ502135 | *CO1* |
| Gasterosteiformes | Syngnathoidei | Syngnathidae | *Hippocampus comes* | AF192657 | *CYB* |
| Gasterosteiformes | Syngnathoidei | Syngnathidae | *Hippocampus coronatus* | AF192658 | *CYB* |
| Gasterosteiformes | Syngnathoidei | Syngnathidae | *Hippocampus erectus* | GQ502136 | *CO1* |
| Gasterosteiformes | Syngnathoidei | Syngnathidae | *Hippocampus erectus* | AF192662 | *CYB* |
| Gasterosteiformes | Syngnathoidei | Syngnathidae | *Hippocampus fisheri* | GQ502139 | *CO1* |
| Gasterosteiformes | Syngnathoidei | Syngnathidae | *Hippocampus fisheri* | DQ288350 | *CYB* |
| Gasterosteiformes | Syngnathoidei | Syngnathidae | *Hippocampus fuscus* | GQ502140 | *CO1* |
| Gasterosteiformes | Syngnathoidei | Syngnathidae | *Hippocampus fuscus* | DQ288354 | *CYB* |
| Gasterosteiformes | Syngnathoidei | Syngnathidae | *Hippocampus guttulatus* | GQ502141 | *CO1* |
| Gasterosteiformes | Syngnathoidei | Syngnathidae | *Hippocampus guttulatus* | AF192664 | *CYB* |
| Gasterosteiformes | Syngnathoidei | Syngnathidae | *Hippocampus hippocampus* | AF192666 | *CYB* |
| Gasterosteiformes | Syngnathoidei | Syngnathidae | *Hippocampus histrix* | GQ502147 | *CO1* |
| Gasterosteiformes | Syngnathoidei | Syngnathidae | *Hippocampus histrix* | AF192671 | *CYB* |
| Gasterosteiformes | Syngnathoidei | Syngnathidae | *Hippocampus ingens* | GQ502148 | *CO1* |
| Gasterosteiformes | Syngnathoidei | Syngnathidae | *Hippocampus ingens* | AF192672 | *CYB* |
| Gasterosteiformes | Syngnathoidei | Syngnathidae | *Hippocampus ingens* | FJ905769 | *rag1* |
| Gasterosteiformes | Syngnathoidei | Syngnathidae | *Hippocampus ingens* | FJ905791 | *rhod* |
| Gasterosteiformes | Syngnathoidei | Syngnathidae | *Hippocampus kelloggi* | GQ502150 | *CO1* |
| Gasterosteiformes | Syngnathoidei | Syngnathidae | *Hippocampus kelloggi* | AF192675 | *CYB* |
| Gasterosteiformes | Syngnathoidei | Syngnathidae | *Hippocampus kuda* | FJ176586 | *CO1* |
| Gasterosteiformes | Syngnathoidei | Syngnathidae | *Hippocampus kuda* | AF356063 | *CYB* |
| Gasterosteiformes | Syngnathoidei | Syngnathidae | *Hippocampus mohnikei* | GQ502159 | *CO1* |
| Gasterosteiformes | Syngnathoidei | Syngnathidae | *Hippocampus mohnikei* | AF192688 | *CYB* |
| Gasterosteiformes | Syngnathoidei | Syngnathidae | *Hippocampus patagonicus* | GQ502160 | *CO1* |
| Gasterosteiformes | Syngnathoidei | Syngnathidae | *Hippocampus patagonicus* | EU871944 | *CYB* |
| Gasterosteiformes | Syngnathoidei | Syngnathidae | *Hippocampus queenslandicus* | GQ502161 | *CO1* |
| Gasterosteiformes | Syngnathoidei | Syngnathidae | *Hippocampus queenslandicus* | AY629245 | *CYB* |
| Gasterosteiformes | Syngnathoidei | Syngnathidae | *Hippocampus ramulosus* | AY368330 | *rhod* |
| Gasterosteiformes | Syngnathoidei | Syngnathidae | *Hippocampus reidi* | GQ502165 | *CO1* |
| Gasterosteiformes | Syngnathoidei | Syngnathidae | *Hippocampus reidi* | AF192692 | *CYB* |
| Gasterosteiformes | Syngnathoidei | Syngnathidae | *Hippocampus reidi* | FJ905790 | *rhod* |
| Gasterosteiformes | Syngnathoidei | Syngnathidae | *Hippocampus spinosissimus* | GQ502169 | *CO1* |
| Gasterosteiformes | Syngnathoidei | Syngnathidae | *Hippocampus spinosissimus* | AF192695 | *CYB* |
| Gasterosteiformes | Syngnathoidei | Syngnathidae | *Hippocampus subelongatus* | GQ502170 | *CO1* |
| Gasterosteiformes | Syngnathoidei | Syngnathidae | *Hippocampus subelongatus* | AF192697 | *CYB* |
| Gasterosteiformes | Syngnathoidei | Syngnathidae | *Hippocampus trimaculatus* | JF700168 | *CO1* |
| Gasterosteiformes | Syngnathoidei | Syngnathidae | *Hippocampus trimaculatus* | AF192700 | *CYB* |
| Gasterosteiformes | Syngnathoidei | Syngnathidae | *Hippocampus whitei* | AF192704 | *CYB* |
| Gasterosteiformes | Syngnathoidei | Syngnathidae | *Hippocampus zosterae* | GQ502176 | *CO1* |
| Gasterosteiformes | Syngnathoidei | Syngnathidae | *Hippocampus zosterae* | AF356071 | *CYB* |
| Gasterosteiformes | Syngnathoidei | Syngnathidae | *Hippocampus zosterae* | FJ905770 | *rag1* |
| Gasterosteiformes | Syngnathoidei | Syngnathidae | *Hypselognathus rostratus* | AF356072 | *CYB* |
| Gasterosteiformes | Syngnathoidei | Syngnathidae | *Idiotropiscis lumnitzeri* | FJ905773 | *rag1* |
| Gasterosteiformes | Syngnathoidei | Syngnathidae | *Idiotropiscis lumnitzeri* | FJ905794 | *rhod* |
| Gasterosteiformes | Syngnathoidei | Syngnathidae | *Kaupus costatus* | AF356074 | *CYB* |
| Gasterosteiformes | Syngnathoidei | Syngnathidae | *Microphis brachyurus brachyurus* | AF356046 | *CYB* |
| Gasterosteiformes | Syngnathoidei | Syngnathidae | *Nerophis lumbriciformis* | EU637987 | *rhod* |
| Gasterosteiformes | Syngnathoidei | Syngnathidae | *Nerophis ophidion* | AF356043 | *CYB* |
| Gasterosteiformes | Syngnathoidei | Syngnathidae | *Phycodurus eques* | GU182932 | *CYB* |
| Gasterosteiformes | Syngnathoidei | Syngnathidae | *Phyllopteryx taeniolatus* | AY787226 | *CYB* |
| Gasterosteiformes | Syngnathoidei | Syngnathidae | *Solegnathus dunckeri* | GQ502177 | *CO1* |
| Gasterosteiformes | Syngnathoidei | Syngnathidae | *Solegnathus dunckeri* | GU182935 | *CYB* |
| Gasterosteiformes | Syngnathoidei | Syngnathidae | *Solegnathus guentheri* | GU182934 | *CYB* |
| Gasterosteiformes | Syngnathoidei | Syngnathidae | *Solegnathus hardwickii* | AY166829 | *CYB* |
| Gasterosteiformes | Syngnathoidei | Syngnathidae | *Stigmatopora argus* | AF356045 | *CYB* |
| Gasterosteiformes | Syngnathoidei | Syngnathidae | *Stigmatopora nigra* | AF356067 | *CYB* |
| Gasterosteiformes | Syngnathoidei | Syngnathidae | *Stigmatopora nigra* | FJ905776 | *rag1* |
| Gasterosteiformes | Syngnathoidei | Syngnathidae | *Stigmatopora nigra* | FJ905797 | *rhod* |
| Gasterosteiformes | Syngnathoidei | Syngnathidae | *Syngnathoides biaculeatus* | JF700183 | *CO1* |
| Gasterosteiformes | Syngnathoidei | Syngnathidae | *Syngnathoides biaculeatus* | AY786432 | *CYB* |
| Gasterosteiformes | Syngnathoidei | Syngnathidae | *Syngnathus abaster* | AF356060 | *CYB* |
| Gasterosteiformes | Syngnathoidei | Syngnathidae | *Syngnathus acus* | GQ502180 | *CO1* |
| Gasterosteiformes | Syngnathoidei | Syngnathidae | *Syngnathus acus* | AF356040 | *CYB* |
| Gasterosteiformes | Syngnathoidei | Syngnathidae | *Syngnathus auliscus* | HQ010060 | *CO1* |
| Gasterosteiformes | Syngnathoidei | Syngnathidae | *Syngnathus californiensis* | GU440543 | *CO1* |
| Gasterosteiformes | Syngnathoidei | Syngnathidae | *Syngnathus floridae* | GU224625 | *CO1* |
| Gasterosteiformes | Syngnathoidei | Syngnathidae | *Syngnathus floridae* | AF356069 | *CYB* |
| Gasterosteiformes | Syngnathoidei | Syngnathidae | *Syngnathus fuscus* | AF356056 | *CYB* |
| Gasterosteiformes | Syngnathoidei | Syngnathidae | *Syngnathus leptorhynchus* | FJ165395 | *CO1* |
| Gasterosteiformes | Syngnathoidei | Syngnathidae | *Syngnathus leptorhynchus* | AF356064 | *CYB* |
| Gasterosteiformes | Syngnathoidei | Syngnathidae | *Syngnathus louisianae* | AF356070 | *CYB* |
| Gasterosteiformes | Syngnathoidei | Syngnathidae | *Syngnathus rostellatus* | AF356041 | *CYB* |
| Gasterosteiformes | Syngnathoidei | Syngnathidae | *Syngnathus schlegeli* | AF356051 | *CYB* |
| Gasterosteiformes | Syngnathoidei | Syngnathidae | *Syngnathus scovelli* | AF356068 | *CYB* |
| Gasterosteiformes | Syngnathoidei | Syngnathidae | *Syngnathus taenionotus* | AF356061 | *CYB* |
| Gasterosteiformes | Syngnathoidei | Syngnathidae | *Syngnathus temminckii* | FJ905775 | *rag1* |
| Gasterosteiformes | Syngnathoidei | Syngnathidae | *Syngnathus temminckii* | FJ905796 | *rhod* |
| Gasterosteiformes | Syngnathoidei | Syngnathidae | *Syngnathus typhle* | AF356042 | *CYB* |
| Gasterosteiformes | Syngnathoidei | Syngnathidae | *Syngnathus typhle* | AY368326 | *rhod* |
| Gasterosteiformes | Syngnathoidei | Syngnathidae | *Vanacampus margaritifer* | FJ905777 | *rag1* |
| Gasterosteiformes | Syngnathoidei | Syngnathidae | *Vanacampus margaritifer* | FJ905798 | *rhod* |
| Gasterosteiformes | Syngnathoidei | Aulostomidae | *Aulostomus chinensis* | FJ582892 | *CO1* |
| Gasterosteiformes | Syngnathoidei | Aulostomidae | *Aulostomus chinensis* | AY786433 | *CYB* |
| Gasterosteiformes | Syngnathoidei | Aulostomidae | *Aulostomus chinensis* | AY141279 | *rhod* |
| Gasterosteiformes | Syngnathoidei | Aulostomidae | *Aulostomus maculatus* | AF327456 | *CYB* |
| Gasterosteiformes | Syngnathoidei | Aulostomidae | *Aulostomus strigosus* | AF327457 | *CYB* |
| Gasterosteiformes | Syngnathoidei | Fistulariidae | *Fistularia commersonii* | EF607383 | *CO1* |
| Gasterosteiformes | Syngnathoidei | Fistulariidae | *Fistularia commersonii* | AY786435 | *CYB* |
| Gasterosteiformes | Syngnathoidei | Fistulariidae | *Fistularia petimba* | FJ211285 | *CO1* |
| Gasterosteiformes | Syngnathoidei | Fistulariidae | *Fistularia petimba* | AY141324 | *rhod* |
| Gasterosteiformes | Syngnathoidei | Fistulariidae | *Fistularia tabacaria* | JN026615 | *CO1* |
| Gasterosteiformes | Syngnathoidei | Centriscidae | *Aeoliscus strigatus* | AY786434 | *CYB* |
| Gasterosteiformes | Syngnathoidei | Centriscidae | *Aeoliscus strigatus* | EU637931 | *rhod* |
| Gasterosteiformes | Syngnathoidei | Centriscidae | *Centriscus scutatus* | FJ265863 | *CO1* |
| Gasterosteiformes | Syngnathoidei | Centriscidae | *Macroramphosus scolopax* | FN688950 | *CO1* |
| Gasterosteiformes | Syngnathoidei | Centriscidae | *Macroramphosus scolopax* | FN688352 | *CYB* |
| Gasterosteiformes | Syngnathoidei | Centriscidae | *Macroramphosus scolopax* | AY141280 | *rhod* |
| Gasterosteiformes | Syngnathoidei | Centriscidae | *Notopogon fernandezianus* | EU074505 | *CO1* |
| Synbranchiformes | none | Synbranchidae | *Monopterus albus* | AY141276 | *rhod* |
| Synbranchiformes | none | Synbranchidae | *Ophisternon aenigmaticum* | AY355008 | *ATP6* |
| Synbranchiformes | none | Synbranchidae | *Ophisternon aenigmaticum* | EU751861 | *CO1* |
| Synbranchiformes | none | Synbranchidae | *Ophisternon aenigmaticum* | AY355082 | *CYB* |
| Synbranchiformes | none | Synbranchidae | *Synbranchus marmoratus* | AY355014 | *ATP6* |
| Synbranchiformes | none | Synbranchidae | *Synbranchus marmoratus* | AY355104 | *CYB* |
| Synbranchiformes | none | Mastacembelidae | *Macrognathus pancalus* | EU417778 | *CO1* |
| Synbranchiformes | none | Mastacembelidae | *Macrognathus siamensis* | EF609404 | *CO1* |
| Synbranchiformes | none | Mastacembelidae | *Macrognathus siamensis* | AB534718 | *ND2* |
| Synbranchiformes | none | Mastacembelidae | *Macrognathus zebrinus* | FN813113 | *CO1* |
| Synbranchiformes | none | Mastacembelidae | *Macrognathus zebrinus* | FN813033 | *CYB* |
| Synbranchiformes | none | Mastacembelidae | *Mastacembelus albomaculatus* | FN813035 | *CO1* |
| Synbranchiformes | none | Mastacembelidae | *Mastacembelus albomaculatus* | FN812957 | *CYB* |
| Synbranchiformes | none | Mastacembelidae | *Mastacembelus armatus* | FN813112 | *CO1* |
| Synbranchiformes | none | Mastacembelidae | *Mastacembelus armatus* | FN813032 | *CYB* |
| Synbranchiformes | none | Mastacembelidae | *Mastacembelus brachyrhinus* | FN813110 | *CO1* |
| Synbranchiformes | none | Mastacembelidae | *Mastacembelus brachyrhinus* | FN813017 | *CYB* |
| Synbranchiformes | none | Mastacembelidae | *Mastacembelus cryptacanthus* | FN813109 | *CO1* |
| Synbranchiformes | none | Mastacembelidae | *Mastacembelus cryptacanthus* | FN813015 | *CYB* |
| Synbranchiformes | none | Mastacembelidae | *Mastacembelus cunningtoni* | FN813044 | *CO1* |
| Synbranchiformes | none | Mastacembelidae | *Mastacembelus cunningtoni* | FN812962 | *CYB* |
| Synbranchiformes | none | Mastacembelidae | *Mastacembelus ellipsifer* | FN813055 | *CO1* |
| Synbranchiformes | none | Mastacembelidae | *Mastacembelus ellipsifer* | FN812966 | *CYB* |
| Synbranchiformes | none | Mastacembelidae | *Mastacembelus erythrotaenia* | AY141275 | *rhod* |
| Synbranchiformes | none | Mastacembelidae | *Mastacembelus flavidus* | FN813057 | *CO1* |
| Synbranchiformes | none | Mastacembelidae | *Mastacembelus flavidus* | FN812973 | *CYB* |
| Synbranchiformes | none | Mastacembelidae | *Mastacembelus frenatus* | FN813087 | *CO1* |
| Synbranchiformes | none | Mastacembelidae | *Mastacembelus frenatus* | FN813009 | *CYB* |
| Synbranchiformes | none | Mastacembelidae | *Mastacembelus micropectus* | FN813058 | *CO1* |
| Synbranchiformes | none | Mastacembelidae | *Mastacembelus micropectus* | FN812974 | *CYB* |
| Synbranchiformes | none | Mastacembelidae | *Mastacembelus moorii* | FN813062 | *CO1* |
| Synbranchiformes | none | Mastacembelidae | *Mastacembelus moorii* | FN812993 | *CYB* |
| Synbranchiformes | none | Mastacembelidae | *Mastacembelus nigromarginatus* | FN813099 | *CO1* |
| Synbranchiformes | none | Mastacembelidae | *Mastacembelus nigromarginatus* | FN813027 | *CYB* |
| Synbranchiformes | none | Mastacembelidae | *Mastacembelus ophidium* | FN813069 | *CO1* |
| Synbranchiformes | none | Mastacembelidae | *Mastacembelus ophidium* | FN812979 | *CYB* |
| Synbranchiformes | none | Mastacembelidae | *Mastacembelus plagiostomus* | FN813070 | *CO1* |
| Synbranchiformes | none | Mastacembelidae | *Mastacembelus plagiostomus* | FN812984 | *CYB* |
| Synbranchiformes | none | Mastacembelidae | *Mastacembelus platysoma* | FN813076 | *CO1* |
| Synbranchiformes | none | Mastacembelidae | *Mastacembelus platysoma* | FN812988 | *CYB* |
| Synbranchiformes | none | Mastacembelidae | *Mastacembelus shiranus* | FN813092 | *CO1* |
| Synbranchiformes | none | Mastacembelidae | *Mastacembelus shiranus* | FN813007 | *CYB* |
| Synbranchiformes | none | Mastacembelidae | *Mastacembelus signatus* | FN813101 | *CO1* |
| Synbranchiformes | none | Mastacembelidae | *Mastacembelus signatus* | FN813025 | *CYB* |
| Synbranchiformes | none | Mastacembelidae | *Mastacembelus stappersii* | FN813104 | *CO1* |
| Synbranchiformes | none | Mastacembelidae | *Mastacembelus stappersii* | FN813019 | *CYB* |
| Synbranchiformes | none | Mastacembelidae | *Mastacembelus taiaensis* | FN813106 | *CO1* |
| Synbranchiformes | none | Mastacembelidae | *Mastacembelus taiaensis* | FN813013 | *CYB* |
| Synbranchiformes | none | Mastacembelidae | *Mastacembelus tanganicae* | FN813078 | *CO1* |
| Synbranchiformes | none | Mastacembelidae | *Mastacembelus tanganicae* | FN812989 | *CYB* |
| Synbranchiformes | none | Mastacembelidae | *Mastacembelus vanderwaali* | FN813097 | *CO1* |
| Synbranchiformes | none | Mastacembelidae | *Mastacembelus vanderwaali* | FN813030 | *CYB* |
| Synbranchiformes | none | Mastacembelidae | *Mastacembelus zebratus* | FN813071 | *CO1* |
| Synbranchiformes | none | Mastacembelidae | *Mastacembelus zebratus* | FN812990 | *CYB* |
| Scorpaeniformes | Dactylopteroidei | Dactylopteridae | *Dactyloptena orientalis* | EU148590 | *CO1* |
| Scorpaeniformes | Dactylopteroidei | Dactylopteridae | *Dactylopterus volitans* | EF439515 | *CYB* |
| Scorpaeniformes | Dactylopteroidei | Dactylopteridae | *Dactylopterus volitans* | AY141282 | *rhod* |
| Scorpaeniformes | Scorpaenoidei | Scorpaenidae | *Apistus carinatus* | JF952670 | *CO1* |
| Scorpaeniformes | Scorpaenoidei | Scorpaenidae | *Dendrochirus biocellatus* | FJ583342 | *CO1* |
| Scorpaeniformes | Scorpaenoidei | Scorpaenidae | *Dendrochirus biocellatus* | FJ607319 | *CYB* |
| Scorpaeniformes | Scorpaenoidei | Scorpaenidae | *Dendrochirus brachypterus* | FJ583345 | *CO1* |
| Scorpaeniformes | Scorpaenoidei | Scorpaenidae | *Dendrochirus brachypterus* | FJ607323 | *CYB* |
| Scorpaeniformes | Scorpaenoidei | Scorpaenidae | *Dendrochirus zebra* | FJ237721 | *CO1* |
| Scorpaeniformes | Scorpaenoidei | Scorpaenidae | *Dendrochirus zebra* | FJ607324 | *CYB* |
| Scorpaeniformes | Scorpaenoidei | Scorpaenidae | *Helicolenus avius* | DQ678402 | *CO1* |
| Scorpaeniformes | Scorpaenoidei | Scorpaenidae | *Helicolenus avius* | DQ678505 | *CYB* |
| Scorpaeniformes | Scorpaenoidei | Scorpaenidae | *Helicolenus barathri* | EF609370 | *CO1* |
| Scorpaeniformes | Scorpaenoidei | Scorpaenidae | *Helicolenus dactylopterus* | HM007748 | *CO1* |
| Scorpaeniformes | Scorpaenoidei | Scorpaenidae | *Helicolenus dactylopterus* | EU036442 | *CYB* |
| Scorpaeniformes | Scorpaenoidei | Scorpaenidae | *Helicolenus dactylopterus* | HM050111 | *ND4* |
| Scorpaeniformes | Scorpaenoidei | Scorpaenidae | *Helicolenus dactylopterus* | EU224153 | *rhod* |
| Scorpaeniformes | Scorpaenoidei | Scorpaenidae | *Helicolenus dactylopterus dactylopterus* | FN689143 | *CO1* |
| Scorpaeniformes | Scorpaenoidei | Scorpaenidae | *Helicolenus dactylopterus dactylopterus* | EF439532 | *CYB* |
| Scorpaeniformes | Scorpaenoidei | Scorpaenidae | *Helicolenus hilgendorfi* | AB096134 | *CYB* |
| Scorpaeniformes | Scorpaenoidei | Scorpaenidae | *Helicolenus lengerichi* | AY927987 | *CYB* |
| Scorpaeniformes | Scorpaenoidei | Scorpaenidae | *Helicolenus percoides* | DQ108054 | *CO1* |
| Scorpaeniformes | Scorpaenoidei | Scorpaenidae | *Hozukius emblemarius* | DQ678499 | *CYB* |
| Scorpaeniformes | Scorpaenoidei | Scorpaenidae | *Inimicus japonicus* | HM021133 | *CYB* |
| Scorpaeniformes | Scorpaenoidei | Scorpaenidae | *Inimicus sinensis* | GU479051 | *CO1* |
| Scorpaeniformes | Scorpaenoidei | Scorpaenidae | *Minous monodactylus* | EU595221 | *CO1* |
| Scorpaeniformes | Scorpaenoidei | Scorpaenidae | *Neomerinthe hemingwayi* | DQ874787 | *rag2* |
| Scorpaeniformes | Scorpaenoidei | Scorpaenidae | *Neomerinthe hemingwayi* | DQ874819 | *rhod* |
| Scorpaeniformes | Scorpaenoidei | Scorpaenidae | *Neosebastes incisipinnis* | DQ108058 | *CO1* |
| Scorpaeniformes | Scorpaenoidei | Scorpaenidae | *Neosebastes scorpaenoides* | DQ108046 | *CO1* |
| Scorpaeniformes | Scorpaenoidei | Scorpaenidae | *Neosebastes thetidis* | DQ108050 | *CO1* |
| Scorpaeniformes | Scorpaenoidei | Scorpaenidae | *Notesthes robusta* | HM007016 | *ATP6* |
| Scorpaeniformes | Scorpaenoidei | Scorpaenidae | *Notesthes robusta* | HM006975 | *CO1* |
| Scorpaeniformes | Scorpaenoidei | Scorpaenidae | *Notesthes robusta* | HM007057 | *CYB* |
| Scorpaeniformes | Scorpaenoidei | Scorpaenidae | *Pontinus furcirhinus* | AF030712 | *CYB* |
| Scorpaeniformes | Scorpaenoidei | Scorpaenidae | *Pontinus kuhlii* | AF100923 | *CYB* |
| Scorpaeniformes | Scorpaenoidei | Scorpaenidae | *Pontinus kuhlii* | DQ197885 | *rhod* |
| Scorpaeniformes | Scorpaenoidei | Scorpaenidae | *Pontinus longispinis* | EU637996 | *rhod* |
| Scorpaeniformes | Scorpaenoidei | Scorpaenidae | *Pontinus macrocephalus* | DQ521009 | *CO1* |
| Scorpaeniformes | Scorpaenoidei | Scorpaenidae | *Pterois andover* | FJ584016 | *CO1* |
| Scorpaeniformes | Scorpaenoidei | Scorpaenidae | *Pterois antennata* | FJ584017 | *CO1* |
| Scorpaeniformes | Scorpaenoidei | Scorpaenidae | *Pterois antennata* | FJ607314 | *CYB* |
| Scorpaeniformes | Scorpaenoidei | Scorpaenidae | *Pterois lunulata* | EU167898 | *rag1* |
| Scorpaeniformes | Scorpaenoidei | Scorpaenidae | *Pterois miles* | EU148592 | *CO1* |
| Scorpaeniformes | Scorpaenoidei | Scorpaenidae | *Pterois miles* | EF209675 | *CYB* |
| Scorpaeniformes | Scorpaenoidei | Scorpaenidae | *Pterois mombasae* | FJ584039 | *CO1* |
| Scorpaeniformes | Scorpaenoidei | Scorpaenidae | *Pterois mombasae* | AJ429428 | *CYB* |
| Scorpaeniformes | Scorpaenoidei | Scorpaenidae | *Pterois radiata* | FJ607318 | *CYB* |
| Scorpaeniformes | Scorpaenoidei | Scorpaenidae | *Pterois russelii* | FJ237902 | *CO1* |
| Scorpaeniformes | Scorpaenoidei | Scorpaenidae | *Pterois volitans* | FJ584044 | *CO1* |
| Scorpaeniformes | Scorpaenoidei | Scorpaenidae | *Pterois volitans* | DQ482583 | *CYB* |
| Scorpaeniformes | Scorpaenoidei | Scorpaenidae | *Scorpaena brasiliensis* | FJ584091 | *CO1* |
| Scorpaeniformes | Scorpaenoidei | Scorpaenidae | *Scorpaena cardinalis* | DQ108051 | *CO1* |
| Scorpaeniformes | Scorpaenoidei | Scorpaenidae | *Scorpaena colorata* | DQ521012 | *CO1* |
| Scorpaeniformes | Scorpaenoidei | Scorpaenidae | *Scorpaena elongata* | EF456020 | *CYB* |
| Scorpaeniformes | Scorpaenoidei | Scorpaenidae | *Scorpaena elongata* | EF456080 | *rhod* |
| Scorpaeniformes | Scorpaenoidei | Scorpaenidae | *Scorpaena grandicornis* | GU224589 | *CO1* |
| Scorpaeniformes | Scorpaenoidei | Scorpaenidae | *Scorpaena guttata* | GU440516 | *CO1* |
| Scorpaeniformes | Scorpaenoidei | Scorpaenidae | *Scorpaena guttata* | AF030711 | *CYB* |
| Scorpaeniformes | Scorpaenoidei | Scorpaenidae | *Scorpaena inermis* | GU225030 | *CO1* |
| Scorpaeniformes | Scorpaenoidei | Scorpaenidae | *Scorpaena maderensis* | DQ197898 | *rhod* |
| Scorpaeniformes | Scorpaenoidei | Scorpaenidae | *Scorpaena notata* | FN688967 | *CO1* |
| Scorpaeniformes | Scorpaenoidei | Scorpaenidae | *Scorpaena notata* | FN688440 | *CYB* |
| Scorpaeniformes | Scorpaenoidei | Scorpaenidae | *Scorpaena notata* | DQ197899 | *rhod* |
| Scorpaeniformes | Scorpaenoidei | Scorpaenidae | *Scorpaena onaria* | EF095642 | *rag1* |
| Scorpaeniformes | Scorpaenoidei | Scorpaenidae | *Scorpaena onaria* | AY141288 | *rhod* |
| Scorpaeniformes | Scorpaenoidei | Scorpaenidae | *Scorpaena porcus* | EF427596 | *CYB* |
| Scorpaeniformes | Scorpaenoidei | Scorpaenidae | *Scorpaena porcus* | EF439437 | *rhod* |
| Scorpaeniformes | Scorpaenoidei | Scorpaenidae | *Scorpaena scrofa* | HQ945856 | *CO1* |
| Scorpaeniformes | Scorpaenoidei | Scorpaenidae | *Scorpaena scrofa* | EU036495 | *CYB* |
| Scorpaeniformes | Scorpaenoidei | Scorpaenidae | *Scorpaena scrofa* | EF439443 | *rhod* |
| Scorpaeniformes | Scorpaenoidei | Scorpaenidae | *Scorpaenodes guamensis* | HQ945882 | *CO1* |
| Scorpaeniformes | Scorpaenoidei | Scorpaenidae | *Scorpaenodes xyris* | GU440518 | *CO1* |
| Scorpaeniformes | Scorpaenoidei | Scorpaenidae | *Scorpaenopsis cirrosa* | JF952853 | *CO1* |
| Scorpaeniformes | Scorpaenoidei | Scorpaenidae | *Scorpaenopsis cirrosa* | AB063195 | *ND2* |
| Scorpaeniformes | Scorpaenoidei | Scorpaenidae | *Scorpaenopsis diabolus* | FJ918952 | *CO1* |
| Scorpaeniformes | Scorpaenoidei | Scorpaenidae | *Scorpaenopsis diabolus* | FJ896468 | *rag1* |
| Scorpaeniformes | Scorpaenoidei | Scorpaenidae | *Scorpaenopsis vittapinna* | EF607539 | *CO1* |
| Scorpaeniformes | Scorpaenoidei | Scorpaenidae | *Sebastapistes strongia* | HQ561462 | *CO1* |
| Scorpaeniformes | Scorpaenoidei | Scorpaenidae | *Sebastes aleutianus* | DQ678315 | *CO1* |
| Scorpaeniformes | Scorpaenoidei | Scorpaenidae | *Sebastes aleutianus* | DQ678418 | *CYB* |
| Scorpaeniformes | Scorpaenoidei | Scorpaenidae | *Sebastes alutus* | DQ678313 | *CO1* |
| Scorpaeniformes | Scorpaenoidei | Scorpaenidae | *Sebastes alutus* | DQ678416 | *CYB* |
| Scorpaeniformes | Scorpaenoidei | Scorpaenidae | *Sebastes alutus* | EF212407 | *rhod* |
| Scorpaeniformes | Scorpaenoidei | Scorpaenidae | *Sebastes atrovirens* | DQ678320 | *CO1* |
| Scorpaeniformes | Scorpaenoidei | Scorpaenidae | *Sebastes atrovirens* | DQ678423 | *CYB* |
| Scorpaeniformes | Scorpaenoidei | Scorpaenidae | *Sebastes auriculatus* | DQ678410 | *CO1* |
| Scorpaeniformes | Scorpaenoidei | Scorpaenidae | *Sebastes auriculatus* | DQ678513 | *CYB* |
| Scorpaeniformes | Scorpaenoidei | Scorpaenidae | *Sebastes auriculatus* | EF212408 | *rhod* |
| Scorpaeniformes | Scorpaenoidei | Scorpaenidae | *Sebastes aurora* | DQ678314 | *CO1* |
| Scorpaeniformes | Scorpaenoidei | Scorpaenidae | *Sebastes aurora* | DQ678417 | *CYB* |
| Scorpaeniformes | Scorpaenoidei | Scorpaenidae | *Sebastes aurora* | EF212409 | *rhod* |
| Scorpaeniformes | Scorpaenoidei | Scorpaenidae | *Sebastes babcocki* | DQ678319 | *CO1* |
| Scorpaeniformes | Scorpaenoidei | Scorpaenidae | *Sebastes babcocki* | DQ678422 | *CYB* |
| Scorpaeniformes | Scorpaenoidei | Scorpaenidae | *Sebastes baramenuke* | DQ678388 | *CO1* |
| Scorpaeniformes | Scorpaenoidei | Scorpaenidae | *Sebastes baramenuke* | DQ678491 | *CYB* |
| Scorpaeniformes | Scorpaenoidei | Scorpaenidae | *Sebastes borealis* | DQ678403 | *CO1* |
| Scorpaeniformes | Scorpaenoidei | Scorpaenidae | *Sebastes borealis* | DQ678506 | *CYB* |
| Scorpaeniformes | Scorpaenoidei | Scorpaenidae | *Sebastes brevispinis* | DQ678316 | *CO1* |
| Scorpaeniformes | Scorpaenoidei | Scorpaenidae | *Sebastes brevispinis* | DQ678419 | *CYB* |
| Scorpaeniformes | Scorpaenoidei | Scorpaenidae | *Sebastes brevispinis* | EF212410 | *rhod* |
| Scorpaeniformes | Scorpaenoidei | Scorpaenidae | *Sebastes capensis* | DQ678317 | *CO1* |
| Scorpaeniformes | Scorpaenoidei | Scorpaenidae | *Sebastes capensis* | AF031503 | *CYB* |
| Scorpaeniformes | Scorpaenoidei | Scorpaenidae | *Sebastes carnatus* | DQ678321 | *CO1* |
| Scorpaeniformes | Scorpaenoidei | Scorpaenidae | *Sebastes carnatus* | DQ678424 | *CYB* |
| Scorpaeniformes | Scorpaenoidei | Scorpaenidae | *Sebastes carnatus* | EF212411 | *rhod* |
| Scorpaeniformes | Scorpaenoidei | Scorpaenidae | *Sebastes caurinus* | DQ678322 | *CO1* |
| Scorpaeniformes | Scorpaenoidei | Scorpaenidae | *Sebastes caurinus* | DQ678425 | *CYB* |
| Scorpaeniformes | Scorpaenoidei | Scorpaenidae | *Sebastes chlorostictus* | DQ678332 | *CO1* |
| Scorpaeniformes | Scorpaenoidei | Scorpaenidae | *Sebastes chlorostictus* | AF031504 | *CYB* |
| Scorpaeniformes | Scorpaenoidei | Scorpaenidae | *Sebastes chlorostictus* | EF212413 | *rhod* |
| Scorpaeniformes | Scorpaenoidei | Scorpaenidae | *Sebastes chrysomelas* | DQ678323 | *CO1* |
| Scorpaeniformes | Scorpaenoidei | Scorpaenidae | *Sebastes chrysomelas* | DQ678426 | *CYB* |
| Scorpaeniformes | Scorpaenoidei | Scorpaenidae | *Sebastes ciliatus* | DQ678412 | *CO1* |
| Scorpaeniformes | Scorpaenoidei | Scorpaenidae | *Sebastes ciliatus* | DQ678515 | *CYB* |
| Scorpaeniformes | Scorpaenoidei | Scorpaenidae | *Sebastes ciliatus* | EF212415 | *rhod* |
| Scorpaeniformes | Scorpaenoidei | Scorpaenidae | *Sebastes constellatus* | DQ678333 | *CO1* |
| Scorpaeniformes | Scorpaenoidei | Scorpaenidae | *Sebastes constellatus* | DQ678436 | *CYB* |
| Scorpaeniformes | Scorpaenoidei | Scorpaenidae | *Sebastes constellatus* | EF212416 | *rhod* |
| Scorpaeniformes | Scorpaenoidei | Scorpaenidae | *Sebastes cortezi* | DQ678394 | *CO1* |
| Scorpaeniformes | Scorpaenoidei | Scorpaenidae | *Sebastes cortezi* | DQ678497 | *CYB* |
| Scorpaeniformes | Scorpaenoidei | Scorpaenidae | *Sebastes crameri* | DQ678334 | *CO1* |
| Scorpaeniformes | Scorpaenoidei | Scorpaenidae | *Sebastes crameri* | DQ678437 | *CYB* |
| Scorpaeniformes | Scorpaenoidei | Scorpaenidae | *Sebastes dallii* | DQ678324 | *CO1* |
| Scorpaeniformes | Scorpaenoidei | Scorpaenidae | *Sebastes dallii* | DQ678427 | *CYB* |
| Scorpaeniformes | Scorpaenoidei | Scorpaenidae | *Sebastes diploproa* | DQ678335 | *CO1* |
| Scorpaeniformes | Scorpaenoidei | Scorpaenidae | *Sebastes diploproa* | DQ678438 | *CYB* |
| Scorpaeniformes | Scorpaenoidei | Scorpaenidae | *Sebastes diploproa* | EF212417 | *rhod* |
| Scorpaeniformes | Scorpaenoidei | Scorpaenidae | *Sebastes elongatus* | DQ678331 | *CO1* |
| Scorpaeniformes | Scorpaenoidei | Scorpaenidae | *Sebastes elongatus* | DQ678434 | *CYB* |
| Scorpaeniformes | Scorpaenoidei | Scorpaenidae | *Sebastes elongatus* | EF212418 | *rhod* |
| Scorpaeniformes | Scorpaenoidei | Scorpaenidae | *Sebastes emphaeus* | DQ678336 | *CO1* |
| Scorpaeniformes | Scorpaenoidei | Scorpaenidae | *Sebastes emphaeus* | DQ678439 | *CYB* |
| Scorpaeniformes | Scorpaenoidei | Scorpaenidae | *Sebastes ensifer* | DQ678337 | *CO1* |
| Scorpaeniformes | Scorpaenoidei | Scorpaenidae | *Sebastes ensifer* | AF031507 | *CYB* |
| Scorpaeniformes | Scorpaenoidei | Scorpaenidae | *Sebastes entomelas* | DQ678338 | *CO1* |
| Scorpaeniformes | Scorpaenoidei | Scorpaenidae | *Sebastes entomelas* | DQ678441 | *CYB* |
| Scorpaeniformes | Scorpaenoidei | Scorpaenidae | *Sebastes entomelas* | EF212419 | *rhod* |
| Scorpaeniformes | Scorpaenoidei | Scorpaenidae | *Sebastes entomelus* | EU548255 | *CYB* |
| Scorpaeniformes | Scorpaenoidei | Scorpaenidae | *Sebastes eos* | DQ678339 | *CO1* |
| Scorpaeniformes | Scorpaenoidei | Scorpaenidae | *Sebastes eos* | DQ678442 | *CYB* |
| Scorpaeniformes | Scorpaenoidei | Scorpaenidae | *Sebastes exsul* | DQ678340 | *CO1* |
| Scorpaeniformes | Scorpaenoidei | Scorpaenidae | *Sebastes exsul* | AF031514 | *CYB* |
| Scorpaeniformes | Scorpaenoidei | Scorpaenidae | *Sebastes fasciatus* | DQ678341 | *CO1* |
| Scorpaeniformes | Scorpaenoidei | Scorpaenidae | *Sebastes fasciatus* | DQ678444 | *CYB* |
| Scorpaeniformes | Scorpaenoidei | Scorpaenidae | *Sebastes flammeus* | DQ678383 | *CO1* |
| Scorpaeniformes | Scorpaenoidei | Scorpaenidae | *Sebastes flammeus* | DQ678486 | *CYB* |
| Scorpaeniformes | Scorpaenoidei | Scorpaenidae | *Sebastes flavidus* | DQ678342 | *CO1* |
| Scorpaeniformes | Scorpaenoidei | Scorpaenidae | *Sebastes flavidus* | DQ678445 | *CYB* |
| Scorpaeniformes | Scorpaenoidei | Scorpaenidae | *Sebastes flavidus* | EF212420 | *rhod* |
| Scorpaeniformes | Scorpaenoidei | Scorpaenidae | *Sebastes gilli* | DQ678343 | *CO1* |
| Scorpaeniformes | Scorpaenoidei | Scorpaenidae | *Sebastes gilli* | DQ678446 | *CYB* |
| Scorpaeniformes | Scorpaenoidei | Scorpaenidae | *Sebastes glaucus* | DQ678385 | *CO1* |
| Scorpaeniformes | Scorpaenoidei | Scorpaenidae | *Sebastes glaucus* | DQ678488 | *CYB* |
| Scorpaeniformes | Scorpaenoidei | Scorpaenidae | *Sebastes goodei* | DQ678358 | *CO1* |
| Scorpaeniformes | Scorpaenoidei | Scorpaenidae | *Sebastes goodei* | DQ678461 | *CYB* |
| Scorpaeniformes | Scorpaenoidei | Scorpaenidae | *Sebastes helvomaculatus* | DQ678393 | *CO1* |
| Scorpaeniformes | Scorpaenoidei | Scorpaenidae | *Sebastes helvomaculatus* | AF031508 | *CYB* |
| Scorpaeniformes | Scorpaenoidei | Scorpaenidae | *Sebastes hopkinsi* | DQ678344 | *CO1* |
| Scorpaeniformes | Scorpaenoidei | Scorpaenidae | *Sebastes hopkinsi* | DQ678447 | *CYB* |
| Scorpaeniformes | Scorpaenoidei | Scorpaenidae | *Sebastes hubbsi* | DQ678398 | *CO1* |
| Scorpaeniformes | Scorpaenoidei | Scorpaenidae | *Sebastes hubbsi* | DQ678501 | *CYB* |
| Scorpaeniformes | Scorpaenoidei | Scorpaenidae | *Sebastes ijimae* | AB096120 | *CYB* |
| Scorpaeniformes | Scorpaenoidei | Scorpaenidae | *Sebastes inermis* | DQ678380 | *CO1* |
| Scorpaeniformes | Scorpaenoidei | Scorpaenidae | *Sebastes inermis* | DQ678483 | *CYB* |
| Scorpaeniformes | Scorpaenoidei | Scorpaenidae | *Sebastes iracundus* | DQ678392 | *CO1* |
| Scorpaeniformes | Scorpaenoidei | Scorpaenidae | *Sebastes iracundus* | DQ678495 | *CYB* |
| Scorpaeniformes | Scorpaenoidei | Scorpaenidae | *Sebastes jordani* | DQ678345 | *CO1* |
| Scorpaeniformes | Scorpaenoidei | Scorpaenidae | *Sebastes jordani* | DQ678448 | *CYB* |
| Scorpaeniformes | Scorpaenoidei | Scorpaenidae | *Sebastes joyneri* | DQ678386 | *CO1* |
| Scorpaeniformes | Scorpaenoidei | Scorpaenidae | *Sebastes joyneri* | DQ678489 | *CYB* |
| Scorpaeniformes | Scorpaenoidei | Scorpaenidae | *Sebastes kiyomatsui* | DQ678384 | *CO1* |
| Scorpaeniformes | Scorpaenoidei | Scorpaenidae | *Sebastes kiyomatsui* | DQ678487 | *CYB* |
| Scorpaeniformes | Scorpaenoidei | Scorpaenidae | *Sebastes koreanus* | JN157872 | *CO1* |
| Scorpaeniformes | Scorpaenoidei | Scorpaenidae | *Sebastes koreanus* | AB188095 | *CYB* |
| Scorpaeniformes | Scorpaenoidei | Scorpaenidae | *Sebastes lentiginosus* | DQ678346 | *CO1* |
| Scorpaeniformes | Scorpaenoidei | Scorpaenidae | *Sebastes lentiginosus* | DQ678449 | *CYB* |
| Scorpaeniformes | Scorpaenoidei | Scorpaenidae | *Sebastes levis* | DQ678347 | *CO1* |
| Scorpaeniformes | Scorpaenoidei | Scorpaenidae | *Sebastes levis* | DQ678450 | *CYB* |
| Scorpaeniformes | Scorpaenoidei | Scorpaenidae | *Sebastes longispinis* | AB188096 | *CYB* |
| Scorpaeniformes | Scorpaenoidei | Scorpaenidae | *Sebastes macdonaldi* | DQ678348 | *CO1* |
| Scorpaeniformes | Scorpaenoidei | Scorpaenidae | *Sebastes macdonaldi* | DQ678451 | *CYB* |
| Scorpaeniformes | Scorpaenoidei | Scorpaenidae | *Sebastes maliger* | DQ678325 | *CO1* |
| Scorpaeniformes | Scorpaenoidei | Scorpaenidae | *Sebastes maliger* | AF031500 | *CYB* |
| Scorpaeniformes | Scorpaenoidei | Scorpaenidae | *Sebastes maliger* | EF212422 | *rhod* |
| Scorpaeniformes | Scorpaenoidei | Scorpaenidae | *Sebastes marinus* | EF456022 | *CYB* |
| Scorpaeniformes | Scorpaenoidei | Scorpaenidae | *Sebastes marinus* | HM050137 | *ND4* |
| Scorpaeniformes | Scorpaenoidei | Scorpaenidae | *Sebastes matsubarae* | DQ678395 | *CO1* |
| Scorpaeniformes | Scorpaenoidei | Scorpaenidae | *Sebastes matsubarae* | AB126390 | *CYB* |
| Scorpaeniformes | Scorpaenoidei | Scorpaenidae | *Sebastes melanops* | DQ678350 | *CO1* |
| Scorpaeniformes | Scorpaenoidei | Scorpaenidae | *Sebastes melanops* | DQ678453 | *CYB* |
| Scorpaeniformes | Scorpaenoidei | Scorpaenidae | *Sebastes melanops* | EF212423 | *rhod* |
| Scorpaeniformes | Scorpaenoidei | Scorpaenidae | *Sebastes melanosema* | DQ678411 | *CO1* |
| Scorpaeniformes | Scorpaenoidei | Scorpaenidae | *Sebastes melanosema* | DQ678514 | *CYB* |
| Scorpaeniformes | Scorpaenoidei | Scorpaenidae | *Sebastes melanostictus* | DQ678311 | *CO1* |
| Scorpaeniformes | Scorpaenoidei | Scorpaenidae | *Sebastes melanostictus* | DQ678414 | *CYB* |
| Scorpaeniformes | Scorpaenoidei | Scorpaenidae | *Sebastes melanostomus* | DQ678351 | *CO1* |
| Scorpaeniformes | Scorpaenoidei | Scorpaenidae | *Sebastes melanostomus* | DQ678454 | *CYB* |
| Scorpaeniformes | Scorpaenoidei | Scorpaenidae | *Sebastes melanostomus* | EF212424 | *rhod* |
| Scorpaeniformes | Scorpaenoidei | Scorpaenidae | *Sebastes mentella* | DQ678352 | *CO1* |
| Scorpaeniformes | Scorpaenoidei | Scorpaenidae | *Sebastes mentella* | EF456023 | *CYB* |
| Scorpaeniformes | Scorpaenoidei | Scorpaenidae | *Sebastes miniatus* | DQ678354 | *CO1* |
| Scorpaeniformes | Scorpaenoidei | Scorpaenidae | *Sebastes miniatus* | DQ678457 | *CYB* |
| Scorpaeniformes | Scorpaenoidei | Scorpaenidae | *Sebastes miniatus* | EF212425 | *rhod* |
| Scorpaeniformes | Scorpaenoidei | Scorpaenidae | *Sebastes minor* | DQ678399 | *CO1* |
| Scorpaeniformes | Scorpaenoidei | Scorpaenidae | *Sebastes minor* | DQ678502 | *CYB* |
| Scorpaeniformes | Scorpaenoidei | Scorpaenidae | *Sebastes moseri* | DQ678406 | *CO1* |
| Scorpaeniformes | Scorpaenoidei | Scorpaenidae | *Sebastes moseri* | DQ678509 | *CYB* |
| Scorpaeniformes | Scorpaenoidei | Scorpaenidae | *Sebastes mystinus* | DQ678355 | *CO1* |
| Scorpaeniformes | Scorpaenoidei | Scorpaenidae | *Sebastes mystinus* | DQ678458 | *CYB* |
| Scorpaeniformes | Scorpaenoidei | Scorpaenidae | *Sebastes mystinus* | EF212426 | *rhod* |
| Scorpaeniformes | Scorpaenoidei | Scorpaenidae | *Sebastes nebulosus* | DQ678326 | *CO1* |
| Scorpaeniformes | Scorpaenoidei | Scorpaenidae | *Sebastes nebulosus* | DQ678429 | *CYB* |
| Scorpaeniformes | Scorpaenoidei | Scorpaenidae | *Sebastes nebulosus* | EF212427 | *rhod* |
| Scorpaeniformes | Scorpaenoidei | Scorpaenidae | *Sebastes nigrocinctus* | DQ678356 | *CO1* |
| Scorpaeniformes | Scorpaenoidei | Scorpaenidae | *Sebastes nigrocinctus* | DQ678459 | *CYB* |
| Scorpaeniformes | Scorpaenoidei | Scorpaenidae | *Sebastes nivosus* | AB188098 | *CYB* |
| Scorpaeniformes | Scorpaenoidei | Scorpaenidae | *Sebastes norvegicus* | DQ678349 | *CO1* |
| Scorpaeniformes | Scorpaenoidei | Scorpaenidae | *Sebastes norvegicus* | DQ678452 | *CYB* |
| Scorpaeniformes | Scorpaenoidei | Scorpaenidae | *Sebastes notius* | DQ678357 | *CO1* |
| Scorpaeniformes | Scorpaenoidei | Scorpaenidae | *Sebastes notius* | DQ678460 | *CYB* |
| Scorpaeniformes | Scorpaenoidei | Scorpaenidae | *Sebastes oblongus* | DQ678400 | *CO1* |
| Scorpaeniformes | Scorpaenoidei | Scorpaenidae | *Sebastes oblongus* | DQ678503 | *CYB* |
| Scorpaeniformes | Scorpaenoidei | Scorpaenidae | *Sebastes oculatus* | DQ678318 | *CO1* |
| Scorpaeniformes | Scorpaenoidei | Scorpaenidae | *Sebastes oculatus* | DQ678421 | *CYB* |
| Scorpaeniformes | Scorpaenoidei | Scorpaenidae | *Sebastes ovalis* | DQ678328 | *CO1* |
| Scorpaeniformes | Scorpaenoidei | Scorpaenidae | *Sebastes ovalis* | DQ678431 | *CYB* |
| Scorpaeniformes | Scorpaenoidei | Scorpaenidae | *Sebastes owstoni* | DQ678390 | *CO1* |
| Scorpaeniformes | Scorpaenoidei | Scorpaenidae | *Sebastes owstoni* | DQ678493 | *CYB* |
| Scorpaeniformes | Scorpaenoidei | Scorpaenidae | *Sebastes pachycephalus* | DQ678387 | *CO1* |
| Scorpaeniformes | Scorpaenoidei | Scorpaenidae | *Sebastes pachycephalus* | DQ678490 | *CYB* |
| Scorpaeniformes | Scorpaenoidei | Scorpaenidae | *Sebastes pachycephalus chalcogrammus* | AB096125 | *CYB* |
| Scorpaeniformes | Scorpaenoidei | Scorpaenidae | *Sebastes paucispinis* | DQ678359 | *CO1* |
| Scorpaeniformes | Scorpaenoidei | Scorpaenidae | *Sebastes paucispinis* | AF031499 | *CYB* |
| Scorpaeniformes | Scorpaenoidei | Scorpaenidae | *Sebastes paucispinis* | EF212430 | *rhod* |
| Scorpaeniformes | Scorpaenoidei | Scorpaenidae | *Sebastes peduncularis* | DQ678401 | *CO1* |
| Scorpaeniformes | Scorpaenoidei | Scorpaenidae | *Sebastes phillipsi* | DQ678360 | *CO1* |
| Scorpaeniformes | Scorpaenoidei | Scorpaenidae | *Sebastes phillipsi* | DQ678463 | *CYB* |
| Scorpaeniformes | Scorpaenoidei | Scorpaenidae | *Sebastes pinniger* | DQ678361 | *CO1* |
| Scorpaeniformes | Scorpaenoidei | Scorpaenidae | *Sebastes pinniger* | DQ678464 | *CYB* |
| Scorpaeniformes | Scorpaenoidei | Scorpaenidae | *Sebastes pinniger* | EF212431 | *rhod* |
| Scorpaeniformes | Scorpaenoidei | Scorpaenidae | *Sebastes polyspinis* | DQ678409 | *CO1* |
| Scorpaeniformes | Scorpaenoidei | Scorpaenidae | *Sebastes polyspinis* | DQ678512 | *CYB* |
| Scorpaeniformes | Scorpaenoidei | Scorpaenidae | *Sebastes proriger* | DQ678362 | *CO1* |
| Scorpaeniformes | Scorpaenoidei | Scorpaenidae | *Sebastes proriger* | DQ678465 | *CYB* |
| Scorpaeniformes | Scorpaenoidei | Scorpaenidae | *Sebastes proriger* | EF212432 | *rhod* |
| Scorpaeniformes | Scorpaenoidei | Scorpaenidae | *Sebastes rastrelliger* | DQ678327 | *CO1* |
| Scorpaeniformes | Scorpaenoidei | Scorpaenidae | *Sebastes rastrelliger* | DQ678430 | *CYB* |
| Scorpaeniformes | Scorpaenoidei | Scorpaenidae | *Sebastes reedi* | DQ678415 | *CYB* |
| Scorpaeniformes | Scorpaenoidei | Scorpaenidae | *Sebastes rosaceus* | DQ678363 | *CO1* |
| Scorpaeniformes | Scorpaenoidei | Scorpaenidae | *Sebastes rosaceus* | AF031512 | *CYB* |
| Scorpaeniformes | Scorpaenoidei | Scorpaenidae | *Sebastes rosenblatti* | AF031511 | *CYB* |
| Scorpaeniformes | Scorpaenoidei | Scorpaenidae | *Sebastes ruberrimus* | DQ678365 | *CO1* |
| Scorpaeniformes | Scorpaenoidei | Scorpaenidae | *Sebastes ruberrimus* | DQ678468 | *CYB* |
| Scorpaeniformes | Scorpaenoidei | Scorpaenidae | *Sebastes ruberrimus* | EF212434 | *rhod* |
| Scorpaeniformes | Scorpaenoidei | Scorpaenidae | *Sebastes rubrivinctus* | DQ678366 | *CO1* |
| Scorpaeniformes | Scorpaenoidei | Scorpaenidae | *Sebastes rubrivinctus* | DQ678469 | *CYB* |
| Scorpaeniformes | Scorpaenoidei | Scorpaenidae | *Sebastes rufinanus* | DQ678405 | *CO1* |
| Scorpaeniformes | Scorpaenoidei | Scorpaenidae | *Sebastes rufinanus* | DQ678508 | *CYB* |
| Scorpaeniformes | Scorpaenoidei | Scorpaenidae | *Sebastes rufus* | DQ678367 | *CO1* |
| Scorpaeniformes | Scorpaenoidei | Scorpaenidae | *Sebastes rufus* | DQ678470 | *CYB* |
| Scorpaeniformes | Scorpaenoidei | Scorpaenidae | *Sebastes saxicola* | DQ678330 | *CO1* |
| Scorpaeniformes | Scorpaenoidei | Scorpaenidae | *Sebastes saxicola* | DQ678433 | *CYB* |
| Scorpaeniformes | Scorpaenoidei | Scorpaenidae | *Sebastes schlegelii* | DQ678378 | *CO1* |
| Scorpaeniformes | Scorpaenoidei | Scorpaenidae | *Sebastes schlegelii* | DQ678481 | *CYB* |
| Scorpaeniformes | Scorpaenoidei | Scorpaenidae | *Sebastes scythropus* | DQ678382 | *CO1* |
| Scorpaeniformes | Scorpaenoidei | Scorpaenidae | *Sebastes scythropus* | AB126389 | *CYB* |
| Scorpaeniformes | Scorpaenoidei | Scorpaenidae | *Sebastes semicinctus* | DQ678368 | *CO1* |
| Scorpaeniformes | Scorpaenoidei | Scorpaenidae | *Sebastes semicinctus* | DQ678471 | *CYB* |
| Scorpaeniformes | Scorpaenoidei | Scorpaenidae | *Sebastes serranoides* | DQ678369 | *CO1* |
| Scorpaeniformes | Scorpaenoidei | Scorpaenidae | *Sebastes serranoides* | DQ678472 | *CYB* |
| Scorpaeniformes | Scorpaenoidei | Scorpaenidae | *Sebastes serriceps* | DQ678370 | *CO1* |
| Scorpaeniformes | Scorpaenoidei | Scorpaenidae | *Sebastes serriceps* | DQ678473 | *CYB* |
| Scorpaeniformes | Scorpaenoidei | Scorpaenidae | *Sebastes simulator* | DQ678404 | *CO1* |
| Scorpaeniformes | Scorpaenoidei | Scorpaenidae | *Sebastes simulator* | DQ678507 | *CYB* |
| Scorpaeniformes | Scorpaenoidei | Scorpaenidae | *Sebastes sinensis* | DQ678408 | *CO1* |
| Scorpaeniformes | Scorpaenoidei | Scorpaenidae | *Sebastes sinensis* | DQ678511 | *CYB* |
| Scorpaeniformes | Scorpaenoidei | Scorpaenidae | *Sebastes spinorbis* | DQ678371 | *CO1* |
| Scorpaeniformes | Scorpaenoidei | Scorpaenidae | *Sebastes spinorbis* | AF031515 | *CYB* |
| Scorpaeniformes | Scorpaenoidei | Scorpaenidae | *Sebastes steindachneri* | DQ678381 | *CO1* |
| Scorpaeniformes | Scorpaenoidei | Scorpaenidae | *Sebastes steindachneri* | DQ678484 | *CYB* |
| Scorpaeniformes | Scorpaenoidei | Scorpaenidae | *Sebastes taczanowskii* | DQ678391 | *CO1* |
| Scorpaeniformes | Scorpaenoidei | Scorpaenidae | *Sebastes taczanowskii* | DQ678494 | *CYB* |
| Scorpaeniformes | Scorpaenoidei | Scorpaenidae | *Sebastes thompsoni* | DQ678379 | *CO1* |
| Scorpaeniformes | Scorpaenoidei | Scorpaenidae | *Sebastes thompsoni* | DQ678482 | *CYB* |
| Scorpaeniformes | Scorpaenoidei | Scorpaenidae | *Sebastes trivittatus* | DQ678389 | *CO1* |
| Scorpaeniformes | Scorpaenoidei | Scorpaenidae | *Sebastes trivittatus* | DQ678492 | *CYB* |
| Scorpaeniformes | Scorpaenoidei | Scorpaenidae | *Sebastes umbrosus* | DQ678372 | *CO1* |
| Scorpaeniformes | Scorpaenoidei | Scorpaenidae | *Sebastes umbrosus* | AF031516 | *CYB* |
| Scorpaeniformes | Scorpaenoidei | Scorpaenidae | *Sebastes variabilis* | DQ678407 | *CO1* |
| Scorpaeniformes | Scorpaenoidei | Scorpaenidae | *Sebastes variabilis* | DQ678510 | *CYB* |
| Scorpaeniformes | Scorpaenoidei | Scorpaenidae | *Sebastes variegatus* | DQ678373 | *CO1* |
| Scorpaeniformes | Scorpaenoidei | Scorpaenidae | *Sebastes variegatus* | DQ678476 | *CYB* |
| Scorpaeniformes | Scorpaenoidei | Scorpaenidae | *Sebastes viviparus* | DQ678374 | *CO1* |
| Scorpaeniformes | Scorpaenoidei | Scorpaenidae | *Sebastes viviparus* | EU492275 | *CYB* |
| Scorpaeniformes | Scorpaenoidei | Scorpaenidae | *Sebastes viviparus* | EU492182 | *rhod* |
| Scorpaeniformes | Scorpaenoidei | Scorpaenidae | *Sebastes vulpes* | DQ678375 | *CO1* |
| Scorpaeniformes | Scorpaenoidei | Scorpaenidae | *Sebastes vulpes* | DQ678478 | *CYB* |
| Scorpaeniformes | Scorpaenoidei | Scorpaenidae | *Sebastes wilsoni* | DQ678376 | *CO1* |
| Scorpaeniformes | Scorpaenoidei | Scorpaenidae | *Sebastes wilsoni* | DQ678479 | *CYB* |
| Scorpaeniformes | Scorpaenoidei | Scorpaenidae | *Sebastes zacentrus* | DQ678377 | *CO1* |
| Scorpaeniformes | Scorpaenoidei | Scorpaenidae | *Sebastes zacentrus* | DQ678480 | *CYB* |
| Scorpaeniformes | Scorpaenoidei | Scorpaenidae | *Sebastes zonatus* | AB096121 | *CYB* |
| Scorpaeniformes | Scorpaenoidei | Scorpaenidae | *Sebastiscus albofasciatus* | AF030768 | *CYB* |
| Scorpaeniformes | Scorpaenoidei | Scorpaenidae | *Sebastiscus marmoratus* | DQ678413 | *CO1* |
| Scorpaeniformes | Scorpaenoidei | Scorpaenidae | *Sebastiscus marmoratus* | DQ678516 | *CYB* |
| Scorpaeniformes | Scorpaenoidei | Scorpaenidae | *Sebastiscus marmoratus* | AB063196 | *ND2* |
| Scorpaeniformes | Scorpaenoidei | Scorpaenidae | *Sebastolobus alascanus* | DQ678397 | *CO1* |
| Scorpaeniformes | Scorpaenoidei | Scorpaenidae | *Sebastolobus alascanus* | AF031497 | *CYB* |
| Scorpaeniformes | Scorpaenoidei | Scorpaenidae | *Sebastolobus altivelis* | FJ165356 | *CO1* |
| Scorpaeniformes | Scorpaenoidei | Scorpaenidae | *Sebastolobus altivelis* | DQ490124 | *rhod* |
| Scorpaeniformes | Scorpaenoidei | Scorpaenidae | *Sebastolobus macrochir* | JF952856 | *CO1* |
| Scorpaeniformes | Scorpaenoidei | Scorpaenidae | *Sebastolobus macrochir* | AB114906 | *CYB* |
| Scorpaeniformes | Scorpaenoidei | Scorpaenidae | *Setarches guentheri* | GU225028 | *CO1* |
| Scorpaeniformes | Scorpaenoidei | Scorpaenidae | *Synanceia species* | EF607409 | *CO1* |
| Scorpaeniformes | Scorpaenoidei | Scorpaenidae | *Synanceia verrucosa* | FJ584136 | *CO1* |
| Scorpaeniformes | Scorpaenoidei | Scorpaenidae | *Synanceia verrucosa* | EU638011 | *rhod* |
| Scorpaeniformes | Scorpaenoidei | Scorpaenidae | *Taenianotus triacanthus* | FJ584158 | *CO1* |
| Scorpaeniformes | Scorpaenoidei | Scorpaenidae | *Trachyscorpia eschmeyeri* | DQ108040 | *CO1* |
| Scorpaeniformes | Scorpaenoidei | Aploactinidae | *Cocotropus monacanthus* | HQ945798 | *CO1* |
| Scorpaeniformes | Scorpaenoidei | Congiopodidae | *Congiopodus peruvianus* | EU074388 | *CO1* |
| Scorpaeniformes | Scorpaenoidei | Congiopodidae | *Congiopodus torvus* | HQ945834 | *CO1* |
| Scorpaeniformes | Scorpaenoidei | Congiopodidae | *Zanclorhynchus spinifer* | EU638021 | *rhod* |
| Scorpaeniformes | Platycephaloidei | Triglidae | *Chelidonichthys capensis* | HM007758 | *CO1* |
| Scorpaeniformes | Platycephaloidei | Triglidae | *Chelidonichthys cuculus* | EF609292 | *CO1* |
| Scorpaeniformes | Platycephaloidei | Triglidae | *Chelidonichthys cuculus* | EU492095 | *CYB* |
| Scorpaeniformes | Platycephaloidei | Triglidae | *Chelidonichthys cuculus* | EU491999 | *rhod* |
| Scorpaeniformes | Platycephaloidei | Triglidae | *Chelidonichthys kumu* | DQ108044 | *CO1* |
| Scorpaeniformes | Platycephaloidei | Triglidae | *Chelidonichthys lucernus* | EF609323 | *CO1* |
| Scorpaeniformes | Platycephaloidei | Triglidae | *Chelidonichthys lucernus* | EU223999 | *CYB* |
| Scorpaeniformes | Platycephaloidei | Triglidae | *Chelidonichthys lucernus* | AY141287 | *rhod* |
| Scorpaeniformes | Platycephaloidei | Triglidae | *Chelidonichthys obscurus* | EF439507 | *CYB* |
| Scorpaeniformes | Platycephaloidei | Triglidae | *Chelidonichthys obscurus* | EF439357 | *rhod* |
| Scorpaeniformes | Platycephaloidei | Triglidae | *Chelidonichthys spinosus* | AB326975 | *CYB* |
| Scorpaeniformes | Platycephaloidei | Triglidae | *Eutrigla gurnardus* | FN688987 | *CO1* |
| Scorpaeniformes | Platycephaloidei | Triglidae | *Eutrigla gurnardus* | EU492099 | *CYB* |
| Scorpaeniformes | Platycephaloidei | Triglidae | *Eutrigla gurnardus* | EU224104 | *rhod* |
| Scorpaeniformes | Platycephaloidei | Triglidae | *Lepidotrigla abyssalis* | GU586126 | *CO1* |
| Scorpaeniformes | Platycephaloidei | Triglidae | *Lepidotrigla cavillone* | EF439536 | *CYB* |
| Scorpaeniformes | Platycephaloidei | Triglidae | *Lepidotrigla faurei* | GU805113 | *CO1* |
| Scorpaeniformes | Platycephaloidei | Triglidae | *Lepidotrigla microptera* | AB126385 | *CYB* |
| Scorpaeniformes | Platycephaloidei | Triglidae | *Lepidotrigla mulhalli* | DQ108021 | *CO1* |
| Scorpaeniformes | Platycephaloidei | Triglidae | *Lepidotrigla vanessa* | DQ108024 | *CO1* |
| Scorpaeniformes | Platycephaloidei | Triglidae | *Prionotus ophryas* | FJ583924 | *CO1* |
| Scorpaeniformes | Platycephaloidei | Triglidae | *Prionotus stephanophrys* | GU440478 | *CO1* |
| Scorpaeniformes | Platycephaloidei | Triglidae | *Pterygotrigla pauli* | DQ108038 | *CO1* |
| Scorpaeniformes | Platycephaloidei | Triglidae | *Pterygotrigla polyommata* | DQ108033 | *CO1* |
| Scorpaeniformes | Platycephaloidei | Triglidae | *Trigla lyra* | FN688976 | *CO1* |
| Scorpaeniformes | Platycephaloidei | Triglidae | *Trigla lyra* | EU036517 | *CYB* |
| Scorpaeniformes | Platycephaloidei | Triglidae | *Trigla lyra* | EF439485 | *rhod* |
| Scorpaeniformes | Platycephaloidei | Triglidae | *Trigloporus lastoviza* | FN688907 | *CO1* |
| Scorpaeniformes | Platycephaloidei | Triglidae | *Trigloporus lastoviza* | EF427546 | *CYB* |
| Scorpaeniformes | Platycephaloidei | Triglidae | *Trigloporus lastoviza* | EF439098 | *rhod* |
| Scorpaeniformes | Platycephaloidei | Peristediidae | *Peristedion cataphractum* | HM049961 | *CYB* |
| Scorpaeniformes | Platycephaloidei | Peristediidae | *Peristedion cataphractum* | HM050128 | *ND4* |
| Scorpaeniformes | Platycephaloidei | Peristediidae | *Peristedion miniatum* | AY308774 | *rag1* |
| Scorpaeniformes | Platycephaloidei | Peristediidae | *Peristedion weberi* | HQ945813 | *CO1* |
| Scorpaeniformes | Platycephaloidei | Peristediidae | *Satyrichthys adeni* | GU804936 | *CO1* |
| Scorpaeniformes | Platycephaloidei | Peristediidae | *Scalicus amiscus* | FJ237914 | *CO1* |
| Scorpaeniformes | Platycephaloidei | Platycephalidae | *Cociella crocodila* | JF952704 | *CO1* |
| Scorpaeniformes | Platycephaloidei | Platycephalidae | *Cymbacephalus nematophthalmus* | DQ108008 | *CO1* |
| Scorpaeniformes | Platycephaloidei | Platycephalidae | *Cymbacephalus staigeri* | DQ108004 | *CO1* |
| Scorpaeniformes | Platycephaloidei | Platycephalidae | *Grammoplites scaber* | EU167896 | *rag1* |
| Scorpaeniformes | Platycephaloidei | Platycephalidae | *Inegocia guttata* | JF952765 | *CO1* |
| Scorpaeniformes | Platycephaloidei | Platycephalidae | *Kumococius rodericensis* | EU595159 | *CO1* |
| Scorpaeniformes | Platycephaloidei | Platycephalidae | *Neoplatycephalus aurimaculatus* | DQ108005 | *CO1* |
| Scorpaeniformes | Platycephaloidei | Platycephalidae | *Neoplatycephalus speculator* | DQ107956 | *CO1* |
| Scorpaeniformes | Platycephaloidei | Platycephalidae | *Papilloculiceps longiceps* | HQ561533 | *CO1* |
| Scorpaeniformes | Platycephaloidei | Platycephalidae | *Platycephalus bassensis* | DQ107991 | *CO1* |
| Scorpaeniformes | Platycephaloidei | Platycephalidae | *Platycephalus caeruleopunctatus* | DQ107994 | *CO1* |
| Scorpaeniformes | Platycephaloidei | Platycephalidae | *Platycephalus endrachtensis* | DQ108000 | *CO1* |
| Scorpaeniformes | Platycephaloidei | Platycephalidae | *Platycephalus fuscus* | DQ107989 | *CO1* |
| Scorpaeniformes | Platycephaloidei | Platycephalidae | *Platycephalus indicus* | DQ107978 | *CO1* |
| Scorpaeniformes | Platycephaloidei | Platycephalidae | *Platycephalus laevigatus* | DQ107980 | *CO1* |
| Scorpaeniformes | Platycephaloidei | Platycephalidae | *Platycephalus longispinis* | DQ107961 | *CO1* |
| Scorpaeniformes | Platycephaloidei | Platycephalidae | *Platycephalus marmoratus* | DQ107950 | *CO1* |
| Scorpaeniformes | Platycephaloidei | Platycephalidae | *Platycephalus westraliae* | DQ107997 | *CO1* |
| Scorpaeniformes | Platycephaloidei | Platycephalidae | *Rogadius portuguesus* | GU804974 | *CO1* |
| Scorpaeniformes | Platycephaloidei | Platycephalidae | *Rogadius prionotus* | HQ561519 | *CO1* |
| Scorpaeniformes | Platycephaloidei | Hoplichthyidae | *Hoplichthys acanthopleurus* | GU805035 | *CO1* |
| Scorpaeniformes | Platycephaloidei | Hoplichthyidae | *Hoplichthys citrinus* | DQ521027 | *CO1* |
| Scorpaeniformes | Anoplopomatoidei | Anoplopomatidae | *Anoplopoma fimbria* | FJ164286 | *CO1* |
| Scorpaeniformes | Anoplopomatoidei | Anoplopomatidae | *Anoplopoma fimbria* | FJ264420 | *CYB* |
| Scorpaeniformes | Hexagrammoidei | Hexagrammidae | *Hexagrammos agrammus* | AB087410 | *CYB* |
| Scorpaeniformes | Hexagrammoidei | Hexagrammidae | *Hexagrammos decagrammus* | FJ164647 | *CO1* |
| Scorpaeniformes | Hexagrammoidei | Hexagrammidae | *Hexagrammos decagrammus* | FJ264357 | *CYB* |
| Scorpaeniformes | Hexagrammoidei | Hexagrammidae | *Hexagrammos lagocephalus* | GU440340 | *CO1* |
| Scorpaeniformes | Hexagrammoidei | Hexagrammidae | *Hexagrammos lagocephalus* | AB087411 | *CYB* |
| Scorpaeniformes | Hexagrammoidei | Hexagrammidae | *Hexagrammos octogrammus* | AB087412 | *CYB* |
| Scorpaeniformes | Hexagrammoidei | Hexagrammidae | *Hexagrammos otakii* | JF511643 | *CO1* |
| Scorpaeniformes | Hexagrammoidei | Hexagrammidae | *Hexagrammos otakii* | AB087409 | *CYB* |
| Scorpaeniformes | Hexagrammoidei | Hexagrammidae | *Hexagrammos stelleri* | GU440341 | *CO1* |
| Scorpaeniformes | Hexagrammoidei | Hexagrammidae | *Hexagrammos stelleri* | AB087413 | *CYB* |
| Scorpaeniformes | Hexagrammoidei | Hexagrammidae | *Ophiodon elongatus* | FJ164942 | *CO1* |
| Scorpaeniformes | Hexagrammoidei | Hexagrammidae | *Ophiodon elongatus* | AY225661 | *CYB* |
| Scorpaeniformes | Hexagrammoidei | Hexagrammidae | *Ophiodon elongatus* | AY225719 | *ND2* |
| Scorpaeniformes | Hexagrammoidei | Hexagrammidae | *Oxylebius pictus* | GU440441 | *CO1* |
| Scorpaeniformes | Hexagrammoidei | Hexagrammidae | *Pleurogrammus azonus* | EU856711 | *CO1* |
| Scorpaeniformes | Hexagrammoidei | Hexagrammidae | *Pleurogrammus azonus* | AB087415 | *CYB* |
| Scorpaeniformes | Hexagrammoidei | Hexagrammidae | *Pleurogrammus monopterygius* | EU856714 | *CO1* |
| Scorpaeniformes | Hexagrammoidei | Hexagrammidae | *Pleurogrammus monopterygius* | AB087414 | *CYB* |
| Scorpaeniformes | Hexagrammoidei | Zaniolepididae | *Zaniolepis latipinnis* | FJ264277 | *CYB* |
| Scorpaeniformes | Cottoidei | Rhamphocottidae | *Rhamphocottus richardsoni* | EU547245 | *CO1* |
| Scorpaeniformes | Cottoidei | Rhamphocottidae | *Rhamphocottus richardsoni* | FJ264314 | *CYB* |
| Scorpaeniformes | Cottoidei | Rhamphocottidae | *Rhamphocottus richardsonii* | GU440501 | *CO1* |
| Scorpaeniformes | Cottoidei | Cottidae | *Artediellus atlanticus* | HM421739 | *CO1* |
| Scorpaeniformes | Cottoidei | Cottidae | *Artediellus fuscimentus* | AB258329 | *CYB* |
| Scorpaeniformes | Cottoidei | Cottidae | *Artediellus scaber* | HM421787 | *CO1* |
| Scorpaeniformes | Cottoidei | Cottidae | *Artedius corallinus* | GU440235 | *CO1* |
| Scorpaeniformes | Cottoidei | Cottidae | *Artedius corallinus* | EF521374 | *CYB* |
| Scorpaeniformes | Cottoidei | Cottidae | *Artedius corallinus* | EF521347 | *ND1* |
| Scorpaeniformes | Cottoidei | Cottidae | *Artedius fenestralis* | FJ164323 | *CO1* |
| Scorpaeniformes | Cottoidei | Cottidae | *Artedius fenestralis* | EU836698 | *CYB* |
| Scorpaeniformes | Cottoidei | Cottidae | *Artedius fenestralis* | EF521350 | *ND1* |
| Scorpaeniformes | Cottoidei | Cottidae | *Artedius harringtoni* | GU440236 | *CO1* |
| Scorpaeniformes | Cottoidei | Cottidae | *Artedius harringtoni* | EU836700 | *CYB* |
| Scorpaeniformes | Cottoidei | Cottidae | *Artedius harringtoni* | EF521351 | *ND1* |
| Scorpaeniformes | Cottoidei | Cottidae | *Artedius lateralis* | JN024814 | *CO1* |
| Scorpaeniformes | Cottoidei | Cottidae | *Artedius lateralis* | EU836703 | *CYB* |
| Scorpaeniformes | Cottoidei | Cottidae | *Artedius lateralis* | EF521346 | *ND1* |
| Scorpaeniformes | Cottoidei | Cottidae | *Artedius notospilotus* | GU440239 | *CO1* |
| Scorpaeniformes | Cottoidei | Cottidae | *Artedius notospilotus* | EF521377 | *CYB* |
| Scorpaeniformes | Cottoidei | Cottidae | *Artedius notospilotus* | EF521352 | *ND1* |
| Scorpaeniformes | Cottoidei | Cottidae | *Batrachocottus multiradiatus* | AY116357 | *CYB* |
| Scorpaeniformes | Cottoidei | Cottidae | *Batrachocottus nikolskii* | AY116358 | *CYB* |
| Scorpaeniformes | Cottoidei | Cottidae | *Chitonotus pugetensis* | FJ164460 | *CO1* |
| Scorpaeniformes | Cottoidei | Cottidae | *Chitonotus pugetensis* | EF521368 | *CYB* |
| Scorpaeniformes | Cottoidei | Cottidae | *Chitonotus pugetensis* | EF521343 | *ND1* |
| Scorpaeniformes | Cottoidei | Cottidae | *Clinocottus acuticeps* | GU440285 | *CO1* |
| Scorpaeniformes | Cottoidei | Cottidae | *Clinocottus acuticeps* | EF521387 | *CYB* |
| Scorpaeniformes | Cottoidei | Cottidae | *Clinocottus acuticeps* | EF521362 | *ND1* |
| Scorpaeniformes | Cottoidei | Cottidae | *Clinocottus analis* | AY833272 | *ATP6* |
| Scorpaeniformes | Cottoidei | Cottidae | *Clinocottus analis* | JN024967 | *CO1* |
| Scorpaeniformes | Cottoidei | Cottidae | *Clinocottus analis* | AY833327 | *CYB* |
| Scorpaeniformes | Cottoidei | Cottidae | *Clinocottus analis* | EF521358 | *ND1* |
| Scorpaeniformes | Cottoidei | Cottidae | *Clinocottus embryum* | GU440287 | *CO1* |
| Scorpaeniformes | Cottoidei | Cottidae | *Clinocottus embryum* | EF521386 | *CYB* |
| Scorpaeniformes | Cottoidei | Cottidae | *Clinocottus embryum* | EF521361 | *ND1* |
| Scorpaeniformes | Cottoidei | Cottidae | *Clinocottus globiceps* | GU440288 | *CO1* |
| Scorpaeniformes | Cottoidei | Cottidae | *Clinocottus globiceps* | EU836696 | *CYB* |
| Scorpaeniformes | Cottoidei | Cottidae | *Clinocottus globiceps* | EF521359 | *ND1* |
| Scorpaeniformes | Cottoidei | Cottidae | *Clinocottus recalvus* | GU440289 | *CO1* |
| Scorpaeniformes | Cottoidei | Cottidae | *Clinocottus recalvus* | EF521385 | *CYB* |
| Scorpaeniformes | Cottoidei | Cottidae | *Clinocottus recalvus* | EF521360 | *ND1* |
| Scorpaeniformes | Cottoidei | Cottidae | *Cottiusculus gonez* | AB258327 | *CYB* |
| Scorpaeniformes | Cottoidei | Cottidae | *Cottiusculus schmidti* | AB258323 | *CYB* |
| Scorpaeniformes | Cottoidei | Cottidae | *Cottocomephorus grewingki* | AY116359 | *CYB* |
| Scorpaeniformes | Cottoidei | Cottidae | *Cottocomephorus inermis* | AY116360 | *CYB* |
| Scorpaeniformes | Cottoidei | Cottidae | *Cottus aleuticus* | AY833273 | *ATP6* |
| Scorpaeniformes | Cottoidei | Cottidae | *Cottus aleuticus* | JN024990 | *CO1* |
| Scorpaeniformes | Cottoidei | Cottidae | *Cottus aleuticus* | AY833328 | *CYB* |
| Scorpaeniformes | Cottoidei | Cottidae | *Cottus amblystomopsis* | AY833274 | *ATP6* |
| Scorpaeniformes | Cottoidei | Cottidae | *Cottus amblystomopsis* | AY833329 | *CYB* |
| Scorpaeniformes | Cottoidei | Cottidae | *Cottus asper* | AY833275 | *ATP6* |
| Scorpaeniformes | Cottoidei | Cottidae | *Cottus asper* | EU523997 | *CO1* |
| Scorpaeniformes | Cottoidei | Cottidae | *Cottus asper* | EU836699 | *CYB* |
| Scorpaeniformes | Cottoidei | Cottidae | *Cottus asperrimus* | AY833276 | *ATP6* |
| Scorpaeniformes | Cottoidei | Cottidae | *Cottus asperrimus* | AY833331 | *CYB* |
| Scorpaeniformes | Cottoidei | Cottidae | *Cottus aturi* | EF416966 | *CO1* |
| Scorpaeniformes | Cottoidei | Cottidae | *Cottus baileyi* | AY833277 | *ATP6* |
| Scorpaeniformes | Cottoidei | Cottidae | *Cottus baileyi* | AY833332 | *CYB* |
| Scorpaeniformes | Cottoidei | Cottidae | *Cottus bairdii* | AY116331 | *ATP6* |
| Scorpaeniformes | Cottoidei | Cottidae | *Cottus bairdii* | JN025005 | *CO1* |
| Scorpaeniformes | Cottoidei | Cottidae | *Cottus bairdii* | AY116363 | *CYB* |
| Scorpaeniformes | Cottoidei | Cottidae | *Cottus bairdii bairdii* | AY833278 | *ATP6* |
| Scorpaeniformes | Cottoidei | Cottidae | *Cottus bairdii bairdii* | AY833333 | *CYB* |
| Scorpaeniformes | Cottoidei | Cottidae | *Cottus bairdii kumlieni* | AY833282 | *ATP6* |
| Scorpaeniformes | Cottoidei | Cottidae | *Cottus bairdii kumlieni* | AY833334 | *CYB* |
| Scorpaeniformes | Cottoidei | Cottidae | *Cottus bairdii punctulatus* | AY833283 | *ATP6* |
| Scorpaeniformes | Cottoidei | Cottidae | *Cottus bairdii punctulatus* | AY833335 | *CYB* |
| Scorpaeniformes | Cottoidei | Cottidae | *Cottus bairdii semiscaber* | AY833284 | *ATP6* |
| Scorpaeniformes | Cottoidei | Cottidae | *Cottus bairdii semiscaber* | AY833336 | *CYB* |
| Scorpaeniformes | Cottoidei | Cottidae | *Cottus beldingii* | AY833286 | *ATP6* |
| Scorpaeniformes | Cottoidei | Cottidae | *Cottus beldingii* | JN025028 | *CO1* |
| Scorpaeniformes | Cottoidei | Cottidae | *Cottus beldingii* | AF549117 | *CYB* |
| Scorpaeniformes | Cottoidei | Cottidae | *Cottus bendirei* | AY833287 | *ATP6* |
| Scorpaeniformes | Cottoidei | Cottidae | *Cottus bendirei* | AY833338 | *CYB* |
| Scorpaeniformes | Cottoidei | Cottidae | *Cottus caeruleomentum* | AY833289 | *ATP6* |
| Scorpaeniformes | Cottoidei | Cottidae | *Cottus caeruleomentum* | JN025045 | *CO1* |
| Scorpaeniformes | Cottoidei | Cottidae | *Cottus caeruleomentum* | AF549122 | *CYB* |
| Scorpaeniformes | Cottoidei | Cottidae | *Cottus carolinae* | AY833290 | *ATP6* |
| Scorpaeniformes | Cottoidei | Cottidae | *Cottus carolinae* | JN025061 | *CO1* |
| Scorpaeniformes | Cottoidei | Cottidae | *Cottus carolinae* | AF549111 | *CYB* |
| Scorpaeniformes | Cottoidei | Cottidae | *Cottus chattahoochee* | JN025077 | *CO1* |
| Scorpaeniformes | Cottoidei | Cottidae | *Cottus cognatus* | AY833293 | *ATP6* |
| Scorpaeniformes | Cottoidei | Cottidae | *Cottus cognatus* | JN025090 | *CO1* |
| Scorpaeniformes | Cottoidei | Cottidae | *Cottus cognatus* | AY116364 | *CYB* |
| Scorpaeniformes | Cottoidei | Cottidae | *Cottus confusus* | AY833294 | *ATP6* |
| Scorpaeniformes | Cottoidei | Cottidae | *Cottus confusus* | AY833343 | *CYB* |
| Scorpaeniformes | Cottoidei | Cottidae | *Cottus duranii* | EF416968 | *CO1* |
| Scorpaeniformes | Cottoidei | Cottidae | *Cottus extensus* | AY833295 | *ATP6* |
| Scorpaeniformes | Cottoidei | Cottidae | *Cottus extensus* | AY833344 | *CYB* |
| Scorpaeniformes | Cottoidei | Cottidae | *Cottus ferrugineus* | AY833296 | *ATP6* |
| Scorpaeniformes | Cottoidei | Cottidae | *Cottus ferrugineus* | AY833345 | *CYB* |
| Scorpaeniformes | Cottoidei | Cottidae | *Cottus girardi* | AY833297 | *ATP6* |
| Scorpaeniformes | Cottoidei | Cottidae | *Cottus girardi* | JN025101 | *CO1* |
| Scorpaeniformes | Cottoidei | Cottidae | *Cottus girardi* | AY833346 | *CYB* |
| Scorpaeniformes | Cottoidei | Cottidae | *Cottus gobio* | AY116334 | *ATP6* |
| Scorpaeniformes | Cottoidei | Cottidae | *Cottus gobio* | EF416971 | *CO1* |
| Scorpaeniformes | Cottoidei | Cottidae | *Cottus gobio* | AY116366 | *CYB* |
| Scorpaeniformes | Cottoidei | Cottidae | *Cottus gobio* | HM050100 | *ND4* |
| Scorpaeniformes | Cottoidei | Cottidae | *Cottus greenei* | AY833298 | *ATP6* |
| Scorpaeniformes | Cottoidei | Cottidae | *Cottus greenei* | AY833347 | *CYB* |
| Scorpaeniformes | Cottoidei | Cottidae | *Cottus gulosus* | AY833299 | *ATP6* |
| Scorpaeniformes | Cottoidei | Cottidae | *Cottus gulosus* | JN025103 | *CO1* |
| Scorpaeniformes | Cottoidei | Cottidae | *Cottus gulosus* | AY833348 | *CYB* |
| Scorpaeniformes | Cottoidei | Cottidae | *Cottus hangiongensis* | AY833300 | *ATP6* |
| Scorpaeniformes | Cottoidei | Cottidae | *Cottus hangiongensis* | AY833349 | *CYB* |
| Scorpaeniformes | Cottoidei | Cottidae | *Cottus hubbsi* | AY833301 | *ATP6* |
| Scorpaeniformes | Cottoidei | Cottidae | *Cottus hubbsi* | JN025104 | *CO1* |
| Scorpaeniformes | Cottoidei | Cottidae | *Cottus hubbsi* | AY833350 | *CYB* |
| Scorpaeniformes | Cottoidei | Cottidae | *Cottus hypselurus* | AY833303 | *ATP6* |
| Scorpaeniformes | Cottoidei | Cottidae | *Cottus hypselurus* | JN025111 | *CO1* |
| Scorpaeniformes | Cottoidei | Cottidae | *Cottus hypselurus* | AF549154 | *CYB* |
| Scorpaeniformes | Cottoidei | Cottidae | *Cottus kazika* | AY833304 | *ATP6* |
| Scorpaeniformes | Cottoidei | Cottidae | *Cottus kazika* | AY833351 | *CYB* |
| Scorpaeniformes | Cottoidei | Cottidae | *Cottus kesslerii* | AY116329 | *ATP6* |
| Scorpaeniformes | Cottoidei | Cottidae | *Cottus kesslerii* | AY116361 | *CYB* |
| Scorpaeniformes | Cottoidei | Cottidae | *Cottus kesslerii* | L42953 | *rhod* |
| Scorpaeniformes | Cottoidei | Cottidae | *Cottus klamathensis* | JN025112 | *CO1* |
| Scorpaeniformes | Cottoidei | Cottidae | *Cottus klamathensis klamathensis* | AY833305 | *ATP6* |
| Scorpaeniformes | Cottoidei | Cottidae | *Cottus klamathensis klamathensis* | AY833352 | *CYB* |
| Scorpaeniformes | Cottoidei | Cottidae | *Cottus klamathensis macrops* | AY833306 | *ATP6* |
| Scorpaeniformes | Cottoidei | Cottidae | *Cottus klamathensis macrops* | AY833353 | *CYB* |
| Scorpaeniformes | Cottoidei | Cottidae | *Cottus klamathensis polyporus* | AY833354 | *CYB* |
| Scorpaeniformes | Cottoidei | Cottidae | *Cottus leiopomus* | AY833308 | *ATP6* |
| Scorpaeniformes | Cottoidei | Cottidae | *Cottus leiopomus* | AY833355 | *CYB* |
| Scorpaeniformes | Cottoidei | Cottidae | *Cottus marginatus* | AY833309 | *ATP6* |
| Scorpaeniformes | Cottoidei | Cottidae | *Cottus marginatus* | AY833356 | *CYB* |
| Scorpaeniformes | Cottoidei | Cottidae | *Cottus nozawae* | AY833310 | *ATP6* |
| Scorpaeniformes | Cottoidei | Cottidae | *Cottus nozawae* | AY833357 | *CYB* |
| Scorpaeniformes | Cottoidei | Cottidae | *Cottus paulus* | AY833311 | *ATP6* |
| Scorpaeniformes | Cottoidei | Cottidae | *Cottus paulus* | AY833358 | *CYB* |
| Scorpaeniformes | Cottoidei | Cottidae | *Cottus perifretum* | EF416978 | *CO1* |
| Scorpaeniformes | Cottoidei | Cottidae | *Cottus perplexus* | AY833313 | *ATP6* |
| Scorpaeniformes | Cottoidei | Cottidae | *Cottus perplexus* | JN025119 | *CO1* |
| Scorpaeniformes | Cottoidei | Cottidae | *Cottus perplexus* | AY833359 | *CYB* |
| Scorpaeniformes | Cottoidei | Cottidae | *Cottus pitensis* | AY833314 | *ATP6* |
| Scorpaeniformes | Cottoidei | Cottidae | *Cottus pitensis* | JN025126 | *CO1* |
| Scorpaeniformes | Cottoidei | Cottidae | *Cottus pitensis* | AY833360 | *CYB* |
| Scorpaeniformes | Cottoidei | Cottidae | *Cottus poecilopus* | AY116336 | *ATP6* |
| Scorpaeniformes | Cottoidei | Cottidae | *Cottus poecilopus* | HQ536340 | *CO1* |
| Scorpaeniformes | Cottoidei | Cottidae | *Cottus poecilopus* | AY116370 | *CYB* |
| Scorpaeniformes | Cottoidei | Cottidae | *Cottus poecilopus volki* | AY833315 | *ATP6* |
| Scorpaeniformes | Cottoidei | Cottidae | *Cottus poecilopus volki* | AY833361 | *CYB* |
| Scorpaeniformes | Cottoidei | Cottidae | *Cottus pollux* | AY116337 | *ATP6* |
| Scorpaeniformes | Cottoidei | Cottidae | *Cottus pollux* | AY116368 | *CYB* |
| Scorpaeniformes | Cottoidei | Cottidae | *Cottus princeps* | AY833316 | *ATP6* |
| Scorpaeniformes | Cottoidei | Cottidae | *Cottus princeps* | AY833362 | *CYB* |
| Scorpaeniformes | Cottoidei | Cottidae | *Cottus reinii* | AY116338 | *ATP6* |
| Scorpaeniformes | Cottoidei | Cottidae | *Cottus reinii* | AY116369 | *CYB* |
| Scorpaeniformes | Cottoidei | Cottidae | *Cottus rhenanus* | EF416986 | *CO1* |
| Scorpaeniformes | Cottoidei | Cottidae | *Cottus rhotheus* | AY833317 | *ATP6* |
| Scorpaeniformes | Cottoidei | Cottidae | *Cottus rhotheus* | JN025132 | *CO1* |
| Scorpaeniformes | Cottoidei | Cottidae | *Cottus rhotheus* | AF549114 | *CYB* |
| Scorpaeniformes | Cottoidei | Cottidae | *Cottus ricei* | AY833318 | *ATP6* |
| Scorpaeniformes | Cottoidei | Cottidae | *Cottus ricei* | JN025136 | *CO1* |
| Scorpaeniformes | Cottoidei | Cottidae | *Cottus ricei* | AY833363 | *CYB* |
| Scorpaeniformes | Cottoidei | Cottidae | *Cottus sibiricus* | AY116335 | *ATP6* |
| Scorpaeniformes | Cottoidei | Cottidae | *Cottus sibiricus* | EF416990 | *CO1* |
| Scorpaeniformes | Cottoidei | Cottidae | *Cottus sibiricus* | AY116367 | *CYB* |
| Scorpaeniformes | Cottoidei | Cottidae | *Cottus tallapoosae* | JN025144 | *CO1* |
| Scorpaeniformes | Cottoidei | Cottidae | *Cottus tenuis* | JN025147 | *CO1* |
| Scorpaeniformes | Cottoidei | Cottidae | *Enophrys bison* | GU440313 | *CO1* |
| Scorpaeniformes | Cottoidei | Cottidae | *Enophrys bison* | EU836693 | *CYB* |
| Scorpaeniformes | Cottoidei | Cottidae | *Enophrys bison* | EF521341 | *ND1* |
| Scorpaeniformes | Cottoidei | Cottidae | *Enophrys diceraus* | HM421800 | *CO1* |
| Scorpaeniformes | Cottoidei | Cottidae | *Enophrys taurina* | GU440315 | *CO1* |
| Scorpaeniformes | Cottoidei | Cottidae | *Gymnocanthus detrisus* | AB126383 | *CYB* |
| Scorpaeniformes | Cottoidei | Cottidae | *Gymnocanthus herzensteini* | AB126384 | *CYB* |
| Scorpaeniformes | Cottoidei | Cottidae | *Gymnocanthus intermedius* | JF952748 | *CO1* |
| Scorpaeniformes | Cottoidei | Cottidae | *Gymnocanthus tricuspis* | HM421749 | *CO1* |
| Scorpaeniformes | Cottoidei | Cottidae | *Hemilepidotus gilberti* | AB114905 | *CYB* |
| Scorpaeniformes | Cottoidei | Cottidae | *Hemilepidotus hemilepidotus* | EF521369 | *CYB* |
| Scorpaeniformes | Cottoidei | Cottidae | *Hemilepidotus hemilepidotus* | EF521344 | *ND1* |
| Scorpaeniformes | Cottoidei | Cottidae | *Hemilepidotus jordani* | AY833324 | *ATP6* |
| Scorpaeniformes | Cottoidei | Cottidae | *Hemilepidotus jordani* | AY833367 | *CYB* |
| Scorpaeniformes | Cottoidei | Cottidae | *Hemilepidotus papilio* | HM421776 | *CO1* |
| Scorpaeniformes | Cottoidei | Cottidae | *Hemilepidotus spinosus* | GU440336 | *CO1* |
| Scorpaeniformes | Cottoidei | Cottidae | *Hemilepidotus spinosus* | FJ264349 | *CYB* |
| Scorpaeniformes | Cottoidei | Cottidae | *Icelinus borealis* | EF521367 | *CYB* |
| Scorpaeniformes | Cottoidei | Cottidae | *Icelinus borealis* | EF521342 | *ND1* |
| Scorpaeniformes | Cottoidei | Cottidae | *Icelinus burchami* | FJ164688 | *CO1* |
| Scorpaeniformes | Cottoidei | Cottidae | *Icelinus burchami* | AY973069 | *CYB* |
| Scorpaeniformes | Cottoidei | Cottidae | *Icelinus cavifrons* | GU440353 | *CO1* |
| Scorpaeniformes | Cottoidei | Cottidae | *Icelinus cavifrons* | AY973070 | *CYB* |
| Scorpaeniformes | Cottoidei | Cottidae | *Icelinus filamentosus* | FJ164695 | *CO1* |
| Scorpaeniformes | Cottoidei | Cottidae | *Icelinus filamentosus* | FJ264425 | *CYB* |
| Scorpaeniformes | Cottoidei | Cottidae | *Icelinus fimbriatus* | GU440354 | *CO1* |
| Scorpaeniformes | Cottoidei | Cottidae | *Icelinus oculatus* | GU440355 | *CO1* |
| Scorpaeniformes | Cottoidei | Cottidae | *Icelinus quadriseriatus* | GU440356 | *CO1* |
| Scorpaeniformes | Cottoidei | Cottidae | *Icelinus tenuis* | GU440357 | *CO1* |
| Scorpaeniformes | Cottoidei | Cottidae | *Icelinus tenuis* | FJ264469 | *CYB* |
| Scorpaeniformes | Cottoidei | Cottidae | *Icelus cataphractus* | AB126381 | *CYB* |
| Scorpaeniformes | Cottoidei | Cottidae | *Icelus spatula* | GU804865 | *CO1* |
| Scorpaeniformes | Cottoidei | Cottidae | *Icelus spiniger* | FJ164703 | *CO1* |
| Scorpaeniformes | Cottoidei | Cottidae | *Jordania zonope* | EF521365 | *CYB* |
| Scorpaeniformes | Cottoidei | Cottidae | *Jordania zonope* | EF521340 | *ND1* |
| Scorpaeniformes | Cottoidei | Cottidae | *Leiocottus hirundo* | HQ010066 | *CO1* |
| Scorpaeniformes | Cottoidei | Cottidae | *Leiocottus hirundo* | EF521382 | *CYB* |
| Scorpaeniformes | Cottoidei | Cottidae | *Leiocottus hirundo* | EF521357 | *ND1* |
| Scorpaeniformes | Cottoidei | Cottidae | *Leptocottus armatus* | AY833323 | *ATP6* |
| Scorpaeniformes | Cottoidei | Cottidae | *Leptocottus armatus* | GU440370 | *CO1* |
| Scorpaeniformes | Cottoidei | Cottidae | *Leptocottus armatus* | EU836697 | *CYB* |
| Scorpaeniformes | Cottoidei | Cottidae | *Leptocottus armatus* | EF521339 | *ND1* |
| Scorpaeniformes | Cottoidei | Cottidae | *Megalocottus platycephalus* | JF278615 | *CO1* |
| Scorpaeniformes | Cottoidei | Cottidae | *Megalocottus platycephalus* | JF290405 | *CYB* |
| Scorpaeniformes | Cottoidei | Cottidae | *Myoxocephalus aenaeus* | AY339240 | *ATP6* |
| Scorpaeniformes | Cottoidei | Cottidae | *Myoxocephalus aenaeus* | AY338277 | *CYB* |
| Scorpaeniformes | Cottoidei | Cottidae | *Myoxocephalus brandtii* | JF278612 | *CO1* |
| Scorpaeniformes | Cottoidei | Cottidae | *Myoxocephalus brandtii* | JF290403 | *CYB* |
| Scorpaeniformes | Cottoidei | Cottidae | *Myoxocephalus jaok* | JF278617 | *CO1* |
| Scorpaeniformes | Cottoidei | Cottidae | *Myoxocephalus jaok* | JF290407 | *CYB* |
| Scorpaeniformes | Cottoidei | Cottidae | *Myoxocephalus ochotensis* | JF278619 | *CO1* |
| Scorpaeniformes | Cottoidei | Cottidae | *Myoxocephalus ochotensis* | JF290409 | *CYB* |
| Scorpaeniformes | Cottoidei | Cottidae | *Myoxocephalus octodecemspinosus* | AY339241 | *ATP6* |
| Scorpaeniformes | Cottoidei | Cottidae | *Myoxocephalus octodecemspinosus* | AY338279 | *CYB* |
| Scorpaeniformes | Cottoidei | Cottidae | *Myoxocephalus polyacanthocephalus* | AY339242 | *ATP6* |
| Scorpaeniformes | Cottoidei | Cottidae | *Myoxocephalus polyacanthocephalus* | AB114909 | *CYB* |
| Scorpaeniformes | Cottoidei | Cottidae | *Myoxocephalus scorpius* | GU804857 | *CO1* |
| Scorpaeniformes | Cottoidei | Cottidae | *Myoxocephalus scorpius* | EU492125 | *CYB* |
| Scorpaeniformes | Cottoidei | Cottidae | *Myoxocephalus scorpius* | EU492226 | *rhod* |
| Scorpaeniformes | Cottoidei | Cottidae | *Myoxocephalus stelleri* | AY339244 | *ATP6* |
| Scorpaeniformes | Cottoidei | Cottidae | *Myoxocephalus stelleri* | AY338282 | *CYB* |
| Scorpaeniformes | Cottoidei | Cottidae | *Myoxocephalus thompsonii* | AY833326 | *ATP6* |
| Scorpaeniformes | Cottoidei | Cottidae | *Myoxocephalus thompsonii* | EU524914 | *CO1* |
| Scorpaeniformes | Cottoidei | Cottidae | *Myoxocephalus thompsonii* | AY833369 | *CYB* |
| Scorpaeniformes | Cottoidei | Cottidae | *Oligocottus maculosus* | FJ164922 | *CO1* |
| Scorpaeniformes | Cottoidei | Cottidae | *Oligocottus maculosus* | EU836704 | *CYB* |
| Scorpaeniformes | Cottoidei | Cottidae | *Oligocottus maculosus* | EF521354 | *ND1* |
| Scorpaeniformes | Cottoidei | Cottidae | *Oligocottus rimensis* | GU440428 | *CO1* |
| Scorpaeniformes | Cottoidei | Cottidae | *Oligocottus rimensis* | EF521380 | *CYB* |
| Scorpaeniformes | Cottoidei | Cottidae | *Oligocottus rimensis* | EF521355 | *ND1* |
| Scorpaeniformes | Cottoidei | Cottidae | *Oligocottus rubellio* | GU440429 | *CO1* |
| Scorpaeniformes | Cottoidei | Cottidae | *Oligocottus rubellio* | EF521381 | *CYB* |
| Scorpaeniformes | Cottoidei | Cottidae | *Oligocottus rubellio* | EF521356 | *ND1* |
| Scorpaeniformes | Cottoidei | Cottidae | *Oligocottus snyderi* | GU440430 | *CO1* |
| Scorpaeniformes | Cottoidei | Cottidae | *Oligocottus snyderi* | EU836695 | *CYB* |
| Scorpaeniformes | Cottoidei | Cottidae | *Oligocottus snyderi* | EF521353 | *ND1* |
| Scorpaeniformes | Cottoidei | Cottidae | *Orthonopias triacis* | GU440439 | *CO1* |
| Scorpaeniformes | Cottoidei | Cottidae | *Orthonopias triacis* | EF521370 | *CYB* |
| Scorpaeniformes | Cottoidei | Cottidae | *Orthonopias triacis* | EF521345 | *ND1* |
| Scorpaeniformes | Cottoidei | Cottidae | *Paracottus knerii* | AY116362 | *CYB* |
| Scorpaeniformes | Cottoidei | Cottidae | *Radulinus asprellus* | FJ165074 | *CO1* |
| Scorpaeniformes | Cottoidei | Cottidae | *Radulinus asprellus* | FJ264438 | *CYB* |
| Scorpaeniformes | Cottoidei | Cottidae | *Ruscarius creaseri* | GU440508 | *CO1* |
| Scorpaeniformes | Cottoidei | Cottidae | *Ruscarius creaseri* | EF521371 | *CYB* |
| Scorpaeniformes | Cottoidei | Cottidae | *Ruscarius creaseri* | EF521348 | *ND1* |
| Scorpaeniformes | Cottoidei | Cottidae | *Ruscarius meanyi* | EF521372 | *CYB* |
| Scorpaeniformes | Cottoidei | Cottidae | *Ruscarius meanyi* | EF521349 | *ND1* |
| Scorpaeniformes | Cottoidei | Cottidae | *Scorpaenichthys marmoratus* | AY833325 | *ATP6* |
| Scorpaeniformes | Cottoidei | Cottidae | *Scorpaenichthys marmoratus* | GU440517 | *CO1* |
| Scorpaeniformes | Cottoidei | Cottidae | *Scorpaenichthys marmoratus* | EU836694 | *CYB* |
| Scorpaeniformes | Cottoidei | Cottidae | *Scorpaenichthys marmoratus* | EF521338 | *ND1* |
| Scorpaeniformes | Cottoidei | Cottidae | *Synchirus gilli* | GU440542 | *CO1* |
| Scorpaeniformes | Cottoidei | Cottidae | *Taurulus bubalis* | EU492318 | *CYB* |
| Scorpaeniformes | Cottoidei | Cottidae | *Taurulus bubalis* | EU492224 | *rhod* |
| Scorpaeniformes | Cottoidei | Cottidae | *Trachidermus fasciatus* | HQ536516 | *CO1* |
| Scorpaeniformes | Cottoidei | Cottidae | *Trichocottus brashnikovi* | HM421806 | *CO1* |
| Scorpaeniformes | Cottoidei | Cottidae | *Triglops macellus* | FJ264350 | *CYB* |
| Scorpaeniformes | Cottoidei | Cottidae | *Triglops nybelini* | HM421731 | *CO1* |
| Scorpaeniformes | Cottoidei | Cottidae | *Triglops pingelii* | GU804861 | *CO1* |
| Scorpaeniformes | Cottoidei | Cottidae | *Triglops pingelii* | FJ264373 | *CYB* |
| Scorpaeniformes | Cottoidei | Cottidae | *Triglopsis quadricornis* | AY339231 | *ATP6* |
| Scorpaeniformes | Cottoidei | Cottidae | *Triglopsis quadricornis* | EU524913 | *CO1* |
| Scorpaeniformes | Cottoidei | Cottidae | *Triglopsis quadricornis* | AY338271 | *CYB* |
| Scorpaeniformes | Cottoidei | Cottidae | *Triglopsis quadricornis* | EU492223 | *rhod* |
| Scorpaeniformes | Cottoidei | Cottidae | *Triglopsis quadricornis thompsonii* | AY338275 | *CYB* |
| Scorpaeniformes | Cottoidei | Cottidae | *Zesticelus profundorum* | HM481476 | *CO1* |
| Scorpaeniformes | Cottoidei | Comephoridae | *Comephorus baicalensis* | AY116323 | *ATP6* |
| Scorpaeniformes | Cottoidei | Comephoridae | *Comephorus baicalensis* | AY116355 | *CYB* |
| Scorpaeniformes | Cottoidei | Comephoridae | *Comephorus dybowskii* | AY116324 | *ATP6* |
| Scorpaeniformes | Cottoidei | Comephoridae | *Comephorus dybowskii* | AY116356 | *CYB* |
| Scorpaeniformes | Cottoidei | Abyssocottidae | *Abyssocottus gibbosus* | AY116309 | *ATP6* |
| Scorpaeniformes | Cottoidei | Abyssocottidae | *Abyssocottus korotneffi* | AY116310 | *ATP6* |
| Scorpaeniformes | Cottoidei | Abyssocottidae | *Asprocottus herzensteini* | AY116312 | *ATP6* |
| Scorpaeniformes | Cottoidei | Abyssocottidae | *Asprocottus herzensteini* | AY116344 | *CYB* |
| Scorpaeniformes | Cottoidei | Abyssocottidae | *Asprocottus korjakovi* | AY116311 | *ATP6* |
| Scorpaeniformes | Cottoidei | Abyssocottidae | *Asprocottus korjakovi* | AY116343 | *CYB* |
| Scorpaeniformes | Cottoidei | Abyssocottidae | *Asprocottus platycephalus* | AY116313 | *ATP6* |
| Scorpaeniformes | Cottoidei | Abyssocottidae | *Asprocottus platycephalus* | AY116345 | *CYB* |
| Scorpaeniformes | Cottoidei | Abyssocottidae | *Asprocottus pulcher* | AY116314 | *ATP6* |
| Scorpaeniformes | Cottoidei | Abyssocottidae | *Asprocottus pulcher* | AY116346 | *CYB* |
| Scorpaeniformes | Cottoidei | Abyssocottidae | *Batrachocottus multiradiatus* | AY116325 | *ATP6* |
| Scorpaeniformes | Cottoidei | Abyssocottidae | *Batrachocottus nikolskii* | AY116326 | *ATP6* |
| Scorpaeniformes | Cottoidei | Abyssocottidae | *Cottinella boulengeri* | AY116315 | *ATP6* |
| Scorpaeniformes | Cottoidei | Abyssocottidae | *Cottinella boulengeri* | AY116347 | *CYB* |
| Scorpaeniformes | Cottoidei | Abyssocottidae | *Cottocomephorus grewingki* | AY116327 | *ATP6* |
| Scorpaeniformes | Cottoidei | Abyssocottidae | *Cottocomephorus inermis* | AY116328 | *ATP6* |
| Scorpaeniformes | Cottoidei | Abyssocottidae | *Cyphocottus eurystomus* | AY116317 | *ATP6* |
| Scorpaeniformes | Cottoidei | Abyssocottidae | *Cyphocottus eurystomus* | AY116348 | *CYB* |
| Scorpaeniformes | Cottoidei | Abyssocottidae | *Limnocottus bergianus* | AY116316 | *ATP6* |
| Scorpaeniformes | Cottoidei | Abyssocottidae | *Limnocottus bergianus* | AY116349 | *CYB* |
| Scorpaeniformes | Cottoidei | Abyssocottidae | *Limnocottus godlewskii* | AY116318 | *ATP6* |
| Scorpaeniformes | Cottoidei | Abyssocottidae | *Limnocottus godlewskii* | AY116350 | *CYB* |
| Scorpaeniformes | Cottoidei | Abyssocottidae | *Limnocottus griseus* | AY116319 | *ATP6* |
| Scorpaeniformes | Cottoidei | Abyssocottidae | *Limnocottus griseus* | AY116351 | *CYB* |
| Scorpaeniformes | Cottoidei | Abyssocottidae | *Limnocottus pallidus* | AY116320 | *ATP6* |
| Scorpaeniformes | Cottoidei | Abyssocottidae | *Limnocottus pallidus* | AY116352 | *CYB* |
| Scorpaeniformes | Cottoidei | Abyssocottidae | *Paracottus knerii* | AY116330 | *ATP6* |
| Scorpaeniformes | Cottoidei | Abyssocottidae | *Procottus jettelesii* | AY116321 | *ATP6* |
| Scorpaeniformes | Cottoidei | Abyssocottidae | *Procottus jettelesii* | AY116353 | *CYB* |
| Scorpaeniformes | Cottoidei | Abyssocottidae | *Procottus major* | AY116322 | *ATP6* |
| Scorpaeniformes | Cottoidei | Abyssocottidae | *Procottus major* | AY116354 | *CYB* |
| Scorpaeniformes | Cottoidei | Hemitripteridae | *Blepsias cirrhosus* | GU440252 | *CO1* |
| Scorpaeniformes | Cottoidei | Hemitripteridae | *Blepsias cirrhosus* | EU836702 | *CYB* |
| Scorpaeniformes | Cottoidei | Hemitripteridae | *Hemitripterus villosus* | JF952751 | *CO1* |
| Scorpaeniformes | Cottoidei | Hemitripteridae | *Hemitripterus villosus* | AB126382 | *CYB* |
| Scorpaeniformes | Cottoidei | Hemitripteridae | *Nautichthys oculofasciatus* | FJ164899 | *CO1* |
| Scorpaeniformes | Cottoidei | Hemitripteridae | *Nautichthys oculofasciatus* | FJ264432 | *CYB* |
| Scorpaeniformes | Cottoidei | Hemitripteridae | *Nautichthys pribilovius* | HM421788 | *CO1* |
| Scorpaeniformes | Cottoidei | Hemitripteridae | *Nautichthys robustus* | FJ164901 | *CO1* |
| Scorpaeniformes | Cottoidei | Agonidae | *Agonopsis chiloensis* | EU637932 | *rhod* |
| Scorpaeniformes | Cottoidei | Agonidae | *Agonopsis sterletus* | GU440208 | *CO1* |
| Scorpaeniformes | Cottoidei | Agonidae | *Agonopsis vulsa* | FJ164244 | *CO1* |
| Scorpaeniformes | Cottoidei | Agonidae | *Agonus cataphractus* | EU492091 | *CYB* |
| Scorpaeniformes | Cottoidei | Agonidae | *Aspidophoroides monopterygius* | HM421786 | *CO1* |
| Scorpaeniformes | Cottoidei | Agonidae | *Aspidophoroides monopterygius* | AB126377 | *CYB* |
| Scorpaeniformes | Cottoidei | Agonidae | *Bathyagonus alascanus* | GU440247 | *CO1* |
| Scorpaeniformes | Cottoidei | Agonidae | *Bathyagonus alascanus* | FJ264430 | *CYB* |
| Scorpaeniformes | Cottoidei | Agonidae | *Bathyagonus infraspinatus* | FJ164352 | *CO1* |
| Scorpaeniformes | Cottoidei | Agonidae | *Bathyagonus nigripinnis* | FJ164359 | *CO1* |
| Scorpaeniformes | Cottoidei | Agonidae | *Bathyagonus nigripinnis* | FJ264479 | *CYB* |
| Scorpaeniformes | Cottoidei | Agonidae | *Bathyagonus pentacanthus* | FJ164367 | *CO1* |
| Scorpaeniformes | Cottoidei | Agonidae | *Bathyagonus pentacanthus* | FJ264423 | *CYB* |
| Scorpaeniformes | Cottoidei | Agonidae | *Chesnonia verrucosa* | GU440275 | *CO1* |
| Scorpaeniformes | Cottoidei | Agonidae | *Freemanichthys thompsoni* | AB126376 | *CYB* |
| Scorpaeniformes | Cottoidei | Agonidae | *Hypsagonus quadricornis* | FJ264435 | *CYB* |
| Scorpaeniformes | Cottoidei | Agonidae | *Leptagonus leptorhynchus* | AB126378 | *CYB* |
| Scorpaeniformes | Cottoidei | Agonidae | *Odontopyxis trispinosa* | FJ164916 | *CO1* |
| Scorpaeniformes | Cottoidei | Agonidae | *Pallasina barbata* | GU440442 | *CO1* |
| Scorpaeniformes | Cottoidei | Agonidae | *Podothecus accipenserinus* | GU440470 | *CO1* |
| Scorpaeniformes | Cottoidei | Agonidae | *Podothecus accipenserinus* | FJ264262 | *CYB* |
| Scorpaeniformes | Cottoidei | Agonidae | *Podothecus sachi* | JF952820 | *CO1* |
| Scorpaeniformes | Cottoidei | Agonidae | *Podothecus sachi* | AB126375 | *CYB* |
| Scorpaeniformes | Cottoidei | Agonidae | *Stellerina xyosterna* | GU440532 | *CO1* |
| Scorpaeniformes | Cottoidei | Agonidae | *Stellerina xyosterna* | AY973043 | *CYB* |
| Scorpaeniformes | Cottoidei | Agonidae | *Stellerina xyosterna* | EF521337 | *ND1* |
| Scorpaeniformes | Cottoidei | Agonidae | *Tilesina gibbosa* | AB126379 | *CYB* |
| Scorpaeniformes | Cottoidei | Agonidae | *Ulcina olrikii* | HM421799 | *CO1* |
| Scorpaeniformes | Cottoidei | Agonidae | *Xeneretmus latifrons* | FJ165457 | *CO1* |
| Scorpaeniformes | Cottoidei | Agonidae | *Xeneretmus latifrons* | EU638018 | *rhod* |
| Scorpaeniformes | Cottoidei | Agonidae | *Xeneretmus leiops* | GU440564 | *CO1* |
| Scorpaeniformes | Cottoidei | Psychrolutidae | *Cottunculus microps* | HM421742 | *CO1* |
| Scorpaeniformes | Cottoidei | Psychrolutidae | *Cottunculus thomsonii* | AY368315 | *rhod* |
| Scorpaeniformes | Cottoidei | Psychrolutidae | *Ebinania vermiculata* | JF952721 | *CO1* |
| Scorpaeniformes | Cottoidei | Psychrolutidae | *Malacocottus kincaidi* | FJ264476 | *CYB* |
| Scorpaeniformes | Cottoidei | Psychrolutidae | *Malacocottus zonurus* | AY116308 | *ATP6* |
| Scorpaeniformes | Cottoidei | Psychrolutidae | *Malacocottus zonurus* | FJ164832 | *CO1* |
| Scorpaeniformes | Cottoidei | Psychrolutidae | *Malacocottus zonurus* | AB126380 | *CYB* |
| Scorpaeniformes | Cottoidei | Psychrolutidae | *Malacoctenus erdmani* | GU224530 | *CO1* |
| Scorpaeniformes | Cottoidei | Psychrolutidae | *Malacoctenus macropus* | GU225382 | *CO1* |
| Scorpaeniformes | Cottoidei | Psychrolutidae | *Psychrolutes phrictus* | GU440485 | *CO1* |
| Scorpaeniformes | Cottoidei | Psychrolutidae | *Psychrolutes sigalutes* | FJ165068 | *CO1* |
| Scorpaeniformes | Cottoidei | Cyclopteridae | *Aptocyclus ventricosus* | JF952680 | *CO1* |
| Scorpaeniformes | Cottoidei | Cyclopteridae | *Cyclopterus lumpus* | AM498313 | *CO1* |
| Scorpaeniformes | Cottoidei | Cyclopteridae | *Cyclopterus lumpus* | EU492083 | *CYB* |
| Scorpaeniformes | Cottoidei | Cyclopteridae | *Cyclopterus lumpus* | AY368316 | *rhod* |
| Scorpaeniformes | Cottoidei | Cyclopteridae | *Eumicrotremus derjugini* | AM498308 | *CO1* |
| Scorpaeniformes | Cottoidei | Cyclopteridae | *Eumicrotremus derjugini* | AM498331 | *CYB* |
| Scorpaeniformes | Cottoidei | Cyclopteridae | *Eumicrotremus spinosus* | AM498301 | *CO1* |
| Scorpaeniformes | Cottoidei | Cyclopteridae | *Eumicrotremus spinosus* | AM498330 | *CYB* |
| Scorpaeniformes | Cottoidei | Liparidae | *Acantholiparis opercularis* | FJ164243 | *CO1* |
| Scorpaeniformes | Cottoidei | Liparidae | *Careproctus attenuatus* | FJ164427 | *CO1* |
| Scorpaeniformes | Cottoidei | Liparidae | *Careproctus canus* | FJ164432 | *CO1* |
| Scorpaeniformes | Cottoidei | Liparidae | *Careproctus colletti* | AB565515 | *CYB* |
| Scorpaeniformes | Cottoidei | Liparidae | *Careproctus continentalis* | HQ712898 | *CO1* |
| Scorpaeniformes | Cottoidei | Liparidae | *Careproctus cypselurus* | GU440261 | *CO1* |
| Scorpaeniformes | Cottoidei | Liparidae | *Careproctus cypselurus* | FJ264499 | *CYB* |
| Scorpaeniformes | Cottoidei | Liparidae | *Careproctus furcellus* | FJ164442 | *CO1* |
| Scorpaeniformes | Cottoidei | Liparidae | *Careproctus georgianus* | EU326326 | *CO1* |
| Scorpaeniformes | Cottoidei | Liparidae | *Careproctus longipectoralis* | HQ712899 | *CO1* |
| Scorpaeniformes | Cottoidei | Liparidae | *Careproctus melanurus* | GU440262 | *CO1* |
| Scorpaeniformes | Cottoidei | Liparidae | *Careproctus rastrinus* | JF952697 | *CO1* |
| Scorpaeniformes | Cottoidei | Liparidae | *Careproctus rastrinus* | AB565588 | *CYB* |
| Scorpaeniformes | Cottoidei | Liparidae | *Careproctus reinhardti* | EF508326 | *CYB* |
| Scorpaeniformes | Cottoidei | Liparidae | *Crystallichthys cyclospilus* | EF508330 | *CYB* |
| Scorpaeniformes | Cottoidei | Liparidae | *Edentoliparis terraenovae* | HQ712980 | *CO1* |
| Scorpaeniformes | Cottoidei | Liparidae | *Ekemblemaria myersi* | FJ381450 | *CO1* |
| Scorpaeniformes | Cottoidei | Liparidae | *Ekemblemaria myersi* | FJ381527 | *rag1* |
| Scorpaeniformes | Cottoidei | Liparidae | *Ekemblemaria myersi* | FJ381562 | *rhod* |
| Scorpaeniformes | Cottoidei | Liparidae | *Ekemblemaria nigra* | HQ654542 | *CO1* |
| Scorpaeniformes | Cottoidei | Liparidae | *Ekemblemaria nigra* | FJ381563 | *rhod* |
| Scorpaeniformes | Cottoidei | Liparidae | *Elassodiscus caudatus* | FJ164562 | *CO1* |
| Scorpaeniformes | Cottoidei | Liparidae | *Elassodiscus caudatus* | EF508329 | *CYB* |
| Scorpaeniformes | Cottoidei | Liparidae | *Elassodiscus tremebundus* | EF508328 | *CYB* |
| Scorpaeniformes | Cottoidei | Liparidae | *Liparis bathyarcticus* | HM400299 | *CO1* |
| Scorpaeniformes | Cottoidei | Liparidae | *Liparis chefuensis* | GU586125 | *CO1* |
| Scorpaeniformes | Cottoidei | Liparidae | *Liparis dennyi* | FJ164720 | *CO1* |
| Scorpaeniformes | Cottoidei | Liparidae | *Liparis dennyi* | FJ264458 | *CYB* |
| Scorpaeniformes | Cottoidei | Liparidae | *Liparis fabricii* | AM498311 | *CO1* |
| Scorpaeniformes | Cottoidei | Liparidae | *Liparis fabricii* | EF508319 | *CYB* |
| Scorpaeniformes | Cottoidei | Liparidae | *Liparis fabricii* | AY368317 | *rhod* |
| Scorpaeniformes | Cottoidei | Liparidae | *Liparis florae* | GU440375 | *CO1* |
| Scorpaeniformes | Cottoidei | Liparidae | *Liparis florae* | FJ264400 | *CYB* |
| Scorpaeniformes | Cottoidei | Liparidae | *Liparis fucensis* | GU440376 | *CO1* |
| Scorpaeniformes | Cottoidei | Liparidae | *Liparis gibbus* | AM498312 | *CO1* |
| Scorpaeniformes | Cottoidei | Liparidae | *Liparis gibbus* | AM498332 | *CYB* |
| Scorpaeniformes | Cottoidei | Liparidae | *Liparis inquilinus* | EF508321 | *CYB* |
| Scorpaeniformes | Cottoidei | Liparidae | *Liparis liparis* | EF508320 | *CYB* |
| Scorpaeniformes | Cottoidei | Liparidae | *Liparis liparis liparis* | EU492093 | *CYB* |
| Scorpaeniformes | Cottoidei | Liparidae | *Liparis montagui* | EU492339 | *CYB* |
| Scorpaeniformes | Cottoidei | Liparidae | *Liparis montagui* | EU492247 | *rhod* |
| Scorpaeniformes | Cottoidei | Liparidae | *Liparis mucosus* | GU440377 | *CO1* |
| Scorpaeniformes | Cottoidei | Liparidae | *Liparis pulchellus* | FJ164725 | *CO1* |
| Scorpaeniformes | Cottoidei | Liparidae | *Liparis pulchellus* | FJ264447 | *CYB* |
| Scorpaeniformes | Cottoidei | Liparidae | *Liparis rutteri* | FJ264467 | *CYB* |
| Scorpaeniformes | Cottoidei | Liparidae | *Liparis tanakae* | GU357851 | *CO1* |
| Scorpaeniformes | Cottoidei | Liparidae | *Liparis tunicatus* | HM400282 | *CO1* |
| Scorpaeniformes | Cottoidei | Liparidae | *Lipariscus nanus* | GU440374 | *CO1* |
| Scorpaeniformes | Cottoidei | Liparidae | *Nectoliparis pelagicus* | FJ164907 | *CO1* |
| Scorpaeniformes | Cottoidei | Liparidae | *Nectoliparis pelagicus* | EF508316 | *CYB* |
| Scorpaeniformes | Cottoidei | Liparidae | *Paraliparis antarcticus* | HQ713127 | *CO1* |
| Scorpaeniformes | Cottoidei | Liparidae | *Paraliparis bathybius* | EU326411 | *CO1* |
| Scorpaeniformes | Cottoidei | Liparidae | *Paraliparis bathybius* | EF508335 | *CYB* |
| Scorpaeniformes | Cottoidei | Liparidae | *Paraliparis charcoti* | HQ713135 | *CO1* |
| Scorpaeniformes | Cottoidei | Liparidae | *Paraliparis copei* | EF508332 | *CYB* |
| Scorpaeniformes | Cottoidei | Liparidae | *Paraliparis dactylosus* | FJ164955 | *CO1* |
| Scorpaeniformes | Cottoidei | Liparidae | *Paraliparis garmani* | EF508333 | *CYB* |
| Scorpaeniformes | Cottoidei | Liparidae | *Paraliparis leobergi* | HQ713139 | *CO1* |
| Scorpaeniformes | Cottoidei | Liparidae | *Paraliparis mawsoni* | HQ713150 | *CO1* |
| Scorpaeniformes | Cottoidei | Liparidae | *Paraliparis melanobranchius* | FJ164959 | *CO1* |
| Scorpaeniformes | Cottoidei | Liparidae | *Paraliparis paucidens* | FJ164965 | *CO1* |
| Scorpaeniformes | Cottoidei | Liparidae | *Paraliparis pectoralis* | FJ164969 | *CO1* |
| Scorpaeniformes | Cottoidei | Liparidae | *Paraliparis rosaceus* | FJ164978 | *CO1* |
| Scorpaeniformes | Cottoidei | Liparidae | *Paraliparis valentinae* | HQ713151 | *CO1* |
| Scorpaeniformes | Cottoidei | Liparidae | *Psednos groenlandicus* | EF508337 | *CYB* |
| Scorpaeniformes | Cottoidei | Liparidae | *Psednos melanocephalus* | EF508339 | *CYB* |
| Scorpaeniformes | Cottoidei | Liparidae | *Psednos micruroides* | EF508338 | *CYB* |
| Scorpaeniformes | Cottoidei | Liparidae | *Rhinoliparis attenuatus* | FJ165100 | *CO1* |
| Scorpaeniformes | Cottoidei | Liparidae | *Rhinoliparis barbulifer* | GU440505 | *CO1* |
| Scorpaeniformes | Cottoidei | Liparidae | *Rhinoliparis barbulifer* | EF508331 | *CYB* |
| Scorpaeniformes | Cottoidei | Liparidae | *Rhodichthys regina* | EF508336 | *CYB* |
| Perciformes | Percoidei | Centropomidae | *Centropomus armatus* | HQ731311 | *rag1* |
| Perciformes | Percoidei | Centropomidae | *Centropomus ensiferus* | HQ731314 | *rag1* |
| Perciformes | Percoidei | Centropomidae | *Centropomus medius* | HQ731310 | *rag1* |
| Perciformes | Percoidei | Centropomidae | *Centropomus parallelus* | EU927360 | *CYB* |
| Perciformes | Percoidei | Centropomidae | *Centropomus undecimalis* | EU752068 | *CO1* |
| Perciformes | Percoidei | Centropomidae | *Centropomus undecimalis* | AF240739 | *CYB* |
| Perciformes | Percoidei | Centropomidae | *Centropomus undecimalis* | GU368817 | *rag1* |
| Perciformes | Percoidei | Centropomidae | *Centropomus viridis* | EU167816 | *rag1* |
| Perciformes | Percoidei | Ambassidae | *Ambassis agassizii* | HM006991 | *ATP6* |
| Perciformes | Percoidei | Ambassidae | *Ambassis agassizii* | HM006949 | *CO1* |
| Perciformes | Percoidei | Ambassidae | *Ambassis agassizii* | HM007031 | *CYB* |
| Perciformes | Percoidei | Ambassidae | *Ambassis agrammus* | HQ731307 | *rag1* |
| Perciformes | Percoidei | Ambassidae | *Ambassis interrupta* | HQ731320 | *rag1* |
| Perciformes | Percoidei | Ambassidae | *Ambassis jacksoniensis* | EU257361 | *ATP6* |
| Perciformes | Percoidei | Ambassidae | *Ambassis macleayi* | HQ731308 | *rag1* |
| Perciformes | Percoidei | Ambassidae | *Ambassis marianus* | HM006992 | *ATP6* |
| Perciformes | Percoidei | Ambassidae | *Ambassis marianus* | HM006950 | *CO1* |
| Perciformes | Percoidei | Ambassidae | *Ambassis marianus* | HM007032 | *CYB* |
| Perciformes | Percoidei | Ambassidae | *Ambassis miops* | HQ654652 | *CO1* |
| Perciformes | Percoidei | Ambassidae | *Denariusa bandata* | GU474307 | *ATP6* |
| Perciformes | Percoidei | Ambassidae | *Denariusa bandata* | EU167824 | *rag1* |
| Perciformes | Percoidei | Ambassidae | *Parambassis wolffii* | EF095647 | *rag1* |
| Perciformes | Percoidei | Ambassidae | *Parambassis wolffii* | EF095612 | *rhod* |
| Perciformes | Percoidei | Ambassidae | *Pseudambassis ranga* | FJ170783 | *CO1* |
| Perciformes | Percoidei | Ambassidae | *Pseudambassis ranga* | EF095646 | *rag1* |
| Perciformes | Percoidei | Ambassidae | *Pseudambassis ranga* | EF095611 | *rhod* |
| Perciformes | Percoidei | Latidae | *Lates calcarifer* | JF919796 | *CO1* |
| Perciformes | Percoidei | Latidae | *Lates calcarifer* | JN006990 | *CYB* |
| Perciformes | Percoidei | Latidae | *Lates calcarifer* | HQ731300 | *rag1* |
| Perciformes | Percoidei | Latidae | *Lates calcarifer* | EU637970 | *rhod* |
| Perciformes | Percoidei | Latidae | *Lates japonicus* | HQ731302 | *rag1* |
| Perciformes | Percoidei | Latidae | *Lates microlepis* | HQ731303 | *rag1* |
| Perciformes | Percoidei | Latidae | *Lates niloticus* | DQ108016 | *CO1* |
| Perciformes | Percoidei | Latidae | *Lates niloticus* | AB117106 | *CYB* |
| Perciformes | Percoidei | Latidae | *Lates niloticus* | EU637971 | *rhod* |
| Perciformes | Percoidei | Latidae | *Perca fluviatilis* | AY141295 | *rhod* |
| Perciformes | Percoidei | Latidae | *Psammoperca waigiensis* | FJ237578 | *CO1* |
| Perciformes | Percoidei | Latidae | *Psammoperca waigiensis* | HQ731304 | *rag1* |
| Perciformes | Percoidei | Moronidae | *Dicentrarchus labrax* | FN689111 | *CO1* |
| Perciformes | Percoidei | Moronidae | *Dicentrarchus labrax* | EF427553 | *CYB* |
| Perciformes | Percoidei | Moronidae | *Dicentrarchus labrax* | EF095651 | *rag1* |
| Perciformes | Percoidei | Moronidae | *Dicentrarchus labrax* | EF439104 | *rhod* |
| Perciformes | Percoidei | Moronidae | *Dicentrarchus punctatus* | AF143191 | *CYB* |
| Perciformes | Percoidei | Moronidae | *Dicentrarchus punctatus* | DQ197846 | *rhod* |
| Perciformes | Percoidei | Moronidae | *Lateolabrax japonicus* | JF952774 | *CO1* |
| Perciformes | Percoidei | Moronidae | *Lateolabrax japonicus* | AB021229 | *CYB* |
| Perciformes | Percoidei | Moronidae | *Lateolabrax japonicus* | EF213745 | *ND2* |
| Perciformes | Percoidei | Moronidae | *Lateolabrax japonicus* | EF095650 | *rag1* |
| Perciformes | Percoidei | Moronidae | *Lateolabrax japonicus* | AY141293 | *rhod* |
| Perciformes | Percoidei | Moronidae | *Lateolabrax latus* | DQ351526 | *CYB* |
| Perciformes | Percoidei | Moronidae | *Lateolabrax maculatus* | EF143385 | *CO1* |
| Perciformes | Percoidei | Moronidae | *Lateolabrax maculatus* | DQ274054 | *CYB* |
| Perciformes | Percoidei | Moronidae | *Morone americana* | EU524137 | *CO1* |
| Perciformes | Percoidei | Moronidae | *Morone americana* | AF240744 | *CYB* |
| Perciformes | Percoidei | Moronidae | *Morone chrysops* | EU524142 | *CO1* |
| Perciformes | Percoidei | Moronidae | *Morone chrysops* | AF240745 | *CYB* |
| Perciformes | Percoidei | Moronidae | *Morone chrysops* | AY308767 | *rag1* |
| Perciformes | Percoidei | Moronidae | *Morone mississippiensis* | JN027260 | *CO1* |
| Perciformes | Percoidei | Moronidae | *Morone mississippiensis* | AF045362 | *CYB* |
| Perciformes | Percoidei | Moronidae | *Morone saxatilis* | EU524143 | *CO1* |
| Perciformes | Percoidei | Moronidae | *Morone saxatilis* | AF240746 | *CYB* |
| Perciformes | Percoidei | Moronidae | *Morone saxatilis* | EU637981 | *rhod* |
| Perciformes | Percoidei | Percichthyidae | *Bostockia porosa* | HQ713632 | *CYB* |
| Perciformes | Percoidei | Percichthyidae | *Bostockia porosa* | HQ713643 | *rag1* |
| Perciformes | Percoidei | Percichthyidae | *Bostockia porosa* | HQ713634 | *rag2* |
| Perciformes | Percoidei | Percichthyidae | *Bostockia porosa* | JF745773 | *rhod* |
| Perciformes | Percoidei | Percichthyidae | *Coreoperca herzi* | EF143382 | *CO1* |
| Perciformes | Percoidei | Percichthyidae | *Coreoperca herzi* | AB108489 | *CYB* |
| Perciformes | Percoidei | Percichthyidae | *Coreoperca kawamebari* | EF143383 | *CO1* |
| Perciformes | Percoidei | Percichthyidae | *Coreoperca kawamebari* | AB108487 | *CYB* |
| Perciformes | Percoidei | Percichthyidae | *Coreoperca kawamebari* | JF745771 | *rhod* |
| Perciformes | Percoidei | Percichthyidae | *Coreoperca loona* | JF745770 | *rhod* |
| Perciformes | Percoidei | Percichthyidae | *Coreoperca whiteheadi* | EF143384 | *CO1* |
| Perciformes | Percoidei | Percichthyidae | *Coreoperca whiteheadi* | AB108488 | *CYB* |
| Perciformes | Percoidei | Percichthyidae | *Coreoperca whiteheadi* | GU368824 | *rag1* |
| Perciformes | Percoidei | Percichthyidae | *Gadopsis marmoratus* | JF745772 | *rhod* |
| Perciformes | Percoidei | Percichthyidae | *Howella brodiei* | EU148199 | *CO1* |
| Perciformes | Percoidei | Percichthyidae | *Howella brodiei* | GU368831 | *rag1* |
| Perciformes | Percoidei | Percichthyidae | *Howella brodiei* | EU637966 | *rhod* |
| Perciformes | Percoidei | Percichthyidae | *Maccullochella peelii* | DQ107930 | *CO1* |
| Perciformes | Percoidei | Percichthyidae | *Macquaria ambigua* | DQ107942 | *CO1* |
| Perciformes | Percoidei | Percichthyidae | *Macquaria colonorum* | DQ107939 | *CO1* |
| Perciformes | Percoidei | Percichthyidae | *Macquaria novemaculeata* | DQ107935 | *CO1* |
| Perciformes | Percoidei | Percichthyidae | *Nannatherina balstoni* | HQ713630 | *CYB* |
| Perciformes | Percoidei | Percichthyidae | *Nannatherina balstoni* | HQ713651 | *rag1* |
| Perciformes | Percoidei | Percichthyidae | *Nannatherina balstoni* | HQ713642 | *rag2* |
| Perciformes | Percoidei | Percichthyidae | *Nannoperca australis* | HM007012 | *ATP6* |
| Perciformes | Percoidei | Percichthyidae | *Nannoperca australis* | HM006971 | *CO1* |
| Perciformes | Percoidei | Percichthyidae | *Nannoperca australis* | GQ470916 | *CYB* |
| Perciformes | Percoidei | Percichthyidae | *Nannoperca australis* | HQ713644 | *rag1* |
| Perciformes | Percoidei | Percichthyidae | *Nannoperca australis* | HQ713635 | *rag2* |
| Perciformes | Percoidei | Percichthyidae | *Nannoperca obscura* | GQ470911 | *CYB* |
| Perciformes | Percoidei | Percichthyidae | *Nannoperca obscura* | HQ713646 | *rag1* |
| Perciformes | Percoidei | Percichthyidae | *Nannoperca obscura* | HQ713637 | *rag2* |
| Perciformes | Percoidei | Percichthyidae | *Nannoperca oxleyana* | HM007013 | *ATP6* |
| Perciformes | Percoidei | Percichthyidae | *Nannoperca oxleyana* | HM006972 | *CO1* |
| Perciformes | Percoidei | Percichthyidae | *Nannoperca oxleyana* | HQ713618 | *CYB* |
| Perciformes | Percoidei | Percichthyidae | *Nannoperca oxleyana* | HQ713647 | *rag1* |
| Perciformes | Percoidei | Percichthyidae | *Nannoperca oxleyana* | HQ713638 | *rag2* |
| Perciformes | Percoidei | Percichthyidae | *Nannoperca variegata* | HQ713623 | *CYB* |
| Perciformes | Percoidei | Percichthyidae | *Nannoperca variegata* | HQ713650 | *rag1* |
| Perciformes | Percoidei | Percichthyidae | *Nannoperca variegata* | HQ713641 | *rag2* |
| Perciformes | Percoidei | Percichthyidae | *Nannoperca vittata* | HQ713626 | *CYB* |
| Perciformes | Percoidei | Percichthyidae | *Nannoperca vittata* | HQ713649 | *rag1* |
| Perciformes | Percoidei | Percichthyidae | *Nannoperca vittata* | HQ713640 | *rag2* |
| Perciformes | Percoidei | Percichthyidae | *Percichthys trucha* | GU368814 | *rag1* |
| Perciformes | Percoidei | Percichthyidae | *Siniperca chuatsi* | EF143387 | *CO1* |
| Perciformes | Percoidei | Percichthyidae | *Siniperca chuatsi* | AF475158 | *CYB* |
| Perciformes | Percoidei | Percichthyidae | *Siniperca chuatsi* | DQ862098 | *ND2* |
| Perciformes | Percoidei | Percichthyidae | *Siniperca chuatsi* | GU368825 | *rag1* |
| Perciformes | Percoidei | Percichthyidae | *Siniperca fortis* | EF143388 | *CO1* |
| Perciformes | Percoidei | Percichthyidae | *Siniperca fortis* | DQ274045 | *CYB* |
| Perciformes | Percoidei | Percichthyidae | *Siniperca knerii* | DQ345337 | *CYB* |
| Perciformes | Percoidei | Percichthyidae | *Siniperca knerii* | GU368826 | *rag1* |
| Perciformes | Percoidei | Percichthyidae | *Siniperca obscura* | EF143390 | *CO1* |
| Perciformes | Percoidei | Percichthyidae | *Siniperca obscura* | DQ345339 | *CYB* |
| Perciformes | Percoidei | Percichthyidae | *Siniperca obscura* | GU368827 | *rag1* |
| Perciformes | Percoidei | Percichthyidae | *Siniperca roulei* | EF143381 | *CO1* |
| Perciformes | Percoidei | Percichthyidae | *Siniperca roulei* | DQ345341 | *CYB* |
| Perciformes | Percoidei | Percichthyidae | *Siniperca roulei* | GU368828 | *rag1* |
| Perciformes | Percoidei | Percichthyidae | *Siniperca scherzeri* | EF143391 | *CO1* |
| Perciformes | Percoidei | Percichthyidae | *Siniperca scherzeri* | DQ345338 | *CYB* |
| Perciformes | Percoidei | Percichthyidae | *Siniperca scherzeri* | GU368829 | *rag1* |
| Perciformes | Percoidei | Percichthyidae | *Siniperca undulata* | EF143393 | *CO1* |
| Perciformes | Percoidei | Percichthyidae | *Siniperca undulata* | DQ345340 | *CYB* |
| Perciformes | Percoidei | Percichthyidae | *Siniperca undulata* | GU368830 | *rag1* |
| Perciformes | Percoidei | Acropomatidae | *Acropoma hanedai* | DQ648436 | *CO1* |
| Perciformes | Percoidei | Acropomatidae | *Acropoma japonicum* | DQ648437 | *CO1* |
| Perciformes | Percoidei | Acropomatidae | *Malakichthys wakiyae* | AB108495 | *CYB* |
| Perciformes | Percoidei | Acropomatidae | *Synagrops bellus* | EU182622 | *rag1* |
| Perciformes | Percoidei | Acropomatidae | *Synagrops bellus* | JF913271 | *rhod* |
| Perciformes | Percoidei | Acropomatidae | *Synagrops japonicus* | JF952871 | *CO1* |
| Perciformes | Percoidei | Serranidae | *Acanthistius joanae* | HQ945945 | *CO1* |
| Perciformes | Percoidei | Serranidae | *Acanthistius patachonicus* | EU074305 | *CO1* |
| Perciformes | Percoidei | Serranidae | *Aethaloperca rogaa* | JN021291 | *CO1* |
| Perciformes | Percoidei | Serranidae | *Aethaloperca rogaa* | EF213721 | *CYB* |
| Perciformes | Percoidei | Serranidae | *Aethaloperca rogaa* | EF213731 | *ND2* |
| Perciformes | Percoidei | Serranidae | *Anthias nicholsi* | HQ731316 | *rag1* |
| Perciformes | Percoidei | Serranidae | *Anyperodon leucogrammicus* | DQ107922 | *CO1* |
| Perciformes | Percoidei | Serranidae | *Anyperodon leucogrammicus* | AY963557 | *CYB* |
| Perciformes | Percoidei | Serranidae | *Anyperodon leucogrammicus* | EF213733 | *ND2* |
| Perciformes | Percoidei | Serranidae | *Aulacocephalus temminckii* | HQ945855 | *CO1* |
| Perciformes | Percoidei | Serranidae | *Caprodon longimanus* | DQ107893 | *CO1* |
| Perciformes | Percoidei | Serranidae | *Caprodon unicolor* | DQ521005 | *CO1* |
| Perciformes | Percoidei | Serranidae | *Centropristis striata* | JN021294 | *CO1* |
| Perciformes | Percoidei | Serranidae | *Cephalopholis argus* | FJ237556 | *CO1* |
| Perciformes | Percoidei | Serranidae | *Cephalopholis argus* | JN157728 | *CYB* |
| Perciformes | Percoidei | Serranidae | *Cephalopholis argus* | DQ862092 | *ND2* |
| Perciformes | Percoidei | Serranidae | *Cephalopholis boenak* | FJ237553 | *CO1* |
| Perciformes | Percoidei | Serranidae | *Cephalopholis boenak* | AF121225 | *CYB* |
| Perciformes | Percoidei | Serranidae | *Cephalopholis cruentata* | GU225176 | *CO1* |
| Perciformes | Percoidei | Serranidae | *Cephalopholis cyanostigma* | DQ107918 | *CO1* |
| Perciformes | Percoidei | Serranidae | *Cephalopholis formosa* | FJ583004 | *CO1* |
| Perciformes | Percoidei | Serranidae | *Cephalopholis formosa* | AY950691 | *CYB* |
| Perciformes | Percoidei | Serranidae | *Cephalopholis formosa* | DQ862093 | *ND2* |
| Perciformes | Percoidei | Serranidae | *Cephalopholis fulva* | EU752070 | *CO1* |
| Perciformes | Percoidei | Serranidae | *Cephalopholis hemistiktos* | HQ149822 | *CO1* |
| Perciformes | Percoidei | Serranidae | *Cephalopholis igarashiensis* | EU871685 | *CO1* |
| Perciformes | Percoidei | Serranidae | *Cephalopholis leopardus* | FJ583010 | *CO1* |
| Perciformes | Percoidei | Serranidae | *Cephalopholis microprion* | FJ237608 | *CO1* |
| Perciformes | Percoidei | Serranidae | *Cephalopholis miniata* | DQ107924 | *CO1* |
| Perciformes | Percoidei | Serranidae | *Cephalopholis miniata* | EF213724 | *CYB* |
| Perciformes | Percoidei | Serranidae | *Cephalopholis miniata* | EU930862 | *ND1* |
| Perciformes | Percoidei | Serranidae | *Cephalopholis miniata* | EF213729 | *ND2* |
| Perciformes | Percoidei | Serranidae | *Cephalopholis pachycentron* | AY738232 | *CYB* |
| Perciformes | Percoidei | Serranidae | *Cephalopholis pachycentron* | DQ862091 | *ND2* |
| Perciformes | Percoidei | Serranidae | *Cephalopholis sexmaculata* | DQ107923 | *CO1* |
| Perciformes | Percoidei | Serranidae | *Cephalopholis sonnerati* | DQ107926 | *CO1* |
| Perciformes | Percoidei | Serranidae | *Cephalopholis sonnerati* | EF213722 | *CYB* |
| Perciformes | Percoidei | Serranidae | *Cephalopholis sonnerati* | DQ862094 | *ND2* |
| Perciformes | Percoidei | Serranidae | *Cephalopholis taeniops* | EF455990 | *CYB* |
| Perciformes | Percoidei | Serranidae | *Cephalopholis taeniops* | HM050093 | *ND4* |
| Perciformes | Percoidei | Serranidae | *Cephalopholis taeniops* | EF456039 | *rhod* |
| Perciformes | Percoidei | Serranidae | *Cephalopholis urodeta* | FJ583014 | *CO1* |
| Perciformes | Percoidei | Serranidae | *Cephalopholis urodeta* | AY786426 | *CYB* |
| Perciformes | Percoidei | Serranidae | *Cephalopholis urodeta* | EF213730 | *ND2* |
| Perciformes | Percoidei | Serranidae | *Cromileptes altivelis* | DQ107892 | *CO1* |
| Perciformes | Percoidei | Serranidae | *Cromileptes altivelis* | DQ683362 | *CYB* |
| Perciformes | Percoidei | Serranidae | *Cromileptes altivelis* | DQ862090 | *ND2* |
| Perciformes | Percoidei | Serranidae | *Cromileptes altivelis* | EU167823 | *rag1* |
| Perciformes | Percoidei | Serranidae | *Dermatolepis dermatolepis* | HM032025 | *CO1* |
| Perciformes | Percoidei | Serranidae | *Dermatolepis dermatolepis* | AY314000 | *CYB* |
| Perciformes | Percoidei | Serranidae | *Dermatolepis inermis* | EU752075 | *CO1* |
| Perciformes | Percoidei | Serranidae | *Dermatolepis inermis* | AY314001 | *CYB* |
| Perciformes | Percoidei | Serranidae | *Dermatolepis striolata* | AY313999 | *CYB* |
| Perciformes | Percoidei | Serranidae | *Diplectrum formosum* | AY662750 | *CO1* |
| Perciformes | Percoidei | Serranidae | *Diplectrum formosum* | AY321798 | *CYB* |
| Perciformes | Percoidei | Serranidae | *Epinephelus acanthistius* | HQ010051 | *CO1* |
| Perciformes | Percoidei | Serranidae | *Epinephelus adscensionis* | FJ583396 | *CO1* |
| Perciformes | Percoidei | Serranidae | *Epinephelus aeneus* | GU199324 | *CYB* |
| Perciformes | Percoidei | Serranidae | *Epinephelus aeneus* | HM050103 | *ND4* |
| Perciformes | Percoidei | Serranidae | *Epinephelus aeneus* | AY141291 | *rhod* |
| Perciformes | Percoidei | Serranidae | *Epinephelus akaara* | EU600147 | *CO1* |
| Perciformes | Percoidei | Serranidae | *Epinephelus akaara* | AY786420 | *CYB* |
| Perciformes | Percoidei | Serranidae | *Epinephelus akaara* | DQ862067 | *ND2* |
| Perciformes | Percoidei | Serranidae | *Epinephelus albomarginatus* | GU804970 | *CO1* |
| Perciformes | Percoidei | Serranidae | *Epinephelus amblycephalus* | FJ237742 | *CO1* |
| Perciformes | Percoidei | Serranidae | *Epinephelus amblycephalus* | AY738236 | *CYB* |
| Perciformes | Percoidei | Serranidae | *Epinephelus amblycephalus* | DQ862068 | *ND2* |
| Perciformes | Percoidei | Serranidae | *Epinephelus areolatus* | DQ107870 | *CO1* |
| Perciformes | Percoidei | Serranidae | *Epinephelus areolatus* | AY786421 | *CYB* |
| Perciformes | Percoidei | Serranidae | *Epinephelus areolatus* | DQ862080 | *ND2* |
| Perciformes | Percoidei | Serranidae | *Epinephelus awoara* | AB108494 | *CYB* |
| Perciformes | Percoidei | Serranidae | *Epinephelus awoara* | DQ862069 | *ND2* |
| Perciformes | Percoidei | Serranidae | *Epinephelus bleekeri* | JN021297 | *CO1* |
| Perciformes | Percoidei | Serranidae | *Epinephelus bleekeri* | AY963558 | *CYB* |
| Perciformes | Percoidei | Serranidae | *Epinephelus bleekeri* | DQ862070 | *ND2* |
| Perciformes | Percoidei | Serranidae | *Epinephelus bruneus* | AY950700 | *CYB* |
| Perciformes | Percoidei | Serranidae | *Epinephelus bruneus* | JF501965 | *ND2* |
| Perciformes | Percoidei | Serranidae | *Epinephelus caninus* | AJ420204 | *CYB* |
| Perciformes | Percoidei | Serranidae | *Epinephelus chlorostigma* | EU392203 | *CO1* |
| Perciformes | Percoidei | Serranidae | *Epinephelus chlorostigma* | AY738239 | *CYB* |
| Perciformes | Percoidei | Serranidae | *Epinephelus chlorostigma* | DQ862071 | *ND2* |
| Perciformes | Percoidei | Serranidae | *Epinephelus clippertonensis* | DQ007242 | *CYB* |
| Perciformes | Percoidei | Serranidae | *Epinephelus coeruleopunctatus* | EF213717 | *CYB* |
| Perciformes | Percoidei | Serranidae | *Epinephelus coeruleopunctatus* | EF213734 | *ND2* |
| Perciformes | Percoidei | Serranidae | *Epinephelus coioides* | DQ107879 | *CO1* |
| Perciformes | Percoidei | Serranidae | *Epinephelus coioides* | DQ354156 | *CYB* |
| Perciformes | Percoidei | Serranidae | *Epinephelus coioides* | DQ862072 | *ND2* |
| Perciformes | Percoidei | Serranidae | *Epinephelus coioides* | FJ426122 | *rhod* |
| Perciformes | Percoidei | Serranidae | *Epinephelus costae* | EU036439 | *CYB* |
| Perciformes | Percoidei | Serranidae | *Epinephelus costae* | EF456050 | *rhod* |
| Perciformes | Percoidei | Serranidae | *Epinephelus daemelii* | EF644431 | *CYB* |
| Perciformes | Percoidei | Serranidae | *Epinephelus daemelii* | EF644434 | *ND2* |
| Perciformes | Percoidei | Serranidae | *Epinephelus diacanthus* | HQ589272 | *CO1* |
| Perciformes | Percoidei | Serranidae | *Epinephelus diacanthus* | AY950699 | *CYB* |
| Perciformes | Percoidei | Serranidae | *Epinephelus diacanthus* | DQ862083 | *ND2* |
| Perciformes | Percoidei | Serranidae | *Epinephelus drummondhayi* | AY313997 | *CYB* |
| Perciformes | Percoidei | Serranidae | *Epinephelus epistictus* | FJ237768 | *CO1* |
| Perciformes | Percoidei | Serranidae | *Epinephelus epistictus* | AY738241 | *CYB* |
| Perciformes | Percoidei | Serranidae | *Epinephelus epistictus* | DQ862073 | *ND2* |
| Perciformes | Percoidei | Serranidae | *Epinephelus ergastularius* | DQ107882 | *CO1* |
| Perciformes | Percoidei | Serranidae | *Epinephelus fario* | EU600142 | *CO1* |
| Perciformes | Percoidei | Serranidae | *Epinephelus fario* | DQ486930 | *CYB* |
| Perciformes | Percoidei | Serranidae | *Epinephelus fario* | DQ862074 | *ND2* |
| Perciformes | Percoidei | Serranidae | *Epinephelus fasciatomaculosus* | EF607565 | *CO1* |
| Perciformes | Percoidei | Serranidae | *Epinephelus fasciatomaculosus* | EF213719 | *CYB* |
| Perciformes | Percoidei | Serranidae | *Epinephelus fasciatus* | EU600146 | *CO1* |
| Perciformes | Percoidei | Serranidae | *Epinephelus fasciatus* | AY786423 | *CYB* |
| Perciformes | Percoidei | Serranidae | *Epinephelus fasciatus* | AB063197 | *ND2* |
| Perciformes | Percoidei | Serranidae | *Epinephelus flavocaeruleus* | EF503635 | *CYB* |
| Perciformes | Percoidei | Serranidae | *Epinephelus flavocaeruleus* | EF213737 | *ND2* |
| Perciformes | Percoidei | Serranidae | *Epinephelus fuscoguttatus* | HQ174860 | *CO1* |
| Perciformes | Percoidei | Serranidae | *Epinephelus fuscoguttatus* | HQ174885 | *CYB* |
| Perciformes | Percoidei | Serranidae | *Epinephelus fuscoguttatus* | DQ862079 | *ND2* |
| Perciformes | Percoidei | Serranidae | *Epinephelus guttatus* | JN021299 | *CO1* |
| Perciformes | Percoidei | Serranidae | *Epinephelus guttatus* | DQ874793 | *rag2* |
| Perciformes | Percoidei | Serranidae | *Epinephelus guttatus* | DQ874825 | *rhod* |
| Perciformes | Percoidei | Serranidae | *Epinephelus haifensis* | AJ420207 | *CYB* |
| Perciformes | Percoidei | Serranidae | *Epinephelus hexagonatus* | AB063198 | *ND2* |
| Perciformes | Percoidei | Serranidae | *Epinephelus itajara* | JN021300 | *CO1* |
| Perciformes | Percoidei | Serranidae | *Epinephelus itajara* | EU823103 | *CYB* |
| Perciformes | Percoidei | Serranidae | *Epinephelus labriformis* | AY728125 | *CYB* |
| Perciformes | Percoidei | Serranidae | *Epinephelus lanceolatus* | HQ174832 | *CO1* |
| Perciformes | Percoidei | Serranidae | *Epinephelus lanceolatus* | HQ174843 | *CYB* |
| Perciformes | Percoidei | Serranidae | *Epinephelus lanceolatus* | DQ862089 | *ND2* |
| Perciformes | Percoidei | Serranidae | *Epinephelus latifasciatus* | EU014216 | *CO1* |
| Perciformes | Percoidei | Serranidae | *Epinephelus latifasciatus* | AY738243 | *CYB* |
| Perciformes | Percoidei | Serranidae | *Epinephelus latifasciatus* | DQ862075 | *ND2* |
| Perciformes | Percoidei | Serranidae | *Epinephelus longispinis* | EF609522 | *CO1* |
| Perciformes | Percoidei | Serranidae | *Epinephelus longispinis* | AY950697 | *CYB* |
| Perciformes | Percoidei | Serranidae | *Epinephelus longispinis* | DQ862081 | *ND2* |
| Perciformes | Percoidei | Serranidae | *Epinephelus macrospilos* | HQ561520 | *CO1* |
| Perciformes | Percoidei | Serranidae | *Epinephelus maculatus* | JN021301 | *CO1* |
| Perciformes | Percoidei | Serranidae | *Epinephelus maculatus* | HQ731317 | *rag1* |
| Perciformes | Percoidei | Serranidae | *Epinephelus malabaricus* | FJ237599 | *CO1* |
| Perciformes | Percoidei | Serranidae | *Epinephelus malabaricus* | AY738244 | *CYB* |
| Perciformes | Percoidei | Serranidae | *Epinephelus malabaricus* | DQ862076 | *ND2* |
| Perciformes | Percoidei | Serranidae | *Epinephelus malabaricus* | AY551565 | *rag1* |
| Perciformes | Percoidei | Serranidae | *Epinephelus marginatus* | HQ611093 | *CO1* |
| Perciformes | Percoidei | Serranidae | *Epinephelus marginatus* | EU264009 | *CYB* |
| Perciformes | Percoidei | Serranidae | *Epinephelus marginatus* | DQ197854 | *rhod* |
| Perciformes | Percoidei | Serranidae | *Epinephelus melanostigma* | EF503634 | *CYB* |
| Perciformes | Percoidei | Serranidae | *Epinephelus melanostigma* | EF213736 | *ND2* |
| Perciformes | Percoidei | Serranidae | *Epinephelus merra* | FJ237598 | *CO1* |
| Perciformes | Percoidei | Serranidae | *Epinephelus merra* | AY786424 | *CYB* |
| Perciformes | Percoidei | Serranidae | *Epinephelus moara* | EU266383 | *CO1* |
| Perciformes | Percoidei | Serranidae | *Epinephelus moara* | AY786427 | *CYB* |
| Perciformes | Percoidei | Serranidae | *Epinephelus moara* | DQ862077 | *ND2* |
| Perciformes | Percoidei | Serranidae | *Epinephelus morio* | JF421453 | *CO1* |
| Perciformes | Percoidei | Serranidae | *Epinephelus morrhua* | DQ107897 | *CO1* |
| Perciformes | Percoidei | Serranidae | *Epinephelus multinotatus* | DQ107886 | *CO1* |
| Perciformes | Percoidei | Serranidae | *Epinephelus multinotatus* | AY426254 | *CYB* |
| Perciformes | Percoidei | Serranidae | *Epinephelus ongus* | DQ107858 | *CO1* |
| Perciformes | Percoidei | Serranidae | *Epinephelus poecilonotus* | FJ237769 | *CO1* |
| Perciformes | Percoidei | Serranidae | *Epinephelus polyphekadion* | EF213716 | *CYB* |
| Perciformes | Percoidei | Serranidae | *Epinephelus polyphekadion* | EF213735 | *ND2* |
| Perciformes | Percoidei | Serranidae | *Epinephelus polyphekadion* | AY279869 | *rag2* |
| Perciformes | Percoidei | Serranidae | *Epinephelus quoyanus* | DQ107863 | *CO1* |
| Perciformes | Percoidei | Serranidae | *Epinephelus quoyanus* | FJ493230 | *CYB* |
| Perciformes | Percoidei | Serranidae | *Epinephelus quoyanus* | DQ862086 | *ND2* |
| Perciformes | Percoidei | Serranidae | *Epinephelus radiatus* | AY950701 | *CYB* |
| Perciformes | Percoidei | Serranidae | *Epinephelus radiatus* | DQ862085 | *ND2* |
| Perciformes | Percoidei | Serranidae | *Epinephelus rivulatus* | DQ885006 | *CO1* |
| Perciformes | Percoidei | Serranidae | *Epinephelus septemfasciatus* | DQ107865 | *CO1* |
| Perciformes | Percoidei | Serranidae | *Epinephelus septemfasciatus* | AY786425 | *CYB* |
| Perciformes | Percoidei | Serranidae | *Epinephelus sexfasciatus* | EF607564 | *CO1* |
| Perciformes | Percoidei | Serranidae | *Epinephelus sexfasciatus* | AY738248 | *CYB* |
| Perciformes | Percoidei | Serranidae | *Epinephelus sexfasciatus* | DQ862078 | *ND2* |
| Perciformes | Percoidei | Serranidae | *Epinephelus spilotoceps* | FJ237775 | *CO1* |
| Perciformes | Percoidei | Serranidae | *Epinephelus spilotoceps* | AY963559 | *CYB* |
| Perciformes | Percoidei | Serranidae | *Epinephelus spilotoceps* | DQ862087 | *ND2* |
| Perciformes | Percoidei | Serranidae | *Epinephelus stictus* | EF213718 | *CYB* |
| Perciformes | Percoidei | Serranidae | *Epinephelus tauvina* | EU148566 | *CO1* |
| Perciformes | Percoidei | Serranidae | *Epinephelus tauvina* | EF456003 | *CYB* |
| Perciformes | Percoidei | Serranidae | *Epinephelus tauvina* | EF213738 | *ND2* |
| Perciformes | Percoidei | Serranidae | *Epinephelus tauvina* | EF456052 | *rhod* |
| Perciformes | Percoidei | Serranidae | *Epinephelus tukula* | EF213715 | *CYB* |
| Perciformes | Percoidei | Serranidae | *Epinephelus tukula* | EF213732 | *ND2* |
| Perciformes | Percoidei | Serranidae | *Epinephelus undulosus* | EF609352 | *CO1* |
| Perciformes | Percoidei | Serranidae | *Epinephelus undulosus* | AY950698 | *CYB* |
| Perciformes | Percoidei | Serranidae | *Epinephelus undulosus* | DQ862082 | *ND2* |
| Perciformes | Percoidei | Serranidae | *Grammistes sexlineatus* | GU805084 | *CO1* |
| Perciformes | Percoidei | Serranidae | *Grammistes sexlineatus* | HQ731319 | *rag1* |
| Perciformes | Percoidei | Serranidae | *Hemanthias signifer* | GU440335 | *CO1* |
| Perciformes | Percoidei | Serranidae | *Hypoplectrus aberrans* | AY321853 | *ATP6* |
| Perciformes | Percoidei | Serranidae | *Hypoplectrus aberrans* | AJ544136 | *CYB* |
| Perciformes | Percoidei | Serranidae | *Hypoplectrus chlorurus* | AY321865 | *ATP6* |
| Perciformes | Percoidei | Serranidae | *Hypoplectrus chlorurus* | AY321774 | *CYB* |
| Perciformes | Percoidei | Serranidae | *Hypoplectrus gummigutta* | AJ544159 | *CYB* |
| Perciformes | Percoidei | Serranidae | *Hypoplectrus guttavarius* | AY321849 | *ATP6* |
| Perciformes | Percoidei | Serranidae | *Hypoplectrus guttavarius* | AJ544155 | *CYB* |
| Perciformes | Percoidei | Serranidae | *Hypoplectrus indigo* | AY321847 | *ATP6* |
| Perciformes | Percoidei | Serranidae | *Hypoplectrus indigo* | AJ544139 | *CYB* |
| Perciformes | Percoidei | Serranidae | *Hypoplectrus nigricans* | AJ544150 | *CYB* |
| Perciformes | Percoidei | Serranidae | *Hypoplectrus puella* | AY321809 | *ATP6* |
| Perciformes | Percoidei | Serranidae | *Hypoplectrus puella* | FJ583581 | *CO1* |
| Perciformes | Percoidei | Serranidae | *Hypoplectrus puella* | AJ544149 | *CYB* |
| Perciformes | Percoidei | Serranidae | *Hypoplectrus unicolor* | AY321829 | *ATP6* |
| Perciformes | Percoidei | Serranidae | *Hypoplectrus unicolor* | AY321737 | *CYB* |
| Perciformes | Percoidei | Serranidae | *Liopropoma rubre* | HQ731318 | *rag1* |
| Perciformes | Percoidei | Serranidae | *Meganthias natalensis* | GU805020 | *CO1* |
| Perciformes | Percoidei | Serranidae | *Mycteroperca bonaci* | GU225648 | *CO1* |
| Perciformes | Percoidei | Serranidae | *Mycteroperca fusca* | DQ197870 | *rhod* |
| Perciformes | Percoidei | Serranidae | *Mycteroperca interstitialis* | FJ583668 | *CO1* |
| Perciformes | Percoidei | Serranidae | *Mycteroperca jordani* | GU440412 | *CO1* |
| Perciformes | Percoidei | Serranidae | *Mycteroperca marginata* | JF421454 | *CO1* |
| Perciformes | Percoidei | Serranidae | *Mycteroperca microlepis* | JN021310 | *CO1* |
| Perciformes | Percoidei | Serranidae | *Mycteroperca rubra* | GU199327 | *CYB* |
| Perciformes | Percoidei | Serranidae | *Mycteroperca rubra* | DQ197871 | *rhod* |
| Perciformes | Percoidei | Serranidae | *Mycteroperca xenarcha* | GU440413 | *CO1* |
| Perciformes | Percoidei | Serranidae | *Nemanthias carberryi* | FJ583698 | *CO1* |
| Perciformes | Percoidei | Serranidae | *Niphon spinosus* | EF143386 | *CO1* |
| Perciformes | Percoidei | Serranidae | *Niphon spinosus* | AB108493 | *CYB* |
| Perciformes | Percoidei | Serranidae | *Niphon spinosus* | HQ731313 | *rag1* |
| Perciformes | Percoidei | Serranidae | *Niphon spinosus* | EU637934 | *rhod* |
| Perciformes | Percoidei | Serranidae | *Odontanthias borbonius* | HQ945920 | *CO1* |
| Perciformes | Percoidei | Serranidae | *Odontanthias elizabethae* | DQ521008 | *CO1* |
| Perciformes | Percoidei | Serranidae | *Paralabrax auroguttatus* | GU440444 | *CO1* |
| Perciformes | Percoidei | Serranidae | *Paralabrax clathratus* | GU440445 | *CO1* |
| Perciformes | Percoidei | Serranidae | *Paralabrax maculatofasciatus* | GU440446 | *CO1* |
| Perciformes | Percoidei | Serranidae | *Paralabrax nebulifer* | HQ010087 | *CO1* |
| Perciformes | Percoidei | Serranidae | *Paralabrax nebulifer* | AY313998 | *CYB* |
| Perciformes | Percoidei | Serranidae | *Paranthias colonus* | GU440449 | *CO1* |
| Perciformes | Percoidei | Serranidae | *Paranthias colonus* | HM049960 | *CYB* |
| Perciformes | Percoidei | Serranidae | *Paranthias colonus* | HM050126 | *ND4* |
| Perciformes | Percoidei | Serranidae | *Plectropomus areolatus* | EF213725 | *CYB* |
| Perciformes | Percoidei | Serranidae | *Plectropomus areolatus* | EF213741 | *ND2* |
| Perciformes | Percoidei | Serranidae | *Plectropomus laevis* | DQ107908 | *CO1* |
| Perciformes | Percoidei | Serranidae | *Plectropomus laevis* | AY963554 | *CYB* |
| Perciformes | Percoidei | Serranidae | *Plectropomus laevis* | DQ862097 | *ND2* |
| Perciformes | Percoidei | Serranidae | *Plectropomus leopardus* | JF750763 | *CO1* |
| Perciformes | Percoidei | Serranidae | *Plectropomus leopardus* | AY963556 | *CYB* |
| Perciformes | Percoidei | Serranidae | *Plectropomus leopardus* | DQ862096 | *ND2* |
| Perciformes | Percoidei | Serranidae | *Plectropomus maculatus* | JF750764 | *CO1* |
| Perciformes | Percoidei | Serranidae | *Plectropomus maculatus* | EF503636 | *CYB* |
| Perciformes | Percoidei | Serranidae | *Plectropomus maculatus* | EF213743 | *ND2* |
| Perciformes | Percoidei | Serranidae | *Plectropomus oligacanthus* | EF213726 | *CYB* |
| Perciformes | Percoidei | Serranidae | *Plectropomus oligacanthus* | AF092409 | *ND2* |
| Perciformes | Percoidei | Serranidae | *Plectropomus pessuliferus* | AY963553 | *CYB* |
| Perciformes | Percoidei | Serranidae | *Pogonoperca punctata* | AY141292 | *rhod* |
| Perciformes | Percoidei | Serranidae | *Pseudanthias cooperi* | GU805058 | *CO1* |
| Perciformes | Percoidei | Serranidae | *Pseudanthias huchtii* | FJ583925 | *CO1* |
| Perciformes | Percoidei | Serranidae | *Pseudanthias lori* | FJ583929 | *CO1* |
| Perciformes | Percoidei | Serranidae | *Pseudanthias pascalus* | FJ583933 | *CO1* |
| Perciformes | Percoidei | Serranidae | *Pseudanthias pleurotaenia* | FJ583936 | *CO1* |
| Perciformes | Percoidei | Serranidae | *Pseudanthias squamipinnis* | FJ583955 | *CO1* |
| Perciformes | Percoidei | Serranidae | *Pseudanthias truncatus* | FJ583959 | *CO1* |
| Perciformes | Percoidei | Serranidae | *Pseudogramma gregoryi* | GU225013 | *CO1* |
| Perciformes | Percoidei | Serranidae | *Rypticus bicolor* | AY321887 | *ATP6* |
| Perciformes | Percoidei | Serranidae | *Rypticus bicolor* | AY321801 | *CYB* |
| Perciformes | Percoidei | Serranidae | *Rypticus saponaceus* | AY321885 | *ATP6* |
| Perciformes | Percoidei | Serranidae | *Rypticus saponaceus* | AY321799 | *CYB* |
| Perciformes | Percoidei | Serranidae | *Rypticus saponaceus* | AY368329 | *rhod* |
| Perciformes | Percoidei | Serranidae | *Serranocirrhitus latus* | FJ584096 | *CO1* |
| Perciformes | Percoidei | Serranidae | *Serranus accraensis* | AY141289 | *rhod* |
| Perciformes | Percoidei | Serranidae | *Serranus atricauda* | EF439229 | *CYB* |
| Perciformes | Percoidei | Serranidae | *Serranus atricauda* | DQ197901 | *rhod* |
| Perciformes | Percoidei | Serranidae | *Serranus baldwini* | AY321875 | *ATP6* |
| Perciformes | Percoidei | Serranidae | *Serranus baldwini* | GU225037 | *CO1* |
| Perciformes | Percoidei | Serranidae | *Serranus baldwini* | AY321784 | *CYB* |
| Perciformes | Percoidei | Serranidae | *Serranus baldwini* | HQ731315 | *rag1* |
| Perciformes | Percoidei | Serranidae | *Serranus cabrilla* | HQ945917 | *CO1* |
| Perciformes | Percoidei | Serranidae | *Serranus cabrilla* | EU036496 | *CYB* |
| Perciformes | Percoidei | Serranidae | *Serranus cabrilla* | DQ197902 | *rhod* |
| Perciformes | Percoidei | Serranidae | *Serranus hepatus* | FN689016 | *CO1* |
| Perciformes | Percoidei | Serranidae | *Serranus hepatus* | EU036500 | *CYB* |
| Perciformes | Percoidei | Serranidae | *Serranus hepatus* | EU036603 | *rhod* |
| Perciformes | Percoidei | Serranidae | *Serranus phoebe* | AY321789 | *CYB* |
| Perciformes | Percoidei | Serranidae | *Serranus psittacinus* | AY321877 | *ATP6* |
| Perciformes | Percoidei | Serranidae | *Serranus psittacinus* | AY321786 | *CYB* |
| Perciformes | Percoidei | Serranidae | *Serranus scriba* | FN689291 | *CO1* |
| Perciformes | Percoidei | Serranidae | *Serranus scriba* | EF439589 | *CYB* |
| Perciformes | Percoidei | Serranidae | *Serranus scriba* | EF439450 | *rhod* |
| Perciformes | Percoidei | Serranidae | *Serranus tabacarius* | AY321879 | *ATP6* |
| Perciformes | Percoidei | Serranidae | *Serranus tabacarius* | FJ584104 | *CO1* |
| Perciformes | Percoidei | Serranidae | *Serranus tabacarius* | AY321792 | *CYB* |
| Perciformes | Percoidei | Serranidae | *Serranus tigrinus* | AY321880 | *ATP6* |
| Perciformes | Percoidei | Serranidae | *Serranus tigrinus* | FJ584106 | *CO1* |
| Perciformes | Percoidei | Serranidae | *Serranus tigrinus* | AY321793 | *CYB* |
| Perciformes | Percoidei | Serranidae | *Serranus tortugarum* | AY321884 | *ATP6* |
| Perciformes | Percoidei | Serranidae | *Serranus tortugarum* | AY321797 | *CYB* |
| Perciformes | Percoidei | Serranidae | *Tosana niwae* | JF952878 | *CO1* |
| Perciformes | Percoidei | Serranidae | *Triso dermopterus* | DQ107934 | *CO1* |
| Perciformes | Percoidei | Serranidae | *Variola albimarginata* | FJ584241 | *CO1* |
| Perciformes | Percoidei | Serranidae | *Variola albimarginata* | EF213728 | *CYB* |
| Perciformes | Percoidei | Serranidae | *Variola albimarginata* | EF213744 | *ND2* |
| Perciformes | Percoidei | Serranidae | *Variola louti* | DQ107957 | *CO1* |
| Perciformes | Percoidei | Serranidae | *Variola louti* | AY786428 | *CYB* |
| Perciformes | Percoidei | Serranidae | *Variola louti* | DQ862095 | *ND2* |
| Perciformes | Percoidei | Ostracoberycidae | *Ostracoberyx dorygenys* | EU167855 | *rag1* |
| Perciformes | Percoidei | Callanthiidae | *Callanthias australis* | EU167814 | *rag1* |
| Perciformes | Percoidei | Callanthiidae | *Callanthias ruber* | EU637945 | *rhod* |
| Perciformes | Percoidei | Pseudochromidae | *Labracinus cyclophthalmus* | FJ583585 | *CO1* |
| Perciformes | Percoidei | Pseudochromidae | *Labracinus lineatus* | FJ583586 | *CO1* |
| Perciformes | Percoidei | Pseudochromidae | *Pictichromis coralensis* | FJ851311 | *CO1* |
| Perciformes | Percoidei | Pseudochromidae | *Pictichromis diadema* | FJ583978 | *CO1* |
| Perciformes | Percoidei | Pseudochromidae | *Pictichromis dinar* | FJ851319 | *CO1* |
| Perciformes | Percoidei | Pseudochromidae | *Pictichromis paccagnellae* | FJ583984 | *CO1* |
| Perciformes | Percoidei | Pseudochromidae | *Pictichromis porphyrea* | FJ583989 | *CO1* |
| Perciformes | Percoidei | Pseudochromidae | *Pseudochromis bitaeniatus* | EU167867 | *rag1* |
| Perciformes | Percoidei | Pseudochromidae | *Pseudochromis fuscus* | FJ583981 | *CO1* |
| Perciformes | Percoidei | Pseudochromidae | *Pseudochromis natalensis* | GU805120 | *CO1* |
| Perciformes | Percoidei | Pseudochromidae | *Pseudochromis olivaceus* | FJ851324 | *CO1* |
| Perciformes | Percoidei | Grammatidae | *Gramma loreto* | AY662751 | *CO1* |
| Perciformes | Percoidei | Grammatidae | *Gramma loreto* | EU167836 | *rag1* |
| Perciformes | Percoidei | Grammatidae | *Gramma melacara* | FJ583481 | *CO1* |
| Perciformes | Percoidei | Plesiopidae | *Assessor flavissimus* | EU637944 | *rhod* |
| Perciformes | Percoidei | Plesiopidae | *Calloplesiops altivelis* | FJ582909 | *CO1* |
| Perciformes | Percoidei | Plesiopidae | *Plesiops cephalotaenia* | EU167862 | *rag1* |
| Perciformes | Percoidei | Opistognathidae | *Opistognathus aurifrons* | FJ583762 | *CO1* |
| Perciformes | Percoidei | Opistognathidae | *Opistognathus aurifrons* | EU167852 | *rag1* |
| Perciformes | Percoidei | Opistognathidae | *Opistognathus punctatus* | FJ583764 | *CO1* |
| Perciformes | Percoidei | Opistognathidae | *Opistognathus rosenblatti* | FJ583767 | *CO1* |
| Perciformes | Percoidei | Banjosidae | *Banjos banjos* | EU167906 | *rag1* |
| Perciformes | Percoidei | Centrarchidae | *Acantharchus pomotis* | JN024711 | *CO1* |
| Perciformes | Percoidei | Centrarchidae | *Acantharchus pomotis* | AY517726 | *ND2* |
| Perciformes | Percoidei | Centrarchidae | *Acantharchus pomotis* | JF742884 | *rag1* |
| Perciformes | Percoidei | Centrarchidae | *Acantharchus pomotis* | AY742558 | *rhod* |
| Perciformes | Percoidei | Centrarchidae | *Ambloplites ariommus* | JN024726 | *CO1* |
| Perciformes | Percoidei | Centrarchidae | *Ambloplites ariommus* | EU501094 | *CYB* |
| Perciformes | Percoidei | Centrarchidae | *Ambloplites ariommus* | AY517727 | *ND2* |
| Perciformes | Percoidei | Centrarchidae | *Ambloplites ariommus* | JF742885 | *rag1* |
| Perciformes | Percoidei | Centrarchidae | *Ambloplites ariommus* | AY742559 | *rhod* |
| Perciformes | Percoidei | Centrarchidae | *Ambloplites cavifrons* | JN024730 | *CO1* |
| Perciformes | Percoidei | Centrarchidae | *Ambloplites cavifrons* | AY115979 | *CYB* |
| Perciformes | Percoidei | Centrarchidae | *Ambloplites cavifrons* | AY517728 | *ND2* |
| Perciformes | Percoidei | Centrarchidae | *Ambloplites cavifrons* | JF742886 | *rag1* |
| Perciformes | Percoidei | Centrarchidae | *Ambloplites cavifrons* | AY742560 | *rhod* |
| Perciformes | Percoidei | Centrarchidae | *Ambloplites constellatus* | JN024731 | *CO1* |
| Perciformes | Percoidei | Centrarchidae | *Ambloplites constellatus* | EU501085 | *CYB* |
| Perciformes | Percoidei | Centrarchidae | *Ambloplites constellatus* | AY517729 | *ND2* |
| Perciformes | Percoidei | Centrarchidae | *Ambloplites constellatus* | JF742887 | *rag1* |
| Perciformes | Percoidei | Centrarchidae | *Ambloplites constellatus* | AY742561 | *rhod* |
| Perciformes | Percoidei | Centrarchidae | *Ambloplites rupestris* | EU524409 | *CO1* |
| Perciformes | Percoidei | Centrarchidae | *Ambloplites rupestris* | EU501063 | *CYB* |
| Perciformes | Percoidei | Centrarchidae | *Ambloplites rupestris* | AY225723 | *ND2* |
| Perciformes | Percoidei | Centrarchidae | *Ambloplites rupestris* | JF742888 | *rag1* |
| Perciformes | Percoidei | Centrarchidae | *Ambloplites rupestris* | AY742562 | *rhod* |
| Perciformes | Percoidei | Centrarchidae | *Archoplites interruptus* | JN024808 | *CO1* |
| Perciformes | Percoidei | Centrarchidae | *Archoplites interruptus* | AY225665 | *CYB* |
| Perciformes | Percoidei | Centrarchidae | *Archoplites interruptus* | AY225725 | *ND2* |
| Perciformes | Percoidei | Centrarchidae | *Archoplites interruptus* | JF742889 | *rag1* |
| Perciformes | Percoidei | Centrarchidae | *Archoplites interruptus* | AY742563 | *rhod* |
| Perciformes | Percoidei | Centrarchidae | *Centrarchus macropterus* | JN024960 | *CO1* |
| Perciformes | Percoidei | Centrarchidae | *Centrarchus macropterus* | AY115982 | *CYB* |
| Perciformes | Percoidei | Centrarchidae | *Centrarchus macropterus* | AY225726 | *ND2* |
| Perciformes | Percoidei | Centrarchidae | *Centrarchus macropterus* | JF742890 | *rag1* |
| Perciformes | Percoidei | Centrarchidae | *Centrarchus macropterus* | AY742564 | *rhod* |
| Perciformes | Percoidei | Centrarchidae | *Chaenobryttus gulosus* | JN026994 | *CO1* |
| Perciformes | Percoidei | Centrarchidae | *Chaenobryttus gulosus* | AY115972 | *CYB* |
| Perciformes | Percoidei | Centrarchidae | *Chaenobryttus gulosus* | AY517737 | *ND2* |
| Perciformes | Percoidei | Centrarchidae | *Chaenobryttus gulosus* | JF742897 | *rag1* |
| Perciformes | Percoidei | Centrarchidae | *Chaenobryttus gulosus* | AY742572 | *rhod* |
| Perciformes | Percoidei | Centrarchidae | *Enneacanthus chaetodon* | JN025316 | *CO1* |
| Perciformes | Percoidei | Centrarchidae | *Enneacanthus chaetodon* | AY115983 | *CYB* |
| Perciformes | Percoidei | Centrarchidae | *Enneacanthus chaetodon* | AY517730 | *ND2* |
| Perciformes | Percoidei | Centrarchidae | *Enneacanthus chaetodon* | JF742891 | *rag1* |
| Perciformes | Percoidei | Centrarchidae | *Enneacanthus chaetodon* | AY742565 | *rhod* |
| Perciformes | Percoidei | Centrarchidae | *Enneacanthus gloriosus* | JN025320 | *CO1* |
| Perciformes | Percoidei | Centrarchidae | *Enneacanthus gloriosus* | AY115985 | *CYB* |
| Perciformes | Percoidei | Centrarchidae | *Enneacanthus gloriosus* | AY517731 | *ND2* |
| Perciformes | Percoidei | Centrarchidae | *Enneacanthus gloriosus* | JF742892 | *rag1* |
| Perciformes | Percoidei | Centrarchidae | *Enneacanthus gloriosus* | AY742566 | *rhod* |
| Perciformes | Percoidei | Centrarchidae | *Enneacanthus obesus* | JN025324 | *CO1* |
| Perciformes | Percoidei | Centrarchidae | *Enneacanthus obesus* | AY115987 | *CYB* |
| Perciformes | Percoidei | Centrarchidae | *Enneacanthus obesus* | AY225724 | *ND2* |
| Perciformes | Percoidei | Centrarchidae | *Enneacanthus obesus* | JF742893 | *rag1* |
| Perciformes | Percoidei | Centrarchidae | *Enneacanthus obesus* | AY742567 | *rhod* |
| Perciformes | Percoidei | Centrarchidae | *Lepomis auritus* | EU524704 | *CO1* |
| Perciformes | Percoidei | Centrarchidae | *Lepomis auritus* | AY115970 | *CYB* |
| Perciformes | Percoidei | Centrarchidae | *Lepomis auritus* | AY517732 | *ND2* |
| Perciformes | Percoidei | Centrarchidae | *Lepomis auritus* | JF742894 | *rag1* |
| Perciformes | Percoidei | Centrarchidae | *Lepomis auritus* | AY742568 | *rhod* |
| Perciformes | Percoidei | Centrarchidae | *Lepomis cyanellus* | EU524709 | *CO1* |
| Perciformes | Percoidei | Centrarchidae | *Lepomis cyanellus* | AY828958 | *CYB* |
| Perciformes | Percoidei | Centrarchidae | *Lepomis cyanellus* | AY517734 | *ND2* |
| Perciformes | Percoidei | Centrarchidae | *Lepomis cyanellus* | JF742895 | *rag1* |
| Perciformes | Percoidei | Centrarchidae | *Lepomis cyanellus* | AY742569 | *rhod* |
| Perciformes | Percoidei | Centrarchidae | *Lepomis gibbosus* | HQ600740 | *CO1* |
| Perciformes | Percoidei | Centrarchidae | *Lepomis gibbosus* | AY828961 | *CYB* |
| Perciformes | Percoidei | Centrarchidae | *Lepomis gibbosus* | AB271766 | *ND1* |
| Perciformes | Percoidei | Centrarchidae | *Lepomis gibbosus* | AY517735 | *ND2* |
| Perciformes | Percoidei | Centrarchidae | *Lepomis gibbosus* | JF742896 | *rag1* |
| Perciformes | Percoidei | Centrarchidae | *Lepomis gibbosus* | AY742571 | *rhod* |
| Perciformes | Percoidei | Centrarchidae | *Lepomis humilis* | EU524726 | *CO1* |
| Perciformes | Percoidei | Centrarchidae | *Lepomis humilis* | AY828964 | *CYB* |
| Perciformes | Percoidei | Centrarchidae | *Lepomis humilis* | AY517739 | *ND2* |
| Perciformes | Percoidei | Centrarchidae | *Lepomis humilis* | JF742899 | *rag1* |
| Perciformes | Percoidei | Centrarchidae | *Lepomis humilis* | AY742574 | *rhod* |
| Perciformes | Percoidei | Centrarchidae | *Lepomis macrochirus* | HQ536393 | *CO1* |
| Perciformes | Percoidei | Centrarchidae | *Lepomis macrochirus* | AY225667 | *CYB* |
| Perciformes | Percoidei | Centrarchidae | *Lepomis macrochirus* | AB167816 | *ND1* |
| Perciformes | Percoidei | Centrarchidae | *Lepomis macrochirus* | AY225727 | *ND2* |
| Perciformes | Percoidei | Centrarchidae | *Lepomis macrochirus* | AY430227 | *rag1* |
| Perciformes | Percoidei | Centrarchidae | *Lepomis macrochirus* | AY742576 | *rhod* |
| Perciformes | Percoidei | Centrarchidae | *Lepomis marginatus* | JN027021 | *CO1* |
| Perciformes | Percoidei | Centrarchidae | *Lepomis marginatus* | AY828971 | *CYB* |
| Perciformes | Percoidei | Centrarchidae | *Lepomis marginatus* | AY517741 | *ND2* |
| Perciformes | Percoidei | Centrarchidae | *Lepomis marginatus* | JF742900 | *rag1* |
| Perciformes | Percoidei | Centrarchidae | *Lepomis marginatus* | AY742578 | *rhod* |
| Perciformes | Percoidei | Centrarchidae | *Lepomis megalotis* | EU751847 | *CO1* |
| Perciformes | Percoidei | Centrarchidae | *Lepomis megalotis* | AY828975 | *CYB* |
| Perciformes | Percoidei | Centrarchidae | *Lepomis megalotis* | AB271767 | *ND1* |
| Perciformes | Percoidei | Centrarchidae | *Lepomis megalotis* | AY517743 | *ND2* |
| Perciformes | Percoidei | Centrarchidae | *Lepomis megalotis* | JF742901 | *rag1* |
| Perciformes | Percoidei | Centrarchidae | *Lepomis megalotis* | AY742580 | *rhod* |
| Perciformes | Percoidei | Centrarchidae | *Lepomis microlophus* | JN027046 | *CO1* |
| Perciformes | Percoidei | Centrarchidae | *Lepomis microlophus* | AY828980 | *CYB* |
| Perciformes | Percoidei | Centrarchidae | *Lepomis microlophus* | AB271768 | *ND1* |
| Perciformes | Percoidei | Centrarchidae | *Lepomis microlophus* | AY742652 | *ND2* |
| Perciformes | Percoidei | Centrarchidae | *Lepomis microlophus* | JF742902 | *rag1* |
| Perciformes | Percoidei | Centrarchidae | *Lepomis microlophus* | AY742581 | *rhod* |
| Perciformes | Percoidei | Centrarchidae | *Lepomis miniatus* | JN027053 | *CO1* |
| Perciformes | Percoidei | Centrarchidae | *Lepomis miniatus* | AY828986 | *CYB* |
| Perciformes | Percoidei | Centrarchidae | *Lepomis miniatus* | AY225728 | *ND2* |
| Perciformes | Percoidei | Centrarchidae | *Lepomis miniatus* | JF742903 | *rag1* |
| Perciformes | Percoidei | Centrarchidae | *Lepomis miniatus* | AY742584 | *rhod* |
| Perciformes | Percoidei | Centrarchidae | *Lepomis punctatus* | JN027054 | *CO1* |
| Perciformes | Percoidei | Centrarchidae | *Lepomis punctatus* | AY828991 | *CYB* |
| Perciformes | Percoidei | Centrarchidae | *Lepomis punctatus* | AY517746 | *ND2* |
| Perciformes | Percoidei | Centrarchidae | *Lepomis punctatus* | JF742904 | *rag1* |
| Perciformes | Percoidei | Centrarchidae | *Lepomis symmetricus* | JN027057 | *CO1* |
| Perciformes | Percoidei | Centrarchidae | *Lepomis symmetricus* | AY828996 | *CYB* |
| Perciformes | Percoidei | Centrarchidae | *Lepomis symmetricus* | AY517747 | *ND2* |
| Perciformes | Percoidei | Centrarchidae | *Lepomis symmetricus* | JF742905 | *rag1* |
| Perciformes | Percoidei | Centrarchidae | *Lepomis symmetricus* | AY742586 | *rhod* |
| Perciformes | Percoidei | Centrarchidae | *Micropterus cataractae* | JN027213 | *CO1* |
| Perciformes | Percoidei | Centrarchidae | *Micropterus cataractae* | AY225718 | *CYB* |
| Perciformes | Percoidei | Centrarchidae | *Micropterus cataractae* | AY225776 | *ND2* |
| Perciformes | Percoidei | Centrarchidae | *Micropterus cataractae* | JF742907 | *rag1* |
| Perciformes | Percoidei | Centrarchidae | *Micropterus cataractae* | AY742587 | *rhod* |
| Perciformes | Percoidei | Centrarchidae | *Micropterus coosae* | JN027215 | *CO1* |
| Perciformes | Percoidei | Centrarchidae | *Micropterus coosae* | AY225713 | *CYB* |
| Perciformes | Percoidei | Centrarchidae | *Micropterus coosae* | AY225773 | *ND2* |
| Perciformes | Percoidei | Centrarchidae | *Micropterus dolomieu* | EU524827 | *CO1* |
| Perciformes | Percoidei | Centrarchidae | *Micropterus dolomieu* | AY225685 | *CYB* |
| Perciformes | Percoidei | Centrarchidae | *Micropterus dolomieu* | AY225746 | *ND2* |
| Perciformes | Percoidei | Centrarchidae | *Micropterus dolomieu* | GU368818 | *rag1* |
| Perciformes | Percoidei | Centrarchidae | *Micropterus dolomieu* | AY742589 | *rhod* |
| Perciformes | Percoidei | Centrarchidae | *Micropterus floridanus* | HQ395644 | *CO1* |
| Perciformes | Percoidei | Centrarchidae | *Micropterus floridanus* | AY225670 | *CYB* |
| Perciformes | Percoidei | Centrarchidae | *Micropterus floridanus* | AY225734 | *ND2* |
| Perciformes | Percoidei | Centrarchidae | *Micropterus floridanus* | JF742913 | *rag1* |
| Perciformes | Percoidei | Centrarchidae | *Micropterus henshalli* | JF742909 | *rag1* |
| Perciformes | Percoidei | Centrarchidae | *Micropterus notius* | JN027230 | *CO1* |
| Perciformes | Percoidei | Centrarchidae | *Micropterus notius* | AY225706 | *CYB* |
| Perciformes | Percoidei | Centrarchidae | *Micropterus notius* | AY225767 | *ND2* |
| Perciformes | Percoidei | Centrarchidae | *Micropterus notius* | JF742910 | *rag1* |
| Perciformes | Percoidei | Centrarchidae | *Micropterus punctulatus* | JN027234 | *CO1* |
| Perciformes | Percoidei | Centrarchidae | *Micropterus punctulatus* | AY225698 | *CYB* |
| Perciformes | Percoidei | Centrarchidae | *Micropterus punctulatus* | AY225761 | *ND2* |
| Perciformes | Percoidei | Centrarchidae | *Micropterus punctulatus* | JF742911 | *rag1* |
| Perciformes | Percoidei | Centrarchidae | *Micropterus punctulatus punctulatus* | EU639405 | *CYB* |
| Perciformes | Percoidei | Centrarchidae | *Micropterus salmoides* | EU524829 | *CO1* |
| Perciformes | Percoidei | Centrarchidae | *Micropterus salmoides* | AF479273 | *CYB* |
| Perciformes | Percoidei | Centrarchidae | *Micropterus salmoides* | AY225740 | *ND2* |
| Perciformes | Percoidei | Centrarchidae | *Micropterus salmoides* | GU368833 | *rag1* |
| Perciformes | Percoidei | Centrarchidae | *Micropterus salmoides* | AY742597 | *rhod* |
| Perciformes | Percoidei | Centrarchidae | *Micropterus salmoides salmoides* | HQ395636 | *CO1* |
| Perciformes | Percoidei | Centrarchidae | *Micropterus salmoides salmoides* | EU639404 | *CYB* |
| Perciformes | Percoidei | Centrarchidae | *Micropterus treculii* | AY225703 | *CYB* |
| Perciformes | Percoidei | Centrarchidae | *Micropterus treculii* | AY225762 | *ND2* |
| Perciformes | Percoidei | Centrarchidae | *Micropterus treculii* | AY742599 | *rhod* |
| Perciformes | Percoidei | Centrarchidae | *Pomoxis annularis* | EU524283 | *CO1* |
| Perciformes | Percoidei | Centrarchidae | *Pomoxis annularis* | AY115990 | *CYB* |
| Perciformes | Percoidei | Centrarchidae | *Pomoxis annularis* | AY517748 | *ND2* |
| Perciformes | Percoidei | Centrarchidae | *Pomoxis annularis* | JF742915 | *rag1* |
| Perciformes | Percoidei | Centrarchidae | *Pomoxis annularis* | AY742600 | *rhod* |
| Perciformes | Percoidei | Centrarchidae | *Pomoxis nigromaculatus* | EU524285 | *CO1* |
| Perciformes | Percoidei | Centrarchidae | *Pomoxis nigromaculatus* | AY115991 | *CYB* |
| Perciformes | Percoidei | Centrarchidae | *Pomoxis nigromaculatus* | AY517750 | *ND2* |
| Perciformes | Percoidei | Centrarchidae | *Pomoxis nigromaculatus* | JF742916 | *rag1* |
| Perciformes | Percoidei | Centrarchidae | *Pomoxis nigromaculatus* | AY742603 | *rhod* |
| Perciformes | Percoidei | Percidae | *Ammocrypta beanii* | JN024764 | *CO1* |
| Perciformes | Percoidei | Percidae | *Ammocrypta beanii* | DQ512379 | *CYB* |
| Perciformes | Percoidei | Percidae | *Ammocrypta bifascia* | DQ323667 | *CO1* |
| Perciformes | Percoidei | Percidae | *Ammocrypta bifascia* | DQ512386 | *CYB* |
| Perciformes | Percoidei | Percidae | *Ammocrypta bifascia* | EF027171 | *ND2* |
| Perciformes | Percoidei | Percidae | *Ammocrypta clara* | JN024772 | *CO1* |
| Perciformes | Percoidei | Percidae | *Ammocrypta clara* | AF183941 | *CYB* |
| Perciformes | Percoidei | Percidae | *Ammocrypta clara* | EF027172 | *ND2* |
| Perciformes | Percoidei | Percidae | *Ammocrypta meridiana* | JN024783 | *CO1* |
| Perciformes | Percoidei | Percidae | *Ammocrypta meridiana* | AF183942 | *CYB* |
| Perciformes | Percoidei | Percidae | *Ammocrypta pellucida* | JN024787 | *CO1* |
| Perciformes | Percoidei | Percidae | *Ammocrypta pellucida* | FJ381008 | *CYB* |
| Perciformes | Percoidei | Percidae | *Ammocrypta pellucida* | FJ381259 | *ND2* |
| Perciformes | Percoidei | Percidae | *Ammocrypta pellucida* | FJ381304 | *rag1* |
| Perciformes | Percoidei | Percidae | *Ammocrypta vivax* | JN024792 | *CO1* |
| Perciformes | Percoidei | Percidae | *Ammocrypta vivax* | AF183944 | *CYB* |
| Perciformes | Percoidei | Percidae | *Bellator xenisma* | HQ010076 | *CO1* |
| Perciformes | Percoidei | Percidae | *Crystallaria asprella* | JN025156 | *CO1* |
| Perciformes | Percoidei | Percidae | *Crystallaria asprella* | AF099898 | *CYB* |
| Perciformes | Percoidei | Percidae | *Crystallaria asprella* | FJ381260 | *ND2* |
| Perciformes | Percoidei | Percidae | *Crystallaria asprella* | FJ381305 | *rag1* |
| Perciformes | Percoidei | Percidae | *Etheostoma acuticeps* | JN025474 | *CO1* |
| Perciformes | Percoidei | Percidae | *Etheostoma acuticeps* | AY742655 | *CYB* |
| Perciformes | Percoidei | Percidae | *Etheostoma acuticeps* | GU015636 | *rag1* |
| Perciformes | Percoidei | Percidae | *Etheostoma akatulo* | JN025475 | *CO1* |
| Perciformes | Percoidei | Percidae | *Etheostoma aquali* | JN025485 | *CO1* |
| Perciformes | Percoidei | Percidae | *Etheostoma aquali* | DQ025497 | *CYB* |
| Perciformes | Percoidei | Percidae | *Etheostoma aquali* | GU015639 | *rag1* |
| Perciformes | Percoidei | Percidae | *Etheostoma artesiae* | JN025497 | *CO1* |
| Perciformes | Percoidei | Percidae | *Etheostoma artesiae* | EF027179 | *ND2* |
| Perciformes | Percoidei | Percidae | *Etheostoma asprigene* | JN025585 | *CO1* |
| Perciformes | Percoidei | Percidae | *Etheostoma asprigene* | DQ465071 | *CYB* |
| Perciformes | Percoidei | Percidae | *Etheostoma asprigene* | EF027180 | *ND2* |
| Perciformes | Percoidei | Percidae | *Etheostoma asprigene* | JF742883 | *rag1* |
| Perciformes | Percoidei | Percidae | *Etheostoma australe* | JN025593 | *CO1* |
| Perciformes | Percoidei | Percidae | *Etheostoma australe* | EF027181 | *ND2* |
| Perciformes | Percoidei | Percidae | *Etheostoma baileyi* | AY964713 | *CYB* |
| Perciformes | Percoidei | Percidae | *Etheostoma barbouri* | JN025594 | *CO1* |
| Perciformes | Percoidei | Percidae | *Etheostoma barbouri* | AF412528 | *CYB* |
| Perciformes | Percoidei | Percidae | *Etheostoma barbouri* | FJ012508 | *ND2* |
| Perciformes | Percoidei | Percidae | *Etheostoma barbouri* | AY251541 | *ND4* |
| Perciformes | Percoidei | Percidae | *Etheostoma barrenense* | JN025596 | *CO1* |
| Perciformes | Percoidei | Percidae | *Etheostoma barrenense* | AF288424 | *CYB* |
| Perciformes | Percoidei | Percidae | *Etheostoma basilare* | JN025600 | *CO1* |
| Perciformes | Percoidei | Percidae | *Etheostoma basilare* | FJ012420 | *CYB* |
| Perciformes | Percoidei | Percidae | *Etheostoma basilare* | FJ012540 | *ND2* |
| Perciformes | Percoidei | Percidae | *Etheostoma basilare* | AY251548 | *ND4* |
| Perciformes | Percoidei | Percidae | *Etheostoma basilare* | JF742866 | *rag1* |
| Perciformes | Percoidei | Percidae | *Etheostoma bellator* | JN025605 | *CO1* |
| Perciformes | Percoidei | Percidae | *Etheostoma bellator* | AF288425 | *CYB* |
| Perciformes | Percoidei | Percidae | *Etheostoma bellator* | JF742870 | *rag1* |
| Perciformes | Percoidei | Percidae | *Etheostoma bellum* | JN025609 | *CO1* |
| Perciformes | Percoidei | Percidae | *Etheostoma bellum* | AY742656 | *CYB* |
| Perciformes | Percoidei | Percidae | *Etheostoma bellum* | GU015641 | *rag1* |
| Perciformes | Percoidei | Percidae | *Etheostoma bison* | JN025612 | *CO1* |
| Perciformes | Percoidei | Percidae | *Etheostoma bison* | FJ381084 | *CYB* |
| Perciformes | Percoidei | Percidae | *Etheostoma bison* | EF027184 | *ND2* |
| Perciformes | Percoidei | Percidae | *Etheostoma blennioides* | JN025617 | *CO1* |
| Perciformes | Percoidei | Percidae | *Etheostoma blennioides* | EU296665 | *CYB* |
| Perciformes | Percoidei | Percidae | *Etheostoma blennioides* | FJ381263 | *ND2* |
| Perciformes | Percoidei | Percidae | *Etheostoma blennioides* | FJ381309 | *rag1* |
| Perciformes | Percoidei | Percidae | *Etheostoma blennioides blennioides* | EU296659 | *CYB* |
| Perciformes | Percoidei | Percidae | *Etheostoma blennioides gutselli* | JN025897 | *CO1* |
| Perciformes | Percoidei | Percidae | *Etheostoma blennioides gutselli* | EU296650 | *CYB* |
| Perciformes | Percoidei | Percidae | *Etheostoma blennius* | JN025641 | *CO1* |
| Perciformes | Percoidei | Percidae | *Etheostoma blennius* | EU296682 | *CYB* |
| Perciformes | Percoidei | Percidae | *Etheostoma blennius blennius* | AY964697 | *CYB* |
| Perciformes | Percoidei | Percidae | *Etheostoma boschungi* | JN025645 | *CO1* |
| Perciformes | Percoidei | Percidae | *Etheostoma boschungi* | EF027185 | *ND2* |
| Perciformes | Percoidei | Percidae | *Etheostoma brevirostrum* | JN025648 | *CO1* |
| Perciformes | Percoidei | Percidae | *Etheostoma brevirostrum* | AF288428 | *CYB* |
| Perciformes | Percoidei | Percidae | *Etheostoma burri* | JN025653 | *CO1* |
| Perciformes | Percoidei | Percidae | *Etheostoma burri* | FJ381080 | *CYB* |
| Perciformes | Percoidei | Percidae | *Etheostoma burri* | FJ381276 | *ND2* |
| Perciformes | Percoidei | Percidae | *Etheostoma burri* | FJ381322 | *rag1* |
| Perciformes | Percoidei | Percidae | *Etheostoma caeruleum* | JN025668 | *CO1* |
| Perciformes | Percoidei | Percidae | *Etheostoma caeruleum* | DQ465091 | *CYB* |
| Perciformes | Percoidei | Percidae | *Etheostoma caeruleum* | FJ381268 | *ND2* |
| Perciformes | Percoidei | Percidae | *Etheostoma caeruleum* | HM050105 | *ND4* |
| Perciformes | Percoidei | Percidae | *Etheostoma caeruleum* | AY430226 | *rag1* |
| Perciformes | Percoidei | Percidae | *Etheostoma camurum* | JN025724 | *CO1* |
| Perciformes | Percoidei | Percidae | *Etheostoma camurum* | GU015047 | *CYB* |
| Perciformes | Percoidei | Percidae | *Etheostoma camurum* | EU814330 | *ND2* |
| Perciformes | Percoidei | Percidae | *Etheostoma camurum* | FJ381307 | *rag1* |
| Perciformes | Percoidei | Percidae | *Etheostoma cervus* | FJ423444 | *CYB* |
| Perciformes | Percoidei | Percidae | *Etheostoma chermocki* | JN025730 | *CO1* |
| Perciformes | Percoidei | Percidae | *Etheostoma chermocki* | AF288429 | *CYB* |
| Perciformes | Percoidei | Percidae | *Etheostoma chienense* | JN025735 | *CO1* |
| Perciformes | Percoidei | Percidae | *Etheostoma chienense* | AF123029 | *CYB* |
| Perciformes | Percoidei | Percidae | *Etheostoma chlorobranchium* | JN025739 | *CO1* |
| Perciformes | Percoidei | Percidae | *Etheostoma chlorobranchium* | GU015089 | *CYB* |
| Perciformes | Percoidei | Percidae | *Etheostoma chlorobranchium* | EU810800 | *ND2* |
| Perciformes | Percoidei | Percidae | *Etheostoma chlorobranchium* | GU015696 | *rag1* |
| Perciformes | Percoidei | Percidae | *Etheostoma chlorosomum* | JN025750 | *CO1* |
| Perciformes | Percoidei | Percidae | *Etheostoma chlorosomum* | AY374264 | *CYB* |
| Perciformes | Percoidei | Percidae | *Etheostoma chlorosomum* | EF027188 | *ND2* |
| Perciformes | Percoidei | Percidae | *Etheostoma chuckwachatte* | JN025752 | *CO1* |
| Perciformes | Percoidei | Percidae | *Etheostoma chuckwachatte* | EU094678 | *CYB* |
| Perciformes | Percoidei | Percidae | *Etheostoma chuckwachatte* | GU015706 | *rag1* |
| Perciformes | Percoidei | Percidae | *Etheostoma cinereum* | JN025755 | *CO1* |
| Perciformes | Percoidei | Percidae | *Etheostoma cinereum* | AY964717 | *CYB* |
| Perciformes | Percoidei | Percidae | *Etheostoma cinereum* | FJ381261 | *ND2* |
| Perciformes | Percoidei | Percidae | *Etheostoma cinereum* | FJ381306 | *rag1* |
| Perciformes | Percoidei | Percidae | *Etheostoma collettei* | JN025759 | *CO1* |
| Perciformes | Percoidei | Percidae | *Etheostoma collettei* | EU046677 | *CYB* |
| Perciformes | Percoidei | Percidae | *Etheostoma collettei* | EF027189 | *ND2* |
| Perciformes | Percoidei | Percidae | *Etheostoma collis* | EF027190 | *ND2* |
| Perciformes | Percoidei | Percidae | *Etheostoma colorosum* | AF288430 | *CYB* |
| Perciformes | Percoidei | Percidae | *Etheostoma coosae* | JN025769 | *CO1* |
| Perciformes | Percoidei | Percidae | *Etheostoma coosae* | AF288431 | *CYB* |
| Perciformes | Percoidei | Percidae | *Etheostoma corona* | JN025772 | *CO1* |
| Perciformes | Percoidei | Percidae | *Etheostoma corona* | AF123030 | *CYB* |
| Perciformes | Percoidei | Percidae | *Etheostoma cragini* | JN025776 | *CO1* |
| Perciformes | Percoidei | Percidae | *Etheostoma cragini* | EF027191 | *ND2* |
| Perciformes | Percoidei | Percidae | *Etheostoma crossopterum* | JN025782 | *CO1* |
| Perciformes | Percoidei | Percidae | *Etheostoma crossopterum* | AF123031 | *CYB* |
| Perciformes | Percoidei | Percidae | *Etheostoma davisoni* | JN025784 | *CO1* |
| Perciformes | Percoidei | Percidae | *Etheostoma denoncourti* | JN025788 | *CO1* |
| Perciformes | Percoidei | Percidae | *Etheostoma denoncourti* | EU094680 | *CYB* |
| Perciformes | Percoidei | Percidae | *Etheostoma denoncourti* | EU810802 | *ND2* |
| Perciformes | Percoidei | Percidae | *Etheostoma denoncourti* | GU015707 | *rag1* |
| Perciformes | Percoidei | Percidae | *Etheostoma derivativum* | JN025790 | *CO1* |
| Perciformes | Percoidei | Percidae | *Etheostoma derivativum* | FJ012468 | *CYB* |
| Perciformes | Percoidei | Percidae | *Etheostoma derivativum* | FJ012614 | *ND2* |
| Perciformes | Percoidei | Percidae | *Etheostoma derivativum* | AY251549 | *ND4* |
| Perciformes | Percoidei | Percidae | *Etheostoma ditrema* | JN025799 | *CO1* |
| Perciformes | Percoidei | Percidae | *Etheostoma ditrema* | FJ012507 | *CYB* |
| Perciformes | Percoidei | Percidae | *Etheostoma ditrema* | EF027192 | *ND2* |
| Perciformes | Percoidei | Percidae | *Etheostoma douglasi* | JN025805 | *CO1* |
| Perciformes | Percoidei | Percidae | *Etheostoma douglasi* | EU094684 | *CYB* |
| Perciformes | Percoidei | Percidae | *Etheostoma douglasi* | GU015709 | *rag1* |
| Perciformes | Percoidei | Percidae | *Etheostoma duryi* | JN025806 | *CO1* |
| Perciformes | Percoidei | Percidae | *Etheostoma duryi* | AF288432 | *CYB* |
| Perciformes | Percoidei | Percidae | *Etheostoma edwini* | JN025808 | *CO1* |
| Perciformes | Percoidei | Percidae | *Etheostoma edwini* | AY374267 | *CYB* |
| Perciformes | Percoidei | Percidae | *Etheostoma edwini* | EF027193 | *ND2* |
| Perciformes | Percoidei | Percidae | *Etheostoma erizonum* | AY964685 | *CYB* |
| Perciformes | Percoidei | Percidae | *Etheostoma etnieri* | JN025818 | *CO1* |
| Perciformes | Percoidei | Percidae | *Etheostoma etnieri* | AF288433 | *CYB* |
| Perciformes | Percoidei | Percidae | *Etheostoma etowahae* | JN025822 | *CO1* |
| Perciformes | Percoidei | Percidae | *Etheostoma etowahae* | DQ025502 | *CYB* |
| Perciformes | Percoidei | Percidae | *Etheostoma etowahae* | GU015711 | *rag1* |
| Perciformes | Percoidei | Percidae | *Etheostoma euzonum* | JN025824 | *CO1* |
| Perciformes | Percoidei | Percidae | *Etheostoma euzonum* | AF289263 | *CYB* |
| Perciformes | Percoidei | Percidae | *Etheostoma euzonum euzonum* | AY964686 | *CYB* |
| Perciformes | Percoidei | Percidae | *Etheostoma exile* | EU524025 | *CO1* |
| Perciformes | Percoidei | Percidae | *Etheostoma exile* | AF386541 | *CYB* |
| Perciformes | Percoidei | Percidae | *Etheostoma exile* | EF027194 | *ND2* |
| Perciformes | Percoidei | Percidae | *Etheostoma flabellare* | EU524036 | *CO1* |
| Perciformes | Percoidei | Percidae | *Etheostoma flabellare* | AF412526 | *CYB* |
| Perciformes | Percoidei | Percidae | *Etheostoma flabellare* | AF412540 | *ND2* |
| Perciformes | Percoidei | Percidae | *Etheostoma flabellare* | FJ381308 | *rag1* |
| Perciformes | Percoidei | Percidae | *Etheostoma flavum* | JN025856 | *CO1* |
| Perciformes | Percoidei | Percidae | *Etheostoma flavum* | AY964714 | *CYB* |
| Perciformes | Percoidei | Percidae | *Etheostoma fonticola* | JN025859 | *CO1* |
| Perciformes | Percoidei | Percidae | *Etheostoma fonticola* | FJ381005 | *CYB* |
| Perciformes | Percoidei | Percidae | *Etheostoma fonticola* | FJ381273 | *ND2* |
| Perciformes | Percoidei | Percidae | *Etheostoma fonticola* | FJ381318 | *rag1* |
| Perciformes | Percoidei | Percidae | *Etheostoma forbesi* | JN025861 | *CO1* |
| Perciformes | Percoidei | Percidae | *Etheostoma forbesi* | AF123032 | *CYB* |
| Perciformes | Percoidei | Percidae | *Etheostoma fragi* | JN025863 | *CO1* |
| Perciformes | Percoidei | Percidae | *Etheostoma fragi* | FJ381035 | *CYB* |
| Perciformes | Percoidei | Percidae | *Etheostoma fragi* | FJ381281 | *ND2* |
| Perciformes | Percoidei | Percidae | *Etheostoma fragi* | FJ381328 | *rag1* |
| Perciformes | Percoidei | Percidae | *Etheostoma fricksium* | JN025865 | *CO1* |
| Perciformes | Percoidei | Percidae | *Etheostoma fricksium* | EF027197 | *ND2* |
| Perciformes | Percoidei | Percidae | *Etheostoma fusiforme* | JN025868 | *CO1* |
| Perciformes | Percoidei | Percidae | *Etheostoma gracile* | JN025883 | *CO1* |
| Perciformes | Percoidei | Percidae | *Etheostoma gracile* | AF386538 | *CYB* |
| Perciformes | Percoidei | Percidae | *Etheostoma grahami* | JN025885 | *CO1* |
| Perciformes | Percoidei | Percidae | *Etheostoma grahami* | EF027198 | *ND2* |
| Perciformes | Percoidei | Percidae | *Etheostoma histrio* | JN025905 | *CO1* |
| Perciformes | Percoidei | Percidae | *Etheostoma histrio* | AY964708 | *CYB* |
| Perciformes | Percoidei | Percidae | *Etheostoma histrio* | EF027199 | *ND2* |
| Perciformes | Percoidei | Percidae | *Etheostoma hopkinsi binotatum* | EF027183 | *ND2* |
| Perciformes | Percoidei | Percidae | *Etheostoma hopkinsi hopkinsi* | EF027200 | *ND2* |
| Perciformes | Percoidei | Percidae | *Etheostoma inscriptum* | JN025906 | *CO1* |
| Perciformes | Percoidei | Percidae | *Etheostoma inscriptum* | AF288435 | *CYB* |
| Perciformes | Percoidei | Percidae | *Etheostoma jessiae* | JN025911 | *CO1* |
| Perciformes | Percoidei | Percidae | *Etheostoma jessiae* | EF027201 | *ND2* |
| Perciformes | Percoidei | Percidae | *Etheostoma jordani* | JN025913 | *CO1* |
| Perciformes | Percoidei | Percidae | *Etheostoma jordani* | EU094685 | *CYB* |
| Perciformes | Percoidei | Percidae | *Etheostoma jordani* | EF027175 | *ND2* |
| Perciformes | Percoidei | Percidae | *Etheostoma jordani* | GU015713 | *rag1* |
| Perciformes | Percoidei | Percidae | *Etheostoma juliae* | JN025916 | *CO1* |
| Perciformes | Percoidei | Percidae | *Etheostoma juliae* | EU094689 | *CYB* |
| Perciformes | Percoidei | Percidae | *Etheostoma juliae* | EF027176 | *ND2* |
| Perciformes | Percoidei | Percidae | *Etheostoma juliae* | GU015716 | *rag1* |
| Perciformes | Percoidei | Percidae | *Etheostoma kanawhae* | JN025926 | *CO1* |
| Perciformes | Percoidei | Percidae | *Etheostoma kanawhae* | AY964689 | *CYB* |
| Perciformes | Percoidei | Percidae | *Etheostoma kantuckeense* | JN025932 | *CO1* |
| Perciformes | Percoidei | Percidae | *Etheostoma kantuckeense* | FJ381098 | *CYB* |
| Perciformes | Percoidei | Percidae | *Etheostoma kantuckeense* | EF027202 | *ND2* |
| Perciformes | Percoidei | Percidae | *Etheostoma kennicotti* | JN025935 | *CO1* |
| Perciformes | Percoidei | Percidae | *Etheostoma kennicotti* | AF412527 | *CYB* |
| Perciformes | Percoidei | Percidae | *Etheostoma kennicotti* | AF412541 | *ND2* |
| Perciformes | Percoidei | Percidae | *Etheostoma kennicotti* | AY251542 | *ND4* |
| Perciformes | Percoidei | Percidae | *Etheostoma lachneri* | JN025949 | *CO1* |
| Perciformes | Percoidei | Percidae | *Etheostoma lachneri* | AF288436 | *CYB* |
| Perciformes | Percoidei | Percidae | *Etheostoma lawrencei* | JN025953 | *CO1* |
| Perciformes | Percoidei | Percidae | *Etheostoma lawrencei* | FJ381089 | *CYB* |
| Perciformes | Percoidei | Percidae | *Etheostoma lawrencei* | FJ381284 | *ND2* |
| Perciformes | Percoidei | Percidae | *Etheostoma lawrencei* | FJ381330 | *rag1* |
| Perciformes | Percoidei | Percidae | *Etheostoma lepidum* | JN025965 | *CO1* |
| Perciformes | Percoidei | Percidae | *Etheostoma lepidum* | EF027204 | *ND2* |
| Perciformes | Percoidei | Percidae | *Etheostoma longimanum* | JN025972 | *CO1* |
| Perciformes | Percoidei | Percidae | *Etheostoma longimanum* | GQ183640 | *CYB* |
| Perciformes | Percoidei | Percidae | *Etheostoma lugoi* | EF027205 | *ND2* |
| Perciformes | Percoidei | Percidae | *Etheostoma luteovinctum* | JN025977 | *CO1* |
| Perciformes | Percoidei | Percidae | *Etheostoma luteovinctum* | FJ381010 | *CYB* |
| Perciformes | Percoidei | Percidae | *Etheostoma luteovinctum* | FJ381269 | *ND2* |
| Perciformes | Percoidei | Percidae | *Etheostoma luteovinctum* | FJ381313 | *rag1* |
| Perciformes | Percoidei | Percidae | *Etheostoma lynceum* | JN025997 | *CO1* |
| Perciformes | Percoidei | Percidae | *Etheostoma lynceum* | EU296688 | *CYB* |
| Perciformes | Percoidei | Percidae | *Etheostoma maculatum* | JN025999 | *CO1* |
| Perciformes | Percoidei | Percidae | *Etheostoma maculatum* | HQ005520 | *CYB* |
| Perciformes | Percoidei | Percidae | *Etheostoma maculatum* | EU810778 | *ND2* |
| Perciformes | Percoidei | Percidae | *Etheostoma maculatum* | HQ005682 | *rag1* |
| Perciformes | Percoidei | Percidae | *Etheostoma meadiae* | JN026005 | *CO1* |
| Perciformes | Percoidei | Percidae | *Etheostoma microlepidum* | JN026010 | *CO1* |
| Perciformes | Percoidei | Percidae | *Etheostoma microlepidum* | AY742664 | *CYB* |
| Perciformes | Percoidei | Percidae | *Etheostoma microlepidum* | HQ005690 | *rag1* |
| Perciformes | Percoidei | Percidae | *Etheostoma microperca* | JN026028 | *CO1* |
| Perciformes | Percoidei | Percidae | *Etheostoma microperca* | FJ381003 | *CYB* |
| Perciformes | Percoidei | Percidae | *Etheostoma microperca* | FJ381275 | *ND2* |
| Perciformes | Percoidei | Percidae | *Etheostoma microperca* | FJ381321 | *rag1* |
| Perciformes | Percoidei | Percidae | *Etheostoma moorei* | JN026030 | *CO1* |
| Perciformes | Percoidei | Percidae | *Etheostoma moorei* | HQ005539 | *CYB* |
| Perciformes | Percoidei | Percidae | *Etheostoma moorei* | GU015725 | *rag1* |
| Perciformes | Percoidei | Percidae | *Etheostoma neopterum* | AF123033 | *CYB* |
| Perciformes | Percoidei | Percidae | *Etheostoma newmanii* | AY964701 | *CYB* |
| Perciformes | Percoidei | Percidae | *Etheostoma nianguae* | AY964692 | *CYB* |
| Perciformes | Percoidei | Percidae | *Etheostoma nigripinne* | JN026036 | *CO1* |
| Perciformes | Percoidei | Percidae | *Etheostoma nigripinne* | AF123034 | *CYB* |
| Perciformes | Percoidei | Percidae | *Etheostoma nigrum* | JN026045 | *CO1* |
| Perciformes | Percoidei | Percidae | *Etheostoma nigrum* | AF183945 | *CYB* |
| Perciformes | Percoidei | Percidae | *Etheostoma nuchale* | JN026068 | *CO1* |
| Perciformes | Percoidei | Percidae | *Etheostoma nuchale* | HM856127 | *ND2* |
| Perciformes | Percoidei | Percidae | *Etheostoma obeyense* | JN026070 | *CO1* |
| Perciformes | Percoidei | Percidae | *Etheostoma obeyense* | FJ012485 | *CYB* |
| Perciformes | Percoidei | Percidae | *Etheostoma obeyense* | FJ012625 | *ND2* |
| Perciformes | Percoidei | Percidae | *Etheostoma obeyense* | AY251543 | *ND4* |
| Perciformes | Percoidei | Percidae | *Etheostoma occidentale* | DQ089063 | *CYB* |
| Perciformes | Percoidei | Percidae | *Etheostoma okaloosae* | JN026071 | *CO1* |
| Perciformes | Percoidei | Percidae | *Etheostoma okaloosae* | JF314730 | *CYB* |
| Perciformes | Percoidei | Percidae | *Etheostoma okaloosae* | EF027209 | *ND2* |
| Perciformes | Percoidei | Percidae | *Etheostoma olivaceum* | JN026072 | *CO1* |
| Perciformes | Percoidei | Percidae | *Etheostoma olivaceum* | AF123036 | *CYB* |
| Perciformes | Percoidei | Percidae | *Etheostoma olmstedi* | JN026081 | *CO1* |
| Perciformes | Percoidei | Percidae | *Etheostoma olmstedi* | GQ183689 | *CYB* |
| Perciformes | Percoidei | Percidae | *Etheostoma olmstedi* | EF027210 | *ND2* |
| Perciformes | Percoidei | Percidae | *Etheostoma oophylax* | JN026091 | *CO1* |
| Perciformes | Percoidei | Percidae | *Etheostoma oophylax* | AF123037 | *CYB* |
| Perciformes | Percoidei | Percidae | *Etheostoma oophylax* | AF412538 | *ND2* |
| Perciformes | Percoidei | Percidae | *Etheostoma oophylax* | JF742873 | *rag1* |
| Perciformes | Percoidei | Percidae | *Etheostoma orientale* | DQ089074 | *CYB* |
| Perciformes | Percoidei | Percidae | *Etheostoma osburni* | JN026092 | *CO1* |
| Perciformes | Percoidei | Percidae | *Etheostoma osburni* | AY964691 | *CYB* |
| Perciformes | Percoidei | Percidae | *Etheostoma pallididorsum* | EF027211 | *ND2* |
| Perciformes | Percoidei | Percidae | *Etheostoma parvipinne* | JN026098 | *CO1* |
| Perciformes | Percoidei | Percidae | *Etheostoma parvipinne* | AF123044 | *CYB* |
| Perciformes | Percoidei | Percidae | *Etheostoma parvipinne* | EF027212 | *ND2* |
| Perciformes | Percoidei | Percidae | *Etheostoma percnurum* | JN026108 | *CO1* |
| Perciformes | Percoidei | Percidae | *Etheostoma percnurum* | AF123038 | *CYB* |
| Perciformes | Percoidei | Percidae | *Etheostoma percnurum* | AF412539 | *ND2* |
| Perciformes | Percoidei | Percidae | *Etheostoma percnurum* | AY251544 | *ND4* |
| Perciformes | Percoidei | Percidae | *Etheostoma perlongum* | JN026110 | *CO1* |
| Perciformes | Percoidei | Percidae | *Etheostoma perlongum* | GQ183705 | *CYB* |
| Perciformes | Percoidei | Percidae | *Etheostoma pholidotum* | EU296648 | *CYB* |
| Perciformes | Percoidei | Percidae | *Etheostoma phytophilum* | JN026115 | *CO1* |
| Perciformes | Percoidei | Percidae | *Etheostoma planasaxatile* | JF497263 | *CYB* |
| Perciformes | Percoidei | Percidae | *Etheostoma planasaxatile* | JF497461 | *rag1* |
| Perciformes | Percoidei | Percidae | *Etheostoma podostemone* | JN026116 | *CO1* |
| Perciformes | Percoidei | Percidae | *Etheostoma podostemone* | AF045346 | *CYB* |
| Perciformes | Percoidei | Percidae | *Etheostoma pottsii* | JN026118 | *CO1* |
| Perciformes | Percoidei | Percidae | *Etheostoma pottsii* | EF027213 | *ND2* |
| Perciformes | Percoidei | Percidae | *Etheostoma proeliare* | JN026163 | *CO1* |
| Perciformes | Percoidei | Percidae | *Etheostoma proeliare* | FJ381006 | *CYB* |
| Perciformes | Percoidei | Percidae | *Etheostoma proeliare* | HM593878 | *ND2* |
| Perciformes | Percoidei | Percidae | *Etheostoma proeliare* | FJ381320 | *rag1* |
| Perciformes | Percoidei | Percidae | *Etheostoma pseudovulatum* | AF123039 | *CYB* |
| Perciformes | Percoidei | Percidae | *Etheostoma punctulatum* | JN026167 | *CO1* |
| Perciformes | Percoidei | Percidae | *Etheostoma punctulatum* | FJ381007 | *CYB* |
| Perciformes | Percoidei | Percidae | *Etheostoma punctulatum* | EF027218 | *ND2* |
| Perciformes | Percoidei | Percidae | *Etheostoma punctulatum* | FJ381319 | *rag1* |
| Perciformes | Percoidei | Percidae | *Etheostoma pyrrhogaster* | FJ423437 | *CYB* |
| Perciformes | Percoidei | Percidae | *Etheostoma radiosum* | JN026177 | *CO1* |
| Perciformes | Percoidei | Percidae | *Etheostoma radiosum* | DQ465070 | *CYB* |
| Perciformes | Percoidei | Percidae | *Etheostoma radiosum* | FJ381271 | *ND2* |
| Perciformes | Percoidei | Percidae | *Etheostoma radiosum* | FJ381311 | *rag1* |
| Perciformes | Percoidei | Percidae | *Etheostoma rafinesquei* | JN026283 | *CO1* |
| Perciformes | Percoidei | Percidae | *Etheostoma rafinesquei* | EU296689 | *CYB* |
| Perciformes | Percoidei | Percidae | *Etheostoma ramseyi* | JN026287 | *CO1* |
| Perciformes | Percoidei | Percidae | *Etheostoma ramseyi* | AF288440 | *CYB* |
| Perciformes | Percoidei | Percidae | *Etheostoma raneyi* | FJ423425 | *CYB* |
| Perciformes | Percoidei | Percidae | *Etheostoma rubrum* | JN026290 | *CO1* |
| Perciformes | Percoidei | Percidae | *Etheostoma rubrum* | AF274446 | *CYB* |
| Perciformes | Percoidei | Percidae | *Etheostoma rubrum* | GU015727 | *rag1* |
| Perciformes | Percoidei | Percidae | *Etheostoma rufilineatum* | JN026297 | *CO1* |
| Perciformes | Percoidei | Percidae | *Etheostoma rufilineatum* | GU015274 | *CYB* |
| Perciformes | Percoidei | Percidae | *Etheostoma rufilineatum* | GU015768 | *rag1* |
| Perciformes | Percoidei | Percidae | *Etheostoma rupestre* | JN026303 | *CO1* |
| Perciformes | Percoidei | Percidae | *Etheostoma rupestre* | AY964704 | *CYB* |
| Perciformes | Percoidei | Percidae | *Etheostoma rupestre* | JF742875 | *rag1* |
| Perciformes | Percoidei | Percidae | *Etheostoma sagitta* | JN026307 | *CO1* |
| Perciformes | Percoidei | Percidae | *Etheostoma sagitta* | AF386542 | *CYB* |
| Perciformes | Percoidei | Percidae | *Etheostoma sagitta sagitta* | AY964695 | *CYB* |
| Perciformes | Percoidei | Percidae | *Etheostoma sagitta spilotum* | EF027220 | *ND2* |
| Perciformes | Percoidei | Percidae | *Etheostoma saludae* | JN026312 | *CO1* |
| Perciformes | Percoidei | Percidae | *Etheostoma sanguifluum* | JN026315 | *CO1* |
| Perciformes | Percoidei | Percidae | *Etheostoma sanguifluum* | HQ005557 | *CYB* |
| Perciformes | Percoidei | Percidae | *Etheostoma sanguifluum* | HQ005706 | *rag1* |
| Perciformes | Percoidei | Percidae | *Etheostoma scotti* | AF288443 | *CYB* |
| Perciformes | Percoidei | Percidae | *Etheostoma segrex* | EF027221 | *ND2* |
| Perciformes | Percoidei | Percidae | *Etheostoma segrex* | JF742880 | *rag1* |
| Perciformes | Percoidei | Percidae | *Etheostoma sequatchiense* | AY964699 | *CYB* |
| Perciformes | Percoidei | Percidae | *Etheostoma serrifer* | JN026318 | *CO1* |
| Perciformes | Percoidei | Percidae | *Etheostoma simoterum* | JN026326 | *CO1* |
| Perciformes | Percoidei | Percidae | *Etheostoma simoterum* | JF497337 | *CYB* |
| Perciformes | Percoidei | Percidae | *Etheostoma simoterum* | EF027222 | *ND2* |
| Perciformes | Percoidei | Percidae | *Etheostoma simoterum* | JF497472 | *rag1* |
| Perciformes | Percoidei | Percidae | *Etheostoma simoterum atripinne* | JF497202 | *CYB* |
| Perciformes | Percoidei | Percidae | *Etheostoma simoterum atripinne* | JF497458 | *rag1* |
| Perciformes | Percoidei | Percidae | *Etheostoma smithi* | JN026327 | *CO1* |
| Perciformes | Percoidei | Percidae | *Etheostoma smithi* | AY374275 | *CYB* |
| Perciformes | Percoidei | Percidae | *Etheostoma smithi* | FJ012647 | *ND2* |
| Perciformes | Percoidei | Percidae | *Etheostoma smithi* | AY251545 | *ND4* |
| Perciformes | Percoidei | Percidae | *Etheostoma spectabile* | JN026386 | *CO1* |
| Perciformes | Percoidei | Percidae | *Etheostoma spectabile* | AF045344 | *CYB* |
| Perciformes | Percoidei | Percidae | *Etheostoma spectabile* | FJ381296 | *ND2* |
| Perciformes | Percoidei | Percidae | *Etheostoma spectabile* | FJ381342 | *rag1* |
| Perciformes | Percoidei | Percidae | *Etheostoma spectabile pulchellum* | FJ381045 | *CYB* |
| Perciformes | Percoidei | Percidae | *Etheostoma spectabile pulchellum* | FJ381297 | *ND2* |
| Perciformes | Percoidei | Percidae | *Etheostoma spectabile pulchellum* | FJ381332 | *rag1* |
| Perciformes | Percoidei | Percidae | *Etheostoma spectabile spectabile* | EF027223 | *ND2* |
| Perciformes | Percoidei | Percidae | *Etheostoma spectabile squamosum* | FJ381072 | *CYB* |
| Perciformes | Percoidei | Percidae | *Etheostoma spectabile squamosum* | FJ381288 | *ND2* |
| Perciformes | Percoidei | Percidae | *Etheostoma spectabile squamosum* | FJ381334 | *rag1* |
| Perciformes | Percoidei | Percidae | *Etheostoma spilotum* | AY964694 | *CYB* |
| Perciformes | Percoidei | Percidae | *Etheostoma squamiceps* | JN026405 | *CO1* |
| Perciformes | Percoidei | Percidae | *Etheostoma squamiceps* | AF412523 | *CYB* |
| Perciformes | Percoidei | Percidae | *Etheostoma squamiceps* | AF412537 | *ND2* |
| Perciformes | Percoidei | Percidae | *Etheostoma squamiceps* | AY251546 | *ND4* |
| Perciformes | Percoidei | Percidae | *Etheostoma stigmaeum* | JN026409 | *CO1* |
| Perciformes | Percoidei | Percidae | *Etheostoma stigmaeum* | AY374276 | *CYB* |
| Perciformes | Percoidei | Percidae | *Etheostoma stigmaeum* | JF742869 | *rag1* |
| Perciformes | Percoidei | Percidae | *Etheostoma striatulum* | JN026415 | *CO1* |
| Perciformes | Percoidei | Percidae | *Etheostoma striatulum* | AF123042 | *CYB* |
| Perciformes | Percoidei | Percidae | *Etheostoma striatulum* | FJ012653 | *ND2* |
| Perciformes | Percoidei | Percidae | *Etheostoma striatulum* | AY251547 | *ND4* |
| Perciformes | Percoidei | Percidae | *Etheostoma susanae* | GQ183636 | *CYB* |
| Perciformes | Percoidei | Percidae | *Etheostoma swaini* | JN026434 | *CO1* |
| Perciformes | Percoidei | Percidae | *Etheostoma swaini* | HM856130 | *ND2* |
| Perciformes | Percoidei | Percidae | *Etheostoma swannanoa* | JN026446 | *CO1* |
| Perciformes | Percoidei | Percidae | *Etheostoma swannanoa* | AY964709 | *CYB* |
| Perciformes | Percoidei | Percidae | *Etheostoma tallapoosae* | JN026451 | *CO1* |
| Perciformes | Percoidei | Percidae | *Etheostoma tallapoosae* | AY258505 | *CYB* |
| Perciformes | Percoidei | Percidae | *Etheostoma tecumsehi* | JN026459 | *CO1* |
| Perciformes | Percoidei | Percidae | *Etheostoma tecumsehi* | FJ381116 | *CYB* |
| Perciformes | Percoidei | Percidae | *Etheostoma tecumsehi* | EF027225 | *ND2* |
| Perciformes | Percoidei | Percidae | *Etheostoma tennesseense* | JN026460 | *CO1* |
| Perciformes | Percoidei | Percidae | *Etheostoma tennesseense* | DQ089054 | *CYB* |
| Perciformes | Percoidei | Percidae | *Etheostoma tetrazonum* | JN026462 | *CO1* |
| Perciformes | Percoidei | Percidae | *Etheostoma tetrazonum* | AF411378 | *CYB* |
| Perciformes | Percoidei | Percidae | *Etheostoma thalassinum* | JN026468 | *CO1* |
| Perciformes | Percoidei | Percidae | *Etheostoma thalassinum* | EU296692 | *CYB* |
| Perciformes | Percoidei | Percidae | *Etheostoma tippecanoe* | JN026469 | *CO1* |
| Perciformes | Percoidei | Percidae | *Etheostoma tippecanoe* | AF274466 | *CYB* |
| Perciformes | Percoidei | Percidae | *Etheostoma tippecanoe* | EU814390 | *ND2* |
| Perciformes | Percoidei | Percidae | *Etheostoma tippecanoe* | GU015838 | *rag1* |
| Perciformes | Percoidei | Percidae | *Etheostoma trisella* | JN026472 | *CO1* |
| Perciformes | Percoidei | Percidae | *Etheostoma trisella* | EF027226 | *ND2* |
| Perciformes | Percoidei | Percidae | *Etheostoma tuscumbia* | JN026474 | *CO1* |
| Perciformes | Percoidei | Percidae | *Etheostoma tuscumbia* | AY374277 | *CYB* |
| Perciformes | Percoidei | Percidae | *Etheostoma tuscumbia* | EF027227 | *ND2* |
| Perciformes | Percoidei | Percidae | *Etheostoma uniporum* | JN026491 | *CO1* |
| Perciformes | Percoidei | Percidae | *Etheostoma uniporum* | EU046696 | *CYB* |
| Perciformes | Percoidei | Percidae | *Etheostoma uniporum* | FJ381292 | *ND2* |
| Perciformes | Percoidei | Percidae | *Etheostoma uniporum* | FJ381338 | *rag1* |
| Perciformes | Percoidei | Percidae | *Etheostoma variatum* | JN026496 | *CO1* |
| Perciformes | Percoidei | Percidae | *Etheostoma variatum* | AF289266 | *CYB* |
| Perciformes | Percoidei | Percidae | *Etheostoma variatum* | EF027230 | *ND2* |
| Perciformes | Percoidei | Percidae | *Etheostoma virgatum* | JN026502 | *CO1* |
| Perciformes | Percoidei | Percidae | *Etheostoma virgatum* | FJ012506 | *CYB* |
| Perciformes | Percoidei | Percidae | *Etheostoma virgatum* | AY251552 | *ND4* |
| Perciformes | Percoidei | Percidae | *Etheostoma virgatum* | JF742868 | *rag1* |
| Perciformes | Percoidei | Percidae | *Etheostoma vitreum* | JN026507 | *CO1* |
| Perciformes | Percoidei | Percidae | *Etheostoma vitreum* | AF045347 | *CYB* |
| Perciformes | Percoidei | Percidae | *Etheostoma vitreum* | EF027231 | *ND2* |
| Perciformes | Percoidei | Percidae | *Etheostoma vitreum* | FJ381310 | *rag1* |
| Perciformes | Percoidei | Percidae | *Etheostoma vulneratum* | JN026510 | *CO1* |
| Perciformes | Percoidei | Percidae | *Etheostoma vulneratum* | EU094718 | *CYB* |
| Perciformes | Percoidei | Percidae | *Etheostoma vulneratum* | GU015842 | *rag1* |
| Perciformes | Percoidei | Percidae | *Etheostoma wapiti* | AY742668 | *CYB* |
| Perciformes | Percoidei | Percidae | *Etheostoma wapiti* | GU015848 | *rag1* |
| Perciformes | Percoidei | Percidae | *Etheostoma whipplei* | JN026559 | *CO1* |
| Perciformes | Percoidei | Percidae | *Etheostoma whipplei* | FJ381029 | *CYB* |
| Perciformes | Percoidei | Percidae | *Etheostoma whipplei* | EF027232 | *ND2* |
| Perciformes | Percoidei | Percidae | *Etheostoma whipplei* | FJ381312 | *rag1* |
| Perciformes | Percoidei | Percidae | *Etheostoma zonale* | JN026596 | *CO1* |
| Perciformes | Percoidei | Percidae | *Etheostoma zonale* | AY964706 | *CYB* |
| Perciformes | Percoidei | Percidae | *Etheostoma zonale* | EF027233 | *ND2* |
| Perciformes | Percoidei | Percidae | *Etheostoma zonale* | HM050106 | *ND4* |
| Perciformes | Percoidei | Percidae | *Etheostoma zonifer* | JN026597 | *CO1* |
| Perciformes | Percoidei | Percidae | *Etheostoma zonistium* | JN026600 | *CO1* |
| Perciformes | Percoidei | Percidae | *Etheostoma zonistium* | AF288450 | *CYB* |
| Perciformes | Percoidei | Percidae | *Gymnocephalus baloni* | AY374279 | *CYB* |
| Perciformes | Percoidei | Percidae | *Gymnocephalus cernuus* | JN026741 | *CO1* |
| Perciformes | Percoidei | Percidae | *Gymnocephalus cernuus* | AF045356 | *CYB* |
| Perciformes | Percoidei | Percidae | *Gymnocephalus cernuus* | HM050108 | *ND4* |
| Perciformes | Percoidei | Percidae | *Gymnocephalus cernuus* | AY141296 | *rhod* |
| Perciformes | Percoidei | Percidae | *Gymnocephalus schraetser* | HM049946 | *CYB* |
| Perciformes | Percoidei | Percidae | *Gymnocephalus schraetser* | HM050109 | *ND4* |
| Perciformes | Percoidei | Percidae | *Perca flavescens* | FJ826536 | *ATP6* |
| Perciformes | Percoidei | Percidae | *Perca flavescens* | EU524242 | *CO1* |
| Perciformes | Percoidei | Percidae | *Perca flavescens* | AF386600 | *CYB* |
| Perciformes | Percoidei | Percidae | *Perca flavescens* | EF027169 | *ND2* |
| Perciformes | Percoidei | Percidae | *Perca flavescens* | GU368821 | *rag1* |
| Perciformes | Percoidei | Percidae | *Perca fluviatilis* | HQ600748 | *CO1* |
| Perciformes | Percoidei | Percidae | *Perca fluviatilis* | AY374281 | *CYB* |
| Perciformes | Percoidei | Percidae | *Perca fluviatilis* | HM050129 | *ND4* |
| Perciformes | Percoidei | Percidae | *Perca schrenkii* | JN027877 | *CO1* |
| Perciformes | Percoidei | Percidae | *Perca schrenkii* | AF546118 | *CYB* |
| Perciformes | Percoidei | Percidae | *Percina antesella* | JN027878 | *CO1* |
| Perciformes | Percoidei | Percidae | *Percina antesella* | AF386587 | *CYB* |
| Perciformes | Percoidei | Percidae | *Percina aurantiaca* | JN027883 | *CO1* |
| Perciformes | Percoidei | Percidae | *Percina aurantiaca* | AF386579 | *CYB* |
| Perciformes | Percoidei | Percidae | *Percina aurantiaca* | FJ381258 | *ND2* |
| Perciformes | Percoidei | Percidae | *Percina aurantiaca* | FJ381302 | *rag1* |
| Perciformes | Percoidei | Percidae | *Percina aurolineata* | JN027891 | *CO1* |
| Perciformes | Percoidei | Percidae | *Percina aurolineata* | AF386575 | *CYB* |
| Perciformes | Percoidei | Percidae | *Percina aurora* | JN027894 | *CO1* |
| Perciformes | Percoidei | Percidae | *Percina aurora* | AF386566 | *CYB* |
| Perciformes | Percoidei | Percidae | *Percina austroperca* | JN027898 | *CO1* |
| Perciformes | Percoidei | Percidae | *Percina austroperca* | DQ493499 | *CYB* |
| Perciformes | Percoidei | Percidae | *Percina austroperca* | DQ493544 | *ND2* |
| Perciformes | Percoidei | Percidae | *Percina bimaculata* | JN028057 | *CO1* |
| Perciformes | Percoidei | Percidae | *Percina bimaculata* | EU293596 | *CYB* |
| Perciformes | Percoidei | Percidae | *Percina bimaculata* | EU293589 | *ND2* |
| Perciformes | Percoidei | Percidae | *Percina brevicauda* | AF386567 | *CYB* |
| Perciformes | Percoidei | Percidae | *Percina burtoni* | JN027907 | *CO1* |
| Perciformes | Percoidei | Percidae | *Percina burtoni* | DQ493471 | *CYB* |
| Perciformes | Percoidei | Percidae | *Percina burtoni* | DQ493508 | *ND2* |
| Perciformes | Percoidei | Percidae | *Percina caprodes* | EU524246 | *CO1* |
| Perciformes | Percoidei | Percidae | *Percina caprodes* | DQ493490 | *CYB* |
| Perciformes | Percoidei | Percidae | *Percina caprodes* | EF027178 | *ND2* |
| Perciformes | Percoidei | Percidae | *Percina caprodes* | HM050127 | *ND4* |
| Perciformes | Percoidei | Percidae | *Percina carbonaria* | JN027961 | *CO1* |
| Perciformes | Percoidei | Percidae | *Percina carbonaria* | AF386553 | *CYB* |
| Perciformes | Percoidei | Percidae | *Percina carbonaria* | DQ493536 | *ND2* |
| Perciformes | Percoidei | Percidae | *Percina copelandi* | JN027974 | *CO1* |
| Perciformes | Percoidei | Percidae | *Percina copelandi* | AY374283 | *CYB* |
| Perciformes | Percoidei | Percidae | *Percina crassa* | JN027977 | *CO1* |
| Perciformes | Percoidei | Percidae | *Percina crassa* | AF386594 | *CYB* |
| Perciformes | Percoidei | Percidae | *Percina cymatotaenia* | JN027982 | *CO1* |
| Perciformes | Percoidei | Percidae | *Percina cymatotaenia* | AF386589 | *CYB* |
| Perciformes | Percoidei | Percidae | *Percina evides* | JN027985 | *CO1* |
| Perciformes | Percoidei | Percidae | *Percina evides* | AF375943 | *CYB* |
| Perciformes | Percoidei | Percidae | *Percina evides* | DQ493545 | *ND2* |
| Perciformes | Percoidei | Percidae | *Percina fulvitaenia* | EU379104 | *CYB* |
| Perciformes | Percoidei | Percidae | *Percina fulvitaenia* | EU379090 | *ND2* |
| Perciformes | Percoidei | Percidae | *Percina gymnocephala* | JN028002 | *CO1* |
| Perciformes | Percoidei | Percidae | *Percina gymnocephala* | AF386581 | *CYB* |
| Perciformes | Percoidei | Percidae | *Percina jenkinsi* | JN028007 | *CO1* |
| Perciformes | Percoidei | Percidae | *Percina jenkinsi* | EU293565 | *CYB* |
| Perciformes | Percoidei | Percidae | *Percina jenkinsi* | EU293572 | *ND2* |
| Perciformes | Percoidei | Percidae | *Percina kathae* | JN028010 | *CO1* |
| Perciformes | Percoidei | Percidae | *Percina kathae* | AF386549 | *CYB* |
| Perciformes | Percoidei | Percidae | *Percina kathae* | DQ493538 | *ND2* |
| Perciformes | Percoidei | Percidae | *Percina kusha* | EF613218 | *CYB* |
| Perciformes | Percoidei | Percidae | *Percina lenticula* | JN028018 | *CO1* |
| Perciformes | Percoidei | Percidae | *Percina lenticula* | AF386586 | *CYB* |
| Perciformes | Percoidei | Percidae | *Percina macrocephala* | JN028025 | *CO1* |
| Perciformes | Percoidei | Percidae | *Percina macrocephala* | EF613202 | *CYB* |
| Perciformes | Percoidei | Percidae | *Percina macrocephala* | DQ493546 | *ND2* |
| Perciformes | Percoidei | Percidae | *Percina macrolepida* | JN028037 | *CO1* |
| Perciformes | Percoidei | Percidae | *Percina macrolepida* | DQ493495 | *CYB* |
| Perciformes | Percoidei | Percidae | *Percina macrolepida* | DQ493539 | *ND2* |
| Perciformes | Percoidei | Percidae | *Percina maculata* | JN028047 | *CO1* |
| Perciformes | Percoidei | Percidae | *Percina maculata* | AF386557 | *CYB* |
| Perciformes | Percoidei | Percidae | *Percina maculata* | AY517725 | *ND2* |
| Perciformes | Percoidei | Percidae | *Percina nasuta* | JN028053 | *CO1* |
| Perciformes | Percoidei | Percidae | *Percina nasuta* | AF386562 | *CYB* |
| Perciformes | Percoidei | Percidae | *Percina nevisense* | AF386596 | *CYB* |
| Perciformes | Percoidei | Percidae | *Percina nigrofasciata* | JN028063 | *CO1* |
| Perciformes | Percoidei | Percidae | *Percina nigrofasciata* | AY374286 | *CYB* |
| Perciformes | Percoidei | Percidae | *Percina notogramma* | JN028073 | *CO1* |
| Perciformes | Percoidei | Percidae | *Percina notogramma* | AF386560 | *CYB* |
| Perciformes | Percoidei | Percidae | *Percina ouachitae* | AY374287 | *CYB* |
| Perciformes | Percoidei | Percidae | *Percina oxyrhynchus* | JN028077 | *CO1* |
| Perciformes | Percoidei | Percidae | *Percina oxyrhynchus* | AF386565 | *CYB* |
| Perciformes | Percoidei | Percidae | *Percina palmaris* | JN028084 | *CO1* |
| Perciformes | Percoidei | Percidae | *Percina palmaris* | AF386583 | *CYB* |
| Perciformes | Percoidei | Percidae | *Percina pantherina* | JN028092 | *CO1* |
| Perciformes | Percoidei | Percidae | *Percina pantherina* | AF386558 | *CYB* |
| Perciformes | Percoidei | Percidae | *Percina peltata* | JN028094 | *CO1* |
| Perciformes | Percoidei | Percidae | *Percina peltata* | AF386595 | *CYB* |
| Perciformes | Percoidei | Percidae | *Percina phoxocephala* | JN028108 | *CO1* |
| Perciformes | Percoidei | Percidae | *Percina phoxocephala* | AF386563 | *CYB* |
| Perciformes | Percoidei | Percidae | *Percina rex* | JN028110 | *CO1* |
| Perciformes | Percoidei | Percidae | *Percina rex* | EU293582 | *CYB* |
| Perciformes | Percoidei | Percidae | *Percina rex* | EU293562 | *ND2* |
| Perciformes | Percoidei | Percidae | *Percina roanoka* | JN028114 | *CO1* |
| Perciformes | Percoidei | Percidae | *Percina roanoka* | AF386597 | *CYB* |
| Perciformes | Percoidei | Percidae | *Percina roanoka* | AY225722 | *ND2* |
| Perciformes | Percoidei | Percidae | *Percina roanoka* | FJ381303 | *rag1* |
| Perciformes | Percoidei | Percidae | *Percina sciera* | JN028124 | *CO1* |
| Perciformes | Percoidei | Percidae | *Percina sciera* | EF613206 | *CYB* |
| Perciformes | Percoidei | Percidae | *Percina shumardi* | JN028132 | *CO1* |
| Perciformes | Percoidei | Percidae | *Percina shumardi* | AF386572 | *CYB* |
| Perciformes | Percoidei | Percidae | *Percina sipsi* | EF613207 | *CYB* |
| Perciformes | Percoidei | Percidae | *Percina smithvanizi* | JN028146 | *CO1* |
| Perciformes | Percoidei | Percidae | *Percina smithvanizi* | EF613215 | *CYB* |
| Perciformes | Percoidei | Percidae | *Percina squamata* | JN028148 | *CO1* |
| Perciformes | Percoidei | Percidae | *Percina squamata* | AF386564 | *CYB* |
| Perciformes | Percoidei | Percidae | *Percina stictogaster* | JN028152 | *CO1* |
| Perciformes | Percoidei | Percidae | *Percina stictogaster* | AF045355 | *CYB* |
| Perciformes | Percoidei | Percidae | *Percina suttkusi* | JN028155 | *CO1* |
| Perciformes | Percoidei | Percidae | *Percina suttkusi* | AF386551 | *CYB* |
| Perciformes | Percoidei | Percidae | *Percina suttkusi* | DQ493542 | *ND2* |
| Perciformes | Percoidei | Percidae | *Percina tanasi* | JN028156 | *CO1* |
| Perciformes | Percoidei | Percidae | *Percina tanasi* | AF386578 | *CYB* |
| Perciformes | Percoidei | Percidae | *Percina uranidea* | JN028160 | *CO1* |
| Perciformes | Percoidei | Percidae | *Percina uranidea* | AF386577 | *CYB* |
| Perciformes | Percoidei | Percidae | *Percina vigil* | JN028178 | *CO1* |
| Perciformes | Percoidei | Percidae | *Percina vigil* | AF386569 | *CYB* |
| Perciformes | Percoidei | Percidae | *Romanichthys valsanicola* | AF546121 | *CYB* |
| Perciformes | Percoidei | Percidae | *Sander canadensis* | EU524371 | *CO1* |
| Perciformes | Percoidei | Percidae | *Sander canadensis* | AF386603 | *CYB* |
| Perciformes | Percoidei | Percidae | *Sander lucioperca* | JN028401 | *CO1* |
| Perciformes | Percoidei | Percidae | *Sander lucioperca* | HM049965 | *CYB* |
| Perciformes | Percoidei | Percidae | *Sander lucioperca* | HM050136 | *ND4* |
| Perciformes | Percoidei | Percidae | *Sander marinus* | EF027170 | *ND2* |
| Perciformes | Percoidei | Percidae | *Sander vitreus* | EU524380 | *CO1* |
| Perciformes | Percoidei | Percidae | *Sander vitreus* | AF045359 | *CYB* |
| Perciformes | Percoidei | Percidae | *Sander vitreus* | FJ381257 | *ND2* |
| Perciformes | Percoidei | Percidae | *Sander vitreus* | FJ381300 | *rag1* |
| Perciformes | Percoidei | Percidae | *Sander volgensis* | AY374292 | *CYB* |
| Perciformes | Percoidei | Percidae | *Zingel streber* | JN028452 | *CO1* |
| Perciformes | Percoidei | Percidae | *Zingel streber* | HM049970 | *CYB* |
| Perciformes | Percoidei | Percidae | *Zingel streber* | HM050145 | *ND4* |
| Perciformes | Percoidei | Percidae | *Zingel zingel* | JN028456 | *CO1* |
| Perciformes | Percoidei | Percidae | *Zingel zingel* | AY742669 | *CYB* |
| Perciformes | Percoidei | Priacanthidae | *Heteropriacanthus cruentatus* | DQ111381 | *ATP6* |
| Perciformes | Percoidei | Priacanthidae | *Heteropriacanthus cruentatus* | EU871696 | *CO1* |
| Perciformes | Percoidei | Priacanthidae | *Heteropriacanthus cruentatus* | EF392590 | *CYB* |
| Perciformes | Percoidei | Priacanthidae | *Heteropriacanthus cruentatus* | EF427478 | *rhod* |
| Perciformes | Percoidei | Priacanthidae | *Priacanthus arenatus* | EU637997 | *rhod* |
| Perciformes | Percoidei | Priacanthidae | *Priacanthus hamrur* | DQ885117 | *CO1* |
| Perciformes | Percoidei | Priacanthidae | *Priacanthus hamrur* | HM037262 | *CYB* |
| Perciformes | Percoidei | Priacanthidae | *Priacanthus hamrur* | EU167865 | *rag1* |
| Perciformes | Percoidei | Priacanthidae | *Priacanthus macracanthus* | EU595246 | *CO1* |
| Perciformes | Percoidei | Priacanthidae | *Priacanthus meeki* | DQ521025 | *CO1* |
| Perciformes | Percoidei | Priacanthidae | *Priacanthus tayenus* | EF609435 | *CO1* |
| Perciformes | Percoidei | Priacanthidae | *Priacanthus tayenus* | EU167866 | *rag1* |
| Perciformes | Percoidei | Priacanthidae | *Pristigenys alta* | GU368822 | *rag1* |
| Perciformes | Percoidei | Priacanthidae | *Pristigenys serrula* | GU440479 | *CO1* |
| Perciformes | Percoidei | Apogonidae | *Apogon angustatus* | FJ346791 | *CO1* |
| Perciformes | Percoidei | Apogonidae | *Apogon angustatus* | FJ346748 | *ND1* |
| Perciformes | Percoidei | Apogonidae | *Apogon angustatus* | FJ346706 | *ND2* |
| Perciformes | Percoidei | Apogonidae | *Apogon apogonoides* | FJ918953 | *CO1* |
| Perciformes | Percoidei | Apogonidae | *Apogon apogonoides* | FJ896469 | *rag1* |
| Perciformes | Percoidei | Apogonidae | *Apogon aureus* | EU167749 | *rag1* |
| Perciformes | Percoidei | Apogonidae | *Apogon aurolineatus* | FJ346800 | *CO1* |
| Perciformes | Percoidei | Apogonidae | *Apogon aurolineatus* | FJ346758 | *ND1* |
| Perciformes | Percoidei | Apogonidae | *Apogon aurolineatus* | FJ346714 | *ND2* |
| Perciformes | Percoidei | Apogonidae | *Apogon cookii* | FJ346792 | *CO1* |
| Perciformes | Percoidei | Apogonidae | *Apogon cookii* | FJ346749 | *ND1* |
| Perciformes | Percoidei | Apogonidae | *Apogon cookii* | FJ346708 | *ND2* |
| Perciformes | Percoidei | Apogonidae | *Apogon doederleini* | EU381023 | *CO1* |
| Perciformes | Percoidei | Apogonidae | *Apogon doederleini* | EU380969 | *CYB* |
| Perciformes | Percoidei | Apogonidae | *Apogon doederleini* | EU380981 | *ND1* |
| Perciformes | Percoidei | Apogonidae | *Apogon doederleini* | EU381003 | *ND2* |
| Perciformes | Percoidei | Apogonidae | *Apogon dovii* | FJ582860 | *CO1* |
| Perciformes | Percoidei | Apogonidae | *Apogon endekataenia* | JF952672 | *CO1* |
| Perciformes | Percoidei | Apogonidae | *Apogon erythrinus* | EF607315 | *CO1* |
| Perciformes | Percoidei | Apogonidae | *Apogon exostigma* | FJ346785 | *CO1* |
| Perciformes | Percoidei | Apogonidae | *Apogon exostigma* | EU380960 | *CYB* |
| Perciformes | Percoidei | Apogonidae | *Apogon exostigma* | EU380984 | *ND1* |
| Perciformes | Percoidei | Apogonidae | *Apogon exostigma* | FJ346702 | *ND2* |
| Perciformes | Percoidei | Apogonidae | *Apogon flagelliferus* | FJ346756 | *ND1* |
| Perciformes | Percoidei | Apogonidae | *Apogon flagelliferus* | FJ346713 | *ND2* |
| Perciformes | Percoidei | Apogonidae | *Apogon holotaenia* | FJ346745 | *ND1* |
| Perciformes | Percoidei | Apogonidae | *Apogon holotaenia* | FJ346705 | *ND2* |
| Perciformes | Percoidei | Apogonidae | *Apogon hyalosoma* | HQ654660 | *CO1* |
| Perciformes | Percoidei | Apogonidae | *Apogon kallopterus* | FJ346793 | *CO1* |
| Perciformes | Percoidei | Apogonidae | *Apogon kallopterus* | DQ821428 | *CYB* |
| Perciformes | Percoidei | Apogonidae | *Apogon kallopterus* | FJ346751 | *ND1* |
| Perciformes | Percoidei | Apogonidae | *Apogon kallopterus* | FJ346710 | *ND2* |
| Perciformes | Percoidei | Apogonidae | *Apogon lateralis* | FJ346753 | *ND1* |
| Perciformes | Percoidei | Apogonidae | *Apogon leslie* | DQ821431 | *CYB* |
| Perciformes | Percoidei | Apogonidae | *Apogon lineatus* | GU357853 | *CO1* |
| Perciformes | Percoidei | Apogonidae | *Apogon luteus* | DQ821432 | *CYB* |
| Perciformes | Percoidei | Apogonidae | *Apogon maculatus* | EU381025 | *CO1* |
| Perciformes | Percoidei | Apogonidae | *Apogon maculatus* | EU380970 | *CYB* |
| Perciformes | Percoidei | Apogonidae | *Apogon maculatus* | AY722254 | *ND1* |
| Perciformes | Percoidei | Apogonidae | *Apogon maculatus* | EU381005 | *ND2* |
| Perciformes | Percoidei | Apogonidae | *Apogon natalensis* | GU805076 | *CO1* |
| Perciformes | Percoidei | Apogonidae | *Apogon nigrofasciatus* | FJ346786 | *CO1* |
| Perciformes | Percoidei | Apogonidae | *Apogon nigrofasciatus* | AY722256 | *ND1* |
| Perciformes | Percoidei | Apogonidae | *Apogon nigrofasciatus* | FJ346703 | *ND2* |
| Perciformes | Percoidei | Apogonidae | *Apogon norfolcensis* | FJ237580 | *CO1* |
| Perciformes | Percoidei | Apogonidae | *Apogon pacificus* | HQ010094 | *CO1* |
| Perciformes | Percoidei | Apogonidae | *Apogon phenax* | GU224703 | *CO1* |
| Perciformes | Percoidei | Apogonidae | *Apogon quadrifasciatus* | EU148586 | *CO1* |
| Perciformes | Percoidei | Apogonidae | *Apogon quadrifasciatus* | EU637940 | *rhod* |
| Perciformes | Percoidei | Apogonidae | *Apogon quadrisquamatus* | FJ346787 | *CO1* |
| Perciformes | Percoidei | Apogonidae | *Apogon quadrisquamatus* | AY722183 | *CYB* |
| Perciformes | Percoidei | Apogonidae | *Apogon quadrisquamatus* | AY722255 | *ND1* |
| Perciformes | Percoidei | Apogonidae | *Apogon sealei* | FJ582864 | *CO1* |
| Perciformes | Percoidei | Apogonidae | *Apogon semilineatus* | JF952679 | *CO1* |
| Perciformes | Percoidei | Apogonidae | *Apogon semilineatus* | AB018995 | *CYB* |
| Perciformes | Percoidei | Apogonidae | *Apogon semilineatus* | AB018980 | *ND2* |
| Perciformes | Percoidei | Apogonidae | *Apogon townsendi* | FJ582867 | *CO1* |
| Perciformes | Percoidei | Apogonidae | *Apogon trimaculatus* | FJ346798 | *CO1* |
| Perciformes | Percoidei | Apogonidae | *Apogon trimaculatus* | FJ346755 | *ND1* |
| Perciformes | Percoidei | Apogonidae | *Apogon trimaculatus* | FJ346712 | *ND2* |
| Perciformes | Percoidei | Apogonidae | *Archamia biguttata* | FJ346782 | *CO1* |
| Perciformes | Percoidei | Apogonidae | *Archamia biguttata* | EU380968 | *CYB* |
| Perciformes | Percoidei | Apogonidae | *Archamia biguttata* | FJ346739 | *ND1* |
| Perciformes | Percoidei | Apogonidae | *Archamia biguttata* | FJ346700 | *ND2* |
| Perciformes | Percoidei | Apogonidae | *Archamia fucata* | EU381021 | *CO1* |
| Perciformes | Percoidei | Apogonidae | *Archamia fucata* | EU380959 | *CYB* |
| Perciformes | Percoidei | Apogonidae | *Archamia fucata* | EU380980 | *ND1* |
| Perciformes | Percoidei | Apogonidae | *Archamia fucata* | EU381001 | *ND2* |
| Perciformes | Percoidei | Apogonidae | *Astrapogon alutus* | FJ609906 | *CO1* |
| Perciformes | Percoidei | Apogonidae | *Astrapogon puncticulatus* | FJ346822 | *CO1* |
| Perciformes | Percoidei | Apogonidae | *Astrapogon puncticulatus* | FJ346779 | *ND1* |
| Perciformes | Percoidei | Apogonidae | *Astrapogon stellatus* | FJ609930 | *CO1* |
| Perciformes | Percoidei | Apogonidae | *Cercamia eremia* | FJ346814 | *CO1* |
| Perciformes | Percoidei | Apogonidae | *Cercamia eremia* | FJ346771 | *ND1* |
| Perciformes | Percoidei | Apogonidae | *Cercamia eremia* | FJ346730 | *ND2* |
| Perciformes | Percoidei | Apogonidae | *Cheilodipterus artus* | FJ346765 | *ND1* |
| Perciformes | Percoidei | Apogonidae | *Cheilodipterus artus* | FJ346722 | *ND2* |
| Perciformes | Percoidei | Apogonidae | *Cheilodipterus isostigmus* | FJ346808 | *CO1* |
| Perciformes | Percoidei | Apogonidae | *Cheilodipterus isostigmus* | FJ346766 | *ND1* |
| Perciformes | Percoidei | Apogonidae | *Cheilodipterus isostigmus* | FJ346724 | *ND2* |
| Perciformes | Percoidei | Apogonidae | *Cheilodipterus macrodon* | EU381027 | *CO1* |
| Perciformes | Percoidei | Apogonidae | *Cheilodipterus macrodon* | EU380972 | *CYB* |
| Perciformes | Percoidei | Apogonidae | *Cheilodipterus macrodon* | EU380985 | *ND1* |
| Perciformes | Percoidei | Apogonidae | *Cheilodipterus macrodon* | EU381007 | *ND2* |
| Perciformes | Percoidei | Apogonidae | *Cheilodipterus macrodon* | EU167750 | *rag1* |
| Perciformes | Percoidei | Apogonidae | *Fowleria aurita* | FJ346802 | *CO1* |
| Perciformes | Percoidei | Apogonidae | *Fowleria aurita* | EU380973 | *CYB* |
| Perciformes | Percoidei | Apogonidae | *Fowleria aurita* | EU380986 | *ND1* |
| Perciformes | Percoidei | Apogonidae | *Fowleria aurita* | EU381008 | *ND2* |
| Perciformes | Percoidei | Apogonidae | *Fowleria isostigma* | FJ346803 | *CO1* |
| Perciformes | Percoidei | Apogonidae | *Fowleria isostigma* | FJ346760 | *ND1* |
| Perciformes | Percoidei | Apogonidae | *Fowleria isostigma* | FJ346717 | *ND2* |
| Perciformes | Percoidei | Apogonidae | *Fowleria marmorata* | FJ346806 | *CO1* |
| Perciformes | Percoidei | Apogonidae | *Fowleria marmorata* | FJ346761 | *ND1* |
| Perciformes | Percoidei | Apogonidae | *Fowleria marmorata* | FJ346720 | *ND2* |
| Perciformes | Percoidei | Apogonidae | *Fowleria variegata* | FJ346804 | *CO1* |
| Perciformes | Percoidei | Apogonidae | *Fowleria variegata* | FJ346719 | *ND2* |
| Perciformes | Percoidei | Apogonidae | *Glossamia aprion* | FJ346812 | *CO1* |
| Perciformes | Percoidei | Apogonidae | *Glossamia aprion* | FJ346770 | *ND1* |
| Perciformes | Percoidei | Apogonidae | *Glossamia aprion* | FJ346728 | *ND2* |
| Perciformes | Percoidei | Apogonidae | *Gymnapogon urospilotus* | FJ346817 | *CO1* |
| Perciformes | Percoidei | Apogonidae | *Gymnapogon urospilotus* | FJ346775 | *ND1* |
| Perciformes | Percoidei | Apogonidae | *Gymnapogon urospilotus* | FJ346734 | *ND2* |
| Perciformes | Percoidei | Apogonidae | *Nectamia fusca* | EF607316 | *CO1* |
| Perciformes | Percoidei | Apogonidae | *Phaeoptyx conklini* | FJ346811 | *CO1* |
| Perciformes | Percoidei | Apogonidae | *Phaeoptyx conklini* | FJ346769 | *ND1* |
| Perciformes | Percoidei | Apogonidae | *Phaeoptyx pigmentaria* | FJ609840 | *CO1* |
| Perciformes | Percoidei | Apogonidae | *Phaeoptyx xenus* | FJ609904 | *CO1* |
| Perciformes | Percoidei | Apogonidae | *Pseudamia gelatinosa* | FJ346819 | *CO1* |
| Perciformes | Percoidei | Apogonidae | *Pseudamia gelatinosa* | FJ346776 | *ND1* |
| Perciformes | Percoidei | Apogonidae | *Pseudamia gelatinosa* | AF391538 | *ND2* |
| Perciformes | Percoidei | Apogonidae | *Pterapogon kauderni* | FJ346767 | *ND1* |
| Perciformes | Percoidei | Apogonidae | *Pterapogon kauderni* | FJ346726 | *ND2* |
| Perciformes | Percoidei | Apogonidae | *Rhabdamia cypselura* | FJ346816 | *CO1* |
| Perciformes | Percoidei | Apogonidae | *Rhabdamia cypselura* | FJ346772 | *ND1* |
| Perciformes | Percoidei | Apogonidae | *Rhabdamia cypselura* | FJ346731 | *ND2* |
| Perciformes | Percoidei | Apogonidae | *Rhabdamia gracilis* | FJ584050 | *CO1* |
| Perciformes | Percoidei | Apogonidae | *Siphamia versicolor* | DQ790846 | *CO1* |
| Perciformes | Percoidei | Apogonidae | *Sphaeramia nematoptera* | FJ584122 | *CO1* |
| Perciformes | Percoidei | Apogonidae | *Sphaeramia nematoptera* | EU638010 | *rhod* |
| Perciformes | Percoidei | Apogonidae | *Sphaeramia orbicularis* | FJ346823 | *CO1* |
| Perciformes | Percoidei | Apogonidae | *Sphaeramia orbicularis* | FJ346780 | *ND1* |
| Perciformes | Percoidei | Apogonidae | *Sphaeramia orbicularis* | FJ346738 | *ND2* |
| Perciformes | Percoidei | Apogonidae | *Zoramia fragilis* | FJ346741 | *ND1* |
| Perciformes | Percoidei | Apogonidae | *Zoramia fragilis* | FJ346701 | *ND2* |
| Perciformes | Percoidei | Epigonidae | *Epigonus angustifrons* | AB639883 | *CO1* |
| Perciformes | Percoidei | Epigonidae | *Epigonus telescopus* | HM007702 | *CO1* |
| Perciformes | Percoidei | Epigonidae | *Epigonus telescopus* | EU167904 | *rag1* |
| Perciformes | Percoidei | Epigonidae | *Epigonus telescopus* | EU637959 | *rhod* |
| Perciformes | Percoidei | Sillaginidae | *Sillaginodes punctatus* | EF609465 | *CO1* |
| Perciformes | Percoidei | Sillaginidae | *Sillago aeolus* | HM131473 | *CO1* |
| Perciformes | Percoidei | Sillaginidae | *Sillago aeolus* | HM131459 | *CYB* |
| Perciformes | Percoidei | Sillaginidae | *Sillago aeolus* | EU167873 | *rag1* |
| Perciformes | Percoidei | Sillaginidae | *Sillago analis* | FJ223181 | *CO1* |
| Perciformes | Percoidei | Sillaginidae | *Sillago bassensis* | EF609466 | *CO1* |
| Perciformes | Percoidei | Sillaginidae | *Sillago bassensis* | HM131464 | *CYB* |
| Perciformes | Percoidei | Sillaginidae | *Sillago ciliata* | FJ223186 | *CO1* |
| Perciformes | Percoidei | Sillaginidae | *Sillago flindersi* | EF609468 | *CO1* |
| Perciformes | Percoidei | Sillaginidae | *Sillago ingenuua* | FJ155368 | *CO1* |
| Perciformes | Percoidei | Sillaginidae | *Sillago japonica* | JF952858 | *CO1* |
| Perciformes | Percoidei | Sillaginidae | *Sillago japonica* | HM131468 | *CYB* |
| Perciformes | Percoidei | Sillaginidae | *Sillago maculata* | FJ223187 | *CO1* |
| Perciformes | Percoidei | Sillaginidae | *Sillago robusta* | EF609470 | *CO1* |
| Perciformes | Percoidei | Sillaginidae | *Sillago sihama* | EU871704 | *CO1* |
| Perciformes | Percoidei | Sillaginidae | *Sillago sihama* | HM131458 | *CYB* |
| Perciformes | Percoidei | Sillaginidae | *Sillago sihama* | EU167874 | *rag1* |
| Perciformes | Percoidei | Sillaginidae | *Sillago sihama* | EU638008 | *rhod* |
| Perciformes | Percoidei | Malacanthidae | *Branchiostegus albus* | AB561163 | *CO1* |
| Perciformes | Percoidei | Malacanthidae | *Branchiostegus albus* | EU821593 | *CYB* |
| Perciformes | Percoidei | Malacanthidae | *Branchiostegus albus* | AB602482 | *rag1* |
| Perciformes | Percoidei | Malacanthidae | *Branchiostegus argentatus* | FJ237635 | *CO1* |
| Perciformes | Percoidei | Malacanthidae | *Branchiostegus argentatus* | EU821591 | *CYB* |
| Perciformes | Percoidei | Malacanthidae | *Branchiostegus argentatus* | AB602480 | *rag1* |
| Perciformes | Percoidei | Malacanthidae | *Branchiostegus auratus* | AB561160 | *CO1* |
| Perciformes | Percoidei | Malacanthidae | *Branchiostegus auratus* | EU821585 | *CYB* |
| Perciformes | Percoidei | Malacanthidae | *Branchiostegus auratus* | AB602484 | *rag1* |
| Perciformes | Percoidei | Malacanthidae | *Branchiostegus doliatus* | GU804908 | *CO1* |
| Perciformes | Percoidei | Malacanthidae | *Branchiostegus japonicus* | JF952691 | *CO1* |
| Perciformes | Percoidei | Malacanthidae | *Branchiostegus japonicus* | EU821587 | *CYB* |
| Perciformes | Percoidei | Malacanthidae | *Branchiostegus japonicus* | AB602486 | *rag1* |
| Perciformes | Percoidei | Malacanthidae | *Branchiostegus semifasciatus* | EU167909 | *rag1* |
| Perciformes | Percoidei | Malacanthidae | *Branchiostegus wardi* | EU821589 | *CYB* |
| Perciformes | Percoidei | Malacanthidae | *Caulolatilus affinis* | GU440265 | *CO1* |
| Perciformes | Percoidei | Malacanthidae | *Caulolatilus microps* | EU167907 | *rag1* |
| Perciformes | Percoidei | Malacanthidae | *Caulolatilus princeps* | GU440266 | *CO1* |
| Perciformes | Percoidei | Malacanthidae | *Hoplolatilus chlupatyi* | FJ583570 | *CO1* |
| Perciformes | Percoidei | Malacanthidae | *Hoplolatilus marcosi* | FJ583571 | *CO1* |
| Perciformes | Percoidei | Malacanthidae | *Hoplolatilus purpureus* | FJ583576 | *CO1* |
| Perciformes | Percoidei | Malacanthidae | *Lopholatilus chamaeleonticeps* | EU167845 | *rag1* |
| Perciformes | Percoidei | Malacanthidae | *Lopholatilus chamaeleonticeps* | EU637973 | *rhod* |
| Perciformes | Percoidei | Malacanthidae | *Lopholatilus villarii* | EU074447 | *CO1* |
| Perciformes | Percoidei | Malacanthidae | *Malacanthus brevirostris* | HQ945947 | *CO1* |
| Perciformes | Percoidei | Lactariidae | *Lactarius lactarius* | EF609529 | *CO1* |
| Perciformes | Percoidei | Dinolestidae | *Dinolestes lewini* | EU167828 | *rag1* |
| Perciformes | Percoidei | Scombropidae | *Scombrops boops* | HQ945916 | *CO1* |
| Perciformes | Percoidei | Scombropidae | *Scombrops boops* | AB473211 | *CYB* |
| Perciformes | Percoidei | Scombropidae | *Scombrops gilberti* | JF952850 | *CO1* |
| Perciformes | Percoidei | Scombropidae | *Scombrops gilberti* | AB550680 | *CYB* |
| Perciformes | Percoidei | Pomatomidae | *Pomatomus saltator* | AF143199 | *CYB* |
| Perciformes | Percoidei | Pomatomidae | *Pomatomus saltatrix* | EU263791 | *CO1* |
| Perciformes | Percoidei | Pomatomidae | *Pomatomus saltatrix* | EU036479 | *CYB* |
| Perciformes | Percoidei | Pomatomidae | *Pomatomus saltatrix* | FJ374825 | *ND2* |
| Perciformes | Percoidei | Pomatomidae | *Pomatomus saltatrix* | EU167741 | *rag1* |
| Perciformes | Percoidei | Pomatomidae | *Pomatomus saltatrix* | DQ080430 | *rhod* |
| Perciformes | Percoidei | Nematistiidae | *Nematistius pectoralis* | GU440421 | *CO1* |
| Perciformes | Percoidei | Nematistiidae | *Nematistius pectoralis* | AY050751 | *CYB* |
| Perciformes | Percoidei | Nematistiidae | *Nematistius pectoralis* | FJ374835 | *ND2* |
| Perciformes | Percoidei | Nematistiidae | *Nematistius pectoralis* | EU167756 | *rag1* |
| Perciformes | Percoidei | Coryphaenidae | *Coryphaena equiselis* | DQ080244 | *CYB* |
| Perciformes | Percoidei | Coryphaenidae | *Coryphaena equiselis* | FJ374827 | *ND2* |
| Perciformes | Percoidei | Coryphaenidae | *Coryphaena equiselis* | DQ080342 | *rhod* |
| Perciformes | Percoidei | Coryphaenidae | *Coryphaena hippurus* | DQ080245 | *CYB* |
| Perciformes | Percoidei | Coryphaenidae | *Coryphaena hippurus* | AF290388 | *ND1* |
| Perciformes | Percoidei | Coryphaenidae | *Coryphaena hippurus* | FJ374826 | *ND2* |
| Perciformes | Percoidei | Coryphaenidae | *Coryphaena hippurus* | EU167822 | *rag1* |
| Perciformes | Percoidei | Coryphaenidae | *Coryphaena hippurus* | DQ874792 | *rag2* |
| Perciformes | Percoidei | Coryphaenidae | *Coryphaena hippurus* | DQ080343 | *rhod* |
| Perciformes | Percoidei | Rachycentridae | *Rachycentron canadum* | HQ589294 | *CO1* |
| Perciformes | Percoidei | Rachycentridae | *Rachycentron canadum* | AB292793 | *CYB* |
| Perciformes | Percoidei | Rachycentridae | *Rachycentron canadum* | AY249491 | *ND2* |
| Perciformes | Percoidei | Rachycentridae | *Rachycentron canadum* | EU167910 | *rag1* |
| Perciformes | Percoidei | Echeneidae | *Echeneis naucrates* | GU440307 | *CO1* |
| Perciformes | Percoidei | Echeneidae | *Echeneis naucrates* | AY050763 | *CYB* |
| Perciformes | Percoidei | Echeneidae | *Echeneis naucrates* | FJ374829 | *ND2* |
| Perciformes | Percoidei | Echeneidae | *Echeneis naucrates* | EU167829 | *rag1* |
| Perciformes | Percoidei | Echeneidae | *Echeneis naucrates* | AY141315 | *rhod* |
| Perciformes | Percoidei | Echeneidae | *Echeneis neucratoides* | FJ374833 | *ND2* |
| Perciformes | Percoidei | Echeneidae | *Ecsenius bicolor* | FJ583382 | *CO1* |
| Perciformes | Percoidei | Echeneidae | *Phtheirichthys lineatus* | GU440459 | *CO1* |
| Perciformes | Percoidei | Echeneidae | *Phtheirichthys lineatus* | FJ374836 | *ND2* |
| Perciformes | Percoidei | Echeneidae | *Remora australis* | GU440495 | *CO1* |
| Perciformes | Percoidei | Echeneidae | *Remora australis* | FJ374837 | *ND2* |
| Perciformes | Percoidei | Echeneidae | *Remora australis* | EU167871 | *rag1* |
| Perciformes | Percoidei | Echeneidae | *Remora brachyptera* | GU440496 | *CO1* |
| Perciformes | Percoidei | Echeneidae | *Remora brachyptera* | FJ374834 | *ND2* |
| Perciformes | Percoidei | Echeneidae | *Remora osteochir* | JF952833 | *CO1* |
| Perciformes | Percoidei | Echeneidae | *Remora osteochir* | FJ374832 | *ND2* |
| Perciformes | Percoidei | Echeneidae | *Remora remora* | GU440498 | *CO1* |
| Perciformes | Percoidei | Echeneidae | *Remora remora* | FJ374830 | *ND2* |
| Perciformes | Percoidei | Echeneidae | *Remorina albescens* | GU440499 | *CO1* |
| Perciformes | Percoidei | Echeneidae | *Remorina albescens* | FJ374828 | *ND2* |
| Perciformes | Percoidei | Carangidae | *Alectis alexandrinus* | AF363738 | *CYB* |
| Perciformes | Percoidei | Carangidae | *Alectis alexandrinus* | EU167759 | *rag1* |
| Perciformes | Percoidei | Carangidae | *Alectis ciliaris* | EF609280 | *CO1* |
| Perciformes | Percoidei | Carangidae | *Alectis ciliaris* | AF363739 | *CYB* |
| Perciformes | Percoidei | Carangidae | *Alectis indicus* | FJ347934 | *CO1* |
| Perciformes | Percoidei | Carangidae | *Alectis indicus* | EF512290 | *CYB* |
| Perciformes | Percoidei | Carangidae | *Alepes djedaba* | EF609501 | *CO1* |
| Perciformes | Percoidei | Carangidae | *Alepes djedaba* | EF512295 | *CYB* |
| Perciformes | Percoidei | Carangidae | *Alepes kleinii* | FJ347910 | *CO1* |
| Perciformes | Percoidei | Carangidae | *Alepes kleinii* | EF512293 | *CYB* |
| Perciformes | Percoidei | Carangidae | *Atropus atropos* | EF609506 | *CO1* |
| Perciformes | Percoidei | Carangidae | *Atropus atropos* | AY050729 | *CYB* |
| Perciformes | Percoidei | Carangidae | *Atule mate* | EF609293 | *CO1* |
| Perciformes | Percoidei | Carangidae | *Carangoides bartholomaei* | GU225171 | *CO1* |
| Perciformes | Percoidei | Carangidae | *Carangoides bartholomaei* | AY050728 | *CYB* |
| Perciformes | Percoidei | Carangidae | *Carangoides chrysophrys* | FJ237546 | *CO1* |
| Perciformes | Percoidei | Carangidae | *Carangoides chrysophrys* | EF512292 | *CYB* |
| Perciformes | Percoidei | Carangidae | *Carangoides coeruleopinnatus* | GU804971 | *CO1* |
| Perciformes | Percoidei | Carangidae | *Carangoides equula* | AY541645 | *CO1* |
| Perciformes | Percoidei | Carangidae | *Carangoides fulvoguttatus* | EF609302 | *CO1* |
| Perciformes | Percoidei | Carangidae | *Carangoides malabaricus* | FJ347878 | *CO1* |
| Perciformes | Percoidei | Carangidae | *Carangoides otrynter* | AF363741 | *CYB* |
| Perciformes | Percoidei | Carangidae | *Carangoides praeustus* | FJ237970 | *CO1* |
| Perciformes | Percoidei | Carangidae | *Carangoides ruber* | GU225166 | *CO1* |
| Perciformes | Percoidei | Carangidae | *Carangoides ruber* | AY050723 | *CYB* |
| Perciformes | Percoidei | Carangidae | *Caranx bucculentus* | EF609303 | *CO1* |
| Perciformes | Percoidei | Carangidae | *Caranx caballus* | AF363747 | *CYB* |
| Perciformes | Percoidei | Carangidae | *Caranx caninus* | EU752066 | *CO1* |
| Perciformes | Percoidei | Carangidae | *Caranx caninus* | AY050715 | *CYB* |
| Perciformes | Percoidei | Carangidae | *Caranx carangus* | EU514501 | *CO1* |
| Perciformes | Percoidei | Carangidae | *Caranx crysos* | GU224385 | *CO1* |
| Perciformes | Percoidei | Carangidae | *Caranx crysos* | EF392575 | *CYB* |
| Perciformes | Percoidei | Carangidae | *Caranx crysos* | EF427460 | *rhod* |
| Perciformes | Percoidei | Carangidae | *Caranx hippos* | FJ347906 | *CO1* |
| Perciformes | Percoidei | Carangidae | *Caranx hippos* | AY050720 | *CYB* |
| Perciformes | Percoidei | Carangidae | *Caranx hippos* | EU167758 | *rag1* |
| Perciformes | Percoidei | Carangidae | *Caranx ignobilis* | EU131644 | *ATP6* |
| Perciformes | Percoidei | Carangidae | *Caranx ignobilis* | DQ885073 | *CO1* |
| Perciformes | Percoidei | Carangidae | *Caranx latus* | AY050724 | *CYB* |
| Perciformes | Percoidei | Carangidae | *Caranx latus* | EU477492 | *rag1* |
| Perciformes | Percoidei | Carangidae | *Caranx melampygus* | EU131652 | *ATP6* |
| Perciformes | Percoidei | Carangidae | *Caranx melampygus* | EF609304 | *CO1* |
| Perciformes | Percoidei | Carangidae | *Caranx papuensis* | GU805039 | *CO1* |
| Perciformes | Percoidei | Carangidae | *Caranx rhonchus* | AY050733 | *CYB* |
| Perciformes | Percoidei | Carangidae | *Caranx sexfasciatus* | EF609305 | *CO1* |
| Perciformes | Percoidei | Carangidae | *Caranx sexfasciatus* | AY050760 | *CYB* |
| Perciformes | Percoidei | Carangidae | *Caranx tille* | GU805027 | *CO1* |
| Perciformes | Percoidei | Carangidae | *Caranx vinctus* | GU440258 | *CO1* |
| Perciformes | Percoidei | Carangidae | *Caranx vinctus* | AY050722 | *CYB* |
| Perciformes | Percoidei | Carangidae | *Chloroscombrus chrysurus* | AY050752 | *CYB* |
| Perciformes | Percoidei | Carangidae | *Chloroscombrus chrysurus* | AY141313 | *rhod* |
| Perciformes | Percoidei | Carangidae | *Chloroscombrus orqueta* | AY050749 | *CYB* |
| Perciformes | Percoidei | Carangidae | *Decapterus akaadsi* | EF607334 | *CO1* |
| Perciformes | Percoidei | Carangidae | *Decapterus akaadsi* | AB374075 | *CYB* |
| Perciformes | Percoidei | Carangidae | *Decapterus macarellus* | EU514517 | *CO1* |
| Perciformes | Percoidei | Carangidae | *Decapterus macarellus* | AB372035 | *CYB* |
| Perciformes | Percoidei | Carangidae | *Decapterus macrosoma* | EU514515 | *CO1* |
| Perciformes | Percoidei | Carangidae | *Decapterus macrosoma* | AB374073 | *CYB* |
| Perciformes | Percoidei | Carangidae | *Decapterus macrosoma* | EU167757 | *rag1* |
| Perciformes | Percoidei | Carangidae | *Decapterus maruadsi* | EF512291 | *CYB* |
| Perciformes | Percoidei | Carangidae | *Decapterus muroadsi* | HQ010055 | *CO1* |
| Perciformes | Percoidei | Carangidae | *Decapterus muroadsi* | AB374072 | *CYB* |
| Perciformes | Percoidei | Carangidae | *Decapterus punctatus* | GU224777 | *CO1* |
| Perciformes | Percoidei | Carangidae | *Decapterus punctatus* | AY050732 | *CYB* |
| Perciformes | Percoidei | Carangidae | *Decapterus russelli* | EU514513 | *CO1* |
| Perciformes | Percoidei | Carangidae | *Decapterus tabl* | AB374074 | *CYB* |
| Perciformes | Percoidei | Carangidae | *Elagatis bipinnulata* | EU014213 | *CO1* |
| Perciformes | Percoidei | Carangidae | *Elagatis bipinnulata* | AY050743 | *CYB* |
| Perciformes | Percoidei | Carangidae | *Gnathanodon speciosus* | EU148563 | *CO1* |
| Perciformes | Percoidei | Carangidae | *Gnathanodon speciosus* | EU637963 | *rhod* |
| Perciformes | Percoidei | Carangidae | *Hemicaranx leucurus* | AY050716 | *CYB* |
| Perciformes | Percoidei | Carangidae | *Hemicaranx zelotes* | AY050735 | *CYB* |
| Perciformes | Percoidei | Carangidae | *Lichia amia* | EF392594 | *CYB* |
| Perciformes | Percoidei | Carangidae | *Lichia amia* | EF427482 | *rhod* |
| Perciformes | Percoidei | Carangidae | *Megalaspis cordyla* | EF609548 | *CO1* |
| Perciformes | Percoidei | Carangidae | *Naucrates ductor* | HQ010068 | *CO1* |
| Perciformes | Percoidei | Carangidae | *Oligoplites altus* | AY050758 | *CYB* |
| Perciformes | Percoidei | Carangidae | *Oligoplites saurus* | GU225649 | *CO1* |
| Perciformes | Percoidei | Carangidae | *Oligoplites saurus* | AY050757 | *CYB* |
| Perciformes | Percoidei | Carangidae | *Parastromateus niger* | EF609429 | *CO1* |
| Perciformes | Percoidei | Carangidae | *Parastromateus niger* | EF095654 | *rag1* |
| Perciformes | Percoidei | Carangidae | *Parastromateus niger* | EF095616 | *rhod* |
| Perciformes | Percoidei | Carangidae | *Parona signata* | EU074524 | *CO1* |
| Perciformes | Percoidei | Carangidae | *Pseudocaranx dentex* | EF609442 | *CO1* |
| Perciformes | Percoidei | Carangidae | *Pseudocaranx dentex* | EF392607 | *CYB* |
| Perciformes | Percoidei | Carangidae | *Pseudocaranx dentex* | EF439301 | *rhod* |
| Perciformes | Percoidei | Carangidae | *Pseudocaranx wrighti* | EF609443 | *CO1* |
| Perciformes | Percoidei | Carangidae | *Scomberoides commersonnianus* | EF609456 | *CO1* |
| Perciformes | Percoidei | Carangidae | *Scomberoides commersonnianus* | EF512294 | *CYB* |
| Perciformes | Percoidei | Carangidae | *Scomberoides lysan* | EF609611 | *CO1* |
| Perciformes | Percoidei | Carangidae | *Scomberoides tol* | DQ885124 | *CO1* |
| Perciformes | Percoidei | Carangidae | *Selar boops* | FJ347891 | *CO1* |
| Perciformes | Percoidei | Carangidae | *Selar crumenophthalmus* | FJ347941 | *CO1* |
| Perciformes | Percoidei | Carangidae | *Selar crumenophthalmus* | AY050731 | *CYB* |
| Perciformes | Percoidei | Carangidae | *Selar crumenophthalmus* | DQ874788 | *rag2* |
| Perciformes | Percoidei | Carangidae | *Selar crumenophthalmus* | DQ874820 | *rhod* |
| Perciformes | Percoidei | Carangidae | *Selaroides leptolepis* | EF607548 | *CO1* |
| Perciformes | Percoidei | Carangidae | *Selaroides leptolepis* | EF512296 | *CYB* |
| Perciformes | Percoidei | Carangidae | *Selene brevoortii* | GU440520 | *CO1* |
| Perciformes | Percoidei | Carangidae | *Selene brevoortii* | AF363749 | *CYB* |
| Perciformes | Percoidei | Carangidae | *Selene brownii* | AF363750 | *CYB* |
| Perciformes | Percoidei | Carangidae | *Selene dorsalis* | AF363744 | *CYB* |
| Perciformes | Percoidei | Carangidae | *Selene dorsalis* | EU638006 | *rhod* |
| Perciformes | Percoidei | Carangidae | *Selene orstedii* | AF363740 | *CYB* |
| Perciformes | Percoidei | Carangidae | *Selene peruviana* | EU752204 | *CO1* |
| Perciformes | Percoidei | Carangidae | *Selene peruviana* | AF363743 | *CYB* |
| Perciformes | Percoidei | Carangidae | *Selene setapinnis* | GU225036 | *CO1* |
| Perciformes | Percoidei | Carangidae | *Selene setapinnis* | AF363745 | *CYB* |
| Perciformes | Percoidei | Carangidae | *Selene vomer* | FJ584093 | *CO1* |
| Perciformes | Percoidei | Carangidae | *Selene vomer* | AF363746 | *CYB* |
| Perciformes | Percoidei | Carangidae | *Seriola carpenteri* | EF392617 | *CYB* |
| Perciformes | Percoidei | Carangidae | *Seriola carpenteri* | EF427511 | *rhod* |
| Perciformes | Percoidei | Carangidae | *Seriola dumerili* | EF609458 | *CO1* |
| Perciformes | Percoidei | Carangidae | *Seriola dumerili* | EF439584 | *CYB* |
| Perciformes | Percoidei | Carangidae | *Seriola fasciata* | EF439233 | *CYB* |
| Perciformes | Percoidei | Carangidae | *Seriola fasciata* | EF439317 | *rhod* |
| Perciformes | Percoidei | Carangidae | *Seriola hippos* | EF609459 | *CO1* |
| Perciformes | Percoidei | Carangidae | *Seriola lalandi* | HM007728 | *CO1* |
| Perciformes | Percoidei | Carangidae | *Seriola lalandi* | AB264296 | *CYB* |
| Perciformes | Percoidei | Carangidae | *Seriola quinqueradiata* | HQ641666 | *CO1* |
| Perciformes | Percoidei | Carangidae | *Seriola quinqueradiata* | AB269355 | *CYB* |
| Perciformes | Percoidei | Carangidae | *Seriola rivoliana* | JN021317 | *CO1* |
| Perciformes | Percoidei | Carangidae | *Seriola rivoliana* | EF392621 | *CYB* |
| Perciformes | Percoidei | Carangidae | *Seriola rivoliana* | EF427515 | *rhod* |
| Perciformes | Percoidei | Carangidae | *Seriola zonata* | AY050744 | *CYB* |
| Perciformes | Percoidei | Carangidae | *Seriolina nigrofasciata* | EU014234 | *CO1* |
| Perciformes | Percoidei | Carangidae | *Seriolina nigrofasciata* | EF512298 | *CYB* |
| Perciformes | Percoidei | Carangidae | *Trachinotus anak* | EF609479 | *CO1* |
| Perciformes | Percoidei | Carangidae | *Trachinotus baillonii* | EF609480 | *CO1* |
| Perciformes | Percoidei | Carangidae | *Trachinotus blochii* | EU148560 | *CO1* |
| Perciformes | Percoidei | Carangidae | *Trachinotus blochii* | EF512289 | *CYB* |
| Perciformes | Percoidei | Carangidae | *Trachinotus carolinus* | AY050756 | *CYB* |
| Perciformes | Percoidei | Carangidae | *Trachinotus coppingeri* | EF609482 | *CO1* |
| Perciformes | Percoidei | Carangidae | *Trachinotus falcatus* | AY050738 | *CYB* |
| Perciformes | Percoidei | Carangidae | *Trachinotus goodei* | AY050741 | *CYB* |
| Perciformes | Percoidei | Carangidae | *Trachinotus goreensis* | HM883017 | *CO1* |
| Perciformes | Percoidei | Carangidae | *Trachinotus goreensis* | AY050746 | *CYB* |
| Perciformes | Percoidei | Carangidae | *Trachinotus ovatus* | FJ238043 | *CO1* |
| Perciformes | Percoidei | Carangidae | *Trachinotus ovatus* | AY050750 | *CYB* |
| Perciformes | Percoidei | Carangidae | *Trachinotus ovatus* | AY141314 | *rhod* |
| Perciformes | Percoidei | Carangidae | *Trachinotus paitensis* | HQ010062 | *CO1* |
| Perciformes | Percoidei | Carangidae | *Trachinotus rhodopus* | AY050739 | *CYB* |
| Perciformes | Percoidei | Carangidae | *Trachinotus teraia* | AY050745 | *CYB* |
| Perciformes | Percoidei | Carangidae | *Trachurus capensis* | HM007776 | *CO1* |
| Perciformes | Percoidei | Carangidae | *Trachurus capensis* | AY526536 | *CYB* |
| Perciformes | Percoidei | Carangidae | *Trachurus declivis* | EF609483 | *CO1* |
| Perciformes | Percoidei | Carangidae | *Trachurus declivis* | AY526542 | *CYB* |
| Perciformes | Percoidei | Carangidae | *Trachurus japonicus* | JF952880 | *CO1* |
| Perciformes | Percoidei | Carangidae | *Trachurus japonicus* | AB018994 | *CYB* |
| Perciformes | Percoidei | Carangidae | *Trachurus japonicus* | AB018979 | *ND2* |
| Perciformes | Percoidei | Carangidae | *Trachurus lathami* | AF363748 | *CYB* |
| Perciformes | Percoidei | Carangidae | *Trachurus mediterraneus* | FN689322 | *CO1* |
| Perciformes | Percoidei | Carangidae | *Trachurus mediterraneus* | AY526548 | *CYB* |
| Perciformes | Percoidei | Carangidae | *Trachurus mediterraneus* | EU224134 | *rhod* |
| Perciformes | Percoidei | Carangidae | *Trachurus murphyi* | EF609484 | *CO1* |
| Perciformes | Percoidei | Carangidae | *Trachurus murphyi* | AF531158 | *CYB* |
| Perciformes | Percoidei | Carangidae | *Trachurus novaezelandiae* | EF609485 | *CO1* |
| Perciformes | Percoidei | Carangidae | *Trachurus novaezelandiae* | AY526545 | *CYB* |
| Perciformes | Percoidei | Carangidae | *Trachurus picturatus* | EU148351 | *CO1* |
| Perciformes | Percoidei | Carangidae | *Trachurus picturatus* | EF439245 | *CYB* |
| Perciformes | Percoidei | Carangidae | *Trachurus picturatus* | HM050142 | *ND4* |
| Perciformes | Percoidei | Carangidae | *Trachurus picturatus* | EF439478 | *rhod* |
| Perciformes | Percoidei | Carangidae | *Trachurus symmetricus* | AY526541 | *CYB* |
| Perciformes | Percoidei | Carangidae | *Trachurus trachurus* | FN689334 | *CO1* |
| Perciformes | Percoidei | Carangidae | *Trachurus trachurus* | AY526534 | *CYB* |
| Perciformes | Percoidei | Carangidae | *Trachurus trachurus* | AB096009 | *ND1* |
| Perciformes | Percoidei | Carangidae | *Trachurus trachurus* | EU638013 | *rhod* |
| Perciformes | Percoidei | Carangidae | *Trachurus trecae* | AY050740 | *CYB* |
| Perciformes | Percoidei | Carangidae | *Uraspis helvola* | JF952885 | *CO1* |
| Perciformes | Percoidei | Carangidae | *Uraspis helvola* | AY050727 | *CYB* |
| Perciformes | Percoidei | Menidae | *Mene maculata* | FJ347937 | *CO1* |
| Perciformes | Percoidei | Menidae | *Mene maculata* | EF095659 | *rag1* |
| Perciformes | Percoidei | Menidae | *Mene maculata* | AY141316 | *rhod* |
| Perciformes | Percoidei | Leiognathidae | *Aurigequula fasciata* | HQ149860 | *CO1* |
| Perciformes | Percoidei | Leiognathidae | *Aurigequula fasciata* | EU637972 | *rhod* |
| Perciformes | Percoidei | Leiognathidae | *Equulites elongatus* | AY541627 | *CO1* |
| Perciformes | Percoidei | Leiognathidae | *Equulites elongatus* | AB100016 | *ND4* |
| Perciformes | Percoidei | Leiognathidae | *Equulites leuciscus* | EF607434 | *CO1* |
| Perciformes | Percoidei | Leiognathidae | *Equulites lineolatus* | FJ237600 | *CO1* |
| Perciformes | Percoidei | Leiognathidae | *Equulites rivulatus* | JF952779 | *CO1* |
| Perciformes | Percoidei | Leiognathidae | *Equulites rivulatus* | AB100019 | *ND4* |
| Perciformes | Percoidei | Leiognathidae | *Equulites stercorarius* | HQ993153 | *CO1* |
| Perciformes | Percoidei | Leiognathidae | *Equulites stercorarius* | AB100021 | *ND4* |
| Perciformes | Percoidei | Leiognathidae | *Equulites stercorarius* | EU167752 | *rag1* |
| Perciformes | Percoidei | Leiognathidae | *Eubleekeria jonesi* | HQ993144 | *CO1* |
| Perciformes | Percoidei | Leiognathidae | *Eubleekeria splendens* | HQ993145 | *CO1* |
| Perciformes | Percoidei | Leiognathidae | *Eubleekeria splendens* | AB100020 | *ND4* |
| Perciformes | Percoidei | Leiognathidae | *Gazza achlamys* | DQ028005 | *CO1* |
| Perciformes | Percoidei | Leiognathidae | *Gazza achlamys* | AB100025 | *ND4* |
| Perciformes | Percoidei | Leiognathidae | *Gazza dentex* | AB100026 | *ND4* |
| Perciformes | Percoidei | Leiognathidae | *Gazza minuta* | DQ648450 | *CO1* |
| Perciformes | Percoidei | Leiognathidae | *Gazza minuta* | AB100027 | *ND4* |
| Perciformes | Percoidei | Leiognathidae | *Gazza minuta* | EU167751 | *rag1* |
| Perciformes | Percoidei | Leiognathidae | *Gazza squamiventralis* | EU381034 | *CO1* |
| Perciformes | Percoidei | Leiognathidae | *Gazza squamiventralis* | EU380963 | *CYB* |
| Perciformes | Percoidei | Leiognathidae | *Gazza squamiventralis* | EU380992 | *ND1* |
| Perciformes | Percoidei | Leiognathidae | *Gazza squamiventralis* | EU381014 | *ND2* |
| Perciformes | Percoidei | Leiognathidae | *Leiognathus brevirostris* | EF607427 | *CO1* |
| Perciformes | Percoidei | Leiognathidae | *Leiognathus daura* | EU148522 | *CO1* |
| Perciformes | Percoidei | Leiognathidae | *Leiognathus dussumieri* | DQ028028 | *CO1* |
| Perciformes | Percoidei | Leiognathidae | *Leiognathus equulus* | EU381032 | *CO1* |
| Perciformes | Percoidei | Leiognathidae | *Leiognathus equulus* | EU380961 | *CYB* |
| Perciformes | Percoidei | Leiognathidae | *Leiognathus equulus* | EU380990 | *ND1* |
| Perciformes | Percoidei | Leiognathidae | *Leiognathus equulus* | AB100017 | *ND4* |
| Perciformes | Percoidei | Leiognathidae | *Leiognathus philippinus* | AY541635 | *CO1* |
| Perciformes | Percoidei | Leiognathidae | *Leiognathus robustus* | DQ028023 | *CO1* |
| Perciformes | Percoidei | Leiognathidae | *Nuchequula decora* | DQ028027 | *CO1* |
| Perciformes | Percoidei | Leiognathidae | *Nuchequula decora* | AB100015 | *ND4* |
| Perciformes | Percoidei | Leiognathidae | *Nuchequula mannusella* | HQ993147 | *CO1* |
| Perciformes | Percoidei | Leiognathidae | *Nuchequula nuchalis* | EF607435 | *CO1* |
| Perciformes | Percoidei | Leiognathidae | *Nuchequula nuchalis* | AB100028 | *ND4* |
| Perciformes | Percoidei | Leiognathidae | *Nuchequula pan* | AB100018 | *ND4* |
| Perciformes | Percoidei | Leiognathidae | *Photopectoralis aureus* | AY541625 | *CO1* |
| Perciformes | Percoidei | Leiognathidae | *Photopectoralis bindus* | EF609533 | *CO1* |
| Perciformes | Percoidei | Leiognathidae | *Photopectoralis panayensis* | AY541634 | *CO1* |
| Perciformes | Percoidei | Leiognathidae | *Secutor hanedai* | AB100022 | *ND4* |
| Perciformes | Percoidei | Leiognathidae | *Secutor indicius* | AY541640 | *CO1* |
| Perciformes | Percoidei | Leiognathidae | *Secutor indicius* | AB100023 | *ND4* |
| Perciformes | Percoidei | Leiognathidae | *Secutor insidiator* | EF607541 | *CO1* |
| Perciformes | Percoidei | Leiognathidae | *Secutor megalolepis* | DQ648454 | *CO1* |
| Perciformes | Percoidei | Leiognathidae | *Secutor megalolepis* | AB100024 | *ND4* |
| Perciformes | Percoidei | Leiognathidae | *Secutor ruconius* | EF609614 | *CO1* |
| Perciformes | Percoidei | Bramidae | *Brama australis* | FJ435626 | *CYB* |
| Perciformes | Percoidei | Bramidae | *Brama brama* | EF609300 | *CO1* |
| Perciformes | Percoidei | Bramidae | *Brama brama* | FJ435627 | *CYB* |
| Perciformes | Percoidei | Bramidae | *Brama brama* | EF439264 | *rhod* |
| Perciformes | Percoidei | Bramidae | *Brama japonica* | GU440256 | *CO1* |
| Perciformes | Percoidei | Bramidae | *Brama japonica* | AY973048 | *CYB* |
| Perciformes | Percoidei | Bramidae | *Brama orcini* | EU167812 | *rag1* |
| Perciformes | Percoidei | Bramidae | *Pterycombus brama* | GU225018 | *CO1* |
| Perciformes | Percoidei | Bramidae | *Pterycombus brama* | EU638001 | *rhod* |
| Perciformes | Percoidei | Bramidae | *Pterycombus petersii* | EF609445 | *CO1* |
| Perciformes | Percoidei | Bramidae | *Taractes asper* | GU440550 | *CO1* |
| Perciformes | Percoidei | Bramidae | *Taractes rubescens* | JF952875 | *CO1* |
| Perciformes | Percoidei | Bramidae | *Taractichthys longipinnis* | EF609476 | *CO1* |
| Perciformes | Percoidei | Bramidae | *Taractichthys longipinnis* | EF392626 | *CYB* |
| Perciformes | Percoidei | Bramidae | *Taractichthys longipinnis* | EF427520 | *rhod* |
| Perciformes | Percoidei | Bramidae | *Taractichthys steindachneri* | EF609477 | *CO1* |
| Perciformes | Percoidei | Bramidae | *Xenobrama microlepis* | EF609495 | *CO1* |
| Perciformes | Percoidei | Caristiidae | *Caristius macropus* | GU440263 | *CO1* |
| Perciformes | Percoidei | Caristiidae | *Paracaristius maderensis* | EU148108 | *CO1* |
| Perciformes | Percoidei | Emmelichthyidae | *Emmelichthys nitidus* | EF609348 | *CO1* |
| Perciformes | Percoidei | Emmelichthyidae | *Erythrocles monodi* | EF456004 | *CYB* |
| Perciformes | Percoidei | Emmelichthyidae | *Erythrocles monodi* | HM050104 | *ND4* |
| Perciformes | Percoidei | Emmelichthyidae | *Erythrocles monodi* | EU167831 | *rag1* |
| Perciformes | Percoidei | Emmelichthyidae | *Erythrocles monodi* | EF456054 | *rhod* |
| Perciformes | Percoidei | Emmelichthyidae | *Erythrocles schlegelii* | EU182623 | *rag1* |
| Perciformes | Percoidei | Emmelichthyidae | *Plagiogeneion macrolepis* | EF609434 | *CO1* |
| Perciformes | Percoidei | Lutjanidae | *Aphareus furca* | DQ784746 | *CYB* |
| Perciformes | Percoidei | Lutjanidae | *Aphareus furca* | HQ676633 | *rag1* |
| Perciformes | Percoidei | Lutjanidae | *Aphareus rutilans* | HQ561493 | *CO1* |
| Perciformes | Percoidei | Lutjanidae | *Aprion virescens* | DQ784747 | *CYB* |
| Perciformes | Percoidei | Lutjanidae | *Apsilus fuscus* | EU637941 | *rhod* |
| Perciformes | Percoidei | Lutjanidae | *Etelis carbunculus* | EF609353 | *CO1* |
| Perciformes | Percoidei | Lutjanidae | *Etelis oculatus* | GU225202 | *CO1* |
| Perciformes | Percoidei | Lutjanidae | *Lutjanus adetii* | EF609393 | *CO1* |
| Perciformes | Percoidei | Lutjanidae | *Lutjanus adetii* | DQ784751 | *CYB* |
| Perciformes | Percoidei | Lutjanidae | *Lutjanus analis* | FJ998455 | *CO1* |
| Perciformes | Percoidei | Lutjanidae | *Lutjanus analis* | EF095662 | *rag1* |
| Perciformes | Percoidei | Lutjanidae | *Lutjanus analis* | EF095620 | *rhod* |
| Perciformes | Percoidei | Lutjanidae | *Lutjanus apodus* | GU224896 | *CO1* |
| Perciformes | Percoidei | Lutjanidae | *Lutjanus argentimaculatus* | EU502686 | *CO1* |
| Perciformes | Percoidei | Lutjanidae | *Lutjanus argentimaculatus* | EF025494 | *CYB* |
| Perciformes | Percoidei | Lutjanidae | *Lutjanus argentimaculatus* | EU627659 | *rag1* |
| Perciformes | Percoidei | Lutjanidae | *Lutjanus argentimaculatus* | EU627658 | *rag2* |
| Perciformes | Percoidei | Lutjanidae | *Lutjanus argentiventris* | GU440385 | *CO1* |
| Perciformes | Percoidei | Lutjanidae | *Lutjanus bengalensis* | EU600135 | *CO1* |
| Perciformes | Percoidei | Lutjanidae | *Lutjanus bengalensis* | EU627660 | *rag1* |
| Perciformes | Percoidei | Lutjanidae | *Lutjanus bengalensis* | EU627673 | *rag2* |
| Perciformes | Percoidei | Lutjanidae | *Lutjanus bohar* | EU600131 | *CO1* |
| Perciformes | Percoidei | Lutjanidae | *Lutjanus bohar* | DQ784753 | *CYB* |
| Perciformes | Percoidei | Lutjanidae | *Lutjanus buccanella* | FJ998465 | *CO1* |
| Perciformes | Percoidei | Lutjanidae | *Lutjanus campechanus* | JN021303 | *CO1* |
| Perciformes | Percoidei | Lutjanidae | *Lutjanus campechanus* | AY374294 | *CYB* |
| Perciformes | Percoidei | Lutjanidae | *Lutjanus campechanus* | AY600332 | *ND4* |
| Perciformes | Percoidei | Lutjanidae | *Lutjanus carponotatus* | EF609395 | *CO1* |
| Perciformes | Percoidei | Lutjanidae | *Lutjanus carponotatus* | DQ784754 | *CYB* |
| Perciformes | Percoidei | Lutjanidae | *Lutjanus colorado* | GU186970 | *CO1* |
| Perciformes | Percoidei | Lutjanidae | *Lutjanus cyanopterus* | GQ329867 | *CO1* |
| Perciformes | Percoidei | Lutjanidae | *Lutjanus decussatus* | AF240750 | *CYB* |
| Perciformes | Percoidei | Lutjanidae | *Lutjanus dentatus* | EU167846 | *rag1* |
| Perciformes | Percoidei | Lutjanidae | *Lutjanus ehrenbergii* | HQ149873 | *CO1* |
| Perciformes | Percoidei | Lutjanidae | *Lutjanus erythropterus* | EU502676 | *CO1* |
| Perciformes | Percoidei | Lutjanidae | *Lutjanus erythropterus* | DQ900664 | *CYB* |
| Perciformes | Percoidei | Lutjanidae | *Lutjanus erythropterus* | EU627661 | *rag1* |
| Perciformes | Percoidei | Lutjanidae | *Lutjanus erythropterus* | EU627653 | *rag2* |
| Perciformes | Percoidei | Lutjanidae | *Lutjanus fulviflamma* | EU502683 | *CO1* |
| Perciformes | Percoidei | Lutjanidae | *Lutjanus fulviflamma* | EF376177 | *CYB* |
| Perciformes | Percoidei | Lutjanidae | *Lutjanus fulviflamma* | EU627662 | *rag1* |
| Perciformes | Percoidei | Lutjanidae | *Lutjanus fulviflamma* | EU627654 | *rag2* |
| Perciformes | Percoidei | Lutjanidae | *Lutjanus fulvus* | EU502667 | *CO1* |
| Perciformes | Percoidei | Lutjanidae | *Lutjanus fulvus* | DQ900679 | *CYB* |
| Perciformes | Percoidei | Lutjanidae | *Lutjanus fulvus* | EU627672 | *rag1* |
| Perciformes | Percoidei | Lutjanidae | *Lutjanus fulvus* | EU627675 | *rag2* |
| Perciformes | Percoidei | Lutjanidae | *Lutjanus gibbus* | GU805121 | *CO1* |
| Perciformes | Percoidei | Lutjanidae | *Lutjanus gibbus* | DQ784757 | *CYB* |
| Perciformes | Percoidei | Lutjanidae | *Lutjanus griseus* | JN021304 | *CO1* |
| Perciformes | Percoidei | Lutjanidae | *Lutjanus griseus* | EU669443 | *ND4* |
| Perciformes | Percoidei | Lutjanidae | *Lutjanus jocu* | FJ998474 | *CO1* |
| Perciformes | Percoidei | Lutjanidae | *Lutjanus johnii* | EU502681 | *CO1* |
| Perciformes | Percoidei | Lutjanidae | *Lutjanus johnii* | DQ900682 | *CYB* |
| Perciformes | Percoidei | Lutjanidae | *Lutjanus johnii* | EU627663 | *rag1* |
| Perciformes | Percoidei | Lutjanidae | *Lutjanus johnii* | EU627678 | *rag2* |
| Perciformes | Percoidei | Lutjanidae | *Lutjanus kasmira* | EU600138 | *CO1* |
| Perciformes | Percoidei | Lutjanidae | *Lutjanus kasmira* | DQ784758 | *CYB* |
| Perciformes | Percoidei | Lutjanidae | *Lutjanus kasmira* | EU627664 | *rag1* |
| Perciformes | Percoidei | Lutjanidae | *Lutjanus kasmira* | EU627652 | *rag2* |
| Perciformes | Percoidei | Lutjanidae | *Lutjanus lemniscatus* | EF609397 | *CO1* |
| Perciformes | Percoidei | Lutjanidae | *Lutjanus lineatus* | EU600134 | *CO1* |
| Perciformes | Percoidei | Lutjanidae | *Lutjanus lutjanus* | EU148543 | *CO1* |
| Perciformes | Percoidei | Lutjanidae | *Lutjanus lutjanus* | EU627665 | *rag1* |
| Perciformes | Percoidei | Lutjanidae | *Lutjanus lutjanus* | EU627677 | *rag2* |
| Perciformes | Percoidei | Lutjanidae | *Lutjanus mahogoni* | FJ998477 | *CO1* |
| Perciformes | Percoidei | Lutjanidae | *Lutjanus mahogoni* | EU182625 | *rag1* |
| Perciformes | Percoidei | Lutjanidae | *Lutjanus malabaricus* | EU502678 | *CO1* |
| Perciformes | Percoidei | Lutjanidae | *Lutjanus malabaricus* | DQ900668 | *CYB* |
| Perciformes | Percoidei | Lutjanidae | *Lutjanus malabaricus* | EU627666 | *rag1* |
| Perciformes | Percoidei | Lutjanidae | *Lutjanus malabaricus* | EU627655 | *rag2* |
| Perciformes | Percoidei | Lutjanidae | *Lutjanus monostigma* | JF952788 | *CO1* |
| Perciformes | Percoidei | Lutjanidae | *Lutjanus monostigma* | DQ784759 | *CYB* |
| Perciformes | Percoidei | Lutjanidae | *Lutjanus notatus* | HQ561527 | *CO1* |
| Perciformes | Percoidei | Lutjanidae | *Lutjanus novemfasciatus* | GU440386 | *CO1* |
| Perciformes | Percoidei | Lutjanidae | *Lutjanus ophuysenii* | EU502670 | *CO1* |
| Perciformes | Percoidei | Lutjanidae | *Lutjanus ophuysenii* | EF376180 | *CYB* |
| Perciformes | Percoidei | Lutjanidae | *Lutjanus ophuysenii* | EU627671 | *rag1* |
| Perciformes | Percoidei | Lutjanidae | *Lutjanus ophuysenii* | EU627674 | *rag2* |
| Perciformes | Percoidei | Lutjanidae | *Lutjanus peru* | EU752116 | *CO1* |
| Perciformes | Percoidei | Lutjanidae | *Lutjanus peru* | AY600367 | *ND4* |
| Perciformes | Percoidei | Lutjanidae | *Lutjanus purpureus* | JN021305 | *CO1* |
| Perciformes | Percoidei | Lutjanidae | *Lutjanus quinquelineatus* | EF609399 | *CO1* |
| Perciformes | Percoidei | Lutjanidae | *Lutjanus quinquelineatus* | DQ784760 | *CYB* |
| Perciformes | Percoidei | Lutjanidae | *Lutjanus rivulatus* | DQ885109 | *CO1* |
| Perciformes | Percoidei | Lutjanidae | *Lutjanus rivulatus* | DQ484045 | *CYB* |
| Perciformes | Percoidei | Lutjanidae | *Lutjanus russellii* | EU502680 | *CO1* |
| Perciformes | Percoidei | Lutjanidae | *Lutjanus russellii* | EF376160 | *CYB* |
| Perciformes | Percoidei | Lutjanidae | *Lutjanus russellii* | EU627667 | *rag1* |
| Perciformes | Percoidei | Lutjanidae | *Lutjanus russellii* | EU627676 | *rag2* |
| Perciformes | Percoidei | Lutjanidae | *Lutjanus sanguineus* | AY590146 | *CYB* |
| Perciformes | Percoidei | Lutjanidae | *Lutjanus sebae* | EU502673 | *CO1* |
| Perciformes | Percoidei | Lutjanidae | *Lutjanus sebae* | EF376170 | *CYB* |
| Perciformes | Percoidei | Lutjanidae | *Lutjanus sebae* | EU627668 | *rag1* |
| Perciformes | Percoidei | Lutjanidae | *Lutjanus sebae* | EU627656 | *rag2* |
| Perciformes | Percoidei | Lutjanidae | *Lutjanus sebae* | EU637974 | *rhod* |
| Perciformes | Percoidei | Lutjanidae | *Lutjanus stellatus* | EU502688 | *CO1* |
| Perciformes | Percoidei | Lutjanidae | *Lutjanus stellatus* | EF376163 | *CYB* |
| Perciformes | Percoidei | Lutjanidae | *Lutjanus stellatus* | EU627670 | *rag1* |
| Perciformes | Percoidei | Lutjanidae | *Lutjanus stellatus* | EU627657 | *rag2* |
| Perciformes | Percoidei | Lutjanidae | *Lutjanus synagris* | JN021307 | *CO1* |
| Perciformes | Percoidei | Lutjanidae | *Lutjanus synagris* | EU676015 | *ND4* |
| Perciformes | Percoidei | Lutjanidae | *Lutjanus vitta* | EU502672 | *CO1* |
| Perciformes | Percoidei | Lutjanidae | *Lutjanus vitta* | DQ900676 | *CYB* |
| Perciformes | Percoidei | Lutjanidae | *Lutjanus vitta* | EU627669 | *rag1* |
| Perciformes | Percoidei | Lutjanidae | *Lutjanus vitta* | EU556712 | *rag2* |
| Perciformes | Percoidei | Lutjanidae | *Lutjanus vivanus* | FJ998486 | *CO1* |
| Perciformes | Percoidei | Lutjanidae | *Macolor niger* | EF609403 | *CO1* |
| Perciformes | Percoidei | Lutjanidae | *Macolor niger* | DQ784764 | *CYB* |
| Perciformes | Percoidei | Lutjanidae | *Ocyurus chrysurus* | JN021311 | *CO1* |
| Perciformes | Percoidei | Lutjanidae | *Ocyurus chrysurus* | JF428896 | *ND4* |
| Perciformes | Percoidei | Lutjanidae | *Paracaesio sordida* | GU805104 | *CO1* |
| Perciformes | Percoidei | Lutjanidae | *Pinjalo pinjalo* | EU167860 | *rag1* |
| Perciformes | Percoidei | Lutjanidae | *Pristipomoides aquilonaris* | FJ998490 | *CO1* |
| Perciformes | Percoidei | Lutjanidae | *Pristipomoides filamentosus* | DQ885120 | *CO1* |
| Perciformes | Percoidei | Lutjanidae | *Pristipomoides multidens* | FJ237568 | *CO1* |
| Perciformes | Percoidei | Lutjanidae | *Pristipomoides sieboldii* | GU805055 | *CO1* |
| Perciformes | Percoidei | Lutjanidae | *Pristipomoides typus* | EF609438 | *CO1* |
| Perciformes | Percoidei | Lutjanidae | *Rhomboplites aurorubens* | JN021316 | *CO1* |
| Perciformes | Percoidei | Lutjanidae | *Rhomboplites aurorubens* | AY294200 | *CYB* |
| Perciformes | Percoidei | Lutjanidae | *Symphorichthys spilurus* | FJ584135 | *CO1* |
| Perciformes | Percoidei | Lutjanidae | *Symphorichthys spilurus* | DQ784768 | *CYB* |
| Perciformes | Percoidei | Lutjanidae | *Symphorus nematophorus* | DQ784769 | *CYB* |
| Perciformes | Percoidei | Lutjanidae | *Symphorus nematophorus* | EU167876 | *rag1* |
| Perciformes | Percoidei | Caesionidae | *Caesio caerulaurea* | FJ237605 | *CO1* |
| Perciformes | Percoidei | Caesionidae | *Caesio caerulaurea* | AF381273 | *CYB* |
| Perciformes | Percoidei | Caesionidae | *Caesio cuning* | AF240749 | *CYB* |
| Perciformes | Percoidei | Caesionidae | *Caesio lunaris* | EU167813 | *rag1* |
| Perciformes | Percoidei | Caesionidae | *Caesio xanthonota* | HQ561536 | *CO1* |
| Perciformes | Percoidei | Caesionidae | *Dipterygonotus balteatus* | AF381270 | *CYB* |
| Perciformes | Percoidei | Caesionidae | *Gymnocaesio gymnoptera* | HQ561486 | *CO1* |
| Perciformes | Percoidei | Caesionidae | *Gymnocaesio gymnoptera* | EU167837 | *rag1* |
| Perciformes | Percoidei | Caesionidae | *Pterocaesio chrysozona* | HQ561529 | *CO1* |
| Perciformes | Percoidei | Caesionidae | *Pterocaesio digramma* | JF952832 | *CO1* |
| Perciformes | Percoidei | Caesionidae | *Pterocaesio digramma* | EU638000 | *rhod* |
| Perciformes | Percoidei | Caesionidae | *Pterocaesio marri* | HQ561455 | *CO1* |
| Perciformes | Percoidei | Caesionidae | *Pterocaesio marri* | DQ784766 | *CYB* |
| Perciformes | Percoidei | Caesionidae | *Pterocaesio pisang* | DQ784767 | *CYB* |
| Perciformes | Percoidei | Caesionidae | *Pterocaesio trilineata* | HQ561504 | *CO1* |
| Perciformes | Percoidei | Lobotidae | *Datnioides polota* | EU637954 | *rhod* |
| Perciformes | Percoidei | Lobotidae | *Lobotes pacificus* | GU440380 | *CO1* |
| Perciformes | Percoidei | Lobotidae | *Lobotes pacificus* | HQ676659 | *rag1* |
| Perciformes | Percoidei | Lobotidae | *Lobotes surinamensis* | FJ237805 | *CO1* |
| Perciformes | Percoidei | Lobotidae | *Lobotes surinamensis* | HQ676660 | *rag1* |
| Perciformes | Percoidei | Gerreidae | *Diapterus auratus* | DQ027996 | *CO1* |
| Perciformes | Percoidei | Gerreidae | *Diapterus auratus* | EF095664 | *rag1* |
| Perciformes | Percoidei | Gerreidae | *Diapterus auratus* | EF095622 | *rhod* |
| Perciformes | Percoidei | Gerreidae | *Diapterus peruvianus* | EU167825 | *rag1* |
| Perciformes | Percoidei | Gerreidae | *Eucinostomus argenteus* | GU225218 | *CO1* |
| Perciformes | Percoidei | Gerreidae | *Eucinostomus currani* | HQ010063 | *CO1* |
| Perciformes | Percoidei | Gerreidae | *Eucinostomus gula* | GU224845 | *CO1* |
| Perciformes | Percoidei | Gerreidae | *Eucinostomus gula* | EF095663 | *rag1* |
| Perciformes | Percoidei | Gerreidae | *Eucinostomus gula* | EF095621 | *rhod* |
| Perciformes | Percoidei | Gerreidae | *Eucinostomus havana* | GU225237 | *CO1* |
| Perciformes | Percoidei | Gerreidae | *Eucinostomus jonesii* | GU224431 | *CO1* |
| Perciformes | Percoidei | Gerreidae | *Eucinostomus melanopterus* | GU224854 | *CO1* |
| Perciformes | Percoidei | Gerreidae | *Eugerres plumieri* | GU225258 | *CO1* |
| Perciformes | Percoidei | Gerreidae | *Eugerres plumieri* | EF095665 | *rag1* |
| Perciformes | Percoidei | Gerreidae | *Eugerres plumieri* | EF095623 | *rhod* |
| Perciformes | Percoidei | Gerreidae | *Gerres cinereus* | GU225290 | *CO1* |
| Perciformes | Percoidei | Gerreidae | *Gerres cinereus* | EF095666 | *rag1* |
| Perciformes | Percoidei | Gerreidae | *Gerres cinereus* | EF095624 | *rhod* |
| Perciformes | Percoidei | Gerreidae | *Gerres equulus* | AY541643 | *CO1* |
| Perciformes | Percoidei | Gerreidae | *Gerres erythrourus* | AY541642 | *CO1* |
| Perciformes | Percoidei | Gerreidae | *Gerres filamentosus* | EU871690 | *CO1* |
| Perciformes | Percoidei | Gerreidae | *Gerres japonicus* | JF952741 | *CO1* |
| Perciformes | Percoidei | Gerreidae | *Gerres limbatus* | EF607391 | *CO1* |
| Perciformes | Percoidei | Gerreidae | *Gerres oblongus* | EF607393 | *CO1* |
| Perciformes | Percoidei | Gerreidae | *Gerres oyena* | EU167833 | *rag1* |
| Perciformes | Percoidei | Gerreidae | *Pentaprion longimanus* | EU392181 | *CO1* |
| Perciformes | Percoidei | Haemulidae | *Anisotremus caesius* | EU694308 | *CYB* |
| Perciformes | Percoidei | Haemulidae | *Anisotremus davidsonii* | GU440220 | *CO1* |
| Perciformes | Percoidei | Haemulidae | *Anisotremus davidsonii* | EU694383 | *CYB* |
| Perciformes | Percoidei | Haemulidae | *Anisotremus davidsonii* | HQ676626 | *rag1* |
| Perciformes | Percoidei | Haemulidae | *Anisotremus dovii* | HQ684719 | *CO1* |
| Perciformes | Percoidei | Haemulidae | *Anisotremus dovii* | EU694297 | *CYB* |
| Perciformes | Percoidei | Haemulidae | *Anisotremus dovii* | HQ676627 | *rag1* |
| Perciformes | Percoidei | Haemulidae | *Anisotremus interruptus* | EU697525 | *CO1* |
| Perciformes | Percoidei | Haemulidae | *Anisotremus interruptus* | EU697499 | *CYB* |
| Perciformes | Percoidei | Haemulidae | *Anisotremus interruptus* | HQ676628 | *rag1* |
| Perciformes | Percoidei | Haemulidae | *Anisotremus moricandi* | EU694318 | *CYB* |
| Perciformes | Percoidei | Haemulidae | *Anisotremus pacifici* | EU694315 | *CYB* |
| Perciformes | Percoidei | Haemulidae | *Anisotremus pacifici* | HQ676629 | *rag1* |
| Perciformes | Percoidei | Haemulidae | *Anisotremus scapularis* | EU694322 | *CYB* |
| Perciformes | Percoidei | Haemulidae | *Anisotremus scapularis* | HQ676630 | *rag1* |
| Perciformes | Percoidei | Haemulidae | *Anisotremus surinamensis* | EU697526 | *CO1* |
| Perciformes | Percoidei | Haemulidae | *Anisotremus surinamensis* | EU697500 | *CYB* |
| Perciformes | Percoidei | Haemulidae | *Anisotremus surinamensis* | HQ676631 | *rag1* |
| Perciformes | Percoidei | Haemulidae | *Anisotremus taeniatus* | EU697527 | *CO1* |
| Perciformes | Percoidei | Haemulidae | *Anisotremus taeniatus* | EU697501 | *CYB* |
| Perciformes | Percoidei | Haemulidae | *Anisotremus taeniatus* | HQ676632 | *rag1* |
| Perciformes | Percoidei | Haemulidae | *Anisotremus virginicus* | EU697524 | *CO1* |
| Perciformes | Percoidei | Haemulidae | *Anisotremus virginicus* | EU694344 | *CYB* |
| Perciformes | Percoidei | Haemulidae | *Anisotremus virginicus* | EU167810 | *rag1* |
| Perciformes | Percoidei | Haemulidae | *Boridia grossidens* | HQ676634 | *rag1* |
| Perciformes | Percoidei | Haemulidae | *Brachydeuterus auritus* | EU167811 | *rag1* |
| Perciformes | Percoidei | Haemulidae | *Conodon nobilis* | GU224768 | *CO1* |
| Perciformes | Percoidei | Haemulidae | *Conodon nobilis* | HQ676635 | *rag1* |
| Perciformes | Percoidei | Haemulidae | *Conodon serrifer* | HQ676636 | *rag1* |
| Perciformes | Percoidei | Haemulidae | *Diagramma labiosum* | DQ784750 | *CYB* |
| Perciformes | Percoidei | Haemulidae | *Diagramma picta* | GU207326 | *CO1* |
| Perciformes | Percoidei | Haemulidae | *Diagramma picta* | HQ676637 | *rag1* |
| Perciformes | Percoidei | Haemulidae | *Diagramma picta* | AY279872 | *rag2* |
| Perciformes | Percoidei | Haemulidae | *Genyatremus luteus* | EU694295 | *CYB* |
| Perciformes | Percoidei | Haemulidae | *Genyatremus luteus* | HQ676639 | *rag1* |
| Perciformes | Percoidei | Haemulidae | *Haemulon album* | EU697523 | *CO1* |
| Perciformes | Percoidei | Haemulidae | *Haemulon album* | EU697498 | *CYB* |
| Perciformes | Percoidei | Haemulidae | *Haemulon aurolineatum* | EU697528 | *CO1* |
| Perciformes | Percoidei | Haemulidae | *Haemulon aurolineatum* | EU697502 | *CYB* |
| Perciformes | Percoidei | Haemulidae | *Haemulon aurolineatum* | EF095661 | *rag1* |
| Perciformes | Percoidei | Haemulidae | *Haemulon aurolineatum* | EF095619 | *rhod* |
| Perciformes | Percoidei | Haemulidae | *Haemulon bonariense* | EU697529 | *CO1* |
| Perciformes | Percoidei | Haemulidae | *Haemulon bonariense* | EU697522 | *CYB* |
| Perciformes | Percoidei | Haemulidae | *Haemulon boschmae* | EU697530 | *CO1* |
| Perciformes | Percoidei | Haemulidae | *Haemulon boschmae* | EU697503 | *CYB* |
| Perciformes | Percoidei | Haemulidae | *Haemulon carbonarium* | GU225296 | *CO1* |
| Perciformes | Percoidei | Haemulidae | *Haemulon carbonarium* | EU697504 | *CYB* |
| Perciformes | Percoidei | Haemulidae | *Haemulon chrysargyreum* | EU697532 | *CO1* |
| Perciformes | Percoidei | Haemulidae | *Haemulon chrysargyreum* | EU697505 | *CYB* |
| Perciformes | Percoidei | Haemulidae | *Haemulon chrysargyreum* | HQ676641 | *rag1* |
| Perciformes | Percoidei | Haemulidae | *Haemulon flaviguttatum* | EU697533 | *CO1* |
| Perciformes | Percoidei | Haemulidae | *Haemulon flaviguttatum* | EU697506 | *CYB* |
| Perciformes | Percoidei | Haemulidae | *Haemulon flaviguttatum* | HQ676642 | *rag1* |
| Perciformes | Percoidei | Haemulidae | *Haemulon flavolineatum* | GU225299 | *CO1* |
| Perciformes | Percoidei | Haemulidae | *Haemulon flavolineatum* | EU697507 | *CYB* |
| Perciformes | Percoidei | Haemulidae | *Haemulon flavolineatum* | HQ676643 | *rag1* |
| Perciformes | Percoidei | Haemulidae | *Haemulon macrostomum* | GU225307 | *CO1* |
| Perciformes | Percoidei | Haemulidae | *Haemulon macrostomum* | EU697509 | *CYB* |
| Perciformes | Percoidei | Haemulidae | *Haemulon macrostomum* | HQ676644 | *rag1* |
| Perciformes | Percoidei | Haemulidae | *Haemulon maculicauda* | EU697537 | *CO1* |
| Perciformes | Percoidei | Haemulidae | *Haemulon maculicauda* | EU697510 | *CYB* |
| Perciformes | Percoidei | Haemulidae | *Haemulon melanurum* | EU697538 | *CO1* |
| Perciformes | Percoidei | Haemulidae | *Haemulon melanurum* | EU697511 | *CYB* |
| Perciformes | Percoidei | Haemulidae | *Haemulon melanurum* | HQ676645 | *rag1* |
| Perciformes | Percoidei | Haemulidae | *Haemulon parra* | EU697539 | *CO1* |
| Perciformes | Percoidei | Haemulidae | *Haemulon parra* | EU697512 | *CYB* |
| Perciformes | Percoidei | Haemulidae | *Haemulon plumierii* | GU224487 | *CO1* |
| Perciformes | Percoidei | Haemulidae | *Haemulon plumierii* | EU697513 | *CYB* |
| Perciformes | Percoidei | Haemulidae | *Haemulon plumierii* | HQ676646 | *rag1* |
| Perciformes | Percoidei | Haemulidae | *Haemulon sciurus* | GU225318 | *CO1* |
| Perciformes | Percoidei | Haemulidae | *Haemulon sciurus* | AF240747 | *CYB* |
| Perciformes | Percoidei | Haemulidae | *Haemulon scudderii* | EU697542 | *CO1* |
| Perciformes | Percoidei | Haemulidae | *Haemulon scudderii* | EU697515 | *CYB* |
| Perciformes | Percoidei | Haemulidae | *Haemulon scudderii* | HQ676648 | *rag1* |
| Perciformes | Percoidei | Haemulidae | *Haemulon sexfasciatum* | EU697543 | *CO1* |
| Perciformes | Percoidei | Haemulidae | *Haemulon sexfasciatum* | EU697516 | *CYB* |
| Perciformes | Percoidei | Haemulidae | *Haemulon squamipinna* | EU697544 | *CO1* |
| Perciformes | Percoidei | Haemulidae | *Haemulon squamipinna* | EU697517 | *CYB* |
| Perciformes | Percoidei | Haemulidae | *Haemulon steindachneri* | EU697547 | *CO1* |
| Perciformes | Percoidei | Haemulidae | *Haemulon steindachneri* | EU697518 | *CYB* |
| Perciformes | Percoidei | Haemulidae | *Haemulon steindachneri* | HQ676649 | *rag1* |
| Perciformes | Percoidei | Haemulidae | *Haemulon striatum* | EU697548 | *CO1* |
| Perciformes | Percoidei | Haemulidae | *Haemulon striatum* | EU697521 | *CYB* |
| Perciformes | Percoidei | Haemulidae | *Haemulopsis axillaris* | HQ676650 | *rag1* |
| Perciformes | Percoidei | Haemulidae | *Haemulopsis leuciscus* | HQ676651 | *rag1* |
| Perciformes | Percoidei | Haemulidae | *Haemulopsis nitidus* | HQ676652 | *rag1* |
| Perciformes | Percoidei | Haemulidae | *Hapalogenys analis* | GU120182 | *CYB* |
| Perciformes | Percoidei | Haemulidae | *Hapalogenys kishinouyei* | HQ676654 | *rag1* |
| Perciformes | Percoidei | Haemulidae | *Hapalogenys nigripinnis* | GU120183 | *CYB* |
| Perciformes | Percoidei | Haemulidae | *Hapalogenys nigripinnis* | HQ676655 | *rag1* |
| Perciformes | Percoidei | Haemulidae | *Isacia conceptionis* | HQ676657 | *rag1* |
| Perciformes | Percoidei | Haemulidae | *Microlepidotus brevipinnis* | HQ676662 | *rag1* |
| Perciformes | Percoidei | Haemulidae | *Orthopristis chalceus* | HQ676664 | *rag1* |
| Perciformes | Percoidei | Haemulidae | *Orthopristis chrysoptera* | GU225651 | *CO1* |
| Perciformes | Percoidei | Haemulidae | *Orthopristis chrysoptera* | HQ676665 | *rag1* |
| Perciformes | Percoidei | Haemulidae | *Parapristipoma octolineatum* | HQ676666 | *rag1* |
| Perciformes | Percoidei | Haemulidae | *Parapristipoma octolineatum* | DQ197879 | *rhod* |
| Perciformes | Percoidei | Haemulidae | *Parapristipoma trilineatum* | EF607481 | *CO1* |
| Perciformes | Percoidei | Haemulidae | *Parapristipoma trilineatum* | AB372026 | *CYB* |
| Perciformes | Percoidei | Haemulidae | *Parapristipoma trilineatum* | HQ676667 | *rag1* |
| Perciformes | Percoidei | Haemulidae | *Plectorhinchus chaetodonoides* | FJ583863 | *CO1* |
| Perciformes | Percoidei | Haemulidae | *Plectorhinchus chaetodonoides* | EU694282 | *CYB* |
| Perciformes | Percoidei | Haemulidae | *Plectorhinchus chaetodonoides* | HQ676668 | *rag1* |
| Perciformes | Percoidei | Haemulidae | *Plectorhinchus chrysotaenia* | DQ784765 | *CYB* |
| Perciformes | Percoidei | Haemulidae | *Plectorhinchus cinctus* | EU595230 | *CO1* |
| Perciformes | Percoidei | Haemulidae | *Plectorhinchus cinctus* | HQ676669 | *rag1* |
| Perciformes | Percoidei | Haemulidae | *Plectorhinchus diagrammus* | HQ676670 | *rag1* |
| Perciformes | Percoidei | Haemulidae | *Plectorhinchus flavomaculatus* | HQ561522 | *CO1* |
| Perciformes | Percoidei | Haemulidae | *Plectorhinchus gaterinus* | HQ561465 | *CO1* |
| Perciformes | Percoidei | Haemulidae | *Plectorhinchus gibbosus* | HQ561466 | *CO1* |
| Perciformes | Percoidei | Haemulidae | *Plectorhinchus gibbosus* | HQ676671 | *rag1* |
| Perciformes | Percoidei | Haemulidae | *Plectorhinchus lessonii* | HQ676672 | *rag1* |
| Perciformes | Percoidei | Haemulidae | *Plectorhinchus macrolepis* | EU167861 | *rag1* |
| Perciformes | Percoidei | Haemulidae | *Plectorhinchus mediterraneus* | HM050130 | *ND4* |
| Perciformes | Percoidei | Haemulidae | *Plectorhinchus mediterraneus* | DQ197881 | *rhod* |
| Perciformes | Percoidei | Haemulidae | *Plectorhinchus orientalis* | FJ237593 | *CO1* |
| Perciformes | Percoidei | Haemulidae | *Plectorhinchus orientalis* | HQ676673 | *rag1* |
| Perciformes | Percoidei | Haemulidae | *Plectorhinchus picus* | FJ583866 | *CO1* |
| Perciformes | Percoidei | Haemulidae | *Plectorhinchus schotaf* | HQ149903 | *CO1* |
| Perciformes | Percoidei | Haemulidae | *Plectorhinchus schotaf* | HQ676674 | *rag1* |
| Perciformes | Percoidei | Haemulidae | *Plectorhinchus sordidus* | HQ676675 | *rag1* |
| Perciformes | Percoidei | Haemulidae | *Pomadasys argyreus* | HQ676677 | *rag1* |
| Perciformes | Percoidei | Haemulidae | *Pomadasys branickii* | HQ676678 | *rag1* |
| Perciformes | Percoidei | Haemulidae | *Pomadasys hasta* | EF607491 | *CO1* |
| Perciformes | Percoidei | Haemulidae | *Pomadasys incisus* | EF439222 | *CYB* |
| Perciformes | Percoidei | Haemulidae | *Pomadasys incisus* | HQ676679 | *rag1* |
| Perciformes | Percoidei | Haemulidae | *Pomadasys incisus* | EF439299 | *rhod* |
| Perciformes | Percoidei | Haemulidae | *Pomadasys kaakan* | HQ676680 | *rag1* |
| Perciformes | Percoidei | Haemulidae | *Pomadasys maculatus* | FJ237889 | *CO1* |
| Perciformes | Percoidei | Haemulidae | *Pomadasys maculatus* | EF512297 | *CYB* |
| Perciformes | Percoidei | Haemulidae | *Pomadasys maculatus* | HQ676681 | *rag1* |
| Perciformes | Percoidei | Haemulidae | *Pomadasys olivaceus* | EU182626 | *rag1* |
| Perciformes | Percoidei | Haemulidae | *Pomadasys panamensis* | HQ676682 | *rag1* |
| Perciformes | Percoidei | Haemulidae | *Pomadasys perotaei* | EF456016 | *CYB* |
| Perciformes | Percoidei | Haemulidae | *Pomadasys perotaei* | HQ676683 | *rag1* |
| Perciformes | Percoidei | Haemulidae | *Pomadasys perotaei* | DQ021404 | *rhod* |
| Perciformes | Percoidei | Haemulidae | *Pomadasys striatus* | HQ676684 | *rag1* |
| Perciformes | Percoidei | Haemulidae | *Pomadasys stridens* | HQ149908 | *CO1* |
| Perciformes | Percoidei | Haemulidae | *Pomadasys stridens* | HQ676685 | *rag1* |
| Perciformes | Percoidei | Haemulidae | *Xenichthys xanti* | HQ676687 | *rag1* |
| Perciformes | Percoidei | Haemulidae | *Xenistius californiensis* | GU440565 | *CO1* |
| Perciformes | Percoidei | Haemulidae | *Xenistius californiensis* | HQ676688 | *rag1* |
| Perciformes | Percoidei | Inermidae | *Emmelichthyops atlanticus* | HQ676638 | *rag1* |
| Perciformes | Percoidei | Inermiidae | *Inermia vittata* | EU697535 | *CO1* |
| Perciformes | Percoidei | Inermiidae | *Inermia vittata* | EU697508 | *CYB* |
| Perciformes | Percoidei | Inermiidae | *Inermia vittata* | EU167905 | *rag1* |
| Perciformes | Percoidei | Nemipteridae | *Nemipterus bathybius* | AB326974 | *CYB* |
| Perciformes | Percoidei | Nemipteridae | *Nemipterus bipunctatus* | HQ423413 | *CO1* |
| Perciformes | Percoidei | Nemipteridae | *Nemipterus furcosus* | EF609413 | *CO1* |
| Perciformes | Percoidei | Nemipteridae | *Nemipterus hexodon* | EF609414 | *CO1* |
| Perciformes | Percoidei | Nemipteridae | *Nemipterus japonicus* | EU871687 | *CO1* |
| Perciformes | Percoidei | Nemipteridae | *Nemipterus japonicus* | AB264298 | *CYB* |
| Perciformes | Percoidei | Nemipteridae | *Nemipterus japonicus* | EU167784 | *rag1* |
| Perciformes | Percoidei | Nemipteridae | *Nemipterus marginatus* | AF240754 | *CYB* |
| Perciformes | Percoidei | Nemipteridae | *Nemipterus marginatus* | EU167785 | *rag1* |
| Perciformes | Percoidei | Nemipteridae | *Nemipterus mesoprion* | EF609560 | *CO1* |
| Perciformes | Percoidei | Nemipteridae | *Nemipterus peronii* | EF609415 | *CO1* |
| Perciformes | Percoidei | Nemipteridae | *Nemipterus virgatus* | FJ237838 | *CO1* |
| Perciformes | Percoidei | Nemipteridae | *Nemipterus virgatus* | AB326977 | *CYB* |
| Perciformes | Percoidei | Nemipteridae | *Parascolopsis eriomma* | HQ945943 | *CO1* |
| Perciformes | Percoidei | Nemipteridae | *Parascolopsis eriomma* | EU167793 | *rag1* |
| Perciformes | Percoidei | Nemipteridae | *Pentapodus bifasciatus* | EU167794 | *rag1* |
| Perciformes | Percoidei | Nemipteridae | *Pentapodus setosus* | EU167795 | *rag1* |
| Perciformes | Percoidei | Nemipteridae | *Scolopsis affinis* | EU167798 | *rag1* |
| Perciformes | Percoidei | Nemipteridae | *Scolopsis ciliata* | AF240753 | *CYB* |
| Perciformes | Percoidei | Nemipteridae | *Scolopsis ciliata* | EU167799 | *rag1* |
| Perciformes | Percoidei | Nemipteridae | *Scolopsis ghanam* | HQ561487 | *CO1* |
| Perciformes | Percoidei | Nemipteridae | *Scolopsis monogramma* | EF609454 | *CO1* |
| Perciformes | Percoidei | Nemipteridae | *Scolopsis taenioptera* | EF609455 | *CO1* |
| Perciformes | Percoidei | Nemipteridae | *Scolopsis taenioptera* | EU167801 | *rag1* |
| Perciformes | Percoidei | Nemipteridae | *Scolopsis vosmeri* | GU805067 | *CO1* |
| Perciformes | Percoidei | Nemipteridae | *Scolopsis vosmeri* | EU167802 | *rag1* |
| Perciformes | Percoidei | Lethrinidae | *Gnathodentex aureolineatus* | JF952744 | *CO1* |
| Perciformes | Percoidei | Lethrinidae | *Gnathodentex aureolineatus* | EU167771 | *rag1* |
| Perciformes | Percoidei | Lethrinidae | *Gymnocranius audleyi* | EF609363 | *CO1* |
| Perciformes | Percoidei | Lethrinidae | *Gymnocranius elongatus* | EF609364 | *CO1* |
| Perciformes | Percoidei | Lethrinidae | *Gymnocranius elongatus* | AF381260 | *CYB* |
| Perciformes | Percoidei | Lethrinidae | *Gymnocranius elongatus* | EU167772 | *rag1* |
| Perciformes | Percoidei | Lethrinidae | *Gymnocranius grandoculis* | EF609365 | *CO1* |
| Perciformes | Percoidei | Lethrinidae | *Gymnocranius grandoculis* | AF381275 | *CYB* |
| Perciformes | Percoidei | Lethrinidae | *Gymnocranius griseus* | AF381259 | *CYB* |
| Perciformes | Percoidei | Sparidae | *Gymnocrotaphus curvidens* | AF240700 | *CYB* |
| Perciformes | Percoidei | Lethrinidae | *Lethrinus atkinsoni* | EF609384 | *CO1* |
| Perciformes | Percoidei | Lethrinidae | *Lethrinus atkinsoni* | AF381255 | *CYB* |
| Perciformes | Percoidei | Lethrinidae | *Lethrinus atkinsoni* | EU167775 | *rag1* |
| Perciformes | Percoidei | Lethrinidae | *Lethrinus atlanticus* | AF381264 | *CYB* |
| Perciformes | Percoidei | Lethrinidae | *Lethrinus atlanticus* | EU167776 | *rag1* |
| Perciformes | Percoidei | Lethrinidae | *Lethrinus borbonicus* | AF381268 | *CYB* |
| Perciformes | Percoidei | Lethrinidae | *Lethrinus conchyliatus* | EU148536 | *CO1* |
| Perciformes | Percoidei | Lethrinidae | *Lethrinus erythracanthus* | AF381257 | *CYB* |
| Perciformes | Percoidei | Lethrinidae | *Lethrinus erythropterus* | AF381274 | *CYB* |
| Perciformes | Percoidei | Lethrinidae | *Lethrinus genivittatus* | AF381272 | *CYB* |
| Perciformes | Percoidei | Lethrinidae | *Lethrinus haematopterus* | FJ237797 | *CO1* |
| Perciformes | Percoidei | Lethrinidae | *Lethrinus haematopterus* | EU167777 | *rag1* |
| Perciformes | Percoidei | Lethrinidae | *Lethrinus harak* | HQ561476 | *CO1* |
| Perciformes | Percoidei | Lethrinidae | *Lethrinus harak* | AF381258 | *CYB* |
| Perciformes | Percoidei | Lethrinidae | *Lethrinus harak* | EU167778 | *rag1* |
| Perciformes | Percoidei | Lethrinidae | *Lethrinus laticaudis* | EF609385 | *CO1* |
| Perciformes | Percoidei | Lethrinidae | *Lethrinus laticaudis* | AF381251 | *CYB* |
| Perciformes | Percoidei | Lethrinidae | *Lethrinus lentjan* | EF609386 | *CO1* |
| Perciformes | Percoidei | Lethrinidae | *Lethrinus lentjan* | AF381267 | *CYB* |
| Perciformes | Percoidei | Lethrinidae | *Lethrinus lentjan* | EU167779 | *rag1* |
| Perciformes | Percoidei | Lethrinidae | *Lethrinus mahsena* | EF609387 | *CO1* |
| Perciformes | Percoidei | Lethrinidae | *Lethrinus microdon* | AF381265 | *CYB* |
| Perciformes | Percoidei | Lethrinidae | *Lethrinus miniatus* | EU148534 | *CO1* |
| Perciformes | Percoidei | Lethrinidae | *Lethrinus miniatus* | AF381266 | *CYB* |
| Perciformes | Percoidei | Lethrinidae | *Lethrinus nebulosus* | DQ885099 | *CO1* |
| Perciformes | Percoidei | Lethrinidae | *Lethrinus nebulosus* | AF381269 | *CYB* |
| Perciformes | Percoidei | Lethrinidae | *Lethrinus obsoletus* | AF381271 | *CYB* |
| Perciformes | Percoidei | Lethrinidae | *Lethrinus obsoletus* | EU167774 | *rag1* |
| Perciformes | Percoidei | Lethrinidae | *Lethrinus olivaceus* | EF609389 | *CO1* |
| Perciformes | Percoidei | Lethrinidae | *Lethrinus olivaceus* | AF381252 | *CYB* |
| Perciformes | Percoidei | Lethrinidae | *Lethrinus olivaceus* | EU167780 | *rag1* |
| Perciformes | Percoidei | Lethrinidae | *Lethrinus ornatus* | EF609390 | *CO1* |
| Perciformes | Percoidei | Lethrinidae | *Lethrinus ornatus* | AF240751 | *CYB* |
| Perciformes | Percoidei | Lethrinidae | *Lethrinus ornatus* | HQ676658 | *rag1* |
| Perciformes | Percoidei | Lethrinidae | *Lethrinus reticulatus* | AF381253 | *CYB* |
| Perciformes | Percoidei | Lethrinidae | *Lethrinus rubrioperculatus* | DQ885103 | *CO1* |
| Perciformes | Percoidei | Lethrinidae | *Lethrinus rubrioperculatus* | AF381263 | *CYB* |
| Perciformes | Percoidei | Lethrinidae | *Lethrinus semicinctus* | EF609391 | *CO1* |
| Perciformes | Percoidei | Lethrinidae | *Lethrinus semicinctus* | AF381276 | *CYB* |
| Perciformes | Percoidei | Lethrinidae | *Lethrinus xanthochilus* | EU167781 | *rag1* |
| Perciformes | Percoidei | Lethrinidae | *Monotaxis grandoculis* | GU805118 | *CO1* |
| Perciformes | Percoidei | Lethrinidae | *Monotaxis grandoculis* | EU167783 | *rag1* |
| Perciformes | Percoidei | Lethrinidae | *Wattsia mossambica* | AF381261 | *CYB* |
| Perciformes | Percoidei | Sparidae | *Acanthopagrus australis* | DQ107853 | *CO1* |
| Perciformes | Percoidei | Sparidae | *Acanthopagrus australis* | AB458391 | *CYB* |
| Perciformes | Percoidei | Sparidae | *Acanthopagrus berda* | EU014245 | *CO1* |
| Perciformes | Percoidei | Sparidae | *Acanthopagrus berda* | AM944832 | *CYB* |
| Perciformes | Percoidei | Sparidae | *Acanthopagrus bifasciatus* | GU805011 | *CO1* |
| Perciformes | Percoidei | Sparidae | *Acanthopagrus bifasciatus* | HM352759 | *CYB* |
| Perciformes | Percoidei | Sparidae | *Acanthopagrus butcheri* | DQ107850 | *CO1* |
| Perciformes | Percoidei | Sparidae | *Acanthopagrus butcheri* | AB458393 | *CYB* |
| Perciformes | Percoidei | Sparidae | *Acanthopagrus butcheri* | DQ354577 | *rhod* |
| Perciformes | Percoidei | Sparidae | *Acanthopagrus chinshira* | AB458397 | *CYB* |
| Perciformes | Percoidei | Sparidae | *Acanthopagrus latus* | EU871695 | *CO1* |
| Perciformes | Percoidei | Sparidae | *Acanthopagrus latus* | GU131151 | *CYB* |
| Perciformes | Percoidei | Sparidae | *Acanthopagrus schlegelii* | GU207325 | *CO1* |
| Perciformes | Percoidei | Sparidae | *Acanthopagrus schlegelii* | AB458395 | *CYB* |
| Perciformes | Percoidei | Sparidae | *Acanthopagrus schlegelii schlegelii* | EF607300 | *CO1* |
| Perciformes | Percoidei | Sparidae | *Acanthopagrus sivicolus* | AB458396 | *CYB* |
| Perciformes | Percoidei | Sparidae | *Acanthopagrus taiwanensis* | AM944818 | *CYB* |
| Perciformes | Percoidei | Sparidae | *Archosargus probatocephalus* | JN021292 | *CO1* |
| Perciformes | Percoidei | Sparidae | *Archosargus probatocephalus* | AF240716 | *CYB* |
| Perciformes | Percoidei | Sparidae | *Archosargus rhomboidalis* | GU225143 | *CO1* |
| Perciformes | Percoidei | Sparidae | *Argyrops bleekeri* | AB458407 | *CYB* |
| Perciformes | Percoidei | Sparidae | *Argyrops filamentosus* | HQ945946 | *CO1* |
| Perciformes | Percoidei | Sparidae | *Argyrops spinifer* | EU148595 | *CO1* |
| Perciformes | Percoidei | Sparidae | *Argyrops spinifer* | AF240717 | *CYB* |
| Perciformes | Percoidei | Sparidae | *Argyrops spinifer* | EU167762 | *rag1* |
| Perciformes | Percoidei | Sparidae | *Argyrozona argyrozona* | HM007755 | *CO1* |
| Perciformes | Percoidei | Sparidae | *Argyrozona argyrozona* | AF240706 | *CYB* |
| Perciformes | Percoidei | Sparidae | *Boops boops* | FN689067 | *CO1* |
| Perciformes | Percoidei | Sparidae | *Boops boops* | FJ440629 | *CYB* |
| Perciformes | Percoidei | Sparidae | *Boops boops* | EU167763 | *rag1* |
| Perciformes | Percoidei | Sparidae | *Boops boops* | EU224148 | *rhod* |
| Perciformes | Percoidei | Sparidae | *Boopsoidea inornata* | AF240711 | *CYB* |
| Perciformes | Percoidei | Sparidae | *Boopsoidea inornata* | EU167764 | *rag1* |
| Perciformes | Percoidei | Sparidae | *Calamus brachysomus* | GU440257 | *CO1* |
| Perciformes | Percoidei | Sparidae | *Calamus calamus* | EU167765 | *rag1* |
| Perciformes | Percoidei | Sparidae | *Calamus nodosus* | AF240718 | *CYB* |
| Perciformes | Percoidei | Sparidae | *Calamus penna* | AY662747 | *CO1* |
| Perciformes | Percoidei | Sparidae | *Cheimerius matsubarai* | AB458408 | *CYB* |
| Perciformes | Percoidei | Sparidae | *Cheimerius nufar* | HQ611103 | *CO1* |
| Perciformes | Percoidei | Sparidae | *Cheimerius nufar* | AF240707 | *CYB* |
| Perciformes | Percoidei | Sparidae | *Cheimerius nufar* | EU167767 | *rag1* |
| Perciformes | Percoidei | Sparidae | *Chrysoblephus anglicus* | HQ611100 | *CO1* |
| Perciformes | Percoidei | Sparidae | *Chrysoblephus anglicus* | EU167768 | *rag1* |
| Perciformes | Percoidei | Sparidae | *Chrysoblephus cristiceps* | GU804927 | *CO1* |
| Perciformes | Percoidei | Sparidae | *Chrysoblephus cristiceps* | AF240719 | *CYB* |
| Perciformes | Percoidei | Sparidae | *Chrysoblephus laticeps* | HM007751 | *CO1* |
| Perciformes | Percoidei | Sparidae | *Chrysoblephus lophus* | GU805095 | *CO1* |
| Perciformes | Percoidei | Sparidae | *Chrysoblephus puniceus* | HQ611087 | *CO1* |
| Perciformes | Percoidei | Sparidae | *Chrysophrys auratus* | DQ107829 | *CO1* |
| Perciformes | Percoidei | Sparidae | *Chrysophrys auratus* | AF240727 | *CYB* |
| Perciformes | Percoidei | Sparidae | *Crenidens crenidens* | AF240699 | *CYB* |
| Perciformes | Percoidei | Sparidae | *Cymatoceps nasutus* | AF240720 | *CYB* |
| Perciformes | Percoidei | Sparidae | *Dentex abei* | AB458400 | *CYB* |
| Perciformes | Percoidei | Sparidae | *Dentex angolensis* | DQ197841 | *rhod* |
| Perciformes | Percoidei | Sparidae | *Dentex canariensis* | DQ197842 | *rhod* |
| Perciformes | Percoidei | Sparidae | *Dentex dentex* | EU036428 | *CYB* |
| Perciformes | Percoidei | Sparidae | *Dentex dentex* | EF427464 | *rhod* |
| Perciformes | Percoidei | Sparidae | *Dentex gibbosus* | EF439249 | *CYB* |
| Perciformes | Percoidei | Sparidae | *Dentex gibbosus* | EF439333 | *rhod* |
| Perciformes | Percoidei | Sparidae | *Dentex hypselosomus* | AB458401 | *CYB* |
| Perciformes | Percoidei | Sparidae | *Dentex macrophthalmus* | EF455992 | *CYB* |
| Perciformes | Percoidei | Sparidae | *Dentex macrophthalmus* | EF427466 | *rhod* |
| Perciformes | Percoidei | Sparidae | *Dentex tumifrons* | AF240708 | *CYB* |
| Perciformes | Percoidei | Sparidae | *Diplodus annularis* | EF392581 | *CYB* |
| Perciformes | Percoidei | Sparidae | *Diplodus annularis* | EF439371 | *rhod* |
| Perciformes | Percoidei | Sparidae | *Diplodus argenteus* | AF240721 | *CYB* |
| Perciformes | Percoidei | Sparidae | *Diplodus bermudensis* | AF240722 | *CYB* |
| Perciformes | Percoidei | Sparidae | *Diplodus bermudensis* | EU167769 | *rag1* |
| Perciformes | Percoidei | Sparidae | *Diplodus cervinus* | HQ945933 | *CO1* |
| Perciformes | Percoidei | Sparidae | *Diplodus cervinus* | AJ277367 | *CYB* |
| Perciformes | Percoidei | Sparidae | *Diplodus cervinus cervinus* | EF439198 | *CYB* |
| Perciformes | Percoidei | Sparidae | *Diplodus holbrookii* | AF240724 | *CYB* |
| Perciformes | Percoidei | Sparidae | *Diplodus puntazzo* | EF455999 | *CYB* |
| Perciformes | Percoidei | Sparidae | *Diplodus puntazzo* | EU036542 | *rhod* |
| Perciformes | Percoidei | Sparidae | *Diplodus sargus* | EU239692 | *CO1* |
| Perciformes | Percoidei | Sparidae | *Diplodus sargus* | EU036435 | *CYB* |
| Perciformes | Percoidei | Sparidae | *Diplodus sargus* | EU036543 | *rhod* |
| Perciformes | Percoidei | Sparidae | *Diplodus sargus kotschyi* | HQ149831 | *CO1* |
| Perciformes | Percoidei | Sparidae | *Diplodus sargus sargus* | EF439201 | *CYB* |
| Perciformes | Percoidei | Sparidae | *Diplodus vulgaris* | FN689126 | *CO1* |
| Perciformes | Percoidei | Sparidae | *Diplodus vulgaris* | EF392587 | *CYB* |
| Perciformes | Percoidei | Sparidae | *Diplodus vulgaris* | EF439277 | *rhod* |
| Perciformes | Percoidei | Sparidae | *Evynnis cardinalis* | EU595132 | *CO1* |
| Perciformes | Percoidei | Sparidae | *Evynnis japonica* | JF952735 | *CO1* |
| Perciformes | Percoidei | Sparidae | *Evynnis japonica* | AB458402 | *CYB* |
| Perciformes | Percoidei | Sparidae | *Lagodon rhomboides* | JN026933 | *CO1* |
| Perciformes | Percoidei | Sparidae | *Lagodon rhomboides* | AF240726 | *CYB* |
| Perciformes | Percoidei | Sparidae | *Lagodon rhomboides* | EU167773 | *rag1* |
| Perciformes | Percoidei | Sparidae | *Lithognathus lithognathus* | GU805002 | *CO1* |
| Perciformes | Percoidei | Sparidae | *Lithognathus mormyrus* | EU036447 | *CYB* |
| Perciformes | Percoidei | Sparidae | *Lithognathus mormyrus* | EU167782 | *rag1* |
| Perciformes | Percoidei | Sparidae | *Lithognathus mormyrus* | EF439392 | *rhod* |
| Perciformes | Percoidei | Sparidae | *Oblada melanura* | EU036459 | *CYB* |
| Perciformes | Percoidei | Sparidae | *Oblada melanura* | EU167786 | *rag1* |
| Perciformes | Percoidei | Sparidae | *Oblada melanura* | EF439410 | *rhod* |
| Perciformes | Percoidei | Sparidae | *Pachymetopon aeneum* | AF240702 | *CYB* |
| Perciformes | Percoidei | Sparidae | *Pachymetopon blochii* | HM007695 | *CO1* |
| Perciformes | Percoidei | Sparidae | *Pachymetopon grande* | EU167787 | *rag1* |
| Perciformes | Percoidei | Sparidae | *Pagellus acarne* | FN689213 | *CO1* |
| Perciformes | Percoidei | Sparidae | *Pagellus acarne* | EF439211 | *CYB* |
| Perciformes | Percoidei | Sparidae | *Pagellus acarne* | EF439413 | *rhod* |
| Perciformes | Percoidei | Sparidae | *Pagellus bellottii* | EF456013 | *CYB* |
| Perciformes | Percoidei | Sparidae | *Pagellus bellottii* | EF427489 | *rhod* |
| Perciformes | Percoidei | Sparidae | *Pagellus bogaraveo* | EF427588 | *CYB* |
| Perciformes | Percoidei | Sparidae | *Pagellus bogaraveo* | EF439288 | *rhod* |
| Perciformes | Percoidei | Sparidae | *Pagellus erythrinus* | FN688959 | *CO1* |
| Perciformes | Percoidei | Sparidae | *Pagellus erythrinus* | EU036463 | *CYB* |
| Perciformes | Percoidei | Sparidae | *Pagellus erythrinus* | EU167790 | *rag1* |
| Perciformes | Percoidei | Sparidae | *Pagellus erythrinus* | DQ197875 | *rhod* |
| Perciformes | Percoidei | Sparidae | *Pagrus auriga* | AF240728 | *CYB* |
| Perciformes | Percoidei | Sparidae | *Pagrus auriga* | EU167788 | *rag1* |
| Perciformes | Percoidei | Sparidae | *Pagrus auriga* | DQ197876 | *rhod* |
| Perciformes | Percoidei | Sparidae | *Pagrus caeruleostictus* | HM352763 | *CYB* |
| Perciformes | Percoidei | Sparidae | *Pagrus caeruleostictus* | EU167789 | *rag1* |
| Perciformes | Percoidei | Sparidae | *Pagrus caeruleostictus* | DQ197877 | *rhod* |
| Perciformes | Percoidei | Sparidae | *Pagrus major* | GU131149 | *CYB* |
| Perciformes | Percoidei | Sparidae | *Pagrus pagrus* | EF439563 | *CYB* |
| Perciformes | Percoidei | Sparidae | *Pagrus pagrus* | AY154385 | *ND1* |
| Perciformes | Percoidei | Sparidae | *Pagrus pagrus* | EU182627 | *rag1* |
| Perciformes | Percoidei | Sparidae | *Pagrus pagrus* | EF439419 | *rhod* |
| Perciformes | Percoidei | Sparidae | *Pagrus pagrus africanus* | EU167792 | *rag1* |
| Perciformes | Percoidei | Sparidae | *Parargyrops edita* | EU871694 | *CO1* |
| Perciformes | Percoidei | Sparidae | *Parargyrops edita* | GU131152 | *CYB* |
| Perciformes | Percoidei | Sparidae | *Petrus rupestris* | GU805053 | *CO1* |
| Perciformes | Percoidei | Sparidae | *Petrus rupestris* | AF240709 | *CYB* |
| Perciformes | Percoidei | Sparidae | *Polyamblyodon germanum* | AF240703 | *CYB* |
| Perciformes | Percoidei | Sparidae | *Polysteganus praeorbitalis* | AF240710 | *CYB* |
| Perciformes | Percoidei | Sparidae | *Porcostoma dentata* | AF240730 | *CYB* |
| Perciformes | Percoidei | Sparidae | *Pterogymnus laniarius* | HM007781 | *CO1* |
| Perciformes | Percoidei | Sparidae | *Pterogymnus laniarius* | AF240731 | *CYB* |
| Perciformes | Percoidei | Sparidae | *Rhabdosargus globiceps* | HM007761 | *CO1* |
| Perciformes | Percoidei | Sparidae | *Rhabdosargus holubi* | EU167796 | *rag1* |
| Perciformes | Percoidei | Sparidae | *Rhabdosargus sarba* | DQ107833 | *CO1* |
| Perciformes | Percoidei | Sparidae | *Rhabdosargus sarba* | AB458405 | *CYB* |
| Perciformes | Percoidei | Sparidae | *Rhabdosargus thorpei* | AF240732 | *CYB* |
| Perciformes | Percoidei | Sparidae | *Sarpa salpa* | EU036484 | *CYB* |
| Perciformes | Percoidei | Sparidae | *Sarpa salpa* | EU167797 | *rag1* |
| Perciformes | Percoidei | Sparidae | *Sarpa salpa* | EF439307 | *rhod* |
| Perciformes | Percoidei | Sparidae | *Sparidentex hasta* | AF240734 | *CYB* |
| Perciformes | Percoidei | Sparidae | *Sparodon durbanensis* | AF240733 | *CYB* |
| Perciformes | Percoidei | Sparidae | *Sparus aurata* | DQ248312 | *CO1* |
| Perciformes | Percoidei | Sparidae | *Sparus aurata* | EF439594 | *CYB* |
| Perciformes | Percoidei | Sparidae | *Sparus aurata* | EF095657 | *rag1* |
| Perciformes | Percoidei | Sparidae | *Sparus aurata* | EU264050 | *rhod* |
| Perciformes | Percoidei | Sparidae | *Spondyliosoma cantharus* | EU036509 | *CYB* |
| Perciformes | Percoidei | Sparidae | *Spondyliosoma cantharus* | EF439173 | *rhod* |
| Perciformes | Percoidei | Sparidae | *Stenotomus chrysops* | AF240736 | *CYB* |
| Perciformes | Percoidei | Sparidae | *Stenotomus chrysops* | EU167806 | *rag1* |
| Perciformes | Percoidei | Sparidae | *Virididentex acromegalus* | EU167807 | *rag1* |
| Perciformes | Percoidei | Centracanthidae | *Centracanthus cirrus* | EU167766 | *rag1* |
| Perciformes | Percoidei | Centracanthidae | *Spicara alta* | AF240738 | *CYB* |
| Perciformes | Percoidei | Centracanthidae | *Spicara alta* | EU167803 | *rag1* |
| Perciformes | Percoidei | Centracanthidae | *Spicara australis* | HQ945949 | *CO1* |
| Perciformes | Percoidei | Centracanthidae | *Spicara flexuosa* | EU036503 | *CYB* |
| Perciformes | Percoidei | Centracanthidae | *Spicara flexuosa* | EU167804 | *rag1* |
| Perciformes | Percoidei | Centracanthidae | *Spicara flexuosa* | EU036607 | *rhod* |
| Perciformes | Percoidei | Centracanthidae | *Spicara maena* | EF439598 | *CYB* |
| Perciformes | Percoidei | Centracanthidae | *Spicara maena* | EU167805 | *rag1* |
| Perciformes | Percoidei | Centracanthidae | *Spicara smaris* | EF439599 | *CYB* |
| Perciformes | Percoidei | Centracanthidae | *Spicara smaris* | EU036605 | *rhod* |
| Perciformes | Percoidei | Polynemidae | *Eleutheronema tetradactylum* | FJ347964 | *CO1* |
| Perciformes | Percoidei | Polynemidae | *Filimanus heptadactyla* | EF609524 | *CO1* |
| Perciformes | Percoidei | Polynemidae | *Leptomelanosoma indicum* | EF609538 | *CO1* |
| Perciformes | Percoidei | Polynemidae | *Pentanemus quinquarius* | EU167859 | *rag1* |
| Perciformes | Percoidei | Polynemidae | *Pentanemus quinquarius* | AY141317 | *rhod* |
| Perciformes | Percoidei | Polynemidae | *Polydactylus approximans* | GU440471 | *CO1* |
| Perciformes | Percoidei | Polynemidae | *Polydactylus macrochir* | EU167863 | *rag1* |
| Perciformes | Percoidei | Polynemidae | *Polydactylus plebeius* | JF952821 | *CO1* |
| Perciformes | Percoidei | Polynemidae | *Polydactylus plebeius* | AB264369 | *CYB* |
| Perciformes | Percoidei | Polynemidae | *Polydactylus sexfilis* | FJ487622 | *ATP6* |
| Perciformes | Percoidei | Polynemidae | *Polydactylus sextarius* | EU392177 | *CO1* |
| Perciformes | Percoidei | Polyprionidae | *Polyprion americanus* | DQ107915 | *CO1* |
| Perciformes | Percoidei | Polyprionidae | *Polyprion americanus* | EF439219 | *CYB* |
| Perciformes | Percoidei | Polyprionidae | *Polyprion americanus* | EF439297 | *rhod* |
| Perciformes | Percoidei | Polyprionidae | *Polyprion oxygeneios* | DQ107904 | *CO1* |
| Perciformes | Percoidei | Polyprionidae | *Stereolepis gigas* | GU440534 | *CO1* |
| Perciformes | Percoidei | Polyprionidae | *Stereolepis gigas* | DQ336172 | *ND2* |
| Perciformes | Percoidei | Polyprionidae | *Stereolepis gigas* | DQ336173 | *rhod* |
| Perciformes | Percoidei | Sciaenidae | *Aplodinotus grunniens* | EU523921 | *CO1* |
| Perciformes | Percoidei | Sciaenidae | *Aplodinotus grunniens* | AY225662 | *CYB* |
| Perciformes | Percoidei | Sciaenidae | *Aplodinotus grunniens* | AY225720 | *ND2* |
| Perciformes | Percoidei | Sciaenidae | *Argyrosomus hololepidotus* | DQ107810 | *CO1* |
| Perciformes | Percoidei | Sciaenidae | *Argyrosomus inodorus* | HM007713 | *CO1* |
| Perciformes | Percoidei | Sciaenidae | *Argyrosomus japonicus* | HM007718 | *CO1* |
| Perciformes | Percoidei | Sciaenidae | *Argyrosomus regius* | EF455986 | *CYB* |
| Perciformes | Percoidei | Sciaenidae | *Argyrosomus regius* | EU637942 | *rhod* |
| Perciformes | Percoidei | Sciaenidae | *Atractoscion aequidens* | HM007698 | *CO1* |
| Perciformes | Percoidei | Sciaenidae | *Atractoscion aequidens* | DQ197828 | *rhod* |
| Perciformes | Percoidei | Sciaenidae | *Atractoscion nobilis* | GQ220049 | *ATP6* |
| Perciformes | Percoidei | Sciaenidae | *Atractoscion nobilis* | GU440241 | *CO1* |
| Perciformes | Percoidei | Sciaenidae | *Atractoscion nobilis* | GQ220018 | *CYB* |
| Perciformes | Percoidei | Sciaenidae | *Bairdiella armata* | GQ220055 | *ATP6* |
| Perciformes | Percoidei | Sciaenidae | *Bairdiella armata* | GQ220024 | *CYB* |
| Perciformes | Percoidei | Sciaenidae | *Bairdiella chrysoura* | GU225146 | *CO1* |
| Perciformes | Percoidei | Sciaenidae | *Bairdiella chrysoura* | DQ060514 | *CYB* |
| Perciformes | Percoidei | Sciaenidae | *Bairdiella ronchus* | GQ220056 | *ATP6* |
| Perciformes | Percoidei | Sciaenidae | *Bairdiella ronchus* | GU225151 | *CO1* |
| Perciformes | Percoidei | Sciaenidae | *Bairdiella ronchus* | GQ220025 | *CYB* |
| Perciformes | Percoidei | Sciaenidae | *Bairdiella sanctaeluciae* | GU225153 | *CO1* |
| Perciformes | Percoidei | Sciaenidae | *Cheilotrema saturnum* | GU440274 | *CO1* |
[truncated: 532,277 more chars]
