# Supplementary material for: Evolutionary Origin of the Scombridae (Tunas and Mackerels): Members of a Paleogene Adaptive Radiation with 14 Other Pelagic Fish Families
Source: PLoS One. 2013 Sep 4;8(9):e73535. doi: 10.1371/journal.pone.0073535 (PMC3762723; doi:10.1371/journal.pone.0073535)
Supplement: Table S3 — List of species used in the mitogenomic analysis. (DOCX) [file pone.0073535.s004.docx]

**Table S3** List of the species used in the mitogenomic analysis

| Order | Suborder | Family | Species | Accession No. |
| --- | --- | --- | --- | --- |
| Aulopiformes | Synodontoidei | Chlorophthalmidae | *Chlorophthalmus_agassizi* | AP002918 |
| Polymixiiformes |  | Polymixiidae | *Polymixia_japonica* | AB034826 |
| Gadiformes |  | Gadidae | *Gadus morhua* | X99772 |
| Beloniformes |  | Adrianichthyidae | *Oryzias latipes* | AP004421 |
| Atheriniformes |  | Goodeidae | *Xenotoca eiseni* | AP006777 |
| Atheriniformes |  | Cyprinodontidae | *Jordanella floridae* | AP006778 |
| Beryciformes | Berycoidei | Berycidae | *Beryx splendens* | AP002939 |
| Beryciformes | Holocentroidei | Holocentridae | *Sargocentron rubrum* | AP004432 |
| Gasterosteiformes | Gasterosteoidei | Gasterosteidae | *Gasterosteus aculeatus* | AP002944 |
| Gasterosteiformes | Syngnathoidei | Macroramphosidae | *Macroramphosus scolopax* | AP005988 |
| Gasterosteiformes | Syngnathoidei | Centriscidae | *Aeoliscus strigatus* | AP009198 |
| Synbranchiformes | Synbranchoidei | Synbranchidae | *Monopterus albus* | AP002945 |
| Synbranchiformes | Synbranchoidei | Synbranchidae | *Synbranchus marmoratus* | AP004439 |
| Syngnathiformes | Mastacembeloidei | Mastacembelidae | *Mastacembelus favus* | AP002946 |
| Scorpaeniformes | Dactylopteroidei | Dactylopteridae | *Dactyloptena tiltoni* | AP004440 |
| Scorpaeniformes | Scorpaenoidei | Scorpaenidae | *Helicolenus hilgendorfi* | AP002948 |
| Scorpaeniformes | Platycephaloidei | Platycephalidae | *Platycephalus indicus* | AP006783 |
| Perciformes | Percoidei | Latidae | *Lates calcarifer* | DQ010541 |
| Perciformes | Percoidei | Serranidae | *Epinephelus merra* | AP005991 |
| Perciformes | Percoidei | Apogonidae | *Apogon semilineatus* | AP005996 |
| Perciformes | Percoidei | Apogonidae | *Pterapogon kauderi* | AP005997 |
| Perciformes | Percoidei | Pomatomidae | *Pomatomus saltarix* | AB355904 |
| Perciformes | Percoidei | Coryphaenidae | *Coryphaena equiselis* | AB355907 |
| Perciformes | Percoidei | Coryphaenidae | *Coryphaena hippurus* | AB355908 |
| Perciformes | Percoidei | Rachycentridae | *Rachycentron canadum* | AB355906 |
| Perciformes | Percoidei | Echeneidae | *Echeneis naucrates* | AB355905 |
| Perciformes | Percoidei | Carangidae | *Carangoides armatus* | AP004444 |
| Perciformes | Percoidei | Carangidae | *Caranx melampygus* | AP004445 |
| Perciformes | Percoidei | Carangidae | *Trachurus japonicus* | AP003091 |
| Perciformes | Percoidei | Menidae | *Mene maculata* | AB355909 |
| Perciformes | Percoidei | Bramidae | *Brama japonica* | AP005998 |
| Perciformes | Percoidei | Bramidae | *Eumegistus illustris* | AP012497 |
| Perciformes | Percoidei | Bramidae | *Taractes asper* | AP012498 |
| Perciformes | Percoidei | Bramidae | *Pteraclis aesticola* | AP012499 |
| Perciformes | Percoidei | Caristiidae | *Platyberyx macropus* | AP005999 |
| Perciformes | Percoidei | Sparidae | *Pagrus major* | AP002949 |
| Perciformes | Percoidei | Toxotidae | *Toxotes jaculatorix* | AP006806 |
| Perciformes | Percoidei | Arripidae | *Arripis trutta* | AP006810 |
| Perciformes | Percoidei | Cirrhitidae | *Paracirrhites arcatus* | AP006012 |
| Perciformes | Labroidei | Cichlidae | *Astronotus ocellatus* | AP009127 |
| Perciformes | Labroidei | Cichlidae | *Etroplus maculatus* | AP009505 |
| Perciformes | Labroidei | Cichlidae | *Hypselecara temporalis* | AP009506 |
| Perciformes | Labroidei | Cichlidae | *Neolamprologus brichardi* | AP006014 |
| Perciformes | Labroidei | Cichlidae | *Oreochromis sp* | AP009126 |
| Perciformes | Labroidei | Cichlidae | *Paratilapia polleni* | AP009508 |
| Perciformes | Labroidei | Cichlidae | *Paretroplus maculatus* | AP009505 |
| Perciformes | Labroidei | Cichlidae | *Ptychochromoides katria* | AP009507 |
| Perciformes | Labroidei | Cichlidae | *Tropheus duboisi* | AP006015 |
| Perciformes | Labroidei | Cichlidae | *Tylochromis polylepis* | AP009509 |
| Perciformes | Labroidei | Pomacentridae | *Abudefduf vaigiensis* | AP006016 |
| Perciformes | Labroidei | Pomacentridae | *Amphiprion ocellaris* | AP006017 |
| Perciformes | Labroidei | Labridae | *Halichoeres melanurus* | AP006018 |
| Perciformes | Labroidei | Labridae | *Pseudolabrus sieboldi* | AP006019 |
| Perciformes | Trachinoidei | Chiasmodontidae | *Chiasmodon niger* | AP006815 |
| Perciformes | Trachinoidei | Chiasmodontidae | *Dysalotus alcocki* | AP006814 |
| Perciformes | Trachinoidei | Chiasmodontidae | *Kali indica* | AP012500 |
| Perciformes | Trachinoidei | Trichodontidae | *Arctoscopus japonicus* | AP003090 |
| Perciformes | Icosteoidei | Icosteidae | *Icosteus aenigmaticus* | AP006026 |
| Perciformes | Gobioidei | Rhyacichthyidae | *Rhyacichthys aspro* | AP004454 |
| Perciformes | Gobioidei | Eleotridae | *Eleotris acanthopoma* | AP004455 |
| Perciformes | Kurtoidei | Kurtidae | *Kurtus gulliveri* | AP006030 |
| Perciformes | Acanthuroidei | Luvaridae | *Luvarus imperialis* | AP009161 |
| Perciformes | Scombroidei | Scombrolabracidae | *Scombrolabrax heterolepis* | AP012517 |
| Perciformes | Scombroidei | Sphyraenidae | *Sphyraena baracuda* | AP006828 |
| Perciformes | Scombroidei | Sphyraenidae | *Sphyraena japonica* | AP012501 |
| Perciformes | Scombroidei | Gempylidae | *Gempylus serpens* | AP012502 |
| Perciformes | Scombroidei | Gempylidae | *Nesiarchus nasutus* | AP012503 |
| Perciformes | Scombroidei | Gempylidae | *Prometichthys prometheus* | AP012504 |
| Perciformes | Scombroidei | Gempylidae | *Thyrsitoides marleyi* | AP012505 |
| Perciformes | Scombroidei | Gempylidae | *Ruvettus pretiosus* | AP012506 |
| Perciformes | Scombroidei | Gempylidae | *Lepidocybium flavobrunneum* | AP012519 |
| Perciformes | Scombroidei | Gempylidae | *Rexea nakamurai* | AP012520 |
| Perciformes | Scombroidei | Gempylidae | *Nealotus tripes* | AP012521 |
| Perciformes | Scombroidei | Gempylidae | *Epinnula magistralis* | AP012943 |
| Perciformes | Scombroidei | Trichiuridae | *Trichiurus lepturus* | AP012507 |
| Perciformes | Scombroidei | Trichiuridae | *Trichiurus japonicus* | EU339148 |
| Perciformes | Scombroidei | Trichiuridae | *Assurger anzac* | AP012508 |
| Perciformes | Scombroidei | Trichiuridae | *Evoxymetopon poeyi* | AP012509 |
| Perciformes | Scombroidei | Trichiuridae | *Aphanopus carbo* | AP012944 |
| Perciformes | Scombroidei | Trichiuridae | *Benthodesmus tenuis* | AP012522 |
| Perciformes | Scombroidei | Scombridae | *Auxis rochei* | AB103467 |
| Perciformes | Scombroidei | Scombridae | *Auxis thazard* | AB105447 |
| Perciformes | Scombroidei | Scombridae | *Euthynnus alletteratus* | AB099716 |
| Perciformes | Scombroidei | Scombridae | *Gasterochisma melampus* | AP006033 |
| Perciformes | Scombroidei | Scombridae | *Gymnosarda unicolor* | AP012510 |
| Perciformes | Scombroidei | Scombridae | *Katsuwonus pelamis* | AB101290 |
| Perciformes | Scombroidei | Scombridae | *Scomber australasicus* | AB488407 |
| Perciformes | Scombroidei | Scombridae | *Scomber colias* | AB488406 |
| Perciformes | Scombroidei | Scombridae | *Scomber japonicus* | AB488405 |
| Perciformes | Scombroidei | Scombridae | *Scomber scombrus* | AB120717 |
| Perciformes | Scombroidei | Scombridae | *Scomberomorus cavalla* | DQ536428 |
| Perciformes | Scombroidei | Scombridae | *Thunnus thynnus* | GU256522 |
| Perciformes | Scombroidei | Scombridae | *Rastrelliger brachysoma* | EU555283 |
| Perciformes | Scombroidei | Scombridae | *Thunnus_alalunga* | AB101291 |
| Perciformes | Scombroidei | Scombridae | *Thunnus_albacares* | GU256528 |
| Perciformes | Scombroidei | Scombridae | *Thunnus_maccoyii* | GU256523 |
| Perciformes | Scombroidei | Scombridae | *Thunnus_obesus* | GU256525 |
| Perciformes | Scombroidei | Scombridae | *Thunnus_orientalis* | AB185022 |
| Perciformes | Scombroidei | Xiphiidae | *Xiphias gladius* | AP006036 |
| Perciformes | Scombroidei | Istiophoridae | *Istiophorus albicans* | AP006035 |
| Perciformes | Scombroidei | Istiophoridae | *Istiophorus platypterus* | AB470306 |
| Perciformes | Scombroidei | Istiophoridae | *Makaira indica* | AB470305 |
| Perciformes | Scombroidei | Istiophoridae | *Makaira mazara* | AB470304 |
| Perciformes | Scombroidei | Istiophoridae | *Tetrapturus angustirostris* | AB470303 |
| Perciformes | Scombroidei | Istiophoridae | *Tetrapturus audax* | AB470302 |
| Perciformes | Stromateoidei | Centrolophoridae | *Hyperoglyphe japonica* | AP006037 |
| Perciformes | Stromateoidei | Centrolophoridae | *Icichthys lockingtoni* | AP012511 |
| Perciformes | Stromateoidei | Centrolophoridae | *Psenopsis anomala* | AP011067 |
| Perciformes | Stromateoidei | Nomeidae | *Cubiceps pauciradiatus* | AP006038 |
| Perciformes | Stromateoidei | Nomeidae | *Psenes cyanophrys* | AP011067 |
| Perciformes | Stromateoidei | Ariommatidae | *Ariomma lurida* | AP012512 |
| Perciformes | Stromateoidei | Ariommatidae | *Ariomma indica* | AP012513 |
| Perciformes | Stromateoidei | Tetragonuridae | *Tetragonurus cuvieri* | AP012514 |
| Perciformes | Stromateoidei | Tetragonuridae | *Tetragonurus atlanticus* | AP012515 |
| Perciformes | Stromateoidei | Stromateidae | *Pampus argenteus* | EU357803 |
| Perciformes | Stromateoidei | Stromateidae | *Pampus* species | AP012516 |
| Perciformes | Stromateoidei | Stromateidae | *Peprilus triacanthus* | AP012518 |
| Perciformes | Anabantoidei | Osphronemidae | *Colisa lalia* | AP006039 |
| Perciformes | Chanoidei | Channidae | *Channa argus* | AP006041 |
| Perciformes | Chanoidei | Channidae | *Parachanna insignis* | AP006042 |
| Pleuronectiformes | Pleuronectoidei | Paralichthyidae | *Paralichthys olivaceus* | AB028664 |
| Perciformes | Pleuronectoidei | Pleuronectidae | *Platichthys bicoloratus* | AP002951 |
| Tetraodontiformes | Tetraodontoidei | Tetraodontidae | *Takifugu rubripes* | AP006045 |
| Tetraodontiformes | Tetraodontoidei | Tetraodontidae | *Tetraodon nigroviridis* | AP006046 |
