# Supplementary material for: Evolutionary Origin of the Scombridae (Tunas and Mackerels): Members of a Paleogene Adaptive Radiation with 14 Other Pelagic Fish Families
Source: PLoS One. 2013 Sep 4;8(9):e73535. doi: 10.1371/journal.pone.0073535 (PMC3762723; doi:10.1371/journal.pone.0073535)
Supplement: Table S5 — Paleontological timescale for the 15 families of Pelagia. (DOCX) [file pone.0073535.s006.docx]

**Table S5** Palaeontological timescale for the 15 families of Pelagia.

|  | **Trichiuridae** | **Gempylidae** | **Caristiidae** | **Icosteidae** | **Bramidae** |
| --- | --- | --- | --- | --- | --- |
| **LAD (Ma)** | 2.588 | 5.333 | 7.246 | 0 | 2.588 |
| **FAD (Ma)** | 61.6 | 56 | 56 | 0 | 49.11 |
| **Duration (Ma)** | 59.012 | 50.667 | 48.754 | 0 | 46.522 |
| ***N*_horizons_** | 113 | 16 | 5 | 0 | 3 |
| **CI_0.975_** | 63.57600691 | 70.12616426 | 129.8558189 | - | 296.8189626 |
| **CI_0.95_** | 63.19972903 | 67.20020889 | 110.3481572 | - | 210.6407089 |
| **CI_0.5_** | 61.96634676 | 58.39625132 | 65.22460368 | - | 68.38004335 |
| **CI_0.025_** | 61.61334128 | 56.0855907 | 56.30956476 | - | 49.70266084 |
| **CI_0.5, 10%_** | 65.26346755 | 79.96251321 | 148.2460368 | - | 241.8104335 |
|  |  |  |  |  |  |
|  | **Scombrolabracidae** | **Centrolophidae** | **Tetragonuridae** | **Chiasmodontidae** | **Stromateidae** |
| **LAD (Ma)** | 11.62 | 11.62 | 2.588 | 11.62 | 15.97 |
| **FAD (Ma)** | 11.62 | 49.11 | 2.588 | 11.62 | 31.35 |
| **Duration (Ma)** | 0 | 37.49 | 0 | 0 | 15.38 |
| ***N*_horizons_** | 1 | 3 | 1 | 1 | 6 |
| **CI_0.975_** | - | 248.727579 | - | - | 48.13387264 |
| **CI_0.95_** | - | 179.280377 | - | - | 43.97027744 |
| **CI_0.5_** | - | 64.63886645 | - | - | 33.6369807 |
| **CI_0.025_** | - | 49.58759887 | - | - | 31.42807508 |
| **CI_0.5, 10%_** | - | 204.3986645 | - | - | 54.219807 |
|  |  |  |  |  |  |
|  | **Ariommatidae** | **Nomeidae** | **Pomatomidae** | **Arripidae** | **Scombridae** |
| **LAD (Ma)** | 5.333 | 2.588 | 3.6 | 0 | 0.126 |
| **FAD (Ma)** | 31.35 | 38 | 49.11 | 0 | 61.6 |
| **Duration (Ma)** | 26.017 | 35.412 | 45.51 | 0 | 61.474 |
| ***N*_horizons_** | 6 | 9 | 8 | 0 | 126 |
| **CI_0.975_** | 59.74180848 | 58.7455244 | 80.68547423 | - | 63.44119558 |
| **CI_0.95_** | 52.69861887 | 54.08467693 | 73.41813818 | - | 63.09107322 |
| **CI_0.5_** | 35.2186851 | 41.20505983 | 53.84711377 | - | 61.94183112 |
| **CI_0.025_** | 31.48207278 | 38.1122468 | 49.27489995 | - | 61.61245236 |
| **CI_0.5, 10%_** | 70.03685102 | 70.05059829 | 96.48113767 | - | 65.01831119 |
