# Supplementary material for: Evolutionary Origin of the Scombridae (Tunas and Mackerels): Members of a Paleogene Adaptive Radiation with 14 Other Pelagic Fish Families
Source: PLoS One. 2013 Sep 4;8(9):e73535. doi: 10.1371/journal.pone.0073535 (PMC3762723; doi:10.1371/journal.pone.0073535)
Supplement: Text S1 — First appearances of the 15 families of Pelagia as body fossils. (DOCX) [file pone.0073535.s007.docx]

**Text S2 First Appearances of Pelagian Families as Body Fossils**

Here we provide minimum times of origin for the 15 families of Pelagia based on the body fossil record. Where first appearances are marked by fragmentary or controversial material, we provide details for the next oldest taxon represented by more definitive remains. Many of the dates provided here represent revisions to those provided for the same families in Patterson’s review of the teleost fossil record [[1](#_ENREF_1)], or new accounts for families not treated independently by Patterson. We have marked such entries with an asterisk (“*”). Age estimates given below are taken from Gradstein *et al*. [[2](#_ENREF_2)].

**Trichiuridae***: Isolated teeth assigned to *Eutrichiurides* from the Danian of Landana, Angola [[3](#_ENREF_3)] and Imin’Tanout, Atlas Mountains, Morocco represent the earliest Trichiuridae [[4](#_ENREF_4)]. The oldest articulated remains of trichiurids are cranial material of *Eutrichurides* from the Ypresian London Clay Formation, UK [[5](#_ENREF_5)], and an articulated specimen of a trichiurid similar to *Anenchelum* from the Ypresian of Monte Salone, Italy [[6](#_ENREF_6)]. We have assigned a fossil-based first appearance of the group of 61.6 Ma, corresponding to the top of the Danian.

**“Gempylidae”*:** Articulated specimen assigned to *Argestichthys* from the latest Thanetian-earliest Ypresian Danatinsk Suite, Turkmenistan represents the earliest ‘Gempylidae’ [[7](#_ENREF_7)]. We have assigned a fossil-based first appearance of the group of 56.0 Ma, corresponding to the Thanetian-Ypresian boundary.

**Caristiidae:** An articulated specimen assigned to *Exellia* from the latest Thanetian-earliest Ypresian Danatinsk Suite, Turkmenistan represents the earliest member of total-group Caristiidae [[1](#_ENREF_1),[8](#_ENREF_8)]. We have assigned a fossil-based first appearance of the group of 56.0 Ma, corresponding to the Thanetian-Ypresian boundary. The interpretation of exelliids as close relatives of caristiids has not been formally tested by cladistic analysis, and the earliest fossils that are definitively attributable to Caristiidae are of Miocene age [[9](#_ENREF_9)].

**Icosteidae:** No fossil record.

**Bramidae:** Intact braincase assigned to *Bramoides* from the Ypresian London Clay, UK [[10](#_ENREF_10)]. The London Clay spans nannoplankton zones NP11 and NP12 [[11](#_ENREF_11)]. The top of NP12 is dated to approximately 49.11 Ma, from which we drive our fossil based first appearance of Bramidae. Patterson [[1](#_ENREF_1)] has questioned the identity of *Bramoides*. If this taxon is not a bramid, the oldest representative of the group would be the Rupelian [[12](#_ENREF_12)].

**Scombrolabracidae*:** Articulated specimen assigned to *Sombrolabrax* from the middle Miocene Himaka Formation of the Morozaki Group, Japan [[13](#_ENREF_13)]. The middle Miocene is an informal stratigraphic division, corresponding to the middle two stages of the Miocene. The top of the top of the Serravallian is dated as 11.62 Ma, from which we derive our estimate for the first appearance of Scombrolabracidae based on fossils.

**Centrolophidae*:** An articulated specimen assigned to *Zorzinia* from Bolca, Italy has been tentatively placed in Centrolophidae [[14](#_ENREF_14)].The fish-bearing limestones at Bolca have been placed at the base of NP14, indicating a late Ypresian age [[15](#_ENREF_15)]. The base of NP14 is dated as 49.11 Ma, which represents a fossil-based minimum age for Centrolophidae.

**Tetragonuridae:** An articulated *Tetragonurus* from the Piacenzian of Marecchia River, Italy represents the first and only tetragonurid body fossil [[16](#_ENREF_16)]. The top of the Piacenzian is dated as 2.588 Ma, which represents a fossil-based age for Tetragonuridae.

**Chiasmodontidae*:** An articulated specimen of *Bannikovichthys* from the Serravallian of Torricella Pelgina, Italy represents the oldest Champsodontidae [[17](#_ENREF_17)]. The top of the Serravallian is dated as 11.62 Ma, representing a fossil-based minimum age for Chiasmodontidae. Older references [[1](#_ENREF_1)] list the Rupelian *Pseudoscopelus* as a chiasmodontid, but Bannikov [[18](#_ENREF_18)] has convincingly argued that this taxon is a champsodontid.

**Stromateidae*:** Articulated material of *Pinichthys* is known from roughly coeval deposits in Poland, Frauenweiler, Germany, and the North Caucasus [[19](#_ENREF_19)], all of which are considered as Rupelian in age; collectively these materials represent the oldest examples of Stromateidae. The top of the Pshekha Horizon in the North Caucasus corresponds approximately to the base of NP23 [[20](#_ENREF_20)]. This has been dated to 31.35 Ma, which represents a palaeontological minimum age for Stromateidae.

**Ariommatidae*:** Articulated specimens of *Isurichthys* are known from early Oligocene deposits in Switzerland, Romania, Iran, Ukraine, and the Caucasus [[19](#_ENREF_19)], and represent the earliest occurring ariommatids. These horizons are considered to be of Rupelian age. The top of the Pshekha Horizon in the North Caucasus corresponds approximately to the base of NP23 [[20](#_ENREF_20)]. This has been dated to 31.35 Ma, which represents a palaeontological minimum age for Ariommatidae.

**Nomeidae*:** Articulated material of *Rybapina* from the Bartonian Kuma Horizon, North Caucasus, has been assigned to Ariommatidae [[21](#_ENREF_21)]. The top of the Bartonian is dated as 38.0 Ma, which represents a fossil-based minimum age for Ariommatidae.

**Pomatomidae:** Articulated material of *Carangopsis* from Bolca, Italy has been tentatively assigned to Pomatomidae [[22](#_ENREF_22)]. The fish-bearing limestones at Bolca have been placed at the base of NP14, indicating a late Ypresian age [[15](#_ENREF_15)]. The base of NP14 is dated as 49.11 Ma, which represents a fossil-based minimum age for Pomatomidae. If the interpretation of *Carangopsis* as a pomatomid is incorrect, then the earliest representative of this family would be *Lednevia*, from the Zuramakent/Sakaraul horizon of the Caucasus. These deposits are regarded as early Miocene (Aquitanian-Burdigalian) in age [[21](#_ENREF_21)].

**Arripidae:** No fossil record.

**Scombridae:** Articulated cranial material of *Landanichthys* from ‘Bed 5’, Landana, Angola represents the oldest material of Scombridae [[3](#_ENREF_3)]. The horizon yielding *Landanichthys* has been interpreted as Danian in age. The top of the Danian is dated as 61.6 Ma, which represents a fossil-based minimum for the appearance of Scombridae.

**References**

1. Patterson C (1993) Osteichthyes: Teleostei. In: Benton MJ, editor. The fossil record 2. London, UK: Chapman & Hall. pp. 621–656.

2. Gradstein FM, Ogg JG, Schmitz M (2012) A geologic time scale 2012. Amsterdam: Elsevier.

3. Dartevelle E, Casier E (1959) Les poissons fossiles du Bas-Congo et des régions voisines. Ann Mus Roy Congo Belge A Sér III 2: 257–568.

4. Arambourg C, Signeux J (1952) Les vertébrés fossiles des gisements de phosphates (Maroc-Algérie-Tunisie). Mém du Serv Géolog Maroc 92: 1–372.

5. Monsch KA (2004) Revision of the scombroid fishes from the Cenozoic of England. Transactions of the Royal Society of Edinburgh: Earth Sciences 95: 445–489.

6. Zorzin R, Bannikov AF, Fornaciari E, Giusberti L, Papazzoni CA, et al. (2011) Ill giacimento a pesci e piante fossili dell'Eocene inferiore di Monte Solane (Verona). Boll Mus Civ Stor Nat Verona Geol Paleontol Preist 35: 57–64.

7. Prokofiev AM (2002) A new genus of cutlassfish from the upper Paleocene of Turkmenistan (Scombroidei: Trichiuroidea). Zoosyst Ross 11: 229–233.

8. Bannikov AF, Tyler JC (2004) A revision of the Eocene fish family Exellidae (Perciformes). Paleontol J 27: 128–140.

9. Fierstine HL, Huddleston RW, Takeuchi GT (2012) Neogene bony fishes of California: a systematic inventory of all published accounts. Occ Pap Calif Acad Sci 159: 1–206.

10. Casier E (1966) Faune ichthyologique du London Clay. London: Trustees of the British Museum (Natural History), London.

11. Ellison RA, Knox RW, Jolley DW, King C (1994) A revision of the lithostratigraphical classification of the early Palaeogene strata of the London Basin and East Anglia. Proceedings of the Geologists' Association 105: 187–197.

12. Baciu DS, Bannikov AF (2003) *Paucaichthys neamtensis* gen. et sp. nova—the first discovery of sea breams (Bramidae) in the Oligocene of Romania. J Ichthyol 43: 598–602.

13. Ohe F (1993) Osteichthyes. Deep fish assemblage from the Middle Miocene Morozaki Group, southern part of Chita Penninsula, Aichi Prefecture, central Japan. In: Society TF, editor. Fossils from the Miocene Morozaki Group. Nagoya, Japan: The Tokai Fossil Society, Nagoya. pp. 199–262.

14. Bannikov AF (2000) A new genus and species of putative centrolophid fish (Perciformes, Stromateoidei) from the Eocene of Bolca, northern Italy. Boll Mus Civ Stor Natur Verona Geol Paleontol Preist 24: 37–46.

15. Papazzoni CA, Trevisani E (2006) Facies analysis, palaeoenvironmental reconstruction, and biostratigraphy of the ‚ÄúPesciara di Bolca‚Äù(Verona, northern Italy): An early Eocene Fossil-Lagerstätte. Palaeogeogr Palaeoclimatol Palaeoecol 242: 21-35.

16. Landini W, Sorbini L (1992) Données récentes sur les téleostéens due Miocenè et du Pliocène d'Italie. Geobios MS 14: 151–157.

17. Carnevale G (2005) Fossil fishes from the Serravallian (middle Miocene) of Torricella Peligna, Italy. Palaeontogr Ital 91: 1–67.

18. Bannikov AF (1998) On the belonging to the family Champsodontidae (Perciformes) of a Caucasian Oligocene species previously classified with family Chaismodontidae. J Ichthyol 38: 478–480.

19. Bannikov AF (2012) The first record of the genus *Isurichthys* (Perciformes, Ariommatidae) in the lower Oligocene of the northern Caucasus. Paleontol J 46: 171–176.

20. Lenov YG, editor (1998) Late Eocene-Early Oligocene geological and biotical events on the territory of the former Soviet Union. Part II. The geological and biotical events. Moscow: GEOS.

21. Bannikov AF (1996) The list of marine fishes from Cenozoic (upper Paleocene-middle Miocene) localities in southern European Russia and adjacent countries. J Ichthyol 37: 133–146.

22. Blot J (1969) Les poissons fossiles du Monte Bolca. Classés jusqu’ici dans les familles des Carangidae, Menidae, Ephippidae, Scatophagidae. Studi Ricer GiaciamenTerz Bolca 1: 1–525.
