# Supplementary material for: Evolutionary Origin of the Scombridae (Tunas and Mackerels): Members of a Paleogene Adaptive Radiation with 14 Other Pelagic Fish Families
Source: PLoS One. 2013 Sep 4;8(9):e73535. doi: 10.1371/journal.pone.0073535 (PMC3762723; doi:10.1371/journal.pone.0073535)
Supplement: Text S2 — Tree topologies in a newick format shown in Figures 2 – 4 . (DOCX) [file pone.0073535.s008.docx]

Figure 2: ATP6 tree

((Sargocentron_rubrum:0.26136011870277531255,(Beryx_splendens:0.52923751754438486117,((Caranx_melampygus:0.12960179999884871571,Caranx_ignobilis:0.09441180555221463655)98:0.46200446438260650561,(((((((Hypoplectrus_indigo:0.00000332819398067269,((Hypoplectrus_puella:0.00000332819398067269,(Hypoplectrus_aberrans:0.00468737967394169258,Hypoplectrus_guttavarius:0.00463171880369247363)62:0.00157325374371702930)12:0.00000332819398067269,(Hypoplectrus_chlorurus:0.00796343883602351407,Hypoplectrus_unicolor:0.00000332819398067269)70:0.00312341654878961613)24:0.00154408461434467261)95:0.16847011311690721080,(Serranus_tabacarius:0.20238381676445743595,Serranus_tortugarum:0.01815089820622005010)99:0.23346943675675624341)89:0.20372695167164622809,((Serranus_psittacinus:0.22270643052529104366,(Serranus_tigrinus:0.48526641715092239115,Serranus_baldwini:0.65564069130154378762)75:0.12722712909315764263)100:0.43913474909098432208,(Bovichtus_variegatus:0.66633345601935367597,(Gerlachea_australis:0.00000332819398067269,((Trematomus_eulepidotus:0.54973270490727155213,(Pseudochaenichthys_georgianus:0.08367284366400244700,Chaenocephalus_aceratus:0.03233110953529601261)60:0.01686636776161272444)26:0.03093596626205997319,Harpagifer_antarcticus:0.12524850601093351354)19:0.07995714845094202550)100:1.54416890204909473816)90:0.88855506458661093205)9:0.08685269688723182280)16:0.16637931207546768908,((Aulorhynchus_flavidus:1.49670638998628957594,Spinachia_spinachia:0.56591409330758768448)59:0.22651495223801026979,(Apeltes_quadracus:0.30322016075130114876,(Culaea_inconstans:0.19740165700248898939,(Gasterosteus_wheatlandi:0.26964121878092467410,Pungitius_pungitius:0.19974426501835904069)21:0.03360458661482541853)27:0.02135471467815787047)75:0.10894082231514111425)75:0.35537106826653719338)5:0.12135106832169494095,((Perca_flavescens:0.19501648009891298097,((Paracottus_knerii:0.00937705517480601040,((Cottocomephorus_grewingki:0.00397629431600013232,Cottocomephorus_inermis:0.00362368964980743554)99:0.04560581572921858229,(((((Cottus_ricei:0.01175020024498637898,(Cottus_sibiricus:0.00806875714444823915,(Cottus_gobio:0.00356124320236934997,Cottus_ferrugineus:0.00366283075731277039)98:0.01307556370261418570)68:0.00371490420859953716)90:0.02064673817709039919,(((((Cottus_nozawae:0.00000332819398067269,Cottus_amblystomopsis:0.00498751607862064645)100:0.12017743715204591559,((Cottus_poecilopus_volki:0.06634284575281666763,Cottus_hangiongensis:0.12591585392479365257)97:0.05189271411628310071,(Cottus_reinii:0.08882340299916130499,(Cottus_pollux:0.08070713422220839040,(Cottus_kazika:0.25002483786180212944,Leptocottus_armatus:0.18182762211558711751)37:0.05808272366804675957)32:0.02864327993131059874)29:0.04084324396272603214)31:0.01629052189297928999)32:0.03685435234381101433,Cottus_poecilopus:0.05755543924534610445)27:0.03675984026090949042,Cottus_greenei:0.10022959735570048656)7:0.00795951301837406582,((Cottus_baileyi:0.04181825334381809867,(Cottus_paulus:0.07619853894670056138,Cottus_carolinae:0.02615259383096213419)42:0.00307406177265677222)97:0.02111750457847088452,(((Cottus_girardi:0.01725670289112722980,(Cottus_rhotheus:0.01467614942565364372,Cottus_caeruleomentum:0.02757398119774864473)31:0.00119354483001867222)42:0.00211780328112303991,((Cottus_bairdii_bairdii:0.01490791215721700737,Cottus_hypselurus:0.03164674177043795555)35:0.00200138043765635265,Cottus_cognatus:0.01755297119647420204)17:0.00163672577732488318)7:0.00000332819398067269,(((Cottus_extensus:0.00807961742515570826,Cottus_bairdii_semiscaber:0.00271426639346565319)63:0.00176323588208401123,((Cottus_hubbsi:0.00000332819398067269,Cottus_bendirei:0.00358056039231458055)100:0.01097693360917184786,Cottus_bairdii_punctulatus:0.00000332819398067269)45:0.00086611086773972926)94:0.01212765681462874001,(Cottus_bairdii:0.01090726646021559880,Cottus_bairdii_kumlieni:0.00000332819398067269)97:0.01476251187827784181)15:0.00370041964979022676)55:0.00679139708220969068)77:0.02401538003354261944)3:0.00481294114334722672)1:0.00295958686156064157,(((Cottus_aleuticus:0.04046918616986306533,((Cottus_marginatus:0.01791509134350695309,((Cottus_pitensis:0.03744179132998595033,Cottus_perplexus:0.00363991907191886944)14:0.00204996041071442165,Cottus_asper:0.00572418749574972376)4:0.00000332819398067269)29:0.00381041854434539112,Cottus_gulosus:0.00000332819398067269)51:0.01125804527978385532)15:0.00000332819398067269,(Cottus_princeps:0.01922637977872955484,(Cottus_asperrimus:0.02458680714993452532,(Cottus_klamathensis_macrops:0.00185631773392158877,Cottus_klamathensis_klamathensis:0.00183638972804888866)86:0.01603713435217247324)68:0.00507957557332077824)100:0.06110236566452990903)73:0.01664856827895950728,(Cottus_confusus:0.01615008366818803873,(Cottus_beldingii:0.00737367344144270690,Cottus_leiopomus:0.02588633608196488911)98:0.02060286333407219256)70:0.00704501076225080847)31:0.00622988749413365319)6:0.00966929480151301236,(((Comephorus_dybowskii:0.00000332819398067269,Comephorus_baicalensis:0.00000332819398067269)100:0.01285114900137784670,(Cyphocottus_eurystomus:0.01890554768927478826,(((Cottinella_boulengeri:0.00971532532040376810,((Asprocottus_korjakovi:0.00000332819398067269,Asprocottus_pulcher:0.00544755946853136500)99:0.00734131027258597916,(Asprocottus_herzensteini:0.01307966276840959521,Asprocottus_platycephalus:0.00188433186931295728)82:0.00192784779555141284)84:0.00579639452386109845)58:0.00119292173673623820,Abyssocottus_korotneffi:0.00836864022180825416)79:0.00748426620643757867,(Abyssocottus_gibbosus:0.00387872152414160589,(((Limnocottus_griseus:0.00000332819398067269,Limnocottus_godlewskii:0.00382133448830945755)65:0.00187905707324896891,(Limnocottus_pallidus:0.00761452636394835142,Limnocottus_bergianus:0.00763305208635736612)38:0.00000332819398067269)99:0.01599043599749581934,(Batrachocottus_nikolskii:0.00624167799070580748,Batrachocottus_multiradiatus:0.01090322314493894461)79:0.00847833194480265492)45:0.00430473626605087489)58:0.00300041799544853351)17:0.00000332819398067269)34:0.00188233809896944169)52:0.00441444156846059355,Cottus_kesslerii:0.08078345235424234938)3:0.00000332819398067269)1:0.00197460254297969831,(Procottus_jettelesii:0.00488121279527687496,Procottus_major:0.00629617370311252693)98:0.01571604195124231065)2:0.00123093535578110559)4:0.01136002375593514468)48:0.11656737771162666017,((((Myoxocephalus_polyacanthocephalus:0.02616579127119721473,Myoxocephalus_stelleri:0.01668422492957728764)88:0.09074521925247663878,(Myoxocephalus_octodecemspinosus:0.01828122962677486071,((Triglopsis_quadricornis:0.00406595241099850548,Myoxocephalus_thompsonii:0.00471028695749424724)83:0.04808424367627445184,Myoxocephalus_aenaeus:0.02025056590036238696)30:0.01815521393127763478)62:0.05263570197309612453)45:0.04793357554672537241,((Clinocottus_analis:0.96786858771651529398,Hemilepidotus_jordani:0.42804934530990951247)29:0.06829443466857486322,Malacocottus_zonurus:0.26684818538719812242)12:0.01341600298748260589)18:0.04897707734973257010,Scorpaenichthys_marmoratus:0.25785788025706191107)23:0.03877857622896725004)95:0.31745099706286589170)60:0.08445889001917457339,(Rypticus_saponaceus:0.04687403761663993390,Rypticus_bicolor:0.01207909883316867633)100:0.71795792967870197820)4:0.01343798956864448654)6:0.08210170686376654092,Notesthes_robusta:0.55212484824739538336)4:0.06338388979577663784,((((((((Premnas_biaculeatus:0.16887096606857918135,(Amphiprion_clarkii:0.02907496389930327649,((Amphiprion_akallopisos:0.02581932208694472097,Amphiprion_perideraion:0.00357764503583849272)97:0.02079087747734778557,(((Amphiprion_akindynos:0.03103124877793520670,((Amphiprion_omanensis:0.03052708851835204268,Amphiprion_nigripes:0.02925812100283049430)23:0.00000332819398067269,Amphiprion_sebae:0.03019504004085306337)9:0.00204467025947611221)1:0.00237971335008546050,(Amphiprion_melanopus:0.04545498410667082184,(Amphiprion_allardi:0.04534622809347881034,Amphiprion_chrysogaster:0.03755678426594577513)32:0.00377779375299879937)7:0.00000332819398067269)15:0.00698652361972110312,Amphiprion_chrysopterus:0.03828504676052520844)25:0.00652436022542064818)67:0.03878838357522963742)100:0.08146517242350355403)91:0.07297862542194814839,((Neoglyphidodon_polyacanthus:0.14307225320100688815,(Neoglyphidodon_melas:0.16691169994942806065,Neoglyphidodon_nigroris:0.08928058391087424772)72:0.01420675261505465956)69:0.05604569282608338338,((((Amblyglyphidodon_aureus:0.10508680951079467369,(Amblyglyphidodon_leucogaster:0.10668210805704105015,Amblyglyphidodon_curacao:0.05790870789145555891)99:0.06770840772174871458)97:0.16979829585699032779,Acanthochromis_polyacanthus:0.29505986092509561836)8:0.01759397729519909109,Hemiglyphidodon_plagiometopon:0.14959017330049315109)9:0.01405480437715663812,Neoglyphidodon_oxyodon:0.23650137056238323963)8:0.01815560607493517734)30:0.04799266969154668272)6:0.03579031065153052277,((((Pomacentrus_pavo:0.21596672838095723468,Pomacentrus_leptus:0.16413948042730017174)96:0.06488116297080070982,Pomacentrus_coelestis:0.17597098844482081392)90:0.03730938711043257916,(Pomacentrus_trilineatus:0.19027474819862880140,((Pomacentrus_grammorhynchus:0.08100535648108252162,(Pomacentrus_vaiuli:0.09276012099924077492,Pomacentrus_bankanensis:0.11138878444345297336)75:0.03607018988051811925)27:0.00842271273850982508,((Pomacentrus_australis:0.14371996802366374113,(((Pomacentrus_reidi:0.05913669497543815046,(Pomacentrus_chrysurus:0.02652942710091975298,(Pomacentrus_milleri:0.00465941137434572675,Pomacentrus_nagasakiensis:0.00630473282308363723)92:0.02179155164028399871)100:0.06606281107710028355)68:0.02691552194280920351,((Pomacentrus_brachialis:0.05675233316498144720,Pomacentrus_nigromanus:0.06567353060955312405)88:0.03642552514361516169,(Pomacentrus_lepidogenys:0.07477109198879877683,Pomacentrus_smithi:0.14807745992390830558)53:0.01979410457494881412)43:0.00929721317491745761)82:0.03395573310092969005,Pomacentrus_philippinus:0.12543340690082213640)98:0.07525742944785136779)64:0.04020206895679083459,(Pomacentrus_adelus:0.14045944652079153658,Pomacentrus_moluccensis:0.14587265539409202675)80:0.05319058184957542457)6:0.00638959288275985256)30:0.00913233828382767690)82:0.03550435382363233833)94:0.10186189013429382266,Chrysiptera_annulata:0.49654509583336225997)40:0.04401871398706719979)16:0.03490672361389199341,(((((Chrysiptera_rex:0.32902101357514990099,(Chrysiptera_taupou:0.06745566338988194521,Chrysiptera_cyanea:0.08585571807027343738)99:0.08487291255504204879)67:0.10602892524691658049,((Chrysiptera_talboti:0.24870756567849749974,Chrysiptera_rollandi:0.17803948228368993156)99:0.10969616908059337534,Chrysiptera_hemicyanea:0.21636609542644680282)28:0.02016171128578077634)92:0.09169101853322339513,(Dischistodus_melanotus:0.16513386644966115968,Pomachromis_fuscidorsalis:0.47312907240772222872)40:0.04451713991315308511)44:0.03672997531905176222,(Chrysiptera_leucopoma:0.18698144141896841375,Chrysiptera_glauca:0.23289751419239015928)99:0.21168438758864444038)22:0.02953608861253238446,(((Neopomacentrus_miryae:0.13372876349206339053,Neopomacentrus_sindensis:0.12553413598416615637)100:0.17057872350383876392,(Neopomacentrus_filamentosus:0.32459363761663156955,(Neopomacentrus_nemurus:0.24398872684507871833,Neopomacentrus_cyanomos:0.22078856671994448457)73:0.04707153753666691587)96:0.12278367045866968310)94:0.14545549627283893557,Chrysiptera_galba:0.29024767541182699970)36:0.03607436049939139605)4:0.01936616046238140085)88:0.16335935785591385039,(((Abudefduf_declivifrons:0.08242224136992233607,(Abudefduf_taurus:0.00864821334890455810,Abudefduf_concolor:0.01342389537312030541)88:0.03576160676706518354)99:0.15661386935118015717,(((((Abudefduf_abdominalis:0.02714968638304180587,((Abudefduf_lorenzi:0.02872640293973938858,Abudefduf_saxatilis:0.03040474114387842555)32:0.00451312464627168751,Abudefduf_troschelii:0.02624120482197932203)30:0.00148750545090658060)86:0.02420368693081905928,Abudefduf_sexfasciatus:0.02507309037208998656)100:0.06091414519541178685,((Abudefduf_margariteus:0.06925618462218872950,Abudefduf_sparoides:0.08032833279131956772)75:0.02076093530610669674,Abudefduf_bengalensis:0.08373044258710528320)39:0.01362187902610006128)58:0.01488926232910856712,Abudefduf_whitleyi:0.04417303211702351684)92:0.08643188709899039812,((Abudefduf_sordidus:0.04732300519057753424,Abudefduf_notatus:0.10909860261081520683)90:0.03111789423295711898,Abudefduf_septemfasciatus:0.06166250171581068307)100:0.09571064388239845788)77:0.06145973355672942201)96:0.10299746384608537975,((Chromis_nitida:0.19227778927891528427,(Chromis_chrysura:0.13047300171117123813,Chromis_flavomaculata:0.07671640454453802771)90:0.04435955846692387489)91:0.07077480406032458926,((((Chromis_multilineata:0.00172657575142461670,Chromis_cyanea:0.00000332819398067269)100:0.38682889571437445442,((Chromis_amboinensis:0.06358150516770511018,((((Chromis_iomelas:0.07046115392268714750,Chromis_margaritifer:0.11702497789745174017)41:0.01506729769764292640,Chromis_agilis:0.05807674310451808181)27:0.00298415143252993374,Chromis_atripes:0.09222832700032573827)49:0.01716021997784866748,Chromis_retrofasciata:0.09246614472215713976)54:0.02207641058390344066)100:0.25835328482400760075,((Dascyllus_aruanus:0.08055030035017425594,Dascyllus_melanurus:0.06416067417101777026)91:0.04770336311115795647,((Dascyllus_flavicaudus:0.06866768026662661839,Dascyllus_marginatus:0.08352586083552562823)52:0.03610071470439712299,((Dascyllus_albisella:0.00000332819398067269,(Dascyllus_strasburgi:0.00561861279339435655,Dascyllus_trimaculatus:0.00000332819398067269)35:0.00184262036855106673)63:0.02084460794158618860,(Dascyllus_reticulatus:0.01524253962591488876,Dascyllus_carneus:0.02629471787809282285)78:0.00726255781670733565)95:0.06825494323623199078)42:0.04049221325195849486)100:0.19567487277078010610)70:0.04734009099758341998)21:0.01960455595684707300,((Chromis_atripectoralis:0.08940479306556226025,Chromis_viridis:0.05519626596263380036)100:0.15343216861947081564,Chromis_atrilobata:0.39691119979560279818)9:0.00607594682539291855)43:0.02612633578884714092,((Chromis_weberi:0.03173182641763918482,Chromis_xanthopterygia:0.03128422262436392903)99:0.11163145339974557546,Chromis_chromis:0.10514271865980105714)69:0.02884501023845833373)35:0.01237968423569678518)97:0.14954332233372960581)51:0.03426648607304590144)47:0.06560887570680751235,((((Plectroglyphidodon_leucozonus:0.19142628484791132659,Plectroglyphidodon_dickii:0.17947367912185405170)61:0.06232461079604782739,(Stegastes_planifrons:0.47364993170089458863,Microspathodon_chrysurus:0.30395746927711203567)52:0.03681659052597141546)67:0.09267581931893474623,Parma_oligolepis:0.38591623002372504869)57:0.08434732476322887029,Mugil_cephalus:0.67452463985587740591)7:0.02911288886395776293)8:0.04371931820998174040,((Embiotoca_lateralis:0.40606963602401796587,((Amphilophus_rhytisma:0.25173554410129517223,((Cichla_temensis:0.00370990835431448708,(((Cichla_melaniae:0.01362987707724171507,Cichla_mirianae:0.01107584549808157334)97:0.01846891670609831018,(Cichla_vazzoleri:0.00535491099630960345,Cichla_thyrorus:0.00204790917200860624)100:0.02415465770760477984)38:0.00181830329564057307,((Cichla_jariina:0.02106917744402988027,Cichla_piquiti:0.02253987997437574900)52:0.00662809264274217495,((Cichla_intermedia:0.02316439772320122437,Cichla_orinocensis:0.02819341667421928349)91:0.01782589673171617109,(Cichla_kelberi:0.01758986278636318762,((Cichla_pleiozona:0.03540577754960696349,Cichla_ocellaris:0.01067144874246637835)28:0.00140925756233851486,Cichla_monoculus:0.01366399314694458078)36:0.00261962707066409461)94:0.04628370617208435456)80:0.05282972248582067099)29:0.00000332819398067269)54:0.01620942637524134394)96:0.13358071645792393700,Retroculus_xinguensis:0.38078317686667512154)63:0.08549993053744661675)71:0.14742245772534057213,(Oreochromis_niloticus:0.14413457532125423244,(((((Neolamprologus_modestus:0.03108844196782240346,(Julidochromis_ornatus:0.02802236595549852799,(Lepidiolamprologus_cunningtoni:0.01096207184099768674,Neolamprologus_tetracanthus:0.01373827022725057807)82:0.03027334670991962615)66:0.01304411551635015036)53:0.00767097219430720300,Variabilichromis_moorii:0.06074965522141028557)62:0.01795336815984580406,(Neolamprologus_tretocephalus:0.04208679719778709444,Neolamprologus_savoryi:0.07600317982937027095)35:0.01264290000612356031)14:0.01353499017743285682,(Lamprologus_ornatipinnis:0.03868918822809684632,Lamprologus_signatus:0.02385366799773453936)74:0.00777864894869907935)10:0.00000332819398067269,((Neolamprologus_multifasciatus:0.00572835218503708367,Neolamprologus_similis:0.00179349637621133173)100:0.02260439577942856271,((((Lamprologus_ocellatus:0.05436650661025171222,(Altolamprologus_calvus:0.00366283337193825526,Altolamprologus_compressiceps:0.00200405893550433109)77:0.00425074297815493211)22:0.00344023000931868717,(Lamprologus_callipterus:0.02854730499542782254,Neolamprologus_fasciatus:0.01037315415567680033)48:0.00415466768058076093)35:0.00586156768926930465,(Lepidiolamprologus_profundicola:0.01427920050381271087,(Lepidiolamprologus_attenuatus:0.01390656734783387964,Lepidiolamprologus_elongatus:0.00569789347924861290)92:0.00979532921839546857)66:0.00998493766110405793)28:0.00679169624072487998,(Lamprologus_lemairii:0.01829558746087657853,((Neolamprologus_brevis:0.00720050307270903803,Neolamprologus_calliurus:0.00919871466173520329)86:0.04256602846603232959,Neolamprologus_leloupi:0.01951964826768819508)19:0.00318730532786523635)30:0.00701466680174699618)7:0.00702748723231433683)23:0.00983073778995129273)27:0.09336705941428037614)95:0.22192173723717883993)49:0.09520406509941167772)22:0.03463600053551765812,(((((((Strongylura_timucu:0.02823109029928547176,Strongylura_senegalensis:0.04572189201227845612)100:0.23607869016444085752,Strongylura_notata:0.28217459738629102617)28:0.03794748987056950629,Belone_belone:0.27136322331265316787)29:0.01945860758978702754,(Strongylura_incisa:0.35074808986910666064,((Tylosurus_crocodilus_crocodilus:0.01482150516106641933,Tylosurus_crocodilus:0.02247063109106057360)95:0.06386560147466771586,(Ablennes_hians:0.22188225546888046269,((Tylosurus_acus_melanotus:0.00872204756471835901,Tylosurus_pacificus:0.01866733266785878739)95:0.03422877766040597486,((Tylosurus_acus_imperialis:0.00168736006545061167,Tylosurus_acus_acus:0.00000332819398067269)100:0.01614110331686186700,Tylosurus_acus_rafale:0.02647732719826892747)55:0.01257056755848703737)93:0.08071656295374488033)92:0.06732196938352101589)93:0.09129769943514147446)89:0.12485848126756542154)44:0.05460535190467491473,((Strongylura_fluviatilis:0.11329356807308649246,Strongylura_scapularis:0.22364714759280363010)96:0.08186149125205688382,(Strongylura_marina:0.15654140111379427625,(Strongylura_exilis:0.08065299465447967053,Strongylura_hubbsi:0.26842235356307569960)56:0.05029274480058175401)61:0.04881619056008883878)100:0.25787884837359881240)99:0.39813943608958979326,Hyporhamphus_unifasciatus:0.39701477858584860448)46:0.10744257811274429693,(((Rhadinocentrus_ornatus:0.84075081356671232946,(Melanotaenia_splendida:0.00000332819398067269,(Melanotaenia_splendida_splendida:0.00000332819398067269,(Melanotaenia_duboulayi:0.01271720060554023916,Melanotaenia_fluviatilis:0.03797747923939040482)58:0.07817858668954912049)6:0.00546954610799050764)78:0.21365631982827451574)100:0.62706011343577472417,(((Craterocephalus_marjoriae:0.07305617429033151033,(Craterocephalus_cuneiceps:0.10327226577903626803,((Craterocephalus_centralis:0.05682205327000023481,(Craterocephalus_fluviatilis:0.00989959612981524371,Craterocephalus_amniculus:0.01376824561068750591)100:0.03644597302140583500)85:0.03552901075228723227,Craterocephalus_munroi:0.14511490313890199055)15:0.00702582059938431595)54:0.03411625946645074209)98:0.20897564029911944172,(Craterocephalus_stramineus:0.48400463645168695370,(Craterocephalus_stercusmuscarum:0.00364052582341103069,(Craterocephalus_randi:0.02772540414888103807,Craterocephalus_stercusmuscarum_fulvus:0.01285595132985579113)59:0.00355313512965696118)79:0.06567924820222795224)74:0.10772153521655650210)96:0.24375625379090415379,(Gambusia_holbrooki:1.24531072284129606942,(Ambassis_marianus:0.64997657395338293007,((Ambassis_agassizii:0.47195179011267274216,Ambassis_jacksoniensis:0.44855554264139008147)79:0.13288852554441782372,Denariusa_bandata:0.53629885139694077889)39:0.10328542814626984203)73:0.06360402822713556725)22:0.09327572902891877182)25:0.05289309664189947163)8:0.05299725234982177735,(Pseudomugil_gertrudae:0.42717426217252801868,(Pseudomugil_tenellus:0.41373636045118022952,(Pseudomugil_signifer:0.28597112873625485374,Pseudomugil_mellis:0.23838075912504783083)100:0.29895414014158011584)99:0.19018069349154984038)50:0.10411253028230378626)6:0.05652762598461394394)9:0.08631258858593639227)2:0.00626309669265144153)10:0.10662543720959972993,((((Scophthalmus_maximus:1.75025331244152737575,(((Plagioscion_squamosissimus:0.27494860290535022562,(Seriphus_politus:0.22496320361067753679,((Stellifer_illecebrosus:0.49611913512302824314,(Bairdiella_ronchus:0.15494967185765456952,Bairdiella_armata:0.03282287934821389447)100:0.21563571720349186678)74:0.09218964921774273902,(Nebris_occidentalis:0.04567738103841952751,Nebris_microps:0.13994960814972687291)98:0.14056894824117086018)46:0.02550399066564059194)51:0.03442268346008245383)27:0.01592988124827500462,(((((Cynoscion_acoupa:0.02747920604385013787,(Cynoscion_xanthulus:0.00518258683667474976,Cynoscion_albus:0.00691337173594300594)100:0.01661069587666587008)95:0.06629652342634165252,Cynoscion_parvipinnis:0.10380450524034431914)84:0.04903820854333700713,(((Cynoscion_reticulatus:0.02929890046578892057,Cynoscion_nothus:0.05180807970966955917)96:0.06618001854810384721,(Cynoscion_jamaicensis:0.11529794578408407435,(Cynoscion_nebulosus:0.10336284271647172561,(Cynoscion_regalis:0.04454560144814988243,Cynoscion_arenarius:0.06755378994944749582)95:0.05576063233684786652)65:0.02638438861009755401)40:0.00739771623848537591)32:0.01070855676490277582,Cynoscion_othonopterus:0.06116663159197776450)62:0.03374403206878030803)52:0.03322373594143428199,(Cynoscion_virescens:0.18921025899023286021,(Cynoscion_microlepidotus:0.12817964320387134625,(((Cynoscion_leiarchus:0.01014606614618214150,Cynoscion_phoxocephalus:0.03867620652651341390)100:0.12084952667207872479,(Isopisthus_remifer:0.15903716274153084642,Cynoscion_analis:0.12937937442160807322)86:0.04191618845878919419)40:0.01730655204838250569,(Cynoscion_praedatorius:0.07428534184666997497,Cynoscion_squamipinnis:0.05466404760215286263)79:0.05176168014276372187)60:0.03263698623851965519)59:0.01235456221492135971)27:0.01518097540701097634)28:0.03445629124981126890,(Atractoscion_nobilis:0.07928736210809136931,Cynoscion_guatucupa:0.12833361587835562245)27:0.02851864839889303380)16:0.03380354954778400783)44:0.07298539349675003951,(Macrodon_mordax:0.03524407508571559405,Macrodon_ancylodon:0.01732108129507156646)100:0.49323392998864640235)91:0.16951925021685992911)7:0.10109422224891315145,(((Sicydium_punctatum:0.12212211007052758971,Sicydium_buscki:0.10017427866463425590)100:0.62994997197907243169,Stethojulis_bandanensis:0.61865930223450404934)16:0.16318524581101676030,Novaculichthys_taeniourus:0.60703468994653486668)1:0.09549176306889536892)0:0.07136096390988658222,(((Ostracion_meleagris:0.36010022587257406457,Zanclus_cornutus:0.29653317001502921268)37:0.04431683542824700617,((Forcipiger_flavissimus:0.92161718733832476147,Cirrhitichthys_oxycephalus:0.67466694167042828845)14:0.21230996079160030510,(Acanthurus_triostegus:0.10834839137744506354,(Ctenochaetus_marginatus:0.13270823396614619960,Acanthurus_nigricans:0.12197365237467605592)62:0.07715520299770936563)97:0.16865164005468519237)3:0.08214343134816526337)1:0.06960730969369774424,((((Heteropriacanthus_cruentatus:1.23592382800353517425,Mulloidichthys_vanicolensis:0.89046619061918974580)15:0.25710167610428924512,(Philypnodon_grandiceps:0.32165476854913549465,Philypnodon_macrostomus:0.27327046089091661107)75:0.21613567391218385660)9:0.07512999219462336564,((Gobiomorphus_australis:0.35791393656841080650,Gobiomorphus_coxii:0.11294883263506677595)60:0.10883211567832154487,((Hypseleotris_galii:0.07354698246656049543,Hypseleotris_compressa:0.06698590466703051305)99:0.21230576580002419318,((Mogurnda_mogurnda:0.01220059133699580474,Mogurnda_clivicola:0.01759009335207477320)95:0.02400439293989863401,(Mogurnda_adspersa:0.02881646588510024948,(Mogurnda_thermophila:0.01418814652559027140,Mogurnda_larapintae:0.02669837804557945171)66:0.01245443148475409642)44:0.00385757641375284854)100:0.47941033810476402222)39:0.06287950683628788784)43:0.11266159177904377930)22:0.18832996607166471725,(((Leiopotherapon_unicolor:0.47241377486172120292,Sectator_ocyurus:0.24734427177150966615)29:0.10443520746119815124,(((Scomber_australasicus:0.00000332819398067269,(Scomber_colias:0.00932159968482472109,Scomber_japonicus:0.01253119037144088087)96:0.02315179573498486396)100:0.33173777324085601537,((Sarda_orientalis:0.14173557964753968896,((Euthynnus_alletteratus:0.16574464638117031301,(Katsuwonus_pelamis:0.14586752720432896724,(Auxis_thazard:0.03267034783914959939,Auxis_rochei:0.05179218013080950922)99:0.10687635070172013529)34:0.03205994018322560341)97:0.10776271239989491935,(Thunnus_alalunga:0.05525743973841942824,(((Thunnus_tonggol:0.00766747148296688299,(Thunnus_atlanticus:0.01199424335652642462,Thunnus_albacares:0.00514736905874262247)41:0.00160952476968110637)42:0.00479850809122264570,Thunnus_thynnus:0.00559392380349268969)24:0.00215735635837705896,Thunnus_obesus:0.02832097950771820735)37:0.02462818787750525873)100:0.19791975804129594008)22:0.02936881082220798439)59:0.04528559455768837311,(((Scomberomorus_guttatus:0.19769166510021746186,Scomberomorus_cavalla:0.23566120315509100913)34:0.05389568923766716035,((Scomberomorus_sierra:0.03944232565650441363,(((Scomberomorus_maculatus:0.00555428744348825717,Scomberomorus_regalis:0.00173108289594825558)97:0.01991942156164689184,Scomberomorus_brasiliensis:0.00714074064986433034)100:0.04627382396352043570,Scomberomorus_concolor:0.03013719786607470599)49:0.01025618188990391928)81:0.03324621599635447372,Scomberomorus_tritor:0.12029371612576747474)100:0.10255384857982563496)27:0.03338437455327779996,Scomberomorus_semifasciatus:0.20989371004703552548)100:0.18389760283000369800)95:0.10066793747736313414)97:0.22363013108880053226,((Cantherhines_dumerilii:0.48540446544483140112,(Nannoperca_australis:0.20432404865981690367,Nannoperca_oxleyana:0.10394946029087721862)100:0.36099722700669895081)43:0.08350629863302588829,Diodon_holocanthus:0.71891410417424539414)22:0.03613275291474227907)11:0.07669068782320268007)5:0.01732366049858348814,((Polydactylus_sexfilis:1.07947733685389524183,(Calotomus_carolinus:0.78732562404352446350,(Scarus_ghobban:0.12766780490395368552,Scarus_rubroviolaceus:0.00000332819398067269)100:0.38430320927727268376)100:0.30553578471725506560)30:0.14141486633275524021,(Takifugu_fasciatus:0.43707520916423764357,(Arothron_meleagris:0.12930832307713127638,Lagocephalus_inermis:1.02650649213373346136)96:0.16961811781639418717)93:0.20431545725874808439)2:0.11625006274479576795)0:0.03498323529338834281)0:0.02755217829896725729)0:0.04444528439569166156)2:0.03712451702544834059,((Doryrhamphus_excisus:0.95220553077267888753,Channa_marulius:0.41544843298818118216)38:0.07921662695473737081,((Kryptolebias_ocellatus:0.03046670834488826238,Kryptolebias_marmoratus:0.01703804281202425663)100:1.43493931715501354951,(Ophisternon_aenigmaticum:0.79032685747165010159,Synbranchus_marmoratus:0.39473383389154442780)93:0.43051460942029656787)12:0.28303220128791406474)3:0.18114910063332059775)0:0.02653174050620385471)1:0.03131470927963130729)13:0.07988510396352331566)42:0.05512857797347917727)37:0.08949733958296025860):0.45000000000000001110,Polymixia_japonica:0.45000000000000001110);

Figure 2: ND1 tree

((Sargocentron_rubrum:0.35205726300541501095,(Beryx_splendens:0.23043045730674427407,(((Pampus_punctatissimus:0.15330261542279724485,(Pampus_cinereus:0.09797926603019145586,Pampus_chinensis:0.10518136681535529553)66:0.05166986340223710117)97:0.17099230501365181301,Thunnus_thynnus_thynnus:0.21478472569013701277)88:0.15279487905515348745,((((Cephalopholis_miniata:0.36539691911980376471,Pseudamia_gelatinosa:0.40119679722793216703)26:0.07947679807847218836,(Pempheris_vanicolensis:0.70617141654014059515,Pagrus_pagrus:0.38187318018181903856)14:0.10208726850196092606)0:0.04330242430591337011,(((((Anarrhichthys_ocellatus:0.09434668291921594063,(Anarhichas_orientalis:0.05670327882542228193,((Anarhichas_lupus:0.01548533219398257749,Anarhichas_minor:0.01623737795066263964)66:0.01025335056694436954,Anarhichas_denticulatus:0.00312582255661581843)72:0.02291181213206972336)85:0.06524767023666573629)99:0.29313133700591775810,(((Leptocottus_armatus:0.18321445706716224522,Icelinus_borealis:0.12431217694794380324)76:0.07447208511405142839,(((Enophrys_bison:0.22195503030547253376,((Ruscarius_creaseri:0.21529443936043099472,Ruscarius_meanyi:0.15135306731744338493)95:0.10849542356859506742,((((Clinocottus_analis:0.13502845737782420699,Leiocottus_hirundo:0.11583567647386899813)75:0.03365937439744035031,(Oligocottus_maculosus:0.15298624116860989886,(Oligocottus_rimensis:0.24887486549278317605,((Orthonopias_triacis:0.16719675663691427325,Oligocottus_rubellio:0.08462201547080606956)39:0.02783689030595017100,Oligocottus_snyderi:0.09771888141200563149)24:0.01733509189377033938)4:0.00713934318391960289)53:0.03004545295298883742)60:0.02375312630359979749,(Clinocottus_globiceps:0.03641164279456698960,(Clinocottus_embryum:0.09335799191751291415,Clinocottus_recalvus:0.11568828682434532940)85:0.02841077031056863170)100:0.15596698610364509441)87:0.07174047912864842114,((Artedius_harringtoni:0.12857689222279955477,((Artedius_corallinus:0.02228069392842052521,Artedius_lateralis:0.06014531360942215049)94:0.06659629895291366286,(Artedius_fenestralis:0.05190028203229098136,Artedius_notospilotus:0.07448937669932444161)94:0.04747417988396284566)34:0.01759012791598288383)31:0.01823782837386800706,Clinocottus_acuticeps:0.34787174165684153770)26:0.03213921833051225363)44:0.00928290494597623465)86:0.09105678634112042424)26:0.03989756202859745526,Chitonotus_pugetensis:0.20178634318002472425)17:0.02415993253234598875,(Jordania_zonope:0.19286559948204348713,(Hemilepidotus_hemilepidotus:0.22471266474867046536,Scorpaenichthys_marmoratus:0.19755444998668555967)71:0.09762865423150451916)31:0.07676418671318939457)11:0.02136158062956227191)33:0.04904504595450815546,Stellerina_xyosterna:0.23471038434083016377)54:0.07612310926251686372)38:0.05489820319508142282,(Trachurus_trachurus:0.41656198421895052064,((Paralichthys_lethostigma:0.15977464531675386605,Paralichthys_californicus:0.08385073673946709383)100:0.21642455803408408577,(((((((Cleisthenes_herzensteini:0.05940436586654267159,Dexistes_rikuzenius:0.09518862150349488571)95:0.01549126076506142369,((Limanda_sakhalinensis:0.04660913612035350734,Limanda_aspera:0.03022796837229782541)99:0.02826769587049553223,(Hippoglossoides_elassodon:0.00847137619750472012,Hippoglossoides_robustus:0.00304219319143671186)100:0.03981293139101808182)43:0.00386365359682109459)100:0.06434150209962784250,(Pseudopleuronectes_americanus:0.09641807039092410714,(((Parophrys_vetulus:0.04322965804394544170,(Isopsetta_isolepis:0.06283304262411332219,Lepidopsetta_bilineata:0.05111406532458966784)88:0.01391205289417068655)86:0.02365580538131061006,Lepidopsetta_polyxystra:0.03752988197956552002)95:0.02181847414104838914,(Limanda_proboscidea:0.06733002756244339804,(Pleuronectes_quadrituberculatus:0.05156846085772399946,(Liopsetta_pinnifasciata:0.03647403494251449940,Platichthys_stellatus:0.03568391468222089496)96:0.02822522445822008008)77:0.01127672628296474792)93:0.02776429672865205617)84:0.01749960782391905834)94:0.02692081788271239937)78:0.03301216615334340299,(((Clidoderma_asperrimum:0.10920562163123183341,(Verasper_variegatus:0.03600786032432590905,Verasper_moseri:0.02771693753132635496)100:0.07595622673083599063)35:0.01429597788175555710,(Eopsetta_jordani:0.12930632176479336937,(Reinhardtius_hippoglossoides:0.06583224952453878287,Hippoglossus_stenolepis:0.06091568419915629334)47:0.01511046973123051848)17:0.00286202020362211120)16:0.00518302719123403793,Lyopsetta_exilis:0.14351369969975680263)46:0.01736722993621230005)63:0.02533468348558019778,((Embassichthys_bathybius:0.04455387556007625594,Microstomus_pacificus:0.10970549472933079083)95:0.04211749996077782804,Glyptocephalus_zachirus:0.09559108635140631249)84:0.03690376215672091370)76:0.07586632936602374921,(Pleuronichthys_coenosus:0.15142552356063307406,Pleuronichthys_decurrens:0.18577543952401462390)100:0.29653317584720317868)80:0.06906855133884033660,(Atheresthes_stomias:0.00255913616070628600,Atheresthes_evermanni:0.01511493715724611386)100:0.24998955823652960206)36:0.03304742648052450688)98:0.19792939530358263922)37:0.07366408333432546141)14:0.05031876296227604656,(((Symphurus_orientalis:0.52196036971398651616,(Takifugu_fasciatus:0.08576651820013864169,Takifugu_alboplumbeus:0.00000271430764945718)100:0.52117416185434306541)32:0.16534796163390907964,(Lagocephalus_inermis:0.23585681159542834506,Lagocephalus_gloveri:0.33112474867591945582)100:0.41870561228140934507)18:0.09642697692398186082,Cheimarrichthys_fosteri:0.37877260991591588102)7:0.02878686468960406364)2:0.01463444375279507326,((((Orestias_agassizii:0.12047170348489821823,(Orestias_imarpe:0.00361263546097905777,Orestias_gilsoni:0.00628181128439346623)99:0.06605659928979180917)100:0.56787644725477370855,(((Anableps_anableps:0.38667231281264602405,((Jenynsia_lineata:0.33083236637110985390,Oxyzygonectes_dovii:0.37505896696519291922)73:0.10196934803085250243,Fluviphylax_pygmaeus:0.70263932890303582024)17:0.00797813389384087641)43:0.10582482613522350978,(Jordanella_floridae:0.11894634319184232973,(Cyprinodon_variegatus:0.20038058296048011364,Floridichthys_carpio:0.53830224176948748838)58:0.14981245766515990936)98:0.27825757222180130812)16:0.04628736699700136681,(((Valencia_letourneuxi:0.16864286974610390168,Valencia_hispanica:0.13038892094268417221)100:0.33627071643875300344,((Aphanius_iberus:0.20348857405775777618,(((Aphanius_persicus:0.04380938828699761783,Aphanius_sophiae:0.02903932492718165267)100:0.03161433819883671670,(Aphanius_vladykovi:0.04480319092287857186,Aphanius_isfahani:0.06614481689551100774)63:0.00698981330134700735)100:0.22301629051954435790,((Aphanius_danfordii:0.10392368507675760203,((Aphanius_anatoliae_transgrediens:0.06333481598732713935,((Aphanius_anatoliae_sureyanus:0.01441810393838461289,Aphanius_anatoliae_splendens:0.01091155436772953125)96:0.01200291514366961208,Aphanius_anatoliae_anatoliae:0.04926892142047267081)91:0.01237390636438973242)95:0.03266192169775993664,Aphanius_villwocki:0.10505509964118317501)82:0.01841368858216922699)94:0.06981384480776757495,(Aphanius_asquamatus:0.33366895640463051231,Aphanius_fasciatus:0.18393748501959220065)62:0.03119655659934369649)84:0.05257315026507675820)75:0.02418802933512729722)98:0.11150441633313466772,((Aphanius_mento:0.25856390992110400706,((Aphanius_dispar_richardsoni:0.05223361108983718554,(Aphanius_ginaonis:0.03429193577934656206,Aphanius_dispar_dispar:0.00917988070801099669)96:0.02982038283174141130)99:0.07456967803573893994,Aphanius_sirhani:0.08577130542733411855)91:0.14332148290673615842)99:0.17881964367711841724,Aphanius_apodus:0.32818900323793120943)52:0.06311292359028332355)99:0.22757034104529361151)68:0.06742624618889177246,((((((Priapella_olmecae:0.04070656660202425642,(Priapella_intermedia:0.03343932447541768382,Priapella_compressa:0.03726398819758325320)73:0.00640412495786944758)100:0.16591156543021831626,(((Scolichthys_greenwayi:0.29414513854943319382,(Xiphophorus_hellerii:0.03766174232131828253,(Xiphophorus_maculatus:0.03839320232970981628,(Xiphophorus_clemenciae:0.02334820709136136568,(Xiphophorus_evelynae:0.01070616124472098356,Xiphophorus_xiphidium:0.01620870837789981089)95:0.00634263469199246050)96:0.01631582094552270698)59:0.00661239612750647889)98:0.11355879410562698395)3:0.00000271430764945718,((Gambusia_affinis:0.09893172909148494065,(Gambusia_wrayi:0.08438924470867993632,(Gambusia_atrora:0.08809502300676122499,Gambusia_vittata:0.09389726695009893709)96:0.03031759600311715805)92:0.02343147132818457559)95:0.08005508660187882208,Heterophallus_milleri:0.28317717727361274571)96:0.20056187304115921655)13:0.00865752675190584013,(Carlhubbsia_stuarti:0.18678340901149362296,(Heterandria_bimaculata:0.00416876277967207203,Heterandria_jonesi:0.02007214521458922443)100:0.21794163257092208363)54:0.03754322135359238616)65:0.03220469641964406049)69:0.04728806836817560366,((Heterandria_formosa:0.26020512802009787556,((Girardinus_metallicus:0.05509947814778073244,Girardinus_creolus:0.07203025703284561543)100:0.09581770931960113813,Quintana_atrizona:0.34175819569554288790)100:0.14514146343607511880)37:0.04784749917585593565,((Poeciliopsis_fasciata:0.17054454001379557027,Poeciliopsis_hnilickai:0.22848879039549116476)99:0.18208207645409371644,((((Phallichthys_amates:0.17654847778349957999,Phallichthys_tico:0.08711422090257676942)54:0.03716949208114452702,(Brachyrhaphis_hartwegi:0.26819811124452147544,(Brachyrhaphis_terrabensis:0.05387962040690645604,Brachyrhaphis_rhabdophora:0.07525515459435649923)74:0.04528735913645715733)46:0.02873600584182892451)52:0.01072899673839831057,(Alfaro_cultratus:0.36198245098919085150,Priapichthys_annectens:0.19308142719550583366)33:0.03387824695195867930)34:0.03286195861953319919,Pseudopoecilia_festae:0.35936160321605947754)12:0.00909096039923924099)8:0.02588842607751322758)10:0.01572439315052279185)12:0.03078448862774853592,(Neoheterandria_tridentiger:0.37177263573686991638,(Cnesterodon_decemmaculatus:0.24507363845245661027,(((Poecilia_latipunctata:0.10617362631397342376,Poecilia_caucana:0.09153576411703896742)92:0.05538266839574489120,(Limia_melanogaster:0.06229765528716983691,(Pamphorichthys_hollandi:0.28126558145766228369,(Limia_tridens:0.04204225051314971345,Limia_dominicensis:0.02965063332729553705)89:0.02682635562186380662)45:0.01691320092256173163)59:0.02387621476999409603)64:0.04514742723298172805,(Micropoecilia_picta:0.24259497485324740818,Poecilia_reticulata:0.18171589986994393651)100:0.10961331939461145535)97:0.09057202028508511138)89:0.06679389362804062646)31:0.03884280098402115994)24:0.07231710790917561527,Phalloceros_caudimaculatus:0.32924847487275848978)42:0.11310339605533700769,(Tomeurus_gracilis:0.90057141743608482010,Xenodexia_ctenolepis:0.55551733187066709707)42:0.08527808261089576958)96:0.18947341233942846528)31:0.04039323766250206560)8:0.04614726941356239037)66:0.21410509968264282432,(Kurtus_gulliveri:0.43875998398905563525,Milyeringa_veritas:0.63350341924517006298)29:0.10394305747193716138)5:0.03713079281609963506,((((((Butis_butis:0.56237779322548331340,(Bostrychus_sinensis:0.25624162834205488348,(((Oxyeleotris_selheimi:0.07874416935836450748,Oxyeleotris_lineolata:0.10582603765086574799)99:0.10299653846672136848,(Oxyeleotris_nullipora:0.53747902464638552811,Oxyeleotris_marmorata:0.13520146917028927347)32:0.05167569075482653096)31:0.05644431100793927125,(Kribia_nana:0.79295260687849722370,Ophiocara_porocephala:0.15736465277539735119)24:0.06713486538146458404)12:0.02349974036932511376)59:0.12945292009715958503)25:0.07462927510670891440,(((Ratsirakia_legendrei:0.27185843596198328020,((Tateurndina_ocellicauda:0.53033982260364820327,(Mogurnda_mogurnda:0.03253648661672219056,Mogurnda_adspersa:0.00979685597330030682)100:0.21507088323849776756)70:0.06340860486078989111,Ophieleotris_aporos:0.18245033088597117921)59:0.05735445296981040580)80:0.13095615268356447514,((((Calumia_godeffroyi:0.26491674547402077611,(Philypnodon_grandiceps:0.21147945820337579748,(Odontobutis_obscura:0.24374742604258947676,Odontobutis_potamophila:0.66690617158709453172)70:0.24269448527229370294)47:0.19036284115770457648)21:0.08728841541772806856,((Gobiomorphus_breviceps:0.19829391447228822409,Gobiomorphus_hubbsi:0.09804637143918298881)99:0.11364289808497936296,Gobiomorphus_australis:0.28922975446099380425)68:0.05954264726296241289)7:0.04126572376342412668,(((((Dormitator_latifrons:0.07822586346831916781,Dormitator_maculatus:0.13717198760949816938)100:0.17351673828133654109,Guavina_micropus:0.23215439299322512823)78:0.11648558738658416900,((Gobiomorus_dormitor:0.22460328300060394824,Hemieleotris_latifasciatus:0.21962218886849452226)52:0.04317177299836980064,Gobiomorus_maculatus:0.35617836584556444857)35:0.02810528339919829074)53:0.06803649618409554622,Microphilypnus_ternetzi:0.44138124577500847945)24:0.02412090173146303959,(((Eleotris_pisonis:0.01005042359037863070,Eleotris_picta:0.00323456605318929794)100:0.28046255179009504932,(Eleotris_fusca:0.15348973879086488803,Eleotris_sandwicensis:0.34111137507415273395)37:0.03267995693472791618)45:0.02914396541515639621,((Eleotris_smaragdus:0.17198851318149951939,Erotelis_armiger:0.29156316223646272512)100:0.40410251196055063660,Eleotris_amblyopsis:0.13582720254531369108)56:0.06121653271307752470)78:0.07184397995235634882)17:0.01745200748760461842)5:0.03354860031375983542,(Hypseleotris_aurea:0.08602920564860828956,((Coryphaena_hippurus:3.26948320854831742110,Hypseleotris_klunzingeri:0.00000271430764945718)13:0.11227827206646169567,Hypseleotris_compressa:0.07446308890640639111)9:0.02088931228414457608)13:0.30115937272059800645)2:0.02210990238562953250)5:0.02880428212032380350,(Leptophilypnus_fluviatilis:0.28306586810887540695,Leptophilypnus_panamensis:0.27286579980841801918)96:0.29663239779283179232)12:0.04032363745490873119)3:0.01526426304964306237,Perccottus_glenii:0.64848549923880893786)6:0.03464934088194924350,((((Periophthalmus_barbarus:0.27910581977761866890,((Scartelaos_histophorus:0.19892798192632987764,Odontamblyopus_rubicundus:0.31424377448119472644)79:0.08677379815187176437,Pseudapocryptes_elongatus:0.26045119147240336499)66:0.07415039943398074596)93:0.09930771949494456019,((Mugilogobius_rivulus:0.36337943019031615499,((Pomatoschistus_minutus:0.46954006093879302464,Acanthogobius_flavimanus:0.49603723948402972388)15:0.13074970006590372340,(Gillichthys_mirabilis:0.29119525357521192088,(Typhlogobius_californiensis:0.22270294552173316993,Chaenogobius_annularis:0.29847852394804108567)67:0.04638159250276494699)94:0.15826302693875393679)1:0.01078341480918098248)4:0.06344071597667164109,(Awaous_guamensis:0.61174867305517643956,Pandaka_lidwilli:0.82218938841306787868)19:0.11722656021377551860)0:0.01661818414351585310)0:0.05295274733677098750,(Stenogobius_hawaiiensis:0.29619797858123186884,((((Gnatholepis_thompsoni:0.03532127965316309015,Gnatholepis_scapulostigma:0.06021148324620124309)100:0.15561339881813349151,Gnatholepis_cauerensis:0.21997932560716496053)44:0.05495686559339541671,(Ctenogobius_saepepallens:0.36451386102781652143,Evorthodus_minutus:0.40883597161884693838)47:0.08372185923461021584)69:0.10275422346340000457,(Stiphodon_elegans:0.17773997625255802402,Sicyopterus_lagocephalus:0.21653325620013033626)85:0.11955755624692879224)39:0.05381364438492150515)60:0.09470833684852815459)1:0.07267076611365824723,(((Callogobius_bifasciatus:0.21883403078121574370,Callogobius_sclateri:0.26266520031651591216)77:0.18664580766946786694,((Trimmatom_eviotops:0.51245760594172762303,((Lythrypnus_zebra:0.47716945076477357279,Lythrypnus_dalli:0.29461823935285946785)100:0.57857508646547284314,((Trimma_flammeum:0.46654595100671641950,Trimma_caesiura:0.33690860826426638486)57:0.09061272788036468795,(Priolepis_eugenius:0.58035441056841996499,(Priolepis_cincta:0.31639318862862014781,(Priolepis_hipoliti:0.00222974209169258045,Lythrypnus_elasson:0.00104747620412379500)100:0.31513487770010811806)63:0.04245182732718032204)99:0.20666331789761402393)27:0.03195262564796855914)29:0.04782416724343117181)33:0.06912800234218369200,(((Schindleria_praematura:0.00332513695421201650,Schindleria_pietschmanni:0.01162791857297094771)100:0.53884059735419120862,Nes_longus:0.35600350009661441142)14:0.14011314338708086291,(Caffrogobius_saldanha:0.37384558699565650919,((Zosterisessor_ophiocephalus:0.40737512868813108291,Gobius_niger:0.32740935466718146252)89:0.14393972454073045153,(Neogobius_melanostomus:0.19596063123697471386,(Benthophilus_abdurahmanovi:0.25041403261332501051,((Neogobius_gymnotrachelus:0.12107276610087298885,Ponticola_cephalargoides:0.10498429604824699446)99:0.08196587235441189778,(Proterorhinus_semilunaris:0.15375254495167775537,Mesogobius_batrachocephalus:0.11715127836271692352)88:0.05266137626211311179)100:0.13010325739048317351)35:0.03253524514183126631)95:0.15963851361557548914)40:0.04882850528131822687)43:0.05602864463528169775)2:0.05637657441009882170)0:0.03707606089227927226)0:0.00418839900505522424,(((Asterropteryx_ensifera:0.11042449495250414304,Asterropteryx_semipunctata:0.17484882737626766813)100:0.17609965058333268417,(((Amblyeleotris_periophthalma:0.18867344931216389892,Amblyeleotris_gymnocephala:0.18724091630988048962)52:0.04883376397267830821,(Amblyeleotris_wheeleri:0.13753228817352894375,((Amblyeleotris_fasciata:0.13401685889574138644,Amblyeleotris_yanoi:0.14798998360024051557)35:0.03339444713115465729,(Amblyeleotris_steinitzi:0.07944262423742164125,Amblyeleotris_guttata:0.05976498068553269538)99:0.08142980384606514355)49:0.02693720567129385610)76:0.06140344258078180711)99:0.15846761283057017589,(((Ctenogobiops_feroculus:0.18609238446660625321,Ctenogobiops_aurocingulus:0.09231653715945144223)84:0.04673168064514725273,((Ctenogobiops_mitodes:0.17179712571207475524,(Ctenogobiops_formosa:0.14155610370249971375,(Ctenogobiops_crocineus:0.05789433963524502624,Ctenogobiops_maculosus:0.04277138285540695029)98:0.05360850469861768580)62:0.03264854605458204079)38:0.02694428398601406149,Ctenogobiops_tongaensis:0.17410943298500258791)61:0.03461053646423726399)91:0.11810072378117551783,(Tomiyamichthys_lanceolatus:0.09967178029155084051,Vanderhorstia_ornatissima:0.18298857351101041613)100:0.28558239602435403492)70:0.07603173229763111884)16:0.03353303105420053776)34:0.07954598992889586051,((((Oplopomus_oplopomus:0.32856018030921724238,(((Exyrias_belissimus:0.20558361240069750187,(Istigobius_rigilius:0.04540527624441707821,Istigobius_decoratus:0.01178506221953647647)100:0.16359836830612001224)97:0.11588389886521398287,Cabillus_tongarevae:0.34119416959698750658)67:0.04827319740080872335,Arenigobius_bifrenatus:0.34229866925619739648)12:0.03533757935991382187)10:0.03773119435862953147,((Favonigobius_melanobranchus:0.21696923131367135129,(Favonigobius_exquisitus:0.00000271430764945718,Afurcagobius_tamarensis:0.01881671962554644015)100:0.36026317594395818045)68:0.10609288609990882857,Cryptocentrus_cinctus:0.28527553227627955312)15:0.04566914369759223630)0:0.01682114127139730173,((Mahidolia_mystacina:0.36203858102241198713,Acentrogobius_pflaumii:0.34278030052903224467)30:0.08967296446437909851,(Tomiyamichthys_oni:0.28265539905973890988,(((Cryptocentrus_albidorsus:0.18869942208000037298,Cryptocentrus_nigroocellatus:0.13815796427841806060)51:0.04219074766173042113,(Cryptocentrus_inexplicatus:0.22378852847717978225,(Cryptocentrus_lutheri:0.15040555335629088307,Cryptocentrus_leptocephalus:0.17110481981957917363)40:0.03763065717840733543)43:0.04441233311732951244)27:0.05510762505066347378,(Cryptocentroides_cristatus:0.29846356074491908750,Stonogobiops_xanthorhinica:0.33257121202346762168)10:0.04468321595202430496)13:0.06720529671480462985)10:0.05948950860422611703)5:0.05024532060666126593)15:0.04951823135009909838,((((Nemateleotris_magnifica:0.38361026184457180932,((Bathygobius_cocosensis:0.25999259010087016897,(Bathygobius_curacao:0.31337025303186005765,Bathygobius_soporator:0.20160244734351029372)84:0.03302370089187177393)94:0.17343361560875475424,(Eviota_afelei:0.51703681325002170954,(Microgobius_microlepis:0.57258160699385129977,(Coryogalops_anomolus:0.90953324647303035455,(((Risor_ruber:0.09281451026719290953,(Kraemeria_bryani:1.08318377946740884710,Kraemeria_cunicularia:0.08537570154479665052)57:0.28809832841158133920)59:0.40370055764611079141,(Ophiogobius_jenynsi:0.82654307331328491504,(Elacatinus_macrodon:0.45933934530757625225,((Elacatinus_horsti:0.00525804977485514607,Elacatinus_evelynae:0.00000271430764945718)100:0.12906533717851842447,Elacatinus_oceanops:0.15260485494447070809)100:0.30733201422865125130)37:0.00634370241295537703)61:0.13828111822971711486)22:0.07636210615377980737,(Gobiosoma_chiquita:0.30476997282195883443,Barbulifer_ceuthoecus:0.60301676830966866039)41:0.07793654895466485544)24:0.03951032674227338004)14:0.06072555032599012692)4:0.03560871850941515343)1:0.02432956995402274156)0:0.02976829017532042418)0:0.02479862938847778378,(Paragobiodon_modestus:0.30540972533257354504,(Gobiodon_quinquestrigatus:0.25646474339814601118,Gobiodon_histrio:0.07021338303742606646)100:0.20310494251057018000)96:0.18857827307600380506)0:0.01576291272311525216,((((Lophogobius_cyprinoides:0.37400790541439443704,(Coryphopterus_hyalinus:0.30683475082890954155,(Coryphopterus_personatus:0.03275445591835533105,Coryphopterus_dicrus:0.10328700185516173637)78:0.25448469645786797599)88:0.11494347175533635441)63:0.22847914481317568858,(Rhinogobiops_nicholsii:0.00324582194425317334,Fusigobius_neophytus:0.00000271430764945718)100:0.46970345331794788324)63:0.10010250058192374234,(Fusigobius_signipinnis:0.00000271430764945718,Fusigobius_duospilus:0.01416076272795010506)100:0.82792635228293653959)11:0.05892729273211313318,((Gunnellichthys_monostigma:0.31621077051208185127,(Ptereleotris_zebra:0.30765307191099838802,(Ptereleotris_monoptera:0.01148224921772460563,Ptereleotris_microlepis:0.01128313428164490033)100:0.20070313213251064766)48:0.00003902226761773356)34:0.09171163421067496380,(Cerdale_floridana:0.36780812224937509125,((Microdesmus_dipus:0.18371085607681716945,((Eucyclogobius_newberryi:0.50639923540922660017,Microdesmus_longipinnis:0.03864669464278212146)61:0.13944233709909753527,Microdesmus_carri:0.20452510567328063829)34:0.04912859735658054589)58:0.07789551215579254562,Microdesmus_bahianus:0.20717707197355064719)40:0.05690399810234515049)60:0.09908916096047849786)11:0.05907053679406162883)4:0.04753731358215416286)0:0.03419617844097577047,(((Bryaninops_yongei:0.61304500355531721389,Psammogobius_biocellatus:0.28585035007928377704)29:0.12374723710487431361,(Amblygobius_phalaena:0.31379840650819096348,Amblygobius_nocturnus:0.28711802305033057303)83:0.11837424956261304032)2:0.02945420031022879734,(Valenciennea_strigata:0.24401850814900033493,((Valenciennea_longipinnis:0.25862649657351721721,Valenciennea_puellaris:0.26054210302295371937)58:0.04550513255394795370,Signigobius_biocellatus:0.39353660628061243321)47:0.05719092770766343037)30:0.06552548705566738074)0:0.02821588302388243832)0:0.02788352698502986199)0:0.02965258030865776037)0:0.01514479574526596088)5:0.05723922732289875304)6:0.02696424122423628916)12:0.05500984079788132086,((Apogon_aurolineatus:0.45292331953924558352,((Astrapogon_puncticulatus:0.33254706796859828666,Apogon_maculatus:0.30425841973911621485)34:0.04946700378744890020,(Phaeoptyx_conklini:0.00000271430764945718,Apogon_quadrisquamatus:0.02893839531194400752)100:0.35841899492450346454)19:0.03676985743581547006)81:0.08062690689541400502,(((((Sphaeramia_orbicularis:0.27135997990844801242,Apogon_doederleini:0.18314327233275484730)6:0.02646090217528266481,((Archamia_biguttata:0.05459296967279578944,(Archamia_fucata:0.00000271430764945718,Apogon_flagelliferus:0.00527815994799414167)90:0.05002001104182467184)100:0.22419273895326546908,Pterapogon_kauderni:0.29034278733584051313)37:0.06293872098656505543)2:0.04888962953575778231,((Apogon_cookii:0.32889559942561352734,((Apogon_angustatus:0.00049559231662316410,(Gymnapogon_urospilotus:0.00000271430764945718,Cercamia_eremia:0.37903155849376024555)53:0.05203725036418842265)96:0.23885798376667483067,Apogon_nigrofasciatus:0.19501463251035630608)53:0.04930469737497199245)49:0.06978178833006369597,(Apogon_holotaenia:0.37931453515486418526,(Cheilodipterus_isostigmus:0.23208447988844499443,(Cheilodipterus_artus:0.09463652009460503389,Cheilodipterus_macrodon:0.13048847976249419722)97:0.05624932415703694927)90:0.10382028802914917975)20:0.01760565401960291487)12:0.01482541958383235339)3:0.04506329139350726681,(Zoramia_fragilis:0.30683566684773899702,Apogon_lateralis:0.43583337406332695041)21:0.05780428870550212428)1:0.01412151923640182251,(((Fowleria_aurita:0.12914273601250156331,Fowleria_isostigma:0.09971352059044299998)100:0.38075411535781089611,(Apogon_trimaculatus:0.23738614075717726082,((Rhabdamia_cypselura:0.13252568672376197068,(Apogon_kallopterus:0.17747450432373218510,(Paraplagusia_japonica:2.95430357162452450481,Cynoglossus_sinicus:0.47723014668102120561)100:1.45609472437288611779)23:0.03526067394654056747)38:0.30987464491471516936,Apogon_exostigma:0.26834951217823355130)13:0.05365713296115206393)13:0.06735042457233784974)5:0.03538885650117886555,Glossamia_aprion:0.44674822930820390354)0:0.01635833217165245934)9:0.05707959832434023051)49:0.20198252500448557467)4:0.02449914582319830808,Gobiopterus_semivestitus:0.66837416819497297293)4:0.01626648613486052566)4:0.05216019301157592825)0:0.03103671451093989453)8:0.03536998037048388194,((Gazza_squamiventralis:0.45765562693976713504,Leiognathus_equulus:0.21053678076538129282)99:0.40168576484297729978,(Lepomis_macrochirus:0.21267219291919997604,((Lepomis_gibbosus:0.18277456808130698729,Lepomis_microlophus:0.16553873263304519359)70:0.00899295272101921679,Lepomis_megalotis:0.17503445899515618356)98:0.11469576010893912932)99:0.20463981115421894796)35:0.04288853311019440173)27:0.07880599803646294588)69:0.14058906189797651387)49:0.06270284860879624111):0.45000000000000001110,Polymixia_japonica:0.45000000000000001110);

Figure 2: ND2 tree

((Sargocentron_rubrum:0.37934903335399317026,(Beryx_splendens:0.29881227986316544198,((((((((((Phtheirichthys_lineatus:0.04665683396624616119,(Echeneis_neucratoides:0.01002995599708429719,Echeneis_naucrates:0.00000176267459066039)100:0.07829559879248801368)100:0.21860093190142046438,(Remora_brachyptera:0.22371094991606943481,(Remora_osteochir:0.18760264560466013983,(Remorina_albescens:0.19854337628829027418,(Remora_australis:0.18701507336819306326,Remora_remora:0.16094742878456902435)41:0.04184734331226409171)20:0.02192572203941375170)12:0.02099929239894656949)29:0.05900181588424279616)100:0.37978282383250711041,(Rachycentron_canadum:0.40384629893288453983,(Coryphaena_hippurus:0.10977982971679517421,Coryphaena_equiselis:0.15663087975819972586)100:0.53786671428984567100)100:0.43332286304703349078)38:0.10025419803891259807,Kurtus_gulliveri:0.51687309609369547214)8:0.03602415904892032616,(Chrysiptera_cyanea:0.43428972532824977604,(((Odontobutis_obscura:0.26971382928031245596,Odontobutis_potamophila:0.26135104763964195351)99:0.34860561967016340246,(Perccottus_glenii:0.58724007206230299794,((Rhyacichthys_aspro:0.20489240347785514507,Protogobius_attiti:0.18679452267023721745)99:0.10764582205591623210,Micropercops_swinhonis:0.65115125814167340579)39:0.02499562474816912835)29:0.05474371515157148982)53:0.10966171558061507674,(((Milyeringa_brooksi:0.01449072597912865260,Milyeringa_veritas:0.02774962101584360283)100:0.59689498852412614660,(((((((Dormitator_latifrons:0.09789470184311652168,Dormitator_maculatus:0.06608111733384894326)100:0.05955431253441657163,Guavina_micropus:0.18394131032356214606)100:0.12453140062211198735,(Microphilypnus_ternetzi:0.34285268428715415245,(Hemieleotris_latifasciatus:0.14432533887006934736,(Gobiomorus_maculatus:0.31027261110663817423,Gobiomorus_dormitor:0.18809994252272077553)74:0.04113583331520920261)98:0.07846233246748766932)66:0.02014073217566304114)77:0.03915876528057930100,Eleotris_oxycephala:0.24906002914267239001)35:0.01754576374115762033,((Calumia_godeffroyi:0.25799289292472532553,(((Eleotris_acanthopoma:0.00030204668490589094,Eleotris_sandwicensis:0.02693779607370674900)100:0.30150212652753943798,((Eleotris_smaragdus:0.32604379356460122530,Erotelis_armiger:0.16424249599079898676)100:0.30935049919659557416,((Eleotris_melanosoma:0.14814232143902206218,Eleotris_amblyopsis:0.19421270158536518835)76:0.04688108145876268562,Eleotris_fusca:0.07250992802935767034)94:0.09188160747473732293)12:0.00372166381337293807)37:0.03079655077807945743,(Eleotris_picta:0.00000176267459066039,Eleotris_pisonis:0.00886595743167908901)100:0.20451991328010901694)71:0.05990977695476105980)31:0.02614727665277727375,(Gobiomorphus_coxii:0.17461566830977348630,(Philypnodon_grandiceps:0.48552387767205529290,((Gobiomorphus_hubbsi:0.09255615235966467569,(Gobiomorphus_breviceps:0.09626888859468213377,Gobiomorphus_cotidianus:0.09110836291641707940)100:0.10568292486511522699)99:0.07324634111003862358,Gobiomorphus_australis:0.14479658417694327155)43:0.01735875869180278017)28:0.02055891377051327282)71:0.04702479630469385413)26:0.02959473589763988652)12:0.02684365565888071140,((Leptophilypnus_fluviatilis:0.19872348026499406237,Leptophilypnus_panamensis:0.24647548611358169324)100:0.39257390939919167838,((Hypseleotris_klunzingeri:0.08231247739569129418,((Hypseleotris_aurea:0.11473553146998173580,Hypseleotris_compressa:0.06662344087222485389)53:0.02555003278907716849,Hypseleotris_galii:0.10053221011364939230)25:0.01873180477108242828)99:0.09367898697200609248,(Hypseleotris_cyprinoides:0.03577619834976247803,(Hypseleotris_dayi:0.00682381143520808565,Hypseleotris_tohizonae:0.00577509644516387925)44:0.02026049198801209775)100:0.19643707910269234129)100:0.13870566165779699142)6:0.02031561793549031825)38:0.03265581516260104844,(((Hypseleotris_agilis:0.06400931363717886347,((Mogurnda_mogurnda:0.00000176267459066039,Mogurnda_adspersa:0.30341075276092066559)95:0.16143933620024153441,Ophieleotris_aporos:0.02718949898468365350)76:0.06952366166879786480)89:0.10852903221800007383,Tateurndina_ocellicauda:0.45862002331376039432)57:0.04920818580495782413,Ratsirakia_legendrei:0.28261622541073189163)63:0.04736949450227077169)79:0.10898188995242959198)32:0.04373623352746985882,(((Ophiocara_porocephala:0.24718895135556398501,(Bostrychus_sinensis:0.18216809240160750094,(Oxyeleotris_lineolata:0.05436929455785232540,(Oxyeleotris_marmorata:0.20732804554895398197,Oxyeleotris_selheimi:0.06991065675442310889)44:0.03178080977114922795)100:0.12940819424009411542)92:0.07331403164106763126)59:0.04693183591695967993,(Butis_amboinensis:0.27194844130481649058,Butis_butis:0.21142274977013386650)98:0.39808913752379143647)35:0.03644218372344885937,((((((((Gymnogobius_urotaenia:0.04825071741249915835,Gymnogobius_isaza:0.01710041525574699031)100:0.12949475364221846307,Chaenogobius_annularis:0.25464969879533055908)94:0.19020382563279816801,(Gillichthys_mirabilis:0.31326269890035890464,(Typhlogobius_californiensis:0.18052117431852060525,Eucyclogobius_newberryi:0.33620601514657477704)97:0.07909117421279647508)67:0.05493476552984359607)86:0.08238532075662693965,(((Acanthogobius_flavimanus:1.23250788754058349106,Rhinogobius_giurinus:0.29205635402984220228)13:0.10897097477322233161,((Pterogobius_virgo:0.14935181301173652835,Pterogobius_zacalles:0.11506262622020943520)97:0.14295154419002847890,(Sagamia_geneionema:0.41581126345363672581,(Pterogobius_elapoides:0.07031031215850000460,Pterogobius_zonoleucus:0.15359016768178740397)99:0.26323415641100728068)67:0.04646228796412979478)91:0.15769658884120163478)7:0.05480253037157067908,(Tridentiger_bifasciatus:0.25530805086263919090,Tridentiger_obscurus:0.17056376616907664712)99:0.20529266335770249285)6:0.07258678717734948216)4:0.08954570556964569494,(((Periophthalmus_barbarus:0.24536601636455043773,Periophthalmus_argentilineatus:0.13816224616446337592)100:0.21151574629544003803,(Odontamblyopus_rubicundus:0.39248635326820624503,(Taenioides_limicola:0.23965076078446395647,Scartelaos_histophorus:0.24663451298097599507)91:0.04568647718395347362)96:0.10865922639540222250)98:0.13156339224774310503,((((Stiphodon_elegans:0.14129223992184561376,(Sicyopterus_lagocephalus:0.11803003681297349892,(Sicydium_crenilabrum:0.08138713261341462557,Sicydium_plumieri:0.15881079645716417170)64:0.03024557188407203320)95:0.08763204713953016978)100:0.13241718390819839923,(Awaous_guamensis:0.29864903434537226623,Stenogobius_hawaiiensis:0.24045026369094457674)30:0.06046644401166802879)63:0.09588492816189941870,((Gnatholepis_thompsoni:0.04092270694560125321,Gnatholepis_scapulostigma:0.05702868213407853021)100:0.22165284324911760372,(Ctenogobius_saepepallens:0.50056811028518044537,Evorthodus_minutus:0.27596425680128794378)73:0.10026542981985525627)52:0.04098326878656766842)54:0.07385373308729682729,(Gnatholepis_davaoensis:0.14152873721545691788,(Gnatholepis_knighti:0.04669178587796049551,(Gnatholepis_anjerensis:0.05808741619989604699,Gnatholepis_cauerensis:0.10953122260033172630)29:0.03971633754661381410)86:0.21420759667887931399)75:0.16495684261436585616)13:0.06807387364168142330)6:0.06190565593667817640)3:0.02477555109789818938,(Pomatoschistus_minutus:0.76911977995889213577,Mugilogobius_rivulus:0.39674750494017618774)42:0.10857848103312650601)5:0.03856829951199983175,Pandaka_lidwilli:1.25370584075561652959)6:0.10479661479971859317,((((((Bathygobius_curacao:0.24534047511265946739,(Bathygobius_fuscus:0.30965062207888827128,Bathygobius_soporator:0.14747704904906219769)25:0.08103412727941729232)35:0.05491378063842056179,Bathygobius_cocosensis:0.26688672002733970778)100:0.27104375209989817197,(Glossogobius_olivaceus:0.64822285329227824491,Psammogobius_biocellatus:0.33029441250773045047)22:0.05400243760069456389)37:0.12162908596403673722,Trimmatom_eviotops:0.43381885180496387866)6:0.01837758048347734596,(((((Asterropteryx_semipunctata:0.12630475846277811058,Asterropteryx_ensifera:0.10508057797873308903)100:0.26786667926947327345,(Callogobius_bifasciatus:0.25390019040968136999,Callogobius_sclateri:0.22585719586754390842)96:0.20182121261469493367)22:0.02951676013116017946,((Trimma_flammeum:0.52939588122578218332,Trimma_caesiura:0.37239170044911557023)75:0.23107776272121022254,((((Amblyeleotris_gymnocephala:0.00000176267459066039,Lythrypnus_elasson:0.00000176267459066039)100:0.14999553063537693354,((Amblyeleotris_guttata:0.07051434920579705001,Amblyeleotris_steinitzi:0.08064957744651637950)99:0.07125335589841830242,(Amblyeleotris_yanoi:0.13006021546877147288,(Amblyeleotris_fasciata:0.00000176267459066039,Amblyeleotris_wheeleri:0.00342594327377586079)100:0.14244259294927649750)91:0.04364683009134427000)92:0.05998308677871069111)77:0.03536514951242589189,(Amblyeleotris_randalli:0.12524226472222121931,Amblyeleotris_periophthalma:0.19791820470684862165)100:0.15705464658052167848)98:0.10866884071765912922,((Vanderhorstia_ornatissima:0.15065654836677824790,Tomiyamichthys_lanceolatus:0.16919033299464553144)100:0.22336146225609707083,((((Eviota_afelei:0.49915233046934276429,Fusigobius_neophytus:0.00000176267459066039)89:0.23921022598370586532,Ctenogobiops_feroculus:0.03302755266069636736)64:0.11131024973346277662,Ctenogobiops_aurocingulus:0.14796494011659858114)38:0.07384397346918010474,(((Ctenogobiops_mitodes:0.23120131940234328605,(Ctenogobiops_formosa:0.17763719069050024069,(Ctenogobiops_crocineus:0.03034981394796777351,(Ctenogobiops_tangaroai:0.04714445104594792296,Ctenogobiops_maculosus:0.09768012500590518565)38:0.01647659676958570379)95:0.07911200642561040663)62:0.03058648968538958332)48:0.03795442221098308672,Ctenogobiops_tongaensis:0.21013043518154320144)51:0.04330298680420490570,Cabillus_tongarevae:1.37882371811462167699)6:0.00000176267459066039)11:0.10265456905981698577)10:0.05566476666645094051)11:0.07466766594684283442)4:0.03630123091298487203)4:0.06554851644940520861,(((Bryaninops_yongei:0.61390163291726551620,Gobiodon_histrio:0.45429777654735814529)95:0.22051038126971189701,((((Barbulifer_ceuthoecus:0.77350921339211298200,(Risor_ruber:0.44958126216609844983,Elacatinus_macrodon:0.55940068071483106671)86:0.20562786464372487250)35:0.05823011483581065628,(Gobiosoma_chiquita:0.00000176267459066039,Elacatinus_evelynae:0.00000176267459066039)100:0.34558353111226219889)48:0.13089084653376364953,(((Mesogobius_batrachocephalus:0.65768702961380309802,Zosterisessor_ophiocephalus:0.42104296839619193005)57:0.08954855489687273129,Coryogalops_anomolus:1.14189990640794736265)27:0.13230183699607370174,((((Priolepis_eugenius:0.85140769403720106823,Priolepis_hipoliti:0.06053813213465859128)70:0.50648845167843048465,Cerdale_floridana:0.01096609049867602360)48:0.45591755327243582707,(Microdesmus_bahianus:0.26247389865386971941,((Microdesmus_carri:0.18933433900559867857,Microdesmus_longipinnis:0.14410524276107303243)79:0.05289235189295962009,Microdesmus_dipus:0.12397487530847496606)99:0.10141452610700911474)92:0.10689211274375380600)40:0.19243890570934046869,Gunnellichthys_monostigma:0.65291078301449811949)34:0.09357754484271846307)1:0.03844720976866302514)1:0.04775615019473584966,(Nemateleotris_magnifica:0.42059779639804978357,(((Ptereleotris_monoptera:0.00516229146175154960,Ptereleotris_microlepis:0.00807489148944301460)100:0.20169268939822040942,(Ptereleotris_heteroptera:0.19032357076862224665,Ptereleotris_zebra:0.17087975255030743438)81:0.06031166674255142301)99:0.09233002108195417779,Ptereleotris_calliura:0.32137687113556007157)49:0.04115500371750135250)25:0.05359809393605140942)0:0.03938163557525740932)0:0.02754757153284727311,(((((Valenciennea_strigata:0.31173723209692394409,Valenciennea_longipinnis:0.24213640224233867748)87:0.07101350354265452414,Valenciennea_puellaris:0.30821020587083669540)84:0.07132862399713756074,Signigobius_biocellatus:0.31223091787043361078)96:0.08602095443383173112,(Amblygobius_nocturnus:0.30893077553638970123,Amblygobius_phalaena:0.34470604771130258781)86:0.06575244645188128845)94:0.08541581704315623358,((Favonigobius_melanobranchus:0.34526137232066095306,(Favonigobius_exquisitus:0.01746536573456112726,Afurcagobius_tamarensis:0.00735656424898150241)100:0.40381636215388472255)82:0.17384112689824224307,(((((Cryptocentrus_leptocephalus:0.26726640346117558344,(Cryptocentrus_inexplicatus:0.19224009809504319368,(Cryptocentrus_albidorsus:0.14600936679359904913,(Cryptocentrus_strigilliceps:0.00000176267459066039,Cryptocentrus_nigroocellatus:0.03162559831148611250)100:0.17246305690477423123)67:0.03209317612169924200)48:0.03810359490092122409)67:0.07671646346668301031,Cryptocentrus_lutheri:0.17572951020332969185)91:0.10936415999974793967,(Tomiyamichthys_oni:0.36836479927513016142,(Mahidolia_mystacina:0.32500631570826371552,Stonogobiops_xanthorhinica:0.44121179350315342660)28:0.01384533296205712333)27:0.04456338917021251955)27:0.04182801592888067688,Cryptocentrus_cinctus:0.29531857214115558863)66:0.09595851199978294721,(Arenigobius_bifrenatus:0.43298226477439649695,(Oplopomus_oplopomus:0.38666442900170094887,(Exyrias_belissimus:0.26712953104675596361,(Istigobius_rigilius:0.06423471701909712339,Istigobius_decoratus:0.02524905686961169404)100:0.17538013024832782571)89:0.13487001565145442794)27:0.05289974716768525154)22:0.02611662208435935537)15:0.02845820053233615379)11:0.06966896125523266980)0:0.01959751305518505488)0:0.05871984910931348639)0:0.05887477914882287278,(Acentrogobius_pflaumii:0.49332331774815624259,(((Gobiopterus_semivestitus:0.70598862604348577854,Lophogobius_cyprinoides:0.38608973052276973625)17:0.10876838460906763928,(Rhinogobiops_nicholsii:0.83375627793364692764,((Coryphopterus_glaucofraenum:0.18820308760128448555,(Coryphopterus_urospilus:0.43991363741965838763,Coryphopterus_dicrus:0.20440889028289657703)77:0.10336030365387377516)100:0.41975979548930669738,(Coryphopterus_lipernes:0.58632999302307153844,Coryphopterus_eidolon:0.16685775700656332865)41:0.07072826060257814129)97:0.24800867262480377762)90:0.24534667133707066533)17:0.36106413780839308192,(((Schindleria_pietschmanni:0.01136478983086763699,Schindleria_praematura:0.02063480746732106186)100:0.89427805889527633987,(Microgobius_microlepis:0.00139075386943734807,Ophiogobius_jenynsi:0.00000176267459066039)100:0.47808997147479559420)44:0.20600335254241386451,(((Lythrypnus_zebra:0.43230527253865935755,Lythrypnus_dalli:0.37946661961417466058)100:0.55406334336787033124,Priolepis_cincta:0.44877758945950646519)13:0.13372168451548127610,Nes_longus:0.61099539633005373052)5:0.08893823183593908832)3:0.03976242890215977988)0:0.01030472457139344419)0:0.03315592347236946680)0:0.04679753622389749845)4:0.03759392399205612606,(Kraemeria_cunicularia:0.45580744287019064886,Kraemeria_bryani:0.42176618761703499327)100:1.03481527695845842985)5:0.07153023842281326650)7:0.00664134080543082959)19:0.04747130194984604951)6:0.01805972323445833097)34:0.13124935018276448484)5:0.04190864625191294962)0:0.02901850983339598553,(((Verasper_variegatus:0.41314009385528799312,Paralichthys_olivaceus:0.23150173124054937435)92:0.35745286979978457831,((Takifugu_fasciatus:0.66960517707290634348,(Lagocephalus_gloveri:0.17929335681981994899,Lagocephalus_inermis:0.30576979097005890784)100:0.14568757787048919328)99:0.39714090496981657719,Trachurus_japonicus:0.34332616538815280416)12:0.06041110847972860215)1:0.04517647240305591033,(((Sebastiscus_marmoratus:0.42706007677349699936,Scorpaenopsis_cirrosa:0.48606426017821019459)70:0.18169752477942857638,(Nandus_oxyrhynchus:0.40561948500170974530,Badis_siamensis:0.52687555562955912247)69:0.11555166420699901353)1:0.04015608827826958743,(Macrognathus_siamensis:0.56118986687174521322,((((Epiplatys_sexfasciatus:0.24805882060400463729,Epiplatys_singa:0.47932959568048605359)100:0.18917067390403194405,(((Nimbapanchax_melanopterygius:0.08789687751136809890,Nimbapanchax_leucopterygius:0.04297451213307874884)100:0.07561608869173490344,(Nimbapanchax_viridis:0.10274987126589303632,Nimbapanchax_jeanpoli:0.05753257393026618705)100:0.07501071246752670507)100:0.22365063442024532558,(Archiaphyosemion_guineense:0.23555712186932242780,(Scriptaphyosemion_guignardi:0.21649872442075318579,Scriptaphyosemion_geryi:0.18706878672742147107)100:0.34677573688431956311)83:0.10773535380961829500)84:0.10063106959629286585)100:0.22506283807626303872,(Aphyosemion_elberti:0.51089792022421254281,(Fundulopanchax_gardneri:0.29773775563195747917,(((Nothobranchius_kafuensis:0.06254217516131617416,Nothobranchius_polli:0.18029712650270285357)100:0.24951330789915149300,((Nothobranchius_ocellatus:0.34743232127875378623,((Nothobranchius_krysanovi:0.06879383219062051857,(Nothobranchius_pienaari:0.06433401855236750566,Nothobranchius_rachovii:0.04450479562233698727)100:0.07577590241730806919)100:0.13152490317903356432,(Nothobranchius_furzeri:0.17642923984882458166,Nothobranchius_orthonotus:0.13887397416904093128)100:0.13610657273414089596)80:0.05133533103880284082)49:0.03225210826562315697,(((Nothobranchius_lucius:0.18515096878665102120,(Nothobranchius_melanospilus:0.16236146181473182226,(Nothobranchius_hengstleri:0.17880452662024770571,Nothobranchius_makondorum:0.14404966244420880361)64:0.03122048592511746795)51:0.01830501204912256957)99:0.09578960519261400797,(Nothobranchius_elongatus:0.09398316890752600461,Nothobranchius_jubbi:0.13372315080386493191)100:0.18140686255105839542)25:0.02211027319477957415,Nothobranchius_eggersi:0.31474391179640492933)34:0.03498555444932603675)38:0.02108129348551889687)80:0.12047010825726046923,Nothobranchius_rubroreticulatus:0.41339998016361334532)100:0.36660235844456551257)40:0.08014508184084552511)100:0.32101747079535825069)92:0.25215666254619323317,(Aplocheilus_panchax:0.76862485847689876461,(((Campellolebias_dorsimaculatus:0.68110906160991580016,Cynopoecilus_melanotaenia:0.51161107875850198035)100:0.58913834828902011953,(((Cynolebias_magnificus:0.37764634727570117745,Cynolebias_bokermanni:0.18872393261057557323)96:0.07349983823516727244,(Cynolebias_bellottii:0.20774861542250794288,Cynolebias_alexandri:0.24873765818672041727)99:0.03936167467106434920)100:0.16783039368260141422,(Nematolebias_whitei:0.54572452350260047815,Cynolebias_myersi:0.39559940697354234462)93:0.12320357597684777895)92:0.15957533826536027255)100:0.30195404339619935641,((Rivulus_cylindraceus:0.45929291097381136355,(Rivulus_roloffi:0.35891196575725176077,(((((Rivulus_magdalenae:0.17562494304025869085,((Rivulus_frommi:0.00754280845872416176,Rivulus_brunneus:0.00211388155908592531)100:0.10026989752823503577,(Rivulus_weberi:0.06531611531140590898,Rivulus_tenuis:0.12292309899166971310)99:0.06572673624093809674)98:0.04823207052785413035)85:0.03704592299393342364,((Rivulus_luelingi:0.09604514801859240225,(Rivulus_haraldsiolii:0.10743618646007434270,Rivulus_santensis:0.10199486871794853637)97:0.03235606888238565931)100:0.30991072145415721195,((Rivulus_punctatus:0.05688961493849546691,Rivulus_pictus:0.05927135491169002735)100:0.08593060990304761393,Rivulus_violaceus:0.07193810855146122452)100:0.28865148824813846584)34:0.03361926972056193674)33:0.01488891257088069929,((((Moema_staecki:0.49082436993216088661,Aphyolebias_peruensis:0.22806435316019318948)82:0.07965592018119389828,((Pterolebias_longipinnis:0.24210518920234527784,Pterolebias_phasianus:0.28200100700844848989)100:0.86913967265402081175,((Renova_oscari:0.58499680192203196150,Terranatos_dolichopterus:0.48621784986313093535)50:0.06448329170043475000,(((((Austrofundulus_guajira:0.12329335701482962984,((Austrofundulus_limnaeus:0.07134004752636860514,Austrofundulus_leoni:0.06701900227072106475)100:0.05029672545057134458,(Austrofundulus_leohoignei:0.08709384920363054394,(Austrofundulus_transilis:0.04411982359913869250,Austrofundulus_rupununi:0.03399714058663201588)100:0.05852317152724383076)100:0.10143049138133296283)62:0.03137986047656922406)100:0.18057444228269742270,(Rachovia_brevis:0.17182781205967415095,(Rachovia_hummelincki:0.16174913257981959891,Rachovia_pyropunctata:0.14578699940082406883)100:0.15069835956517368958)94:0.09204438801299388850)100:0.14193668531280395628,Rachovia_maculipinnis:0.52784195441973624785)87:0.09550968980130739028,Micromoema_xiphophorus:0.80241645941034334122)32:0.01758288053341731938,(Llanolebias_stellifer:0.51489636091903856485,(Gnatholebias_zonatus:0.00536209022871612928,Gnatholebias_hoignei:0.09930486656848733218)100:0.33809515387826633015)51:0.06479589164988723560)20:0.01984421406074828031)70:0.06742937858794488537)38:0.06510771668403825263)40:0.03593923883547234244,(Trigonectes_rubromarginatus:0.15817879499379491892,(Trigonectes_balzanii:0.00000176267459066039,Trigonectes_aplocheiloides:0.00774825932905609180)100:0.09260712732959298921)100:0.19171915822468341140)92:0.13069071321073444381,((((Rivulus_ophiomimus:0.14410308346951103409,Rivulus_compressus:0.12175376439887822255)38:0.03907071988748642838,(Rivulus_obscurus:0.25119635537359902289,Rivulus_jucundus:0.14765350876473384845)28:0.01666726034286758820)67:0.03432686092973367964,Rivulus_iridescens:0.17149246923822905608)73:0.03496402801772090924,(Rivulus_hartii:0.24781265260948678875,(Rivulus_waimacui:0.11293354379464760895,((Rivulus_stagnatus:0.08052474789342342620,Rivulus_urophthalmus:0.07041990242404896905)80:0.02121525919385816583,(Rivulus_deltaphilus:0.04661501790518096655,Rivulus_cryptocalus:0.03047959194374366687)100:0.06204105066900043181)100:0.06502609393505673352)100:0.05943132235318605849)82:0.02348765295803670328)62:0.02562402854413679820)57:0.04012678521608731286)41:0.02083302044395009614,(Pituna_poranga:0.60065549287902542108,(Papiliolebias_bitteri:0.51418750594182038860,(Maratecoara_formosa:0.11579576514995384984,Maratecoara_lacortei:0.09553883263311310770)100:0.29824755622881499173)22:0.04400995600673725944)100:0.50610428680668340107)98:0.11608668788959866647,((Rivulus_duckensis:0.90613490700245769016,(Rivulus_breviceps:0.25854562259564894955,(Rivulus_lyricauda:0.39571019129208084841,Rivulus_gransabanae:0.28765768161524996049)55:0.04947202966847423500)92:0.08134387268183780606)50:0.06770675929602323895,((Rivulus_frenatus:0.07987041668898987867,(Rivulus_agilae:0.07299308923709527108,Rivulus_geayi:0.08050002255333534029)52:0.03123231872583909219)100:0.34812061595350646259,(Rivulus_rectocaudatus:0.24763908319380092959,Rivulus_tecminae:0.30127581059364288985)92:0.12812642037083105118)65:0.07222826391911384147)93:0.11849121393465854302)61:0.05286092902304895258)54:0.05936340560816815576)96:0.16396814300679116161,((Kryptolebias_marmoratus:0.09648133730859123625,Rivulus_caudomarginatus:0.13352222407425942685)100:0.26651474340575886934,(Kryptolebias_sepia:0.00000176267459066039,Kryptolebias_braziliensis:0.00000176267459066039)100:0.35254610189481311311)95:0.13819386350860973778)84:0.08507805467071399841)98:0.35722085992626667617)61:0.05672534928141009791)70:0.18723918278829168593)21:0.10114133699779294384)0:0.04928160566446670998)0:0.03533483500938500166)0:0.04508874303697844865,((((Cichla_temensis:0.18648399901441445325,(((Satanoperca_jurupari:0.25876380194536841017,(Crenicichla_lepidota:0.19485731845240006344,((Crenicichla_missioneira:0.00408245388080528786,Crenicichla_minuano:0.00466044254665752729)100:0.10342406989195261635,(Crenicichla_lacustris:0.15375453898561070032,((Crenicichla_hu:0.03633238131000721127,((Crenicichla_mandelburgeri:0.00568375800033611798,Crenicichla_yaha:0.01068401015076187073)70:0.00416080762303344502,Crenicichla_tesay:0.00486738923782409436)95:0.01099130531955106252)97:0.01849443530202253202,(Crenicichla_vittata:0.07120049529552933965,(Crenicichla_punctata:0.05322411551902734433,Crenicichla_gaucho:0.03950555619441599442)100:0.04562605355175418376)58:0.01220457664782957641)95:0.04904243132037162251)93:0.06410817121513873174)91:0.10621984568331857557)100:0.32150108999547305499)39:0.05541296078575305706,Astronotus_ocellatus:0.34947895395092770654)54:0.04947019965593581203,(Hoplarchus_psittacus:0.19399524311513877128,((Amphilophus_macracanthus:0.11562903886718425839,(((Nandopsis_tetracanthus:0.15923016453123894909,Nandopsis_haitiensis:0.11905599439882028068)86:0.04916404279973148744,(Caquetaia_kraussii:0.16014204028270914715,(Cryptoheros_spilurus:0.07256566883744575147,(((Cichlasoma_urophthalmum:0.03032005095739932765,Petenia_splendida:0.04890068410113609237)100:0.06188938827005465576,((Amphilophus_citrinellus:0.04297105840221931983,Cichlasoma_trimaculatum:0.04623197776013288768)95:0.01907835111424615801,Cichlasoma_istlanum:0.10861453668699923292)54:0.01004077091456689538)29:0.00688101002327699742,Parachromis_managuensis:0.07584412014837327298)48:0.00901759310044576712)85:0.03065479204103919650)44:0.02127767129706260169)26:0.01620113561935455568,Rocio_octofasciata:0.16786520709687688391)18:0.01019018298678148540)24:0.01103990634121132172,(((Vieja_intermedia:0.05746921183444256287,Cichlasoma_pearsei:0.03301367652494952704)100:0.03598927858464603946,((Paraneetroplus_bulleri:0.04264777551354691915,(Vieja_fenestrata:0.01052754301948872345,((Vieja_bifasciata:0.00120463163895823751,Vieja_hartwegi:0.00122558525853621453)100:0.01869006238688208227,Theraps_irregularis:0.01208907870121650983)66:0.00878006773207956268)95:0.02717485105596063877)98:0.04745571590898213510,((Herichthys_bartoni:0.04678032493760760080,(Herichthys_steindachneri:0.01732438071719849884,Herichthys_pantostictus:0.02405515797697470315)85:0.01524560263781003962)100:0.04043579077364303082,((Herichthys_cyanoguttatus:0.01166309747643218438,(Herichthys_tamasopoensis:0.00953350488837898490,Herichthys_deppii:0.00652997567261288936)74:0.00252267744420948785)100:0.02691966540286235346,Herichthys_minckleyi:0.02589533009831603005)100:0.03974470656818433062)100:0.03749738335834579966)43:0.01005618152128619908)98:0.05358285981054793090,(((Thorichthys_helleri:0.03263816998911854561,(Thorichthys_affinis:0.00226336865603718983,Thorichthys_meeki:0.01524334349118652496)94:0.02363303219134024052)100:0.07812036832814979459,(Thorichthys_socolofi:0.06740546813108269641,(Thorichthys_callolepis:0.03926802098054460455,(Thorichthys_maculipinnis:0.00000176267459066039,Thorichthys_ellioti:0.00235405713278327320)99:0.01945187702577211644)100:0.04308764781658904613)100:0.05575002147918121237)99:0.05943211621547674073,Cichlasoma_salvini:0.18303048227703688777)40:0.00615231398002611275)88:0.03136358894396517466)99:0.08796110935552642340)96:0.05756007396363623657)32:0.03119163265395636167)87:0.08796769477301570606,(((Tylochromis_leonensis:0.16083480832038829500,Tylochromis_polylepis:0.12024801774415457001)99:0.06731365226893487597,(((Pelmatochromis_buettikoferi:0.11715740812588024133,(Pelmatochromis_nigrofasciatus:0.14472377909482284086,Pterochromis_congicus:0.14523213754169836398)62:0.01780119209185070800)100:0.07148013157825289232,((((Steatocranus_irvinei:0.19912570946599966737,(Tilapia_busumana:0.02137780722530541189,Tilapia_discolor:0.01833377833229554374)100:0.09305159032966262012)86:0.03615312943251793726,(Tilapia_brevimanus:0.13008009360216066241,Gobiocichla_wonderi:0.19822018423688017585)84:0.03815294266034219978)91:0.05265140449549597645,(Etia_nguti:0.15262249847250305979,((((Tilapia_walteri:0.03240513689566548727,((Tilapia_coffea:0.02115185952166374547,Tilapia_zillii:0.01014372446750834607)100:0.01726061638205150447,Tilapia_rendalli:0.05326448464020729773)86:0.00895177210306465423)100:0.04016078254350500393,(((Tilapia_louka:0.01642062736832122782,Tilapia_guineensis:0.02015532115798641596)100:0.04157541543210689639,Tilapia_dageti:0.03698803862493785927)100:0.03911378122034787908,(Tilapia_tholloni:0.12623119525071790914,(Tilapia_buttikoferi:0.03406713828956289825,Tilapia_cessiana:0.04301158686561760286)94:0.02695390734646019651)41:0.00868535226453326890)24:0.00631291071081760326)50:0.01537838215661983463,Tilapia_joka:0.10284476645134497941)100:0.05317983765216023617,(((((Tilapia_sparrmanii:0.01157554738714587118,Tilapia_guinasana:0.01306530228619717272)100:0.10767752141199526950,(Chilochromis_duponti:0.13446569527945972933,Tilapia_ruweti:0.11360112979551319212)23:0.01233992439713935997)9:0.01255643533023137991,(Sarotherodon_caudomarginatus:0.03537229085560606584,(Sarotherodon_occidentalis:0.04077686588256219241,(((Sarotherodon_mvogoi:0.03414806700513786653,(((((Oreochromis_schwebischi:0.02593722306227646407,Oreochromis_tanganicae:0.01731030491517439665)88:0.01262896650210804626,(((Oreochromis_karongae:0.02229105649906578876,(Nyasalapia_variabilis:0.01211222465572336406,Alcolapia_alcalica:0.04197324785364141969)61:0.00578626160561576241)97:0.01648470375088018081,Oreochromis_amphimelas:0.02531188166687270424)56:0.00392911615909633059,Oreochromis_urolepis:0.04360445183395065399)31:0.00279958615421199057)43:0.00586378910794810564,((Oreochromis_andersonii:0.00113343691044914254,Oreochromis_mossambicus:0.00863031086051585516)99:0.00971678273958609395,(Oreochromis_mweruensis:0.00736613348899461083,Oreochromis_macrochir:0.02314539237596049265)68:0.00081479605980790180)100:0.02856303203943982155)59:0.00777572929844196764,(Oreochromis_esculentus:0.05640060960695571224,Oreochromis_leucostictus:0.05567686591774268640)26:0.01042022141517738761)13:0.00112849710665306985,Oreochromis_niloticus:0.05271195905891749856)100:0.04174580292404588000)19:0.00214114949348039084,(Iranocichla_hormuzensis:0.12640924462984989840,(((((Oreochromis_aureus:0.01095766195878594522,Sarotherodon_galilaeus:0.00245998346485935956)83:0.00114229983681238051,Sarotherodon_galilaeus_multifasciatus:0.00360979083280328671)84:0.00116160612412505586,((Stomatepia_pindu:0.00119024840199544830,((Stomatepia_mongo:0.00359669586619338960,(Pungu_maclareni:0.00479863252037228318,(Konia_eisentrauti:0.00724476382293272672,Konia_dikume:0.00238856085136904348)97:0.00239309795524970427)85:0.00239797228470967723)12:0.00000176267459066039,(((Sarotherodon_caroli:0.00240718488472212253,(Sarotherodon_linnellii:0.00482695797546865354,Sarotherodon_lohbergeri:0.00239978183315081714)25:0.00000176267459066039)18:0.00000176267459066039,Sarotherodon_steinbachi:0.00361998209711607071)73:0.00239658257514982964,Myaka_myaka:0.00600840201657077723)19:0.00000176267459066039)5:0.00000176267459066039)11:0.00000176267459066039,Stomatepia_mariae:0.00119015099026965475)50:0.00243620113861028973)80:0.00435711803121292558,Sarotherodon_galilaeus_sanagaensis:0.02525509116536693577)99:0.02362271234329990643,(Sarotherodon_melanotheron_nigripinnis:0.01550405264138498586,Sarotherodon_melanotheron:0.00409002672407009302)100:0.03902505717922939016)54:0.01088283744806697526)8:0.00051085517682981929)44:0.00869190019255515825,Tristramella_simonis:0.05725814570508383494)6:0.00000176267459066039)77:0.01812715940701503659)74:0.01560897724679486190)7:0.00435628916983935889,(Tilapia_mariae:0.08103952756929350132,Tilapia_cabrae:0.08699934532264097520)77:0.02755310037671062848)16:0.00667335187699125577,((((Steatocranus_bleheri:0.04359933272802927334,Steatocranus_rouxi:0.03977777555596963277)66:0.01111479070377741594,(Steatocranus_tinanti:0.02392520713179186950,((Steatocranus_mpozoensis:0.00674165971216038936,Steatocranus_glaber:0.00901589839995677392)100:0.01852083513093748246,Steatocranus_gibbiceps:0.01962031948923271749)96:0.01364148410982166625)97:0.02471237480249085725)73:0.01319058964528719083,(Steatocranus_ubanguiensis:0.05803467832610103433,Steatocranus_casuarius:0.05586814796153324880)73:0.01088008808128882766)100:0.16174693809021481461,((Boulengerochromis_microlepis:0.05962422331551489596,(((Trematocara_unimaculatum:0.11152403409401007162,Telotrematocara_macrostoma:0.11599006982867500481)100:0.08829935796100858714,Hemibates_stenosoma:0.09301998624273088989)66:0.00444032459464860536,(Bathybates_minor:0.07105995913803364594,(Bathybates_graueri:0.03333468738159506978,((Bathybates_fasciatus:0.03512645571847956144,Bathybates_hornii:0.03667275943513417791)75:0.00851453816078378417,((Bathybates_leo:0.01400819873754473484,Bathybates_vittatus:0.02104948206240297645)83:0.01035648484372577068,Bathybates_ferox:0.04175556443154306019)33:0.00224397763115517183)59:0.00740101980230304605)100:0.04470325401590765496)95:0.04229237481790677072)95:0.02009232294181347023)35:0.00897959059699548166,((((Cyprichromis_microlepidotus:0.02001475877997785716,((Cyprichromis_leptosoma:0.02303259888460199717,Cyprichromis_coloratus:0.01655660614797550140)76:0.00574385602042569617,(Cyprichromis_zonatus:0.03431000067746552140,Cyprichromis_pavo:0.01992790320334127813)48:0.00425229287179768392)55:0.00531533204517165275)100:0.02616075178921484570,(Paracyprichromis_brieni:0.01006603189535292854,Paracyprichromis_nigripinnis:0.00000176267459066039)100:0.02260852913230393738)98:0.02285044415646566782,(Cyphotilapia_frontosa:0.07272354918658095746,(((((Orthochromis_luichensis:0.05164401279882175483,(Orthochromis_uvinzae:0.07106941968190555459,(Orthochromis_rugufuensis:0.02152752728113288549,(Orthochromis_rubrolabialis:0.05349341554210212957,(Orthochromis_kasuluensis:0.02612092202745979613,((Orthochromis_malagaraziensis:0.00000176267459066039,Orthochromis_mazimeroensis:0.00484317806869959903)98:0.00109714523896623515,Orthochromis_mosoensis:0.01373837533698354756)100:0.01911401875572519768)46:0.00181609905340682502)45:0.00148188634910148683)77:0.01202063623330518541)100:0.03170966160930514116)100:0.03912266006405358543,(Xenochromis_hecqui:0.02211271502068517386,(((Plecodus_multidentatus:0.02538213344574861280,Perissodus_eccentricus:0.02848721264605160244)88:0.00537451161640724231,(Perissodus_microlepis:0.01681038263859546342,Plecodus_straeleni:0.00221294252721192849)100:0.04663526978729543027)74:0.00552271416955069268,(Haplotaxodon_microlepis:0.01990425748853015542,(Haplotaxodon_trifasciatus:0.02148561571403533738,(Plecodus_paradoxus:0.01588781876055731304,Plecodus_elaviae:0.00212492890278443997)99:0.00847070592386423883)91:0.00806513698250406301)31:0.00099547782836034303)51:0.01362953394502261495)100:0.04121619456661376052)20:0.00084563939916123296,((Callochromis_macrops:0.02511003078457258245,(Callochromis_pleurospilus:0.03474142578176129398,Callochromis_stappersii:0.03013020372240600084)40:0.00149831566935474812)100:0.05903249006049931386,(((Grammatotria_lemairii:0.10471046481242227144,(Xenotilapia_caudafasciata:0.02100504187160910988,Xenotilapia_longispinis:0.00261016729259473147)100:0.03523514956892510180)42:0.01007958235652394591,((Xenotilapia_leptura:0.04394001369682435515,((((Xenotilapia_sima:0.01584881287295139551,Xenotilapia_boulengeri:0.00432246619529802999)100:0.01352530086960420340,Xenotilapia_ochrogenys:0.04223823903729597568)44:0.00565000350135351492,Xenotilapia_melanogenys:0.04675032883553495444)41:0.00391590880418583691,Xenotilapia_flavipinnis:0.03458300286325886180)54:0.00441852965145623361)46:0.00616401159143729546,((Xenotilapia_bathyphila:0.04634080013235907652,(Microdontochromis_tenuidentatus:0.02808018117992914023,Xenotilapia_spiloptera:0.02506972328751125648)86:0.00571771127139053016)45:0.00342106327649237003,Microdontochromis_rotundiventralis:0.05865975047948863291)37:0.00580587152542720239)47:0.01236264466382301815)74:0.01044255880657798952,(((Lestradea_stappersii:0.02358205320678541383,(Lestradea_perspicax:0.01950545834598656561,(Aulonocranus_dewindti:0.01657148532593607712,Cunningtonia_longiventralis:0.00434050580965139945)99:0.01472310025439329921)71:0.00556865109304348349)94:0.00763836136621254790,Ectodus_descampsii:0.04343310298068433356)88:0.00996973172701920429,(Cardiopharynx_schoutedeni:0.02833552782624085276,(((Cyathopharynx_furcifer:0.01358369617397379471,Ophthalmotilapia_ventralis:0.01209407055430861827)71:0.00742893397412480727,(Ophthalmotilapia_nasuta:0.00897742105987821976,Ophthalmotilapia_heterodonta:0.00631454033013804580)100:0.01519806033132108623)67:0.00214254709305696086,Ophthalmotilapia_boops:0.02298986789000432435)87:0.00731867709094244801)99:0.02031286364675739808)100:0.02715200964393330724)45:0.01508621548562976561)100:0.04229467883736962724)29:0.00775663562762016548,((Benthochromis_tricoti:0.00918931625385705809,Benthochromis_melanoides:0.00177023935038273005)100:0.07330287059412256279,(((Astatoreochromis_alluaudi:0.09531388874509366671,(Ctenochromis_pectoralis:0.13577392003406890675,((Pseudocrenilabrus_multicolor:0.00252931310555311404,Pseudocrenilabrus_multicolor_victoriae:0.00496702045935075529)100:0.03999922965637751210,(Pseudocrenilabrus_nicholsi:0.05772434904617845808,(Pseudocrenilabrus_philander:0.00528901339192165004,Orthochromis_machadoi:0.00882706514649110943)100:0.01392238227763354513)49:0.00760584439053584461)100:0.07214154052039349618)13:0.00000176267459066039)17:0.00281107819450651403,(((Tropheus_polli:0.01746245914750002570,(Tropheus_brichardi:0.00288474649305244446,Tropheus_moorii:0.00317359586762801108)100:0.01051329603098095922)100:0.00815908004624335027,((((Petrochromis_famula:0.02126617684547027487,(Interochromis_loocki:0.01006656463002681377,Petrochromis_fasciolatus:0.00426233628580619580)96:0.00727825341184208056)93:0.00776927893451132608,((Petrochromis_trewavasae_ephippium:0.00594980383099390393,Petrochromis_polyodon:0.00365925268520096237)100:0.00948512465015239649,Petrochromis_trewavasae:0.02407658822388265840)78:0.00362417548023657781)31:0.00120867613420391502,(Lobochilotes_labiatus:0.02206898547122649809,((((Gnathochromis_pfefferi:0.04409782274160128912,Simochromis_marginatus:0.02366631233935368589)61:0.00914116659207954776,(((Ctenochromis_horei:0.01744740005266202584,Limnotilapia_dardennii:0.00682975010122182681)69:0.00606352994080295877,(Simochromis_pleurospilus:0.01540256141990292010,(Simochromis_diagramma:0.00213882532050796003,Simochromis_babaulti:0.00487170157185504073)100:0.03007260503982227620)97:0.02027788761369627574)4:0.00000176267459066039,Pseudosimochromis_curvifrons:0.01632264091280225329)19:0.00191859463295658713)26:0.00122738322060167741,Petrochromis_orthognathus:0.02677793945667606909)78:0.00895928449072342162,Petrochromis_macrognathus:0.02466109999894259067)4:0.00000176267459066039)33:0.00215523006776664706)78:0.00627618607027585589,Tropheus_duboisi:0.03076406402972709647)98:0.00982284035210145845)99:0.01496938159791373797,((Haplochromis_bloyeti:0.05876611770388909251,((Haplochromis_paludinosus:0.04692809750549828407,(Astatotilapia_stappersii:0.00263309742710074327,((Haplochromis_squamipinnis:0.00235105737526214529,Haplochromis_insidiae:0.00351531809920781722)57:0.00586213777370427676,(Xystichromis_phytophagus:0.00118678562536521457,(((Ptyochromis_sauvagei:0.00356226395408249224,Haplochromis_obliquidens:0.00358614630793113254)13:0.00000176267459066039,Platytaeniodus_degeni:0.00000176267459066039)13:0.00000176267459066039,(Haplochromis_nubilus:0.01564548484968213485,Haplochromis_phytophagus:0.00117965215835179977)26:0.00000176267459066039)61:0.00118453230652266128)92:0.00495140464436771149)33:0.00218586078332598825)100:0.04199181869907746972)88:0.00836663806121649110,Haplochromis_gracilior:0.05069535584060116951)92:0.00599547754847581851)63:0.00391907945785268524,(Haplochromis_burtoni:0.04175765772586629426,(((Astatotilapia_calliptera:0.01794701583603614675,((((((((((Maylandia_livingstonii:0.00119120164859739110,Genyochromis_mento:0.00119063487612469242)19:0.00000176267459066039,Melanochromis_auratus:0.00119075330055998956)30:0.00118586331204626654,(((Lethrinops_longipinnis:0.01045021425001748522,Lethrinops_microdon:0.00253132723302972300)66:0.00127187478126448940,Alticorpus_pectinatum:0.00255164264959578755)31:0.00000176267459066039,Lethrinops_gossei:0.00384055080350199918)24:0.00000176267459066039)1:0.00000176267459066039,((Maylandia_heteropictus:0.00132430322414125870,Placidochromis_milomo:0.00353115518457926139)43:0.00000176267459066039,Melanochromis_parallelus:0.00000176267459066039)60:0.00000176267459066039)2:0.00000176267459066039,Labeotropheus_fuelleborni:0.00000176267459066039)1:0.00000176267459066039,(Pseudotropheus_elongatus:0.00118131000358017834,Cynotilapia_afra:0.00118128168206082921)4:0.00000176267459066039)1:0.00000176267459066039,((Aulonocara_hansbaenschi:0.00000176267459066039,Maylandia_aurora:0.00118081769230846405)69:0.00236800525204417618,Labeotropheus_trewavasae:0.00117834921101024961)14:0.00000176267459066039)6:0.00000176267459066039,Aulonocara_stuartgranti:0.00118074290331535636)41:0.00118105262196624753,Cyathochromis_obliquidens:0.00000176267459066039)85:0.00354001762863532252,Copadichromis_virginalis:0.02450234010276506127)55:0.00193499518889446963)43:0.00134976431672308357,((((((Protomelas_spilopterus:0.00465038491526930872,Cyrtocara_moorii:0.00230517927221292069)26:0.00000176267459066039,(((Otopharynx_heterodon:0.00115923073107613715,Protomelas_annectens:0.00231071299912997486)12:0.00000176267459066039,Mylochromis_inicola:0.00000176267459066039)3:0.00000176267459066039,((Copadichromis_jacksoni:0.00130387549274494155,Ctenopharynx_pictus:0.00231424312940385710)43:0.00000176267459066039,Mchenga_eucinostomus:0.00347792665309947218)20:0.00000176267459066039)4:0.00000176267459066039)22:0.00000176267459066039,Otopharynx_walteri:0.00584967210728502222)54:0.00150150079749927843,Mylochromis_anaphyrmus:0.00618667225413643308)79:0.00659264825587282510,(((Exochochromis_anagenys:0.01558887666764273774,(Buccochromis_oculatus:0.00636508129189994505,(((((Nimbochromis_linni:0.00348654736230266269,Nimbochromis_venustus:0.00116002830386617616)64:0.00115138300564681349,(Hemitilapia_oxyrhyncha:0.00115316820398848169,(Nimbochromis_livingstonii:0.00000176267459066039,(Lethrinops_auritus:0.00395608710105828897,Nimbochromis_polystigma:0.00115736061328512285)5:0.00000176267459066039)2:0.00000176267459066039)6:0.00000176267459066039)59:0.00115257600673171240,((Taeniolethrinops_praeorbitalis:0.00000176267459066039,((Aristochromis_christyi:0.00115047972611653500,Buccochromis_heterotaenia:0.00000176267459066039)68:0.00115140148886720781,((Otopharynx_speciosus:0.01153120218403484720,Tramitichromis_variabilis:0.01975291614771804496)27:0.00000176267459066039,Taeniolethrinops_laticeps:0.00125561205088950086)26:0.00000176267459066039)3:0.00000176267459066039)15:0.00000176267459066039,((((Labidochromis_caeruleus:0.00579063088652803735,(Protomelas_fenestratus:0.00351804951553323295,(Mylochromis_mola:0.00231516398192072948,(Cheilochromis_euchilus:0.00233816565780297714,Placidochromis_johnstoni:0.00232877440716218855)45:0.00115492647464733589)9:0.00000176267459066039)32:0.00115483253751638508)5:0.00000176267459066039,(Copadichromis_quadrimaculatus:0.00125676239753120676,Protomelas_taeniolatus:0.00000176267459066039)55:0.00115116437559913891)1:0.00000176267459066039,Eclectochromis_ornatus:0.00463631329028469513)38:0.00230641889941113039,Taeniolethrinops_furcicauda:0.00000176267459066039)41:0.00115077001399129658)9:0.00115168888353896649)0:0.00000176267459066039,(((Dimidiochromis_compressiceps:0.00347221747614824376,Copadichromis_borleyi:0.00000176267459066039)55:0.00115229090662890481,(Dimidiochromis_kiwinge:0.00348157703308831380,Nimbochromis_fuscotaeniatus:0.00231873927907346594)39:0.00115148974313431310)8:0.00000176267459066039,((Protomelas_similis:0.00115030475814947397,Protomelas_spilonotus:0.00231157597925105108)4:0.00000176267459066039,(Nyassachromis_prostoma:0.00347025993345360896,Copadichromis_mbenjii:0.00230176151876691163)25:0.00000176267459066039)1:0.00000176267459066039)0:0.00000176267459066039)1:0.00000176267459066039,Fossorochromis_rostratus:0.00230927712472511889)3:0.00000176267459066039)5:0.00000176267459066039)16:0.00341204489447971332,((Stigmatochromis_woodi:0.00251747328244730740,((Otopharynx_brooksi:0.00253222964544670909,Sciaenochromis_benthicola:0.01182933439214235369)60:0.00126146600455905402,Sciaenochromis_psammophilus:0.00000176267459066039)14:0.00000176267459066039)35:0.00125735542300725893,(Tyrannochromis_nigriventer:0.00634592202612590563,Tyrannochromis_macrostoma:0.00000176267459066039)50:0.00125113443719349678)2:0.00000176267459066039)17:0.00107724005312703265,Docimodus_evelynae:0.00489444936793689573)51:0.00291063721880516386)52:0.00355387051905823540,(Lethrinops_furcifer:0.00762251349950077931,Tramitichromis_brevis:0.00921540323922893576)53:0.00541220014778903257)82:0.01279007774738094051)57:0.00159771686566900599,((Diplotaxodon_limnothrissa:0.01395945916495785338,(Diplotaxodon_greenwoodi:0.00638762453280529267,(Diplotaxodon_macrops:0.00126390952682345001,Pallidochromis_tokolosh:0.00253420224724092797)64:0.00000176267459066039)93:0.00179033990838895060)100:0.00875672173365958303,(Rhamphochromis_esox:0.00133636656749595937,(Rhamphochromis_macrophthalmus:0.00797842033172926207,Rhamphochromis_leptosoma:0.01326277993267667606)83:0.00111923997830173992)100:0.02041980311913505972)88:0.00289130821116282723)99:0.01403787263919108533)25:0.00144354416297109598)56:0.00362357220962711106)94:0.01815313294572904260)29:0.00304094216647761038,(((Schwetzochromis_stormsi:0.00967598801928401184,Orthochromis_polyacanthus:0.01771849436828882435)100:0.05776808837332449154,(Haplochromis_oligacanthus:0.04632187334948009022,((Haplochromis_polli:0.08357671950025387375,((Serranochromis_robustus:0.00095510913580517493,Serranochromis_thumbergi:0.01156987454535360338)100:0.01263243253272797878,(Serranochromis_stappersi:0.00971160070569745439,(Thoracochromis_buysi:0.00852570502147385542,Thoracochromis_albolabris:0.00684073403938942771)96:0.01171087610057282812)60:0.00430144252909768487)77:0.00379308215452864046)37:0.00557362415666799813,((Chetia_brevis:0.00122159697162951678,((Serranochromis_altus:0.00479371422670751401,Serranochromis_angusticeps:0.00728274501336850875)24:0.00000176267459066039,Sargochromis_coulteri:0.00360088819899827576)58:0.00118149384880049521)97:0.00772536973430139155,(((((Pharyngochromis_acuticeps:0.00240785666254876443,Chetia_flaviventris:0.00856363461835375761)26:0.00000176267459066039,(Sargochromis_giardi:0.00241365129563231003,Sargochromis_mellandi:0.00241892310478350209)15:0.00000176267459066039)23:0.00000176267459066039,Sargochromis_codringtonii:0.00364187634557854457)61:0.00120891157833846415,(Chetia_brevicauda:0.01109433210792052857,Sargochromis_carlottae:0.00000176267459066039)32:0.00000176267459066039)96:0.00747668201883295441,Serranochromis_macrocephalus:0.00877000977582082127)50:0.00301981009498306563)96:0.01111454980951195014)49:0.00350326927121309391)100:0.03414101967595741666)80:0.01493076234714150324,(Schwetzochromis_neodon:0.05037025151161039771,(Cyclopharynx_fwae:0.00219150941677200223,Thoracochromis_brauschi:0.00603552384805402901)100:0.04085795526310630199)93:0.02115994377497710036)78:0.01349901428381398809)96:0.02057585805270303880)15:0.00000176267459066039)14:0.00327980139157348647,((Limnochromis_abeelei:0.02548337088197070652,(Limnochromis_staneri:0.01717832334067092431,(Greenwoodochromis_christyi:0.00499673895353812576,Greenwoodochromis_bellcrossi:0.00456988348266680958)100:0.01666208972161693780)24:0.00270308720915169668)98:0.01607916156907596844,((Gnathochromis_permaxillaris:0.03375967922939478866,((Reganochromis_calliurus:0.01495478016860967568,Baileychromis_centropomoides:0.01718622167436283099)100:0.02342490814370849772,Limnochromis_auritus:0.01464987876300540351)77:0.00178172263996843319)92:0.00797793345447182084,Triglachromis_otostigma:0.05166649787135518118)61:0.00329138879550094606)91:0.01335047114916049216)11:0.00545282659605931305)9:0.00289546646207184928)95:0.01731442688032845678,(((((Neolamprologus_ventralis:0.02559674136742786862,(Lamprologus_ornatipinnis:0.02329831671834067808,((Lamprologus_laparogramma:0.00000176267459066039,Lamprologus_signatus:0.00354581245420795789)100:0.01565953130184049788,Lamprologus_kungweensis:0.02115923611071142407)96:0.00404156771836354338)97:0.01357814657727941798)99:0.01768509079675327847,(((Neolamprologus_calliurus:0.00403846728453036326,(Lamprologus_speciosus:0.00591243984387902852,Neolamprologus_brevis:0.00341896612798514620)59:0.00181481994565172561)100:0.04052709271127556689,Neolamprologus_multifasciatus:0.02633542215609439158)72:0.00256173354243826971,((Neolamprologus_caudopunctatus:0.03281406954222262223,Lamprologus_lemairii:0.01511193861323005191)95:0.01057889444723031543,(((Lamprologus_meleagris:0.05540829941654160790,Lepidiolamprologus_nkambae:0.02511203559528247045)48:0.00136446912054768109,(((Lepidiolamprologus_elongatus:0.01591677682412933476,Lepidiolamprologus_profundicola:0.01228747398801095271)100:0.00668305334628494646,(Neolamprologus_variostigma:0.01225540246417119485,((Lepidiolamprologus_kendalli:0.00237989112480220012,Lepidiolamprologus_attenuatus:0.00724614197250452244)9:0.00000176267459066039,(Neolamprologus_hecqui:0.00617466100613169987,Neolamprologus_boulengeri:0.00367698970313808972)66:0.00241142708841106564)79:0.00493864815059256220)75:0.00325564681566740781)83:0.01140238903822801513,(((Altolamprologus_calvus:0.00852415771775247993,Altolamprologus_compressiceps:0.00255888668948942065)98:0.01935859186247505456,(Lamprologus_callipterus:0.02983383217772416635,(Neolamprologus_wauthioni:0.02433597267020643634,Neolamprologus_fasciatus:0.01928188315124940849)47:0.00260329669419197534)70:0.00452844256022758192)77:0.00770343276816938712,Lamprologus_ocellatus:0.03488651875029401273)97:0.01304167507943611651)49:0.00321029833067698915)74:0.00892037685154768896,Neolamprologus_leloupi:0.09080197095738722080)34:0.00229395460868544185)85:0.01440882924596918399)81:0.00988492184722585855)60:0.00219072265080348183,(Neolamprologus_meeli:0.02557840193156162706,Neolamprologus_similis:0.03678260153787830250)88:0.02705551140555695691)80:0.01024956781191480211,(((((Neolamprologus_leleupi:0.00605594762105747097,Neolamprologus_longior:0.00309034414586202436)100:0.04007164772486392079,(Neolamprologus_obscurus:0.03274580022223638059,Neolamprologus_prochilus:0.02355611788188077468)96:0.01363077045780233809)80:0.01156464221201504265,(((Neolamprologus_buescheri:0.04452035689111781547,(((Chalinochromis_brichardi:0.00988145388438641144,(Julidochromis_dickfeldi:0.01223649796931134369,(Julidochromis_transcriptus:0.00116212131531927623,Julidochromis_ornatus:0.00953033767766181018)100:0.00845062953258588455)98:0.00214798036798340012)100:0.02252607916865527124,Neolamprologus_furcifer:0.03490362573890125331)94:0.00988114367644574686,((Neolamprologus_tetracanthus:0.00222501141729797595,Neolamprologus_modestus:0.01053886313377708911)100:0.00437198437681936367,Lepidiolamprologus_cunningtoni:0.01355898655836984198)100:0.03875072449881664799)65:0.00587143078854287723)65:0.00400193062994505522,(((Neolamprologus_petricola:0.03179347535227885352,(((Lamprologus_congoensis:0.00544040015918815199,((Lamprologus_teugelsi:0.00226781148240798704,Lamprologus_mocquardi:0.00225686121579965352)100:0.00454143913425497127,Lamprologus_werneri:0.00126730310043807496)64:0.00267927465271564210)100:0.02439758192771657175,(Neolamprologus_devosi:0.02397270749014401500,Telmatochromis_vittatus:0.03329015169577985434)58:0.00356582680158087873)68:0.00791347676426445816,Neolamprologus_christyi:0.03505059570075352154)54:0.00166764003914535532)84:0.00791830730690553340,(((Telmatochromis_bifrenatus:0.01070272745646354273,(Telmatochromis_dhonti:0.00697217256875006484,Telmatochromis_temporalis:0.00815366091637362463)85:0.00544374672355105171)100:0.01490522582004809998,((Julidochromis_regani:0.00567731445321045629,Julidochromis_marlieri:0.00346608408745748085)99:0.02011732598378063869,Telmatochromis_brichardi:0.00000176267459066039)53:0.01405465833024002048)91:0.01632918046887852845,((Neolamprologus_brichardi:0.00861772859483186243,((Neolamprologus_pulcher:0.01423978994723023792,Neolamprologus_splendens:0.00760247912209224483)100:0.00959351524511728969,(Neolamprologus_gracilis:0.00341173898683495109,Neolamprologus_helianthus:0.00692194049616209808)57:0.00268962984471805182)67:0.00391199937075868264)63:0.00313945743839382342,(Neolamprologus_falcicula:0.00000176267459066039,Neolamprologus_walteri:0.02105694242042670597)59:0.00981868045716005090)28:0.00485368069545734360)100:0.01424614810200975148)46:0.00000176267459066039,Neolamprologus_mondabu:0.05754362660107938815)100:0.02096820883924795792)68:0.00616594367284135914,Variabilichromis_moorii:0.07322515812760903298)19:0.00144286554206753907)72:0.01367163574165635234,(Neolamprologus_toae:0.05968740460131138903,(Neolamprologus_sexfasciatus:0.00517218775464287246,Neolamprologus_tretocephalus:0.00446623434279994069)100:0.07091051910944548564)22:0.00354951111757411059)25:0.00339909557397292342,((Neolamprologus_niger:0.03415851339787794733,(Lamprologus_tigripictilis:0.09941400583187959272,((Neolamprologus_olivaceous:0.01106923731013592128,Neolamprologus_savoryi:0.01992974485391252673)99:0.04171115483909060978,(Neolamprologus_marunguensis:0.04099177968272805367,Neolamprologus_longicaudatus:0.02358542835185841943)57:0.00581499331820821506)43:0.00696577219801177133)50:0.00315304009915814889)89:0.01582715688489227598,(Neolamprologus_mustax:0.03121236273599054672,((Neolamprologus_nigriventris:0.01613207403094388259,Neolamprologus_pectoralis:0.02954684877295588041)93:0.01194482939734048746,(Neolamprologus_bifasciatus:0.02899089766467421656,Neolamprologus_cylindricus:0.02592471210191794537)55:0.00591853642853334104)33:0.00000176267459066039)99:0.01742757648285505473)61:0.01351711770901995076)35:0.00340012560161398383)100:0.05141083713263289168,(Eretmodus_cyanostictus:0.03353888518457660145,(Tanganicodus_irsacae:0.04142255943639443910,(Spathodus_marlieri:0.00335265871553505970,Spathodus_erythrodon:0.00961953461260066020)100:0.04966749262030119988)53:0.00618282643580511659)100:0.07548985478848074804)60:0.00487322952768143303)100:0.03412009273670757459)62:0.01412022753565495697)3:0.00227635329565725004)75:0.02139601962597146786)68:0.01921338236597699631)57:0.00690888048492972404)67:0.02677013633195496345,((Teleogramma_depressa:0.00434180167981883507,Teleogramma_gracile:0.00390305535395226019)100:0.39463843713482998909,((Chromidotilapia_guentheri:0.27119525044998560315,(Pelvicachromis_pulcher:0.27827130768517754911,((Nanochromis_nudiceps:0.00935483843990717219,(Nanochromis_parilus:0.00230961374225404715,Nanochromis_teugelsi:0.00244300904464334360)78:0.00769593221979323651)79:0.02461161866726058883,((Nanochromis_consortus:0.00525569327842770068,Nanochromis_splendens:0.00924671031681800759)100:0.02897199505917412779,((Nanochromis_wickleri:0.02758015995469590212,Nanochromis_transvestitus:0.03129858616962623991)85:0.02029550612624343373,Nanochromis_minor:0.11160216779043680146)42:0.00573506549323690544)71:0.01399175272810967985)100:0.41857040429149966476)64:0.05748417822735761573)45:0.03436485114695490839,Thysochromis_ansorgii:0.15062969775726525734)41:0.04153887533703102403)89:0.09410491624079181383)19:0.01459281654141482767)27:0.02702071906199024140,Hemichromis_elongatus:0.29050531607433388581)90:0.05866915650556460415)97:0.12502176452461821743,(Oxylapia_polli:0.45743395627961147554,Heterochromis_multidens:0.23871346368693796558)39:0.04228435299921241147)22:0.02221104346859637674)53:0.06758477915874208652,(((Menidia_extensa:0.21432877016618767652,((Menidia_menidia:0.19753282217213255501,((((Chirostoma_attenuatum:0.07152178264097253102,(Chirostoma_sphyraena:0.00208562299763967928,((Chirostoma_grandocule:0.00099918655939841257,(Chirostoma_patzcuaro:0.00099584972587099551,Chirostoma_consocium:0.00000176267459066039)40:0.00000176267459066039)97:0.00710322655449096597,(Chirostoma_promelas:0.00302061628244472543,(Chirostoma_lucius:0.00100518668141489796,(Chirostoma_humboldtianum:0.00205419925164739177,Chirostoma_chapalae:0.00308632071895514112)46:0.00096315058796617376)53:0.00101265798255675442)64:0.00095630335594597748)94:0.00909863920836482924)99:0.02619009202157324134)70:0.01740768979744063485,(Chirostoma_labarcae:0.09821001205613451301,Chirostoma_jordani:0.05390609219184917117)40:0.01909730386044103043)68:0.01235156804616503387,((Chirostoma_arge:0.00120105053141889321,Chirostoma_contrerasi:0.00564425238298150739)100:0.06666547640993276957,(Chirostoma_riojai:0.04400921483988443766,(Poblana_ferdebueni:0.02191233328453002019,((Poblana_letholepis:0.00098137707149181710,Poblana_squamata:0.00000176267459066039)100:0.00671473051869690262,Poblana_alchichica:0.00123066849649662016)100:0.03305229947043873684)100:0.02469770480344116170)100:0.04766980762334004779)59:0.00752258836159693575)91:0.03113972884606155261,(Menidia_beryllina:0.06361344198428575680,(Menidia_colei:0.03007092087994537796,(Menidia_conchorum:0.00500276244435958733,Menidia_peninsulae:0.00646560056353431053)98:0.01604825592721546151)100:0.04138825354386621580)96:0.03792341577723976759)98:0.07090309696962304731)59:0.05029010357114537672,Labidesthes_sicculus:0.22188018265550146779)50:0.05963123272407925418)99:0.11280822854308952818,(Membras_martinica:0.18126378232200587859,((Atherinella_crystallina:0.23540815384553856471,Atherinella_milleri:0.19385431992904253451)97:0.07643416658308894995,Atherinella_schultzi:0.19664020295842593589)78:0.04734416068280238726)91:0.08842826726050906361)100:0.15385931942658112592,((Floridichthys_carpio:0.33225017299585879460,(Jordanella_floridae:0.27404196413289227952,(Cualac_tessellatus:0.17037933220231835096,(Megupsilon_aporus:0.09448352423703014480,((((((Cyprinodon_salinus:0.00433608209289378099,((Cyprinodon_diabolis:0.01072560204202032547,Cyprinodon_nevadensis:0.00629541213967861786)97:0.00109179972891661962,Cyprinodon_nevadensis_nevadensis:0.00316865392248036294)99:0.00428641683830619218)100:0.01877685208770022915,Cyprinodon_fontinalis:0.01909530158098289923)77:0.00803985898194533276,((((Cyprinodon_macularius:0.00000176267459066039,Cyprinodon_macularius_macularius:0.00109712491561577102)100:0.00563170320511724443,Cyprinodon_macularius_eremus:0.00554739359442238140)98:0.01136520699590321676,(Cyprinodon_albivelis:0.00325342730946751252,Cyprinodon_pisteri:0.00000176267459066039)100:0.01882824585789707669)63:0.00536870602283691031,Cyprinodon_radiosus:0.04385195397094437952)43:0.00326333251325568348)99:0.04123044904953110379,(((Cyprinodon_pachycephalus:0.00458987930818237182,Cyprinodon_eximius:0.00084641593051788344)100:0.03295497160319590252,Cyprinodon_macrolepis:0.03979909067443570397)99:0.02881566148626515944,((Cyprinodon_bifasciatus:0.00107068920914908054,Cyprinodon_atrorus:0.00000176267459066039)100:0.03595430156536071759,(Cyprinodon_nazas:0.04454345888177602825,Cyprinodon_meeki:0.02978367395250415550)96:0.01815920633907674650)60:0.00384311871839344583)67:0.00994713473049672718)26:0.00320342682182706180,((Cyprinodon_alvarezi:0.00963700498613787038,Cyprinodon_veronicae:0.01460374183610652250)100:0.02515207512067406739,((Cyprinodon_rubrofluviatilis:0.02928279646302099984,((Cyprinodon_bovinus:0.00475884891931079718,Cyprinodon_pecosensis:0.00971329445673302719)100:0.02849068150289127410,Cyprinodon_elegans:0.02801577630527085985)98:0.01652580772532701373)73:0.00764204668856062475,((Cyprinodon_bondi:0.00168379365482896975,Cyprinodon_nichollsi:0.00382747647933618469)100:0.03218732609609253198,(Cyprinodon_tularosa:0.02759208051986284868,((Cyprinodon_higuey:0.01626303184825447778,(Cyprinodon_variegatus:0.00724698364955319533,Cyprinodon_variegatus_riverendi:0.00782315338420788708)79:0.00347972338902858202)81:0.00427565195325255425,Cyprinodon_dearborni:0.03262236456495776205)65:0.00101598570412896722)100:0.02063553788776244582)100:0.02510816823392783559)88:0.01714479500155425759)27:0.00299023251531810438)33:0.01126658092651751722,Cyprinodon_artifrons:0.05806778565904575223)41:0.01562719253290144675)100:0.13467210218568392843)38:0.03963586394781972583)97:0.11721972880399081363)98:0.09436340565098622069,((((Xenodexia_ctenolepis:0.61994344731401052062,((Quintana_atrizona:0.24008455938821185449,(Girardinus_metallicus:0.10603608511523777980,Girardinus_creolus:0.04073290002365918816)100:0.12942870504349909822)96:0.09079937262419351951,((Phalloceros_caudimaculatus:0.40659679687320976482,((Cnesterodon_decemmaculatus:0.08512831337260308440,Cnesterodon_hypselurus:0.09140152258098112326)100:0.14064259888131439014,(((Poecilia_wingei:0.03685359646819507534,Poecilia_reticulata:0.05169651834382990585)100:0.09990788388344133686,(Micropoecilia_picta:0.15501006181219614821,((Micropoecilia_bifurca:0.09115723172885120684,Micropoecilia_branneri:0.09967627082366256608)99:0.09310593243657522278,Micropoecilia_parae:0.14554188514422539136)100:0.05509074539619369243)74:0.04234666991455336704)100:0.08114686811164940894,((Pamphorichthys_scalpridens:0.05053375286236799768,((Pamphorichthys_hasemani:0.10815358214137747728,Pamphorichthys_hollandi:0.06700765134962402558)88:0.05855909572143014330,(Pamphorichthys_minor:0.02550675413698099206,Pamphorichthys_araguaiensis:0.02121940407599427758)88:0.01822527267439689666)54:0.02980525368574478071)100:0.14352673918274602705,(((Poecilia_vivipara:0.12125662030932168878,(Poecilia_caucana:0.11385988552746674485,(((Poecilia_sphenops:0.02032344159570304010,Poecilia_catemaconis:0.03171884008745481237)100:0.05982766234508660880,(((Poecilia_gillii:0.01166918483046739882,Poecilia_orri:0.00469378074172740762)100:0.01710785516238498560,(Poecilia_mexicana_limantouri:0.01637854932797422683,Poecilia_sulphuraria:0.01511361795269708201)83:0.00745716647316742049)100:0.01766434371303797143,Poecilia_butleri:0.03925844904221508641)100:0.04672881584444001785)98:0.01991870491251563832,((Poecilia_petenensis:0.03735089316584130209,Poecilia_latipunctata:0.03672771704090994904)98:0.02127238125821318687,(Poecilia_latipinna:0.03238578619962499683,Poecilia_velifera:0.02432646487713580052)83:0.01103341603812813508)99:0.04408928515871478326)95:0.01754254621284836307)100:0.06112054176346021911)45:0.01470826254834254297,(((Limia_caymanensis:0.01542091654784026079,Limia_vittata:0.01073457152971577823)100:0.05236258128934414419,((Limia_pauciradiata:0.01629119985556271022,Limia_dominicensis:0.01544838104032214207)97:0.01285767837063948003,((Limia_tridens:0.00120562729207331098,(Limia_perugiae:0.00120727879681446488,Limia_melanonotata:0.00000176267459066039)47:0.00000176267459066039)100:0.01489768776022426348,Limia_nigrofasciata:0.01081901124650334715)100:0.03458603242771648295)76:0.02018875354262120170)100:0.03023351707739444644,((Limia_versicolor:0.04017544021963871670,Limia_zonata:0.05146848939780181847)97:0.02708360811177620842,Limia_melanogaster:0.07174268549263487782)46:0.00521557299365556139)99:0.05416941854933072370)31:0.01361111384304875167,Limia_heterandria:0.13557477254380698173)28:0.01086868434839624240)57:0.01572179397550759611)100:0.06077218509125980467)65:0.03983939820533480974)80:0.03270260408547429748,((((((((Poeciliopsis_occidentalis:0.00973549168800675289,Poeciliopsis_occidentalis_sonorensis:0.00443509136902305295)94:0.00853465260305606016,((Poeciliopsis_lucida:0.02082462152016661613,Poeciliopsis_infans:0.12484896906858183874)40:0.00587115866942830491,Poeciliopsis_prolifica:0.05451226895972311609)37:0.00858870969111094444)100:0.25879051600542368750,((Poeciliopsis_viriosa:0.10979945879774845319,Poeciliopsis_monacha:0.08866477492324324750)91:0.06269000009842229382,(((Poeciliopsis_turrubarensis:0.04096658665398623944,Poeciliopsis_scarlli:0.05789099363803604931)100:0.17536415210416964050,(((Poeciliopsis_hnilickai:0.01024365082851321114,Poeciliopsis_catemaco:0.00855630519703990539)99:0.01606162085620140012,Poeciliopsis_gracilis:0.01962240631879493338)100:0.08812615467073663111,(Poeciliopsis_turneri:0.06552542511414823079,Poeciliopsis_presidionis:0.05765785567848757259)94:0.04427306800332769104)100:0.07064244498582471221)100:0.16205254881841762993,(Poeciliopsis_baenschi:0.05207534033579538230,(Poeciliopsis_latidens:0.06195802432370194068,Poeciliopsis_fasciata:0.05302572391125720541)94:0.03418351321165536555)100:0.16331200293190897832)47:0.07877033730795895972)56:0.08525452033948426045)100:0.50187156652802411561,Poeciliopsis_elongata:0.11994118711925877419)34:0.06352146817313562843,Poeciliopsis_paucimaculata:0.19481387117870932468)34:0.06787908910550582986,Neoheterandria_tridentiger:0.32041821758042177137)37:0.05679411093425312113,(Pseudopoecilia_festae:0.30399381686963072857,(((((Heterandria_formosa:0.29305691598607652715,Brachyrhaphis_hartwegi:0.24208169061933401678)19:0.03949677487763234018,Phallichthys_tico:0.10340852582423502859)6:0.00464062437444589561,(Xenophallus_umbratilis:0.32258584142061996758,Phallichthys_amates:0.18614153903855532035)38:0.01778413664352061774)33:0.02005876401335042122,(Brachyrhaphis_rhabdophora:0.10635888936749249289,Brachyrhaphis_terrabensis:0.04590317770336059533)100:0.10497221892311729696)36:0.01673324840109986084,Priapichthys_annectens:0.20226365418933184293)82:0.04282335174381291659)65:0.02713867090723371886)13:0.02476704602608765030,((((Priapella_compressa:0.03734509391943886814,(Priapella_intermedia:0.05104773306812605826,Priapella_olmecae:0.04432743782297393631)94:0.01912864953069811497)100:0.17839071523699800981,(Xiphophorus_hellerii:0.03649250194136788999,(((Xiphophorus_clemenciae:0.01274193699565464438,(Xiphophorus_xiphidium:0.02601799229760246718,Xiphophorus_evelynae:0.00909941929042431229)97:0.00822833566205791699)99:0.01956913637135905201,Xiphophorus_maculatus:0.04620880785959013276)89:0.01092305341666869288,Xiphophorus_nigrensis:0.10391666629311836767)50:0.00991771678992254754)99:0.09790894543169237352)64:0.02405437912242725310,((Heterandria_bimaculata:0.03058290127941035511,Heterandria_jonesi:0.00770853095969088221)100:0.21825116645249950653,(Scolichthys_greenwayi:0.22984812444717445112,Carlhubbsia_stuarti:0.15575689971277090207)55:0.03311187804675781027)38:0.01496117485196456903)51:0.02090014331449455837,(Belonesox_belizanus:0.36560758282525523954,((Heterophallus_milleri:0.02881782689515915291,Heterophallus_rachovii:0.02380495985900485736)100:0.33731213337289461807,(Gambusia_wrayi:0.10997577494741460347,((Gambusia_atrora:0.10052022535867637110,Gambusia_vittata:0.09362021047198584855)40:0.01168494713813013168,(Gambusia_hubbsi:0.12778149511808806205,(Gambusia_affinis:0.03211974284386480416,Gambusia_holbrooki:0.03387520995836353077)100:0.05403155395252648430)48:0.00825978655697780575)45:0.02100347819516658679)97:0.06180275537524983903)98:0.09195441965140560558)98:0.14744180707896775706)49:0.02611929714740337735)41:0.02968802394402573347)35:0.03401183812464740608)97:0.20728613477588461733)100:0.40072487107081739000,(Anableps_anableps:0.40314828811188213598,(Jenynsia_lineata:0.36304265775712440956,Oxyzygonectes_dovii:0.29895066913275963882)100:0.14532168886880927894)44:0.03365467054993320078)65:0.04739231506504051894,((Valencia_hispanica:0.15351887121446594620,Valencia_letourneuxi:0.13388466228805645519)100:0.29141711135675385513,(Aphanius_apodus:0.33876907394037164556,((Aphanius_iberus:0.24900912669632827012,((Aphanius_vladykovi:0.03307616846162955704,(Aphanius_isfahani:0.06197675974092856088,(Aphanius_persicus:0.03295141721150508102,Aphanius_sophiae:0.03669173729440346199)65:0.01291009258009854707)72:0.01980543880931236178)100:0.23032843661490398635,(((Aphanius_danfordii:0.08860923625440622620,(Aphanius_villwocki:0.09727647028707286925,(Aphanius_anatoliae_anatoliae:0.02570449475982148138,((Aphanius_anatoliae_splendens:0.01398063770984228280,Aphanius_anatoliae_sureyanus:0.01670571604396981630)99:0.01229524795622144277,Aphanius_anatoliae_transgrediens:0.06720054870069203101)49:0.00931100901150486680)100:0.07361008457954325290)78:0.01471350414119926633)95:0.04581510628376948141,Aphanius_asquamatus:0.25627354807696561823)88:0.04268574162197519978,Aphanius_fasciatus:0.21111362732942820508)55:0.02481642102884224280)86:0.05083624290259685513)87:0.05304349541468059148,(Aphanius_mento:0.22358771928731097089,(Aphanius_sirhani:0.08307442924011021468,((Aphanius_ginaonis:0.02955919761731510415,Aphanius_dispar_dispar:0.01495018808642055788)99:0.05363188634365050750,Aphanius_dispar_richardsoni:0.05408742041865349792)74:0.03487294319945478255)100:0.15716269008309974309)98:0.10405158923622546963)82:0.05767309649906537244)50:0.06177459533573835537)47:0.06237264031905502548)43:0.04492844527338566568,(Fluviphylax_pygmaeus:0.66429475484909739080,(Orestias_agassizii:0.07861845539828656027,(Orestias_imarpe:0.01033251795602397639,Orestias_gilsoni:0.01285945497485466854)100:0.08536644985292629606)100:0.41262716014950046040)50:0.08587274263575617184)88:0.04346348873670437263)94:0.12976709036155500976)49:0.08025422189232343795)10:0.04281739712717221036,((((((Sphaeramia_orbicularis:0.30274323750520859555,Fowleria_marmorata:0.36333499385894563183)72:0.07477773825460388990,((((((((Rhabdamia_cypselura:0.16520154841303932858,Pterapogon_kauderni:0.09381981169380104735)57:0.16377549005400438009,Zoramia_fragilis:0.00000176267459066039)99:0.28458531804452014136,Apogon_exostigma:0.05540825605765980350)41:0.04611082869481859109,(Fowleria_variegata:0.00754753656693880632,(Fowleria_isostigma:0.35416492327071569246,Fowleria_aurita:0.32413100965722335678)89:0.10964986707370601160)93:0.15371957417221693287)37:0.16767696233948548024,((Cheilodipterus_macrodon:0.05446165647776963353,Glossamia_aprion:0.16211251264605891498)95:0.11951571342848454904,((Cheilodipterus_artus:0.04302256490710602843,(Apogon_trimaculatus:0.08942819922324835025,(Apogon_kallopterus:0.10762944873237852150,(Cercamia_eremia:0.00426004554073314539,Gymnapogon_urospilotus:0.22819805644204416151)99:0.14537583580553717355)99:0.14984266612749133629)35:0.04224912938312502808)74:0.08842384025574918927,(Cheilodipterus_isostigmus:0.02301028990387612647,(Pseudamia_gelatinosa:0.30158488403838284997,(Archamia_fucata:0.01863077209785434646,Archamia_biguttata:0.08400475230266055138)99:0.06101076376295628406)98:0.04813290180048261646)97:0.20117724099446313013)34:0.02270671879000988810)32:0.02518427702596218176)10:0.03403525230802209589,((Apogon_cookii:0.30994639374584775915,((Apogon_flagelliferus:0.12849924744049298631,Apogon_semilineatus:0.16520847853179526643)63:0.04295047584142988595,Apogon_nigrofasciatus:0.16354636269081143651)3:0.00000176267459066039)40:0.03537902794827398750,Apogon_doederleini:0.17982384005511925507)32:0.01635015126660941537)10:0.03178478523371199710,Apogon_holotaenia:0.34597457374101048266)23:0.20187452817090165147,Apogon_angustatus:0.25003182394742873296)18:0.09070334951855212047)18:0.04701547538312002283,Apogon_maculatus:0.47246555475243495748)25:0.11042815855831304706,((Clinus_cottoides:0.16720201687982610972,Muraenoclinus_dorsalis:0.26159305962233303511)98:0.12558714969471096445,Clinus_superciliosus:0.25802052993006996395)100:0.48578799800900057670)1:0.05444195180387049332,((Gymnodraco_acuticeps:0.11129093200409018172,((Gerlachea_australis:0.08660687343164698293,(((Artedidraco_skottsbergi:0.01455881872693415811,(Histiodraco_velifer:0.02482264180588991329,((Pogonophryne_marmorata:0.00323121362958598722,Pogonophryne_barsukovi:0.00323073542581910718)59:0.00105362290563603268,(((Pogonophryne_cerebropogon:0.00215043988629766598,((Pogonophryne_orangiensis:0.00106794371863882360,(Pogonophryne_macropogon:0.00107116730096535966,(Pogonophryne_mentella:0.00213871910144558877,Pogonophryne_eakini:0.00000176267459066039)21:0.00000176267459066039)54:0.00106667361116257820)20:0.00106981883576027299,Pogonophryne_squamibarbata:0.00000176267459066039)13:0.00000176267459066039)42:0.00106256934094339762,(Pogonophryne_stewarti:0.00103760448201358044,Pogonophryne_immaculata:0.00320276019218325219)84:0.00320690844095842089)40:0.00107406217906839637,Pogonophryne_scotti:0.00864058826013747866)4:0.00000176267459066039)78:0.01060514835221861539)94:0.01456708427768340502)97:0.06083204644137447936,((Notothenia_rossii:0.05355359612019713195,((Notothenia_coriiceps:0.04714147615273773378,Paranotothenia_magellanica:0.12061911014864371006)64:0.02085613960325519156,(Notothenia_microlepidota:0.15017085811551927899,Notothenia_angustata:0.04994615363232648986)99:0.05690310368097890870)61:0.00606807777290699953)100:0.07808512296182867907,((Pleuragramma_antarctica:0.23035830941381227333,(Patagonotothen_tessellata:0.16723440478476644944,(Trematomus_scotti:0.13435627688545861980,((Trematomus_newnesi:0.10695854868335814836,Pagothenia_borchgrevinki:0.06977613150315913060)54:0.01587087212315169138,(((Trematomus_bernacchii:0.00083509659375262781,Trematomus_vicarius:0.00474701950043701811)100:0.03475087349703116085,(Trematomus_nicolai:0.04732123233640822546,((Trematomus_pennellii:0.04952254538452362936,Cryothenia_amphitreta:0.04924938191187262782)59:0.01248724030878946636,(Trematomus_tokarevi:0.04592505018874566691,(Trematomus_hansoni:0.03487372220689702318,Trematomus_eulepidotus:0.04781654286296756207)41:0.01071842748917531916)18:0.00707246899496109891)26:0.00791050844794480290)28:0.00870959645250461018)36:0.00653627824329901241,(Trematomus_lepidorhinus:0.00552061735559386188,Trematomus_loennbergii:0.00265635289774103110)100:0.03681011589524135763)55:0.01190645613390046292)63:0.02180037253085365459)63:0.04234666140424619279)100:0.24469627854773823294)45:0.00723212186750867535,(Aethotaxis_mitopteryx:0.09937440555551771415,(Dissostichus_mawsoni:0.06525835590294055966,Dissostichus_eleginoides:0.06475947140072177421)98:0.04028602723546926417)54:0.00884214141347477306)96:0.05286368611874477097)100:0.06111299068920081345)76:0.02733507685779320087,((Bathydraco_marri:0.03553142377694328696,Akarotaxis_nudiceps:0.02315523848735346821)96:0.01684027013047732432,Racovitzia_glacialis:0.02743063599185480150)100:0.05404710508460949109)44:0.01414802322663257471)34:0.00542764563539568402,((Dacodraco_hunteri:0.02776619557277769726,((((Chionodraco_myersi:0.00908318078006309197,(Chionodraco_rastrospinosus:0.00824293901389845304,Chionodraco_hamatus:0.02258831445902810542)60:0.00342885226460261632)92:0.00412948488819663354,Chaenodraco_wilsoni:0.02007919138556857330)97:0.01253355457007548392,(Channichthys_rhinoceratus:0.02869351777936923753,(Chionobathyscus_dewitti:0.01252782033853421309,(Cryodraco_antarcticus:0.00358626947894048797,Cryodraco_atkinsoni:0.01047892487578345501)97:0.00958147283896597715)60:0.00309789423315304496)57:0.00546984327825869587)17:0.00026307643132396531,Chaenocephalus_aceratus:0.02410274787314038020)89:0.01902832884992636947)47:0.00629692146304421368,((Champsocephalus_esox:0.01143314489616441970,Champsocephalus_gunnari:0.00606263103741311749)100:0.09685157122144842645,((Neopagetopsis_ionah:0.03575308068060811806,Pseudochaenichthys_georgianus:0.04076560222643527132)98:0.02297025143113347323,(Pagetopsis_maculatus:0.02776420686813087235,Pagetopsis_macropterus:0.02503862154967497963)56:0.00675664698594058769)87:0.01009673584713306933)85:0.00758612829437500281)89:0.08927553419326836326)51:0.04313983397099330208)100:0.54867777873252177034,((Scophthalmus_maximus:1.94467698766996210935,(Apogon_aurolineatus:0.00000176267459066039,Pempheris_vanicolensis:0.00000176267459066039)100:1.23803089902427521096)10:0.29873042012245265564,Aplodinotus_grunniens:0.29363605304050721667)2:0.09783802588405798861)0:0.05312986228263125860)0:0.01582121023939334070,Ditrema_temminckii:0.51258759971290668300)0:0.02221123788903668628)0:0.01565689190640699377)0:0.01691045729148253629,(((Variola_louti:0.12569896124752596989,Variola_albimarginata:0.08526780035136262426)100:0.25047142698948782202,((Plectropomus_areolatus:0.10891100824944094372,(Plectropomus_maculatus:0.05676915728627582314,Plectropomus_leopardus:0.03800896737930527236)77:0.03116761835141994980)44:0.07143121632001506183,(Plectropomus_oligacanthus:0.04513443594736655379,Plectropomus_laevis:0.08083546249121287275)100:0.12806163983983032129)100:0.34631103896327458669)28:0.04014595603268356072,((((Epinephelus_radiatus:0.04097378389177951802,(Epinephelus_epistictus:0.04264030466172111233,Epinephelus_amblycephalus:0.07204919684791807888)74:0.02529133730350844461)96:0.07291403765193052444,((Anyperodon_leucogrammicus:0.11716778093107545200,((Epinephelus_polyphekadion:0.21464317165187810077,(((Epinephelus_moara:0.00000176267459066039,Epinephelus_bruneus:0.00000176267459066039)100:0.09824386460079434502,(Epinephelus_latifasciatus:0.07631928600836641874,(Epinephelus_lanceolatus:0.13814342141614516235,((Epinephelus_coioides:0.06160688310408572038,Epinephelus_malabaricus:0.03653188070273432481)87:0.02043127943861855825,Epinephelus_tukula:0.07537533067475687965)79:0.02284708359606149250)36:0.01734019604327284581)22:0.01701727676348541163)17:0.01104997859657432142,Epinephelus_daemelii:0.11803403405248195923)22:0.02012562570197071188)12:0.01155009485287614504,(Epinephelus_fuscoguttatus:0.09650602974105744469,Cromileptes_altivelis:0.14632420496471526539)23:0.01318809107278181431)23:0.01650304059989216032)42:0.02788537040171513992,Epinephelus_coeruleopunctatus:0.06918678550800747007)96:0.06844829092087409417)52:0.02354730232765294209,((((Epinephelus_tauvina:0.12065443999812879017,(Epinephelus_quoyanus:0.09544320474792639120,Epinephelus_fario:0.08718765456370002054)100:0.04207004371998603054)99:0.08640908485240972325,(Epinephelus_spilotoceps:0.11996905338648120032,((Epinephelus_longispinis:0.10337364729799744523,Epinephelus_bleekeri:0.08174180315775958494)99:0.05403399652450165125,(Epinephelus_undulosus:0.07209196691209850127,(Epinephelus_flavocaeruleus:0.07047613833499753733,Epinephelus_areolatus:0.08123414620583524481)74:0.02677425396510426867)99:0.04584859265582907212)70:0.01837563730684548327)29:0.00626198512138021780)38:0.02920484135529152758,(Epinephelus_fasciatus:0.12879439764007008518,(Epinephelus_melanostigma:0.00000176267459066039,Epinephelus_hexagonatus:0.00824529590645087934)100:0.10646996310735881708)89:0.06029315487151316183)67:0.00914262846842211867,(Epinephelus_sexfasciatus:0.12300154623601730619,((Epinephelus_awoara:0.07935113730149138955,Epinephelus_diacanthus:0.07631811578037336896)22:0.01731993784157542446,(Epinephelus_akaara:0.07187818898183445659,Epinephelus_chlorostigma:0.07520020836607642367)20:0.01588780435524650078)38:0.01193252227970388601)96:0.04571950811681779053)84:0.02052574814573513567)97:0.10894806510804129485,((Cephalopholis_miniata:0.07594856826855325704,(Cephalopholis_urodeta:0.06409236564156521598,Cephalopholis_sonnerati:0.03751433875649807376)55:0.02001385267612842619)100:0.12198264957129831276,((Cephalopholis_pachycentron:0.16001113270806158195,Cephalopholis_formosa:0.09174805905927138627)100:0.09193853126908300977,(Aethaloperca_rogaa:0.11634731156530769836,Cephalopholis_argus:0.10733399396398858594)95:0.06539967638090536661)92:0.07010329885358750068)69:0.04805623898488096263)95:0.10951772344435672935)23:0.08686816550897277778)0:0.01601441108606363115,(((Cheimarrichthys_fosteri:0.42413006737014719016,Siniperca_chuatsi:0.13924711909865403192)13:0.07965070871735530078,((((((((((((Scomberomorus_regalis:0.00234202553918322386,Scomberomorus_maculatus:0.00000176267459066039)100:0.01619049899335846601,Scomberomorus_brasiliensis:0.02274932043692589473)90:0.02730622611356323115,(Scomberomorus_concolor:0.03164482537784759492,Scomberomorus_sierra:0.02760609789832899758)90:0.01688257392346779323)81:0.03236283636843428901,Scomberomorus_tritor:0.08429703262689754173)99:0.10205027574531713719,(Scomberomorus_guttatus:0.19110501538949553613,Scomberomorus_cavalla:0.12518104800849053082)49:0.01482553227169381219)98:0.12035228010852624947,((Thunnus_thynnus:0.11987702437933656086,(Auxis_thazard:0.06913028805330272142,Auxis_rochei:0.05736930331521551685)100:0.17948115140364356845)67:0.05620431759311284720,Sarda_sarda:0.18124014394302692676)60:0.04346399053953117764)38:0.02147453554151004113,(Pomatomus_saltatrix:0.31811442898232333842,(Scomber_japonicus:0.15722335776577150979,Scomber_scombrus:0.08735829665760576346)100:0.21377418158860442277)66:0.09622957360811273952)10:0.01787130368168465822,Acanthocybium_solandri:0.25625522531515176894)41:0.00793317334306683705,(Pampus_cinereus:0.09247108250080472713,((Pampus_punctatissimus:0.00000176267459066039,Pampus_echinogaster:0.00380826273835903947)100:0.49431249171713848023,Pampus_chinensis:0.09849090971210641698)40:0.03704126832016845644)74:0.36584700958764876377)28:0.03056895049589949606,((((((Assurger_anzac:0.18569710978745784802,Lepidopus_altifrons:0.15808590637817312996)63:0.04868742609340820371,Lepidopus_caudatus:0.20283076735670838020)38:0.03792372863905828229,(Psenopsis_anomala:1.66427882840308560830,Trichiurus_lepturus:0.22209341117670822197)8:0.27825457058381247721)7:0.04774130137328946172,(Benthodesmus_simonyi:0.16173943835639270628,Aphanopus_carbo:0.11934951810274004036)79:0.04068024252070812780)7:0.11930278695624185747,Neoepinnula_americana:0.28781680191156738813)0:0.02907793447549006371,((Lepidocybium_flavobrunneum:0.25783361071853966306,Ruvettus_pretiosus:0.17800561873745354702)55:0.04811346369074340906,((Rexea_solandri:0.13742904679468490037,(Nealotus_tripes:0.28280176187640265750,((Diplospinus_multistriatus:0.29408268010886323696,Paradiplospinus_antarcticus:0.50225047518074816821)100:0.30885948714901861889,Promethichthys_prometheus:0.20839330039421921636)48:0.07671939310947488433)44:0.06662933461997734563)41:0.05434360030001705172,((Gempylus_serpens:0.36291967806017250142,Thyrsites_atun:0.41652697676336708499)50:0.05684947868585663033,Nesiarchus_nasutus:0.20349384611846940696)14:0.04428227277423945479)15:0.05783535640358249130)3:0.00143641170048639986)10:0.03238437309362082045)10:0.00110819817488560265,Arripis_georgianus:0.52020117818813316202)37:0.13483848846757739981,((((((Enneacanthus_gloriosus:0.01689513313896362154,Enneacanthus_obesus:0.02292059599596956571)100:0.08242389427754248654,Enneacanthus_chaetodon:0.09687421044798026626)100:0.08331454757617634543,(Centrarchus_macropterus:0.14164792259404221375,((Pomoxis_nigromaculatus:0.13617453344530774939,Pomoxis_annularis:0.14265922233199390678)95:0.07577440851456122839,(((Ambloplites_ariommus:0.03361880117017251868,Ambloplites_rupestris:0.02232302307484456197)98:0.07247792142734425791,(Ambloplites_cavifrons:0.05484321931907124026,Ambloplites_constellatus:0.06717947388825959432)56:0.01159336983105416392)97:0.08421707534779739046,Archoplites_interruptus:0.11814846895483617495)67:0.02378997320185708211)25:0.01223069103520249383)27:0.01346886649544582794)67:0.03875396961555044317,(Acantharchus_pomotis:0.19867535380742298856,(((Micropterus_punctulatus:0.00156648719640541285,Micropterus_dolomieu:0.00933570409605571395)100:0.09094854012472826377,(Micropterus_cataractae:0.05830944246981561996,(Micropterus_coosae:0.06369480674773991824,(((Micropterus_salmoides:0.02636688024817254034,Micropterus_floridanus:0.03014396673569966906)94:0.02093952454387629572,Micropterus_treculii:0.05862034140844607499)79:0.01874028117608132413,Micropterus_notius:0.06432489302028479572)47:0.00838507556664367000)37:0.00925050584594604673)56:0.02474954807796407238)100:0.14815031325594008038,(((Lepomis_symmetricus:0.03397432194312826476,Lepomis_cyanellus:0.02220612459180623319)100:0.08841082776079257910,Chaenobryttus_gulosus:0.09048933244465698356)100:0.11932435768789814712,(((Lepomis_microlophus:0.03302050325330883884,(Lepomis_punctatus:0.04233592171104696295,Lepomis_miniatus:0.04501537431068382972)98:0.02305120148480593531)100:0.11878957116813214367,((Lepomis_auritus:0.06490805978827440814,(Lepomis_megalotis:0.03478536737872429258,Lepomis_marginatus:0.06399369998406609361)99:0.03592268960632017533)100:0.13822768871146146052,Lepomis_gibbosus:0.08946354228288661004)45:0.01287677027403713766)100:0.13055370400971588740,(Lepomis_humilis:0.06509208300316837725,Lepomis_macrochirus:0.06684050064853895579)100:0.10340550344108143110)48:0.02742323144438425092)100:0.20277776755076265536)46:0.02983835766612473461)27:0.02109692326066429230)72:0.11764012620495128969,((((((Etheostoma_cinereum:0.19857819961666348108,((Ammocrypta_bifascia:0.15321125872565355408,(Ammocrypta_clara:0.07189533702424834305,Ammocrypta_pellucida:0.10598572412764484252)61:0.02258136279353372550)98:0.06311335431471014334,((((Etheostoma_camurum:0.02887041018626796893,Etheostoma_chlorobranchium:0.01493454680989480046)98:0.02502640537359668987,Etheostoma_maculatum:0.03844273530928096760)100:0.05796771623569176024,(Etheostoma_jordani:0.11885600396509182763,(Etheostoma_tippecanoe:0.00947782665601271286,Etheostoma_denoncourti:0.06082127657658538411)100:0.10128259218111398954)68:0.02405871545498063438)72:0.02175332781370337490,Etheostoma_juliae:0.19285086364883419208)99:0.04769604978031947995)24:0.00550122516094473518)34:0.01839088934584988400,(((Etheostoma_sagitta_spilotum:0.06985560592902161081,Etheostoma_variatum:0.06689693245802516564)100:0.08088968801675265607,((((((Etheostoma_obeyense:0.07473916478231876215,((Etheostoma_smithi:0.02290535698478127907,Etheostoma_striatulum:0.03084593159014259456)100:0.03588318432423536625,Etheostoma_derivativum:0.06753199075372637306)89:0.02183765941723696605)100:0.08156656715546314884,((Etheostoma_barbouri:0.19407149045637200890,Etheostoma_kennicotti:0.11639617079326126936)25:0.01508745416832376826,(Etheostoma_flabellare:0.04142508257686012413,Etheostoma_percnurum:0.10692923141042827428)100:0.06074956655441215991)18:0.00920117897431655014)32:0.01209203938010307636,Etheostoma_basilare:0.17844352731015292179)89:0.04777786057531765301,((((Etheostoma_uniporum:0.02676121465051082421,Etheostoma_spectabile:0.01748162888330608358)100:0.04474230451772145956,((Etheostoma_ditrema:0.04724685620155265331,(Etheostoma_swaini:0.00075707215659273110,Etheostoma_nuchale:0.00219169165882248567)100:0.07409325850684246595)100:0.04163960491215348836,((Etheostoma_asprigene:0.04087305727491637175,Etheostoma_collettei:0.03166500522938404255)98:0.02758093523247596643,Etheostoma_caeruleum:0.06825896396568691127)70:0.00953018364439453362)72:0.00670023261982477959)100:0.06962682620798907318,(Etheostoma_boschungi:0.06032277958516192584,Etheostoma_tuscumbia:0.10306109479494218739)100:0.05536437397768864677)74:0.01922145450481854700,((Etheostoma_pallididorsum:0.13209493692100637841,Etheostoma_cragini:0.14694413792869390201)46:0.01256612007336936794,(((Etheostoma_australe:0.17379751065526322096,((Etheostoma_pottsii:0.05442053879258346161,((Etheostoma_lugoi:0.01321814621466211453,Etheostoma_segrex:0.01017255399173785067)100:0.02739623865743079020,Etheostoma_grahami:0.03261940226717466307)81:0.01524514908888381474)100:0.05326438944417814791,Etheostoma_lepidum:0.11797258638279702059)98:0.04073900447046972101)84:0.02085763282213799058,(Etheostoma_exile:0.10098897506473164531,Etheostoma_luteovinctum:0.07652498360061699145)99:0.03312609792521074847)49:0.00910471720539370206,(Etheostoma_radiosum:0.07372343060451852503,(Etheostoma_spectabile_pulchellum:0.02363926122376634742,(Etheostoma_whipplei:0.02335785993409028247,Etheostoma_artesiae:0.02577985469688941905)61:0.01095836334366038362)100:0.07593287743485034602)100:0.06302170449590649426)57:0.01220776410080864904)42:0.00492152569315429736)96:0.02668567171692270978)38:0.01495445315604770897,((Etheostoma_trisella:0.21222815106300221832,Etheostoma_histrio:0.15680951837230755475)37:0.05203409175990121593,((Etheostoma_microperca:0.20609217189795450698,((Etheostoma_punctulatum:0.13016917318120638236,(Etheostoma_spectabile_squamosum:0.05740913573153767074,((Etheostoma_bison:0.04668979583925676746,(Etheostoma_lawrencei:0.03339704755434680372,(Etheostoma_tecumsehi:0.00765311085305710702,Etheostoma_kantuckeense:0.01139263951034407731)100:0.02580688107450215471)80:0.00957884757698224641)97:0.02027800290029330155,(Etheostoma_spectabile_spectabile:0.02727740640243068657,Etheostoma_burri:0.02561815739989023455)100:0.01909795971040727930)76:0.01916122791293318334)100:0.05676140971956279008)38:0.01578320517966603706,(Etheostoma_fonticola:0.06938158030811682198,Etheostoma_proeliare:0.02824147116528367973)100:0.13565154344941535158)52:0.02091860476710595551)92:0.05518023126893207425,((Etheostoma_simoterum:0.15206712635188010529,Etheostoma_zonale:0.14711754953137887170)22:0.02082071467392773365,Etheostoma_blennioides:0.13407711402910424403)10:0.00889578211152067776)16:0.01888298751268442655)9:0.00394350118177874947)7:0.01288784651730845096,((((Etheostoma_hopkinsi_binotatum:0.18804325613202776957,(Etheostoma_edwini:0.16215238417193528209,(Etheostoma_hopkinsi_hopkinsi:0.04811142918808541946,Etheostoma_fricksium:0.06628065452454183493)100:0.07782699575385040869)93:0.06656572821239933879)48:0.02003669938982323082,((Etheostoma_parvipinne:0.16986960437264683743,Etheostoma_okaloosae:0.17466131961662320626)40:0.00929634935730904582,Etheostoma_collis:0.25726508568992029202)8:0.01067421310726358180)24:0.03012155374470940061,Etheostoma_fragi:0.20879247860625615996)8:0.01641356483671277747,((Etheostoma_oophylax:0.18294445560523514560,Etheostoma_squamiceps:0.16547045685677966298)66:0.05296776063610512952,((Etheostoma_jessiae:0.15032692163593358581,Etheostoma_chlorosomum:0.15589186578586755605)53:0.03499789226637263495,(Etheostoma_olmstedi:0.01297871904580960578,Etheostoma_vitreum:0.02267407972034682709)100:0.12943278606438493594)14:0.00908060795540042466)3:0.00298227212011217260)0:0.00604247628643264124)39:0.01907817917319818909)92:0.04551750051668457253,(Percina_roanoka:0.12715703310726700348,((((((Percina_burtoni:0.01512805668634271787,((Percina_macrolepida:0.00549430762023616487,Percina_suttkusi:0.00326181886487927550)58:0.00207929128239074798,(Percina_caprodes:0.00643859268387865762,(Percina_carbonaria:0.01309839462317059951,((Percina_bimaculata:0.00637811658638984345,(Percina_austroperca:0.00543578215534515526,Percina_kathae:0.00218870801437029763)77:0.00318622706317514982)44:0.00106352562536209116,Percina_fulvitaenia:0.00212509204118141318)27:0.00106091882847710603)18:0.00000176267459066039)26:0.00105576318015882160)100:0.01296444846656680076)93:0.01141897072924184624,Percina_jenkinsi:0.04116674004720489999)98:0.01905814331598863071,Percina_rex:0.04931603925676822375)99:0.04949218454351317353,(Percina_aurantiaca:0.08357072387187769458,Percina_evides:0.06505803467402604401)98:0.04959915065840685144)36:0.00616704421102613493,Percina_maculata:0.09887681794371054600)56:0.01758566556531336242,Percina_macrocephala:0.11161999838644916239)99:0.04450325116859083635)99:0.06699727138602372400)43:0.00705716627653594185)39:0.04179697611633900745,Crystallaria_asprella:0.16796820480076987692)88:0.06315001038699212532,(Sander_marinus:0.15128764368340202884,(Sander_vitreus:0.86594713324541794375,Perca_flavescens:0.13242578545253402833)32:0.04627406652345338739)60:0.05149957484314113959)60:0.13281514206643549314,(Ophiodon_elongatus:0.38138798674633583863,Caffrogobius_saldanha:0.34272746267287707012)13:0.03200952312215376433)4:0.06054664216846558644,((((Oplegnathus_fasciatus:0.08876155815794595227,Oplegnathus_punctatus:0.11367982962574778305)100:0.20369387874122729665,((Kyphosus_cinerascens:0.03469046312032884882,Kyphosus_vaigiensis:0.01582259452080006859)100:0.10018369217008930849,(((Girella_leonina:0.06876510910276352473,Girella_punctata:0.05853271889888276430)48:0.01894665697936245841,Girella_mezina:0.07634339228636256858)57:0.01670366723712308349,(Girella_elevata:0.06491686747622017750,(Girella_nigricans:0.08382314204117986833,Girella_tricuspidata:0.06476815602329955013)79:0.01534994532283750004)93:0.04117969866016187030)100:0.27687963783518892980)49:0.02617649657438831517)6:0.00139203782791775307,Kuhlia_mugil:0.34864360100970753775)28:0.01643685422236385724,(((Terapon_jarbua:0.24067083483173509761,Terapon_theraps:0.28179657193716123320)100:0.29728223302079614943,(Scorpis_lineolata:0.16473586433092129999,Labracoglossa_argentiventris:0.26771239627339060707)97:0.18938794586736371062)10:0.03149833690887485910,Microcanthus_strigatus:0.32560881594441454245)3:0.00000176267459066039)39:0.09473850992879565003)0:0.01661324791529706496)0:0.02156118870749684086,Stereolepis_gigas:0.16085518744466079100)0:0.00967236206511593380)0:0.01664282628239716760)0:0.01757739164144692806,(((Sphyraena_barracuda:0.83691609493227259176,Nematistius_pectoralis:0.33655025625316675741)15:0.07342172609019863294,(Xiphias_gladius:0.24966077391067684199,(Tetrapturus_belone:0.00000176267459066039,(Tetrapturus_pfluegeri:0.00000176267459066039,(Tetrapturus_angustirostris:0.00494350169005048842,(((Tetrapturus_albidus:0.00336950204655743013,Tetrapturus_audax:0.00282254781294729532)98:0.02588365211008124625,(Makaira_indica:0.03616452637561834438,(Istiophorus_platypterus:0.04334344723654243864,Makaira_nigricans:0.02847529594273310802)61:0.01683477788276695197)36:0.00544232595105807043)61:0.02726980293425862109,Tetrapturus_georgii:0.01312398406654418856)46:0.02355704742237583679)26:0.00128992896295056073)37:0.00248026223324973290)100:0.24502894739707753446)56:0.06873903250720370117)2:0.05143283005568655142,((((Parachanna_obscura:0.12990317219757177569,(Parachanna_insignis:0.23467023733295891419,Parachanna_africana:0.35193257165001584630)69:0.05905666984636068900)100:0.65945854634461453792,Gazza_squamiventralis:0.98600498624428145611)13:0.07523420644334187501,(Trichopsis_vittata:0.54064257299846008031,Anabas_testudineus:0.41430084804190586700)33:0.09792980419717503549)2:0.06586696784745678379,((Channa_pleurophthalma:0.14826652763684555758,Channa_micropeltes:0.15531267816209454180)100:0.21471107678528864038,(((Channa_striata:0.30420663969043942876,((Channa_asiatica:0.18933679890074167629,(Channa_argus:0.08853680538040027692,Channa_maculata:0.04890225407555098130)100:0.22322831125660114182)95:0.13035406063257359843,(Channa_marulius:0.07814452582624543953,Channa_marulioides:0.08045634305035982703)99:0.14328954913081495826)48:0.02108789136231249695)69:0.04288627003844920627,(((Channa_orientalis:0.05700601050372083173,Channa_gachua:0.10231651419887252685)100:0.17263493257077797138,(Channa_bleheri:0.15751062137294019605,(Channa_burmanica:0.10640457603292444166,Channa_barca:0.10777080549378953434)94:0.03999161956375014981)85:0.06513681079383193406)100:0.21792169964343074962,(Channa_punctata:0.17843311274713985481,Channa_panaw:0.20001017962036474196)100:0.15726304575581989686)100:0.14253219435445768637)76:0.07385660211804553898,(Channa_lucius:0.09638684299203557904,Channa_bankanensis:0.15202575467661968989)100:0.28679886017491856842)69:0.05055223820230221599)63:0.05492401881364005617)5:0.05026335821803826209)1:0.01093548020339609253)0:0.03224824943065316557)1:0.02602048148028898722,(Lateolabrax_japonicus:0.28697517605494726745,Pseudolabrus_japonicus:0.35011157612232490788)21:0.13136057053817115636)60:0.12180952062816859105)63:0.06633730089524166040):0.45000000000000001110,Polymixia_japonica:0.45000000000000001110);

Figure 2: ND4 tree

((Pachycara_crossacanthum:0.61694027679858587376,((((Coryphoblennius_galerita:0.49542947277246024429,Salaria_fluviatilis:0.45256549898616621475)97:0.34452790453298859363,Opeatogenys_gracilis:1.88324171345246149656)5:0.18443770563888567393,(Paranthias_colonus:1.38010011175825098029,((((((Nuchequula_nuchalis:0.04734551881037937088,Nuchequula_decora:0.07504393151234220460)99:0.04714408619307060289,Nuchequula_pan:0.10757057909740254387)100:0.12125556551635774394,(Equulites_elongatus:0.17337096769899396076,(Equulites_rivulatus:0.15593956089098962026,Equulites_stercorarius:0.17839485787208642087)40:0.04556702936076434396)100:0.08654222913807092799)100:0.10208069862874315226,Eubleekeria_splendens:0.30399443582338375736)26:0.01549783741564142968,((Secutor_indicius:0.28797375604558500539,(Secutor_megalolepis:0.12643908650695043216,Secutor_hanedai:0.13422616524762104318)95:0.04654157778457277544)97:0.13290643126909912342,((Gazza_achlamys:0.13578659520975178143,Gazza_dentex:0.14794547870826171843)94:0.07008313459295140158,Gazza_minuta:0.30907930540033601297)82:0.10720526476411067207)32:0.03200117993609066097)46:0.03780080717000298962,Leiognathus_equulus:0.32470740488776445032)93:0.38143894946681561331)7:0.05191452206844533040)4:0.09426857853682353572,(((((((((Etheostoma_basilare:0.12605294305773873109,(Etheostoma_percnurum:0.12009653889631990986,Etheostoma_kennicotti:0.13221747025039518042)15:0.00616574288680428635)12:0.01101252443259422009,(Etheostoma_barbouri:0.13444338871005362401,(Etheostoma_virgatum:0.08525655862430980836,((Etheostoma_derivativum:0.04233277125408901886,(Etheostoma_striatulum:0.01963135645982711994,Etheostoma_smithi:0.02466802982529325144)100:0.03952959149223182755)89:0.01722947308198225294,Etheostoma_obeyense:0.07615961554422387425)93:0.01141980961622864575)99:0.07143897609476845822)13:0.01607899139059043581)20:0.01549743074206635582,(Etheostoma_zonale:0.14806649543790262324,Etheostoma_squamiceps:0.15114070520917358142)10:0.03208511611644078443)39:0.04524782769373283248,Etheostoma_caeruleum:0.12887625802182844925)50:0.05330329883941348212,Percina_caprodes:0.26692150660488844771)79:0.04324750023911801827,((Sander_lucioperca:0.15636404080210952738,Zingel_streber:0.20350601436733106731)79:0.07842132091020159734,(Perca_fluviatilis:0.16718438204888982912,(Gymnocephalus_schraetser:0.04048785349535444222,Gymnocephalus_cernuus:0.04013179393609041939)100:0.13303408014007794824)69:0.05769006291048383445)47:0.05753414310379899310)84:0.09087134902678477288,(Peristedion_cataphractum:0.39498141826983063085,(Zoarces_viviparus:0.24515915970090554343,Cottus_gobio:0.38323838319141689812)55:0.10095556659846541270)7:0.02804320205362595755)7:0.03366342408954704768,(((Epinephelus_aeneus:0.36207917236591785626,Cephalopholis_taeniops:0.23056078970223736757)98:0.32526406321635531471,(Bembrops_greyi:0.12330776295467063552,Bembrops_heterurus:0.16129896614447031267)90:0.12025373698191710314)22:0.12431353826079598324,(Bovichtus_diacanthus:0.65014418033340326541,((Sebastes_marinus:0.11323292098456776145,Helicolenus_dactylopterus:0.05297949520538986629)100:0.45167174652961639136,(Pseudaphritis_urvillii:0.20132111485429826647,(Gobionotothen_gibberifrons:0.07563546471888323020,((Pogonophryne_scotti:0.03011274004087327247,(Harpagifer_kerguelensis:0.09671765986330538301,(Parachaenichthys_charcoti:0.12510955455277417148,(Champsocephalus_gunnari:0.04122309652040930666,(Chionodraco_hamatus:0.01285294511080087299,Chaenocephalus_aceratus:0.02207190316203247460)98:0.02937362334783327408)100:0.04579984560430881008)85:0.02148852655675373424)38:0.01064018456185053703)69:0.03778599505071765507,((Trematomus_newnesi:0.09203578700705683513,(Lepidonotothen_larseni:0.08666303084818828550,Lepidonotothen_squamifrons:0.04799498029437641172)79:0.04178435798837389775)100:0.14296644447153231128,Notothenia_coriiceps:0.11203375630427118981)47:0.01586370524429512804)72:0.04857002709968661486)96:0.38957880715096293578)59:0.06844241228840176883)25:0.04525099377406079787)24:0.14988524003302172716)2:0.00634983721050693100)7:0.08477108063721616737,(((Trachurus_picturatus:0.31613397277054022805,(Pleuronectes_platessa:0.50762738547675900769,(Istiophorus_platypterus:0.01951242124223955335,(Makaira_nigricans:0.02571877493105806031,(Makaira_indica:0.01805304167665399903,((Tetrapturus_georgii:0.01870866034274404691,(Tetrapturus_belone:0.00335026083629381575,Tetrapturus_pfluegeri:0.00190375784531435829)100:0.02483087779366752873)97:0.02360787547908365508,(Tetrapturus_audax:0.00262867354515545744,Tetrapturus_albidus:0.00090666077597796211)100:0.02202281481835079852)78:0.00449100352041833205)61:0.01314498007618231969)49:0.02324871274595990620)99:0.24309400561188548862)46:0.05353991648043422702)7:0.05022498182663019672,((((Lepidocybium_flavobrunneum:0.39634510504113640295,Scomberomorus_brasiliensis:0.08745775878678202497)24:0.06109986469821585692,(Auxis_rochei:0.08140662944162353754,Auxis_thazard:0.05307206824992290878)86:0.11850905552824934908)14:0.03606722753521359598,(Thunnus_obesus:0.01220077021327640142,Thunnus_tonggol:0.01373261965339350917)99:0.20275044119800761377)43:0.12186321031484216582,(((Sicydium_gymnogaster:0.14100397964119107397,Sicyopterus_japonicus:0.16731991751998800955)100:0.10540222444803906687,(((Stiphodon_pelewensis:0.01761351271277853542,Stiphodon_elegans:0.06501731201783558789)97:0.07720456324578241214,(Stiphodon_caeruleus:0.13063892480132119345,Stiphodon_percnopterygionus:0.08370656338267734586)44:0.03454884320553096994)28:0.01847018211034721566,Stiphodon_rutilaureus:0.13274388829402691825)41:0.03716590422418339368)100:0.67010677652987982178,(((((Cichla_intermedia:0.03395937068455953667,Cichla_orinocensis:0.01690013013518345750)96:0.04504153116147331659,(Cichla_monoculus:0.00000785557705005884,Cichla_temensis:0.00251814962774616962)99:0.05635148945183242331)97:0.12473801728978871350,((Acarichthys_heckelii:0.32423937202341884811,(((Taeniacara_candidi:0.92756268996595259146,((Apistogramma_hoignei:0.15202016785700039891,Apistogrammoides_pucallpaensis:0.14469959699024989574)83:0.06197532279489619683,Apistogramma_agassizii:0.35308197168778243658)84:0.06462146734211021704)55:0.07951859360312386882,(Satanoperca_daemon:0.17241598520100495651,(Satanoperca_leucosticta:0.05454212466099367385,(Satanoperca_jurupari:0.01756866000386868992,Satanoperca_mapiritensis:0.01333774480987441960)93:0.03024208317503766091)100:0.16360276437405979477)86:0.05663920755694262787)47:0.07904791583434518654,((Mazarunia_mazarunii:0.25444618587728679016,(((Crenicichla_reticulata:0.12652467278650755822,Crenicichla_geayi:0.09162675882875194466)100:0.27630269730789575044,(Crenicichla_sveni:0.28162940340002884376,Crenicichla_lenticulata:0.32761103315888118193)94:0.11351373056094793412)100:0.99949958121301152314,(Biotodoma_wavrini:0.06097761765795763761,Biotodoma_cupido:0.62504855504681300360)90:0.10230241200310126459)12:0.11230576306692684196)15:0.08042393598456243353,(Biotoecus_dicentrarchus:0.37489310187256424944,((((Geophagus_dicrozoster:0.06056636568306619783,(Geophagus_abalios:0.04948102331252255542,(Geophagus_surinamensis:0.02660154133812746638,Geophagus_brachybranchus:0.03018421136236601118)75:0.01398786967588007586)71:0.01816886679238939942)82:0.03102747992644472319,(Geophagus_harreri:0.10909381891117959917,(Geophagus_taeniopareius:0.10964830466750638549,Geophagus_grammepareius:0.06634946721367573907)65:0.05590415881324095615)64:0.02612042331431000536)57:0.02757505135243223937,Geophagus_steindachneri:0.25775078980560173214)62:0.06124362230970822885,(((Mikrogeophagus_ramirezi:0.19888405605011624799,Mikrogeophagus_altispinosus:0.23996251619624148210)64:0.05155202727110519412,(Gymnogeophagus_rhabdotus:0.05230108350565083886,Gymnogeophagus_setequedas:0.05307078417601305415)97:0.18773444538941541637)19:0.02310152006314280734,Geophagus_brasiliensis:0.12403332384725973547)11:0.01177051383723980160)7:0.01776489264975480639)6:0.03021280133232885981)0:0.00448248602524799981)3:0.01871633547924102595)12:0.02643159549073944084,((Chaetobranchus_flavescens:0.09777426578468302598,Chaetobranchopsis_orbicularis:0.28676656203844369530)96:0.11088467803475732243,((Cleithracara_maronii:0.25617192066842492393,(((Acaronia_vultuosa:0.10842917943056212815,Acaronia_nassa:0.09657998581623551682)99:0.09262301592017600782,((Laetacara_thayeri:0.19130634078644945251,Laetacara_dorsigera:0.13833358873590534399)78:0.07541958190337438861,(Tahuantinsuyoa_macantzatza:0.20310792213140063667,((Andinoacara_latifrons:0.00972842741839560592,(Andinoacara_pulcher:0.03786701789664996542,Andinoacara_coeruleopunctatus:0.05444251253243745370)45:0.03314148115029871833)100:0.13381143407046691696,(Andinoacara_biseriatus:0.08335715594207832035,(Andinoacara_rivulatus:0.02717449910518978870,Andinoacara_stalsbergi:0.03288611332719069968)100:0.09836661747522623678)63:0.02099023059009415618)98:0.05342130864313530958)45:0.02666456653272253688)55:0.02746052841075562537)66:0.05795937813827791579,(Aequidens_potaroensis:0.18890993289088489626,((Aequidens_diadema:0.04836197843957583398,Aequidens_tetramerus:0.03540007569312703106)98:0.04683047274263497928,(Cichlasoma_dimerus:0.09717828853802991385,(Cichlasoma_orinocense:0.00803289123425326816,Cichlasoma_amazonarum:0.00744614393331233075)99:0.04872061074300444716)97:0.06481004607829324304)90:0.06865880588501135917)46:0.02364574703218918356)32:0.01264994877432752164)35:0.02199004008599542495,(((((Cryptoheros_nanoluteus:0.15317377301264303857,(Nandopsis_tetracanthus:0.09835273782240343265,Nandopsis_haitiensis:0.17850404951493814565)30:0.01581835402513243408)18:0.00853192977294777929,((((Cryptoheros_panamensis:0.05849946624471014794,(Hypsophrys_nematopus:0.05099085981802907452,Hypsophrys_unimaculatus:0.03695846027502058817)100:0.03661599035666576046)34:0.00723942021215408238,(((Cryptoheros_sajica:0.05943467813647963782,(Parachromis_loisellei:0.05977380818611301161,(Parachromis_dovii:0.02904827283709019575,Parachromis_managuensis:0.02820357838956231988)94:0.02093137065000876332)27:0.00456755352397162587)29:0.01041950213751096678,(Theraps_wesseli:0.07038036392221341808,((Cryptoheros_chetumalensis:0.01508489062939097694,Cryptoheros_cutteri:0.04040887338684324981)100:0.07849121162332589607,((Cichlasoma_trimaculatum:0.04113633605205343352,Amphilophus_lyonsi:0.02643065894676446120)94:0.02395105496570704498,(((Amphilophus_hogaboomorum:0.00000785557705005884,Amphilophus_citrinellus:0.00000785557705005884)57:0.00182882574615550312,Amphilophus_labiatus:0.00000785557705005884)100:0.03367379005463945474,Archocentrus_centrarchus:0.09941803063781860794)42:0.01359564532241806963)22:0.00834638995752400199)3:0.00627248843663747498)0:0.00000785557705005884)7:0.00612602507800532204,(Australoheros_facetus:0.07577005009123406742,(Cryptoheros_myrnae:0.00666466995845086535,Amatitlania_nigrofasciata:0.00335118401401315403)100:0.07252223402680589570)22:0.01293639359729778504)16:0.00566185018442181935)11:0.00000785557705005884,Petenia_splendida:0.09969843853142267009)41:0.01766349769146085408,((Caquetaia_kraussii:0.16410626130631414399,Caquetaia_umbrifera:0.10727304032539218548)17:0.00089484223627885778,Heroina_isonycterina:0.12049165835693251281)25:0.00927007769457246882)52:0.02548240961296338591)8:0.00598097850363657740,((((Amphilophus_robertsoni:0.05846396017249875371,(Amphilophus_diquis:0.03789770542439396817,(Amphilophus_rostratus:0.01220373795927881012,Amphilophus_longimanus:0.01609073660826161253)99:0.01388958298519987235)97:0.03662141599831544164)97:0.05413885523863191940,Rocio_octofasciata:0.13117795851084995684)16:0.01757679903114399422,((((Thorichthys_affinis:0.03725507550580085120,Thorichthys_helleri:0.03184098186034787364)73:0.00918781385018894829,Thorichthys_pasionis:0.03800492755977018244)97:0.08297251588472907835,(Tomocichla_tuba:0.03346080419450067622,Tomocichla_asfraci:0.04266400382046898337)85:0.02880063053500920775)26:0.01437146392885602086,(Cichlasoma_festae:0.10614626009325729694,(Cichlasoma_grammodes:0.13348297865408789709,((Cichlasoma_ufermanni:0.04457324396978942782,(Theraps_irregularis:0.01914013252600227019,Amphilophus_nourissati:0.03864547074068317573)95:0.01822667797931851186)39:0.00579638815303822920,((Vieja_guttulata:0.00841453047788389558,Vieja_bifasciata:0.01450411806189387184)100:0.04892376412515388184,(Cichlasoma_bocourti:0.03096290553778016652,(Theraps_lentiginosus:0.09421317259721095905,((Herichthys_bartoni:0.02875789882344270340,(Herichthys_pantostictus:0.01479018311453202637,Herichthys_steindachneri:0.01924900853883943982)83:0.00546837137268900604)100:0.03499179812588650018,(Amphilophus_calobrensis:0.05028243960873833573,((Herichthys_cyanoguttatus:0.00329343200387415469,Herichthys_labridens:0.01678314547436406123)45:0.00507518909537247719,(Herichthys_tamasopoensis:0.00455881937649190404,Herichthys_carpintis:0.00173450318646706475)62:0.00000785557705005884)63:0.00809380226849251184)97:0.04047477310852624216)73:0.01989269139197961250)9:0.00111997717202016686)10:0.00511918429119757878)14:0.00178989337204427568)68:0.02267891052760653836)43:0.01889029373047345500)9:0.00263762223447371351)14:0.01579133364063670802)6:0.00559664877615514457,(Archocentrus_multispinosus:0.15289947122755215281,Amphilophus_macracanthus:0.12326610781962689389)8:0.02399371802530607042)5:0.00808286036543654114)17:0.01556653606313370027,(Heros_efasciatus:0.07644138036101293110,((Mesonauta_egregius:0.02108143079261917338,Mesonauta_insignis:0.01516208391707033268)100:0.15035484240907623366,((Uaru_amphiacanthoides:0.06222107179844454938,Uaru_fernandezyepezi:0.03945407455295332072)100:0.06855632893970298181,(Symphysodon_aequifasciata:0.00984923890231968573,Symphysodon_discus:0.00694431874544474120)100:0.11141428140510049871)50:0.00922956927370459541)43:0.01202927098362310473)68:0.02268974777571161616)48:0.03297437043835961534,((Pterophyllum_scalare:0.28174861416974900097,Hoplarchus_psittacus:0.10646768200240790125)38:0.03859072940384559308,Hypselecara_coryphaenoides:0.12024891985463342925)22:0.03079837009277514373)45:0.04690311627328399430)12:0.01199827207256699141)27:0.02998975073942135999)16:0.01563602211696901029)33:0.10028431162888278017,(Chromidotilapia_guntheri:0.38309883772974578919,(((Hemichromis_fasciatus:0.00000785557705005884,Paratilapia_polleni:0.00000785557705005884)100:0.23243269666902177661,(Tropheus_moorii:0.07317588484373002689,Neolamprologus_modestus:0.11435348611527589024)40:0.03413561678810636546)27:0.02311466135889990448,Oreochromis_tanganicae:0.08687002141726139770)35:0.01124110480711938351)79:0.10143432824705361783)29:0.07384354841781889944,Etroplus_suratensis:0.33958752236407069303)16:0.05720788055838244013)9:0.08759766003470312312)1:0.05797260436265805339)0:0.00941498133005342491,(((((Labrus_merula:0.29302041863562494628,(Ctenolabrus_rupestris:0.08569493310154950583,Tautogolabrus_adspersus:0.07563432182238929991)85:0.08130502868308141229)45:0.08642880202716807236,(Sparisoma_cretense:0.82540542183336318427,(Thalassoma_pavo:0.77597500966381649512,Coris_julis:0.48614697313286558966)81:0.24190400230328756859)8:0.02556684299842509542)51:0.22431239116686554902,((Lagocephalus_lunaris:0.33381831235668113500,Takifugu_fasciatus:0.47744960927461699285)92:0.18916013542030538086,(Stephanolepis_hispidus:0.85302904255874945072,Balistes_capriscus:0.58308445929605934310)4:0.00000785557705005884)8:0.06833894927145794940)3:0.05677011061459385360,(Lutjanus_griseus:0.04559948426128022925,(Lutjanus_synagris:0.08037452212154315434,(Ocyurus_chrysurus:0.10606269685966102090,(Lutjanus_peru:0.00757098259878120124,Lutjanus_campechanus:0.01670018506047778184)86:0.03009816592636484905)32:0.03029718271105938387)92:0.10913839393924609777)71:0.15017973464781747550)0:0.06273290393302193013,((Erythrocles_monodi:0.26429987498860896267,Plectorhinchus_mediterraneus:0.32326182844418149820)30:0.06414857751480147752,((Menticirrhus_littoralis:0.10623847678594415533,Menticirrhus_americanus:0.07510630814002318412)100:0.11668666519901982337,((Stellifer_lanceolatus:0.31582596558484177240,(Satanoperca_pappaterra:0.17032498018780278803,Larimus_fasciatus:0.14624514725085521749)38:0.02020098754346991154)29:0.01901458391791880156,(Micropogonias_undulatus:0.16410098390159946757,((Sciaenops_ocellatus:0.09941421564866671778,((Cynoscion_nebulosus:0.11612676925504640535,Cynoscion_arenarius:0.09000700097281506828)97:0.07255511497281036393,Leiostomus_xanthurus:0.15150093781310103314)27:0.02191553741000062949)20:0.01598727897782607418,Pogonias_cromis:0.15579374849253543878)6:0.00092599748381059393)19:0.01965216556187482019)27:0.03308045912209033740)78:0.12182365410944934192)11:0.01412880529306595294)4:0.05824443267207513042)0:0.05640646584820181025)0:0.04556300526289422392)35:0.17589333126292572285):0.45000000000000001110,Chlorophthalmus_agassizi:0.45000000000000001110);

Figure 2: COI tree

((((Scorpaenopsis_diabolus:0.20765082429375425477,Sargocentron_rubrum:0.28008036620259046945)32:0.08675486455715246403,(((((Monocirrhus_polyacanthus:0.43533470091940268398,(((Decodon_melasma:0.41232790180516903922,((Apogon_lineatus:0.34490878122120582905,((((Sphaeramia_orbicularis:0.15687446629489881778,(Cercamia_eremia:0.04060758259537110976,Gymnapogon_urospilotus:0.09012889098949686262)98:0.15791441878755729800)56:0.10170940370880247883,(Apogonichthyoides_taeniatus:0.32720284600919269380,(Fowleria_marmorata:0.33926410763825448624,(Fowleria_variegata:0.13266696153508852896,(Fowleria_aurita:0.08096703834294889657,Fowleria_isostigma:0.13151170017145669133)96:0.05689247323518594607)93:0.07760864265005924800)87:0.08716195858318542822)32:0.06102608811880117640)4:0.02664278361178361310,(((Glossamia_aprion:0.46980391955577677221,Apogon_trimaculatus:0.17643812302327438468)10:0.06501825157699109292,((((Nectamia_fusca:0.13527658073592918608,Apogon_quadrifasciatus:0.08049048962032789856)98:0.13374296869027652024,((Apogon_sealei:0.13442688018196025102,((Apogon_doederleini:0.19572840567232885345,Apogon_angustatus:0.11643593668556125043)61:0.03851010036429994288,Apogon_nigrofasciatus:0.13862654082876704753)42:0.01416427657860096570)38:0.03478976166477269066,(((Apogon_semilineatus:0.11971051908365644423,Apogon_erythrinus:0.09461588884443224157)44:0.02282247995224755133,Apogon_apogonoides:0.12505636595659108679)46:0.03979313172595004555,(Apogon_cookii:0.24858874714432568909,Apogon_endekataenia:0.14624555700005767434)61:0.10023037602798511025)47:0.05494891844421295746)20:0.00401755264050515995)24:0.02554040845698320530,(((Archamia_fucata:0.03428410179811455794,Archamia_biguttata:0.05847952361742995986)100:0.16474715018713526060,(Apogon_natalensis:0.22420155312256381808,Apogon_hyalosoma:0.14553145611154913452)27:0.07299204729385921908)1:0.01354768138346214637,(Cheilodipterus_macrodon:0.11322471824845312116,Cheilodipterus_isostigmus:0.13108533858384366355)100:0.13959136806963792776)3:0.01581489476490529136)6:0.01411515164577116510,(Apogon_exostigma:0.18659589433586198925,Apogon_kallopterus:0.24650447985360091074)66:0.08860227023469212371)4:0.00897889562917522817)1:0.01599159207057426063,Sphaeramia_nematoptera:0.20397515150778930404)0:0.00067508755483302305)1:0.04030606466876617694,(((Astrapogon_alutus:0.19116633983839992350,(Astrapogon_puncticulatus:0.35367835720654816622,Astrapogon_stellatus:0.14648337681726744663)60:0.03390695726734017923)75:0.06336458500342977140,(Apogon_phenax:0.09502233432732554164,((Apogon_maculatus:0.08366743598143086547,(Apogon_pacificus:0.09460484564747029257,Apogon_dovii:0.07643176773774489063)92:0.05391541551675922694)87:0.06356036380888251125,(Rhabdamia_gracilis:0.20021413325616549406,Apogon_townsendi:0.16768545409172211369)41:0.02506325021090494848)37:0.02187326751813468279)87:0.12378257255165096840)68:0.05073343988632874474,((Phaeoptyx_xenus:0.17175542245469707647,(Phaeoptyx_pigmentaria:0.15228465974653981885,(Phaeoptyx_conklini:0.00591951957212114282,Apogon_quadrisquamatus:0.00409445087071008944)100:0.15009055747781593904)75:0.02169611512888217422)75:0.05709882811142304865,Apogon_aurolineatus:0.41948868877056233462)92:0.08768834259480015603)90:0.09448838952708542294)0:0.00543407903498959507)3:0.02340273726971126012,(Siphamia_versicolor:0.64441831583619968438,Rhabdamia_cypselura:0.31255842430746949567)4:0.09485112594549074960)1:0.01930247218319902300)0:0.01802682136737347035,(Apogon_norfolcensis:0.28907507190545495002,((Pempheris_schwenkii:0.24874073386153272569,(Pempheris_adusta:0.16320060867462513055,Pempheris_vanicolensis:0.23313269405909636811)83:0.06243078060798194079)100:0.31026539009723769214,Parapriacanthus_ransonneti:0.45583187667111102481)28:0.11653316766626108103)7:0.06410326873614678733)0:0.01925668242716029391,((Glaucosoma_magnificum:0.25712870933059950307,(Glaucosoma_hebraicum:0.15871175678692556188,(Glaucosoma_buergeri:0.04931419767891649653,Glaucosoma_scapulare:0.01900706317484930630)99:0.10525012054748639212)74:0.05083926862364233912)93:0.15629633837860998358,(((Chilomycterus_reticulatus:0.09249303184631393893,((Diodon_hystrix:0.14109523256769274102,Diodon_holocanthus:0.04350746404233681724)88:0.06874261203505092899,Chilomycterus_antillarum:0.25818984908859421434)77:0.06769057217039259333)91:0.29450492945691358893,(Capros_aper:0.42343653701363792585,(Pentaprion_longimanus:0.47782132221776701941,(Haletta_semifasciata:0.39797825837788414205,Kurtus_gulliveri:0.42262974398155250810)9:0.07343823239652125245)0:0.02637853150671051403)0:0.04528207363271262442)0:0.00952307077135093084,(Mola_mola:0.20457369126406244053,Ranzania_laevis:0.13308572688811881601)97:0.22060904598053809877)0:0.03860853030366580979)0:0.04444957042221679699)0:0.00843093789110202496)0:0.02271286743822247337,(((Hoplichthys_acanthopleurus:0.06076531331463468077,Hoplichthys_citrinus:0.03185044052031250211)100:0.31542579261255365708,(Ophioblennius_atlanticus:0.36692630873668691249,(Eugerres_plumieri:0.19815144052612479464,Diapterus_auratus:0.23525355703060202295)96:0.14719188155509463356)3:0.08334030043468108884)1:0.04354677972473623704,(((Scorpaenopsis_cirrosa:0.00000176456401954691,Scorpaenopsis_vittapinna:0.00695382043501013240)98:0.34358127880957067823,((Pseudamia_gelatinosa:0.38872836611846983690,(Notesthes_robusta:0.24079204582508015320,(Apistus_carinatus:0.38663980245061674301,((Elassoma_evergladei:0.17010897303297461969,(Elassoma_zonatum:0.25479993513262355620,Elassoma_alabamae:0.11698787460137659300)31:0.12642010976244183462)99:0.35311238952581958106,(Minous_monodactylus:0.39737132507007127957,Hypodytes_indicus:0.18555410681137954332)47:0.07596435526554776163)2:0.06459968057588395818)0:0.00000176456401954691)0:0.09460256899972656341)0:0.04415501690737502066,((Scorpaena_inermis:0.13252824370252913599,(Scorpaena_brasiliensis:0.20388795919759192254,Scorpaena_grandicornis:0.31521780838219315424)83:0.14249452375158702666)75:0.15507476736751266144,Synanceia_verrucosa:0.39670601604723126909)12:0.07108739133937908017)0:0.00000176456401954691)0:0.05790957345817209639,((((Marosatherina_ladigesi:0.39199124258153339451,(Atherion_elymus:0.44038767451147031728,Rhadinocentrus_ornatus:0.33979830988518594515)16:0.07583126958587121935)1:0.08066501246213100829,((Aphyosemion_pascheni:0.00000176456401954691,(Aphyosemion_festivum:0.02230787684131491089,(((Aphyosemion_heinemanni:0.06435553533270232940,(Aphyosemion_ahli:0.02067631639129689780,Aphyosemion_edeanum:0.05672452787578628752)74:0.02738577018064568616)72:0.04742024978066647356,(Aphyosemion_australe:0.11491826302807223170,(Aphyosemion_calliurum:0.05143482348485114292,Aphyosemion_celiae:0.06283334871193119175)99:0.06323964380121983864)44:0.01384382564927354523)34:0.01846119294445921696,Aphyosemion_franzwerneri:0.12458857639162276842)47:0.07020272200003120633)38:0.03122977860762845073)92:0.43648154874780747958,((Perccottus_glenii:0.38558265050655504691,(Odontobutis_interrupta:0.08479049793619930497,Odontobutis_potamophila:0.27879145748712091946)95:0.16130147950373951993)21:0.06220613846630421856,(Cubanichthys_cubensis:0.24036803193182243454,Cubanichthys_pengelleyi:0.26720895459811461192)51:0.09735221855331976926)2:0.03026321386545345257)0:0.07244347984921484240)0:0.00000176456401954691,((Ophisternon_aenigmaticum:0.52662268642130805052,((Gramma_melacara:0.34840193353045462166,Gramma_loreto:0.04615286242402934919)98:0.24498313291502515909,Ecsenius_bicolor:0.36884407444806105447)42:0.12849738843221014672)0:0.09056230154222523476,((Ambassis_miops:0.14271270662451848721,Ambassis_marianus:0.10283904662580502232)97:0.27344703740521447699,(Chanda_nama:0.27486839054197187426,Ambassis_agassizii:0.50268359427621611690)16:0.09805945194689240363)15:0.06022061837924954503)0:0.02558521532338341870)0:0.06744303191873494974,((((((Amblychaeturichthys_hexanema:0.36680479027948076354,Fusigobius_neophytus:0.31255496090958401201)16:0.15380709619505630870,((Bathygobius_laddi:0.28158302996136297391,(((((Bathygobius_ramosus:0.05966617960849320695,Bathygobius_soporator:0.10927407348981610369)100:0.11126663744476214535,Bathygobius_curacao:0.14090330272659870592)13:0.01216834419828830699,(Bathygobius_andrei:0.06956868766713945884,Bathygobius_lacertus:0.06849044628748461594)92:0.07948279116604090033)39:0.02335695836899350994,Bathygobius_mystacium:0.20366491513735232588)62:0.04900069490913923353,Bathygobius_cocosensis:0.22782715235609374282)62:0.07156808708266310104)39:0.06463317050378153406,(((Fusigobius_inframaculatus:0.27795288685948998930,(Fusigobius_signipinnis:0.27425933082504372340,Fusigobius_duospilus:0.42908937188476176106)50:0.10756223104522456835)8:0.09607602658366269643,((((Coryphopterus_lipernes:0.21571888038240394359,(Coryphopterus_hyalinus:0.06839781807293453253,Coryphopterus_personatus:0.06067235410603437351)91:0.09238286248575852677)49:0.10069090941900260550,Lophogobius_cyprinoides:0.30217584072570563558)11:0.06288512593864066524,((Coryphopterus_dicrus:0.27198558434447095422,(Coryphopterus_tortugae:0.07838205637056699926,(Coryphopterus_glaucofraenum:0.11221907955686113989,(Coryphopterus_bol:0.00000176456401954691,Coryphopterus_venezuelae:0.00878209528406395136)98:0.03651230835286892185)86:0.06702576315313732436)96:0.14586170410681753573)80:0.11781641069445791814,(Coryphopterus_alloides:0.22880508025118745374,(Coryphopterus_eidolon:0.20653227842068933029,Coryphopterus_thrix:0.31838864316751830374)73:0.05400600397862657248)12:0.02195561756022055763)36:0.02744537384814794631)45:0.08521741571781091262,Rhinogobiops_nicholsii:0.51100006089682792343)52:0.05194815542477244225)11:0.06225945087859734162,Coryphopterus_kuna:0.48651513162341014640)1:0.03242452579248132627)0:0.02137116932360529423)0:0.00319152346872099077,(Salaria_fluviatilis:0.27667770348700942984,((Parablennius_rouxi:0.13682035166058192921,Parablennius_pilicornis:0.18367938075711456869)56:0.00857273662234868047,Parablennius_cornutus:0.27511336743520903303)91:0.16074583678014076060)33:0.14531740363706918684)0:0.03877700970702343403,(((Priolepis_cincta:0.32671708460712661770,(Lythrypnus_elasson:0.00253115999722244162,Priolepis_hipoliti:0.00476571870290945325)99:0.38801022811313296002)33:0.25276630119179782641,(Trimma_flammeum:0.51570467444436796089,(((Lythrypnus_dalli:0.16764965714849738854,Lythrypnus_zebra:0.62501317192327487948)99:0.58480123870943567788,Salarias_fasciatus:0.27304966686966231082)0:0.09672928562352745008,((Heterostichus_rostratus:0.33116119321179138257,(Gibbonsia_montereyensis:0.06061900199212731655,Gibbonsia_elegans:0.07464481780205652062)94:0.02656352565823819761)92:0.31166558091122198437,(((Alloclinus_holderi:0.42058276421093820918,((Cocotropus_monacanthus:0.60000171412100422597,Parapercis_maritzi:0.77911516332447372513)5:0.24381480054498219645,(((Mullus_barbatus:0.12165372617716867232,Mullus_surmuletus:0.06549497596812001166)89:0.07942673007868178425,((Mulloidichthys_martinicus:0.16006341403834437043,(((Upeneus_moluccensis:0.10975245366503633138,(Upeneus_vittatus:0.09239713963674826835,Upeneus_sulphureus:0.06256441903567536156)58:0.00765610604136095999)88:0.08027862826369024096,((Upeneus_tragula:0.05117057726911850013,Upeneus_japonicus:0.14746200230090161787)50:0.02338936350634499353,Mulloidichthys_vanicolensis:0.09638464232199740744)98:0.13876422603484331320)84:0.08399579871209890414,((((Parupeneus_pleurostigma:0.22253713970683644541,Parupeneus_heptacanthus:0.09381385689810432804)59:0.04127750443428614135,(Mulloidichthys_auriflamma:0.00000176456401954691,Parupeneus_forsskali:0.00439605635108861279)100:0.15591633189905526602)45:0.03337009786416960527,((((Parupeneus_cyclostomus:0.19751703819862623246,(Parupeneus_indicus:0.11641693149556602005,((Parupeneus_spilurus:0.04103308659662226188,Parupeneus_ciliatus:0.06400515332981285277)90:0.05195588823476841173,Parupeneus_bifasciatus:0.07372141658901232297)26:0.01195202187656937802)16:0.00900237818892468851)3:0.00083033039920588439,(Parupeneus_barberinus:0.01623151928176813294,Parupeneus_multifasciatus:0.00807368783005230593)95:0.07309412081195430788)34:0.03057195893290768837,Parupeneus_chrysonemus:0.16868062964651639457)38:0.02294880012899351909,Parupeneus_fraserorum:0.20829970883009185645)7:0.01218730526466588887)19:0.01875574739033725180,Pseudupeneus_grandisquamis:0.37394409162396208268)10:0.01786382126571190929)12:0.02527790702138607223)9:0.03399444982288413186,Parupeneus_barberinoides:0.25796358359343118938)14:0.03155570493584813202)23:0.05847987438956848277,Upeneichthys_vlamingii:0.33487998485724057574)35:0.04365434481284522633)0:0.01404634753794195903)0:0.04385083420208852473,((Paraclinus_marmoratus:0.21087448953677653818,Paraclinus_integripinnis:0.27294368269805496796)28:0.07435584541319338603,((Chorisochismus_dentex:0.50353713609272610086,(Labrisomus_bucciferus:0.23784677433643736100,(Gillellus_jacksoni:0.38783953769871282180,Gillellus_uranidea:0.29355246409591001511)30:0.15247011342442440274)14:0.10477414518544633792)0:0.08249947242628662136,(Paraclinus_fasciatus:0.20083389013782163013,((Mccoskerichthys_sandae:0.40971927879773717418,Neoclinus_blanchardi:0.26234626051173665084)15:0.04950365421365218038,(Labrisomus_nuchipinnis:0.48045790371721663892,(Malacoctenus_erdmani:0.44889163944381865745,Malacoctenus_macropus:0.13249492457262984635)56:0.08400023355971070194)16:0.08124027888972606970)2:0.08523880626493479662)0:0.01407326009856458247)0:0.03155627679625055970)0:0.05447280748156881763)0:0.03862588185746159558,((((Hemiemblemaria_simulus:0.50804511091482251750,((Emblemaria_nivipes:0.32842172595547042269,(Emblemaria_diphyodontis:0.24598769399576420058,Emblemaria_pandionis:0.18382181399829514157)94:0.15198835451193409840)33:0.06800476406337559176,(Emblemaria_hyltoni:0.50939146474463403713,Lucayablennius_zingaro:0.34448142087080874107)16:0.03826976446721557651)8:0.02631591000303686034)4:0.03926569610154747453,((Ekemblemaria_myersi:0.33290155622955391879,Ekemblemaria_nigra:0.26714259375207294323)45:0.18259799522636979696,(Chaenopsis_limbaughi:0.39110438639761246904,(Emblemaria_vitta:0.29572848497322606898,(Chaenopsis_alepidota:0.21418423238897274508,Chaenopsis_schmitti:0.42266706533605197249)48:0.11935760000088942168)19:0.04946389639605960897)1:0.02925547617677101697)0:0.06111775137467385238)1:0.04694873117635870591,(((Emblemariopsis_arawak:0.09275060894106604281,(((Emblemariopsis_pricei:0.00000176456401954691,Emblemariopsis_bahamensis:0.01092307315756093321)98:0.11946505869555258672,Emblemariopsis_randalli:0.15532246605925648097)42:0.04357921632723134320,(Emblemariopsis_carib:0.12864924330212365589,Emblemariopsis_signifera:0.14618625015211403340)23:0.03375826241278704481)34:0.04676768455396600882)44:0.04910035445467315907,Emblemariopsis_ruetzleri:0.38809248935156986926)75:0.22899586811550143306,(((((Acanthemblemaria_aspera:0.14695666199899640181,Acanthemblemaria_paula:0.14590218115694406076)95:0.37660274579415342622,((Acanthemblemaria_betinensis:0.23470985408376085490,Acanthemblemaria_greenfieldi:0.47954998010442106970)22:0.08182063942446483884,Acanthemblemaria_exilispinus:0.21295047904030042774)6:0.03933119558530163984)1:0.05205876524643457404,(((Coralliozetus_cardonae:0.29393858657782007127,Coralliozetus_springeri:0.29719806478934490679)94:0.34873819043472087387,((Coralliozetus_boehlkei:0.37433882920671435190,Coralliozetus_rosenblatti:0.41509943179920794076)50:0.07921097367668145095,Coralliozetus_angelicus:0.39014757226919422051)20:0.09638871605057480751)34:0.31891583285577584927,(Acanthemblemaria_spinosa:0.39834313477425153938,Acanthemblemaria_chaplini:0.28667622470326564743)27:0.12927344597624859657)0:0.05937909943292224435)0:0.02906837737216461798,(Acanthemblemaria_castroi:0.24887758262884404625,Acanthemblemaria_rivasi:0.27367176356618316824)63:0.14412559076794803636)0:0.01768588515753354018,(Acanthemblemaria_crockeri:0.24382746478412087310,(Acanthemblemaria_macrospilus:0.23458971837919431747,Acanthemblemaria_hancocki:0.14940253946039364075)90:0.09098780216563141787)58:0.18386421431438987328)0:0.04379421262530697190)0:0.04659960921084522339)0:0.02120220369623604384,((Cirriemblemaria_lucasana:0.54252498374834778883,(Emblemaria_hypacanthus:0.16509689250509065883,Emblemaria_piratica:0.21368283425848469403)99:0.21220792917796010846)3:0.07484951900092598476,((Starksia_elongata:0.21052051088699402781,(Starksia_guttata:0.01328709724172322942,((Starksia_ocellata:0.03010305472590535728,Starksia_occidentalis:0.01737050216359568161)48:0.01265764039107136404,Starksia_culebrae:0.05890754934104594498)45:0.02103843321291655119)95:0.14668082254898781591)86:0.11790305044827535663,((Starksia_nanodes:0.33893094655113742419,((Starksia_williamsi:0.05713068745621280309,(Starksia_robertsoni:0.00634673780772483653,(Starksia_lepicoelia:0.00453265049020342391,Starksia_weigti:0.00435560037768215925)91:0.01206854729657283812)93:0.04770511356209761883)90:0.25657040907824790743,(Labrisomus_nigricinctus:0.00537362327948069738,(Starksia_sangreyae:0.00192693352217527506,Starksia_atlantica:0.00481516311877248828)78:0.02285000115059528450)90:0.18416153945909918832)37:0.05782974741520011119)29:0.10173048241254037394,(Starksia_hassi:0.39098974789177132205,(Starksia_multilepis:0.22488343238291916282,((Starksia_fasciata:0.03704602064616836776,Starksia_greenfieldi:0.10243784499417422917)98:0.17597275359017999774,(Starksia_langi:0.00000176456401954691,Starksia_sluiteri:0.00661471732830989315)93:0.10500547446925356798)50:0.09075015398182235959)63:0.08875574334678498767)11:0.03715122252092455807)21:0.06768670901900830650)26:0.11107720849892784021)0:0.03076026238256376888)0:0.01408555236365616019)0:0.00496571635538826418)0:0.03188677169929720473)0:0.01952375830378484320)0:0.02057018402740234606)0:0.02405584963629567655,((((Bryaninops_yongei:0.50534004465141457718,((Gunnellichthys_monostigma:0.42657128715221737103,((Microdesmus_bahianus:0.07117340734194624319,Priolepis_eugenius:0.30330393335995159410)64:0.14965755180156192772,(Microdesmus_longipinnis:0.21022295795665263407,Cerdale_floridana:0.25853594782919625539)96:0.10472960635464009793)61:0.14736751798353656118)25:0.06069581153117877687,((((Elacatinus_dilepis:0.00441396203322575939,Elacatinus_pallens:0.00000176456401954691)100:0.45656673928762486758,Risor_ruber:0.30041824082261819884)39:0.03949976916208799288,(Ophiogobius_jenynsi:0.33743626089753597785,((Elacatinus_horsti:0.00354071748070828793,Elacatinus_evelynae:0.02402210835247636789)100:0.15171543933450984398,Elacatinus_oceanops:0.19424231466802280299)98:0.17632990309402271634)74:0.08525526416332546153)55:0.13505448881493306579,Gobiosoma_chiquita:0.39623007114267072559)1:0.06370815727392213634)0:0.04548189223383805602)0:0.03350271754884839859,((Amblygobius_nocturnus:0.25397234808812818585,(Amblygobius_decussatus:0.19181285101490394562,(Amblygobius_albimaculatus:0.12000245418769760197,Amblygobius_phalaena:0.07482339421630659904)98:0.15337922087097966006)76:0.05000899485779062431)71:0.12189428737475954678,(Signigobius_biocellatus:0.30321607198910766057,(((((Favonigobius_exquisitus:0.00260889108804609115,Valenciennea_longipinnis:0.00346224153393487159)100:0.14901195332981367891,Valenciennea_muralis:0.11767744951307150536)79:0.09789159638860776835,(Valenciennea_sexguttata:0.18052676415757584749,(Valenciennea_strigata:0.21124916001414648092,Valenciennea_wardii:0.20498902376845726248)41:0.05210773618004096303)39:0.02163496756746074307)14:0.04602464810144740737,Valenciennea_puellaris:0.28176256743849209530)39:0.04442741213830859320,Valenciennea_helsdingenii:0.23284158951637207169)38:0.05400377240791311151)20:0.03632589750658350020)5:0.03077438353036286364)0:0.01700607746741494147,(((Ptereleotris_microlepis:0.00751379700263323826,Ptereleotris_monoptera:0.00000176456401954691)99:0.11805187838837929992,((Ptereleotris_heteroptera:0.21904212302183714844,Ptereleotris_evides:0.17525171337104161751)17:0.00000176456401954691,Ptereleotris_zebra:0.08843180199410798081)84:0.08068529684329815710)87:0.14697953420577791750,(((Favonigobius_melanobranchus:0.26823008581205870504,Afurcagobius_tamarensis:0.33137005657466273023)23:0.11936193270040908188,(Microgobius_microlepis:0.34012374639957626554,(Schindleria_praematura:0.00726778530997986092,Schindleria_pietschmanni:0.00000176456401954691)100:0.49974811205008801140)7:0.03502230410393912530)2:0.09362836506775427947,(Nemateleotris_decora:0.11669797251701409524,Nemateleotris_magnifica:0.15751655660136223980)98:0.18970739155878940663)0:0.02984078211941592826)0:0.07797314874228451764)0:0.02803039226601429512,((((Amblyeleotris_gymnocephala:0.13672069416892126958,((Amblyeleotris_periophthalma:0.07255999866110868224,Amblyeleotris_randalli:0.04700643727065508676)99:0.15514509430040959725,((Amblyeleotris_wheeleri:0.00244839197058837731,Amblyeleotris_fasciata:0.00000176456401954691)100:0.11689507349846248174,(((Amblyeleotris_steinitzi:0.00123184713017566809,Cryptocentrus_strigilliceps:0.00000176456401954691)100:0.04419968632772194872,Amblyeleotris_guttata:0.05108430890878622177)99:0.07836071571920061318,(Amblyeleotris_aurora:0.02429767231108946948,(Amblyeleotris_sungami:0.00346074263476778407,Amblyeleotris_yanoi:0.00568261812215052359)93:0.01849570019021062883)98:0.06203192339842368563)97:0.02763776062749687124)95:0.06392303907450208600)34:0.00501957795164310536)96:0.09073897520610413325,(((Ctenogobiops_aurocingulus:0.09766370325777989914,Ctenogobiops_feroculus:0.12484776399234671918)81:0.03411247472136870462,(Ctenogobiops_tongaensis:0.12905308404207033179,(Ctenogobiops_mitodes:0.14893377687062972492,(Ctenogobiops_formosa:0.12931198135167229490,(Ctenogobiops_crocineus:0.04766310462664865438,Ctenogobiops_tangaroai:0.03434613745970530735)97:0.03792090335362516273)90:0.03716894080920776083)53:0.03211582441746006766)48:0.03023279442557728902)81:0.09618728361982581410,((Croilia_mossambica:0.07761252666571499814,Vanderhorstia_ornatissima:0.09534250800504499856)76:0.03213371869471580433,Tomiyamichthys_lanceolatus:0.19598642535743121540)99:0.16241418146924860300)48:0.02553182382686622343)52:0.05838616313016836701,(Asterropteryx_semipunctata:0.14050905345951611447,Asterropteryx_ensifera:0.08156045579039897242)100:0.23492568643316291688)38:0.08420914558139147577,(((Oplopomus_oplopomus:0.45524550787954365383,(Exyrias_belissimus:0.22934546233486133304,(Istigobius_decoratus:0.00645094206367500221,Istigobius_rigilius:0.02332854243677643866)100:0.18856685897684546949)70:0.06309984187986845960)9:0.02382767215669365918,(((Acentrogobius_caninus:0.37332110844703880748,(Kraemeria_cunicularia:0.26299272165812043189,Kraemeria_bryani:0.31748800971124818693)99:0.26364473843315228141)5:0.10217091594254289200,((Cabillus_tongarevae:0.43606460495478421846,(Glossogobius_aureus:0.07123951564081557053,(Awaous_melanocephalus:0.36970679542863854605,Parapercis_ommatura:0.15638278100819952288)73:0.11410978137325361592)99:0.27962306275818732404)3:0.05815025056044686308,Arenigobius_bifrenatus:0.29773310488273285745)1:0.00772414291382966280)0:0.06348171839702292096,((((Cryptocentrus_albidorsus:0.14179732156473895399,((Cryptocentrus_leptocephalus:0.12669133147970007558,Cryptocentrus_lutheri:0.14399158432123151941)93:0.05135115612042999433,Cryptocentrus_inexplicatus:0.15257151035942939643)75:0.03998929691128649822)39:0.02392533059915885954,Cryptocentrus_nigroocellatus:0.17191736099200422005)95:0.09486285174925504493,((Cryptocentrus_cinctus:0.12829864778415098203,(Cryptocentrus_cryptocentrus:0.10234684540649081896,(Cryptocentrus_pavoninoides:0.10472765604881573154,Cryptocentrus_cyanotaenia:0.13222882709908245769)53:0.03936883366190278843)48:0.02763424389205660150)91:0.14739663942561076482,Mahidolia_mystacina:0.28125963988929042481)34:0.05682059899827863331)29:0.04518759643562501871,(Myersina_filifer:0.19267958618230485168,Stonogobiops_xanthorhinica:0.19911445075446618236)86:0.14898594895307670782)49:0.06332171661319473122)0:0.04558887681718410551)0:0.06444029489396620181,(((Caffrogobius_saldanha:0.31204248207420065553,((Coryogalops_anomolus:0.48749532970367054352,Caffrogobius_caffer:0.45959535573539800346)67:0.35941299274403426534,((((Mesogobius_batrachocephalus:0.13899477155667072648,((Proterorhinus_semilunaris:0.09724590435338002770,Proterorhinus_marmoratus:0.17710330862862280754)100:0.08225953246333286839,(Neogobius_gymnotrachelus:0.10971906699436659305,(Ponticola_syrman:0.05457803679112137318,((Ponticola_gorlap:0.02217140037335406377,((Ponticola_ratan:0.00227318816838410157,Ponticola_eurycephalus:0.00118890475674330188)100:0.01081457782002244808,Ponticola_kessleri:0.01478838578850736989)100:0.03126669873141330047)99:0.05112125815326853256,((Ponticola_rhodioni:0.01414739614577576951,(Ponticola_constructor:0.00368704286401845419,Ponticola_platyrostris:0.01199285468452110891)70:0.00277648431741405518)99:0.02172611030736614629,(Ponticola_cephalargoides:0.02116380554312394247,Ponticola_cyrius:0.03122035140690062466)59:0.00758856856349399839)84:0.02782197511965679021)98:0.03281871081859125128)96:0.04569536465699999828)98:0.08749122873076624340)30:0.00000176456401954691)98:0.12131726266690155691,(Caspiosoma_caspium:0.11957149781601415783,((Benthophilus_mahmudbejovi:0.00464959133544000196,Benthophilus_stellatus:0.00878701244102261456)100:0.01762788633772429184,(Benthophilus_granulosus:0.02959144955880085165,Benthophilus_abdurahmanovi:0.03199070497924902390)97:0.02736605497287230085)100:0.09722334002249123641)97:0.07579453548773230909)76:0.04524186758143406567,(Neogobius_fluviatilis:0.07748284005053167334,(Neogobius_melanostomus:0.08248379653204800099,Neogobius_caspius:0.04909021042750106451)81:0.02089722828088013892)97:0.09071975679496817324)83:0.15680591647097980035,((Gobius_cruentatus:0.00059454257774636755,Gobius_paganellus:0.10741627313280889544)99:0.20605300689396380265,(Zosterisessor_ophiocephalus:0.09114130489602832730,(Gobius_bucchichi:0.09161658802586859773,(Gobius_xanthocephalus:0.00000176456401954691,Gobius_fallax:0.00434890374047800735)100:0.14407752810552362788)56:0.03010076812332411478)94:0.10444454632082807166)73:0.07859433932457339511)24:0.04182866339993637772)1:0.00222932959541638888)1:0.04501219996212199592,(Chromogobius_zebratus:0.43389708894933504135,Gobius_niger:0.23900292190315383656)28:0.09377239089606669020)1:0.11295789816491194624,((Psammogobius_biocellatus:0.42965543427771624163,(Paragobiodon_lacunicolus:0.11114010924698175531,Paragobiodon_modestus:0.16495254190560329488)100:0.26473992180744460034)7:0.03513619347832907386,(Gobiodon_ceramensis:0.06747149486951990738,(Gobiodon_histrio:0.10381399670850943728,(Gobiodon_okinawae:0.18910333418878272349,Gobiodon_quinquestrigatus:0.17926571854937675687)51:0.05922364896737752210)60:0.04114212445285337499)95:0.14422725558525023581)9:0.09520019103771022728)0:0.02614983533520321562)0:0.01156667738202226368)0:0.02644033810564682882)0:0.01698190151807565593)0:0.01737744450282268163)0:0.01355622645608124677,((((Nes_longus:0.41871639023254392242,Pycnomma_roosevelti:0.27987194943985937989)28:0.19280804149689231841,Barbulifer_ceuthoecus:0.38897750759135613086)0:0.03269819007108441811,(((Aphanius_anatoliae:0.39862109760745745302,Aspidontus_taeniatus:0.36159926415207255257)0:0.08266763404196296461,(Omobranchus_banditus:0.19138844531132456761,Omobranchus_woodi:0.07998455852453317805)98:0.25777411602727018369)0:0.04282119888116898282,(Knipowitschia_caucasica:0.25282148071489518593,(Pomatoschistus_minutus:0.15294376066070419773,Pomatoschistus_tortonesei:0.12363609841022024649)95:0.08889470311180164286)83:0.25568358129236312681)0:0.03951827522835513889)0:0.05440841831256176125,((Pteragogus_flagellifer:0.36666192539455660926,(((Gnatholepis_scapulostigma:0.05338053712211381779,Gnatholepis_thompsoni:0.08139729412165939981)100:0.10480443144917177267,(Gnatholepis_anjerensis:0.00804642202815695866,Gnatholepis_cauerensis:0.00000176456401954691)100:0.17248616980119357844)93:0.12561732137350989369,((Oligolepis_keiensis:0.25886185903409592024,((Stenogobius_polyzona:0.00000176456401954691,Stenogobius_hawaiiensis:0.00993995890175237290)100:0.28492595937454118227,(((Cotylopus_acutipinnis:0.06443365999217423590,Cotylopus_rubripinnis:0.06412563150703121106)100:0.10017771964616339897,((Sicydium_punctatum:0.09326323443260541024,((Sicyopterus_lagocephalus:0.08714221332295701838,Sicyopterus_pugnans:0.12282828873429441074)91:0.06963930369922044505,Sicydium_plumieri:0.09565727698167603155)58:0.02316028746079494202)79:0.04946643373030130270,(((Sicyopus_chloe:0.05698579943011311544,Sicyopus_sasali:0.03483275951776819424)100:0.10978134925690810619,(((Akihito_futuna:0.05750225194350639529,Akihito_vanuatu:0.04852904636742784439)96:0.05869481374170302385,Sicyopus_zosterophorum:0.08705734498681304279)71:0.02975059613698216587,(Lentipes_armatus:0.02611039623559996067,Lentipes_kaaea:0.03177097119321476565)99:0.07943924692533756160)30:0.01517623623729875623)16:0.01674122361629068814,((Stiphodon_atratus:0.00000176456401954691,Stiphodon_elegans:0.00491383144861359984)99:0.09880487986315872329,((Stiphodon_sapphirinus:0.00886232162823167441,Stiphodon_hydroreibatus:0.00003722954728608116)100:0.10295217646562017377,(Stiphodon_rubromaculatus:0.03470765596585200646,Stiphodon_rutilaureus:0.07704187456417302404)98:0.02132316524854016224)96:0.02807198368192613189)83:0.07188053054420112664)18:0.02375510413994762551)9:0.00805929249107522304)24:0.01888578912406375584,(Awaous_guamensis:0.10908971121877572563,Awaous_aeneofuscus:0.07779451003238847806)100:0.12953887440978054113)28:0.03670112814694872355)67:0.04327629298294573962)14:0.04867992251189486030,(Oligolepis_acutipennis:0.27120784313564677559,((Gobioides_broussonnetii:0.18864828694091304029,Gobionellus_oceanicus:0.21967547143142274191)84:0.12602957833821473721,(Favonigobius_gymnauchen:0.20692182653235002832,((Ctenogobius_sagittula:0.27199609979956435035,Ctenogobius_boleosoma:0.21328874837416425536)23:0.04524775906145903775,Ctenogobius_saepepallens:0.26989852260764674030)74:0.12832498568766304015)73:0.14314638570512538962)19:0.00000176456401954691)31:0.04745153108209579362)3:0.02229464292205437376)51:0.02825495343347151841)2:0.02458966756379599508,((((Eucyclogobius_newberryi:0.30536474386853934559,Lethops_connectens:0.15159561889018025038)75:0.09526808440706689696,((Typhlogobius_californiensis:0.21405341539219355229,(Gymnogobius_castaneus:0.20925696917321931489,(Chaenogobius_annularis:0.12770831571869398213,Gymnogobius_urotaenia:0.07187994594633620449)94:0.07585633267905578236)90:0.08665550311892628033)37:0.02281653208107356501,Gillichthys_mirabilis:0.25448745124126825878)52:0.03195318226123249061)71:0.08538168606860646603,((Acanthogobius_hasta:0.17377481461247365480,Acanthogobius_flavimanus:0.13633088775200521980)100:0.34950994640595517415,((Gobiopterus_semivestitus:0.46920505698755859969,(Periophthalmus_barbarus:0.34897399891773989156,(Pseudapocryptes_elongatus:0.34060719384846432911,(Odontamblyopus_rubicundus:0.40393686043995113977,Scartelaos_histophorus:0.18967526910616006042)20:0.01212615843035835632)63:0.09018452732128026672)38:0.03939435352256891176)2:0.03263793146155150038,(Rhinogobius_brunneus:0.21900299630811853602,Rhinogobius_giurinus:0.17754177763136608226)77:0.07752880567440191550)2:0.01624355850857507610)0:0.02007692169711004088)0:0.02455457902312781562,(Amblychaeturichthys_sciistius:0.43816137879773336961,Butis_butis:0.40320434768613377940)13:0.11731683882983459655)0:0.00620865778489399639)0:0.02750085559088782591)0:0.03702319739905285401)0:0.01300078962587114875,((Tridentiger_brevispinis:0.13889247966966788983,Tridentiger_obscurus:0.30208388436765370999)92:0.22064207779301359125,((((Oxyeleotris_marmorata:0.17757647246255073292,(Oxyeleotris_lineolata:0.06721948732987005626,Oxyeleotris_selheimi:0.09201751391278560621)99:0.08100891651921600378)96:0.06365315476212446422,Bostrychus_sinensis:0.33432584086734340456)81:0.05218383499856433377,Kribia_nana:0.56990234592894584154)76:0.11693234699985399772,(((Microphilypnus_ternetzi:0.44335594386761828511,((((Gobiomorphus_coxii:0.18351990862736361487,(Gobiomorphus_breviceps:0.10521229156564343432,Gobiomorphus_hubbsi:0.13833623919769785782)94:0.06672523147717716874)74:0.07301785283516214198,(((Leptophilypnus_fluviatilis:0.23757847637717366407,Leptophilypnus_panamensis:0.46772386619268080388)82:0.18992014539200852141,(Philypnodon_macrostomus:0.43942854867328534629,Philypnodon_grandiceps:0.26033727845321902317)11:0.04567871815256693835)6:0.08005271163322076611,Gobiomorphus_australis:0.23840318664897286460)1:0.04352401725626190571)1:0.03892467696528428833,(Gobiomorus_maculatus:0.29851422511075476063,(Hemieleotris_latifasciatus:0.17178215805172317165,Gobiomorus_dormitor:0.14889383046407861366)50:0.03114216590202756665)46:0.05978271227011263539)1:0.02179690995359783237,((((Hypseleotris_klunzingeri:0.10998742354594935600,Hypseleotris_compressa:0.04754178662191320404)66:0.00447985921060632761,Hypseleotris_aurea:0.04901020586606661539)59:0.02430133274529434895,Hypseleotris_galii:0.05436150388104447895)100:0.18438412849425594997,((Calumia_godeffroyi:0.28474630058443956004,Erotelis_armiger:0.33934483343646631992)28:0.05082928623961555786,(((Eleotris_amblyopsis:0.15772230375244936096,Eleotris_fusca:0.12756211908980377712)96:0.06338447092454685405,Eleotris_sandwicensis:0.25281763565179871112)87:0.04822014606886677879,(Eleotris_picta:0.00947806591603318066,Eleotris_pisonis:0.00214507733349969575)100:0.23209338073043483952)41:0.02689427495152648187)9:0.02729509072439618989)2:0.02508574154695871611)0:0.02518815660697313469)1:0.00000176456401954691,(Guavina_micropus:0.20357246178682544824,(Dormitator_maculatus:0.05638360004356516436,Dormitator_latifrons:0.06718196676971215087)100:0.09243319082308491808)92:0.10488754275250862513)9:0.02707630617187654562,(Ratsirakia_legendrei:0.29406426222194576026,((Mogurnda_mogurnda:0.03648106883743473111,Mogurnda_adspersa:0.00335248114923191723)100:0.22242648566648356989,(Tateurndina_ocellicauda:0.36050392549463916581,Ophieleotris_aporos:0.14912101752567427648)9:0.00000176456401954691)97:0.11125733496156693658)82:0.08346988927247001033)18:0.04755767666110613523)4:0.02476257647003272250)0:0.04205367780470984501)0:0.02862722614321663861)0:0.02238418967795650916)0:0.01612254937968342350)0:0.01546189372761208949)0:0.00000176456401954691,((Aulostomus_chinensis:0.70307510291731645236,(Prionotus_ophryas:0.18478249975432928243,(Bellator_xenisma:0.22887242571509006095,Prionotus_stephanophrys:0.24595660501302493706)43:0.02279767848276772688)98:0.21297123095394410175)5:0.10028932567934815967,(((((Cociella_crocodila:0.29457954477397185755,Inegocia_guttata:0.32198212116714941544)47:0.04914239359594051865,((Rogadius_prionotus:0.19065735670382863431,Rogadius_portuguesus:0.17360342422607186186)93:0.19766700629629266328,(Cymbacephalus_nematophthalmus:0.32954509262127473779,(Cymbacephalus_staigeri:0.20751801610054745884,Papilloculiceps_longiceps:0.21470488329167891628)82:0.07131937736995665189)69:0.07840494884980914181)32:0.03231705455694298956)12:0.03194808102852535886,Kumococius_rodericensis:0.32758507449569540526)54:0.14489703850439389354,((Mastacembelus_moorii:0.01336023853636944016,((((Mastacembelus_platysoma:0.09635111476892949112,Mastacembelus_micropectus:0.04981466396602627494)36:0.01254284515551748989,Mastacembelus_ophidium:0.01784783057496918540)25:0.01270016580707044630,((Mastacembelus_tanganicae:0.00639350335015819501,Mastacembelus_albomaculatus:0.01685872322479829710)87:0.03647612498304521333,((((Mastacembelus_cryptacanthus:0.13009606158796491182,Mastacembelus_taiaensis:0.17295608500170064903)61:0.04766596808016811820,(((Mastacembelus_signatus:0.02450363908182555742,Mastacembelus_brachyrhinus:0.02554826527165660749)100:0.13656477842249098442,Mastacembelus_nigromarginatus:0.14612380656707366078)18:0.00416981050102784023,((Macrognathus_siamensis:0.24661560630421636153,(Macrognathus_pancalus:0.09236884636122316694,Macrognathus_zebrinus:0.12240617801925285368)83:0.04583348529306671693)47:0.04122106327860569630,Mastacembelus_armatus:0.16953795015844583260)59:0.05974477596635241472)6:0.00746872891128035413)35:0.03549788388013049717,((Mastacembelus_vanderwaali:0.01367567311988244008,(Mastacembelus_frenatus:0.02090796771102442983,Mastacembelus_shiranus:0.03052912004978731997)62:0.01423516911740430047)44:0.00874398734999998033,Mastacembelus_stappersii:0.02750559540949889734)100:0.13828102695845517767)42:0.04295621725101213956,Mastacembelus_cunningtoni:0.02717780944000432880)31:0.01815453747862381950)16:0.01173218382858209723)5:0.00639777451900465453,(Mastacembelus_ellipsifer:0.03095066322703437373,(Mastacembelus_flavidus:0.02113431900401705268,(Mastacembelus_zebratus:0.00461525753515634480,Mastacembelus_plagiostomus:0.00671790975352313646)75:0.00714855185087040577)71:0.00474948715077076061)65:0.01068333382473681155)25:0.03204088682089163004)96:0.28435340652941543116,(Neoplatycephalus_aurimaculatus:0.32856044372799014797,(((Platycephalus_bassensis:0.10154663457115779834,Platycephalus_longispinis:0.09612006030538777779)95:0.09418340124399772473,((Platycephalus_laevigatus:0.11503759581560382697,Platycephalus_marmoratus:0.13573983622099136670)48:0.03568134757796321416,(Neoplatycephalus_speculator:0.09043749602200686077,Platycephalus_caeruleopunctatus:0.07668502116524450463)66:0.06423741028104022754)17:0.00864824735166308013)38:0.02384099918022693915,(Platycephalus_endrachtensis:0.20571597634717922465,(Platycephalus_fuscus:0.08254245913765180520,(Platycephalus_westraliae:0.08418408336618031507,Platycephalus_indicus:0.08615165493102498739)88:0.05124177679175010575)98:0.13123801663215062252)55:0.02726205921246405844)51:0.05086944432537004318)93:0.12504496788485580661)7:0.05776236931298064231)0:0.00205972093041975656,((Synchiropus_atrilabiatus:0.47372266133733237359,Xiphister_atropurpureus:0.50405774311950679589)24:0.28452248153436265898,((Channa_micropeltes:0.10229417266864314351,Channa_diplogramme:0.09137968065628117309)100:0.25162682936694719116,(Channa_striata:0.33001562652351501548,(Channa_punctata:0.18597551537561884949,(Channa_marulius:0.26816452975470189690,((Channa_gachua:0.10672531174657937103,(Channa_aurantimaculata:0.07927000444836490578,Channa_stewartii:0.15509825659877907178)68:0.04000617428739341314)63:0.05517940819858038748,(Channa_barca:0.06940500464331085528,Channa_bleheri:0.00222010300361761897)100:0.13836310940356691401)100:0.24175262545176454121)14:0.01190825558513949542)39:0.06683793727858873546)77:0.09627666162101310154)64:0.10152693352191595466)0:0.01176037312985039190)0:0.00475041210886998841)0:0.00783485221490410472)0:0.01249691348310430668,(((((((Parapercis_colias:0.49145404904542655533,(Parapercis_hexophtalma:0.21106583162396969078,Parapercis_punctulata:0.41442173553805961017)60:0.19602030041552123696)12:0.10835949349946792652,(((((Pervagor_melanocephalus:0.06104944163864303719,(Pervagor_aspricaudus:0.04475784627836958129,Pervagor_nigrolineatus:0.05075730046335492213)40:0.01828997936456159407)24:0.00000176456401954691,Pervagor_spilosoma:0.05981843781204371213)92:0.28160192956087171101,(((((Cantherhines_verecundus:0.14161171906437344803,Cantherhines_pullus:0.26162832034419286620)85:0.11691514207374087420,((Thamnaconus_modestoides:0.00000176456401954691,Thamnaconus_modestus:0.00221515864617760671)100:0.02893636109994367211,(Thamnaconus_septentrionalis:0.01976472844478625174,Thamnaconus_arenaceus:0.01810323967517463162)93:0.01645078746973569969)97:0.11773767618095772047)48:0.06532565927139874495,(Anacanthus_barbatus:0.51308793373338967836,(Aluterus_monoceros:0.13873840638585679952,Aluterus_scriptus:0.14445035250346677236)69:0.01055421923193081667)10:0.07421625903742051777)4:0.05353009138086674973,((Oxymonacanthus_longirostris:0.34603124657998557412,(Stephanolepis_setifer:0.03199289512023687637,(Stephanolepis_cirrhifer:0.00215165738528601399,Stephanolepis_diaspros:0.00000176456401954691)80:0.00000176456401954691)100:0.48511342463099549338)32:0.10669062879549245926,(Paramonacanthus_sulcatus:0.13016168457031793149,Monacanthus_chinensis:0.09846546461190072708)96:0.17299244517853801240)5:0.00000176456401954691)5:0.06948088859949147489,Monacanthus_ciliatus:0.35732924661921783294)6:0.04038542026108547095)2:0.05750271948393015364,((Cepola_pauciradiata:0.00000176456401954691,Cepola_macrophthalma:0.00452589636224545993)100:0.56686644460731971940,(((((Abalistes_stellaris:0.26414813314394214316,(Rhinecanthus_verrucosus:0.06267601439741013469,(Rhinecanthus_rectangulus:0.05108840405379453525,Rhinecanthus_aculeatus:0.04968911539134310273)38:0.01898407282598205820)79:0.14199382343941760465)21:0.02388288146530141362,(Canthidermis_maculata:0.21572270254998401895,(((Odonus_niger:0.07096390117863957125,Melichthys_niger:0.03102257097669918612)86:0.10614367712152923684,((Balistoides_conspicillum:0.13707946473015750199,(Melichthys_indicus:0.03299706290296265665,Xanthichthys_ringens:0.02204302120481515387)94:0.08486333314546239126)30:0.01329504331895825682,Balistapus_undulatus:0.21131974615706994314)33:0.02613819043352085675)34:0.02837152484728305488,((Xanthichthys_mento:0.00680402508038792288,(Xanthichthys_lineopunctatus:0.01511271060363033117,Xanthichthys_auromarginatus:0.00980803270851275145)71:0.01785671311122613222)96:0.07335358192858822124,Pseudobalistes_flavimarginatus:0.22182920301035533028)95:0.13142471686163259559)38:0.05478967951256337454)11:0.01068869255185117724)19:0.04992787590534486508,Pseudobalistes_fuscus:0.20691168197736437007)13:0.02278468973582234436,(Balistes_polylepis:0.08149691888089018177,(Sufflamen_bursa:0.05279244644864703601,Sufflamen_chrysopterum:0.06563992670445657762)57:0.04647072929049066853)86:0.16084506530463921203)28:0.03459478440999862875,Balistes_capriscus:0.26572684279464486856)67:0.21104734972113120550)1:0.02865891392095673909)0:0.03339944976912015417,((Ctenopoma_acutirostre:0.47883458308377474522,((((Gerres_filamentosus:0.32176968721673931961,(Gerres_oblongus:0.01368401923609042538,(Gerres_equulus:0.00000176456401954691,Gerres_japonicus:0.00000176456401954691)100:0.00000176456401954691)98:0.12985338355343784000)90:0.15042463338814041074,(Gerres_erythrourus:0.08996286853031527919,Gerres_limbatus:0.29795913531733186908)95:0.17196212074133715442)33:0.03421748245567618474,Gerres_cinereus:0.21528566387406947169)52:0.06931705336529737915,(((Eucinostomus_melanopterus:0.12686027109119771272,Eucinostomus_currani:0.11463854259811022407)96:0.12035933315392316478,(Eucinostomus_argenteus:0.16901876076771446544,(Eucinostomus_jonesii:0.14413759982445906171,Eucinostomus_gula:0.15425145771480469614)58:0.07122906651768808051)53:0.04754940597692449561)44:0.03977154998622986493,Eucinostomus_havana:0.32575752274980529499)91:0.13870104262471030787)56:0.11078191833298481339)5:0.04809389285977540979,(Meiacanthus_smithi:0.50314155939508464765,((Priacanthus_macracanthus:0.17297073003857518780,((Priacanthus_meeki:0.03252309530358007961,Priacanthus_hamrur:0.02940286210027804464)100:0.15572500151945173341,Priacanthus_tayenus:0.30339924977383836424)48:0.02758488681697608344)97:0.26983093289961096461,((Bairdiella_sanctaeluciae:0.20583995285848341594,Bairdiella_ronchus:0.23561004233185633971)93:0.10747300348123238478,((((Aplodinotus_grunniens:0.17522371720385232496,(((Pogonias_cromis:0.17916590952064206932,(((((((Johnius_elongatus:0.00327286987296874143,(Johnius_dussumieri:0.00717620599468061105,(Johnius_vogleri:0.00000176456401954691,Johnius_borneensis:0.00422909918847433949)100:0.21514372288446387271)48:0.03086537810299815054)58:0.09727074493215841222,Otolithes_ruber:0.03019258686434251465)57:0.12386704641736280552,Otolithes_cuvieri:0.19028001241852079972)58:0.19822681094437141769,(((Pennahia_macrocephalus:0.04780300395094706778,Nibea_albiflora:0.01374102296277314450)99:0.23723374717841611004,((Protonibea_diacanthus:0.15880039953040031575,(Pennahia_anea:0.07123580012310259946,Pennahia_argentata:0.00000176456401954691)100:0.19516095865668173226)29:0.04792620240813067617,(Chrysochir_aureus:0.27264433556738870701,(Parapercis_robinsoni:0.43606488414424354749,(Nibea_maculata:0.19822407879920048157,Dendrophysa_russelii:0.24876718548141152354)87:0.02766416491160716973)59:0.14432877732154200667)18:0.02688021956221799819)7:0.01522761115786713630)8:0.01939256406994464485,(Larimichthys_crocea:0.34946136310825387516,Miichthys_miiuy:0.10523547094031143745)26:0.02606689650521144472)9:0.04220763408119227594)22:0.04850176049161716851,((Argyrosomus_hololepidotus:0.02360989570604125551,Argyrosomus_japonicus:0.01360026989614783639)98:0.04185479132499630994,Argyrosomus_inodorus:0.04012501822874178781)87:0.07563936742142396419)4:0.00568513292202649494,Otolithoides_biauritus:0.32576328332740200899)5:0.01343016379204581626,(Sciaenops_ocellatus:0.11751176845022273665,(Plagioscion_auratus:0.25626452408477201628,(Menticirrhus_undulatus:0.05224957019504557904,Menticirrhus_americanus:0.18848633076202286896)99:0.27619813619584959730)9:0.01185277881186189153)3:0.04116583255530798652)2:0.01583497925939527512)6:0.02465925732865624498,Leiostomus_xanthurus:0.16930561595189530522)2:0.01550053075209137377,((Bairdiella_chrysoura:0.01054186743135879754,(Micropogonias_furnieri:0.02744926085024840257,(Roncador_stearnsii:0.00690937313952849196,Micropogonias_undulatus:0.00000176456401954691)100:0.08252071057352980410)54:0.01280309847165907351)97:0.17107592388839659714,((((Cynoscion_nebulosus:0.13942321988127739929,(Cynoscion_regalis:0.04534174300255764539,Cynoscion_arenarius:0.03543116311818183400)81:0.03158397593587068675)38:0.01918117779363740297,Cynoscion_nothus:0.08859807246854504992)31:0.02708642910093356226,(Cynoscion_virescens:0.13533542288095556927,Cynoscion_parvipinnis:0.14841877076019921455)31:0.02090189379894202837)39:0.04234501685856351810,(Atractoscion_aequidens:0.09419494606156748884,(Cynoscion_guatucupa:0.17681102792706121507,Atractoscion_nobilis:0.04497889302294448555)25:0.00982593119315564555)54:0.03450719730509032440)41:0.01909638270403745189)11:0.01289936108873416219)0:0.01479932352604734595)8:0.03746387417121143509,Umbrina_roncador:0.22202681956291039911)4:0.03835504734651967729,((Pareques_acuminatus:0.16590966417217359319,(Equetus_lanceolatus:0.02723985017806019801,Equetus_punctatus:0.06269447806659414479)99:0.06609110663850226164)88:0.03218390452831625115,Cheilotrema_saturnum:0.15276262605470539158)91:0.09131433164099432565)6:0.03170608042044260888,Genyonemus_lineatus:0.07350375356296949547)5:0.05249235362796254162)63:0.15626727086398312094)21:0.07963369102826822754)0:0.03334786509234901469)0:0.01730168410449310634)0:0.00727531737569306596)0:0.01859677274963192697,((Monotaxis_grandoculis:0.13840243734286547528,Gnathodentex_aureolineatus:0.28751806968070525716)43:0.02087086341678030463,(((Gymnocranius_audleyi:0.11431168825231527442,Gymnocranius_grandoculis:0.10299384223696123053)51:0.04028882912811637684,Gymnocranius_elongatus:0.07688428992097889880)93:0.06916384039596119859,Neostethus_bicornis:0.51684090483199351951)7:0.12723322733396594253)3:0.12231732403898804007)0:0.01165405050735558896,((((Achirus_lineatus:0.19817136531763385188,(Trinectes_inscriptus:0.12785335181518633907,Trinectes_maculatus:0.17029193482782867264)98:0.15907711459954018696)97:0.41415331988052095369,((((Chascanopsetta_prorigera:0.80868642641129762083,Citharoides_macrolepis:0.31165799194054516486)19:0.08347806628309899224,(((Citharichthys_xanthostigma:0.00889913029470448987,Citharichthys_sordidus:0.00281384356367374213)100:0.21032571703877656066,Citharichthys_stigmaeus:0.26554409984475318973)97:0.24969994291113792317,(((Cynoglossus_interruptus:0.52597315988888515559,(Cynoglossus_canariensis:0.35411209536868437642,((Cynoglossus_capensis:0.11060116706269212949,Cynoglossus_zanzibarensis:0.16552250331611484402)99:0.30692607629613416487,(Cynoglossus_browni:0.23312366575177387307,(Cynoglossus_sinicus:0.00858222752766831312,Cynoglossus_senegalensis:0.00856606714197019441)81:0.29349450094453688465)51:0.13359285204173554362)27:0.07493988490984870854)40:0.13530901289781430141)4:0.02419720319069835687,(((Cynoglossus_bilineatus:0.28226202195870819800,(Cynoglossus_macrostomus:0.02223116805565279394,(Cynoglossus_puncticeps:0.00000176456401954691,Cynoglossus_lingua:0.00000176456401954691)100:0.00000176456401954691)100:0.29914728325040135237)66:0.10608748754366326139,((Cynoglossus_marleyi:0.25425862796783055275,((Cynoglossus_abbreviatus:0.00231086283712284924,Cynoglossus_purpureomaculatus:0.00000176456401954691)100:0.10837795015543276456,Cynoglossus_semilaevis:0.18635150792750865278)98:0.20059889070339226302)57:0.08229165098274728030,Cynoglossus_itinus:0.32078478829433865194)36:0.06116355715898100925)27:0.08501081290661713363,(Cynoglossus_joyneri:0.01213962344279449421,Cynoglossus_lighti:0.00000176456401954691)99:0.25265659169959331365)42:0.07984118152634731014)26:0.16281369351050783978,Cynoglossus_dubius:0.95576012097507212495)94:0.28833620202074722316)2:0.03613949265306719283)0:0.01316042945364544886,((Citharus_linguatula:0.62183269123529139044,(((((Cyclopsetta_fimbriata:0.27620990832459307862,Syacium_papillosum:0.43023994374531754348)95:0.38762018059399855341,Paraplagusia_japonica:0.45430549256783525625)9:0.13783517170230599747,(Pardachirus_pavoninus:0.36711571401254072811,(Heteromycteris_japonicus:0.24514624480015009822,Pardachirus_marmoratus:0.52181061014489871219)68:0.09173354394832917980)44:0.12590992168583908795)0:0.06875272370110541154,(Brachirus_orientalis:0.50251407969324657365,((Synaptura_lusitanica:0.39592093818184959098,Synaptura_marginata:0.38578926926977785694)100:0.36682255024200527416,(Austroglossus_microlepis:0.03454403057995360238,Austroglossus_pectoralis:0.02117834249979105854)99:0.26723272369795564307)21:0.13095320152078596121)2:0.05726248135382653454)0:0.05691389090709407517,((((Symphurus_civitatium:0.00000176456401954691,Symphurus_plagiusa:0.01796299185637602325)100:0.26696162352524605010,(Symphurus_atricaudus:0.46792110851388513115,Symphurus_thermophilus:0.60044558012314974693)71:0.30539218668458123496)100:0.69682774185993168192,((Buglossidium_luteum:0.38827317008014167188,(Microchirus_azevia:0.15494177701843320216,(Microchirus_variegatus:0.24059389846799009161,(Bathysolea_profundicola:0.19224082551542298614,Dicologlossa_hexophthalma:0.23764757625302654342)34:0.02402073728639336075)43:0.01979912510539569293)79:0.11830544353045512052)58:0.06424947030405096793,((Solea_ovata:0.33357570123091073722,((Synapturichthys_kleinii:0.01804288713486888740,Pegusa_impar:0.00000176456401954691)100:0.18096743446884391848,((Solea_senegalensis:0.06058082979611111724,Solea_aegyptiaca:0.10003232664271775876)93:0.07033490404784221994,Solea_solea:0.07561095835300417345)59:0.04366060902428500062)56:0.07113647707236991913)45:0.03345003889853905010,(Dicologlossa_cuneata:0.37194919919460978086,Pegusa_lascaris:0.29843923791944165780)75:0.09201649465584374465)57:0.05482133846261018351)68:0.07701289892578963570)19:0.02522476210284005024,(Zebrias_quagga:0.43806460250955825408,Zebrias_synapturoides:0.37767438667749819725)33:0.19245359006928020040)1:0.04984734516176098085)0:0.03496608955353584791)0:0.03163531783888918342,(((Neoachiropsetta_milfordi:0.03417647661187540875,Mancopsetta_maculata:0.00000176456401954691)99:0.32850965002620141631,((Ammotretis_rostratus:0.43504989212923250719,Pelotretis_flavilatus:0.42821905351603661849)32:0.08648644065566335470,(Rhombosolea_tapirina:0.42700297798326197762,Peltorhamphus_novaezeelandiae:0.32928050493420191192)74:0.12060725658955361028)39:0.07306203272937988880)41:0.15859170802534888756,(((Paralichthys_orbignyanus:0.15435902703398080016,(Paralichthys_lethostigma:0.29829653073203704627,(Paralichthys_californicus:0.21619592510060198221,Paralichthys_patagonicus:0.03681732861397287587)56:0.04808665229426562632)49:0.02793902206001560792)58:0.08767379443470613154,((Xystreurys_liolepis:0.13885230965529529734,Xystreurys_rasile:0.07991935668181614216)99:0.15041597839059203667,((Pseudorhombus_malayanus:0.23796969171471260562,(Pseudorhombus_natalensis:0.12866566910326657225,(Pseudorhombus_pentophthalmus:0.24537404763344983216,(Pseudorhombus_arsius:0.00000176456401954691,Pseudorhombus_elevatus:0.09007390025289710445)100:0.22282699353897733596)44:0.07083919400962210289)72:0.08094397837429223586)99:0.35507722443200667728,Paralichthys_isosceles:0.22694285766391550374)12:0.03029910620672679814)5:0.01675905557461341427)6:0.04481872248109629309,(((Verasper_moseri:0.03277651554958869312,Verasper_variegatus:0.01246065647355995565)98:0.10198425829636030560,(((((Pseudopleuronectes_americanus:0.03233539748730516333,Pseudopleuronectes_herzensteini:0.02257437845770003323)100:0.03406812012292790431,((Parophrys_vetulus:0.04655548776431320257,(Isopsetta_isolepis:0.03331917369940156498,Psettichthys_melanostictus:0.05261203730636004189)67:0.00803338672798732147)63:0.01444471900991821899,(Lepidopsetta_bilineata:0.07996267756502066504,Lepidopsetta_polyxystra:0.01786554067063560527)64:0.00832600314949104650)65:0.02374284244831073934)64:0.01816249092917744321,((Pleuronectes_platessa:0.04351929291079386503,(Platichthys_stellatus:0.03252569771209964239,Lepidotrigla_abyssalis:0.03844256378781815003)79:0.01386967878159207912)37:0.00534682168333422934,(Limanda_proboscidea:0.01184913941651616169,(Platichthys_flesus:0.03271283168904935007,Limanda_ferruginea:0.04557558388653485532)90:0.00852739978874393796)91:0.02529342790427492257)51:0.01932926377053488867)62:0.01545736563630212626,((((Hippoglossoides_elassodon:0.02988937759631851362,Hippoglossoides_robustus:0.00000176456401954691)68:0.02526700006836264320,(Hippoglossoides_dubius:0.02437055514702360023,Hippoglossoides_platessoides:0.00998010575929612204)100:0.04778651944039638555)56:0.01609253612928020910,(Cleisthenes_pinetorum:0.04295658161513632445,Dexistes_rikuzenius:0.06647304037222123707)93:0.03953995124588071536)36:0.00924564623933319014,(Limanda_sakhalinensis:0.04127192034805516468,(Limanda_limanda:0.01530978798617475128,Limanda_aspera:0.02113508939639023587)100:0.05156114636218768477)78:0.02808777032698453122)97:0.08469483654712567366)87:0.05912780822296519884,((Clidoderma_asperrimum:0.11034301794310970302,(Eopsetta_jordani:0.15631167842913862298,(Reinhardtius_hippoglossoides:0.08029535563687649480,(Hippoglossus_stenolepis:0.01610125771986433557,Hippoglossus_hippoglossus:0.00989603227199610004)97:0.03770900106318220840)34:0.01295904971084518333)9:0.00741292149362386948)19:0.01887561948858938049,(Lyopsetta_exilis:0.15069837969393934385,((Embassichthys_bathybius:0.06941727525043168978,(Microstomus_kitt:0.11047339817103050963,Microstomus_pacificus:0.13376812738015042559)70:0.02035800413226088873)99:0.09746806492375525988,(Glyptocephalus_zachirus:0.02091588090146500808,(Glyptocephalus_cynoglossus:0.04866964305396156293,Glyptocephalus_stelleri:0.01700898497254816483)86:0.01237511114826814543)97:0.06730579613977293041)63:0.04915805087107000260)16:0.01057864821096980538)16:0.02068232437884878760)11:0.02199238548717296834)78:0.10251846776434452024,(Atheresthes_stomias:0.43737423232897254533,(Hypsopsetta_guttulata:0.26566187319836415126,(Pleuronichthys_ritteri:0.09208856850203810118,(Pleuronichthys_coenosus:0.08587463557596959618,(Pleuronichthys_decurrens:0.13111906512534049485,Pleuronichthys_verticalis:0.08699610417428300935)66:0.04320561128131854278)50:0.04648054587127617432)95:0.09789801739301977335)49:0.05304834177547756296)10:0.00246216965014512670)32:0.02809950297657438228)19:0.08795474997982449805)1:0.04134777346966549139)0:0.01223802508961752133)0:0.00823820532237989206,(Laeops_pectoralis:0.49670407949314437923,((((((Bothus_ocellatus:0.08482331813607674187,Bothus_podas:0.15585171366520914060)91:0.09570477750457871691,Bothus_lunatus:0.33299390429575093098)94:0.16586270419412468824,Parabothus_chlorospilus:0.37811099069178838450)41:0.05723047896326978468,(Taeniopsetta_radula:0.37674700624650225844,(Crossorhombus_valderostratus:0.56537072945989874651,(Arnoglossus_rueppelii:0.26568059282240102936,(Arnoglossus_laterna:0.36453266427483516088,((Arnoglossus_capensis:0.03734272531468012940,Arnoglossus_imperialis:0.05736127573807098257)64:0.04424287927092692557,Arnoglossus_thori:0.11916921271558658657)55:0.05937949468185003965)17:0.02790942055347557169)71:0.14929500194021333437)40:0.11673811129749112059)4:0.05625144840471946184)12:0.01641709411473355959,Bothus_leopardinus:0.62181077249141247787)11:0.03350548893143884266,(Engyprosopon_xenandrus:0.27035640115158338093,Arnoglossus_polyspilus:0.32961095233020404338)53:0.09288556217676292859)11:0.02607079353938842464)17:0.14268611447448864560)0:0.01235004927377877926)0:0.02295879911992584899,(Polycentropsis_abbreviata:0.55258747874435543146,(Psettodes_erumei:0.15876571620756343983,Psettodes_bennettii:0.15041166730450397648)98:0.15452843583653055881)13:0.07203989348116254798)0:0.01424121008068274588,(((Rachycentron_canadum:0.70470116464672194478,(Carangoides_praeustus:0.21550627540219494116,(Selaroides_leptolepis:0.20856664382853315476,((((Decapterus_macrosoma:0.01195130507780183676,Decapterus_muroadsi:0.00000176456401954691)100:0.09971410299742379524,(Decapterus_akaadsi:0.01953792548334560425,Decapterus_russelli:0.03580861738297352187)42:0.03052907418104243431)96:0.05668030161919323379,(Decapterus_punctatus:0.13125248148736526344,(Trachurus_novaezelandiae:0.00255316194094448940,((((Trachurus_murphyi:0.00302557340433921374,Trachurus_picturatus:0.01055512849890889553)89:0.01375965929021763859,(Trachurus_trachurus:0.00000176456401954691,Trachurus_capensis:0.00268048459726148323)97:0.00943727443805844092)35:0.00490426668512903684,Trachurus_mediterraneus:0.00337683044637513124)42:0.00523202638984964982,(Trachurus_japonicus:0.00000176456401954691,Trachurus_declivis:0.00000176456401954691)94:0.00000176456401954691)47:0.00638466579613138682)75:0.03525269310225833536)63:0.02255266091780065707)91:0.06905958701667072597,((((Selar_crumenophthalmus:0.00285566157918628639,Decapterus_macarellus:0.01102384036777594167)99:0.00866200864151077908,Selar_boops:0.02243929927041155709)100:0.23619227446894250355,((Pseudocaranx_wrighti:0.09200384834837989356,Pseudocaranx_dentex:0.06056483700995476255)66:0.03015042282685491057,Carangoides_equula:0.04735376215631681429)81:0.03773518950096319685)64:0.03694066390870222316,((((Atule_mate:0.10712331429350176182,(Alepes_kleinii:0.00692314609817302161,Alepes_djedaba:0.00000176456401954691)100:0.12064033021269064683)41:0.02725576278381734024,(Caranx_carangus:0.09799541776276400606,Gnathanodon_speciosus:0.15436068440285580228)43:0.02906400648151125435)15:0.02612975665424500937,(Caranx_vinctus:0.17155469567555198251,((Carangoides_ruber:0.15874565159036754802,Caranx_crysos:0.08056995169299104032)86:0.04953544528793655394,((Megalaspis_cordyla:0.15371830987007531477,(((Carangoides_bartholomaei:0.00227184768271828730,Caranx_sexfasciatus:0.00230562726418300726)100:0.02934536106142188530,(Caranx_caninus:0.09841577803640563782,Caranx_papuensis:0.05586178817301077487)42:0.02280778298309109678)39:0.02150511141496853212,(Caranx_tille:0.03420957492320730181,(Caranx_hippos:0.00289107628542546345,Caranx_ignobilis:0.00613857720080657459)100:0.09921363519381709573)43:0.01414051320970954352)47:0.02481084921119584494)61:0.02930474027047934651,Caranx_bucculentus:0.10102040204970330617)32:0.01067401397908380838)86:0.06297151748714194253)10:0.01478521535308313130)33:0.03564579853269105730,((((Alectis_indicus:0.00227546442180005673,Alectis_ciliaris:0.00000176456401954691)100:0.09489067605849588194,((Selene_setapinnis:0.32308783655115796973,Selene_peruviana:0.07118233974975571310)17:0.02505908435365011150,(Selene_brevoortii:0.04270172653596685658,Selene_vomer:0.04378056718939553338)99:0.07432595208248153618)27:0.07496914996083320348)9:0.00196649775075336160,((Carangoides_coeruleopinnatus:0.08991102842687716257,(Atropus_atropos:0.15992674773344414674,Carangoides_fulvoguttatus:0.07376689266464526995)24:0.01545327189549041726)42:0.02645111439204417270,(Caranx_melampygus:0.01118421396003817583,(Carangoides_malabaricus:0.00432777019867365293,Carangoides_chrysophrys:0.00228467220853831657)54:0.00462816624688430853)96:0.17748553004993175564)42:0.02538189913804437745)4:0.00800116034137619142,(Parastromateus_niger:0.12639262721922081556,Uraspis_helvola:0.15158823295126994113)77:0.05855560089525573692)8:0.03515191084023677748)1:0.00014234145607245669)4:0.02547025825788062220)8:0.02959817825699541730)4:0.00000176456401954691)19:0.05616784528066534610)3:0.01847565408135587797,((((Toxotes_jaculatrix:0.40855226430946700589,Nematistius_pectoralis:0.23233914319104015389)33:0.05322116963234586884,(Xiphias_gladius:0.12669516329815763700,(Istiophorus_platypterus:0.00000176456401954691,(Istiophorus_albicans:0.00686848941253650081,(((Makaira_nigricans:0.00099275674325543790,Tetrapturus_audax:0.00698818249701090456)100:0.00395778780597797874,(Tetrapturus_pfluegeri:0.00392607736196458248,Tetrapturus_albidus:0.00393808920259764231)100:0.00095449735936051661)100:0.01704166006049621235,(Makaira_indica:0.01785324752632470868,(Tetrapturus_angustirostris:0.00747532438570297757,Tetrapturus_georgii:0.01978375658046737859)100:0.01553400140340865238)62:0.00186257769079902760)100:0.03401436939108348073)100:0.06329954240637908347)100:0.21266109967500460010)80:0.11345345129063272305)5:0.05383401706951057575,Lactarius_lactarius:0.26132630555258751226)2:0.01998388892488909066,((((Scomberoides_tol:0.00000176456401954691,((Paralichthys_dentatus:0.08131245965102550100,Scomberoides_commersonnianus:0.06584939315315471720)2:0.07063999568030417120,Scomberoides_lysan:0.00000176456401954691)1:0.00234112248710189067)4:0.13206307009897136351,Oligoplites_saurus:0.34707754574214022547)4:0.15936926056325734224,Parona_signata:0.32942613232547246493)0:0.03666351428889792763,(((Trachinotus_baillonii:0.06304133765913134957,Trachinotus_coppingeri:0.03220639276247030253)100:0.13036100443137202398,(((Trachinotus_blochii:0.02461515152141636326,(Trachinotus_anak:0.00000176456401954691,Trachinotus_ovatus:0.01389588926571542517)100:0.03497029990353375806)96:0.05062582857386826313,Trachinotus_paitensis:0.05510059251150307980)92:0.04713317345680172188,Trachinotus_goreensis:0.14888597276084811205)78:0.05925461670190477342)98:0.17352642680759045035,(Elagatis_bipinnulata:0.21281944140134975707,(Naucrates_ductor:0.11146789937946859073,((((Seriola_dumerili:0.05542406201217591616,Seriola_rivoliana:0.04798504878607677548)94:0.06420526840711586469,Seriola_hippos:0.04876378492218396560)35:0.02505687110531603159,Seriolina_nigrofasciata:0.23245654930557047013)20:0.00679963342881597949,(Seriola_lalandi:0.03658186440641846005,Seriola_quinqueradiata:0.05022782508651318922)46:0.02106518069695417078)36:0.05113108239238656205)93:0.12677714031314601950)52:0.08419056388220483300)25:0.02304865951922684120)2:0.02356367033689964890)0:0.02398520810443918391)0:0.03835686687199874045,(((Marleyella_bicolorata:0.12636304378138585314,(Poecilopsetta_hawaiiensis:0.03990229893649500925,Poecilopsetta_natalensis:0.01212016130847860113)99:0.13129064786338917603)91:0.18525726722858637530,Mene_maculata:0.36905973196452779295)15:0.06721082972983881410,((((Centropomus_undecimalis:0.55310731924145239979,(((Sphyraena_argentea:0.62883765135658853396,(Sphyraena_ensis:0.21467096120250278579,(Sphyraena_putnamae:0.00214529265951799494,Sphyraena_jello:0.00000176456401954691)100:0.23207872299025339435)63:0.00539158446629095164)20:0.09826313496637004152,Sphyraena_forsteri:0.19211818084706314136)44:0.07215871943408645739,(Sphyraena_obtusata:0.12399215915289707457,Sphyraena_pinguis:0.18714962675506896517)100:0.35609790985885175774)27:0.14032938690161106399)3:0.02340264425392655362,(((Echeneis_naucrates:0.04271930540207446308,Phtheirichthys_lineatus:0.03441004001824064795)100:0.18673216538003445386,(((Remorina_albescens:0.20479629563463785935,Remora_australis:0.20932475910550141229)11:0.00000176456401954691,(Remora_brachyptera:0.26087183469526548096,Remora_osteochir:0.10290079787169642556)40:0.05519210376637977145)12:0.02236930489048946299,Remora_remora:0.14033304563830037437)57:0.08720489817779633612)99:0.30809644769520283525,(((Eleutheronema_tetradactylum:0.33403412028000806222,Polydactylus_approximans:0.24672150720087118736)45:0.06119996981065511238,(Leptomelanosoma_indicum:0.28204384353448613831,(Filimanus_heptadactyla:0.13697246277936525605,Polydactylus_sextarius:0.12231263815963795694)100:0.21090028908832669785)24:0.01493993603812623916)59:0.10604433588326495075,Polydactylus_plebeius:0.24828650130922727546)96:0.18308417339626029130)42:0.09052004432304568138)4:0.00994498467336695120,(Leptobrama_muelleri:0.25553532041641269235,((((((Scophthalmus_rhombus:0.16962021640780716725,Scophthalmus_maximus:0.16909290933556236913)89:0.11302464142417985304,Scophthalmus_aquosus:0.36697312345720545235)56:0.21015972600578594620,Lepidorhombus_boscii:0.08858362050128669452)6:0.02930377275556392963,Lepidorhombus_whiffiagonis:0.08354424475845689813)8:0.07914137529484358002,Zeugopterus_punctatus:0.19766120416818175332)22:0.14925426742776493061,Phrynorhombus_norvegicus:0.33703377593133149182)94:0.29328140523930434913)16:0.04476661788142226317)0:0.01535302112683535267,((Lates_calcarifer:0.43975406971926939148,Lates_niloticus:0.16332546977612760752)76:0.21639593260996198287,Psammoperca_waigiensis:0.43227399643414221586)10:0.06626950833858187562)0:0.01985030687285493922)0:0.00880949968459867304)0:0.02025218922370879832)0:0.03188217579955150704)0:0.00991928143264853186,Parachanna_obscura:0.54066273743121529005)0:0.01101550129771986311,((((Heteropriacanthus_cruentatus:0.22444157023045771537,Pristigenys_serrula:0.12745723562369218329)77:0.18305910703718236698,((((Prionurus_scalprum:0.17982452254929917612,(((((Aplodactylus_etheridgii:0.07851937266752280487,(((Morone_chrysops:0.09806328041847994781,Dicentrarchus_labrax:0.22962614996986596694)27:0.06235846714853560818,Morone_saxatilis:0.03073007656466943821)32:0.02576565648388404184,Crinodus_lophodon:0.08650266795213476523)1:0.05608707002530931296)1:0.00000176456401954691,(Morone_mississippiensis:0.02616927534346483175,Morone_americana:0.04859600984872397555)93:0.08454101207241918048)2:0.03545344550513614135,(Aplodactylus_arctidens:0.05030714311585106346,(Aplodactylus_punctatus:0.03226927014319449366,Aplodactylus_westralis:0.07114797460324179257)17:0.00822920664597406350)22:0.02545872832377253300)7:0.18160430159272950013,Chironemus_marmoratus:0.22274154400908599549)3:0.06063246235717289351,(Naso_vlamingii:0.00684857058746027141,((Parapercis_maculata:0.00199747968897463811,Naso_annulatus:0.00250249430996922354)89:0.03301045174824582262,((Naso_elegans:0.01145891489466963759,Naso_lituratus:0.00887057702384184792)56:0.06350613299273946688,(Cheilodactylus_fasciatus:0.01986316033491261276,(Naso_unicornis:0.00000176456401954691,Cheilodactylus_pixi:0.03104458249812758544)0:0.00000176456401954691)4:0.06584999006072562999)3:0.06500934234149303992)1:0.01479114617975568757)5:0.21075479207760380862)0:0.00761757707319994821)0:0.02339542837056543881,(((((Centropyge_vrolikii:0.02899261369748383152,(Centropyge_flavissima:0.08477491201243822494,Centropyge_eibli:0.06032652931554970815)40:0.02134386722364631575)77:0.00000176456401954691,(Latris_lineata:0.08339948914755315745,(Latridopsis_forsteri:0.03614232693014269443,Latridopsis_ciliaris:0.05795785510524863876)69:0.04884392360861783411)63:0.02991683010825445244)4:0.03728917494422439444,(Centropyge_tibicen:0.10156067241389532541,((Centropyge_heraldi:0.06335228153332864154,Centropyge_nox:0.13893908156143244814)28:0.01758177539176876414,Centropyge_bicolor:0.12369239141226787138)92:0.07518150505708755260)91:0.07537128825863434467)4:0.08190101790192896858,((((Pygoplites_diacanthus:0.14531985830186622266,Cheilodactylus_quadricornis:0.03578124792661179637)1:0.03555856771678189698,(((Holacanthus_tricolor:0.12507332593289707812,Holacanthus_ciliaris:0.11652907023819754351)99:0.10046718301154013153,(Apolemichthys_trimaculatus:0.05754457157039647580,Apolemichthys_xanthurus:0.06461376894458163711)89:0.04533227274500191417)45:0.03831004862196537436,(Centropyge_multifasciata:0.06031929927703752470,Sumireyakko_venustus:0.09175420364031949827)96:0.09226395638582243763)13:0.00489838606010393769)0:0.00997167517391618456,((Genicanthus_watanabei:0.09535370046891120110,(Genicanthus_melanospilos:0.02523489453268579555,Genicanthus_lamarck:0.06773341061772396310)69:0.02173931263702872860)87:0.06307294824348437523,(((Cheilodactylus_vestitus:0.06842236409512145179,((Chaetodontoplus_mesoleucus:0.27423106992381612868,(Chaetodontoplus_melanosoma:0.08230741498338993434,(Chaetodontoplus_caeruleopunctatus:0.00822015163322155004,Chaetodontoplus_septentrionalis:0.01337693792467437200)91:0.00478557487411662014)100:0.22354446576452408069)87:0.18951887551778698326,((Pomacanthus_navarchus:0.07265664046889726280,((Pomacanthus_imperator:0.11262848981224214684,((Pomacanthus_semicirculatus:0.02561653973581293903,Pomacanthus_rhomboides:0.06551942013064805670)80:0.01752441415191367119,Pomacanthus_maculosus:0.03974184055569567053)100:0.09613474572273195129)47:0.03299239690157231703,(Pomacanthus_annularis:0.06425072594245090363,(Pomacanthus_zonipectus:0.03493592732290604769,Pomacanthus_paru:0.06068231576811024958)100:0.13736889680806660397)24:0.01351229901834880703)44:0.05032705990544675961)100:0.15903977015366996262,Cheilodactylus_gibbosus:0.01046112220676938091)0:0.00000176456401954691)0:0.03350002117660252909)0:0.02530191568440177605,(Cheilodactylus_zebra:0.03276213746751563405,(Cheilodactylus_plessisi:0.04450864783872861968,Cheilodactylus_vittatus:0.06461436920931415417)26:0.00739723538240021593)69:0.05702105667350452350)0:0.04854486678763772489,(Mendosoma_lineatum:0.07656262345994427343,((Dactylophora_nigricans:0.08736154035170876075,(Cheilodactylus_fuscus:0.06444128833841089588,Cheilodactylus_ephippium:0.05712365235522504775)74:0.04183723865479602178)20:0.01954515759520172521,(((Cheilodactylus_spectabilis:0.05143850269298444466,Chirodactylus_variegatus:0.01427473647061052939)41:0.01890971779638124517,(((Nemadactylus_douglasii:0.03978339309005827357,Nemadactylus_valenciennesi:0.00754215193551742175)80:0.03654855631065855037,Nemadactylus_macropterus:0.03263579501777706671)75:0.07606989652483878150,(Chirodactylus_brachydactylus:0.01805005619209962275,Chirodactylus_jessicalenorum:0.03513368558361831123)55:0.01166763834850193318)29:0.01707623250374711021)5:0.00647721250005936500,Cheilodactylus_nigripes:0.03459754759990882333)8:0.01047392611049343530)2:0.00018560791428513774)3:0.03179978452664410093)0:0.00000176456401954691)0:0.01957810917192010988)1:0.00741162488046665565,((Centropyge_acanthops:0.00676236672305173536,Centropyge_argi:0.00000176456401954691)99:0.17580402892328386710,((Cheilodactylus_rubrolabiatus:0.00000176456401954691,Centropyge_potteri:0.02216451783281887106)4:0.02924663216164847743,((Centropyge_multicolor:0.01473153924365956310,(Centropyge_ferrugata:0.02050796424205146573,Centropyge_bispinosa:0.02555648922865777495)100:0.04832697501338669571)95:0.02377168828682208607,Centropyge_loricula:0.08136623207734686170)92:0.03503791317941996192)5:0.02909311005215951818)6:0.09022266494764412392)1:0.04073282488401661072)7:0.11842631322592893361,(Zanclus_cornutus:0.37642860551796231139,(((Paracanthurus_hepatus:0.19075777087048012559,Zebrasoma_veliferum:0.11035802520186302478)71:0.03257163923706865583,(Zebrasoma_rostratum:0.01611498014301844528,(Zebrasoma_flavescens:0.00000176456401954691,Zebrasoma_scopas:0.00442955810868536550)99:0.00994823953208018481)98:0.14488848382489324940)97:0.14023211664137688603,(Acanthurus_thompsoni:0.09518985653363584709,(((((Acanthurus_achilles:0.04083293471169417710,((Bodianus_loxozonus:0.12409724908944622102,Acanthurus_leucosternon:0.00000176456401954691)91:0.01534976534767207995,(Acanthurus_japonicus:0.00950558207159647728,Acanthurus_nigricans:0.00451898544281348520)41:0.00197017005924613950)47:0.00672207531906773067)97:0.11668665178795781245,(Acanthurus_coeruleus:0.11009018252616617484,Acanthurus_guttatus:0.04026208020643466667)15:0.00607207241788443233)48:0.03784794860155406282,((Acanthurus_nigricauda:0.07998678336292111557,((Acanthurus_dussumieri:0.08073095017353053293,(Acanthurus_mata:0.05203804451250500923,Acanthurus_xanthopterus:0.05446436142128749380)12:0.00000176456401954691)27:0.01138105459969890147,Acanthurus_blochii:0.04776886913349532443)86:0.03448985376852155282)64:0.02227189304012829052,(Acanthurus_olivaceus:0.03821406876382373030,Acanthurus_tennentii:0.02778814210512526098)94:0.06548248382765610442)29:0.01445089752210432059)6:0.01155362788574917227,(((Acanthurus_bahianus:0.04309817989389552045,Acanthurus_chirurgus:0.04447795046483064146)92:0.04116811645845359913,Acanthurus_lineatus:0.15021562961689419180)9:0.01824825868742244173,(Acanthurus_triostegus:0.16275349717899162405,((Acanthurus_nubilus:0.06420141211867325115,(Ctenochaetus_striatus:0.03309156310964288944,(Ctenochaetus_flavicauda:0.00172032016964076282,Ctenochaetus_strigosus:0.01203921627276573030)96:0.02189118863728471698)93:0.03488561959544972391)86:0.04549907306771505677,Acanthurus_pyroferus:0.07992513251630027626)54:0.01111319929206047025)26:0.03738931562040585799)1:0.01056860541755241974)5:0.00387052641181232990,Acanthurus_nigrofuscus:0.16878167242724392838)33:0.03311451411351390428)91:0.17783046071829164414)15:0.03144318980770988842)0:0.03249385433502538501)0:0.00820983871355098795)0:0.04545548900498912193,((Prognathodes_falcifer:0.27534963266761736111,(((((Chaetodon_ocellicaudus:0.16574081908266621865,(Chaetodon_rafflesii:0.04355150753405426928,(Chaetodon_ulietensis:0.05002135718142734083,(((Chaetodon_auriga:0.01577920718490144134,Chaetodon_vagabundus:0.02119770156316473295)45:0.00686993018829053486,Chaetodon_decussatus:0.02847475282096163102)66:0.02062859284048010333,(Chaetodon_collare:0.02706494547410095639,((Chaetodon_lunula:0.00000176456401954691,Chaetodon_flavirostris:0.05890294585816861772)100:0.04421192185511639561,(Chaetodon_wiebeli:0.02384742829426332242,Chaetodon_auripes:0.02092547265465713457)44:0.00755437116447655287)27:0.00370219446628747501)42:0.01497988061627823428)12:0.00765675882081845623)6:0.00803232077103238976)63:0.04482376627308750500)56:0.00000176456401954691,(Chaetodon_semeion:0.08153069865968397278,Chaetodon_ephippium:0.04949821934142575180)98:0.05957755313664626212)79:0.05146528947908474844,((Chaetodon_capistratus:0.03438346603900827719,Chaetodon_striatus:0.05974528463946764817)85:0.03205136087204969036,Chaetodon_ocellatus:0.10304872124857664129)100:0.12574273335307345478)30:0.01129591632363706813,(Chaetodon_speculum:0.11507393950299463214,((Chaetodon_kleinii:0.10999109174583684978,Chaetodon_unimaculatus:0.09431110994729502428)95:0.12291462154724938638,((Chaetodon_argentatus:0.06426866685519530553,(Chaetodon_xanthurus:0.00524767809860333251,Chaetodon_madagaskariensis:0.01961346955477862725)93:0.05651835772497663252)92:0.04033699567240247447,(Chaetodon_quadrimaculatus:0.06389873409281235372,(Chaetodon_punctatofasciatus:0.00987110866483534993,Chaetodon_multicinctus:0.00087424468641330070)100:0.08061035704989517880)95:0.05091667922718485367)97:0.04793499688003192982)27:0.04552332902312713342)14:0.02857588395086865449)3:0.00606189330224217449,((Chaetodon_meyeri:0.06943456412248982801,Chaetodon_ornatissimus:0.04053927347375600038)100:0.14802556285581064399,(Chaetodon_lunulatus:0.02056332914302309509,Chaetodon_trifasciatus:0.05031327653052940219)100:0.10419023614219499740)73:0.05345123010579126005)71:0.06111789221312126374)72:0.09255746984717637404,(Chelmon_rostratus:0.38243166666083916461,(Forcipiger_flavissimus:0.22599214784787108323,(Hemitaurichthys_polylepis:0.20726166419927480322,((((Heniochus_acuminatus:0.02348701585608383227,Heniochus_singularius:0.06269651142950954936)98:0.07636370118185910327,Heniochus_diphreutes:0.08134938572437970528)60:0.03137225687037378113,Heniochus_chrysostomus:0.11535209705371983480)23:0.01902267134069295096,Heniochus_varius:0.14025803169340164689)85:0.08549643706700078838)82:0.05359283942221325370)82:0.08869118604752838919)56:0.07560722887312079643)51:0.10657547062502480129)0:0.03759546008522365867,((Monodactylus_argenteus:0.23458254991876217876,(((Drepane_punctata:0.31009983048426065233,(Chaetodipterus_faber:0.12543897519849794842,(Ephippus_orbis:0.41108549729963561603,((Platax_teira:0.02097015897200911125,Platax_orbicularis:0.02325754176960631928)89:0.09901021123690724457,(Platax_pinnatus:0.06151694359875935214,Platax_batavianus:0.06320504297923933146)90:0.08797240074685415412)66:0.00711349096272018231)68:0.10025592152249220013)59:0.06664060074236682896)10:0.05724374039449982993,(Parapristipoma_trilineatum:0.18216314369008498875,((Plectorhinchus_schotaf:0.13958559586824351162,Plectorhinchus_flavomaculatus:0.06726567977443076440)97:0.09933489980453383272,(Lactoria_cornuta:0.19172050726629832895,((Plectorhinchus_gibbosus:0.13098043623184343898,Plectorhinchus_cinctus:0.07982959968642783233)90:0.11284612652048991999,(Plectorhinchus_picus:0.08292340434306029462,(((Diagramma_picta:0.00000176456401954691,Plectorhinchus_orientalis:0.00640248725521901530)96:0.11246115606863950342,Plectorhinchus_chaetodonoides:0.05268493817572352661)81:0.04170141977312154247,Plectorhinchus_gaterinus:0.15108574530556179161)45:0.03755036050564902750)58:0.03290691076124679232)63:0.05186003674197457725)51:0.03213009660884148105)69:0.08141544464968339390)61:0.08138945765652891295)1:0.01540385142624688293,((Symphorichthys_spilurus:0.25195009380656713249,(((Lutjanus_lutjanus:0.00923127940725621508,(Lutjanus_erythropterus:0.06946192324252621619,Lutjanus_malabaricus:0.07134209055955005596)81:0.00354839898023615085)99:0.09541818494323592303,(Lutjanus_sebae:0.14353200225620790786,Lutjanus_adetii:0.07652126255103446073)93:0.02201996598123303825)96:0.07619937087019973410,(((Macolor_niger:0.09560787550095325993,((Caesio_xanthonota:0.05599131680637964986,((Pterocaesio_digramma:0.01397049121551578038,((Pterocaesio_marri:0.00238774920470150335,Pterocaesio_chrysozona:0.00216310413280222527)97:0.03624203429844802954,Pterocaesio_trilineata:0.01836640299559937767)41:0.01460232102224798094)75:0.02479194896230279763,Gymnocaesio_gymnoptera:0.07306193284287688938)74:0.04394725998095204422)86:0.05886745346625518044,Caesio_caerulaurea:0.10425936074749024562)53:0.03425930107983968936)47:0.02402018225281440586,Lutjanus_gibbus:0.21406552536286443944)40:0.02443822125521094915,(((((Lutjanus_ophuysenii:0.05650150074328619265,Lutjanus_vitta:0.09031383334920831119)99:0.04649281489185817795,Lutjanus_lineatus:0.05781749878889440308)93:0.03369482540193213804,(Lutjanus_lemniscatus:0.00425422553769520036,(Johnius_belangerii:0.91941143219627907701,Lutjanus_fulvus:0.00000176456401954691)0:0.00281656441433198292)1:0.08005608318352819275)1:0.03867267056799363056,((Lutjanus_fulviflamma:0.03371248017692037718,Lutjanus_johnii:0.06494808507064948888)77:0.03832800200987995787,(Lutjanus_carponotatus:0.04125822707734070477,((Lutjanus_monostigma:0.00000176456401954691,Lutjanus_russellii:0.03137126740038215927)99:0.06172153231223679454,Lutjanus_ehrenbergii:0.05543167597495074483)24:0.00854736913551581295)48:0.01938206723445079493)22:0.00779813191952897101)0:0.04163086706675408338,(((Lutjanus_griseus:0.01488676397350625728,(Lutjanus_apodus:0.03197627192134046281,(Lutjanus_argentiventris:0.00765528104372078817,(Lutjanus_jocu:0.00234401294882128882,Lutjanus_bohar:0.00702683597579987010)67:0.00169700606721318254)76:0.01164173493073290475)47:0.01540308845227682244)98:0.11603086872979448407,((Lutjanus_notatus:0.03501421937793343375,(Lutjanus_quinquelineatus:0.05202763752387856711,(Lutjanus_bengalensis:0.05953841131439887441,Lutjanus_kasmira:0.02912078231852884697)48:0.01036999412143037660)83:0.03066597084806102491)98:0.11499395266139508798,((Lutjanus_argentimaculatus:0.04774251814247350983,((Lutjanus_novemfasciatus:0.02587001790931633960,Lutjanus_cyanopterus:0.01606431918013783919)99:0.06258124194089301207,Lutjanus_colorado:0.07337631139135671055)91:0.02494790142883211367)98:0.05565212729889164323,(Lutjanus_rivulatus:0.14892073210533710026,Lutjanus_stellatus:0.05353442383599148202)34:0.01781310475837869639)35:0.02446276512173972337)28:0.01731404857381810616)12:0.00825651947103875350,(Rhomboplites_aurorubens:0.09870307426332468326,(((Ocyurus_chrysurus:0.06286856088342102078,(Lutjanus_buccanella:0.04473380430666125396,Lutjanus_analis:0.01984599932842341999)36:0.00804463242809391570)6:0.00000176456401954691,(Lutjanus_mahogoni:0.04096580742636356937,Lutjanus_synagris:0.03721444825171680432)53:0.00993701125397109149)29:0.01373429453453384880,(Lutjanus_vivanus:0.02979296693482096081,(Lutjanus_peru:0.00799845187035279001,(Lutjanus_purpureus:0.00215935965567325572,Lutjanus_campechanus:0.00000176456401954691)100:0.01507454026331560108)99:0.01310699083236412966)98:0.04552425470042984618)16:0.00339475027002384896)92:0.06500878378836934257)11:0.02410662959578242781)1:0.00909219022374833930)0:0.01653351609804500078)4:0.04265747175443853484)2:0.05037415373734111662,(Paracaesio_sordida:0.21382632386696598381,((Etelis_oculatus:0.09167738849803912238,Etelis_carbunculus:0.03621588229709175877)93:0.07103522888299539095,(Pristipomoides_aquilonaris:0.14476795138779746663,(Pristipomoides_sieboldii:0.10658456526869562742,((Pristipomoides_filamentosus:0.08547930517545587747,Pristipomoides_typus:0.10754586016421366357)97:0.07593997222075908604,Aphareus_rutilans:0.09046823409345788880)89:0.05331652332086108531)53:0.01386461017900309824)87:0.05303639788942381139)77:0.07057847297279783161)11:0.02406795843056882753)4:0.02383339477165309292)0:0.00180510419950675820)0:0.01378432619001616899,(Plagiogeneion_macrolepis:0.06153983945312431481,Emmelichthys_nitidus:0.07424548148144391457)93:0.12772689469472034429)0:0.02891990842547364701)0:0.01175786043315658891)0:0.00829330893837927630,(((((Anoplopoma_fimbria:0.33814948521647403279,(((((Pomadasys_maculatus:0.20785948722991190452,Pomadasys_hasta:0.15773004142484992052)65:0.08247614868246087860,((Anisotremus_dovii:0.22367192586479978100,(Orthopristis_chrysoptera:0.21344078084407747120,Conodon_nobilis:0.12828366844105440481)38:0.02183613304166403396)39:0.03446676796798391756,((Haemulon_chrysargyreum:0.16900863048279002321,(Xenistius_californiensis:0.09039065734606589742,(((Haemulon_maculicauda:0.00000176456401954691,Haemulon_flaviguttatum:0.00216643496821769012)100:0.04974101462355739328,(Haemulon_steindachneri:0.07402894772435776594,Haemulon_boschmae:0.11107738761275726125)55:0.01419608808037947992)83:0.02363882264456987695,((Haemulon_aurolineatum:0.09211666740900749384,(Haemulon_plumierii:0.05783903910577187224,Haemulon_sciurus:0.07124550461288685355)90:0.05227328958928501024)21:0.00533734192774542194,(((Inermia_vittata:0.07425870689609113950,Haemulon_striatum:0.03326894588674171593)100:0.08271652656566741424,(((Haemulon_squamipinna:0.07679093336093467670,(Haemulon_bonariense:0.00211109655086543207,(Haemulon_macrostomum:0.00000176456401954691,Haemulon_parra:0.00000176456401954691)95:0.00425823682832387595)100:0.05879177880391969063)95:0.04884851074874069915,(Haemulon_sexfasciatum:0.04784422908376299854,Haemulon_scudderii:0.05716530272934412099)98:0.04474681659040190274)8:0.00000176456401954691,(Haemulon_melanurum:0.06789142258708275035,Haemulon_album:0.05457951833350790233)83:0.03355531370276558512)14:0.00320973655339401890)17:0.00764733621935806754,(Haemulon_flavolineatum:0.05099502016867632492,Haemulon_carbonarium:0.07005457994920165354)85:0.05865409487728970306)7:0.00489879009505834670)32:0.01746521124455369112)49:0.03695118341444318666)24:0.01952967275315545953)89:0.13743386445077213076,(Hemichromis_bimaculatus:0.48187244039747229030,(Pomadasys_stridens:0.29058868507773549794,(((Anisotremus_surinamensis:0.00972559781549916028,Anisotremus_interruptus:0.01861163724140265860)97:0.05830130009834159910,Anisotremus_davidsonii:0.06767328529183365415)80:0.05733128453957784393,(Anisotremus_virginicus:0.01606258090921182380,Anisotremus_taeniatus:0.01189060353925567790)88:0.04744213816681307899)54:0.02748575727625633680)44:0.03931860457132095588)23:0.05641492349483546997)3:0.02206936384705308202)20:0.01104395446069360298)41:0.06408756837661626349,(((Sillago_aeolus:0.05368563758625108373,Sillago_maculata:0.07178090917954428607)97:0.09498398419683018457,(((Sillago_bassensis:0.07933121236258280917,Sillago_flindersi:0.05532709686435665491)98:0.12193629041053596906,(Sillago_robusta:0.23713528602166458747,(Sillago_japonica:0.40780069191979484433,(Sillago_ingenuua:0.21876054243901485452,Sillaginodes_punctatus:0.18045792528404516752)38:0.01889745311721632848)2:0.00000176456401954691)25:0.04161469460130035047)46:0.11862451791157949121,(Sillago_ciliata:0.00208427344923379186,Sillago_analis:0.00000176456401954691)100:0.09058857709521088430)10:0.05079350787916187687)6:0.00244974797825670302,Sillago_sihama:0.34569291129815937769)87:0.16025098542800803036)10:0.03476755442455427281,((Acantharchus_pomotis:0.22006148698572783329,(((((Lepomis_gibbosus:0.12619739084982545263,(Lepomis_megalotis:0.00930993162837648672,Lepomis_marginatus:0.02411249296081665813)99:0.16769018324546708376)59:0.04864092052652917164,((Lepomis_miniatus:0.02395831614162054896,(Lepomis_punctatus:0.02774578407797427190,Lepomis_microlophus:0.04167059996994133125)18:0.00094317864334285450)95:0.03274766868469387165,Lepomis_auritus:0.07649262597400004193)80:0.03473247767826091043)87:0.08227297694818593021,((Lepomis_humilis:0.06409599665914957056,Lepomis_macrochirus:0.05602404770024038694)95:0.09052746198948212475,((Notocirrhitus_splendens:0.11456942416164195286,Chaenobryttus_gulosus:0.03669316112094692106)7:0.07146550404881774066,(Lepomis_symmetricus:0.00926974608581669114,Lepomis_cyanellus:0.00209438618703682586)97:0.06817994892524036066)10:0.10697394060581624786)7:0.02080087261429802367)16:0.08537916009815414708,((Milyeringa_brooksi:0.60045787660898053417,(Enneacanthus_chaetodon:0.08631454918114432606,(Enneacanthus_gloriosus:0.01543010197348916417,Enneacanthus_obesus:0.01560838161344863494)84:0.08301171789344569119)77:0.04024810759786066894)11:0.10036014409374974243,(((((Ambloplites_ariommus:0.03102080968649977705,Ambloplites_rupestris:0.02452008311944271768)99:0.07534814038406020820,(Ambloplites_cavifrons:0.06979286179944164947,Ambloplites_constellatus:0.05077592383381412250)57:0.01783343482845734657)95:0.06050350576464177954,(Pomoxis_nigromaculatus:0.16342103847288186325,Pomoxis_annularis:0.11233526376547831038)49:0.05079093372132126305)6:0.00000176456401954691,Archoplites_interruptus:0.14718706469683856541)26:0.02137579667331885799,Centrarchus_macropterus:0.21303820353735500315)27:0.01279116934792565163)6:0.03636853952488508512)0:0.04027278550246555372,((Micropterus_dolomieu:0.00224890241267281573,Micropterus_punctulatus:0.00210424080160384608)100:0.07141670661231308559,(Micropterus_coosae:0.04765563786108357319,(Micropterus_cataractae:0.05604891366244209305,(Micropterus_notius:0.06932105002493327284,(Micropterus_floridanus:0.04057169726277808780,(Micropterus_salmoides:0.00000176456401954691,Micropterus_salmoides_salmoides:0.00000176456401954691)100:0.02539647849255271400)87:0.02191144444668828573)54:0.00961842814505235906)40:0.01929018583542878654)29:0.00433965376283380021)99:0.16853820097575505921)0:0.02581995622140584659)1:0.07700478684279689345,Percophis_brasiliensis:0.33434119672428008752)0:0.02777118174929509020)0:0.01465709340124297412,((((Variola_albimarginata:0.03800496304824949045,Variola_louti:0.10900445347948969910)99:0.39014019492744633766,(((((((((Epinephelus_ongus:0.12454294389954538580,Cromileptes_altivelis:0.13495255354492613487)42:0.01275158162685536070,Epinephelus_moara:0.05413312281247047164)48:0.02728489687199889269,Epinephelus_latifasciatus:0.10007646600023002714)35:0.02115453437249709828,((Epinephelus_malabaricus:0.04111916203630276018,(Epinephelus_coioides:0.00458680371681479803,Epinephelus_tauvina:0.00000176456401954691)100:0.02077535749044085236)97:0.07909303438268097675,(Epinephelus_itajara:0.03843166061626236002,Epinephelus_lanceolatus:0.10037874547378090873)97:0.07990851615647096007)45:0.02183685042113729310)23:0.00000176456401954691,Epinephelus_fuscoguttatus:0.09402181908920735631)33:0.02071309361575982000,Anyperodon_leucogrammicus:0.14742511434602528775)78:0.02343953103843642721,((Epinephelus_albomarginatus:0.09360387166443623341,(((((Mycteroperca_interstitialis:0.02630986607186496656,Mycteroperca_xenarcha:0.04856048862262437454)98:0.02933240846083580780,(Mycteroperca_bonaci:0.03518587735029623381,Mycteroperca_jordani:0.03619000616629378803)69:0.01968335225924992132)78:0.02845236845763729830,Mycteroperca_microlepis:0.03084439345992549472)100:0.08086897262397937935,(Epinephelus_morrhua:0.03247488577711731689,(Epinephelus_poecilonotus:0.10470188048527166724,Epinephelus_epistictus:0.05559937039300800327)85:0.04099257436030343676)61:0.01367120478584447907)21:0.01279036190809768742,(Mycteroperca_marginata:0.00000176456401954691,Epinephelus_marginatus:0.00000176456401954691)100:0.07695637704853015848)21:0.01448333900188081627)67:0.03018955215771380873,((Triso_dermopterus:0.19279800425287385579,(Dermatolepis_dermatolepis:0.02251676549087960888,Dermatolepis_inermis:0.05036008132506307466)100:0.16306339872302158533)11:0.00199520037127209936,((Epinephelus_septemfasciatus:0.03030608141677191419,Epinephelus_ergastularius:0.00525117226297662932)100:0.04541465540653494282,Epinephelus_acanthistius:0.03982774377622177314)75:0.02575064071200965782)48:0.04097284793340412457)19:0.01332886726032815432)20:0.01865903949690901967,((((Epinephelus_quoyanus:0.07160880889689147377,(Epinephelus_fario:0.04685821854012094806,Epinephelus_macrospilos:0.04433343899953875317)59:0.02190193184613366956)38:0.04674792406279185131,Epinephelus_rivulatus:0.13844630587164505342)93:0.08209979250817149576,(Epinephelus_morio:0.04851667651724967528,Epinephelus_guttatus:0.12851050404039060515)100:0.09313230161011876174)65:0.06050839288226819712,((Epinephelus_adscensionis:0.20153938526656686725,((Epinephelus_merra:0.20295772968109820900,Epinephelus_fasciatus:0.12611449074236749657)48:0.05321777012829809195,(((Epinephelus_bleekeri:0.00000176456401954691,Epinephelus_akaara:0.00230295473333448283)100:0.07118805945428274828,(Epinephelus_maculatus:0.07715070734635399996,Epinephelus_longispinis:0.06821440306710292723)90:0.05947888452944666976)58:0.02283813668226008370,(Epinephelus_undulosus:0.09877467320430657893,((Epinephelus_spilotoceps:0.07898013945680772141,(Epinephelus_areolatus:0.08463602904944481087,Epinephelus_multinotatus:0.05882922370638663662)36:0.00000176456401954691)68:0.00983805023063863762,Epinephelus_chlorostigma:0.05513732591672730610)80:0.01751371655049531462)100:0.06605333067393064084)58:0.03414797922328777213)7:0.01027409311122902229)13:0.03008738868402647051,(Epinephelus_amblycephalus:0.09667991188822686044,((Epinephelus_fasciatomaculosus:0.04390060922741992333,Epinephelus_sexfasciatus:0.04783884201734815017)84:0.03250221750396013742,Epinephelus_diacanthus:0.20116128345696090984)26:0.01959407601764606016)79:0.04661830760264319001)12:0.00851335488687238705)76:0.04330296838893519379)69:0.06476647679132518931,(((((Cephalopholis_miniata:0.03888225238421136321,Cephalopholis_sexmaculata:0.02916429640954032107)99:0.05379420766359779144,((Cynoglossus_acaudatus:0.01397167640952641510,(Cephalopholis_sonnerati:0.00467177754452283156,Cephalopholis_argus:0.01832560238325825833)88:0.00577930163901562918)98:0.03260294775709692006,Cephalopholis_urodeta:0.06553655409802171261)57:0.02100716841136514301)79:0.03205063312690409338,(Paranthias_colonus:0.09782395555393456898,Cephalopholis_fulva:0.09911034927253638271)54:0.04199615939039555434)40:0.02079885453427204878,(Cephalopholis_hemistiktos:0.06900881508521926333,Cephalopholis_leopardus:0.06515915371010305712)79:0.02006333889733457124)97:0.13176267936609287190,((Cephalopholis_formosa:0.06059442306308856357,((Cephalopholis_boenak:0.00897611921860579608,Cephalopholis_igarashiensis:0.02225392077529176130)100:0.11003706798649776077,(Cephalopholis_cyanostigma:0.00297031000607516889,Cephalopholis_microprion:0.00843916935682332073)95:0.03998696840024561910)71:0.01828547809273799521)100:0.14749282472887320639,(Cephalopholis_cruentata:0.24775654863742077594,Aethaloperca_rogaa:0.09165669184658727098)52:0.05016186482927089546)57:0.05512068548322709982)61:0.04933792275217075557)53:0.08131071984529149310)29:0.07100882654476051159,((((((((Chrysoblephus_cristiceps:0.00398203812341102210,(Chrysoblephus_puniceus:0.06044389254873079598,Chrysoblephus_laticeps:0.03189043781846377656)81:0.00853007582158834751)95:0.03745131875040766389,Petrus_rupestris:0.05659426413547160445)21:0.00000176456401954691,(Chrysoblephus_anglicus:0.12699468566847460504,Chrysoblephus_lophus:0.07069294871374404154)49:0.02694503443862388339)54:0.03233307255737599695,Argyrozona_argyrozona:0.17506381699429207632)64:0.04374763388779793344,(Spicara_australis:0.08679148345412752985,Pterogymnus_laniarius:0.10007479330136627438)33:0.02241328406191418071)42:0.03826542909931836267,(Pagellus_erythrinus:0.10456411111220224874,((Argyrops_filamentosus:0.10062554707942018839,(Cheimerius_nufar:0.18510624630510857935,Argyrops_spinifer:0.07845639020852746004)45:0.03724486098036294823)17:0.02737872112219853424,(Chrysophrys_auratus:0.04842926438589217203,((Evynnis_japonica:0.00225226680058925478,Parargyrops_edita:0.02071243580676779272)72:0.00000176456401954691,Evynnis_cardinalis:0.00632234593830276019)99:0.08190249058669943349)79:0.02838904087282751593)6:0.00000176456401954691)45:0.02616960581519567342)88:0.09935305563727847566,((((Pachymetopon_blochii:0.09285835527385948029,Lithognathus_lithognathus:0.11960512757314041565)95:0.09931713950952524017,(Boops_boops:0.22658789483998614811,(((Acanthopagrus_butcheri:0.03712152235107726850,Acanthopagrus_australis:0.04116706717265618959)94:0.03718846898543581858,(Acanthopagrus_latus:0.08779714736823619747,(Acanthopagrus_schlegelii_schlegelii:0.00000176456401954691,Acanthopagrus_schlegelii:0.00000176456401954691)100:0.06339035361251661727)76:0.02268598848752697780)27:0.00000176456401954691,Acanthopagrus_bifasciatus:0.11739148633168479396)91:0.09017494127912664692)34:0.00397215604042961117)26:0.01425924031770707187,((Sparus_aurata:0.12480350370692527839,(Rhabdosargus_globiceps:0.07982453256282560916,(Rhabdosargus_sarba:0.01074078792232660041,Acanthopagrus_berda:0.03235832731698187054)100:0.09481368586549554500)78:0.04655288138188740099)62:0.04961770543566480091,(Pagellus_acarne:0.18080342670174193165,(Diplodus_vulgaris:0.04753464730778222957,((Diplodus_sargus_kotschyi:0.00153806089235047146,Diplodus_sargus:0.01227291133353684317)98:0.03766057704637853359,Diplodus_cervinus:0.06876824617684228202)94:0.05828148189689421471)69:0.04152058810554107304)11:0.00985670470884879302)7:0.01329067780466937874)15:0.02167808960372721166,((((Enneanectes_boehlkei:0.00000176456401954691,Axoclinus_multicinctus:0.56749419896351926962)86:0.50553899043027583637,Calamus_brachysomus:0.21005575145392998970)1:0.03087039593511167399,Calamus_penna:0.09196033772738329559)0:0.02348750329916677107,((Archosargus_probatocephalus:0.05076075368949502231,Archosargus_rhomboidalis:0.13683304308377036707)86:0.05841879797551483705,Lagodon_rhomboides:0.07509807471481289021)57:0.02434334661002682254)1:0.04223681605663134192)0:0.06172933218366025848)3:0.17123209117240797972,Taenianotus_triacanthus:0.45957518512143641365)0:0.04364799374783565750)0:0.06993466799588843319,(((Congiopodus_peruvianus:0.32684494948104608758,(Neosebastes_thetidis:0.13536543595704234044,(Neosebastes_incisipinnis:0.06796482778325431406,Neosebastes_scorpaenoides:0.10977330631873249978)50:0.03848877579582767050)90:0.10971118443211751170)3:0.01617322325731867377,(((Scorpaenodes_xyris:0.28999153857108195265,Scorpaenodes_guamensis:0.21781932434179498981)39:0.10971298454052294535,((Sebastolobus_macrochir:0.00757340292081505172,(Sebastolobus_altivelis:0.01348793075225210650,Sebastolobus_alascanus:0.00450034957349929477)91:0.01061397959852275301)98:0.14496439167786998037,((Pontinus_macrocephalus:0.34280713575820348504,((Helicolenus_avius:0.00431827075809017129,Helicolenus_percoides:0.00707494416609386056)86:0.00489126102014060538,(Helicolenus_barathri:0.00221990835827218453,(Helicolenus_dactylopterus_dactylopterus:0.00936966864204660278,Helicolenus_dactylopterus:0.00535798587179981984)54:0.00268099949875595352)57:0.00551805809931928560)70:0.01412144814290748271)32:0.04474577020641027497,(Sebastiscus_marmoratus:0.15333816357105842232,((Sebastes_viviparus:0.01993577047366957775,(Sebastes_fasciatus:0.00000176456401954691,(Sebastes_norvegicus:0.00128213417663623893,Sebastes_mentella:0.00000176456401954691)73:0.00127864108240016423)90:0.00000176456401954691)94:0.01568639138381600420,((Sebastes_baramenuke:0.01005403385924663780,(Sebastes_alutus:0.01094926798185325879,(Sebastes_polyspinis:0.00519521822373191292,(Sebastes_ciliatus:0.00000176456401954691,(Sebastes_variabilis:0.00000176456401954691,Sebastes_crameri:0.00392619132107158673)69:0.00129022771202507098)52:0.00000176456401954691)100:0.01049804066638499074)51:0.00272774904072753746)35:0.00054697990865784770,(((Sebastes_brevispinis:0.00912121868784020705,((Sebastes_borealis:0.00256937864951281006,(Sebastes_flammeus:0.00384508573664739698,Sebastes_iracundus:0.00256109822107738723)15:0.00000176456401954691)66:0.00127897934657813378,(Sebastes_matsubarae:0.00897323876351606604,(Sebastes_proriger:0.01275757467234838079,(Sebastes_emphaeus:0.00307648081232156541,(Sebastes_variegatus:0.00128782935867051136,(Sebastes_wilsoni:0.00524104049742066865,Sebastes_zacentrus:0.00132240301245399052)73:0.00125581851874676030)85:0.00208855492891125202)100:0.00697909749118216620)81:0.00433437193572339726)39:0.00278190117175618678)5:0.00000176456401954691)44:0.00333739336834458673,((Sebastes_glaucus:0.01661549091233846184,((Sebastes_owstoni:0.04701337110467251196,(Sebastes_minor:0.01897547929096168526,Sebastes_steindachneri:0.00679869199807887643)81:0.00751521781973473694)26:0.00477066865827324108,(Sebastes_aleutianus:0.00253423537097194817,Sebastes_melanostictus:0.00384901418643410166)99:0.00660097822590303453)44:0.00532796655341536210)29:0.00621423529645187198,((((Sebastes_levis:0.02539640663090398515,(Sebastes_gilli:0.01465864211212798349,(Sebastes_phillipsi:0.00257538997105753254,Sebastes_aurora:0.00388751111933388887)95:0.00375212716044165742)63:0.00940968991556796058)28:0.00332322372735944019,(((Sebastes_ovalis:0.00427144685518569562,Sebastes_hopkinsi:0.00770104766208352682)99:0.01762268528528353195,((Sebastes_rufus:0.00924494644582578122,(((((Sebastes_notius:0.00776900338551419697,(Sebastes_spinorbis:0.00256812103430602637,(Sebastes_constellatus:0.00000176456401954691,(Sebastes_capensis:0.00128209853372380708,Sebastes_oculatus:0.00127852486130895196)64:0.00127264596351963212)97:0.00514669280029920670)16:0.00000176456401954691)54:0.00000176456401954691,(Sebastes_exsul:0.00522172024366207924,(Sebastes_umbrosus:0.00521393709262337780,Sebastes_lentiginosus:0.00000176456401954691)83:0.00131074017182784808)66:0.00126760538711536558)95:0.00524963027136008276,Sebastes_rosaceus:0.01315654863830979793)99:0.00397126927663723692,(((Sebastes_ensifer:0.00133937194678840138,(Sebastes_chlorostictus:0.00000176456401954691,Sebastes_eos:0.00260513881445293511)99:0.00661868636520271305)68:0.00125440302553887989,Sebastes_simulator:0.00000176456401954691)51:0.00000176456401954691,Sebastes_helvomaculatus:0.00389016497764002470)91:0.00257136027998609677)93:0.00650141930476680688,(Sebastes_babcocki:0.00530722949032846284,((Sebastes_serriceps:0.01680450730030674800,Sebastes_rubrivinctus:0.01071653653405774988)36:0.00223036149646503460,Sebastes_nigrocinctus:0.00901895830158323288)42:0.00152837215869428781)64:0.00130668918468213871)92:0.00937496869460159761)50:0.00273348652255113326,(Sebastes_rufinanus:0.01224207897896450493,Sebastes_moseri:0.01389873893817727447)59:0.00374480482362292335)22:0.00133301942722236053)10:0.00126067529758586757,(((Sebastes_paucispinis:0.06484866004433834097,Sebastes_jordani:0.05126147586678089091)11:0.01291162083970513914,(((Sebastes_pinniger:0.01736381515507879128,Sebastes_miniatus:0.01833705333225025061)92:0.01737319459540232966,((Sebastes_entomelas:0.00263270996764359893,Sebastes_mystinus:0.00260791703711861673)100:0.00976829459442674249,(Sebastes_melanops:0.00485936914526158541,(Sebastes_serranoides:0.00603352838361181573,Sebastes_flavidus:0.00316051955194770204)50:0.00125638595537515534)100:0.01573441733006947924)66:0.00465673186020265899)18:0.00308537481137817222,(Sebastes_elongatus:0.03328154369327152029,((Sebastes_semicinctus:0.02145277517539051146,Sebastes_saxicola:0.01462283868567769603)76:0.00539980709409692682,(((((Sebastes_atrovirens:0.01320230457462748773,((Sebastes_carnatus:0.00259365583242990696,Sebastes_chrysomelas:0.00392266265881064183)23:0.00122798727525094166,Sebastes_caurinus:0.01291852581270690019)31:0.00000176456401954691)40:0.00127962690150956434,Sebastes_nebulosus:0.01294234000809803796)17:0.00000176456401954691,Sebastes_maliger:0.00383165188169506130)47:0.00126841213264367600,Sebastes_dallii:0.02127962762643654387)9:0.00000176456401954691,(Sebastes_rastrelliger:0.01083455201620963336,Sebastes_auriculatus:0.00942507886690420807)72:0.00229607436128689271)60:0.00324681890025151987)65:0.00599221166385355795)62:0.00784218049359111137)4:0.00115535568368509730)5:0.00142584955742543836,(Sebastes_diploproa:0.00132416311920106780,((Sebastes_cortezi:0.00000176456401954691,(Sebastes_sinensis:0.00260637648170630428,Sebastes_melanosema:0.00129194970144152284)13:0.00000176456401954691)31:0.00000176456401954691,Sebastes_peduncularis:0.00259724281065388911)65:0.00126348641084172248)100:0.02144764332479276447)2:0.00125334982306447851)0:0.00103464705953195389)8:0.00723963304832378821,Sebastes_melanostomus:0.03215846918920687442)6:0.00240175622803245371,((Sebastes_scythropus:0.05634284121820474361,Sebastes_kiyomatsui:0.03109418218723941293)37:0.00632959934484927499,((Sebastes_inermis:0.01098791174047773940,(Sebastes_thompsoni:0.00000176456401954691,Sebastes_joyneri:0.00785675101516460257)100:0.00636636226208741902)99:0.02686337029557082576,((Sebastes_trivittatus:0.00317671456954110370,Sebastes_vulpes:0.01176546847658295827)99:0.00848961650137480643,((Sebastes_pachycephalus:0.02258438943279783562,(Sebastes_oblongus:0.03533214592994960573,Sebastes_hubbsi:0.05339072777700756833)96:0.00807751860931885460)99:0.01731355938975573730,((Sebastes_schlegelii:0.00271383462832519833,Sebastes_koreanus:0.00000176456401954691)100:0.03484757807279640490,Sebastes_taczanowskii:0.02173564770630098328)88:0.01010662971868317786)78:0.00515032013618544650)78:0.01210408428531645382)17:0.00306514890093048447)25:0.00793109634721313704)1:0.00000176456401954691)1:0.00312115345263774347)0:0.00342195200316796786,((Sebastes_ruberrimus:0.02183566143301439097,Sebastes_macdonaldi:0.02987965218400360709)58:0.00999230462685628436,Sebastes_goodei:0.04669380985339983275)11:0.00816920410248488625)15:0.00541137206140381109)17:0.00737744170598677359)65:0.04077937788401399255)58:0.02270336274797889881)37:0.11260136505277644481)31:0.03885272846261981355)10:0.04103345925405492423,((Dendrochirus_biocellatus:0.22679448523721246200,(((Dendrochirus_zebra:0.12871605404648642224,(Pterois_mombasae:0.04108154329945082339,(Pterois_andover:0.12087948104386148085,Pterois_antennata:0.01317477974649471431)90:0.05290583901573531894)34:0.00000176456401954691)93:0.07003743645126347950,(Pterois_miles:0.03518516254360308515,(Pterois_volitans:0.00583270135818072730,Pterois_russelii:0.00304840767624096377)95:0.03050623128385730395)100:0.11224268265495283836)86:0.07091903942589523890,Dendrochirus_brachypterus:0.12832049585988611340)87:0.06242036528621534852)68:0.14970134368114559886,(Trachyscorpia_eschmeyeri:0.17681095841528962631,Setarches_guentheri:0.23716380705195785894)18:0.02344866899226941831)1:0.02840382027267508019)4:0.04782111185022978883)0:0.02412908439930750923,(((((((Sander_vitreus:0.07761723491419172949,Sander_canadensis:0.07737572850192661611)97:0.05486134895123331162,(Sander_lucioperca:0.13024545451767427506,(Zingel_zingel:0.08113756602328266176,Zingel_streber:0.05276727316752388708)98:0.08118640174248112351)48:0.03952568339866986691)35:0.06648099474085152061,(Perca_fluviatilis:0.06074131929855668827,(Perca_schrenkii:0.03875791541173338045,Perca_flavescens:0.05255093307897232280)100:0.03969158932688022362)97:0.10563006090744619936)15:0.02087183483543957399,Gymnocephalus_cernuus:0.23570622769493129733)75:0.04515453392809871264,((((((Etheostoma_fonticola:0.05757589862782221124,Etheostoma_proeliare:0.02399736480725091561)100:0.13266119264182746340,((Etheostoma_microperca:0.19095135445192837342,(Etheostoma_bison:0.03260266973888499564,((Etheostoma_burri:0.03032029157272729536,Etheostoma_spectabile:0.00983100628480216600)90:0.02092171437494334124,((Etheostoma_tecumsehi:0.03178660176288181094,Etheostoma_lawrencei:0.01697530513299624899)83:0.00527952110752975563,Etheostoma_kantuckeense:0.01958743057282090125)86:0.01594235040504378073)59:0.01853651228924154196)99:0.09238644630017818493)38:0.02350971921349079979,Etheostoma_punctulatum:0.13062258316132580038)18:0.01171064220565857551)69:0.05136253439413095145,((Etheostoma_cragini:0.14021820260578832240,(((Etheostoma_boschungi:0.04433891966537981122,Etheostoma_tuscumbia:0.09035560362350654462)100:0.07470925671023952164,((Etheostoma_whipplei:0.06525237450657568605,Etheostoma_radiosum:0.10244405352572555357)95:0.05413163199386185342,(((Etheostoma_lepidum:0.07581864856513256812,(Etheostoma_grahami:0.04651364970652201170,Etheostoma_pottsii:0.00000176456401954691)100:0.08065850111291428259)96:0.04611323393224857348,Etheostoma_australe:0.10018806238057399727)70:0.03237111083903850689,(Etheostoma_luteovinctum:0.11426544739134060491,Etheostoma_exile:0.06793149510280295511)61:0.01514280256250090731)55:0.01693547022098469690)9:0.00000176456401954691)32:0.01595745748062936051,((Etheostoma_ditrema:0.04420036058866181478,Etheostoma_nuchale:0.08081982690927434765)61:0.01479849330359631239,((Etheostoma_uniporum:0.01086797946021564321,(Etheostoma_caeruleum:0.01879252684252510561,Etheostoma_artesiae:0.03122530497037784639)34:0.00000176456401954691)99:0.04892542452052076052,(Etheostoma_swaini:0.07308865475740250472,(Etheostoma_collettei:0.05351534813878574409,Etheostoma_asprigene:0.06849599133803167816)71:0.02373103313695224328)33:0.00268187725784282743)55:0.01291593196161722017)95:0.05931686131935889894)53:0.02175199358811804110)83:0.03870536336602969918,(((Etheostoma_davisoni:0.12267590551728252413,Etheostoma_chlorosomum:0.11512252668332322025)65:0.02157740959883136175,((Etheostoma_akatulo:0.07969618982582055144,(Etheostoma_jessiae:0.05517956618545916819,Etheostoma_stigmaeum:0.06316774885479108093)97:0.03425331498428359212)27:0.00581167407412646383,Etheostoma_meadiae:0.06973381592126097483)93:0.05464040785261376626)61:0.01885034705226497354,Etheostoma_trisella:0.23594788911631286998)58:0.02550678373552125663)18:0.01374263636882651698)0:0.00569124992656657933,(((((Etheostoma_rafinesquei:0.03416522482444647735,Etheostoma_barrenense:0.01895976954739487033)100:0.08769380223059018709,(Etheostoma_tennesseense:0.00000176456401954691,Etheostoma_simoterum:0.00615684694208414908)100:0.09452893306334110568)59:0.03852001304377611346,(Etheostoma_zonale:0.04860768089896511102,Etheostoma_lynceum:0.01861365409174547264)100:0.08066650074121932412)12:0.01305371782865476747,((Etheostoma_blennioides:0.05708235034268548808,(Etheostoma_rupestre:0.03811306523986488642,(Etheostoma_blennius:0.02064405445389309868,Etheostoma_blennioides_gutselli:0.02450913186460134535)97:0.02179527445919890449)74:0.00702462488715946409)90:0.02377479744787054303,(Etheostoma_swannanoa:0.04506352850281033356,(Etheostoma_inscriptum:0.03283604286467262795,Etheostoma_thalassinum:0.06220449046717011626)92:0.01742508343504214560)59:0.01195868685080712576)91:0.05259056086403779418)8:0.00898236151345720678,((((Etheostoma_kennicotti:0.12749071377197687682,Etheostoma_saludae:0.14518962271673255260)9:0.01671547690148212501,(((Etheostoma_virgatum:0.08321599873526328017,((Etheostoma_smithi:0.01525722692224354415,Etheostoma_striatulum:0.02292846821558809298)100:0.03535954787969354529,(Etheostoma_obeyense:0.06865762990213279682,Etheostoma_derivativum:0.03321708086209322619)60:0.01176809305972198461)61:0.02834139503345382838)98:0.04401484910969846037,(Etheostoma_basilare:0.09898710880639460807,(Etheostoma_percnurum:0.10040871563660642984,Etheostoma_flabellare:0.06997002042719506487)81:0.02545337759232903924)42:0.00776437168562930068)33:0.00791186009916195788,Etheostoma_barbouri:0.13300633846576015440)52:0.02199312598122109622)4:0.01166354656900966610,(Etheostoma_serrifer:0.17348894378735768562,(((Etheostoma_okaloosae:0.09653043861878231324,(Etheostoma_zonifer:0.06398735404840018293,Etheostoma_gracile:0.12360519249778009754)90:0.03849577347643915598)99:0.09736008664542747382,(Etheostoma_edwini:0.08937507588229126609,Etheostoma_fricksium:0.11127342773867353320)61:0.01261090123106916629)10:0.02990435383864008950,((Etheostoma_phytophilum:0.00000176456401954691,Etheostoma_parvipinne:0.01066157074409802147)100:0.10330034169472905592,Etheostoma_fusiforme:0.09883100115064273605)61:0.03755497677587810862)6:0.00654091979073955724)5:0.02680135779969424967)1:0.02395091342621979980,(((((Etheostoma_longimanum:0.00879521149775196187,Etheostoma_olmstedi:0.02362417835957155224)87:0.01108495483524725607,Etheostoma_vitreum:0.01186740775393650867)98:0.03812316690656984003,((Etheostoma_podostemone:0.00000176456401954691,Etheostoma_nigrum:0.00877472906605037024)99:0.01800265795994879237,Etheostoma_perlongum:0.05833583285254263784)96:0.02536409784454714267)92:0.04872645746453090065,((Etheostoma_sagitta:0.04941663917432514291,(Etheostoma_variatum:0.03036909341392849890,(Etheostoma_tetrazonum:0.02853363565389888112,Etheostoma_euzonum:0.03293625805950275987)98:0.00652121435906540849)98:0.02853252708971455720)91:0.03193158947955280352,(Etheostoma_osburni:0.00660452614525396022,Etheostoma_kanawhae:0.00000176456401954691)97:0.03358434992129111923)97:0.08940591325169743031)9:0.00990877643923885124,((Etheostoma_histrio:0.19409223224238464356,(Etheostoma_etnieri:0.05536575996163663888,((Etheostoma_chermocki:0.02604943301477944881,(Etheostoma_lachneri:0.01949672089866670219,Etheostoma_bellator:0.02770639374203026639)47:0.00174976755541500755)62:0.01818042796197649399,((Etheostoma_ramseyi:0.04793156398195998896,Etheostoma_tallapoosae:0.02737061235674108015)89:0.03197026339819716151,Etheostoma_zonistium:0.05906438914903693049)35:0.00563805431423020347)60:0.02666735545174139105)76:0.02554205570293353530)30:0.03376341115217709743,((Etheostoma_brevirostrum:0.07428217154176113002,Etheostoma_coosae:0.07741644943662202849)100:0.03300362351970510116,(Etheostoma_flavum:0.04397811788909773700,Etheostoma_duryi:0.05426527985905382767)99:0.04363277816966722178)76:0.02440417388154323894)19:0.02574736536283335139)2:0.00864884561946566849)0:0.00266768583614059593)0:0.01551580449297315298)2:0.01988101623476716193,(((Etheostoma_olivaceum:0.00988520587559274348,Etheostoma_crossopterum:0.00334007716258367992)99:0.05267653319108935028,Etheostoma_squamiceps:0.04460086757993291656)98:0.08162853786334653416,(Etheostoma_fragi:0.19559633118485650338,((Etheostoma_chienense:0.01452194259843536672,Etheostoma_oophylax:0.02180376242580077048)100:0.07675052809777099805,((Etheostoma_nigripinne:0.02725995757828864746,Etheostoma_corona:0.02707268825189566158)97:0.03617093526846374557,Etheostoma_forbesi:0.02991725973314022721)90:0.04195767917607182845)100:0.07331290729339130785)25:0.02182717653598527721)10:0.01760447701962578743)49:0.03949622585424212434,(((Etheostoma_juliae:0.18282689234692703351,((Etheostoma_tippecanoe:0.02669412475377172175,Etheostoma_denoncourti:0.02454767056582203913)100:0.07699763473439494299,(((Etheostoma_chuckwachatte:0.07295104675936167626,((Etheostoma_douglasi:0.02914010442969429404,Etheostoma_jordani:0.01206488112574722119)49:0.00000176456401954691,Etheostoma_etowahae:0.02947991104956735470)100:0.06701770334063722467)96:0.02326590151971635184,Etheostoma_acuticeps:0.05767289021837865987)57:0.02641406246002498889,(((Etheostoma_vulneratum:0.00227876672947039359,Etheostoma_camurum:0.00231424047609015164)99:0.01870064378793045295,((((Etheostoma_cinereum:0.00000176456401954691,Etheostoma_aquali:0.00000176456401954691)100:0.01840310630377018503,Etheostoma_maculatum:0.01322625217735393793)1:0.00000176456401954691,(Etheostoma_microlepidum:0.00435895227086282797,Etheostoma_sanguifluum:0.00700475927170155725)58:0.00230116073433319192)97:0.02324595310121810310,(Etheostoma_chlorobranchium:0.00958263980878293342,(Etheostoma_bellum:0.02509537987505868623,(Etheostoma_rubrum:0.00438590833366881653,Etheostoma_moorei:0.01430877527822957233)98:0.00934316336900397303)72:0.00746100842002856736)97:0.02542388491501090808)53:0.00235496070650671654)89:0.01784226097755828647,Etheostoma_rufilineatum:0.05510000049569232367)27:0.00000176456401954691)26:0.01620020785233142191)87:0.07865114257007950471)72:0.04174902819212092386,((Percina_roanoka:0.07436567602659881204,(Percina_crassa:0.02855593235914713826,Percina_peltata:0.05779797668499223051)98:0.04327493475286289804)99:0.07465723767083987106,((((Percina_aurantiaca:0.08031497795909652015,Percina_evides:0.06265282490196488752)70:0.02709834964393066994,(Percina_nigrofasciata:0.10181691551791600958,(Percina_lenticula:0.08807487898702098805,(((Percina_vigil:0.04280817159945410344,Percina_shumardi:0.02385580867612495035)61:0.01000769739783431700,((Percina_tanasi:0.00639136869843493806,Percina_uranidea:0.00943288774739974543)100:0.05077697426680542053,((((Percina_nasuta:0.01914090192265677920,Percina_phoxocephala:0.00718574673450737399)96:0.03100724882911898295,(Percina_gymnocephala:0.07565516052912127365,(Percina_oxyrhynchus:0.01578642270900549802,Percina_squamata:0.00931022836966036507)84:0.01619812495719478995)3:0.00000176456401954691)24:0.00572618584974569477,Percina_cymatotaenia:0.05302958842074214502)32:0.01542811147641749529,((Percina_stictogaster:0.04741314117277678486,(Percina_sciera:0.04374440445147004947,Percina_aurolineata:0.03595484097947172775)99:0.05247040597365607018)47:0.00841724001596720617,((Percina_pantherina:0.01732934495312132145,Percina_notogramma:0.01633498254876685277)69:0.00268854213042339337,Percina_maculata:0.01088461121510209823)100:0.03613535281457879583)34:0.00645737849864122266)18:0.00902776167613613192)3:0.00420983864956219790)1:0.00857117960028865380,(Percina_antesella:0.03814691448666717583,(Percina_aurora:0.05175576213389013885,Percina_copelandi:0.04456470093702582375)46:0.01432944662484863553)17:0.00688942930193272175)5:0.00000176456401954691)37:0.01678738867125975989)11:0.00072273987116516086)5:0.00769369431208363937,((Percina_rex:0.03415859325511540201,(Percina_jenkinsi:0.02212678941129940513,(Percina_burtoni:0.02308127597745778664,((Percina_carbonaria:0.01736848196047028475,(Percina_austroperca:0.01276825125256249131,(Percina_kathae:0.00419427646694240427,Percina_bimaculata:0.00000176456401954691)77:0.00000176456401954691)89:0.00622701542279892849)68:0.00421886804235683914,(Percina_caprodes:0.00415038369721859417,(Percina_macrolepida:0.00000176456401954691,Percina_suttkusi:0.00204827975790203591)77:0.00206572541717173902)64:0.00000176456401954691)89:0.01261604522221454125)91:0.02493862934931298167)89:0.02745926680936579614)90:0.04381625045424628745,Percina_macrocephala:0.12209036325046553062)5:0.00000176456401954691)18:0.00906554997551297162,(Percina_palmaris:0.09429432163102274833,Percina_smithvanizi:0.07723526720516966926)40:0.01695103114715523079)65:0.03536049572644109351)67:0.04026898314556315306)22:0.02183488541703487024,(Crystallaria_asprella:0.19313088595557248439,(((Ammocrypta_bifascia:0.00527814605896869543,Ammocrypta_beanii:0.00639356135476279366)100:0.10040249322314731550,(Ammocrypta_meridiana:0.03303184133727635891,Ammocrypta_vivax:0.06141491575661008062)81:0.01538717564472426996)96:0.07318017365257298290,(Ammocrypta_clara:0.08901076987594559731,Ammocrypta_pellucida:0.08050321115450874421)82:0.02441438795024183581)87:0.06669118510154886659)48:0.02317053655203704307)10:0.00896631893463149809)66:0.07201488959295415659)56:0.07148097393368982311,(Ammodytes_hexapterus:0.58632220425219405691,(((Xenocephalus_armatus:0.20934185865579119090,(Kathetostoma_averruncus:0.14682899456459655441,Kathetostoma_giganteum:0.12664494100324005799)95:0.14054403040779775425)80:0.06962783152918229634,(Uranoscopus_archionema:0.07390748463902130361,Uranoscopus_oligolepis:0.14124875783339579938)95:0.46884209513818392301)59:0.20953474379356681889,(Cheimarrichthys_fosteri:0.25615453891758943605,(Pseudopercis_semifasciata:0.03395351583531276002,Pinguipes_brasilianus:0.08292193045032505982)91:0.13400565570089004175)82:0.15189186974261800755)12:0.03069299048163217844)4:0.03496165783577450531)0:0.01376219800152172351,((Scorpaena_guttata:0.21392650744046098898,(Aulacocephalus_temminckii:0.32550795827180917241,((Scorpaena_notata:0.24895240447286187679,Scorpaena_scrofa:0.05592555493686037632)86:0.14444184133351001265,(Scorpaena_cardinalis:0.12678104981821355923,Scorpaena_colorata:0.23955165552544926966)60:0.04822566866930146773)36:0.05126884347051392787)15:0.06427752429036986959)13:0.06845570618010553277,(((Peristedion_weberi:0.24524062908266427985,(Satyrichthys_adeni:0.05660591287166077956,Scalicus_amiscus:0.10147955951782748618)97:0.06945245899549208624)61:0.07708635208733115307,((Pterygotrigla_polyommata:0.04323307514618227043,Pterygotrigla_pauli:0.06962864272537439003)100:0.24389411701831759105,(Trigla_lyra:0.10602493601637308362,((Lepidotrigla_faurei:0.12317602716550120345,(Lepidotrigla_mulhalli:0.02735461511089172515,Lepidotrigla_vanessa:0.03499922140742852544)100:0.08664356549008660202)79:0.05648034239054480071,((Eutrigla_gurnardus:0.01652831137899808678,((Chelidonichthys_capensis:0.00918848427202080716,((Congiopodus_torvus:0.00179870752696187136,Chelidonichthys_kumu:0.01366803131156038922)72:0.00554013249214073064,Chelidonichthys_lucernus:0.02380238611818108821)46:0.00325958710479824169)96:0.04106064543184340804,Chelidonichthys_cuculus:0.02795229635142462352)29:0.01400035774467258201)89:0.04711203883992030883,Trigloporus_lastoviza:0.05101476205916661427)94:0.07272705523914013703)69:0.04775049856613518967)65:0.04597951213654603853)42:0.04931129763465395832)34:0.09768936297096965371,(Oxylebius_pictus:0.27764262807647488440,(((Bathymaster_derjugini:0.02806081845727803267,Bathymaster_leurolepis:0.03619181248353334907)100:0.11381862167185151369,((Rhodymenichthys_dolichogaster:0.09025805057876011561,(((Pholis_fasciata:0.00519234086609141835,(Pholis_crassispina:0.01139421036053794856,Pholis_picta:0.00000176456401954691)88:0.01265700078057626493)84:0.02404794820240360384,(((Pholis_clemensi:0.03911765724091646051,Pholis_laeta:0.05245852303463405947)29:0.01221021061757738072,Pholis_ornata:0.04681861313931121732)32:0.01265383005552649272,Pholis_nebulosa:0.05201286612304586998)20:0.00748833638022617428)89:0.05445868706723041436,Apodichthys_flavidus:0.14244227003325085623)83:0.02646255617270057772)84:0.05775911279148741168,(((((((Lycenchelys_camchatica:0.05220199304640937843,(Lycodes_albonotatus:0.01099248458170203058,Lycodes_toyamensis:0.01213968701715580946)100:0.01888445016925753908)69:0.01391130913344381347,(((Pachycara_thermophilum:0.03825360697501159479,(Pachycara_lepinium:0.03495831783204361720,Pachycara_gymninium:0.02870915496820559965)60:0.00442962880554422476)56:0.00447777620804115043,((Oidiphorus_mcallisteri:0.02708935764943526392,(Melanostigma_pammelas:0.05750790137472343300,(Iluocoetes_fimbriatus:0.05084483460350788797,Austrolycus_laticinctus:0.06966371233198237478)95:0.01923122810761460078)62:0.00961283256165164877)52:0.01106673549118845391,(Lycenchelys_aratrirostris:0.01049599726855960508,(Pachycara_brachycephalum:0.01221524886535871150,Lycenchelys_tristichodon:0.02119607094962928581)39:0.00401687053599639968)39:0.00674659385492725090)12:0.00257867370011062623)24:0.00231206066234736479,((Ophthalmolycus_amberensis:0.02166567596196099926,Lycodichthys_antarcticus:0.02278805992550590742)34:0.00468684173465460947,Lycenchelys_jordani:0.03153205542215724300)33:0.00426836184753858448)62:0.01249638587376997352)53:0.01331423530870642913,(((Lycogrammoides_schmidti:0.03489218404765829368,Bothrocarina_nigrocaudata:0.04167224475335250949)70:0.00754632596034236162,Bothrocarina_microcephala:0.02682841759660388459)49:0.01566965611696361296,((((Lycodapus_fierasfer:0.01989479527467709341,(Lycodapus_leptus:0.02321684294030064433,(Lycodapus_endemoscotus:0.01791539051129566185,Lycodapus_mandibularis:0.01608296680864844397)71:0.00435863864274215352)58:0.00207211350312394224)73:0.00800594231001162948,Lycodapus_pachysoma:0.02643334763220642203)64:0.00607021615808654973,((Lycodes_cortezianus:0.05683085462949815508,Lycodes_diapterus:0.05584521018174521539)37:0.00622496107936178298,(((Lycodes_frigidus:0.01202647349412245062,Lycodes_multifasciatus:0.02370249752574262250)84:0.00891887859706266307,(Lycodes_raridens:0.02146513105449107672,((Lycodes_turneri:0.01245214476079369720,Lycodes_seminudus:0.00000176456401954691)53:0.00208580377649876482,(Lycodes_mucosus:0.00413654081369130809,(Lycodes_polaris:0.00963953047141073889,Lycodes_knipowitschi:0.00441059143720718468)100:0.01219784414253103320)77:0.00210584569251435995)93:0.01120112323703478276)77:0.01046237665196206833)55:0.00988450357430285288,((Lycodes_nakamurae:0.02802208305314556752,((Lycodes_soldatovi:0.01023322974795037171,Lycodes_brunneofasciatus:0.01668071770524134217)85:0.01065437458584837967,Lycodes_yamatoi:0.02457750344597021269)44:0.00217414514267106436)23:0.00239959594605904840,Lycodes_fulvus:0.05002862855098932221)20:0.00171457781489340005)88:0.02553088966097716642)23:0.00686618434502563740)30:0.00647406771765429104,((Lyconema_barbatum:0.13993174651906398198,Bothrocara_hollandi:0.02750206886225136857)21:0.01337155866195970447,((Bothrocara_pusillum:0.00258771700501421887,Bothrocara_molle:0.01269001248060770345)96:0.02599528963902289491,(Bothrocara_soldatovi:0.00840669312375869580,Bothrocara_brunneum:0.00238378647345546769)100:0.01917680443155319464)95:0.02458975553434408767)5:0.00241647413567542147)11:0.00653459716412070363)63:0.02199835999567754594)94:0.04142376428461157223,(Zoarces_americanus:0.04937801953046631886,(Zoarces_andriashevi:0.00611401645601475884,(Zoarces_elongatus:0.01746019912216489542,(Zoarces_viviparus:0.00863008542398155734,(Zoarces_fedorovi:0.00114943102310154500,Pholidapus_dybowskii:0.01292386494181387881)67:0.00170927403606185445)94:0.01567223116185768872)80:0.01006889453088794482)75:0.01411025030312882018)100:0.09153197027114112760)39:0.01263567039975915449,((Anarhichas_lupus:0.08527899577602637837,(Gymnelus_hemifasciatus:0.00549724007778377988,Gymnelus_viridis:0.01011194038761288778)100:0.05766620964190256882)56:0.01723097583519665743,(Gymnelopsis_ochotensis:0.08667902229027382721,(Bilabria_ornata:0.08899317909954597428,(Hadropareia_middendorffii:0.04356050681533216867,Magadanichthys_skopetsi:0.04328988253247328943)99:0.05361796532365534912)72:0.02632800264367202159)65:0.02412727937146604343)12:0.00900595822120711133)20:0.01673806251153647090,Neozoarces_pulcher:0.15775398598004497508)95:0.05073814660633589602,((Plectobranchus_evides:0.18019414881792047978,Ptilichthys_goodei:0.15322194868462918338)18:0.02156008877052240091,(((((Xiphister_mucosus:0.15094550063731290712,((Chirolophis_decoratus:0.07208258803484901556,Chirolophis_japonicus:0.07936093761280001158)99:0.07055016489010608460,((Ernogrammus_hexagrammus:0.16209258180974628338,((Stichaeus_grigorjewi:0.00456802097169019088,Stichaeus_nozawae:0.00000176456401954691)99:0.11637611670812489373,Eumesogrammus_praecisus:0.08393092315957075000)46:0.01279292134311475759)44:0.02941860831639088458,Stichaeopsis_nevelskoi:0.06599087740181995565)29:0.01186890083504114554)12:0.01378759280390061577)6:0.00000176456401954691,(Alectrias_alectrolophus:0.09421699732798802240,Anoplarchus_purpurescens:0.06932238523385335360)88:0.06524521774254329887)18:0.01003574593544735716,(Stichaeus_ochriamkini:0.12098177761437550748,Apodichthys_fucorum:0.13748255488845292915)45:0.03119432641726963343)50:0.00857024439014713105,((Opisthocentrus_tenuis:0.13557966240224289534,Kasatkia_seigeli:0.10757091131266646733)71:0.05607118916890619503,((Rathbunella_hypoplecta:0.23468280967075730303,Ronquilus_jordani:0.11532978015277610406)23:0.01127556880424891023,(Esselenichthys_carli:0.13838381011427286493,(Cebidichthys_violaceus:0.06390688838547491091,(Dictyosoma_rubrimaculatum:0.00000176456401954691,Dictyosoma_burgeri:0.02093956582852126921)100:0.13220002369487240945)100:0.05632188593240566171)84:0.03956489861329731683)32:0.03368601606077563371)3:0.01266662453786046062)8:0.02767796993739344083,(((Cryptacanthodes_aleutensis:0.07410674785409504528,Cryptacanthodes_giganteus:0.04894015113098198339)98:0.06213601632528054086,(((Lumpenus_sagitta:0.00124814758744641249,Lumpenus_fabricii:0.00318078341140976256)100:0.06383555046002335753,(Anisarchus_medius:0.07913503041678805749,Lumpenella_longirostris:0.06413898293759938418)48:0.00912328404300508794)41:0.02029819686524868272,Poroclinus_rothrocki:0.06370620908326991849)23:0.01000545999276579966)9:0.00584164809770260866,Zaprora_silenus:0.13445846638034358866)27:0.02383802943086065634)11:0.01281352052725132432)10:0.01138801512679044579)29:0.02489509896589366458)33:0.03088140091519445476)88:0.09772849671235310509,(((Pleurogrammus_azonus:0.04262516923882959941,Pleurogrammus_monopterygius:0.05152944377440497881)99:0.09346872857597202100,(((Hexagrammos_stelleri:0.02815877184739811953,Hexagrammos_otakii:0.03756118279010092764)29:0.00695278247552088072,Hexagrammos_lagocephalus:0.04271363088933085445)36:0.01713675666303066114,Hexagrammos_decagrammus:0.07190057296593234393)86:0.04319704551465702869)90:0.09032024419010666050,((((Hemilepidotus_spinosus:0.06772577573447538524,Hemilepidotus_papilio:0.03331068749716638300)93:0.06485376215990520943,(((Hemitripterus_villosus:0.11302968103926522292,(Blepsias_cirrhosus:0.00801908666497324132,Nautichthys_robustus:0.00996535289293679939)100:0.11877147620081741908)45:0.02140228893189739801,Scorpaenichthys_marmoratus:0.14556099809650163701)19:0.01923026327725414417,((((Podothecus_sachi:0.02918914879501744580,Podothecus_accipenserinus:0.02155484957870343679)99:0.05768099897192251885,(Ulcina_olrikii:0.00818143844641151113,Aspidophoroides_monopterygius:0.01506327246949241104)100:0.07735744596493802916)85:0.05120467472017912330,((Nautichthys_oculofasciatus:0.16911540992292328767,Nautichthys_pribilovius:0.10400193351415844278)88:0.11042599432897597100,(((Odontopyxis_trispinosa:0.08514562377876361277,(Bathyagonus_infraspinatus:0.01781716374206488915,(Bathyagonus_pentacanthus:0.00915574859042369085,(Bathyagonus_nigripinnis:0.00908403772941164880,(Xeneretmus_leiops:0.01143758551186364533,Xeneretmus_latifrons:0.01120592993015344201)93:0.01112259109046497343)18:0.00000176456401954691)21:0.01059296434304746529)29:0.01370124041930839177)62:0.03477469874776676489,(Agonopsis_sterletus:0.04832559976429819104,Agonopsis_vulsa:0.04417234559513161934)99:0.05892808993606910017)84:0.06127869234023680667,((Chesnonia_verrucosa:0.14987870773872208341,Pallasina_barbata:0.03319805660569476874)67:0.02714795644858137430,Stellerina_xyosterna:0.11282744698301344632)63:0.01873007432040012937)27:0.01843042395714313345)11:0.00307818933418839771)43:0.02293162799994488688,((Rhamphocottus_richardsonii:0.00000176456401954691,Rhamphocottus_richardsoni:0.00000176456401954691)100:0.18755260371940765673,(Trichocottus_brashnikovi:0.08557561318570670872,(((Bathyagonus_alascanus:0.00000176456401954691,Radulinus_asprellus:0.00908065969242329886)100:0.12242553866551149355,(Zesticelus_profundorum:0.05408089921739810818,(Artediellus_scaber:0.00863143428253792394,Artediellus_atlanticus:0.01612849357580847451)99:0.01677947764488113905)98:0.05940652302530397000)44:0.02379825143834047793,((((Triglops_pingelii:0.01762370287479127565,Triglops_nybelini:0.02886825197436306348)100:0.08674133235623213145,((Icelinus_oculatus:0.00000176456401954691,Icelinus_fimbriatus:0.01323773169665154979)94:0.02236706260601786442,(((Icelus_spiniger:0.01506341559075232046,Icelinus_cavifrons:0.01693861784951200891)100:0.05040661223004215541,(Icelinus_quadriseriatus:0.08865217729899511900,(Icelinus_tenuis:0.00218648282349785065,Icelinus_filamentosus:0.00000176456401954691)100:0.01497067145772689421)45:0.00902483994467934841)66:0.01464116996617221937,Icelinus_burchami:0.04726461925300240452)21:0.00391933005821750325)100:0.07412385976981658575)2:0.00977645093115650163,(((Chitonotus_pugetensis:0.15971412550668273589,(Ruscarius_creaseri:0.18952458485019438306,((((Leiocottus_hirundo:0.15440188694392573132,Clinocottus_analis:0.10928990501174862260)40:0.02148220414751251042,(Oligocottus_rimensis:0.31586034660156409037,(Oligocottus_rubellio:0.18347808078690439060,(Oligocottus_maculosus:0.04405046855336420403,Oligocottus_snyderi:0.15849756894847769062)94:0.04981987007389070826)71:0.02175787747343269074)18:0.02635831830547838428)15:0.01820957464843681040,(Orthonopias_triacis:0.23220888521554808315,(Clinocottus_embryum:0.06399601920510281095,(Clinocottus_recalvus:0.11027262962313251482,Clinocottus_globiceps:0.05955065354622505330)80:0.03120000593841772835)82:0.03964717322217460593)46:0.02086512324426950324)24:0.02641715653004355138,(((Artedius_fenestralis:0.05825916451776402827,Artedius_notospilotus:0.07056449990813920548)73:0.01774087893831158280,((Artedius_corallinus:0.02722624307351225945,Artedius_lateralis:0.07299909588796431315)97:0.09682070025435013383,Artedius_harringtoni:0.18879550045693199722)30:0.01724539048469246605)50:0.03273337554895418278,(Synchirus_gilli:0.19113726040804637774,Clinocottus_acuticeps:0.11508320581223663304)36:0.03525910115915581455)58:0.03736247997659382369)72:0.05683664801135551031)81:0.02813861970665767695)92:0.07511677148877098087,((Cottus_poecilopus:0.06266606799401866490,((Cottus_aleuticus:0.02311210910319812933,((((Cottus_asper:0.00671888179456461929,Cottus_gulosus:0.00247705032715602250)80:0.00485660878810125277,Cottus_perplexus:0.01870356312254967818)47:0.00442896352763564455,Cottus_pitensis:0.00709383929629790917)45:0.00818243768671074283,(Cottus_klamathensis:0.01896887288294330301,Cottus_tenuis:0.00901415781342304254)82:0.04561106897416380979)13:0.00572160501974795441)28:0.01943709375776199802,((Cottus_ricei:0.00484639593062269509,(((Cottus_aturi:0.00997790692735466000,Cottus_gobio:0.00723583264572882218)68:0.00241852212747391531,(Cottus_rhenanus:0.00967067235271233498,(Cottus_duranii:0.00478248337811581527,Cottus_perifretum:0.00238732603352158259)34:0.00000176456401954691)68:0.00242000866583798541)77:0.00514972911706777520,Cottus_sibiricus:0.00725821110037090742)52:0.00477536399738643334)81:0.01315001283404449058,((Cottus_hypselurus:0.05851871964288660882,((((((Cottus_bairdii:0.01613968768264561690,((Cottus_tallapoosae:0.00544485133776684196,Cottus_chattahoochee:0.00107539945351563856)100:0.03991162606713748867,Cottus_carolinae:0.00910361966480794163)70:0.01189370262128641199)18:0.00000176456401954691,Cottus_caeruleomentum:0.01625208721785863925)37:0.00431057189430494349,Cottus_cognatus:0.02096087573935131662)12:0.00000176456401954691,Cottus_rhotheus:0.01371868616491334590)19:0.00231098664710232713,Cottus_hubbsi:0.01132974836819710188)15:0.00239792794131954857,Cottus_girardi:0.02638563148720497048)41:0.00848191881931779391)67:0.01721646713310154633,Cottus_beldingii:0.07531086218607946359)48:0.00392294904665592761)55:0.01794560057508090556)24:0.03010548947644118428)17:0.02328880735443383809,(Trachidermus_fasciatus:0.27071178195519229881,Leptocottus_armatus:0.21189787154859118790)25:0.02262224208992746899)70:0.12591170103531873492)5:0.02377950576207544728,(Gymnocanthus_tricuspis:0.02362901671269947504,Gymnocanthus_intermedius:0.06311121129244831596)99:0.04925392924670937700)1:0.01219584684598217991)0:0.00641409668129223464,((Enophrys_diceraus:0.09549366184150007153,((((Triglopsis_quadricornis:0.00000176456401954691,Myoxocephalus_thompsonii:0.00655578652594660319)100:0.02029422297971202396,Megalocottus_platycephalus:0.03276650286978700921)98:0.04750962052416396464,(Myoxocephalus_scorpius:0.02583739058387825305,(Myoxocephalus_ochotensis:0.02247662337526937920,(Myoxocephalus_jaok:0.00093271730878370044,Myoxocephalus_brandtii:0.01048176643061105202)89:0.00205834564133787219)85:0.01966310951962936190)100:0.06060158820469063734)48:0.01802148780515336335,(Enophrys_bison:0.07268783363883138393,Enophrys_taurina:0.05915205040725120267)100:0.07583942659029134503)38:0.00672378861453399538)94:0.07293027858449457890,((Malacocottus_zonurus:0.10594006280379958396,(Psychrolutes_sigalutes:0.10450531924797698546,(Psychrolutes_phrictus:0.02978320526605860927,(Ebinania_vermiculata:0.02139534185285045867,Cottunculus_microps:0.02501989599650743940)65:0.01450300308985835729)85:0.04326836329314355523)46:0.00782448798767460159)100:0.05119387972409237403,Icelus_spatula:0.07767426271549954242)30:0.01516504474811948902)10:0.00451639067922787827)3:0.01439011352666595342)14:0.02117793243606181167)37:0.02948353622368900251)13:0.02343884296515555757)5:0.01668690516607168953)6:0.02165824669364897065)24:0.05327882185621544281,(Trichodon_trichodon:0.22221730095422387308,((Nectoliparis_pelagicus:0.13555704799437837127,((Liparis_florae:0.15032705626757561501,((((Liparis_chefuensis:0.01700825142967249751,Liparis_tanakae:0.00104055145516610079)100:0.08423351809082819941,((Liparis_gibbus:0.00676400651175426346,Liparis_bathyarcticus:0.00220710427565857636)98:0.02839651514776594596,(Liparis_dennyi:0.00000176456401954691,Liparis_fucensis:0.00218256985283806848)85:0.01477569000018299009)44:0.00944113438641194271)51:0.02173027800884950317,(Liparis_tunicatus:0.01042623767797692946,Liparis_fabricii:0.01032350611791656853)92:0.03520542143159799409)79:0.03573656129654009111,(Liparis_pulchellus:0.12227692971867744343,Liparis_mucosus:0.14305708982332515644)85:0.06310760075900248567)67:0.02818695403736809491)73:0.04064864936839314435,(((((Careproctus_melanurus:0.05163701913938806981,(Careproctus_cypselurus:0.00000176456401954691,Careproctus_furcellus:0.00000176456401954691)100:0.01389385120039555131)86:0.01938334442187619744,(Careproctus_canus:0.03972483206215299267,Careproctus_georgianus:0.05346345795547079865)59:0.01073045194602587245)100:0.12356181563506321575,(Careproctus_rastrinus:0.05491146031024198709,Elassodiscus_caudatus:0.05926475478634384009)63:0.01418661347040428219)95:0.04994603158884357297,Acantholiparis_opercularis:0.06981029774667973919)70:0.00778294373764473572,((Careproctus_attenuatus:0.04807130373999724676,(Careproctus_longipectoralis:0.03381741857246143429,Careproctus_continentalis:0.02205450596054362583)70:0.01645263672553393119)68:0.01038629459611400442,((Rhinoliparis_attenuatus:0.00221918055684228047,Rhinoliparis_barbulifer:0.00000176456401954691)100:0.06011805791277084060,((Paraliparis_bathybius:0.06625848155620311930,Paraliparis_rosaceus:0.07083280436745656439)41:0.02348763643085785904,(((((Paraliparis_valentinae:0.01852035218459122112,(Paraliparis_antarcticus:0.01341792834297853530,(Paraliparis_leobergi:0.00898229912806103835,Paraliparis_charcoti:0.00219509778235905950)95:0.00497857852106969780)93:0.01250692513997944157)78:0.01862693202619254695,Edentoliparis_terraenovae:0.03716062874763830653)85:0.01572381783618407095,(Paraliparis_mawsoni:0.03901702614195463881,Paraliparis_paucidens:0.02120329666165726365)95:0.02279341625852983333)40:0.00560566108843788425,(Lipariscus_nanus:0.06485981680623048584,(Paraliparis_melanobranchius:0.01589638914331151578,Paraliparis_dactylosus:0.01995155441942339514)100:0.03180609210030559825)52:0.00682067337012270004)23:0.00493613218008490638,Paraliparis_pectoralis:0.12462607422618074415)6:0.00499299208839754875)21:0.00752432098868008326)44:0.02723145096248960270)22:0.00837342029056997034)97:0.06524190250849898343)91:0.10200186120947615787)97:0.13687615205867881274,((Eumicrotremus_derjugini:0.06011746659426117634,(Cyclopterus_lumpus:0.15277016971803680789,Eumicrotremus_spinosus:0.04327704885568931653)25:0.00315933453868475194)36:0.01768846897769258547,Aptocyclus_ventricosus:0.07158190633937555614)70:0.05203607845907807367)93:0.09291406825896091071)20:0.02281323530686475698)23:0.03243395348244919318,Ophiodon_elongatus:0.18354255913324518668)35:0.02647704162801505640)47:0.09175558079334834860)10:0.02116280190460266553)1:0.02828252953239892772)1:0.02169628165982369697)0:0.02428790482562327713)0:0.01043286644527720818)0:0.02889587895594775135)0:0.02723239435420965471)0:0.00000176456401954691)0:0.02559807343525389092,(Inimicus_sinensis:0.36599114290633827373,(((((Thyrsites_atun:0.45931814952114258244,Nesiarchus_nasutus:0.12039221831130195173)58:0.04743258074856452483,Scombrolabrax_heterolepis:0.16290799598048211205)16:0.04194738954788077012,(((Nealotus_tripes:0.29842383537095007462,((Diplospinus_multistriatus:0.28168443695231326629,Paradiplospinus_antarcticus:0.33828970166788641105)70:0.18746505406366900659,Promethichthys_prometheus:0.25432199253342308021)5:0.04033210259403591930)2:0.03620675190168967661,Rexea_solandri:0.18894576207695640280)0:0.02879836527065587556,Gempylus_serpens:0.32044639696071569590)0:0.01886294032547310151)0:0.03764125787815804369,(Lepidocybium_flavobrunneum:0.22372294397456149451,((Neoepinnula_orientalis:0.09523066903646976167,Neoepinnula_americana:0.13899058183057705351)98:0.22541578869902331728,(((Pterycombus_brama:0.00000176456401954691,Pterycombus_petersii:0.02980193629024088450)100:0.21331682424933687270,((Taractichthys_longipinnis:0.02381787326951243997,Taractichthys_steindachneri:0.04236431346158274491)96:0.10626966206774032808,((Brama_brama:0.01898927672437435155,Brama_japonica:0.04885907366692981285)100:0.16246037202691948620,(Taractes_asper:0.00000176456401954691,Taractes_rubescens:0.00227222333720719172)100:0.14772473801324217946)31:0.03872747834856682392)17:0.00551628490248379146)39:0.03424248603860552409,Xenobrama_microlepis:0.10200428218598528440)86:0.06066610509809623047)22:0.04208877638625268397)1:0.02622571469775303310)0:0.01854404339290489229,((((Pampus_punctatissimus:0.05260478611058830301,Pampus_chinensis:0.04713384581460836747)91:0.06150671512644503847,((Pampus_cinereus:0.00238854600549053995,Pampus_minor:0.00000176456401954691)100:0.20892255076382174050,Pampus_argenteus:0.20581428036748189436)50:0.02867537818100434829)100:0.26927989692203924132,((Stromateus_stellatus:0.00000176456401954691,Stromateus_brasiliensis:0.00221808726643636179)100:0.10868727692424894060,(Peprilus_medius:0.08230343115397273368,Peprilus_simillimus:0.04017271773857954142)100:0.08479794603761839356)96:0.07276958378675162098)36:0.06256736330151439762,((Benthodesmus_simonyi:0.36772027386320171871,((((Lepturacanthus_savala:0.13390202382356486410,((Trichiurus_russelli:0.17352515155955788861,Trichiurus_lepturus:0.05502621884441230571)98:0.04668153052219396054,Trichiurus_japonicus:0.10935643017590614134)99:0.12618919408667483850)99:0.25926857924790081000,(Lepidopus_altifrons:0.25494742401638131879,Assurger_anzac:0.18669919230871712124)60:0.04165558493133942469)35:0.01990859802318284411,Lepidopus_caudatus:0.22239943061927477941)61:0.07919615955553138686,(Aphanopus_intermedius:0.00546923719144186676,(Benthodesmus_elongatus:0.00000176456401954691,Aphanopus_carbo:0.00000176456401954691)100:0.00972840952036944581)99:0.17261993394731786622)20:0.04208147762532307018)48:0.08239556728926493434,(((Acanthocybium_solandri:0.15231529516107664146,Ruvettus_pretiosus:0.18339834551402386942)5:0.05365270694884855668,((((Kali_indica:0.06016008590177158305,Kali_macrura:0.03690688957846809809)100:0.20834950174725075378,Dysalotus_alcocki:0.16214229576329941551)74:0.17714871527385075978,Tetragonurus_cuvieri:0.20635332026863004162)17:0.03855716388632952907,(((Ariomma_brevimanus:0.21174035576832558880,(Ariomma_indica:0.13589236029526299832,Ariomma_lurida:0.20546663649276145081)66:0.05414512666282192410)64:0.06249765297441284284,((Cubiceps_gracilis:0.03169535767261238285,(((Cubiceps_whiteleggii:0.00225583267946236239,Cubiceps_squamiceps:0.00000176456401954691)100:0.11151864816285830817,((Hyperoglyphe_pringlei:0.00000176456401954691,Psenes_cyanophrys:0.00000176456401954691)100:0.06601282427906511596,((Psenes_arafurensis:0.07644379492667399689,Psenes_maculatus:0.05028779791983897796)87:0.03071746691540143057,Psenes_pellucidus:0.06871549219180687895)82:0.01892964954557981028)99:0.08277446488821330051)93:0.04707750520095477231,(Cubiceps_baxteri:0.00299151616376202613,Cubiceps_capensis:0.00208678025414721531)100:0.04800810413950869909)32:0.01183697712337826273)38:0.00923738555228641525,Cubiceps_paradoxus:0.10811544129983376161)71:0.03648006073807004424)37:0.03244842181278102811,(Icosteus_aenigmaticus:0.11987937677886884025,(Caristius_macropus:0.04260384044863477909,Paracaristius_maderensis:0.04490388344018105687)100:0.14612862207344068377)46:0.03414114579259535515)2:0.01883913691435500082)6:0.00166300524884069865)0:0.01577620976744480846,(((((Centrolophus_niger:0.10639179404214335611,(Tubbia_tasmanica:0.04492781187064794762,((Schedophilus_ovalis:0.00592137277792154863,Hyperoglyphe_moselii:0.00000176456401954691)97:0.01979412138948853997,Schedophilus_labyrinthicus:0.01634199837391269589)98:0.02840745493729496599)70:0.01246961639539182105)75:0.01412341252743273302,Icichthys_lockingtoni:0.08469178930971565933)90:0.03922091017615788461,(((Seriolella_punctata:0.00000176456401954691,Seriolella_porosa:0.00228647361159934259)100:0.02905762408259260354,((Psenopsis_anomala:0.27589950200354801391,Psenopsis_cyanea:0.10902836054497568041)93:0.16777719200642918218,(Seriolella_caerulea:0.03847931021989237782,Seriolella_brama:0.03337664766628124086)78:0.00570327464971041170)20:0.00366673753381418187)53:0.03077078893442636298,Hyperoglyphe_antarctica:0.10743411669224639526)40:0.01434697894253445828)96:0.12690343344903390332,((Arripis_georgianus:0.16425554106116072028,(Arripis_truttaceus:0.00938500689281203740,Arripis_trutta:0.00938274991078321373)100:0.15339453912912690003)100:0.16367265071921724418,Kali_normani:0.31608840510660407563)13:0.07704722626674483099)2:0.02947972391411408238,(Pomatomus_saltatrix:0.24994237956677867185,(Gasterochisma_melampus:0.22595939963392172589,((Grammatorcynus_bicarinatus:0.25832503794326266977,(((Scomberomorus_sierra:0.05241793958298884837,((Scomberomorus_maculatus:0.00341302475846649769,Scomberomorus_regalis:0.00437063472640783837)61:0.01173906520792373544,Scomberomorus_brasiliensis:0.01667829991331614553)51:0.00716889471372424581)100:0.14717371133940593264,(Scomberomorus_semifasciatus:0.20234969108259673320,Scomberomorus_cavalla:0.12339935457618993186)31:0.02606348917461538336)11:0.00597492712923373276,((Scomberomorus_queenslandicus:0.06886080048495842043,Scomberomorus_commerson:0.01387006743672014597)100:0.09603963203281898342,((Scomberomorus_munroi:0.12832938104493846199,Scomberomorus_niphonius:0.08357537986970159372)73:0.03415327914933689174,Scomberomorus_guttatus:0.16773275792775266257)46:0.03569487568836438030)35:0.02588698340695671368)77:0.03442929205560015665)15:0.03101191616264386761,(((((Rastrelliger_brachysoma:0.01270577287948737588,(Rastrelliger_kanagurta:0.00000176456401954691,Rastrelliger_faughni:0.00000176456401954691)100:0.00741163270387474801)100:0.24312453485980972667,(Scomber_scombrus:0.13751737575666181379,((Scomber_japonicus:0.01029508345742827582,Scomber_colias:0.00940681074982132638)56:0.00633084519220954156,Scomber_australasicus:0.00517270682605562148)99:0.06825296705917539841)100:0.19512052696618242820)37:0.07838408416149751645,(((Auxis_rochei:0.02412845109235296770,(Auxis_thazard:0.00364383395289708499,Sarda_orientalis:0.00000176456401954691)100:0.01963263208064085913)99:0.06287911509742746818,Katsuwonus_pelamis:0.05363824610238775853)58:0.02255642396191615690,((Euthynnus_affinis:0.00492586738206496691,Euthynnus_lineatus:0.00218688649550956770)100:0.01462176518740567442,Euthynnus_alletteratus:0.04961637797669862793)99:0.05983089109189129728)74:0.02750775623721487401)17:0.01639095823590731166,((((Thunnus_obesus:0.00303966084781087924,(Thunnus_tonggol:0.00379062154286306059,Thunnus_alalunga:0.02004156309325601584)31:0.00126704263722554871)29:0.00118192233948313883,Thunnus_atlanticus:0.00743983183189538683)13:0.00000176456401954691,(Thunnus_maccoyii:0.00221462351504741464,((Thunnus_thynnus:0.00000176456401954691,Thunnus_orientalis:0.00000176456401954691)95:0.02903168413455608707,Thunnus_thynnus_thynnus:0.00000176456401954691)49:0.00630437359526951104)82:0.00936735514331004347)28:0.00156434832741912644,Thunnus_albacares:0.00753196573174275564)99:0.09151375463575711633)18:0.03249066318814123472,(Cybiosarda_elegans:0.09438860894155710890,(Allothunnus_fallai:0.06255487868946041363,((Sarda_sarda:0.01330678789997650352,Sarda_chiliensis:0.00032934061346940946)99:0.02304171315476679499,Sarda_australis:0.04177054461221617332)99:0.05386269696639466725)28:0.01880870644228459579)39:0.01384802711429786171)15:0.03964783700123667570)14:0.02861373661653310654)5:0.01293475643944278983)3:0.01257281514050434187)0:0.01381179416565491563)1:0.02259283935616231490)2:0.00924449319683412311)0:0.00624316043977681933)10:0.06521392289913312268)1:0.05301806796698362717)0:0.00668916448387997954,(((((Synagrops_japonicus:0.33292835093691514059,Symphysanodon_berryi:0.19410310299784452881)9:0.09042805114856479476,((((Pseudopentaceros_richardsoni:0.02807596255014543907,(Pentaceros_decacanthus:0.01038637553244589791,Pseudopentaceros_pectoralis:0.00829510437080978325)72:0.01456901286687972523)93:0.05344970372184908169,((Histiopterus_typus:0.04382266580972728198,Evistias_acutirostris:0.03624662317005582918)93:0.04238665756895330927,(((Paristiopterus_gallipavo:0.00459974354434596576,Paristiopterus_labiosus:0.00000176456401954691)100:0.06285232314078734173,Pentaceropsis_recurvirostris:0.05565132722981717267)97:0.03437656179205365176,Zanclistius_elevatus:0.05642924488333834337)97:0.04624178795879677756)89:0.04011407787648149648)75:0.05030222888870127668,(Stereolepis_gigas:0.08721778540370295207,(Polyprion_oxygeneios:0.01502146058859401244,Polyprion_americanus:0.03273816336910226332)97:0.07524645677621903672)60:0.03418515720555629495)72:0.07066219445592877846,((Lachnolaimus_maximus:0.28378935237831337535,(Scombrops_gilberti:0.02261924437813672001,Scombrops_boops:0.04937278768120959699)93:0.07229348508372369919)6:0.05344562236772137931,((Acropoma_hanedai:0.19899801101800188019,Acropoma_japonicum:0.31605774034652195370)65:0.15238274711407648332,(Epigonus_angustifrons:0.06009303163240142104,Epigonus_telescopus:0.06087094527009429684)94:0.11701861354779480306)13:0.03093453954958585994)2:0.03289935923830694664)0:0.01697046307963259726)0:0.03172514573422401812,(Lateolabrax_maculatus:0.03544664076663584656,Lateolabrax_japonicus:0.08389005293970934474)100:0.29804115703549988181)0:0.02109757578466945477,(((Medialuna_californiensis:0.15534299697045136912,(Scorpis_lineolata:0.06725646473214234866,Scorpis_aequipinnis:0.02504502627549860458)95:0.05110123929892659150)92:0.10114788487535232575,(Atypichthys_strigatus:0.11367419115925390949,Microcanthus_strigatus:0.18276772454319847871)57:0.03096993748825040485)53:0.02396305434834010428,((Oplegnathus_woodwardi:0.09715167337669460002,Oplegnathus_fasciatus:0.23231115883823622026)62:0.07177803538210036916,((Kyphosus_vaigiensis:0.04248251830889274161,Kyphosus_bigibbus:0.03285471754541506584)99:0.06649250969381365062,Hermosilla_azurea:0.10072604936178711221)85:0.05601402309577059591)23:0.02400140902524247263)44:0.06114240517016884813)0:0.02013044580431291863,((Niphon_spinosus:0.12400306475232589221,((Acanthistius_joanae:0.15953544231952229704,((Pseudogramma_gregoryi:0.34961144847583192075,Grammistes_sexlineatus:0.17471912803652062118)45:0.07229772467172114470,Acanthistius_patachonicus:0.19067919431953869736)24:0.05992686998802774362)17:0.00922786779624865106,Howella_brodiei:0.29978295376512259507)1:0.03487995582903118813)2:0.05974150346133953043,(((((Terapon_theraps:0.37231385843951581016,(Leiopotherapon_unicolor:0.06034637310400935845,Leiopotherapon_plumbeus:0.06612377207490710562)93:0.08993001965626379324)23:0.09018726597905807330,Terapon_jarbua:0.29059606994274966851)11:0.03301738534544385734,(Rhynchopelates_oxyrhynchus:0.24125353185992076677,(Hephaestus_jenkinsi:0.05424092473515625124,Bidyanus_bidyanus:0.10230999373650942474)94:0.11393284347502610765)14:0.01442324952539438508)22:0.11694361580670421807,((Coreoperca_whiteheadi:0.16942565599622380623,(Coreoperca_kawamebari:0.13614806599833670364,Coreoperca_herzi:0.13828380888474922639)70:0.04341786896339890761)94:0.16003084934247935323,((Macquaria_colonorum:0.04162302392263301370,Macquaria_novemaculeata:0.04487248748035925494)100:0.28180787122725081684,(Nemadactylus_bergi:0.04343202231633654792,(Chirodactylus_grandis:0.11015868039080770058,Cheilodactylus_zonatus:0.18078842262789912576)82:0.07005579657352234424)91:0.09280045831718164440)27:0.05921903268789330960)7:0.04377641890652922418)1:0.01886545858483212879,(((Nannoperca_australis:0.09063059092722387800,Nannoperca_oxleyana:0.16548640463050995342)95:0.24229724281668887920,(Maccullochella_peelii:0.58951438331911754087,Macquaria_ambigua:0.23712910626889310839)14:0.00000176456401954691)2:0.10592802041691995463,(Siniperca_roulei:0.06499773897195713912,((Siniperca_chuatsi:0.03612890372398051136,Siniperca_obscura:0.05674333656978111651)34:0.00936037044457613092,(Siniperca_scherzeri:0.05045686818132222307,(Siniperca_undulata:0.04177364738211038209,Siniperca_fortis:0.02354089216768965806)40:0.00527846615984635045)64:0.01489673482229031398)38:0.01434897872786110307)69:0.08124594366312923310)1:0.10515663817527813595)0:0.00000176456401954691)0:0.03749765413553529658)0:0.03091320020325661472)0:0.00805554749190439540,(Pristolepis_fasciata:0.31003538261774354901,((Elacatinus_multifasciatus:0.64863413066314212774,(Selenotoca_multifasciata:0.25000471434939636195,Scatophagus_argus:0.11211251690732314257)98:0.23371689366345804739)3:0.09090602526495908386,(Badis_badis:0.60046682017889918548,Hemanthias_signifer:0.27302257662003531768)6:0.05016420150119887261)0:0.00936971971926667084)1:0.02505103007591632028)0:0.00000176456401954691,((Antigonia_rubescens:0.33885422062564724000,((((Caulolatilus_affinis:0.11002484014387202416,Caulolatilus_princeps:0.07631230961152102310)98:0.15439377821628927356,Hoplolatilus_chlupatyi:0.30632541462870149784)68:0.01951384521121664328,Malacanthus_brevirostris:0.41218572655149998685)31:0.11656435472515556950,((Lopholatilus_villarii:0.21491118113910753773,(Branchiostegus_doliatus:0.14926327261958172166,(Branchiostegus_albus:0.04997151260565533409,(Branchiostegus_argentatus:0.04412032821637003333,(Branchiostegus_japonicus:0.00210717082003001027,Branchiostegus_auratus:0.02043188783404998113)86:0.02793521558905288915)59:0.01908243593968190002)82:0.05408600589648565604)93:0.06714963257072839586)91:0.08807465051845310422,(Hoplolatilus_marcosi:0.06371637091723367230,Hoplolatilus_purpureus:0.04646864203830664702)100:0.32340154442338397978)41:0.05241677054551319159)11:0.10609570475098141495)0:0.01583553287352959874,((((((((Pomacentrus_trichourus:0.12314589665440359156,Pomacentrus_moluccensis:0.07257625354559242570)79:0.06077892980265722084,(Pomacentrus_nagasakiensis:0.20380724460896121331,(Pomacentrus_alleni:0.02758847605448908405,(Pomacentrus_coelestis:0.02753994738750275770,Pomacentrus_caeruleus:0.02107724997324587207)89:0.02078724676967086057)99:0.08949482313266150790)25:0.02317779415630199027)33:0.01994721234467214041,(((Pseudambassis_ranga:0.83221924796899748866,Premnas_biaculeatus:0.08197315651225041411)0:0.05323021688176324262,(((Amphiprion_melanopus:0.00172089268828272803,Amphiprion_frenatus:0.03561894653279038675)83:0.01213954527435829808,((Amphiprion_chrysopterus:0.03129991800497840954,(Amphiprion_clarkii:0.00844199403156960064,Amphiprion_sebae:0.01295813910823382688)95:0.04360168595375256417)44:0.00307064753510158080,((Amphiprion_latifasciatus:0.01344098998016442573,(Amphiprion_sandaracinos:0.01704202252980518972,(Amphiprion_chrysogaster:0.00512466498376008047,Amphiprion_polymnus:0.02395612234132703244)77:0.01000142775736809903)44:0.00874021870057896215)36:0.01398651639936452674,(Amphiprion_akallopisos:0.00445987881844010954,Amphiprion_perideraion:0.00723366895781379481)77:0.01987852852580535359)9:0.00000176456401954691)23:0.00705694149067136439)45:0.01658737788572406738,Amphiprion_nigripes:0.01131308519603224823)98:0.08087983861580982925)0:0.06794536230977211988,(Amblyglyphidodon_leucogaster:0.15255829364837919515,(Neoglyphidodon_oxyodon:0.12574053306108343153,(Neoglyphidodon_nigroris:0.07921562135250795333,(((Cirrhilabrus_flavidorsalis:0.09987942304127883641,Cirrhilabrus_lubbocki:0.00000176456401954691)100:0.22037864299041548533,Cirrhilabrus_exquisitus:0.00000176456401954691)99:0.26453965741139645607,Neoglyphidodon_melas:0.01110733736145226060)57:0.07421619854878250266)46:0.02679005948678848947)48:0.04003330479974839123)55:0.04290342902304659228)0:0.06203714724374962547)0:0.01976996500801567885,(Chrysiptera_talboti:0.21314576983103247776,((Dischistodus_pseudochrysopoecilus:0.05814919448821190967,Dischistodus_prosopotaenia:0.10757011579675516966)100:0.11634847042151662266,(((Chrysiptera_galba:0.08413671414280742000,Chrysiptera_rex:0.03876546977371632396)100:0.15680926750896440058,(Chrysiptera_taupou:0.03887876583988368606,Chrysiptera_cyanea:0.04479176882848828756)100:0.09559544233906232258)98:0.12260169424014356887,Chrysiptera_parasema:0.17272320517423628283)27:0.03582375529142126092)13:0.00961206017613375728)20:0.04951075389748183647)0:0.02937179826367008334,Chrysiptera_starcki:0.18463859772574109375)0:0.04475682920972352818,Chromis_dasygenys:0.27805282391450392154)0:0.09593384058468423792,(((Abudefduf_septemfasciatus:0.10110770738416581527,Abudefduf_saxatilis:0.03920219477078894232)98:0.18111286859121661363,Abudefduf_notatus:0.13731303620104348684)83:0.12124733415532849834,(((Chromis_viridis:0.03946936390509642872,(Chromis_atripectoralis:0.06983966038810476840,Chromis_caerulea:0.04559289439500961716)63:0.01416560003286292634)97:0.09065851648115517969,(Chromis_notata_notata:0.14359964296360208658,(((Chromis_cyanea:0.19980610493047179510,(Chromis_margaritifer:0.24525485195398874505,((Dascyllus_flavicaudus:0.05189980566909723619,(Dascyllus_trimaculatus:0.03792658197191747099,Dascyllus_reticulatus:0.04732043013309798762)95:0.03961976199458538961)90:0.08963597660719901938,(Dascyllus_melanurus:0.09310574579237063109,Dascyllus_aruanus:0.07897058623976577207)80:0.05633551059767180624)90:0.10480526668524428602)76:0.09291008869612576992)21:0.04407785193364856247,((Azurina_hirundo:0.11602338322309767304,Chromis_multilineata:0.04786182969089475964)97:0.10716773048258045054,Chromis_brevirostris:0.14304141253562585590)32:0.02634130774283781340)19:0.02839073437679777087,(Chromis_punctipinnis:0.12757331675253458236,(Chromis_alta:0.04537854492836049941,(Chromis_insolata:0.05364829547580109159,(((Chromis_abyssus:0.00390411127866798716,Chromis_circumaurea:0.00305180369695494868)99:0.04682989740541256690,Chromis_degruyi:0.07859860247635690189)93:0.06964000059512631680,(((Chromis_woodsi:0.05753081398892142401,Chromis_agilis:0.10092014405126216448)27:0.01159479117208524375,Chromis_opercularis:0.10024470736996399234)79:0.06504414376115190410,Chromis_cadenati:0.14153558553976353096)27:0.00705125607445437983)24:0.01118302405603873763)13:0.00950447509783704583)23:0.02371378328377290665)79:0.06445131671608372859)15:0.02794786404124901744)13:0.01670324142324505420)40:0.06655684901914996066,(((Stegastes_leucostictus:0.24125836703967395991,(Stegastes_planifrons:0.08859718239533834194,Stegastes_partitus:0.22822456112314545851)36:0.01168780449383659790)44:0.02195404098139842886,(Stegastes_adustus:0.03720214836621452831,Stegastes_diencaeus:0.06318916121894654903)93:0.11713283124963715098)84:0.11104572495171913249,(Plectroglyphidodon_leucozonus:0.17207732589438543003,(Hypsypops_rubicundus:0.07436124472538621633,Microspathodon_chrysurus:0.17248656255335015342)92:0.08044926506083177609)42:0.03096224922287036524)41:0.05363579094495854460)11:0.02764619459326709411)37:0.04088142904434812624)0:0.07755004821285108041,(((Nemanthias_carberryi:0.28601912638269849642,((Pseudanthias_lori:0.49210218651298170967,Pseudanthias_pascalus:0.19808844637622843865)37:0.10548911887001517773,(((Pseudanthias_truncatus:0.19056644373810105453,Pseudanthias_cooperi:0.22979454557131070991)55:0.05728947967142508646,(Pseudanthias_pleurotaenia:0.11530283432232227292,Tosana_niwae:0.13858452637462798096)48:0.07269672574403995835)43:0.08985559778671188547,(Pseudanthias_squamipinnis:0.05763791837625877740,Pseudanthias_huchtii:0.12438171606468889641)67:0.06846363291720705679)26:0.04094350033082436863)36:0.09439372893960037980)5:0.07469792552356112336,((Lobotes_surinamensis:0.03932623228595035630,Lobotes_pacificus:0.08294633518504587744)100:0.51962632996291779897,((Girella_punctata:0.06503105065894709824,(Girella_nigricans:0.05951198354487513553,(Girella_tricuspidata:0.03900589009820562558,Girella_zebra:0.04808693431773736204)58:0.01925671126090588645)64:0.08059111844881332309)86:0.27806821613088755774,Serranocirrhitus_latus:0.17302172453648190231)14:0.03352795708780326872)0:0.00000176456401954691)0:0.02044072236177127064,((Caprodon_unicolor:0.01554654036886818531,Caprodon_longimanus:0.00000176456401954691)100:0.28398351024991497216,((Meganthias_natalensis:0.06952507793286798188,Odontanthias_elizabethae:0.28528221587046065366)72:0.08224355464275065475,Odontanthias_borbonius:0.05303863798414260033)96:0.15762904261606613954)19:0.07757432054104802799)0:0.04744952178975367363)0:0.02717716385732191556)0:0.02668270690357706434)0:0.00839355124561394147)0:0.02543283156555552899,(((Cirrhilabrus_scottorum:0.17334813740423690720,((Cirrhilabrus_cyanopleura:0.10638481811268626909,Cirrhilabrus_rubriventralis:0.09968822785713504775)86:0.11697573237496512732,Cirrhilabrus_rubrimarginatus:0.19158128088970730052)42:0.05511420229459040171)82:0.26570996313780104181,(((Pseudocheilinus_octotaenia:0.29438117754979509222,Pseudocheilinus_tetrataenia:0.19000562766618256383)34:0.07559988622479357767,Pseudocheilinus_hexataenia:0.26195931877308076263)16:0.03735224287393362763,Pseudocheilinus_evanidus:0.26035779128707059460)23:0.06155886155735450227)5:0.10674305675849297959,(Triacanthus_biaculeatus:0.33411869164223817474,(Lactoria_diaphana:0.15603463707156348117,(((Ostracion_cubicus:0.00000176456401954691,Ostracion_immaculatus:0.00000176456401954691)100:0.01184912524828791829,(Ostracion_rhinorhynchos:0.04355583079582552031,(Ostracion_meleagris:0.04425076158635307677,Ostracion_nasus:0.03772551174434149446)40:0.00399906084985144229)43:0.01289757837192880355)95:0.11518343585267103169,(Tetrosomus_gibbosus:0.10119486772709879174,((Lactophrys_trigonus:0.05219333241273600710,Lactophrys_bicaudalis:0.08085160688690468112)94:0.12142988974108462341,Acanthostracion_quadricornis:0.16261734096941823924)76:0.09768868924481066696)16:0.01825786414749994493)13:0.03514445306391837665)69:0.11711602386689549948)4:0.12535118600139033562)0:0.04399855643899066443)0:0.02053540356378997719)0:0.01467444504248127148,(((((((((Liza_melinoptera:0.11640330205242356565,((Valamugil_engeli:0.00341298584470493089,Liza_subviridis:0.00106050090816602028)100:0.04297753882259248981,Liza_haematocheila:0.08866595120181135214)89:0.01760205270232121505)93:0.04941798325884652621,((Liza_macrolepis:0.18413562756133450282,(Liza_carinata:0.02469400897466538305,Liza_parsia:0.05464325744967624826)97:0.11261232132182046051)32:0.01594407037210793243,((Liza_ramado:0.09902931383596101644,Liza_saliens:0.07201882585073431098)17:0.00000176456401954691,(Chelon_labrosus:0.04675640424587783339,Liza_aurata:0.05970861966193565656)26:0.00944899098390084360)84:0.10337007648562378248)82:0.06610950262849378534)86:0.07578709447382195319,(((Liza_tade:0.21674233955865279677,Valamugil_cunnesius:0.10902924565940677604)99:0.20046644293802937109,((Crenimugil_crenilabis:0.04552333644808027879,Valamugil_buchanani:0.09648116165967403346)97:0.08156698678254771517,Valamugil_seheli:0.02144811847079796871)96:0.07932960686786778848)67:0.05883494687490728459,Aldrichetta_forsteri:0.26897556098875297081)29:0.01356818027336703474)41:0.07746393708488495677,Agonostomus_monticola:0.31520281929685439737)50:0.05899319209605585757,((Mugil_trichodon:0.12728712607889283714,Mugil_curema:0.10855341111161555256)100:0.14176669112213050039,(Mugil_cephalus:0.05193874392083641656,(Mugil_liza:0.00000176456401954691,Mugil_platanus:0.00223261426301693154)98:0.00058529009116291264)100:0.27313343676383489145)68:0.06006697772944634417)42:0.09623557682227197596,((((Lethrinus_laticaudis:0.08155585837887431577,(((Lethrinus_lentjan:0.08246751313451858301,Lethrinus_ornatus:0.05166443633009675657)39:0.02141287532672837141,((Lethrinus_atkinsoni:0.02040776782105747228,Lethrinus_mahsena:0.01564605958471118607)94:0.07512465055915398815,Lethrinus_nebulosus:0.04729434451986279375)56:0.01821325851263387932)22:0.01435722064474914683,Lethrinus_harak:0.07325050657524340147)40:0.03162367632849057142)70:0.10896297170532295850,(((Lethrinus_conchyliatus:0.00435303577580861949,Lethrinus_rubrioperculatus:0.00000176456401954691)100:0.14134470464130582945,(Lethrinus_miniatus:0.03809737428702343609,Lethrinus_olivaceus:0.01570693706593839431)100:0.14442111533387497202)67:0.05189098697093376400,Lethrinus_semicinctus:0.19402389363496408414)41:0.04229281497080895902)20:0.05257004346537245271,Lethrinus_haematopterus:0.19693170309004531804)66:0.10056665762367972272,(((Secutor_megalolepis:0.08276756709831298298,Secutor_ruconius:0.09517457817590396774)98:0.08300658670877027190,Secutor_indicius:0.15316618603073051119)98:0.14610440426351645193,((((Leiognathus_daura:0.14668688593741358028,Leiognathus_dussumieri:0.15445265863976387344)66:0.10908038923661594888,(Eubleekeria_jonesi:0.05070069871954400315,(Nuchequula_decora:0.03386481878957523645,(Nuchequula_mannusella:0.05494909644707880736,Nuchequula_nuchalis:0.07668183446793176705)55:0.04364308850237725440)48:0.03228124833979999914)74:0.08080081177009880433)69:0.04975784451503156847,((Equulites_rivulatus:0.11925541250324880882,(Equulites_stercorarius:0.14628955373529603512,(Equulites_lineolatus:0.20720286685308073049,Equulites_elongatus:0.16444693872686680369)39:0.02352263871408552184)38:0.03981160622168633534)59:0.04652128779987901169,Equulites_leuciscus:0.09545929773429308318)94:0.08737339404994773351)85:0.09115569323972130822,(((Photopectoralis_bindus:0.07939764198928997430,((Secutor_insidiator:0.03033865754173684440,Photopectoralis_panayensis:0.01003044365628127585)100:0.08307438017249851869,Photopectoralis_aureus:0.09857280504582688341)89:0.02872956894496146371)96:0.10400378742688015654,(Leiognathus_philippinus:0.00000176456401954691,Eubleekeria_splendens:0.00000176456401954691)100:0.24298843118083848780)47:0.04838791119024796195,((Aurigequula_fasciata:0.16396767710898918491,(Leiognathus_equulus:0.07817874417621073491,Leiognathus_robustus:0.12127886874458838895)100:0.08358799979058330332)100:0.24503023825821704240,(Gazza_squamiventralis:0.31248584292130676676,(Gazza_achlamys:0.15288630334149291845,(Gazza_minuta:0.00000176456401954691,Leiognathus_brevirostris:0.00000176456401954691)100:0.26442008722127663400)81:0.06066570883450169566)59:0.05995479171473735991)42:0.05205568054891582253)18:0.05150278678638788715)40:0.04683508252091377055)98:0.20104378622334245774)2:0.06431427414493234851)0:0.02925772505866618828,(((((Nemipterus_japonicus:0.15298557477393451287,(Nemipterus_virgatus:0.10116762646225445355,((Nemipterus_hexodon:0.13379476703249512659,Nemipterus_mesoprion:0.05334856645333783953)65:0.04683633587381303559,Nemipterus_bipunctatus:0.15301345045168737724)46:0.02250123005468109005)96:0.12033049698994391363)86:0.09388845121158527485,(Nemipterus_furcosus:0.05701778492021641193,Nemipterus_peronii:0.10202517465594497514)100:0.16149168223348459028)99:0.12097404035743083983,(Parascolopsis_eriomma:0.24341992988336277892,(Scolopsis_ghanam:0.29515883465928360874,(((Scolopsis_monogramma:0.15824859656199111169,Scolopsis_taenioptera:0.12196820534433920613)28:0.00891542401064816629,Pristipomoides_multidens:0.12013701155595322978)97:0.16403796059487338321,Scolopsis_vosmeri:0.17907158961152430665)64:0.04988391392000746105)75:0.09840739546687995998)38:0.04619493193312622370)37:0.12921714778817611213,((((Oxyzygonectes_dovii:0.34610994494226027252,((((Profundulus_candalarius:0.00944463989212131552,Profundulus_punctatus:0.00471612135538939173)95:0.01168576023206470069,Profundulus_labialis:0.00000176456401954691)68:0.00000176456401954691,Profundulus_guatemalensis:0.00696022990336884161)99:0.28339171683145486513,((Empetrichthys_latos:0.03473391218413410947,Crenichthys_baileyi:0.07927951794063163582)78:0.05994621749228543073,((Characodon_audax:0.01327626276471253509,Characodon_lateralis:0.01724288297710445741)100:0.11702009617483111192,((Goodea_atripinnis:0.05908177293034497929,((Xenotoca_eiseni:0.01439774263626689838,Xenotoca_melanosoma:0.04077197720489685040)90:0.02605568964678297780,((((Alloophorus_robustus:0.06525585087915597438,Ameca_splendens:0.02515848057774872082)33:0.00765197190067400373,Xenotoca_variatus:0.04909667402152480958)32:0.00556395917152738718,(Chapalichthys_encaustus:0.00194738197946466929,Chapalichthys_pardalis:0.00735259626794320352)98:0.04555104155912582942)58:0.03012855170352839868,((Zoogoneticus_tequila:0.08331089204721152386,Zoogoneticus_quitzeoensis:0.10261288246414208836)67:0.03525356726359941961,Xenoophorus_captiva:0.04926578555376374957)9:0.00748023098477582082)35:0.00924239297535411376)54:0.03227080567244040160)46:0.02007534099180578735,(((((Skiffia_francesae:0.00231910906061279145,Skiffia_multipunctata:0.00233781343358441815)99:0.02940479500876891708,Skiffia_lermae:0.03536671130047436967)97:0.07928509202870687167,(Hubbsina_turneri:0.18990702754093469196,(Skiffia_bilineatus:0.08754927942750950765,(Allotoca_regalis:0.04418274461318227875,(Allotoca_dugesii:0.03234154247000552845,(Allotoca_goslinei:0.06861078996847436162,(Allotoca_maculata:0.09337486307380930828,(Allotoca_catarinae:0.00742271237723245419,Allotoca_diazi:0.01199550908703566308)100:0.02989088483396505971)34:0.01278637212232062656)41:0.00716414570338108972)88:0.07220580831991944837)71:0.03750596273161587585)26:0.01078774520521112897)15:0.00775211249825106564)24:0.01385880168666907448,(Girardinichthys_multiradiatus:0.05919819081470710509,Girardinichthys_viviparus:0.06489318056596916906)84:0.03743869767922346214)15:0.00821768878399579440,(((Allodontichthys_hubbsi:0.04995063470456236054,(Allodontichthys_zonistius:0.01218599569153716589,(Allodontichthys_tamazulae:0.02938547731225316442,Allodontichthys_polylepis:0.01165666178247137634)83:0.00604303444149837093)80:0.02548946939664700762)100:0.06505890910792058723,(Xenotaenia_resolanae:0.13573988626787722445,(Ilyodon_furcidens:0.00477616800095198926,Ilyodon_whitei:0.00718279368178395116)99:0.03271824875492947149)63:0.02112528993837673938)70:0.05169712410477017572,Ataeniobius_toweri:0.09019870915376669573)8:0.00306840294606485885)9:0.00407415550470210421)9:0.01859923019045327253)33:0.07040432221162402915)81:0.19230503705765791822)57:0.12268077006114086469)18:0.12710071755507953983,((Garmanella_pulchra:0.28516094541272263285,(Floridichthys_polyommus:0.02896966428961580964,Floridichthys_carpio:0.03202034820072418436)93:0.18464336367718001131)77:0.09604344296490006927,(Jordanella_floridae:0.22927537009488035968,(Cyprinodon_artifrons:0.04575080538727526597,Cyprinodon_variegatus:0.05192653946367265655)97:0.08255855130611089443)76:0.10326827973094315771)17:0.03611838743911589128)0:0.08287636794088058811,((((Iso_natalensis:0.34471076560219565676,(((Hypoatherina_valenciennei:0.11350663434278669695,Atherinomorus_endrachtensis:0.11090900690771014758)94:0.09114639560087420267,(Teramulus_waterloti:0.19684589181001091451,(Atherinomorus_stipes:0.06934830827381274765,((Atherinosoma_microstoma:0.24626660360884741752,Craterocephalus_honoriae:0.33063936690250461670)48:0.18583484315466769488,Atherinomorus_lacunosus:0.06588783468444246727)39:0.07074381128876498781)33:0.03406094607644528927)33:0.07575791174657552085)10:0.02578661737393616801,Alepidomus_evermanni:0.26154222367645407754)6:0.01037919362310722390)3:0.02626984851074564212,(((Pseudomugil_signifer:0.19521752635289160716,Pseudomugil_mellis:0.19417627555537858575)66:0.18033908320234531830,((Craterocephalus_marjoriae:0.13848954730117141176,(Craterocephalus_stramineus:0.19405516980693410245,Craterocephalus_stercusmuscarum:0.10039625112039536614)93:0.18540866571418371245)65:0.17848727006228920500,((((Menidia_beryllina:0.00000176456401954691,Menidia_menidia:0.00428454399876002784)99:0.13583331570480966510,(((Labidesthes_sicculus:0.23451324386792049559,((((Chromaphyosemion_bivittatum:0.00290099191635695593,Chirostoma_riojai:0.00000176456401954691)0:0.02108275063582653111,(Poblana_alchichica:0.00436578388719239749,(Poblana_letholepis:0.00000176456401954691,Poblana_squamata:0.00000176456401954691)92:0.00000176456401954691)97:0.00879397050776809137)1:0.02027181676827095880,(Chirostoma_labarcae:0.08106837412058431891,Chirostoma_jordani:0.03156892677605227515)83:0.01462702223358258204)0:0.04122914471673586045,((Chromaphyosemion_loennbergii:0.03697481299691571244,(Chromaphyosemion_punctulatum:0.02345598502558358597,(Chromaphyosemion_splendopleure:0.01486879640567480461,(Chromaphyosemion_volcanum:0.01580191060310393372,Chromaphyosemion_poliaki:0.00699190470425876259)97:0.04753610395828537782)81:0.01577310297679343151)62:0.00718257720852020533)83:0.04173163556953387449,(Chromaphyosemion_riggenbachi:0.08780662115052756400,(Chromaphyosemion_kouamense:0.04542950057694108285,(Chromaphyosemion_melanogaster:0.02949066287756179142,Chromaphyosemion_malumbresi:0.04679840269399002106)66:0.00413906226044721739)78:0.02759977613420816836)35:0.02117893014108960387)40:0.03258103570239224284)0:0.00000176456401954691)0:0.01630202933732970844,(Chromaphyosemion_alpha:0.28731458167960416406,(Chromaphyosemion_lugens:0.07114219626117400386,Aphyosemion_lividum:0.42971454641448014744)20:0.00863364618627821684)51:0.07607390264364009835)0:0.01178154009633259822,Chromaphyosemion_bitaeniatum:0.08562482842024464469)0:0.02961915560937534650)0:0.08247739458323835593,Atherinella_panamensis:0.33098510903203098854)0:0.10079204521627120605,((Atherina_hepsetus:0.10030374846158712776,Atherina_boyeri:0.18668278078561728606)96:0.10069812318842503995,Atherina_breviceps:0.24170827237984379710)68:0.12927443468455293574)0:0.02988349544421164267)0:0.04626627169366427650)0:0.00938396171117844569,(((((Melanotaenia_fluviatilis:0.02018480318350845959,Melanotaenia_duboulayi:0.02328514595996242220)96:0.07470477379033579890,Melanotaenia_splendida:0.04039855040601753045)90:0.07611864717219994636,(Melanotaenia_praecox:0.09303684223442290624,(Glossolepis_incisus:0.11109786125743677243,Chilatherina_bleheri:0.05468278562136476284)58:0.00862519788772183812)97:0.09767529312532541308)84:0.08091549124890083655,Iriatherina_werneri:0.27536423862764625170)77:0.09725527926916147092,((Rheocles_wrightae:0.03485069624895089507,(Rheocles_alaotrensis:0.01547295532033868617,Rheocles_lateralis:0.00870135782420007782)100:0.07908503939244852787)96:0.10260534973612257803,(((Bedotia_geayi:0.05165798923440483026,Bedotia_madagascariensis:0.02933966562005772133)49:0.01250555210966321044,(Bedotia_marojejy:0.03445600365269848447,(Bedotia_masoala:0.01693613849178552538,Bedotia_longianalis:0.03003102065212245875)93:0.04980544117542155813)43:0.01042851994840414930)48:0.03309803723316444291,(Rheocles_derhami:0.01450393740028705150,Rheocles_vatosoa:0.04867317346412843720)100:0.13196282323466315689)70:0.08347900647286676801)87:0.15482187331698440969)29:0.07655116317940942328)0:0.01862279033283214050)0:0.04132414833472373289,(((Plectropomus_laevis:0.02061638462678193687,(Plectropomus_leopardus:0.01267240538139711645,Plectropomus_maculatus:0.06606362894997318269)100:0.13446129826801414553)95:0.29332708618365094244,(((Strongylura_notata:0.18911505429282554624,Cololabis_saira:0.24954507851991386547)49:0.07503739527619966709,(((((Nothobranchius_furzeri:0.09155518678975199665,(Nothobranchius_orthonotus:0.07229667625148855636,Nothobranchius_kuhntae:0.06191977930406024344)56:0.06451362453039256872)81:0.12188926720549492222,((Nothobranchius_eggersi:0.12505077831628200191,((Nothobranchius_foerschi:0.11602950238568339458,Nothobranchius_cardinalis:0.13331276341107040073)57:0.02785894208283689819,Nothobranchius_kilomberoensis:0.24242474116750159507)30:0.01422582879211532411)23:0.00862878292599458460,Nothobranchius_guentheri:0.17694054057608774033)61:0.07386130001850808868)12:0.01560117621437149063,(Strongylura_strongylura:0.24818711147677741113,((Ablennes_hians:0.11794285067896849439,(Tylosurus_crocodilus_crocodilus:0.00000176456401954691,Tylosurus_crocodilus:0.00000176456401954691)99:0.08663058436809618901)59:0.07248447515810725605,(Strongylura_leiura:0.17199174298398961502,Nothobranchius_kirki:0.14083843103944479469)4:0.03571628833942123993)1:0.05730888824814442395)3:0.01069323043097171119)0:0.01330792966884922386,(Nothobranchius_rachovii:0.31547644638358213198,((Nothobranchius_krammeri:0.23892944159786122515,Nothobranchius_hengstleri:0.13017790004030940776)62:0.05826660721828991890,Nothobranchius_melanospilus:0.13492030688097472746)72:0.08702683390345676562)30:0.03442664370680324076)1:0.02737571728868100351,Strongylura_timucu:0.22364295283714777374)0:0.02530295611607930387)2:0.03535596131076580717,Pronothobranchius_kiyawensis:0.31897800979595569704)4:0.05440205930040074855)1:0.10358819858788222146,((((Hyporhamphus_xanthopterus:0.16834990490063525548,Hyporhamphus_affinis:0.04576826145338391827)88:0.06018446337525092810,(Chriodorus_atherinoides:0.33277948531623435802,(Hyporhamphus_quoyi:0.14404242752053966758,Hyporhamphus_limbatus:0.09883820727925624050)79:0.08211085197222754706)13:0.01700818508195095502)17:0.05204575167841420102,Hyporhamphus_unifasciatus:0.20129797886935968387)6:0.03541878034007613085,(((Hemiramphus_archipelagicus:0.05255145510689089583,Hemiramphus_far:0.04655789247951781090)90:0.14453328668320525319,(Parexocoetus_mento:0.18749931025747040847,(Fodiator_acutus:0.21603776997605791954,((Cheilopogon_pinnatibarbatus:0.08228356431766335743,(Hirundichthys_oxycephalus:0.00651043343343355937,Hirundichthys_rondeletii:0.02006113808500782636)97:0.04932605286407053952)84:0.05032848029109592003,Exocoetus_monocirrhus:0.07711650212539705729)83:0.08496029200822825811)47:0.03172528080351915553)51:0.05244754675854018666)18:0.02043548829174133760,Hyporhamphus_dussumieri:0.18482177174937794439)44:0.07636872962205423776)3:0.04161141325497783733)0:0.03531565256275054354)0:0.01181134361630245891,(((Leuresthes_tenuis:0.11231502936025593298,Atherinops_affinis:0.04687010131851106209)88:0.06336578828571072519,(Basilichthys_microlepidotus:0.01842403066349667870,Basilichthys_semotilus:0.02634923218835461683)100:0.12721993779392687385)67:0.04300938819429607485,(Odontesthes_incisa:0.00487695177643334126,((Odontesthes_argentinensis:0.00217431811642065497,Odontesthes_bonariensis:0.00448625688982139028)99:0.02599405520687501220,(Odontesthes_platensis:0.01985977563512420982,((Odontesthes_smitti:0.00459312536227014095,Odontesthes_regia:0.00446257964095247983)77:0.00440288730447978890,Odontesthes_hatcheri:0.00697341163510248330)78:0.00871807848944386801)25:0.00120370683365485557)93:0.04795598947174359306)93:0.10160390551152941041)80:0.18588517208167895656)0:0.00817063393491511934)0:0.02214556635111274499,((((Paretroplus_damii:0.06200062850693421890,(Paretroplus_tsimoly:0.01145527555964188232,Paretroplus_nourissati:0.00000176456401954691)89:0.04673629302560669552)84:0.07582898254759184997,(Paretroplus_polyactis:0.05646858375187794460,(Paretroplus_kieneri:0.11511758425791326910,((Paretroplus_menarambo:0.02195318430607677215,Paretroplus_maromandia:0.01804292685642822774)10:0.00000176456401954691,Paretroplus_dambabe:0.03238796712481515488)75:0.06124472258528319835)52:0.03461917530534543846)65:0.07574608768885277010)91:0.18097457277068726422,(Etroplus_suratensis:0.11469271014703864842,Etroplus_canarensis:0.42760622873925729914)53:0.27986099942451148070)3:0.12886494222635619389,((Heterochromis_multidens:0.32406210799004669942,(((Oxylapia_polli:0.18992727798115147109,(Ptychochromoides_vondrozo:0.00288465919503396069,Ptychochromoides_betsileanus:0.00646399152875491191)100:0.10277061887032713783)98:0.12306097102135454824,(Ptychochromis_grandidieri:0.07883267647603094119,(Ptychochromis_inornatus:0.04151067578885727499,Ptychochromis_oligacanthus:0.03820280195560446512)91:0.02564911808905331533)87:0.15640651684641818098)75:0.17396339900840618320,(((Tylochromis_sudanensis:0.26820358188018134094,(Chromidotilapia_guntheri:0.21892019638751464283,(Etia_nguti:0.17445117493273284981,((Tilapia_dageti:0.01041450595746185905,Tilapia_mariae:0.01018129742186268949)94:0.09379449660608445150,(((Maylandia_zebra:0.02554872558592807819,((Astatotilapia_flaviijosephi:0.05200170091203139278,(Haplochromis_simpsoni:0.00228425830976344574,Astatoreochromis_alluaudi:0.00000176456401954691)100:0.03731021349800801162)66:0.01049310168287374140,Haplochromis_burtoni:0.03399972213323304571)43:0.01098961733976213870)98:0.06753127316135584812,Chalinochromis_popelini:0.12755935310648847114)87:0.03980495997555964782,((((((Sarotherodon_lohbergeri:0.00600865363001727021,((Sarotherodon_galilaeus:0.00476222147477597772,Sarotherodon_galilaeus_boulengeri:0.00232520790059628779)84:0.00285486231235917683,Oreochromis_aureus:0.00435149267386127688)69:0.00453853779418870052)90:0.03037133050674490839,Sarotherodon_melanotheron:0.05510990819656906570)42:0.00538335160236841446,Tristramella_simonis:0.05948721801270339532)67:0.02011231574413680639,(Oreochromis_niloticus:0.04931414899602128143,((Oreochromis_mossambicus:0.03417811770227966589,Oreochromis_urolepis_hornorum:0.03555258771572141624)50:0.00714626645776445836,Oreochromis_esculentus:0.04265168457145226966)47:0.01557687972234416719)70:0.03941385798784595440)25:0.00409362411061576538,Steatocranus_tinanti:0.23088685469626613056)37:0.00702645479242495433,(Tilapia_guineensis:0.02443029643782103008,Tilapia_zillii:0.03699496863337760344)99:0.13337717517469457884)18:0.00930483028992679269)18:0.01665406077582666858)57:0.05902293463663662448)41:0.06685843112580061587)20:0.00904131534876030825)32:0.02383937321446876617,((Hemichromis_fasciatus:0.14975055169109327546,(Hemichromis_letourneuxi:0.00301649358411759183,Hemichromis_guttatus:0.01119314796572949862)100:0.24666948405420077717)85:0.10305818528367109710,Pelvicachromis_pulcher:0.70239895451934042381)5:0.00000176456401954691)35:0.02792452296602500328,Gobiocichla_ethelwynnae:0.26321503022493419888)45:0.06092219174792128794)9:0.02402204182506510743)11:0.02356287071990405269,(Retroculus_xinguensis:0.44409993866975766563,((((((Australoheros_facetus:0.17691295290946248975,Aequidens_tetramerus:0.09250981061668325789)70:0.05831882523262879592,Aequidens_diadema:0.12212198394091759723)85:0.09916278012834275901,((Acarichthys_heckelii:0.28774649132017876552,(((Crenicara_punctulatum:0.46841932158719284951,(Apistogramma_bitaeniata:0.68922227221560095067,(Crenicichla_lacustris:0.14391427379495835837,(Teleocichla_monogramma:0.39995267862780342627,((Crenicichla_alta:0.10769305853418704999,Crenicichla_lepidota:0.11342972190259237308)80:0.12609523151766383053,Crenicichla_acutirostris:0.12581341790345765275)65:0.03412306186449257789)64:0.15425195738899238895)100:0.32499198452404842730)45:0.12187513227983096109)15:0.07660865262191333958,(Biotoecus_dicentrarchus:0.56684687384448306791,(Apistogrammoides_pucallpaensis:0.38310449068399382577,Taeniacara_candidi:0.48310740167287485880)80:0.32010765717587291723)17:0.06688002337908978412)7:0.01670095178525616078,Satanoperca_leucosticta:0.28279183528730822417)1:0.01128529967059082109)3:0.02658549848401158683,(Mikrogeophagus_altispinosus:0.40991346029005093499,((Geophagus_surinamensis:0.19126170749670304283,Geophagus_steindachneri:0.19553979133831822512)52:0.01635539070071802270,(Gymnogeophagus_gymnogenys:0.26977525370285704298,(Geophagus_proximus:0.02226393938752287138,Geophagus_brasiliensis:0.00345724994052590317)100:0.09845533545884230242)73:0.07411686905087681854)33:0.02368721162280947728)11:0.04878236570554109197)18:0.02940564076644687497)18:0.01354619388039467361,(((Chaetobranchus_semifasciatus:0.11928318730763405597,Chaetobranchus_flavescens:0.16185244624047967488)57:0.01415958208549985588,Chaetobranchopsis_orbicularis:0.19136530258057984910)98:0.20397579510568544792,(Pterophyllum_scalare:0.28815641302349803698,(Hoplarchus_psittacus:0.19227237130799004761,(Symphysodon_discus:0.12114990306882905680,((Mesonauta_festivus:0.15276445616062686583,(Uaru_amphiacanthoides:0.08356466998346166741,(Heros_severus:0.00000176456401954691,Heros_appendiculatus:0.00000176456401954691)100:0.11166898628745704214)48:0.01027346434533586185)28:0.01242820493050433850,((((Caquetaia_myersi:0.10221560218397601760,(Caquetaia_kraussii:0.11664838812674060975,Caquetaia_spectabilis:0.08171524564144680425)69:0.03628744496742151299)57:0.04143029373031818091,((Theraps_wesseli:0.09195627194469382926,(((Cryptoheros_spilurus:0.10427527247060329463,((Cryptoheros_sajica:0.06997178363612063801,((Parachromis_friedrichsthalii:0.02347508586841583894,(Parachromis_motaguensis:0.01021578019823419191,Parachromis_loisellei:0.00000176456401954691)78:0.01290421603512388431)92:0.02110638573910447388,(Parachromis_dovii:0.02843646054129226816,Parachromis_managuensis:0.03323329755150911807)78:0.02346766977315870376)38:0.00000176456401954691)16:0.00341842636950766277,Hypsophrys_nicaraguensis:0.09473508575392990128)4:0.00162502206233884876)12:0.00687252172195145731,((((Amphilophus_calobrensis:0.06361528245397285453,(Petenia_splendida:0.01436043747455528936,Cichlasoma_urophthalmum:0.03462132880655970080)100:0.03682576953366650113)84:0.03255905872133486867,((Amphilophus_citrinellus:0.02563198726235490360,Archocentrus_centrarchus:0.03940376070827325550)73:0.00575592492150634859,(Cichlasoma_trimaculatum:0.04006616139594245535,Amphilophus_lyonsi:0.02842035305035176679)82:0.01437717557654734010)67:0.01260856516089507544)6:0.00309452657845824463,Cryptoheros_panamensis:0.07809609614633283847)4:0.00000176456401954691,Cryptoheros_myrnae:0.05912512487319389332)3:0.00367273241508190001)0:0.00000176456401954691,(Amatitlania_nigrofasciata:0.05720672953487763307,(Vieja_tuyrensis:0.07210634507304009011,Tomocichla_sieboldii:0.05914872031927437268)51:0.02248038851395885163)25:0.01644973554077891129)7:0.01273756547299418479)19:0.00730727247018030622,Caquetaia_umbrifera:0.09799315462995396298)39:0.01242694126815923998)42:0.02822427874529486597,((Nandopsis_ramsdeni:0.03253229682314202204,Nandopsis_tetracanthus:0.02412948290560255199)99:0.08691032656271065193,Nandopsis_haitiensis:0.13930428538304850949)43:0.04107220878424557020)27:0.01798861448630532586,(((Amphilophus_robertsoni:0.12601659565508721572,Rocio_octofasciata:0.11967696881187313285)18:0.00160589638410183963,Archocentrus_spinosissimus:0.13590616063236507505)39:0.03329214625656833765,(((Cichlasoma_grammodes:0.10020949420179990630,((Cichlasoma_salvini:0.16592192858716947601,((Thorichthys_helleri:0.03326389084667031454,(Thorichthys_pasionis:0.03645415496721559484,Thorichthys_meeki:0.01304814458279145856)88:0.01538342529084519780)99:0.04059009449389040097,(Thorichthys_aureus:0.04110436171729579119,Thorichthys_ellioti:0.03682113687421248449)100:0.08659702951600944942)99:0.06015760718671767093)37:0.03335717128157472677,Tomocichla_asfraci:0.10262470615738177038)6:0.00441782656339154492)2:0.01112434489776664356,(((Vieja_intermedia:0.02392321941764909490,((Cichlasoma_ufermanni:0.05384216259417549943,Vieja_godmanni:0.00502228824841435394)51:0.01119340498580079744,((Vieja_heterospila:0.00000176456401954691,Vieja_maculicauda:0.00000176456401954691)100:0.09082457113831721163,Theraps_lentiginosus:0.08166953088077896772)15:0.00491660867100157228)21:0.00762062001662847273)34:0.01936210495051907815,((Herichthys_labridens:0.06227806898906478039,(Herichthys_deppii:0.00470066975124555121,(Herichthys_cyanoguttatus:0.00479390800981081486,(Herichthys_carpintis:0.00000176456401954691,Herichthys_tamasopoensis:0.00247708212281350527)68:0.00252234589662923446)71:0.00765382761551226722)100:0.06493799699166796779)61:0.03249953269642444353,(((Vieja_argentea:0.02279950155586940932,Vieja_regani:0.00844585841035650452)100:0.03860916599147163480,(Vieja_bifasciata:0.01781773571904735759,(Vieja_melanura:0.00245286054651837641,Vieja_synspila:0.00249485718679806666)78:0.00000176456401954691)69:0.01292829193333622229)80:0.01369719520179393107,Vieja_fenestrata:0.02990782399110795964)98:0.04125689898449921844)21:0.01052490322000297802)37:0.02425720346112918840,Archocentrus_multispinosus:0.12680658541928849226)15:0.01642074595849354890)9:0.01399668635982694379,Cichlasoma_festae:0.09600640411218909487)24:0.03079466749871967446)7:0.01611507947471615618)23:0.04690861370247560724)24:0.02478243711415642481)47:0.03824321827965269927)47:0.02093392773630395332)59:0.06639250248751540906)11:0.00976051238992666978)10:0.03447774577393054179,((Cleithracara_maronii:0.39850552448504039660,Cichla_temensis:0.19218392005581619308)18:0.08365557948044620440,(((Laetacara_thayeri:0.17663829848036086134,(Bujurquina_vittata:0.13929568705391728867,Tahuantinsuyoa_macantzatza:0.09330566178238178243)98:0.12078575555196970071)56:0.05204398252351385978,(Andinoacara_pulcher:0.12110345833239823110,Andinoacara_rivulatus:0.15930154093864634657)98:0.12798736918355452241)39:0.01316612329592225031,Acaronia_nassa:0.14081288705851041643)64:0.06564209855850482167)5:0.02728182151346211434)7:0.02340170889927111267,(Biotodoma_wavrini:0.86565225578570625942,Nannacara_taenia:0.23680460737851985464)52:0.21720124709587571021)46:0.03813806367296997124)25:0.06208004579434268921)42:0.04090010926576229805)0:0.06061308314649803408)0:0.00623458467223462522)0:0.00713544322513221668,(((((Fundulus_parvipinnis:0.28756215681253138872,(Fundulus_similis:0.31807444247820998440,((Fundulus_waccamensis:0.00628586278474282337,Fundulus_diaphanus:0.00322624854822355133)89:0.06305541320921546899,(((Fundulus_heteroclitus:0.07067123206765370258,Fundulus_grandis:0.06290588461078713833)95:0.11160305985291560371,(Fundulus_catenatus:0.04585673757389256699,Fundulus_stellifer:0.04335768441424611708)93:0.05623425047785281239)21:0.01753421347797988125,((Fundulus_sciadicus:0.10826546458789108873,(Fundulus_kansae:0.02656264490887842375,Fundulus_zebrinus:0.08446114471871152274)97:0.09405469130979708658)76:0.04819848804965611022,(((Adinia_xenica:0.16528085427642913441,Fundulus_chrysotus:0.15913388386039056810)80:0.06239058900116613987,((Fundulus_dispar:0.02539878704487397512,Fundulus_blairae:0.03262802424077446833)100:0.14114702475362381207,Fundulus_lineolatus:0.07512929392221304181)59:0.04207499150048595732)30:0.02449994214330178388,(Fundulus_notatus:0.01967919598858184488,(Fundulus_euryzonus:0.00543363295630499080,Fundulus_olivaceus:0.00559060804742658214)99:0.05559514581115713311)98:0.16308336632255995835)50:0.04254504990142170956)63:0.11713405372255898673)14:0.04133101483232182877)9:0.03620202369866156594)10:0.10755677487648057644)21:0.09763095302226527938,Leptolucania_ommata:0.29828600937008470373)21:0.13917409766189228759,(Hyporhamphus_sajori:0.30470778470710707664,((((Rivulus_rectocaudatus:0.35094265155704085224,((Rivulus_xiphidius:0.20262174531086804552,Rivulus_frenatus:0.20974873520180165043)51:0.12015522894805218124,(Rivulus_agilae:0.04487396487725339722,(Rivulus_strigatus:0.01990580555241640640,Rivulus_geayi:0.03156052027510063140)93:0.06638635903361977952)86:0.12104706392392836078)29:0.04334175576444531525)18:0.08317272744932532880,((((Rachovia_maculipinnis:0.23272215939362517201,(Terranatos_dolichopterus:0.37419961786706679163,(Austrofundulus_limnaeus:0.33008366464807992191,Renova_oscari:0.32644294518931954441)22:0.09485500260332847822)25:0.03084101337210521862)33:0.14394853923532588413,(Trigonectes_rubromarginatus:0.29676649386631576144,((Aphyolebias_peruensis:0.22405495332732947023,Moema_piriana:0.18676797744563525261)15:0.04050297697253743556,(Moema_staecki:0.28428336711543034099,((Plesiolebias_aruana:0.22057199251970072140,(Papiliolebias_bitteri:0.22359460558518981510,(Maratecoara_lacortei:0.26999840864168533905,Pituna_poranga:0.44144617996791568615)35:0.10437903490530525685)19:0.11366435368577618159)40:0.22505150684545416340,(Neofundulus_paraguayensis:0.36620456249783922553,((Rivulus_insulaepinorum:0.00947727510840588178,Rivulus_cylindraceus:0.01910310858340302786)95:0.00000176456401954691,(Pterolebias_phasianus:0.07083530395357125198,Pterolebias_longipinnis:0.14203082888082049773)77:0.07073845738665240912)0:0.09095290058282663737)0:0.06367550761169582518)0:0.04158554999941688202)0:0.03380066496249537616)0:0.04294476075302969958)0:0.06873953671080150485)0:0.04613410957199275131,((((Rivulus_luelingi:0.07857064384917285260,(Rivulus_janeiroensis:0.11035526212451324257,Rivulus_santensis:0.14791010638417484424)59:0.05660675156499050259)63:0.19668754166022964225,(Rivulus_rubrolineatus:0.14723842687965160714,Rivulus_ophiomimus:0.07971580932959612675)25:0.01337847649777640716)8:0.02301218532070636522,Rivulus_jucundus:0.13589299119211661893)19:0.07005844988537032747,((Rivulus_immaculatus:0.10780991584190621757,Rivulus_amphoreus:0.09479803428282534972)35:0.03674513543339615745,(Rivulus_deltaphilus:0.02764427449756584262,Rivulus_stagnatus:0.04110746372253705483)92:0.04558887509128619481)74:0.16366477768774800872)6:0.02844024996560405930)0:0.04524550829156430420,((Rivulus_violaceus:0.12873324245217421247,(Rivulus_apiamici:0.03088612652459596375,Rivulus_punctatus:0.03874674298203926309)74:0.02071154627832018716)69:0.21206100832322671845,((Rivulus_hildebrandi:0.19106290005852655245,((Rivulus_uroflammeus:0.09758251134617658418,(Rivulus_birkhani:0.15019268893034556922,((Rivulus_isthmensis:0.04000455080248599415,Rivulus_fuscolineatus:0.05664216044476175216)73:0.14347653459383932772,Rivulus_weberi:0.06667637501021324298)38:0.03662653291358531488)44:0.08207541468714610322)21:0.04390171547522973838,(Rivulus_chucunaque:0.21119556090170948925,Rivulus_magdalenae:0.09348996573243298358)58:0.10341835404435791024)39:0.11974725854080958687)15:0.07914837798328312746,(Rivulus_roloffi:0.19053975327355524017,Rivulus_tenuis:0.01216386045552144725)2:0.16987123121865274622)1:0.05285910677396673391)0:0.04751131247694399123)0:0.04035453304154009574)0:0.05188371467242266361,Rivulus_lyricauda:0.31516664918194231548)0:0.08684694041285405397,(((Leptolebias_minimus:0.44718920176438575753,Leptolebias_citrinipinnis:0.38620253491915390587)16:0.01874057490296634695,(Leptolebias_aureoguttatus:0.27890639901288405422,(((Cynolebias_costai:0.18213026466514981028,Nematolebias_whitei:0.34343331222404654968)54:0.03116203884239647504,(Cynolebias_antenori:0.09035018848917751588,(Simpsonichthys_mediopapillatus:0.03216575822394147216,(Simpsonichthys_flavicaudatus:0.02145557062298411588,(Simpsonichthys_guanambi:0.02171087409352791958,(Simpsonichthys_flagellatus:0.00266948601063047870,Simpsonichthys_janaubensis:0.00938711236193787331)63:0.00357058279040563600)73:0.00304316607535364729)100:0.09161716039680534163)76:0.02932937537288608787)94:0.16238842829843033733)73:0.10891978607591500106,Cynolebias_affinis:0.21800521759937127597)58:0.13847056618026323815)19:0.02707412679908000747)12:0.01449727494966154655,Kryptolebias_marmoratus:0.38940067604377565091)0:0.10253844973879872038)0:0.14276762889445968052)0:0.04580529367506178118)0:0.06503255260195320298,((((Labracinus_lineatus:0.06818662131487870925,(Rivulus_occellatus:0.00000176456401954691,Labracinus_cyclophthalmus:0.00000176456401954691)0:0.00609614468474481506)0:0.44865292871172812728,(Antennablennius_bifilum:0.32930607421724411576,Pseudomugil_gertrudae:0.46838959476585584341)6:0.07082623167146197274)0:0.03501290412765048488,((Trichopsis_vittata:0.38785370245832090363,(Betta_ocellata:0.24963586090711611942,((Betta_smaragdina:0.10515471628880489197,(Betta_imbellis:0.06629245515689491386,Betta_splendens:0.14886207778768723031)91:0.13356472633155039298)94:0.27989830325969050628,(Betta_uberis:0.20896807580902354573,(Betta_albimarginata:0.19254705433742294596,(Betta_falx:0.12875811921770999313,(Betta_anabatoides:0.14011981574695023256,Betta_fusca:0.07106786083848544433)70:0.04185915695002688508)77:0.08689535339889244148)59:0.05860159172618277512)48:0.09330541582721131599)28:0.11694937423120649322)53:0.13922236040989366401)22:0.17190797339021834933,(((Rimicola_muscarum:0.34350573817876184357,Acyrtops_beryllinus:0.35366132693943747034)34:0.12838419268308490229,((((Hippocampus_biocellatus:0.03191133065880298603,Hippocampus_trimaculatus:0.10297132274362698345)78:0.03664200653217565551,(((Hippocampus_barbouri:0.08164685527165527201,((Hippocampus_subelongatus:0.01169628405973532036,Hippocampus_angustus:0.01970008221036418511)89:0.01050831449625358460,Hippocampus_comes:0.02543345054880889744)88:0.02972198868145593809)77:0.02846709576701837718,Hippocampus_histrix:0.10089415713147918685)72:0.05743026246482733216,(Hippocampus_abdominalis:0.16830218056192236542,(Hippocampus_guttulatus:0.11517435535540868075,(((Hippocampus_queenslandicus:0.00510858564901812626,Hippocampus_spinosissimus:0.01409110789859643539)90:0.04987244263446218018,((Hippocampus_zosterae:0.20795292894896177938,(Hippocampus_patagonicus:0.07372818208257873873,Hippocampus_erectus:0.04156907371113604394)94:0.10103796878268551940)10:0.02330777175573553808,Hippocampus_kelloggi:0.04085089906201843507)4:0.02332987460358351001)5:0.02414566611533062318,(Hippocampus_camelopardalis:0.23650186220668095727,(((Hippocampus_capensis:0.00239511818348689485,(Hippocampus_fuscus:0.00961418566820197197,Hippocampus_kuda:0.01897590784543803574)42:0.00411116611572289771)33:0.00576424408286485990,Hippocampus_algiricus:0.02070485729606042977)61:0.00677642229544763320,(Hippocampus_fisheri:0.03374175796390746496,(Hippocampus_reidi:0.00227588865819967640,Hippocampus_ingens:0.00000176456401954691)96:0.01895160313938071966)27:0.00273365754743999309)91:0.05523914450887314892)5:0.01613618510371653270)12:0.02886601040900261747)27:0.03958710830474621023)25:0.01816015931493631871)43:0.04037252888419774755)24:0.02280858686475478736,Hippocampus_mohnikei:0.10655046875159289443)80:0.31883869896076255657,((Syngnathoides_biaculeatus:0.58022800034839228100,Solegnathus_dunckeri:0.16420460353523563812)13:0.13290438529085832342,((Hippichthys_cyanospilos:0.47107779433597790630,(Scriptaphyosemion_geryi:0.00000176456401954691,Bryx_dunckeri:0.22494370326375473512)4:0.14762922304544995322)2:0.11258490129911256916,(((Syngnathus_leptorhynchus:0.00228507318867841435,Syngnathus_californiensis:0.00000176456401954691)99:0.04393233356033567766,Syngnathus_auliscus:0.09057210150441710739)95:0.22747603003214125761,((Syngnathus_floridae:0.08377279834308508366,Cosmocampus_elucens:0.00477884960423129689)97:0.10945645067101643366,Syngnathus_acus:0.12059317108927868067)95:0.10479131714038537881)79:0.14470219873539327815)2:0.06121069345644130494)0:0.06598629762869726023)0:0.08574103837456902022)0:0.05060254976498818058,(Entelurus_aequoreus:0.50480499443682846916,(Doryrhamphus_dactyliophorus:0.33100674776651939357,Doryrhamphus_pessuliferus:0.08067475789102625250)99:0.31169088269869948959)51:0.20903914086609412526)0:0.03882016550082004885)0:0.05180529040163251581)0:0.04505290331108441348,((((((Pagothenia_borchgrevinki:0.05471212897885784771,(Trematomus_newnesi:0.06997284348003059151,((Trematomus_pennellii:0.04120229606606148898,((Trematomus_eulepidotus:0.05185925785680428612,(Trematomus_hansoni:0.04608749177292396221,(Trematomus_vicarius:0.00000176456401954691,Trematomus_bernacchii:0.00000176456401954691)100:0.02274080242195383739)37:0.00961088589777165520)19:0.00880576525068785601,((Trematomus_scotti:0.10076484492095928724,Trematomus_tokarevi:0.03907783077057395277)41:0.01281304909173821768,(Trematomus_lepidorhinus:0.00130560924678328272,Trematomus_loennbergii:0.00801876303390621457)99:0.04461510001191233699)9:0.00520429915296242995)23:0.00480344431025425418)23:0.00776514654515404659,Trematomus_nicolai:0.05232076731647374718)16:0.01670145068691212750)15:0.01271900578903932197)34:0.02362982358105433778,(((Patagonotothen_tessellata:0.00405371010995411295,Patagonotothen_guntheri:0.00417637698119143093)43:0.00537927661993830436,Patagonotothen_ramsayi:0.00945448356098071985)99:0.07942397962750448459,(Lepidonotothen_squamifrons:0.07022790042209686723,(Lepidonotothen_larseni:0.04360907411325392474,(Gobionotothen_marionensis:0.00676953113157586963,Lepidonotothen_nudifrons:0.00993855574922371428)100:0.06362292173778606730)97:0.05081107915954502507)71:0.00707549894970996383)95:0.07117554823906835360)92:0.10431568203707505815,(((((Acanthodraco_dewitti:0.05824411008148496632,(((Artedidraco_skottsbergi:0.01667345047316053791,(((Histiodraco_velifer:0.01137708427107910446,(Dolloidraco_longedorsalis:0.00442685752963190970,Artedidraco_shackletoni:0.00901143800456361173)34:0.00000176456401954691)74:0.00513800175931370794,Artedidraco_orianae:0.01556738870047007922)61:0.00515153895788224726,(Pogonophryne_scotti:0.01732224112994502907,Artedidraco_lonnbergi:0.01518673771855727321)40:0.00370758735835744705)35:0.00447232214884797554)100:0.05518449783436132211,((((Bathydraco_marri:0.00000176456401954691,(Bathydraco_macrolepis:0.00221358153821346040,(Bathydraco_joannae:0.00000176456401954691,Bathydraco_antarcticus:0.00443461787022718292)36:0.00000176456401954691)83:0.00443285405674394804)86:0.00726784410548913292,Akarotaxis_nudiceps:0.04384652508326442044)86:0.02208054863254997266,(Vomeridens_infuscipinnis:0.02240915870013242966,Racovitzia_glacialis:0.02187231958162785123)50:0.00856187807371065757)40:0.00280072829748876295,Prionodraco_evansii:0.06017983734985519334)75:0.03795620296904227142)27:0.00468461036955445873,(((Parachaenichthys_charcoti:0.05721933612496166860,Cygnodraco_mawsoni:0.02235024794558427771)65:0.01082191564601572321,Gerlachea_australis:0.04792482262241787999)59:0.00981226452559942619,Gymnodraco_acuticeps:0.04079579570940346234)43:0.00858760870685231284)16:0.00603363085667773212)31:0.01434819360972443454,(Champsocephalus_gunnari:0.09985013956211045982,(((Chaenocephalus_aceratus:0.02506597403718012484,(Chionobathyscus_dewitti:0.01644145882547465992,Cryodraco_antarcticus:0.00850532376401357329)44:0.00276945920364620292)21:0.00189177869855136670,((Chionodraco_hamatus:0.01280041151583012453,(Chionodraco_myersi:0.01123864005243677307,Chionodraco_rastrospinosus:0.01550065796695589831)33:0.00274852756232615630)44:0.00709019225136988621,Chaenodraco_wilsoni:0.02619690507787307554)26:0.00227412674953751757)74:0.01585619603538613795,(Dacodraco_hunteri:0.04120062480367314012,((Pagetopsis_maculatus:0.02333835766542065662,Pagetopsis_macropterus:0.01103599232015246845)100:0.02497127989314213564,(Neopagetopsis_ionah:0.02400431468730059026,Pseudochaenichthys_georgianus:0.04252051467103184351)99:0.01847027760256659407)99:0.02066695742304723582)79:0.01423367515784291701)78:0.01472055472060531181)95:0.03745675950877688787)74:0.07043042657230658143,(Cryothenia_peninsulae:0.05029813392147313572,(Dissostichus_eleginoides:0.02981520858648564754,Dissostichus_mawsoni:0.02960446350938460841)95:0.04662410412107195756)69:0.05079663289505011170)15:0.01424329841976479903,(Pleuragramma_antarctica:0.10527444642484984350,Gobionotothen_gibberifrons:0.08329254747495346001)37:0.02203484379043816818)17:0.04286082601166453510,(Notothenia_rossii:0.03637436037830124591,Notothenia_coriiceps:0.03849492272035470730)99:0.05280169834669461160)34:0.06298787906724137575)69:0.13146692995063799514,Eleginops_maclovinus:0.30806807824245180383)54:0.13538276705664545330,(Plagiotremus_tapeinosoma:0.35824193191073860909,(Oryzias_sinensis:0.38294414505122675862,(Stathmonotus_gymnodermis:0.29940068762951976833,Stathmonotus_stahli:0.29855365539216649484)40:0.09922406149237271433)19:0.11452203051624884611)3:0.06086956561632735768)1:0.04969328779911184185,((Callionymus_decoratus:0.40176610923333400116,((Repomucenus_richardsonii:0.25292393512248317888,Callionymus_bairdi:0.28178545707136604115)45:0.07503532083887243243,((Draculo_celetus:0.45072858476974153596,(Callionymus_valenciennei:0.12642176338724375739,Bathycallionymus_kaianus:0.15006329123505132705)98:0.12409257906670093097)25:0.09378465050317255636,((Synchiropus_picturatus:0.04565882029146983018,Synchiropus_splendidus:0.14179006770229676815)91:0.16133477644474419899,(Dactylopus_dactylopus:0.10668749833874904831,Synchiropus_stellatus:0.09191954371518054612)94:0.25060025378130956764)62:0.14422725241978434640)6:0.05219054437795526968)25:0.10986063939866549943)43:0.15577433999601056347,(Stathmonotus_culebrai:0.33566993622841645450,Gobiesox_maeandricus:0.35275655021174051607)33:0.18397124547387438964)0:0.00475632543831113527)0:0.03230719748562268373)0:0.03119877858540872836)0:0.05826480865257691738,(((((Macroramphosus_scolopax:0.09572171582583449689,Notopogon_fernandezianus:0.11624159184351856577)96:0.21965578734409538231,Centriscus_scutatus:0.25760918647770730816)6:0.04575363227588116755,(Amphistichus_rhodoterus:0.15535281718699806008,((((Cymatogaster_aggregata:0.00000176456401954691,Hyperprosopon_anale:0.00219822095730192813)100:0.05953923220512955666,Hysterocarpus_traskii:0.15836668894518782236)90:0.04074110803307264689,Micrometrus_minimus:0.21667706107273035365)85:0.03912940483743040931,((Embiotoca_jacksoni:0.05948029301402682489,(Brachyistius_frenatus:0.08064611733953050421,((Embiotoca_lateralis:0.09096512040021993706,Rhacochilus_vacca:0.02558080312453013885)40:0.00000176456401954691,Phanerodon_atripes:0.04385109095061745510)72:0.02667606166620009600)77:0.04597178136887566241)95:0.03016713872340580707,(Neoditrema_ransonnetii:0.09986185652539861235,Ditrema_temminckii:0.08152800285279906967)100:0.11297535606473281533)73:0.02704788227856547156)95:0.15271356055806920016)98:0.27633898928094124958)5:0.06454767120163275307,(((Tripterygion_tripteronotus:0.12524587938494374795,((Tripterygion_delaisi_delaisi:0.11350317813787330623,Tripterygion_delaisi_xanthosoma:0.03633149877875598011)94:0.10690434711590129924,Tripterygion_melanurus:0.12981523251313287215)45:0.02399797354361571725)92:0.24776803096422039818,Starksia_starcki:0.47639638989604587982)0:0.08423347491461445624,(Opistognathus_aurifrons:0.26465276870897874728,(Opistognathus_rosenblatti:0.24699853186825507234,Opistognathus_punctatus:0.36564724795680314529)17:0.06133577881983162672)9:0.03267803028831994805)0:0.02359782416273320527)0:0.06190751737506880459,((((((Gasterosteus_wheatlandi:0.26439687279645268658,((Pungitius_pungitius:0.06650168582533787232,Pungitius_kaibarae:0.09570449798134114672)92:0.11482957944512066362,Culaea_inconstans:0.32120568509181618433)72:0.10112007461726105206)12:0.05964942890415626114,Apeltes_quadracus:0.20057869472686445933)68:0.20488264702923478233,Aulorhynchus_flavidus:0.23978002741209122894)23:0.14519471406624459564,Diplectrum_formosum:0.29322285959933885868)2:0.05713649632999823202,(Serranus_tabacarius:0.27204747343309826357,(Sebastapistes_strongia:0.43675979539049947231,(Aplocheilichthys_hutereaui:0.39222229011682824273,(Petroscirtes_breviceps:0.00437131393761079276,(Cyclichthys_orbicularis:0.01942891125391480941,Petroscirtes_variabilis:0.00740126574678478220)1:0.00000176456401954691)100:0.37023010636720998789)2:0.03065555450277284918)4:0.08085972687199279030)1:0.04356755212990473525)0:0.02730704598160578131,((Hypoplectrus_puella:0.41059593469821931144,((Paralabrax_maculatofasciatus:0.02584212593466840915,((Paralabrax_auroguttatus:0.01797925437456193015,Paralabrax_clathratus:0.06648253761598410527)26:0.00343437526275556767,Paralabrax_nebulifer:0.02092202774404854429)66:0.02534110632022066462)79:0.11073006225898303356,(Serranus_cabrilla:0.14723775393596824634,(Serranus_hepatus:0.15413970359023163037,Serranus_scriba:0.12590405331560974855)48:0.04063222130184870884)14:0.02862257036280926523)43:0.11494286997404253026)13:0.00477018321097782017,(Serranus_baldwini:0.21143914522035281434,Serranus_tigrinus:0.46036949737812726813)60:0.19539590829799111105)6:0.06064378016101971275)0:0.09836702090998790393)0:0.00000176456401954691)0:0.05309271460844120633)0:0.02350693201423561737)0:0.02991592272682543768,((Calloplesiops_altivelis:0.43590424702574520621,(Acanthocepola_limbata:0.50736403048388656334,((((Pictichromis_porphyrea:0.04029876468749295920,(Pictichromis_dinar:0.06057074208459120590,(Pictichromis_paccagnellae:0.03466775035099777663,Pictichromis_diadema:0.02920236128108929727)76:0.01935948118120570799)43:0.01572768063977841857)96:0.06745889050569557621,Pictichromis_coralensis:0.08902895887888283288)99:0.30369719637514175359,((Pseudochromis_olivaceus:0.51755365407410680589,Pseudochromis_fuscus:0.17010459743332059790)31:0.05413822289712399288,Pseudochromis_natalensis:0.35637831542533443541)54:0.18744821844081072104)27:0.11825023348346161423,(((Quintana_atrizona:0.31835059594813064354,(Girardinus_denticulatus:0.12653288516495700411,((Girardinus_uninotatus:0.01685092332895292769,Girardinus_falcatus:0.04329854988018651263)90:0.04220596220320013692,(Girardinus_creolus:0.04163864150581636503,(Girardinus_microdactylus:0.06055324351415053430,Girardinus_metallicus:0.05094361599481073144)88:0.04725642871332282058)37:0.01122968666687990114)75:0.05973761252178309139)82:0.09631722136631343245)84:0.05110874658053183445,((((Poeciliopsis_pleurospilus:0.00000176456401954691,Poeciliopsis_gracilis:0.00000176456401954691)100:0.00258497124716891005,Poeciliopsis_catemaco:0.03201036261879514760)99:0.28898120684563133320,(Phalloceros_uai:0.02410089688344719400,Phalloceros_reisi:0.05900840058671994598)100:0.22005909877144377851)54:0.06558839277152488101,(Priapella_intermedia:0.12535265606457285381,((((Xiphophorus_alvarezi:0.02074700381097065519,Xiphophorus_hellerii:0.00925729498559320699)92:0.03842499435020452214,Xiphophorus_maculatus:0.05179651491059786284)93:0.09355116244427279237,((Limia_vittata:0.13279452653412246455,((Pamphorichthys_hollandi:0.11844799480056851515,Poecilia_reticulata:0.25893841536944978143)50:0.06609779362620638032,(Poecilia_mexicana:0.04369604458596461349,(Poecilia_petenensis:0.02658555984494292979,(Poecilia_sphenops:0.01239072334080037856,Poecilia_latipinna:0.01773734930638963536)92:0.02313039898015491896)59:0.00753393222558142693)86:0.06403840646217208443)25:0.02691242768961715076)91:0.12514625895767922392,Heterandria_bimaculata:0.15019398385019147590)55:0.08030768829338150172)19:0.00000176456401954691,(Belonesox_belizanus:0.18601023903812710647,((Gambusia_holbrooki:0.01137918032668412335,Gambusia_affinis:0.03351659928213850809)95:0.08088058125103536611,((Gambusia_yucatana:0.03221371082735723784,Gambusia_puncticulata:0.03970442717730800902)100:0.07399130441909451683,(Gambusia_sexradiata:0.14463666275623532353,(Gambusia_rhizophorae:0.03034244251036581977,Gambusia_punctata:0.04479325341836452867)99:0.04462208483856688196)44:0.01059968695894485981)28:0.00692955738723207218)99:0.24031156898320882309)80:0.12916659865305130106)26:0.05941327229537817639)15:0.03302439391177804801)20:0.05733960710183018394)52:0.03460294076219315451,Heterandria_formosa:0.26023793416950874446)74:0.16945822651699973815)0:0.06414757574777739391)0:0.05259376379029245818)0:0.04047572156311946695,(((Cirrhitops_fasciatus:0.17910940035649869362,Neocirrhites_armatus:0.17853973984698140454)84:0.14431748747924688647,((Oxycirrhites_typus:0.29081746722644558067,Cirrhitichthys_guichenoti:0.18107430401722579560)66:0.10575860590881697165,(Cirrhitichthys_falco:0.13556993679490542126,(Cirrhitichthys_oxycephalus:0.11908729291397850136,Cirrhitichthys_aprinus:0.28665215388172160527)60:0.04693905838082555737)64:0.04474979994009396778)27:0.06904711354504944332)4:0.04467985645341299161,(((((Siganus_argenteus:0.07485414012606690648,(Siganus_sutor:0.07424618272414421072,((Siganus_spinus:0.11165726145399666736,Siganus_vermiculatus:0.00000176456401954691)69:0.03819465454202782689,Siganus_fuscescens:0.01728062906235098076)43:0.00890640030802578551)24:0.02403474953305745343)36:0.06140626801316277639,(Siganus_javus:0.05830955687655414721,(Siganus_puellus:0.07232201143195668425,((Siganus_guttatus:0.03457828138147958641,(Siganus_corallinus:0.01856783358198075065,Siganus_virgatus:0.01459084721527753592)98:0.01551671772626261533)98:0.07703258320556084782,(Siganus_punctatus:0.03522200344385370552,(Siganus_vulpinus:0.00596438370507734912,Siganus_unimaculatus:0.00380108268131980169)100:0.04894152390533580005)97:0.02756477087693361486)59:0.01398145792840029510)37:0.01334348298625944243)96:0.12235648891283826267)92:0.14710273648225286047,(Amblycirrhitus_pinos:0.32647087798374974099,(Paracirrhites_forsteri:0.11093611341670693571,Paracirrhites_arcatus:0.17360770218495044648)55:0.04863501730519807775)52:0.08161421317802548814)3:0.00862941852449847745,(((Calotomus_zonarchus:0.24075332926007755874,Calotomus_carolinus:0.01530186760048500745)100:0.30231933994606741134,((((Scarus_vetula:0.04571079109453771161,(Scarus_chameleon:0.07822295084694592948,Leptoscarus_vaigiensis:0.04346534548750367877)69:0.01380237558780867367)73:0.04089327095994874095,(((Scarus_rubroviolaceus:0.02431837363299726301,(Chlorurus_gibbus:0.00264230638475540817,Scarus_ghobban:0.00186146044671525908)100:0.03668009317797501362)89:0.03485512901269046532,Scarus_iseri:0.06687270360735604602)73:0.01634524365369308813,Scarus_frenatus:0.05015504302954396781)74:0.03221040137987682406)84:0.07811162021401228672,((Nicholsina_usta:0.35696718808569960490,Cryptotomus_roseus:0.32416017015361842102)32:0.10313168452128526620,((Sparisoma_cretense:0.09154886586215266187,((Sparisoma_chrysopterum:0.03073709345759521872,(Sparisoma_rubripinne:0.07920512684188309172,Sparisoma_viride:0.04895371543321296731)39:0.00594946633592732636)62:0.03031270429152457813,Sparisoma_aurofrenatum:0.11998782835120415380)46:0.06206226110910456617)24:0.01455414843450633829,Sparisoma_radians:0.13540276715486057602)40:0.09583506814503893467)45:0.12254893299176007615)19:0.06115637056318273118,Cetoscarus_bicolor:0.14472831216965356682)27:0.07297315126537255725)44:0.13504550400588821724,(Oxycheilinus_digramma:0.33168269174289338741,((Oxycheilinus_bimaculatus:0.19925668525896042538,(Cheilinus_oxycephalus:0.10667014677274958001,Cheilinus_chlorourus:0.07762974552999733768)99:0.09062164906322744962)82:0.07380551267435528362,(Cheilinus_undulatus:0.25404952823223958536,Cheilinus_fasciatus:0.13282412445844993254)91:0.10912130444392069417)76:0.05125854175297183540)62:0.06880066623014037541)3:0.02424181784525133304)0:0.04731188700807843067,(((Owstonia_weberi:0.43387891459325889087,Pseudodax_moluccanus:0.25623636023813939344)5:0.09721697328812481387,(((((Bodianus_rufus:0.07422981877594009292,Bodianus_pulchellus:0.08625920697804931314)97:0.19290656143072221207,(((Bodianus_scrofa:0.18236741353748830985,Semicossyphus_pulcher:0.12880381062051410312)66:0.05149860633529056847,(Bodianus_axillaris:0.26503755472460949205,(Bodianus_tanyokidus:0.17547814804043951331,Bodianus_anthioides:0.12684221594472949723)10:0.00000176456401954691)5:0.01424882589668982451)12:0.02697951274065819166,(Clepticus_parrae:0.26884100087608509000,(Bodianus_perditio:0.28369533865686064056,Bodianus_speciosus:0.13014611412361937193)79:0.06703772207835577424)14:0.01837502166529846048)21:0.02081238839676563188)65:0.05605214465790454847,((Bodianus_oxycephalus:0.02321906908606997944,Bodianus_unimaculatus:0.06078880035618966504)100:0.12826241681162051100,Achoerodus_viridis:0.19245269874622103479)43:0.03399511238371185490)44:0.07567181442324706631,((Choerodon_venustus:0.20867411360421153632,((Choerodon_schoenleinii:0.06024139497805475890,Choerodon_rubescens:0.02903747802286626295)98:0.09276562078203900996,Choerodon_cephalotes:0.11228878477266387992)73:0.03151976536914487259)80:0.05151151181743311713,Choerodon_azurio:0.26761783898539809323)54:0.10911072502733833167)9:0.02856367043397219432,((Anchichoerops_natalensis:0.34543396512746249449,(Novaculichthys_taeniourus:0.25936020575659662901,((Xyrichtys_splendens:0.18871360876107182158,(Xyrichtys_martinicensis:0.08593777367933549960,Xyrichtys_novacula:0.08064375401098064500)95:0.07919370324747296297)72:0.07298133808664881983,Iniistius_pavo:0.20437511040188513500)83:0.05840090017692717295)15:0.05689482435841925617)0:0.03821675460655653278,((Paracheilinus_mccoskeri:0.04945941887440010593,Paracheilinus_carpenteri:0.04956393216681035524)98:0.45250149378950510481,(((Lappanella_fasciata:0.22624115677163261773,(Cichlasoma_bimaculatum:0.13801623974691368257,((Labrus_viridis:0.00000176456401954691,Labrus_merula:0.00215690154245629139)100:0.01356331299554085039,Labrus_bergylta:0.00894704910346550905)100:0.11005460700790402340)82:0.07497311185418316370)70:0.04796645577261215293,(Tautoga_onitis:0.08188010541734568049,(Ctenolabrus_rupestris:0.11628937270496701728,(Acantholabrus_palloni:0.13711718941479478229,Tautogolabrus_adspersus:0.03788121732289863319)58:0.02445977067014699566)88:0.07774109908761832333)71:0.08088629858561915964)55:0.05331736673360322476,((Centrolabrus_exoletus:0.00790930578417274946,Symphodus_melanocercus:0.00204313897688470063)99:0.12444905796487416849,(Symphodus_bailloni:0.13222249773818861573,(((((Symphodus_melops:0.00000176456401954691,Symphodus_roissali:0.00218210432307361195)100:0.07738247195571748560,(Symphodus_cinereus:0.08795224947881882804,Symphodus_mediterraneus:0.06843124921292818852)11:0.00631797837605502666)11:0.00643525724677171916,Symphodus_rostratus:0.06082842627759354331)20:0.01825853861992626434,Symphodus_tinca:0.09301103273180871822)32:0.02336747246330910313,Symphodus_doderleini:0.07639847195052663908)53:0.04735922245225104854)91:0.13502138588940218455)86:0.11586966599423596802)57:0.05107807829643289110)1:0.05117599706615764271)0:0.00000176456401954691)0:0.02084543215747815259)0:0.01141613384326855586,(((((((((Halichoeres_lapillus:0.21315149681138878535,(Macropharyngodon_kuiteri:0.16990291491933853796,(Macropharyngodon_bipartitus:0.11678017213948348185,((Macropharyngodon_choati:0.12673793606152222724,((Macropharyngodon_cyanoguttatus:0.02974260706891069042,Macropharyngodon_ornatus:0.02230763151764994165)99:0.06760698978115968483,(Macropharyngodon_geoffroy:0.05812143239777974729,Macropharyngodon_meleagris:0.01228240834243734397)100:0.05994786905724082404)97:0.05295831853953277685)84:0.05034993218147035265,Macropharyngodon_negrosensis:0.14219959001029680579)61:0.02469998597424634401)91:0.07056972774685120464)65:0.04968500667573277213)57:0.04074154707285172761,((((Halichoeres_leucoxanthus:0.07193675698427298704,Halichoeres_chrysus:0.04858996214517951467)100:0.11784824291254566986,(Halichoeres_trispilus:0.08961794389644038572,(Halichoeres_melasmapomus:0.08788591591059412289,Halichoeres_ornatissimus:0.08664965675275716761)72:0.04978510354893200124)46:0.03096567298213433531)53:0.02882736854986985009,Halichoeres_biocellatus:0.13084839662367461766)92:0.15462482092313906135,(((((Coris_pictoides:0.17524906849003790699,Halichoeres_prosopeion:0.03854227945458352927)52:0.05390428790599192249,Halichoeres_solorensis:0.22521585211137951088)24:0.00358466629092542245,Coris_aurilineata:0.17463370045995277313)96:0.14040948648505607865,((((Halichoeres_argus:0.23411070030910921536,Halichoeres_richmondi:0.17818613593311330434)45:0.06859680108739216187,(Halichoeres_nigrescens:0.24723842041258187185,(Halichoeres_papilionaceus:0.15612107312694625816,Halichoeres_binotopsis:0.15125407778485272514)49:0.02115382541089454518)29:0.01274650207443885769)11:0.00000176456401954691,(Halichoeres_marginatus:0.02340427904236441065,Halichoeres_leucurus:0.04313868162567673775)97:0.15671204151823456274)97:0.15375939422597159623,Halichoeres_brownfieldi:0.27539701115950260268)30:0.04667051598218398267)11:0.03684696981434883167,((Coris_batuensis:0.14715368457377861477,(Halichoeres_podostigma:0.27267748989874696086,Halichoeres_chloropterus:0.09854495790622627704)85:0.07652261040713687068)73:0.05395287117918820541,(Halichoeres_margaritaceus:0.11687431141977587534,Halichoeres_nebulosus:0.18627429246045276723)89:0.11837445548172184639)48:0.06965179171843993711)7:0.02027769263639359401)7:0.02948936699098935257)11:0.02023456687227800227,Halichoeres_maculipinna:0.25253825188014389358)4:0.03779718565668204605,(((((Anampses_lineatus:0.06157516989103491156,Anampses_meleagrides:0.06436985081789586838)88:0.05496052614288782223,(Anampses_twistii:0.09552620767068915886,Anampses_neoguinaicus:0.06109430603854373171)84:0.05255521702920892130)88:0.07136040837454359076,Anampses_caeruleopunctatus:0.14978755130599308298)80:0.08136069408634678812,Anampses_geographicus:0.18661428870879775976)90:0.14095874897131074888,((((Coris_julis:0.04556060117863063030,Coris_atlantica:0.05115834861740811701)100:0.22975366110668879460,Coris_dorsomacula:0.14846483055807010998)62:0.05578340573026625837,(((Coris_gaimard_africana:0.00376659562881386835,Coris_gaimard:0.00725224744953029393)100:0.07260145056415716336,(Pseudocoris_bleekeri:0.13737331516926512553,(Coris_formosa:0.00000176456401954691,Coris_frerei:0.00661443674760561588)97:0.00423112285107573021)47:0.03528779101278030350)95:0.08255316101766201065,(Pseudocoris_heteroptera:0.06088270182178561613,Pseudocoris_yamashiroi:0.05603790499503129990)100:0.12380760551492732413)80:0.04664483145544370130)32:0.02832622355750333276,(Coris_picta:0.16003058646075757343,(Coris_aygula:0.07695871307899233849,Coris_flavovittata:0.08626475584711119915)93:0.04652381594113045443)71:0.05073916712078008900)54:0.04169852329528501989)6:0.01345576119673372491)0:0.02450709160249412571,(((Hemigymnus_fasciatus:0.05432408071565938273,Hemigymnus_melapterus:0.12745580269376216576)99:0.22680720811057414332,(Halichoeres_hortulanus:0.17897802433058920824,(Halichoeres_scapularis:0.06536412784230302697,Halichoeres_trimaculatus:0.12827079767140556199)99:0.15918936910680120400)55:0.07795535688546338660)6:0.04851272645302313891,(((Labroides_phthirophagus:0.15387008698054241429,Labroides_dimidiatus:0.14973157734499320526)98:0.15993322623631014978,(Labrichthys_unilineatus:0.07591160911397983047,Halichoeres_miniatus:0.10235456094787018233)99:0.13836896745119575680)20:0.04855049939032085593,((Halichoeres_zeylonicus:0.08345082883879322011,Halichoeres_hartzfeldii:0.02903427024311027935)100:0.17996413171953612453,(Cheilio_inermis:0.36027513533604627050,(Parajulis_poecilepterus:0.17780094480539146429,Leptojulis_cyanopleura:0.11577256243845812644)97:0.14088252757361935386)6:0.04627046609825880702)4:0.03422942376733825731)0:0.02820082386358280582)0:0.00755382703365124319)0:0.00667647454625308265,((Oxyjulis_californica:0.07287726294129460991,Halichoeres_semicinctus:0.02235199828297594268)100:0.20800862390038196703,((Halichoeres_socialis:0.13155840489045261821,(Halichoeres_dispilus:0.07130259151225552816,Halichoeres_pictus:0.11148717744930886075)81:0.04705619424517124005)95:0.11858098695753568896,(Halichoeres_notospilus:0.05684963971333838878,(Halichoeres_bivittatus:0.07200042982156440452,((Halichoeres_nicholsi:0.05319517521211566957,Halichoeres_poeyi:0.05419043703646785753)11:0.01484822204487272652,(Halichoeres_radiatus:0.05265866501527388666,Halichoeres_garnoti:0.07704678458677573227)42:0.01133376794596074010)2:0.00000176456401954691)61:0.05243010004250422268)88:0.05431336134649716874)76:0.06020490110358059366)45:0.05121292589261018657)0:0.03342019476111220133,(Istiblennius_edentulus:0.38965285343446925959,(Hologymnosus_doliatus:0.00000176456401954691,Hologymnosus_annulatus:0.01509435575623526045)100:0.23312457947358283805)5:0.09126232891468313846)0:0.00916389605597061480,((Stethojulis_interrupta:0.26845241202783909351,(Stethojulis_trilineata:0.24351795063688777554,(Stethojulis_balteata:0.01514650125461571885,(Stethojulis_bandanensis:0.01372993440349369598,Stethojulis_albovittata:0.03027535696971047838)37:0.00397278078345124786)55:0.07639023649204694510)64:0.09475905351953030353)87:0.15950224556820138955,(Doratonotus_megalepis:0.36100570927540975852,(Notolabrus_tetricus:0.07617893973478342962,Notolabrus_gymnogenis:0.04424907029382949691)100:0.22332325168982636310)10:0.05881169105567273653)0:0.01810028715668089011)0:0.01858344313713393078,(Thalassoma_ballieui:0.20467015099932081568,((((Gomphosus_varius:0.08977314689292358041,(Thalassoma_lunare:0.10555058908201184198,(Thalassoma_lutescens:0.00427886103117981840,(Thalassoma_duperrey:0.01124885925303535129,Thalassoma_genivittatum:0.00224871492598817955)40:0.00000176456401954691)99:0.05248939605454722851)39:0.02069134304578371802)22:0.01253930635110872376,(Thalassoma_quinquevittatum:0.07709977581314041151,(Thalassoma_hardwicke:0.02691471292289326564,Thalassoma_jansenii:0.04501490051584965124)97:0.02823666354974457143)82:0.03769682264329673321)13:0.00967538793880954803,Thalassoma_pavo:0.08505440560933774652)32:0.02832357797900670465,Thalassoma_bifasciatum:0.10851787939780475900)63:0.04424199804679524589)95:0.17267813254589178817)0:0.00862073863240800375)0:0.02540148862449830014)0:0.03406252308790935074)0:0.00579261845734155550)0:0.01356823412019069787)0:0.01636541539673064607,(((Protogobius_attiti:0.22607246010890907906,Rhyacichthys_guilberti:0.20396284991673041542)49:0.08795537352355246863,(Corythoichthys_intestinalis:0.76071683073118556706,(Fistularia_tabacaria:0.32129095242942490307,(Fistularia_petimba:0.23031817971131979261,Fistularia_commersonii:0.19841578953618208003)95:0.17554307528758281154)64:0.09514900600957057475)5:0.12683385458249057942)3:0.02037648685521335074,(((Sphoeroides_pachygaster:0.29565905896792227248,(Sphoeroides_spengleri:0.07767707362824161077,(Sphoeroides_annulatus:0.02588876489698855102,Sphoeroides_testudineus:0.01951461181582524879)100:0.13911677668452332446)85:0.05334679583752695992)59:0.05223357676483626061,(((Lagocephalus_lunaris:0.17850797958981406977,Lagocephalus_inermis:0.20938991351243452232)74:0.05568829576253692126,(Lagocephalus_lagocephalus:0.08964119825363534022,((Dactyloptena_orientalis:0.00268847923997147164,Lagocephalus_guentheri:0.00588493330921536443)99:0.05482956896980957506,(Lagocephalus_wheeleri:0.00000176456401954691,(Lagocephalus_spadiceus:0.00000176456401954691,Lagocephalus_gloveri:0.00095325266611290249)65:0.00000176456401954691)100:0.03878315183394494270)93:0.05190907119298138889)100:0.09940397713805022850)90:0.10765624934142557778,((Canthigaster_solandri:0.07188534389092229859,((Canthigaster_valentini:0.07345950151917038373,(Canthigaster_bennetti:0.09507227915554082587,Canthigaster_amboinensis:0.06457157109570138553)30:0.01189381236880963748)17:0.00576194684741926366,((Canthigaster_rostrata:0.01974070433579746750,Canthigaster_rivulata:0.01558770048347186792)96:0.02912956115723024098,Canthigaster_coronata:0.10766400283022881101)22:0.00705287624120390279)49:0.03264908258777745459)91:0.17210511231923189546,(((Arothron_immaculatus:0.04973018624171020308,Arothron_nigropunctatus:0.06338951689835539693)95:0.03489380075125392278,Arothron_meleagris:0.03073946338457887381)55:0.01643459150609812014,Arothron_hispidus:0.02164729006369225592)90:0.09940746195619071246)95:0.14006879257311843912)35:0.03881853298392698437)35:0.06088194809400229723,(Takifugu_oblongus:0.02538305247731644995,((Takifugu_fasciatus:0.00472249202045030118,Takifugu_obscurus:0.00000176456401954691)98:0.01722892594951644027,(((Takifugu_vermicularis:0.03257140940714151056,Takifugu_xanthopterus:0.01752883677998597414)24:0.00406720800121025249,(Takifugu_poecilonotus:0.03670947850610115082,Takifugu_pardalis:0.02342228239601030007)11:0.00222168265276535696)9:0.00240671083182563989,(Takifugu_niphobles:0.01955161449208935726,Takifugu_stictonotus:0.02749777462871240419)47:0.00576612173458658332)36:0.00733235687072266058)55:0.02153159047022137548)100:0.35119749307499414925)53:0.06092361088566205368)4:0.08779393088332071438)0:0.01276514499725306222)0:0.01203210859473827568)0:0.04993425744740853656,(Centropristis_striata:0.41861893622601081821,Cottoperca_gobio:0.31098435525202827723)3:0.07483710924689401800)7:0.06573902581658146171)29:0.07998378585256379902,Beryx_splendens:0.29157948434947889016):0.45000000000000001110,Polymixia_japonica:0.45000000000000001110);

Figure 2: Cytb tree
[truncated: 413,118 more chars]
